# Unraveling the Catalytic Mechanism of β‑Cyclodextrin in the Vitamin D Formation

**DOI:** 10.1021/acs.jcim.3c02049

**Published:** 2024-04-10

**Authors:** David Ferro-Costas, Pedro A. Sánchez-Murcia, Antonio Fernández-Ramos

**Affiliations:** † Departamento de Química Física, 16780 Universidade de Santiago de Compostela, 15782 Santiago de Compostela, Spain; ‡ Laboratory of Computer-Aided Molecular Design, Division of Medicinal Chemistry, Medical University of Graz, Neue Stiftingtalstraße 6/III, A-8010 Graz, Austria; § Institute of Theoretical Chemistry, University of Vienna, Währinger Straße 17, 1090 Vienna, Austria; ∥ Centro Singular de Investigación en Química Biolóxica e Materiais Moleculares (CIQUS), Universidade de Santiago de Compostela, 15782 Santiago de Compostela, Spain; ⊥ BioTechMed-Graz, Mozartgasse 12/II, 8010 Graz, Austria

## Abstract

Previous experimental studies have shown that the isomerization reaction of previtamin D3 (PreD3) to vitamin D3 (VitD3) is accelerated 40-fold when it takes place within a β-cyclodextrin dimer, in comparison to the reaction occurring in conventional isotropic solutions. In this study, we employ quantum mechanics-based molecular dynamics (MD) simulations and statistical multistructural variational transition state theory to unveil the origin of this acceleration. We find that the conformational landscape in the PreD3 isomerization is highly dependent on whether the system is encapsulated. In isotropic media, the triene moiety of the PreD3 exhibits a rich torsional flexibility. However, when encapsulated, such a flexibility is limited to a more confined conformational space. In both scenarios, our calculated rate constants are in close agreement with experimental results and allow us to identify the PreD3 flexibility restriction as the primary catalytic factor. These findings enhance our understanding of VitD3 isomerization and underscore the significance of MD and environmental factors in biochemical modeling.

## Graphical abstract

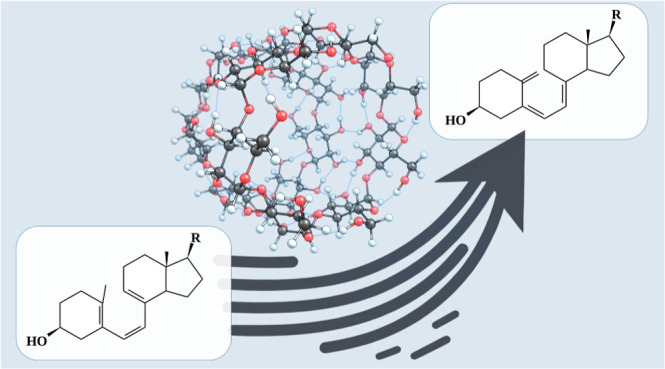

## Introduction

The “sunshine vitamin”, vitamin D3 (VitD3), is a steroid hormone hailing from cholesterol that holds a crucial place in the maintenance of calcium homeostasis and the enhancement of normal bone mineralization within the human anatomy.^1^ This vital nutrient is also intricately involved in several other indispensable biological processes,^2–4^ and its insufficiency has been identified as a contributing factor to various health conditions, such as rickets and osteoporosis in children and adults,^1^ autoimmune diseases,^5–7^ and even to the onset of depression in later life.^8,9^ Despite its fundamental significance, hypovitaminosis D is a widespread global challenge, affecting approximately one billion individuals across the world, even in regions boasting high levels of sun exposure.^10^

VitD3 can be obtained by humans through two sources, namely, endogenous production and dietary intake, with the former serving as the principal source for the vast majority of individuals. Regarding the latter, the utilization of VitD3 supplements has witnessed an unprecedented surge in recent years. For example, their sales reached a staggering $936 million in the United States alone in the year 2017, a 9-fold increase from the previous decade.^11^ Given the growing demand for VitD3, an in-depth comprehension of the mechanism behind its formation holds immense significance for both the pharmaceutical and food industries, as it holds the potential to pave the way for the creation of more efficient and economically viable synthetic procedures.

The endogenous biosynthesis of VitD3 within the skin involves a two-step process, as depicted in Figure 1.^12^ The first step is the UV-induced 6π electrocyclic ring opening of 7-dehydrocholesterol, otherwise known as provitamin D3 (ProD3), resulting in the formation of the secosteroid previtamin D3 (PreD3). The second step entails the thermal antarafacial [1,7]-sigmatropic hydrogen shift reaction, culminating in the formation of the much-coveted VitD3. In order for this second step of the biosynthesis of VitD3 to occur, it is necessary for the single bonds of the triene moiety, which connect rings **A** and **C**, to be in the s-cis conformation (−c or +c, depending on whether the corresponding dihedral angle is between −90 and 0° or between 0 and 90°, respectively). As a result, this reaction manifests two potential antarafacial hydrogen exchange pathways: +c+c and −c−c, or just (+) and (−), as illustrated in Figure 2. The nomenclature for these arrangements is derived from the torsion angles about the two single bonds of the triene moiety (ϕ_1_ and ϕ_2_, see Figure 1).

**Figure 1:**
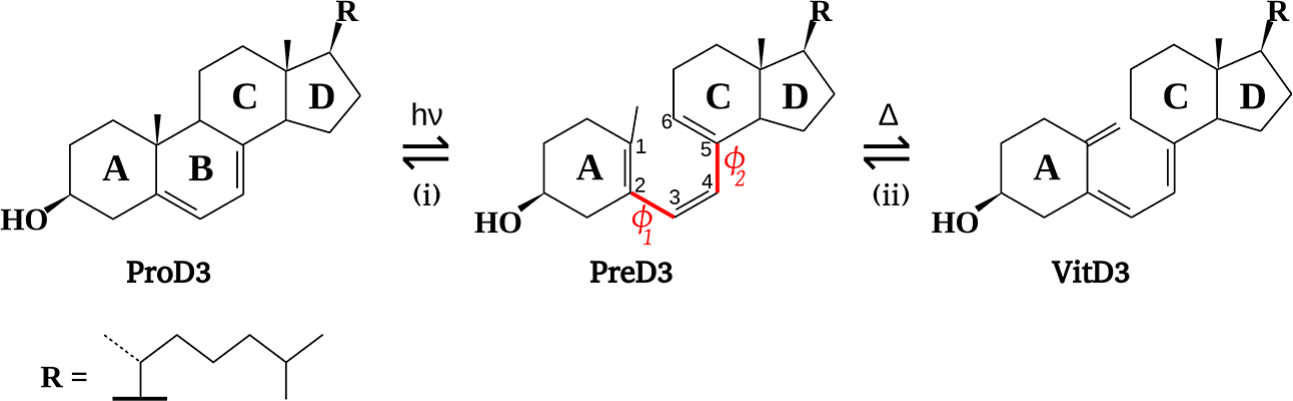
Reactions in the synthesis of VitD3: (i) electrocyclic ring opening and (ii) antarafacial sigmatropic hydrogen shift. Dihedral angles associated with the triene moiety in PreD3 are also shown: ϕ_1_ (1–2–3–4) and ϕ_2_ (3–4–5–6).

**Figure 2:**
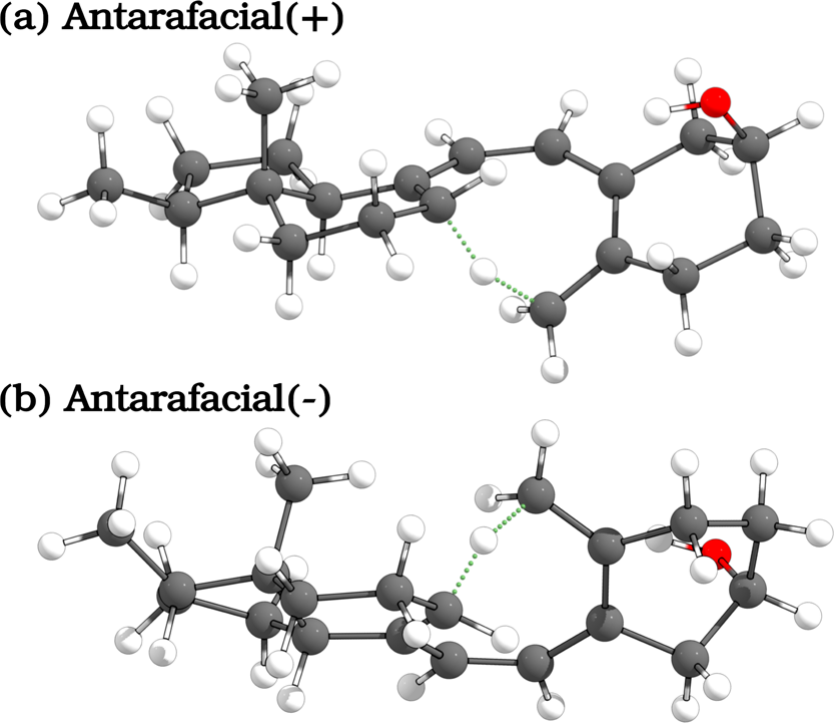
Transition states for the antarafacial H-exchanges in the thermal sigmatropic reaction. The H transfer is highlighted with green dotted lines.

According to the nonadiabatic molecular dynamics (MD) simulations carried out by Furche and co-workers,^13^ the UV-induced ring opening results in PreD3 in the −c−c arrangement (we refer to Figure 11 of their work). However, the small interconversion barriers in PreD3 allows the thermal isomerization to take place through the +c+c configuration, which is known to be about 10 times faster than the −c−c pathway.^14^ It is also important to note that the most stable conformer of PreD3, which has been found^14,15^ to present a +t−c triene configuration, does not correspond to either of the two reactive triene arrangements (+t implies that the corresponding dihedral angle, in this case ϕ_1_, is between +90 and +180°). Examples of these triene configurations can be found in Figure 3.

**Figure 3:**
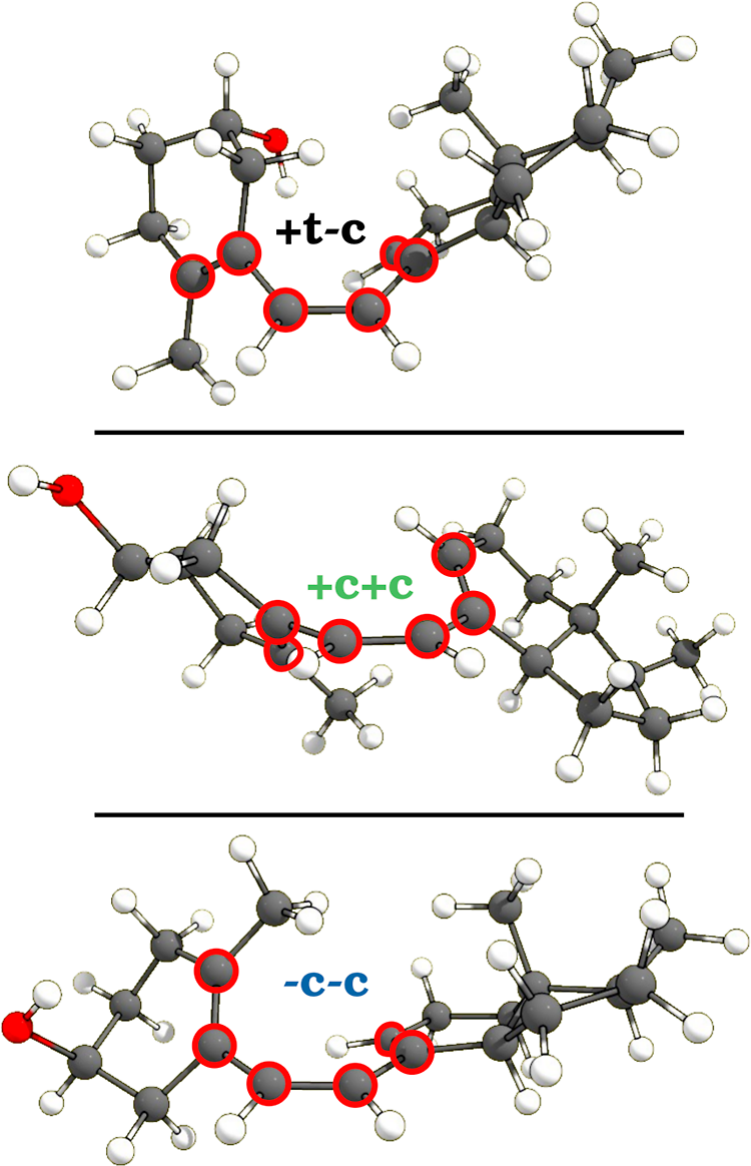
Examples of nonreactive (NR) +t−c and reactive +c+c and −c−c triene configurations. Atoms associated with the triene moiety are highlighted.

Holick and co-workers have delved into the thermal isomerization of PreD3 in a range of media. Their findings show that the thermal reaction necessitates a substantial amount of time, spanning several days, in an isotropic organic solution, such as *n*-hexane, at a temperature equivalent to that of the human body.^12^ However, they have uncovered that this transformation occurs at a 10-fold faster pace when taking place inside different skin types, including human^16^ and even reptilian skin.^17^ Their experiments^18^ show a remarkable acceleration in the rate of this thermal isomerization—by 40 times—when PreD3 remains encapsulated within a β-cyclodextrin dimer (β-CD dimer),^19^ compared to its rate in an isotropic medium. In this specific experiment, ProD3 was first encapsulated inside the β-CD dimer and then irradiated to produce PreD3. Subsequently, the thermal isomerization of PreD3 to VitD3 was studied. Given the high excess of β-CD and the insolubility of free ProD3 in water, Holick and co-workers reported that the system (ProD3, PreD3, and VitD3) remained fully complexed throughout the experiment. Therefore, the observed 40-fold increase in the reaction is exclusively due to the isomerization reaction and is unaffected by the encapsulation and decapsulation processes.

To explain the catalytic effect of the β-CD dimer, Holick and co-workers proposed that the reactive conformers of PreD3 are stabilized upon complexation, thereby elevating the isomerization rate constant. On the other hand, Meana-Pañeda and Fernández-Ramos posited that this increase is a result of the diminutive cavity of the β-CD dimer, which restricts rotations about the single bonds of the triene moiety connecting the **A** and **C** rings in PreD3, thus isolating the reactive conformers.^14^ Regrettably, a lack of quantitative computational evidence has prevented either of these hypotheses from being supported, and the catalytic mechanism remains an open question.

With the intention of illuminating the catalytic mechanism, we conducted a comprehensive analysis of the dynamics of the isomerization reaction of PreD3 when encapsulated within the β-CD dimer. To achieve this, we utilized the statistical multistructural variational transition state theory (MS-VTST)^20^ in combination with MD simulations.

## Calculation Method

It has been demonstrated that the lateral chain (**R** in Figure 1) is not directly involved in the thermal isomerization and that, therefore, it can be safely replaced by a methyl group when studying this reaction in both gas-phase and *n*-hexane environments.^14^ We assume that this approximation also holds true within the β-CD dimer, and for this reason, we have also substituted this fragment by a methyl group. Hereinafter, we will replace the PreD3 name by PreD to refer to the modified reactant.

### Molecular Dynamics Simulations

MD simulations were conducted for both the free [f] and encapsulated [e] versions of PreD, in order to compare the accessible torsional space of the triene moiety in each case. The most stable HL conformer of PreD exhibiting a −c−c triene arrangement was selected as the initial configuration for the simulation. For the encapsulated version, this conformer was manually inserted inside the β-CD dimer cavity and optimized at the HF/6-31G level of theory before the MD simulation.

The xTB software (version 6.4.1), developed by Grimme et al.,^21^ was used, in combination with the semiempirical GFN2-xTB methodology,^22^ to calculate the forces throughout the simulation. Each system was propagated for 1 μs in five independent simulations with a time step of 2 ps at 310.15 K in an *NVT* ensemble (total simulation time per system of 5 μs). A Berendsen thermostat was used for keeping constant the temperature of the system, and the SHAKE algorithm was applied to all bonds.

### Conformational Search

All stationary-point geometries (equilibrium configurations and transition state structures) for the free system were located with the TorsiFlex software^23,24^ at the MPWB1K density functional method^25^ with the 6-31+G­(d,p) basis set,^26^ a combination which has previously demonstrated reliability for this system.^14^ Regarding the encapsulated system, each located conformer of interest for the free system was placed inside the β-CD dimer (see Figure 4) and optimized. The electronic structure calculations for the encapsulated system were carried out using the ONIOM hybrid method,^27^ where the high layer, consisting of PreD atoms, was computed at the MPWB1K/6-31+G­(d,p) level, and the low layer, reserved for the β-CD dimer atoms, was studied using the density-functional-based tight-binding semiempirical level with analytic expressions for the matrix elements (DFTBA).^28^ Finally, the absolute energy of each conformer, considering both the free and the encapsulated scenarios, was refined by means of single-point calculations with the MPWB1K/6-31+G­(d,p) level utilizing Truhlar and co-workers’ SMD variation^29^ of the integral equation formalism variant of the polarizable continuum model (IEFPCM).^30^ All electronic structure calculations were automatically managed by TorsiFlex and carried out with the *Gaussian*09 package.^31^

**Figure 4:**
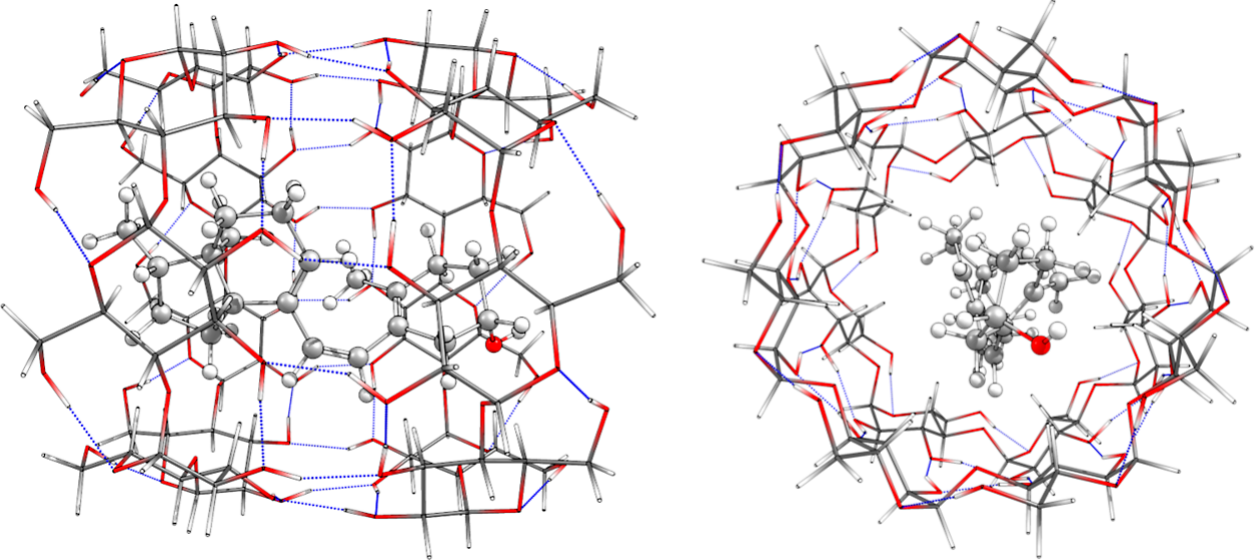
Molecule of PreD encapsulated inside the β-CD dimer.

### MS-VTST Rate Constants

We have calculated the multistructural (MS) partition functions^20,32,33^ for PreD by considering their thermically accessible conformers (based on the MD results). We notice that this partition function effectively weighs the contribution of each conformer of reactants based on its stability. Thus, even if the lowest-energy reactant conformer is not directly involved in the reaction, its influence on the overall reaction kinetics is fully considered in the calculations. The same applies to the two types of transition states, TS­(+) and TS(−).

The calculation of the MS partitions functions was performed with the aid of Pilgrim.^34^ With this information, Pilgrim is also able to estimate the unimolecular reaction rate constant through MS-TST as

1 $$k^{MS-TST}=\frac{k_{B}T}{h}\frac{Q^{MS,‡}}{Q_{R}^{MS}}e^{-V_{0}/\left(k_{B}T\right)}$$

where *Q*_R_^MS^ and *Q* ^MS,‡^ are, respectively, the MS ro-vibrational partition functions for the reactant (PreD) and the transition state, either TS­(+) or TS(−). The energy difference between the most stable conformer of the reactant and the transition state is denoted by *V*_0_. Vibrational frequencies, required for the calculation of the ro-vibrational partition functions, were determined applying the recommended parametrized scaling factor of 0.951 for the MPWB1K/6-31+G­(d,p) level.^35^ *k*_B_ and *h* are Boltzmann and Planck constants. *T* is the temperature, which was set to 37 °C (human body temperature).

MS-TST rate constants for both the (+) and (−) reactions were corrected, by considering variational effects and quantum tunneling, as follows

2 $$k^{MS-VTST}=\Gamma_{0}^{CVT}\cdot \kappa_{0}^{SCT}\cdot k^{MS-TST}=\gamma_{0}^{CVT/SCT}\cdot k^{MS-TST}$$

with Γ_0_^CVT^ being the variational coefficient that accounts for recrossing effects (calculated within the canonical variational transition state theory, CVT),^36,37^ and κ_0_^SCT^ being the tunneling transmission coefficient calculated by the small curvature tunneling (SCT) approximation.^14,38–40^ The 0 subscript indicates that these effects are calculated by employing the transition state conformation with the lowest energy for each reaction channel. The product of these two coefficients, γ_0_^CVT/SCT^, is the total transmission coefficient. In order to obtain γ_0_^CVT/SCT^, minimum energy path (MEP) calculations were performed for the most stable conformers of TS­(+) and TS(−) of the free previtamin and for the most stable conformer of TS(−) in the encapsulated scenario. Each MEP was followed using the Page-McIver^41^ algorithm with steps of 0.010 bohr with Hessian calculations every nine steps and automatically extended until κ_0_^SCT^ converged within an error smaller than 0.5%.

The magnitude of the catalytic effect can be estimated by the ratio between the MS-VTST rate constants for the encapsulated and free systems

3 $$\varphi =k_{\left[e\right]}^{MS-VTST}/k_{\left[f\right]}^{MS-VTST}$$

Notice that both eq 3 and Holick’s experimental findings assess the isomerization rate constant of the system under encapsulated conditions compared to its free state, neglecting the influence of the encapsulation and the decapsulation processes. In this context, the experimental and computational catalytic ratios might not accurately capture the magnitude of the catalytic effect if the free form of PreD were considered to be the common starting point.

### Factorization of the Catalytic Ratio

For the encapsulated systems, the rovibrational partition function includes contributions from both PreD and the β-CD dimer. The Hessian matrix for the encapsulated system, denoted as *F*_[e]_, comprises four distinct blocks
4

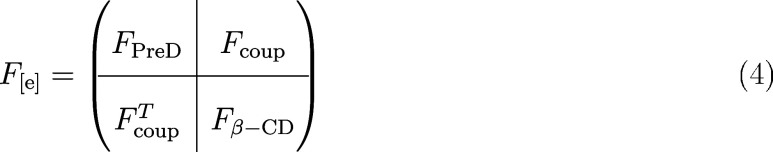




Here, *F*_PreD_ represents part of the matrix for PreD, while *F*_β‑CD_ is for the β-CD dimer. The *F*_coup_ parts show how these two components interact with each other.

The *F*_PreD_ submatrix in conjunction with the corresponding geometry of the PreD molecule allows estimating the contribution of PreD to the ro-vibrational partition function of the encapsulated system, *Q*_PreD_^[e]^. Similarly, *F*_β‑CD_ together with the geometric parameters of the β-CD dimer facilitates the estimation of the contribution associated with the β-CD dimer, *Q*_β‑CD_^[e]^. This allows factorizing the partition function into three distinct terms

5 $$Q^{\left[e\right]}=Q_{PreD}^{\left[e\right]}\cdot Q_{\beta -CD}^{\left[e\right]}\cdot f_{coup}$$

were the last term accounts for the coupling between PreD and the β-CD dimer and is calculated from *Q* ^[e]^ and two previous contributions.

Considering the previous decomposition, the catalytic ratio (φ) can be easily factored as a product of five terms

6 $$\varphi =\varphi_{\gamma_{0}}\cdot \varphi_{V_{0}}\cdot \varphi_{PreD}\cdot \varphi_{\beta -CD}\cdot \varphi_{coup}$$

The first term, φ_γ_0__, accounts for the change in the total CVT/SCT transmission coefficient. φ_*V*_0__ measures the modification of the energy barrier upon encapsulation. The third term examines the impact of encapsulation, specifically on the PreD atoms, in both the reactant and the transition state

$$\varphi_{PreD}={\left(\frac{Q_{PreD}^{\left[e\right]}}{Q^{\left[f\right]}}\right)}^{‡}\cdot {\left(\frac{Q_{PreD}^{\left[e\right]}}{Q^{\left[f\right]}}\right)}_{R}^{-1}$$ 7

where *Q* ^[f]^ represents the rovibrational partition function of the free previtamin D. The fourth term assesses the change in the β-CD dimer when transitioning from the reactant to the transition state.

8 $$\varphi_{\beta -CD}=\frac{{\left(Q_{\beta -CD}^{\left[e\right]}\right)}^{‡}}{{\left(Q_{\beta -CD}^{\left[e\right]}\right)}_{R}}$$

Meanwhile, φ_coup_ delves into the coupling between the PreD atoms and the dimer during the same process.

## Results and Discussion

In this section, we study and compare the dynamics of the thermal isomerization of PreD in its free [f] and its encapsulated version [e]. First, we inspect the outcome of the MD in order to understand how the β-CD dimer restricts the torsional space of PreD (subsection Accessible Conformers). Second, we explore the results of the conformational analysis (subsection Conformational Analysis). Finally, we estimate the reaction rate constants for thermal isomerization under both the free and the encapsulated conditions to determine the catalytic effect and comprehend its underlying causes (Thermal Rate Constants and Catalytic Effect).

### Accessible Conformers

Based on the findings from Furche and co-workers’ nonadiabatic MD simulations, it is clear that the UV-induced ring–opening reaction in ProD3 directly produces PreD3 in the −c−c reactive arrangement. For this reason, the −c−c triene configuration was used as the starting point for the MD simulations. These were carried out for both free and encapsulated PreD in order to determine the accessible domain within the triene subspace.

As anticipated, PreD is capable of attaining all stable triene arrangements in the free scenario, as evidenced in Figure 5 (top left panel). This clearly suggests an “unrestricted” molecular flexibility when PreD is not encapsulated. In other words, all PreD conformers are accessible at the reaction temperature and, thus, must be taken into account when estimating its partition function and, consequently, when estimating the rate constant of the thermal sigmatropic reaction. In contrast, MD simulations for the encapsulated system reveal a restricted torsional movement of the triene moiety (Figure 5, top right panel). Simulations initiated from the reactive −c−c configuration become trapped in the torsional space associated with the starting conformer, leaving the torsional space associated with the +c+c reactive arrangement, and to the nonreactive (NR) ones, unexplored.

**Figure 5:**
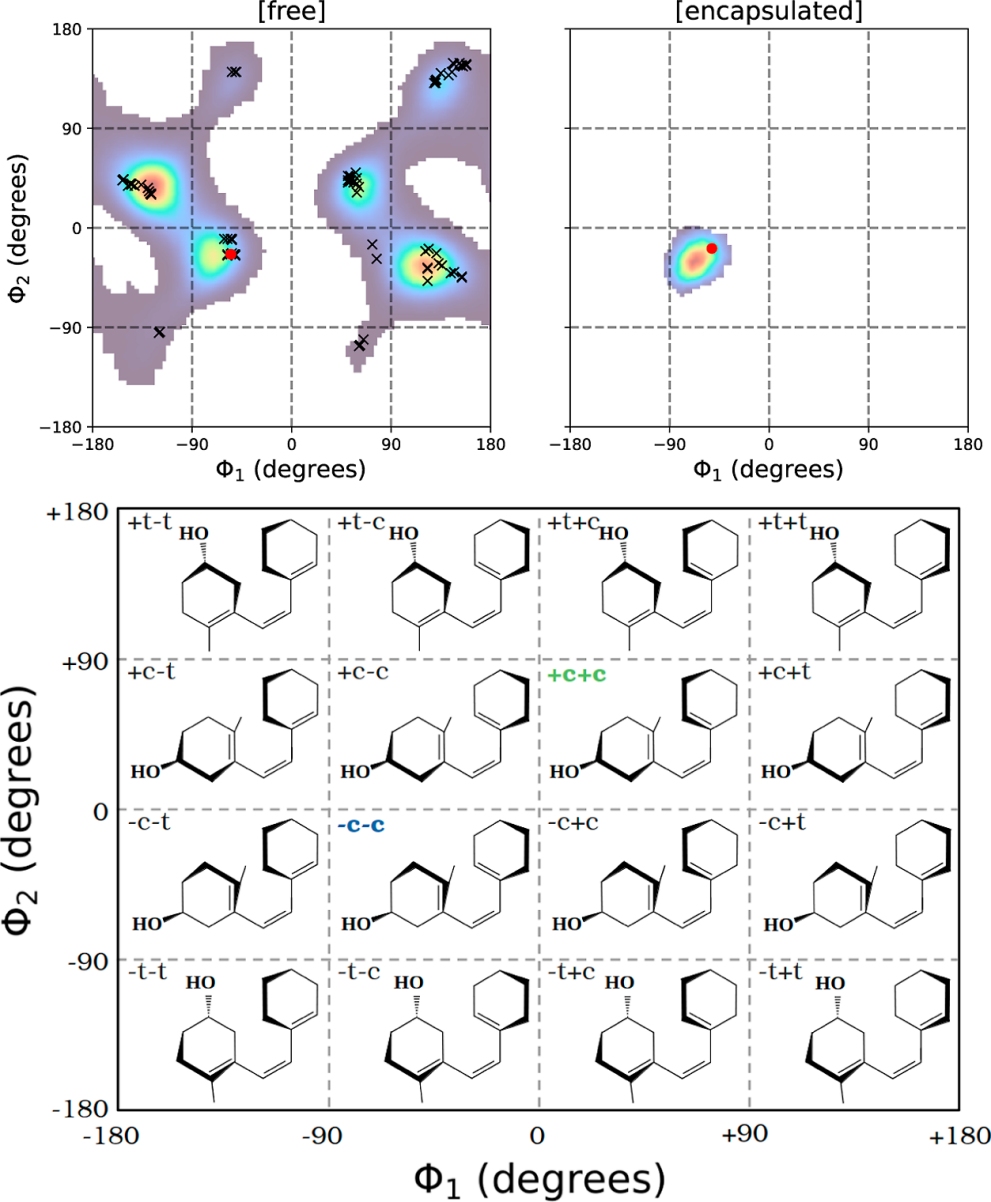
Upper plots are bidimensional histograms representing the triene torsional space explored during the MD simulation for PreD in both its free [f] and encapsulated [e] forms. Dihedral angles ϕ_1_ and ϕ_2_ are defined in Figure 1. The colors indicate the density of data points, with darker and warmer colors representing lower and higher densities, respectively. The initial −c−c geometry of the MD simulations is highlighted with a red circle. Conformers identified by TorsiFlex for the free system are marked with ‘x’. The lower plot illustrates the triene configuration relative to its dihedral angle values. The reactive −c−c and +c+c configuration are highlighted in blue and green, respectively. The **D** ring has been omitted (see Figure 1).

Hence, in accordance with Meana-Pañeda and Fernández-Ramos’ predictions, the β-CD dimer cavity impedes rotations about the single bonds in the triene moiety, rendering a fraction of the system’s conformers inaccessible at the temperature at which the reaction takes place. Consequently, only the −c−c pathway will lead to VitD when the system is encapsulated inside the β-CD dimer.

### Conformational Analysis

The conformational exploration using TorsiFlex identified a total of 103 conformers for PreD and 24 conformers for each transition state. This is nearly 3 times the count from an earlier study,^14^ primarily because it omitted conformers derived from OH group rotation. The distribution of these conformers by energy is shown in Figure 6a, and their Cartesian coordinates are provided in the Supporting Information.

**Figure 6:**
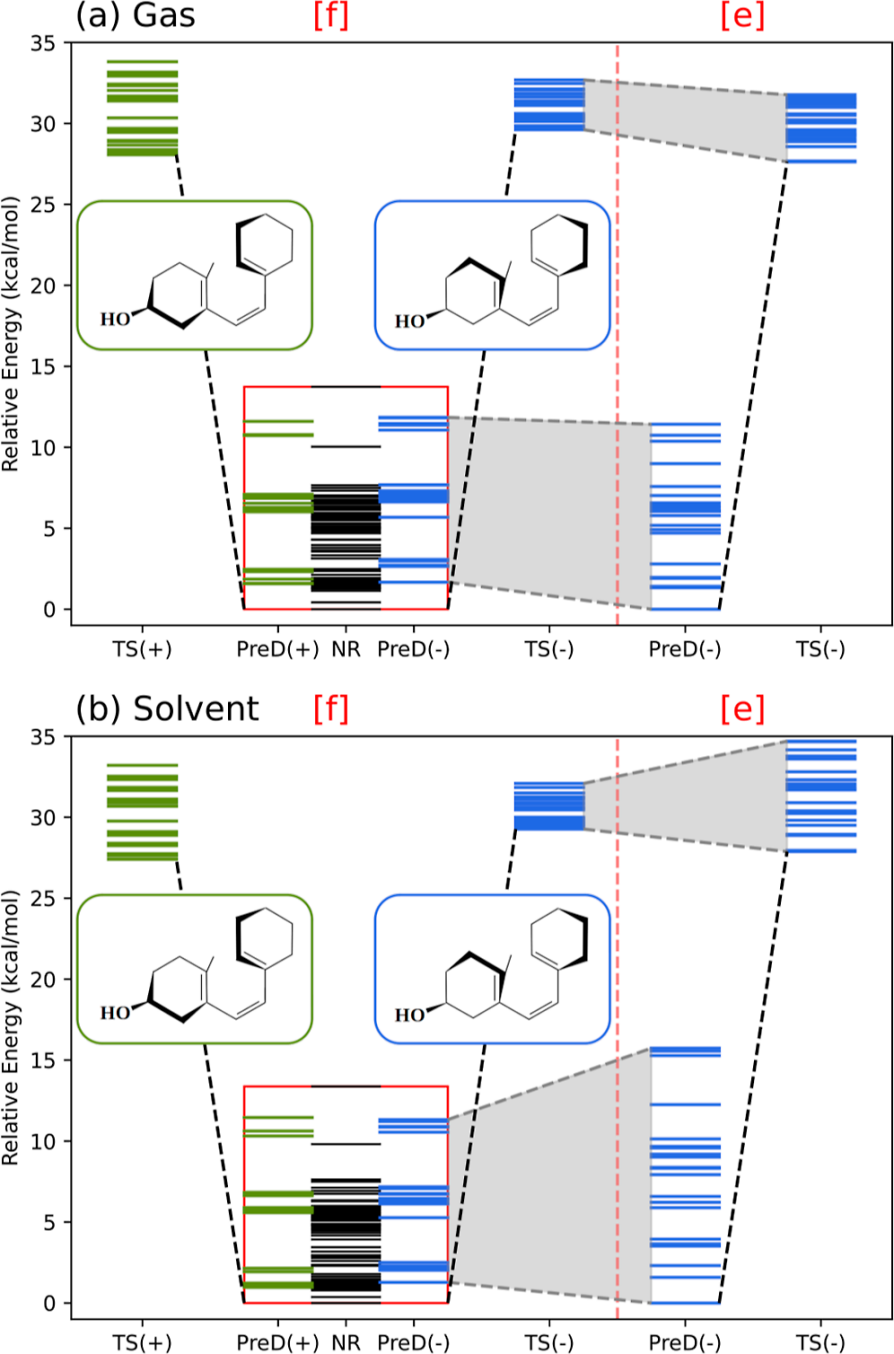
Energy distribution of PreD and TS conformers for both the free [f] and encapsulated [e] situations. Plot (a) corresponds to gas phase calculations, whereas plot (b) corresponds to SMD calculations. Black lines indicate NR configurations of reactants. Green and blue lines represent the energy of +c+c and −c−c reactants [PreD­(+), PreD(−)] and transition states [TS­(+) and TS(−)], respectively.

Echoing prior findings,^14,15^ the prevalent configuration of free PreD in the gas phase is the NR +t−c (Figure 3, top) with angles (ϕ_1_, ϕ_2_) = (123, −48°). The first reactive conformation stands at an energy 1.58 kcal/mol higher, corresponding to the +c+c triene with angles (ϕ_1_, ϕ_2_) = (+54, +43°), which is depicted in the center of Figure 3. The energy of the optimal −c−c conformation is marginally higher at 1.67 kcal/mol (Figure 3, bottom). Concerning the interconversion between triene conformations, we conducted a 2D potential energy surface (2D-PES) calculation at the MPWB1K/6-31+G­(d,p) level, as illustrated in Figure 7. This 2D-PES was generated by maintaining the remaining proper torsional angles of PreD at the values found in the most stable conformer. We observe that a direct interconversion between reactive conformers, specifically from −c−c to +c+c, is hindered due to a high energy barrier. However, it is feasible to transition between these conformers by passing through other conformations with distinct triene configurations, leading to interconversion barriers that are less than 6 kcal/mol. These results are congruent with those obtained from the MD simulation, which deliberately avoided triene configurations where ϕ_1_ approaches zero, but effectively explored regions associated with all system conformers.

**Figure 7:**
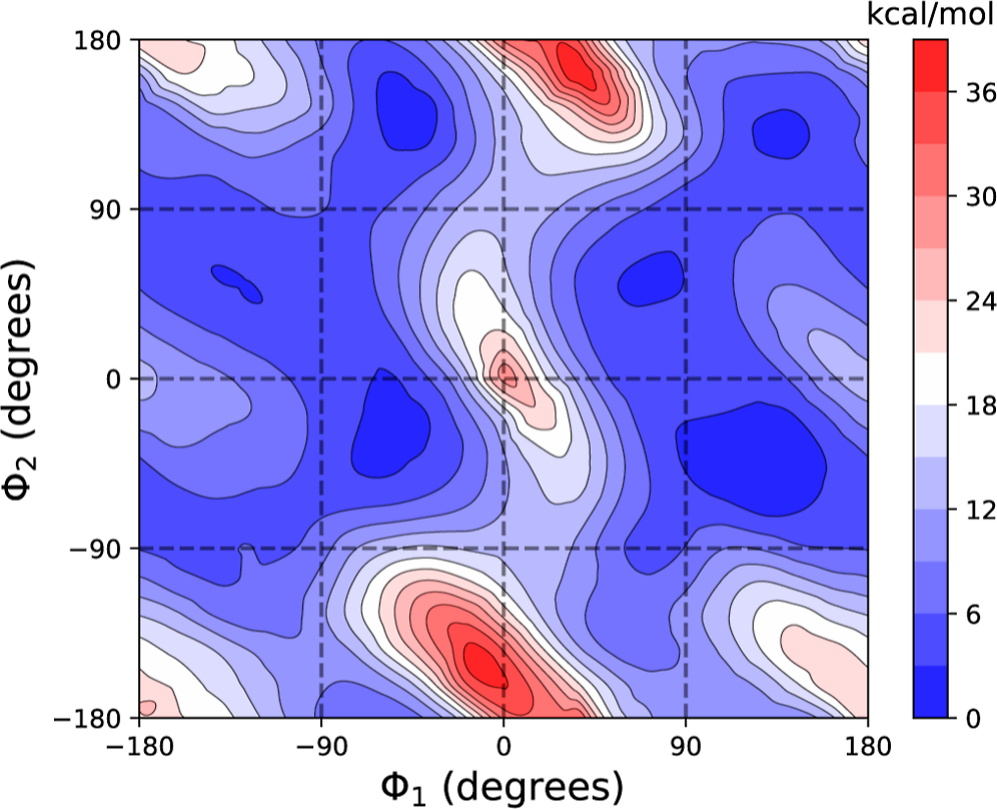
Contour plot illustrating the 2D-PES for the triene moiety of PreD, computed using the MPWB1K/6-31+G­(d,p) level of theory. The calculations were performed with the remaining proper torsions constrained to the values of the most stable conformer. Dihedral angles ϕ_1_ and ϕ_2_ are defined in Figure 1.

Regarding the thermal reaction, its barrier height (*V*_0_), which represents the energy difference between the most stable conformer of the reactant and the transition state, is 28.07 kcal/mol for the (+) pathway. Comparatively, the first transition state conformer for the (−) pathway is at 29.61 kcal/mol. This energy difference of 1.54 kcal/mol between the barriers of the two pathways aligns with the observation that the (+) channel exhibits a reaction rate approximately ten times faster than the (−) one.^14^

Encapsulation within the β-CD dimer dramatically alters this conformational energy distribution (Figure 6a). MD simulations reveal that the NR and +c+c triene configurations become inaccessible and, hence, the reactant and transition state are now restricted to the −c−c triene setup, bringing the reaction energy barrier down to *V*_0_ = 27.63 kcal/mol. This decrease of Δ*V*_0_ = −0.44 kcal/mol in the reaction barrier suggests a 2-fold augmentation of the rate constant upon encapsulation, estimated using φ ∼ exp­(−Δ*V*_0_/(*k*_B_ *T*)). Yet, this would fall short of experimental observations.

When the solvent environment is considered, Figure 6b, the results seem to contrast with experiments. Specifically, the energy barrier rises from *V*_0_ = 27.40 to *V*_0_ = 27.88 kcal/mol upon encapsulation, implying that the rate constant would actually halve at 37 °C. This indicates that the catalytic effect does not arise from alterations in the energy barrier.

In conclusion, the encapsulation and solvent environment significantly influence the conformational energy distribution and reaction barriers. However, the primary catalytic effect cannot be ascribed to these energy barrier alterations, pointing to other factors at play.

### Thermal Rate Constants and Catalytic Effect

Our MD simulations show that all conformers must be considered for the free scenario when computing partition functions and, hence, when calculating the rate constants. However, in the encapsulated scenario, only those with a −c−c triene arrangement should be incorporated. Resulting rate constants are 1.01×10^–5^ s^–1^ for the free system in *n*-hexane and 6.07×10^–4^ s^–1^ for the encapsulated system in water. While both values align closely with the experimental outcome reported by Holick and co-workers (0.68×10^–5^ and 3.07×10^–4^ s^–1^),^18^ they are slightly overestimated. It is worth noting that our rate constant for the free system closely agrees with the estimate from a previous computational study,^14^ which reported a value of 0.90×10^–5^ s^–1^. Notably, and as previously indicated, this prior study did not consider conformations resulting from OH group rotations, potentially explaining minor observed differences.

With the previous rate constants, the computed catalytic effect approximates 60, which closely aligns with the experimentally observed value of 45. When we dissect this ratio as per eq 6, it is evident that every component, except the reaction barrier, plays a role in influencing the catalytic effect (refer to Table 1). Notably, φ_PreD_ stands out as the contribution that is the most significant contributor. As expected, this underscores that the true essence of the catalytic effect is intricately tied to how the encapsulation modifies the PreD moiety.

**Table 1:** Ratio Between the Rate Constant for the Encapsulated System, [e], and the Free System, [f]^a^

	φ	φ_γ_0__	φ_*V*_0__	φ_PreD_	φ_β‑CD_	φ_coup_
experimental	45.10					
theoretical	59.79	1.19	0.46	19.58	3.38	1.64

a This ratio is split according to eq 6.

Several factors may influence φ_PreD_, including the reduction of conformational space; the impact of the β-CD dimer on encapsulated PreD conformers; and the solvent environment. To quantify these factors, we considered several hypothetical intermediate reactions, as depicted in Figure 8. For naming conventions, we employ **f** and **e** to denote reactions in the free and encapsulated systems, respectively. The subscripts **g**, **nh**, and **w** are utilized to estimate conditions in the gas phase, *n*-hexane, and water. Additionally, superscript **(−)** signifies that our focus is solely on the −c−c conformations for both reactants and transition state conformers.

(i) *Reduction of the conformational space*. The singular effect of conformational restriction imposed by the β-CD dimer can be appraised by analyzing two versions of the reaction within the free scenario in *n*-hexane: the real reaction incorporating all conformations, denoted as **f**_**nh**_, and a hypothetical one involving only the −c−c conformations, denoted as **f**_nh_^(−)^. Under the consideration of these two reactions, φ_PreD_ ∼ 5. This value accounts for the reduction in the number of conformers to be considered, which notably affects the reactant, since reactive +c+c and NR arrangements are no longer accessible upon encapsulation (the number of conformers is reduced from 103 to 22). Conversely, the impact on the transition state is less pronounced, as only the reactive +c+c configurations are rendered unavailable.
(ii) *Impact of the* β*-CD dimer on encapsulated PreD conformers*. Upon consideration of the gas-phase reaction, solely encompassing reactive −c−c conformers, for both the free system, denoted as **f**_**g**_^(−)^, and the encapsulated counterpart, **e**_**g**_^(−)^, it is possible to isolate the effect of the encapsulation from the conformational restriction previously measured. This effect also contributes to the catalytic effect, but only with a factor of φ_PreD_ ∼ 2. The origin of this acceleration can be found on the low-frequency vibrations of PreD. These low-frequency movements are more restricted upon encapsulation, leading to an increase of their vibrational frequencies and, consequently, to a decrease in the rovibrational partition function. For instance, the first three vibrational frequencies for the −c−c conformers of the reactant in gas phase are 24 ± 5, 31 ± 5, and 46 ± 5 cm^–1^, whereas these frequencies are, for the encapsulated system,[Fn fn1] 42 ± 5, 54 ± 4, and 64 ± 3 cm^–1^. This significant variation (more than 10%) takes place in the first six vibrational frequencies, where the value always increases upon encapsulation. This effect is less significant in the transition state structures, basically because the transferred H links ring **A** to ring **C**, already restricting these movements. As the increase of these low-frequencies is more significant in the reactant, the whole effect contributes to φ_PreD_ with a factor greater than 1.
(iii) *Effect of the solvent environment*. Ultimately, the impact of solvent alteration can be gauged through the analysis of two sets of reactions: **f**_nh_^(−)^ to **f**_**g**_^(−)^ and **e**_**g**_^(−)^ to **e**_**w**_^(−)^. Although the change in solvent significantly contributes to the catalytic mechanism, it represents the least influential effect in φ_PreD_, as it only imparts a factor of approximately 1.7.

**Figure 8:**
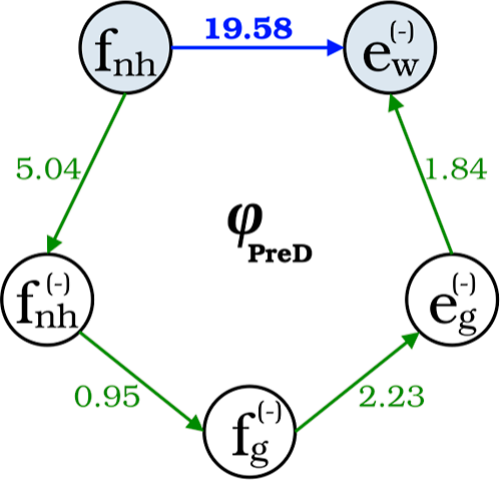
Contribution to the catalytic effect associated with the partition function of the PreD atoms, in bold blue, and its factorization by considering different intermediate reactions.

Drawing from our computational chemistry findings, it is apparent that the dominant factor driving the catalytic effect is the constrained rotation of single bonds in the triene moiety, resulting from the small cavity of the β-CD dimer. This conformational restriction leads to the seclusion of reactive −c−c conformers, manifested as a pronounced reduction in the number of conformers to be considered. Notably, this primarily affects the reactant, thereby elevating the rate constant. However, it should be acknowledged that this is not the sole effect contributing to the observed acceleration of Vitamin D’s thermal isomerization.

## Conclusions

Our computational results revealed a richer conformational landscape than previously documented in the PreD isomerization, emphasizing the importance of thorough conformational studies for molecular understanding.

Notably, the relevance of specific conformers varied dramatically between free and encapsulated conditions. In the encapsulated scenario, only the −c−c triene conformers are accessible. Our computed rate constants for both scenarios aligned closely with experimental observations, reinforcing the accuracy and reliability of our approach.

The crux of our findings is that the catalytic enhancement is primarily an entropic phenomenon rather than an enthalpic one. While enthalpic factors, such as the reaction barrier, would predict a slowdown in the reaction, it is the entropic factors, particularly the reduction in conformer variety and an upswing in low vibrational frequencies, that exert dominance.

More specifically, the restricted rotation of the triene moiety within the β-CD dimer emerged as the primary factor enhancing the catalytic effect. This pivotal observation resonates with the hypothesis of Meana-Pañeda and Fernández-Ramos, highlighting the relationship between conformational restraint and catalysis in Vitamin d isomerization.

Overall, our findings refine our understanding of Vitamin d isomerization catalysis and advocate for incorporating MD and environmental factors in biochemical modeling.

## Supplementary Material



## Data Availability

Chemical kinetics calculations were conducted using TorsiFlex^23,24^ and Pilgrim,^34^ which are components of the Cathedral Package^42^ of programs, freely accessible on *GitHub*:^43^ https://github.com/cathedralpkg. Initial geometries required for the conformational search with TorsiFlex can be found in the Supporting Information, together with the geometries and relative energies of the located conformers, which were used in the calculations with Pilgrim. The MD trajectories generated using the xTB software in this study are not publicly available due to their substantial size, exceeding 10 GB. Nonetheless, the initial geometries are provided in the Supporting Information, and the parameters utilized for these MD simulations are thoroughly detailed within the main manuscript.

## Footnotes

1 These frequencies are calculated by diagonalizing a reduced Hessian matrix that contains the terms exclusively associated with the PreD atoms (see subsection 2.4, Factorization of the Catalytic Ratio).

## Notes

Elaboration on computational aspects: expanded insights into the conformational search and the partition function calculation, relative energies of identified conformers, and Cartesian coordinates of the located conformers and of the starting geometries used in the MD simulations (PDF)
