# Supplementary material for: Unraveling the Catalytic Mechanism of β‑Cyclodextrin in the Vitamin D Formation
Source: J Chem Inf Model. 2024 Apr 10;64(9):3865–73. doi: 10.1021/acs.jcim.3c02049 (PMC12529775; doi:10.1021/acs.jcim.3c02049)
Supplement: Supplementary file 1 [file ci3c02049_si_001.pdf]

Supporting Information

Unraveling the Catalytic Mechanism of β-Cyclodextrin in the Vitamin D Formation

David Ferro-Costas,[*,†,¶] Pedro A. Sánchez-Murcia,[‡,¶] and Antonio Fernández-Ramos[†,§]

†*Departamento de Química Física, Universidade de Santiago de Compostela, 15782 Santiago de Compostela, Spain*

‡*Laboratory of Computer-Aided Molecular Design, Division of Medicinal Chemistry, Medical University of Graz, Neue Stiftingtalstraße 6/III, A-8010 Graz, Austria*

¶*Institute of Theoretical Chemistry, University of Vienna, Währinger Straße 17, 1090 Vienna, Austria*

§*Centro Singular de Investigación en Química Biolóxica e Materiais Moleculares (CIQUS), Universidade de Santiago de Compostela, 15782 Santiago de Compostela, Spain*

E-mail: david.ferro@usc.es

Contents

S1 Elaboration on Computational Aspects **3**

S1.1 Conformational Search with `TorsiFlex` 3

S1.1.1 Reference Z-matrix and target torsions for PreD 4

S1.1.2 Reference Z-matrix and target torsions for TS(+) 8

S1.1.3 Reference Z-matrix and target torsions for TS(−) 12

S1.2 Finding Conformers within the Encapsulated System 16

S1.3 Calculation of Multi-Structural Partition Functions 17

S2 Relative Energy of the Located Conformers **18**

S2.1 Located conformers for free PreD 18

S2.2 Located conformers for free TS(+) 22

S2.3 Located conformers for free TS(−) 24

S2.4 Located conformers for encapsulated PreD 26

S2.5 Located conformers for encapsulated TS(−) 28

S3 Cartesian Coordinates **30**

S3.1 Cartesian Coordinates of PreD Conformers 30

S3.2 Cartesian Coordinates of TS(+) Conformers 168

S3.3 Cartesian Coordinates of TS(−) Conformers 200

S3.4 Cartesian Coordinates for the β-cyclodextrin dimer 232

S3.5 Cartesian Coordinates of encapsulated PreD Conformers 240

S3.6 Cartesian Coordinates of encapsulated TS Conformers 431

S3.7 Initial MD Geometry for the Free System 640

S3.8 Initial MD Geometry for the Encapsulated System 642

References **651**

S1. Elaboration on Computational Aspects

S1.1. Conformational Search with `TorsiFlex`

The `TorsiFlex` software[1–3] was employed to generate *all* the conformers of PreD and the transition state (TS) in their free form. The program locates conformers using a dual-level protocol. Initially, an inexpensive level of theory (HF/3-21G) referred to as low-level (LL) was utilized. This electronic structure method is known for yielding more precise transition states than Density Functional Theory (DFT) methods and is particularly suitable for hydrogen transfer reactions.[4]

Subsequently, high-level (HL) reoptimizations of LL conformers were conducted using the MPWB1K/6-31+G(d,p) level of theory,[5,6] which has exhibited reliability in prior studies related to this system.[7] The electronic structure calculations were automatically managed by `TorsiFlex`, employing the Gaussian 09 package.[8]

In the case of PreD, a comprehensive exploration of nine torsions was undertaken (see S1.1.1). Among these, two torsions accounted for the conformations of the triene moiety, one for the hydroxyl group, and six to evaluate potential ring configurations (specifically three for ring **A** and three for ring **C**; see Fig. S1). Notably, for the two transition states (see S1.1.2 and S1.1.3), the torsions linked to the triene moiety were excluded from the conformational search. It should be noted that while `TorsiFlex` is not specifically tailored to handle torsions in cyclic moieties, it may still be applied with careful calibration of the preconditioned angles and their corresponding dihedral domains.

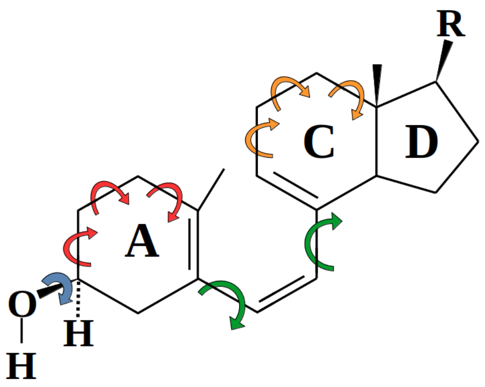

Figure S1: Selected torsions for the conformational search in previtamin D.

S1.1.1. Reference Z-matrix and target torsions for PreD

This section provides the Z-matrix used in the conformational search with `TorsiFlex` for PreD.

```
c
c    1  cc2
c    2  cc3     1  ccc3
c    3  cc4     2  ccc4     1  dih4
c    4  cc5     3  ccc5     2  dih5
c    5  cc6     4  ccc6     3  dih6
c    6  cc7     5  ccc7     4  dih7
h    7  hc8     6  hcc8     5  dih8
c    6  cc9     5  ccc9     7  dih9
c    9  cc10    6  ccc10    5  dih10
c   10  cc11    9  ccc11    6  dih11
c   11  cc12   10  ccc12    9  dih12
c    1  cc13    2  ccc13    8  dih13
c   13  cc14    1  ccc14    2  dih14
c   14  cc15   13  ccc15    1  dih15
c   15  cc16   14  ccc16   13  dih16
c   15  cc17   14  ccc17   16  dih17
c   17  cc18   15  ccc18   16  dih18
c   18  cc19   17  ccc19   15  dih19
o   11  oc20   12  occ20   10  dih20
h   20  ho21   11  hoc21   10  dih21
c   15  cc22   17  ccc22   14  dih22
```

c	17	cc23	15	ccc23	18	dih23
h	11	hc24	10	hcc24	12	dih24
h	12	hc25	11	hcc25	5	dih25
h	12	hc26	11	hcc26	5	dih26
h	10	hc27	9	hcc27	11	dih27
h	10	hc28	9	hcc28	11	dih28
h	9	hc29	10	hcc29	6	dih29
h	9	hc30	10	hcc30	6	dih30
h	7	hc31	6	hcc31	8	dih31
h	7	hc32	6	hcc32	8	dih32
h	4	hc33	5	hcc33	3	dih33
h	3	hc34	4	hcc34	2	dih34
h	1	hc35	2	hcc35	8	dih35
h	13	hc36	1	hcc36	14	dih36
h	13	hc37	1	hcc37	14	dih37
h	14	hc38	13	hcc38	15	dih38
h	14	hc39	15	hcc39	13	dih39
h	17	hc40	18	hcc40	15	dih40
h	18	hc41	17	hcc41	19	dih41
h	18	hc42	17	hcc42	19	dih42
h	19	hc43	18	hcc43	16	dih43
h	19	hc44	18	hcc44	16	dih44
h	22	hc45	15	hcc45	14	dih45
h	22	hc46	45	hch46	15	dih46
h	22	hc47	45	hch47	15	dih47
h	23	hc48	17	hcc48	15	dih48
h	23	hc49	48	hch49	17	dih49
h	23	hc50	48	hch50	17	dih50
h	16	hc51	15	hcc51	2	dih51

The value associated to each internal coordinate is listed below. Bond distances are given in Å; bond and dihedral angles, in degrees.

cc2	1.4043	cc15	1.5385	hc27	1.1062
cc3	1.4065	ccc15	110.8194	hcc27	110.9426
ccc3	123.261	dih15	−35.0779	dih27	238.6915
cc4	1.4033	cc16	1.5658	hc28	1.1058
ccc4	126.541	ccc16	106.8036	hcc28	110.6709
dih4	7.53	dih16	59.1182	dih28	121.4403
cc5	1.3955	cc17	1.5625	hc29	1.1106
ccc5	133.495	ccc17	114.6914	hcc29	109.2817
dih5	23.903	dih17	110.7069	dih29	236.5242
cc6	1.3964	cc18	1.5549	hc30	1.113
ccc6	130.581	ccc18	104.1193	hcc30	109.4333
dih6	22.146	dih18	−40.0545	dih30	121.7931
cc7	1.4187	cc19	1.5471	hc31	1.0793
ccc7	123.692	ccc19	107.3137	hcc31	120.2107
dih7	4.726	dih19	18.258	dih31	244.5619
hc8	1.2011	oc20	1.4352	hc32	1.0878
hcc8	99.881	occ20	110.0049	hcc32	118.3276
dih8	−70.185	dih20	−122.0778	dih32	101.1239
cc9	1.5142	ho21	0.9743	hc33	1.1075
ccc9	122.7197	hoc21	111.6475	hcc33	114.1996
dih9	−179.0000	dih21	299.0057	dih33	182.7093
cc10	1.5264	cc22	1.5342	hc34	1.0917
ccc10	115.3032	ccc22	111.4167	hcc34	116.0347
dih10	9.4789	dih22	−126.4291	dih34	174.0624
cc11	1.5316	cc23	1.5241	hc35	1.1008
ccc11	108.3499	ccc23	115.1865	hcc35	117.1663
dih11	−43.8393	dih23	−124.1997	dih35	94.6898
cc12	1.5321	hc24	1.1137	hc36	1.1119
ccc12	110.1531	hcc24	111.9991	hcc36	107.6681
dih12	62.9355	dih24	234.46	dih36	238.1848
cc13	1.5174	hc25	1.1101	hc37	1.1079
ccc13	122.7439	hcc25	108.3141	hcc37	109.6712
dih13	−118.5948	dih25	236.4529	dih37	123.9534
cc14	1.5405	hc26	1.1095	hc38	1.1056
ccc14	116.1624	hcc26	109.4493	hcc38	109.8864
dih14	13.0016	dih26	121.957	dih38	121.946

hc39	1.1047	hcc43	110.8008	dih47	237.4823
hcc39	110.4291	dih43	239.968	hc48	1.0958
dih39	121.8312	hc44	1.1007	hcc48	111.0384
hc40	1.1167	hcc44	111.7942	dih48	184.6061
hcc40	108.37	dih44	121.1382	hc49	1.0959
dih40	245.0015	hc45	1.095	hch49	107.1815
hc41	1.1019	hcc45	111.5065	dih49	122.9687
hcc41	111.0911	dih45	184.4403	hc50	1.0957
dih41	239.1288	hc46	1.0963	hch50	107.6529
hc42	1.1008	hch46	106.996	dih50	238.1248
hcc42	110.4982	dih46	122.1396	hc51	1.1266
dih42	121.4641	hc47	1.0939	hcc51	107.9845
hc43	1.1033	hch47	107.8187	dih51	114.5547

The torsions selected for the conformational search are:

- `torsion1`: `dih10`
- `torsion2`: `dih11`
- `torsion3`: `dih12`
- `torsion4`: `dih14`
- `torsion5`: `dih15`
- `torsion6`: `dih16`
- `torsion7`: `dih21`
- `torsion8`: `dih6`
- `torsion9`: `dih4`

S1.1.2. Reference Z-matrix and target torsions for TS(+)

This section provides the Z-matrix used in the conformational search with `TorsiFlex` for TS(+).

```
c
c    1  cc2
c    2  cc3       1  ccc3
c    3  cc4       2  ccc4      1  dih4
c    4  cc5       3  ccc5      2  dih5
c    5  cc6       4  ccc6      3  dih6
c    6  cc7       5  ccc7      4  dih7
h    7  hc8       6  hcc8      5  dih8
c    6  cc9       5  ccc9      7  dih9
c    9  cc10      6  ccc10     5  dih10
c   10  cc11      9  ccc11     6  dih11
c   11  cc12     10  ccc12     9  dih12
c    1  cc13      2  ccc13     8  dih13
c   13  cc14      1  ccc14     2  dih14
c   14  cc15     13  ccc15     1  dih15
c   15  cc16     14  ccc16    13  dih16
c   15  cc17     14  ccc17    16  dih17
c   17  cc18     15  ccc18    16  dih18
c   18  cc19     17  ccc19    15  dih19
o   11  oc20     12  occ20    10  dih20
h   20  ho21     11  hoc21    10  dih21
c   15  cc22     17  ccc22    14  dih22
c   17  cc23     15  ccc23    18  dih23
h   11  hc24     10  hcc24    12  dih24
h   12  hc25     11  hcc25     5  dih25
h   12  hc26     11  hcc26     5  dih26
h   10  hc27      9  hcc27    11  dih27
h   10  hc28      9  hcc28    11  dih28
h    9  hc29     10  hcc29     6  dih29
h    9  hc30     10  hcc30     6  dih30
h    7  hc31      6  hcc31     8  dih31
h    7  hc32      6  hcc32     8  dih32
h    4  hc33      5  hcc33     3  dih33
h    3  hc34      4  hcc34     2  dih34
h    1  hc35      2  hcc35     8  dih35
```

h	13	hc36	1	hcc36	14	dih36
h	13	hc37	1	hcc37	14	dih37
h	14	hc38	13	hcc38	15	dih38
h	14	hc39	15	hcc39	13	dih39
h	17	hc40	18	hcc40	15	dih40
h	18	hc41	17	hcc41	19	dih41
h	18	hc42	17	hcc42	19	dih42
h	19	hc43	18	hcc43	16	dih43
h	19	hc44	18	hcc44	16	dih44
h	22	hc45	15	hcc45	14	dih45
h	22	hc46	45	hch46	15	dih46
h	22	hc47	45	hch47	15	dih47
h	23	hc48	17	hcc48	15	dih48
h	23	hc49	48	hch49	17	dih49
h	23	hc50	48	hch50	17	dih50
h	16	hc51	15	hcc51	2	dih51

The value associated to each internal coordinate is listed below. Bond distances are given in Å; bond and dihedral angles, in degrees.

cc2	1.4043	cc15	1.5385	hc27	1.1062
cc3	1.4065	ccc15	110.8194	hcc27	110.9426
ccc3	123.261	dih15	−35.0779	dih27	238.6915
cc4	1.4033	cc16	1.5658	hc28	1.1058
ccc4	126.541	ccc16	106.8036	hcc28	110.6709
dih4	7.53	dih16	59.1182	dih28	121.4403
cc5	1.3955	cc17	1.5625	hc29	1.1106
ccc5	133.495	ccc17	114.6914	hcc29	109.2817
dih5	23.903	dih17	110.7069	dih29	236.5242
cc6	1.3964	cc18	1.5549	hc30	1.113
ccc6	130.581	ccc18	104.1193	hcc30	109.4333
dih6	22.146	dih18	−40.0545	dih30	121.7931
cc7	1.4187	cc19	1.5471	hc31	1.0793
ccc7	123.692	ccc19	107.3137	hcc31	120.2107
dih7	4.726	dih19	18.258	dih31	244.5619
hc8	1.3948	oc20	1.4352	hc32	1.0878
hcc8	99.881	occ20	110.0049	hcc32	118.3276
dih8	−70.185	dih20	−122.0778	dih32	101.1239
cc9	1.5142	ho21	0.9743	hc33	1.1075
ccc9	122.7197	hoc21	111.6475	hcc33	114.1996
dih9	−180.0000	dih21	299.0057	dih33	182.7093
cc10	1.5264	cc22	1.5342	hc34	1.0917
ccc10	115.3032	ccc22	111.4167	hcc34	116.0347
dih10	9.4789	dih22	−126.4291	dih34	174.0624
cc11	1.5316	cc23	1.5241	hc35	1.1008
ccc11	108.3499	ccc23	115.1865	hcc35	117.1663
dih11	−43.8393	dih23	−124.1997	dih35	94.6898
cc12	1.5321	hc24	1.1137	hc36	1.1119
ccc12	110.1531	hcc24	111.9991	hcc36	107.6681
dih12	62.9355	dih24	234.46	dih36	238.1848
cc13	1.5174	hc25	1.1101	hc37	1.1079
ccc13	122.7439	hcc25	108.3141	hcc37	109.6712
dih13	−118.5948	dih25	236.4529	dih37	123.9534
cc14	1.5405	hc26	1.1095	hc38	1.1056
ccc14	116.1624	hcc26	109.4493	hcc38	109.8864
dih14	13.0016	dih26	121.957	dih38	121.946

hc39	1.1047	hcc43	110.8008	dih47	237.4823
hcc39	110.4291	dih43	239.968	hc48	1.0958
dih39	121.8312	hc44	1.1007	hcc48	111.0384
hc40	1.1167	hcc44	111.7942	dih48	184.6061
hcc40	108.37	dih44	121.1382	hc49	1.0959
dih40	245.0015	hc45	1.095	hch49	107.1815
hc41	1.1019	hcc45	111.5065	dih49	122.9687
hcc41	111.0911	dih45	184.4403	hc50	1.0957
dih41	239.1288	hc46	1.0963	hch50	107.6529
hc42	1.1008	hch46	106.996	dih50	238.1248
hcc42	110.4982	dih46	122.1396	hc51	1.1266
dih42	121.4641	hc47	1.0939	hcc51	107.9845
hc43	1.1033	hch47	107.8187	dih51	114.5547

The torsions selected for the conformational search are:

- `torsion1`: `dih10`
- `torsion2`: `dih11`
- `torsion3`: `dih12`
- `torsion4`: `dih14`
- `torsion5`: `dih15`
- `torsion6`: `dih16`
- `torsion7`: `dih21`

S1.1.3. Reference Z-matrix and target torsions for TS(−)

This section provides the Z-matrix used in the conformational search with `TorsiFlex` for TS(−).

```
c
c    1  cc2
c    2  cc3     1  ccc3
c    3  cc4     2  ccc4     1  dih4
c    4  cc5     3  ccc5     2  dih5
c    5  cc6     4  ccc6     3  dih6
c    6  cc7     5  ccc7     4  dih7
h    7  hc8     6  hcc8     5  dih8
c    6  cc9     5  ccc9     7  dih9
c    9  cc10    6  ccc10    5  dih10
c   10  cc11    9  ccc11    6  dih11
c   11  cc12   10  ccc12    9  dih12
c    1  cc13    2  ccc13    8  dih13
c   13  cc14    1  ccc14    2  dih14
c   14  cc15   13  ccc15    1  dih15
c   15  cc16   14  ccc16   13  dih16
c   15  cc17   14  ccc17   16  dih17
c   17  cc18   15  ccc18   16  dih18
c   18  cc19   17  ccc19   15  dih19
o   11  oc20   12  occ20   10  dih20
h   20  ho21   11  hoc21   10  dih21
c   15  cc22   17  ccc22   14  dih22
c   17  cc23   15  ccc23   18  dih23
h   11  hc24   10  hcc24   12  dih24
h   12  hc25   11  hcc25    5  dih25
h   12  hc26   11  hcc26    5  dih26
h   10  hc27    9  hcc27   11  dih27
h   10  hc28    9  hcc28   11  dih28
h    9  hc29   10  hcc29    6  dih29
h    9  hc30   10  hcc30    6  dih30
h    7  hc31    6  hcc31    8  dih31
h    7  hc32    6  hcc32    8  dih32
h    4  hc33    5  hcc33    3  dih33
h    3  hc34    4  hcc34    2  dih34
h    1  hc35    2  hcc35    8  dih35
```

h	13	hc36	1	hcc36	14	dih36
h	13	hc37	1	hcc37	14	dih37
h	14	hc38	13	hcc38	15	dih38
h	14	hc39	15	hcc39	13	dih39
h	17	hc40	18	hcc40	15	dih40
h	18	hc41	17	hcc41	19	dih41
h	18	hc42	17	hcc42	19	dih42
h	19	hc43	18	hcc43	16	dih43
h	19	hc44	18	hcc44	16	dih44
h	22	hc45	15	hcc45	14	dih45
h	22	hc46	45	hch46	15	dih46
h	22	hc47	45	hch47	15	dih47
h	23	hc48	17	hcc48	15	dih48
h	23	hc49	48	hch49	17	dih49
h	23	hc50	48	hch50	17	dih50
h	16	hc51	15	hcc51	2	dih51

The value associated to each internal coordinate is listed below. Bond distances are given in Å; bond and dihedral angles, in degrees.

cc2	1.4043	cc15	1.5384	hc27	1.1063
cc3	1.4065	ccc15	112.9651	hcc27	110.8312
ccc3	123.261	dih15	−6.809	dih27	238.4043
cc4	1.4033	cc16	1.5661	hc28	1.1063
ccc4	126.541	ccc16	107.7546	hcc28	110.3998
dih4	−7.53	dih16	47.2923	dih28	121.5527
cc5	1.3955	cc17	1.5649	hc29	1.1105
ccc5	133.495	ccc17	114.4856	hcc29	108.819
dih5	−23.903	dih17	110.674	dih29	236.0912
cc6	1.3964	cc18	1.554	hc30	1.1129
ccc6	130.581	ccc18	104.0849	hcc30	109.5843
dih6	−22.146	dih18	−40.2129	dih30	121.4545
cc7	1.4187	cc19	1.5464	hc31	1.0887
ccc7	123.692	ccc19	107.1721	hcc31	118.0933
dih7	−4.726	dih19	20.1834	dih31	258.607
hc8	1.3948	oc20	1.4351	hc32	1.0792
hcc8	99.881	occ20	110.1499	hcc32	120.1716
dih8	70.185	dih20	−121.9732	dih32	115.2253
cc9	1.5153	ho21	0.9743	hc33	1.1073
ccc9	122.3378	hoc21	111.6441	hcc33	114.2678
dih9	−179.0000	dih21	298.6715	dih33	177.6985
cc10	1.5269	cc22	1.5335	hc34	1.0917
ccc10	115.4896	ccc22	111.1205	hcc34	116.1067
dih10	21.6924	dih22	−125.2957	dih34	186.4153
cc11	1.5316	cc23	1.5239	hc35	1.0994
ccc11	109.0148	ccc23	115.4581	hcc35	117.8834
dih11	−43.8599	dih23	−124.2909	dih35	−94.4334
cc12	1.5321	hc24	1.1135	hc36	1.1085
ccc12	110.0278	hcc24	112.1301	hcc36	108.8441
dih12	60.7436	dih24	234.5785	dih36	236.0314
cc13	1.5163	hc25	1.1093	hc37	1.1113
ccc13	121.5388	hcc25	108.1987	hcc37	107.7292
dih13	118.1725	dih25	236.6696	dih37	122.3042
cc14	1.545	hc26	1.1088	hc38	1.1063
ccc14	117.3018	hcc26	110.0451	hcc38	109.0636
dih14	−23.1601	dih26	121.7195	dih38	122.3382

hc39	1.1052	hcc43	111.0221	dih47	237.4728
hcc39	109.8149	dih43	239.9081	hc48	1.096
dih39	122.7563	hc44	1.1007	hcc48	111.0007
hc40	1.1171	hcc44	111.6787	dih48	184.7598
hcc40	108.3303	dih44	121.1349	hc49	1.096
dih40	245.1691	hc45	1.0947	hch49	107.1404
hc41	1.1021	hcc45	111.5751	dih49	122.8937
hcc41	111.0287	dih45	175.1133	hc50	1.0958
dih41	239.2407	hc46	1.0953	hch50	107.6167
hc42	1.1007	hch46	107.5202	dih50	238.1438
hcc42	110.5757	dih46	122.5144	hc51	1.1264
dih42	121.5348	hc47	1.0944	hcc51	107.6799
hc43	1.1034	hch47	107.7005	dih51	114.7759

The torsions selected for the conformational search are:

- `torsion1`: `dih10`
- `torsion2`: `dih11`
- `torsion3`: `dih12`
- `torsion4`: `dih14`
- `torsion5`: `dih15`
- `torsion6`: `dih16`
- `torsion7`: `dih21`

S1.2. Finding Conformers within the Encapsulated System

The conformers of PreD and TS featuring the -c-c triene arrangement were enclosed within the β-CD dimer and underwent a sequential optimization protocol. First, the system was partially optimized while freezing the PreD (or TS) structure. The resulting structure was partially optimized while freezing the β-CD dimer structure. Finally, the entire system was subjected to geometry optimization, followed by frequency calculations (see Fig. S2). This three-step protocol allowed the β-CD dimer to adapt initially to the encapsulated conformer, facilitating a closer alignment between the final conformer and its initial geometry.

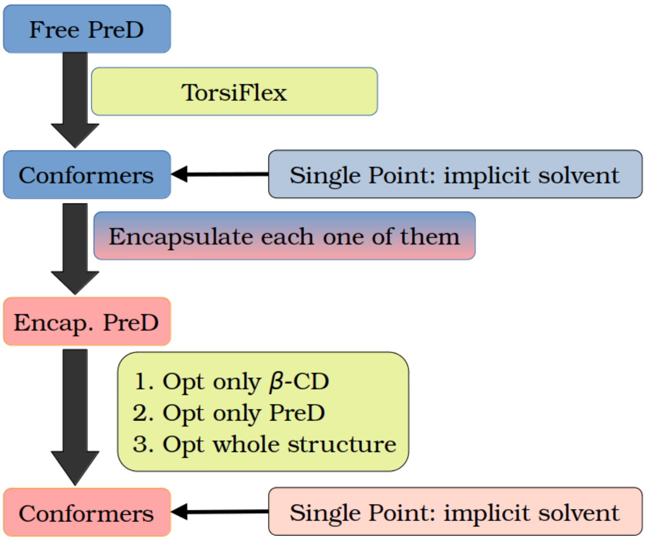

Figure S2: Process of obtaining conformers for the encapsulated system.

The electronic structure calculations for the encapsulated system were carried out using the ONIOM hybrid method,[9] which involved two layers: the high layer, consisting of PreD atoms, was computed at the MPWB1K/6-31+G(d,p) level, while the low layer, reserved for the β-CD dimer atoms, was studied using the density-functional-based tight-binding semi-empirical level with analytic expressions for the matrix elements (DFTBA).[10]

S1.3. Calculation of Multi-Structural Partition Functions

The computation of multi-structural (MS) rovibrational partition functions is conducted using the Pilgrim software,[1,11] employing the following expression:

$$Q_{\rm rv}^{\rm MS} = \sum_i Q_{{\rm rv},i}^{\rm 1W} \cdot e^{-U_i \beta} \tag{S1}$$

In this equation, the summation accounts for thermally accessible conformers within the system. Here, β represents $(k_B T)^{-1}$, where T denotes the temperature and k_B denotes the Boltzmann constant. Additionally, U_i refers to the relative energy, while $Q_{{\rm rv},i}^{\rm 1W}$ stands for the rovibrational one-well (1W), also referred to as single-structure, partition function of the i-th conformer.

The 1W rovibrational partition function is commonly approached using the rigid-rotor harmonic-oscillator (RR-HO) approximation. This approximation method involves the factorization of the partition function into separate contributions from rotational and vibrational degrees of freedom,:

$$Q_{\rm rv}^{RR-HO} = Q_{\rm r}^{\rm RR} \cdot Q_{\rm v}^{\rm HO} \tag{S2}$$

While computing the rovibrational partition function, the zero-point energies (ZPEs) were calculated using a scaling factor of 0.951, as recommended for our selected high-level of theory.[12] When incorporating such a scaling factor, the resulting vibrational partition function is often termed quasi-harmonic (QH). Consequently, $Q_{\rm rv}^{\text{RR-HO}}$ transforms into $Q_{\rm rv}^{\text{RR-QH}}$ in this context.

S2. Relative Energy of the Located Conformers

In this section, we display the relative energy of each located conformer for each studied system. U represents the total energy, whereas $U + \varepsilon$ refers to the total energy plus the vibrational zero point energy (ε). Conformers are sorted according to their ΔU in gas phase.

S2.1. Located conformers for free PreD

The following reference energies were considered:

- For gas phase calculations:

  Ref. U $\rightarrow$ -854.99312 Hartree

  Ref. $U + \varepsilon \rightarrow$ -854.54477 Hartree

- For solvent phase calculations:

  Ref. U $\rightarrow$ -855.00933 Hartree

  Ref. $U + \varepsilon \rightarrow$ -854.56097 Hartree

Table S1: Relative energies (in kcal mol^{-1}) for the conformers of free PreD.

		**gas**		**SMD**	
Conformer	Triene	ΔU	$\Delta(U + \epsilon)$	ΔU	$\Delta(U + \epsilon)$
1	+t-c	0.00	0.00	0.00	0.00
2	-c+t	0.42	0.71	0.37	0.66
3	+t-c	1.14	0.96	0.90	0.73
4	-c+t	1.27	1.32	1.16	1.21
5	-c+t	1.29	1.34	1.06	1.11
6	+t+t	1.31	1.50	1.22	1.41
7	-c+t	1.42	1.45	1.17	1.20
8	-t+c	1.51	1.10	0.78	0.37
9	-t+c	1.52	1.49	0.87	0.84
10	+t-c	1.57	1.57	0.83	0.82
11	+c+c	1.58	1.56	0.97	0.96

12	+c+c	1.58	1.53	1.23	1.18
13	+c+c	1.60	1.57	0.99	0.95
14	-c+t	1.67	1.68	1.48	1.49
15	-c-c	1.67	1.74	1.28	1.34
16	-c+t	1.69	1.73	1.37	1.41
17	-t+c	1.72	1.36	0.97	0.61
18	+t-c	1.73	1.73	1.79	1.79
19	-t+c	1.84	1.46	1.04	0.66
20	+c+c	1.86	1.82	1.17	1.13
21	+t-c	1.93	1.84	1.17	1.08
22	+t-c	2.13	1.97	1.29	1.14
23	+c+c	2.32	2.04	1.93	1.66
24	-t+c	2.36	2.12	1.63	1.39
25	+t+t	2.38	2.27	2.30	2.19
26	+c+c	2.46	2.22	2.15	1.90
27	+t+t	2.48	2.42	2.36	2.30
28	-c-c	2.64	2.46	2.03	1.85
29	-c-c	2.64	2.48	2.14	1.97
30	-c-c	2.72	2.48	2.10	1.86
31	-c-c	2.97	2.73	2.28	2.04
32	-c-c	3.07	2.85	2.50	2.28
33	-t+c	3.13	2.88	2.35	2.09
34	+t+t	3.14	2.97	2.60	2.42
35	+t+t	3.33	3.07	2.78	2.52
36	+t+t	3.56	3.28	2.96	2.67
37	+t+t	3.63	3.56	2.88	2.81
38	+c-t	3.79	4.08	3.93	4.22
39	+t+t	3.96	3.87	3.18	3.09
40	+t+t	4.29	4.22	3.45	3.38
41	-c+t	4.30	4.44	4.34	4.48
42	+c-t	4.69	4.89	5.13	5.33

43	+c-t	4.75	5.00	5.11	5.35
44	+t-c	4.88	4.74	4.17	4.03
45	-t-t	4.98	4.46	4.57	4.05
46	-t+c	5.01	4.89	4.48	4.36
47	-t-t	5.08	4.58	4.64	4.14
48	-c+t	5.12	5.29	5.23	5.41
49	-t-t	5.25	4.75	4.76	4.25
50	-t+c	5.31	5.17	4.80	4.66
51	+t-c	5.49	5.11	5.27	4.89
52	-t+c	5.55	5.30	4.84	4.59
53	+t-c	5.61	5.27	4.89	4.56
54	-c-c	5.68	5.82	5.27	5.42
55	+t-c	5.68	5.58	5.72	5.62
56	+t-c	5.69	5.60	5.60	5.51
57	-t+c	5.72	5.51	5.09	4.87
58	-c+t	5.79	5.87	5.75	5.83
59	+t-c	5.92	5.70	5.39	5.18
60	+t+t	5.92	5.85	5.39	5.32
61	-t+c	5.96	5.53	5.29	4.87
62	+c+c	6.01	6.02	5.88	5.89
63	+c+c	6.09	5.73	5.58	5.22
64	+c+c	6.13	5.93	5.87	5.67
65	-c+t	6.14	6.19	5.93	5.99
66	-t+c	6.22	5.64	5.50	4.92
67	+t-c	6.22	5.87	5.93	5.58
68	+c+c	6.28	5.87	5.70	5.30
69	+c+c	6.29	5.83	5.70	5.25
70	-t+c	6.35	5.74	5.58	4.96
71	-c+t	6.49	6.46	6.34	6.31
72	+c+c	6.54	6.07	5.88	5.41
73	+t+t	6.57	6.49	5.99	5.92

74	-c-c	6.63	6.54	6.12	6.03
75	-t+c	6.68	6.27	5.82	5.42
76	-c-c	6.76	6.57	6.13	5.95
77	+t+t	6.77	6.39	6.75	6.36
78	+c+c	6.85	6.74	6.69	6.58
79	-c-c	6.87	6.63	6.22	5.98
80	-c-c	6.91	6.70	6.42	6.21
81	+t+t	6.93	6.93	6.29	6.28
82	+c+c	6.99	6.65	6.63	6.29
83	+t+t	7.03	6.73	6.93	6.63
84	-c-c	7.06	6.98	6.46	6.37
85	+c+c	7.10	6.65	6.83	6.38
86	-c-c	7.13	6.89	6.41	6.17
87	-c-c	7.23	6.82	6.72	6.31
88	-c-c	7.32	7.10	6.76	6.55
89	+t+t	7.32	7.34	7.12	7.14
90	+t+t	7.49	7.46	7.50	7.46
91	+t+t	7.65	7.79	7.63	7.77
92	-c-c	7.67	7.36	7.18	6.87
93	-c-c	7.68	7.36	7.07	6.74
94	+c-c	10.03	9.99	9.81	9.76
95	+c+c	10.72	10.37	10.62	10.27
96	+c+c	10.78	10.17	10.32	9.71
97	-c-c	11.06	10.91	10.55	10.40
98	-c-c	11.37	11.15	10.86	10.64
99	-c-c	11.46	11.32	10.91	10.76
100	+c+c	11.60	10.96	11.46	10.81
101	-c-c	11.81	11.62	11.32	11.12
102	-c-c	11.85	11.61	11.21	10.98
103	+c-c	13.74	13.47	13.37	13.10

S2.2. Located conformers for free TS(+)

The following reference energies were considered:

- For gas phase calculations:

  Ref. U $\rightarrow$ -854.94840 Hartree

  Ref. $U + \varepsilon \rightarrow$ -854.50402 Hartree

- For solvent phase calculations:

  Ref. U $\rightarrow$ -854.96566 Hartree

  Ref. $U + \varepsilon \rightarrow$ -854.52130 Hartree

Table S2: Relative energies (in kcal mol^{-1}) for the conformers of free TS(+).

	gas		SMD	
Conformer	ΔU	$\Delta(U+\epsilon)$	ΔU	$\Delta(U+\epsilon)$
1	0.00	0.32	0.25	0.58
2	0.01	0.00	0.00	0.00
3	0.05	0.02	0.03	0.00
4	0.14	0.40	0.35	0.62
5	0.32	0.26	0.24	0.20
6	0.59	0.64	0.85	0.90
7	0.82	1.03	0.92	1.14
8	0.88	1.14	0.99	1.26
9	1.38	1.56	1.48	1.67
10	1.52	1.56	1.71	1.76
11	1.63	1.75	1.62	1.76
12	2.27	2.29	2.36	2.39
13	3.32	3.20	3.28	3.18
14	3.37	3.23	3.31	3.18
15	3.41	3.46	3.58	3.64
16	3.58	3.59	3.72	3.74
17	3.62	3.47	3.51	3.37
18	3.98	4.00	4.25	4.28
19	4.26	4.23	4.30	4.28

20	4.36	4.48	4.44	4.57
21	4.86	4.90	4.93	4.98
22	5.00	4.88	5.13	5.02
23	5.10	5.15	5.07	5.13
24	5.74	5.61	5.80	5.68

S2.3. Located conformers for free TS$(-)$

The following reference energies were considered:

- For gas phase calculations:

  Ref. U $\rightarrow$ -854.94594 Hartree

  Ref. $U + \varepsilon \rightarrow$ -854.50139 Hartree

- For solvent phase calculations:

  Ref. U $\rightarrow$ -854.96269 Hartree

  Ref. $U + \varepsilon \rightarrow$ -854.51807 Hartree

Table S3: Relative energies (in kcal mol^{-1}) for the conformers of free TS$(-)$.

	**gas**		**SMD**	
Conformer	ΔU	$\Delta(U+\epsilon)$	ΔU	$\Delta(U+\epsilon)$
1	0.00	0.00	0.04	0.00
2	0.07	0.15	0.00	0.04
3	0.08	0.12	0.04	0.04
4	0.25	0.35	0.29	0.36
5	0.51	0.43	0.18	0.06
6	0.62	0.52	0.27	0.13
7	0.71	0.62	0.39	0.26
8	0.79	0.69	0.38	0.25
9	0.82	0.71	0.49	0.34
10	0.85	0.69	0.67	0.46
11	0.89	0.74	0.74	0.54
12	1.00	0.88	0.60	0.44
13	1.50	1.50	1.35	1.31
14	1.51	1.33	1.19	0.97
15	1.57	1.36	1.28	1.03
16	1.73	1.67	1.55	1.45
17	1.79	1.65	1.64	1.46
18	2.02	1.81	1.84	1.59
19	2.21	1.93	1.99	1.67

20	2.23	2.15	1.95	1.83
21	2.44	2.32	2.23	2.07
22	2.52	2.30	2.23	1.97
23	2.85	2.58	2.57	2.26
24	3.07	2.95	2.82	2.66

S2.4. Located conformers for encapsulated PreD

The following reference energies were considered:

- For gas phase calculations:

  Ref. U $\rightarrow$ -1284.22617 Hartree

  Ref. $U + \varepsilon \rightarrow$ -1281.58291 Hartree

- For solvent phase calculations:

  Ref. U $\rightarrow$ -9402.49457 Hartree

  Ref. $U + \varepsilon \rightarrow$ -9399.85139 Hartree

Table S4: Relative energies (in kcal mol^{-1}) for the conformers of encapsulated PreD.

	**gas**		**SMD**	
Conformer	ΔU	$\Delta(U+\epsilon)$	ΔU	$\Delta(U+\epsilon)$
1	0.00	0.00	2.31	2.36
2	1.33	0.96	3.52	3.20
3	1.41	1.16	1.59	1.38
4	1.91	2.78	3.65	4.57
5	1.96	2.58	3.56	4.22
6	2.80	2.75	0.00	0.00
7	4.70	5.86	8.38	9.58
8	4.92	4.86	9.57	9.56
9	5.18	5.82	6.58	7.26
10	5.78	6.53	9.67	10.46
11	6.04	6.89	9.19	10.09
12	6.08	6.10	7.93	7.99
13	6.09	6.91	5.89	6.75
14	6.27	5.96	9.02	8.75
15	6.38	7.07	10.13	10.87
16	6.56	7.58	8.32	9.38
17	7.02	6.75	3.94	3.72
18	7.58	8.50	6.22	7.19
19	8.99	9.98	15.74	16.78

20	10.37	11.13	12.25	13.05
21	10.74	11.47	15.28	16.05
22	11.42	12.30	15.56	16.48

S2.5. Located conformers for encapsulated TS$(-)$

The following reference energies were considered:

- For gas phase calculations:

  Ref. U $\rightarrow$ -1284.18214 Hartree

  Ref. $U + \varepsilon$ $\rightarrow$ -1281.54287 Hartree

- For solvent phase calculations:

  Ref. U $\rightarrow$ -9402.45014 Hartree

  Ref. $U + \varepsilon$ $\rightarrow$ -9399.81119 Hartree

Table S5: Relative energies (in kcal mol^{-1}) for the conformers of encapsulated TS$(-)$.

	**gas**		**SMD**	
Conformer	ΔU	$\Delta(U+\epsilon)$	ΔU	$\Delta(U+\epsilon)$
1	0.00	0.00	4.44	4.63
2	0.05	0.05	3.02	3.22
3	0.94	0.88	1.00	1.13
4	1.25	1.28	4.42	4.64
5	1.33	1.23	2.36	2.45
6	1.46	1.49	6.28	6.50
7	1.64	1.53	3.81	3.89
8	1.64	1.51	2.52	2.58
9	1.72	1.63	5.90	6.00
10	1.85	1.69	4.03	4.07
11	1.99	2.13	5.72	6.06
12	2.40	2.36	6.78	6.94
13	2.46	2.36	0.00	0.09
14	2.55	2.38	3.88	3.90
15	2.59	2.47	2.41	2.48
16	2.87	2.75	1.06	1.14
17	2.96	2.98	2.41	2.63
18	3.36	4.32	2.44	3.58
19	3.54	3.28	0.07	0.00

20	3.61	3.32	4.19	4.09
21	3.76	4.68	4.93	6.04
22	3.88	3.40	1.93	1.65
23	3.97	3.49	1.63	1.34
24	4.15	3.81	6.82	6.68

S3. Cartesian Coordinates

Included within this section are the Cartesian coordinates of the PreD and transition state conformers. The conformers for the free system were optimized using the MPWB1K/6-31+G(d,p) method, whereas the conformers for the encapsualted system were optimized using the ONIOM(MPWB1K/6-31+G(d,p):DFTBA). Lines beginning with an asterisk denote the relative energy of each conformer (excluding zero-point energy) and the dihedral angle for respective target torsions, delineated by underscores. Furthermore, the initial Cartesian coordinates (in Å) employed for the β-cyclodextrin dimer, housing conformers with the `-c-c` triene configuration (refer to section S1.2), are provided. The selected starting geometries for the MD simulations are also included.

S3.1. Cartesian Coordinates of PreD Conformers

```
51
 * E =  +0.000 kcal/mol ; (1) 349_041_298_010_320_062_281_123_312
 C   +0.00000000   +0.00000000   +0.00000000
 C   +0.00000000   +0.00000000   +1.33695909
 C   +1.23748215   +0.00000000   +2.12078425
 C   +2.29943403   −0.78889925   +1.94256394
 C   +2.37925924   −1.92975485   +1.00901379
 C   +3.30590870   −2.01346487   +0.04696038
 C   +4.29304803   −0.92571468   −0.22151192
 H   +4.27790693   −0.64672940   −1.27572546
 C   +3.40356331   −3.19387745   −0.87357643
 C   +2.55022454   −4.37710178   −0.45939689
 C   +1.19731198   −3.90190363   +0.03001641
 C   +1.38581995   −3.03644286   +1.25552399
 C   −1.22842427   +0.04857710   −0.85340219
 C   −2.50644042   +0.34977056   −0.07293541
 C   −2.49030192   −0.38456549   +1.25278198
 C   −1.30479082   +0.13796108   +2.06111640
```

C	−3.62284131	−0.06867407	+2.23373849
C	−3.03440892	−0.50096898	+3.59141091
C	−1.50144124	−0.49374383	+3.43080478
O	+0.51200149	−3.19090222	−0.97828033
H	+0.88321953	−2.30760408	−1.00828024
C	−2.41001405	−1.88728531	+1.00676696
C	−4.97113312	−0.68288298	+1.93049291
H	+0.56317269	−4.75301627	+0.26858461
H	+1.72363726	−3.64685401	+2.09562870
H	+0.43112073	−2.60550965	+1.55118902
H	+3.03623168	−4.92661557	+0.34724960
H	+2.42618141	−5.06304473	−1.29455250
H	+3.12073356	−2.86915975	−1.87913901
H	+4.44922857	−3.49466785	−0.95711430
H	+5.30778778	−1.26201355	−0.00505657
H	+4.09523093	−0.03382099	+0.36466761
H	+3.16237031	−0.61570925	+2.57446652
H	+1.27997913	+0.72552380	+2.92548851
H	+0.95467486	−0.02075850	−0.51251967
H	−1.32493269	−0.91099087	−1.37009227
H	−1.08924223	+0.78836692	−1.64218665
H	−2.57524851	+1.42218992	+0.12046250
H	−3.37712415	+0.07576471	−0.66961267
H	−3.73790640	+1.01895997	+2.23727129
H	−3.39232787	−1.49674361	+3.85165159
H	−3.36363105	+0.16100904	+4.38925269
H	−1.09574792	−1.50488273	+3.44717090
H	−0.99987369	+0.05830494	+4.22184430
H	−2.47142087	−2.45427917	+1.93410601
H	−1.49108748	−2.16978991	+0.50005799
H	−3.23971506	−2.20432154	+0.37673883
H	−5.71725147	−0.35344939	+2.65116924
H	−4.93002669	−1.76965980	+1.97681415
H	−5.32465307	−0.40163171	+0.93942216
H	−1.48208545	+1.21292916	+2.19079883

51

* E = +0.424 kcal/mol ; (2) 352_043_296_006_321_064_286_310_141

C	+0.00000000	+0.00000000	+0.00000000
C	+0.00000000	+0.00000000	+1.33887766
C	+1.28587844	+0.00000000	+2.04470096

C	+1.64191000	+0.65346865	+3.15693316
C	+0.87399667	+1.61721713	+3.94631657
C	+0.24584376	+2.68290460	+3.41873926
C	+0.33060657	+3.08104742	+1.98216948
H	+0.47027070	+4.15988764	+1.91193439
C	−0.56691412	+3.61972759	+4.26471319
C	−0.76714964	+3.15180766	+5.69125850
C	+0.53866435	+2.60857751	+6.23388499
C	+0.93135803	+1.38528058	+5.43558731
C	−1.21709114	+0.01915688	−0.86652915
C	−2.51455768	+0.19434309	−0.08571989
C	−2.44252581	−0.59615910	+1.20382337
C	−1.31766735	−0.00577870	+2.05824451
C	−3.61402847	−0.43220425	+2.17425351
C	−3.00830391	−0.87491349	+3.51992785
C	−1.47988603	−0.73456423	+3.38327498
O	+1.55203854	+3.58783026	+6.19449671
H	+1.82441863	+3.69479532	+5.28286143
C	−2.22236210	−2.07530050	+0.89896693
C	−4.89439446	−1.15164658	+1.81297402
H	+0.43486714	+2.34645369	+7.28394791
H	+0.29782765	+0.54153749	+5.71455864
H	+1.94805286	+1.09671865	+5.70690339
H	−1.51660551	+2.36014927	+5.72646883
H	−1.12300297	+3.97084156	+6.31215882
H	−0.08974020	+4.60385657	+4.25858319
H	−1.53165564	+3.77411153	+3.77753312
H	−0.58946112	+2.84986699	+1.44221692
H	+1.14506332	+2.58699456	+1.46465683
H	+2.63318261	+0.44131818	+3.54333645
H	+2.05292447	−0.60879744	+1.58124257
H	+0.95304085	−0.01561989	−0.51581701
H	−1.24810950	−0.91262223	−1.43819508
H	−1.11487708	+0.80727385	−1.61353545
H	−2.66012159	+1.24907818	+0.15762768
H	−3.36331686	−0.11287002	−0.69776215
H	−3.82880748	+0.63885251	+2.22579718
H	−3.28778628	−1.90625502	+3.73418686
H	−3.39994255	−0.27637232	+4.33972134
H	−0.99260858	−1.70760853	+3.34758594

H −1.03799771 −0.18827340 +4.20875704

H −2.32463960 −2.69092082 +1.79012714

H −1.23372291 −2.25772420 +0.48619434

H −2.95981770 −2.42342726 +0.17707841

H −5.67619366 −0.93807137 +2.53939829

H −4.75027030 −2.23061483 +1.79655371

H −5.26303881 −0.84663802 +0.83452242

H −1.60398968 +1.03396247 +2.24811756

51

* E = +1.136 kcal/mol ; (3) 345_044_299_009_321_062_199_123_323

C +0.00000000 +0.00000000 +0.00000000

C +0.00000000 +0.00000000 +1.33859740

C +1.22710733 +0.00000000 +2.13691275

C +2.37810026 −0.63238220 +1.88822770

C +2.62986234 −1.60923468 +0.81230216

C +3.59164725 −1.44633600 −0.10104278

C +4.44874020 −0.22579648 −0.16654494

H +4.39443332 +0.22215908 −1.15951877

C +3.84162923 −2.45174033 −1.18247859

C +3.18583462 −3.79518713 −0.93800317

C +1.76778132 −3.60540435 −0.45569247

C +1.76969024 −2.84250737 +0.84889226

C −1.23083333 +0.07403119 −0.84982444

C −2.51099269 +0.36026815 −0.06908626

C −2.49201916 −0.38088168 +1.25206976

C −1.30710481 +0.14112893 +2.06102654

C −3.62863767 −0.07478951 +2.23065550

C −3.04074528 −0.50161402 +3.59000516

C −1.50774889 −0.49039441 +3.43136889

O +1.07838946 −2.89914932 −1.46459378

H +0.29448110 −2.50579617 −1.08516730

C −2.40372116 −1.88353359 +1.00675375

C −4.97117360 −0.69906684 +1.92164782

H +1.29395662 −4.57890520 −0.30315241

H +2.11401115 −3.50115720 +1.64962785

H +0.74967040 −2.56556366 +1.11878321

H +3.73269185 −4.35240519 −0.17742621

H +3.19237005 −4.39156782 −1.84808369

H +3.47187756 −2.03461003 −2.12165862

H +4.91747392 −2.57987651 −1.31332285

H	+5.49633838	−0.47709872	+0.00354498
H	+4.15390833	+0.52874157	+0.55590109
H	+3.19719748	−0.44224862	+2.57173019
H	+1.18454103	+0.60820378	+3.03325311
H	+0.94691033	−0.05026455	−0.52160113
H	−1.33831740	−0.86385586	−1.40488150
H	−1.08531929	+0.83125602	−1.62062001
H	−2.58500390	+1.43029671	+0.13348119
H	−3.38084460	+0.08818806	−0.66800608
H	−3.75105651	+1.01179489	+2.23270502
H	−3.39658251	−1.49712673	+3.85450590
H	−3.37315588	+0.16233794	+4.38472360
H	−1.09973407	−1.50022478	+3.45007058
H	−1.01066354	+0.06319111	+4.22389224
H	−2.50459760	−2.45336457	+1.92815612
H	−1.45257241	−2.15824942	+0.55645748
H	−3.19744191	−2.20383583	+0.33387956
H	−5.72161443	−0.37960254	+2.64212692
H	−4.92150560	−1.78585481	+1.96363470
H	−5.32533836	−0.41633269	+0.93126041
H	−1.48528844	+1.21585587	+2.18947324

51

* E = +1.273 kcal/mol ; (4) 346_048_297_006_321_064_187_310_141

C	+0.00000000	+0.00000000	+0.00000000
C	+0.00000000	+0.00000000	+1.33896485
C	+1.28643920	+0.00000000	+2.04398676
C	+1.64103971	+0.64933458	+3.15918926
C	+0.87015242	+1.60505381	+3.95424791
C	+0.23609549	+2.66932897	+3.43859868
C	+0.32354936	+3.08681649	+2.00848668
H	+0.47882908	+4.16466576	+1.95859836
C	−0.56970206	+3.59191446	+4.30047269
C	−0.86326552	+3.03460677	+5.67612442
C	+0.41051972	+2.50112887	+6.28413052
C	+0.93012015	+1.35776292	+5.43785885
C	−1.21641434	+0.01527604	−0.86761542
C	−2.51504063	+0.18647590	−0.08799936
C	−2.44134968	−0.60211173	+1.20255033
C	−1.31878284	−0.00716863	+2.05706949
C	−3.61458944	−0.43939608	+2.17094859

C	−3.01007602	−0.87871232	+3.51798881
C	−1.48204632	−0.73157676	+3.38452528
O	+1.31421922	+3.58331115	+6.32894561
H	+2.17179685	+3.26879035	+6.60487845
C	−2.21690893	−2.08102869	+0.90001615
C	−4.89291732	−1.16217626	+1.80893388
H	+0.22099371	+2.14007767	+7.29825706
H	+0.38027265	+0.44900806	+5.69271457
H	+1.96767323	+1.14268327	+5.71189372
H	−1.58254913	+2.21753089	+5.60912890
H	−1.29145021	+3.80195008	+6.31736372
H	−0.03073475	+4.53624981	+4.39695731
H	−1.50224604	+3.82711800	+3.78398902
H	−0.60087327	+2.87849148	+1.46629139
H	+1.13141393	+2.59183768	+1.48173275
H	+2.63599792	+0.44030341	+3.53944207
H	+2.05568893	−0.60060522	+1.57340592
H	+0.95335456	−0.01302959	−0.51531569
H	−1.24437249	−0.91656910	−1.43947837
H	−1.11637499	+0.80388727	−1.61442238
H	−2.66432025	+1.24084608	+0.15428993
H	−3.36233511	−0.12454305	−0.70030992
H	−3.83153482	+0.63126462	+2.22062389
H	−3.28577847	−1.91123228	+3.73192191
H	−3.40614069	−0.28132285	+4.33661058
H	−0.98963922	−1.70230917	+3.35556681
H	−1.04654721	−0.17757709	+4.20836583
H	−2.31617001	−2.69549477	+1.79238564
H	−1.22788903	−2.26045419	+0.48681276
H	−2.95373054	−2.43298700	+0.17926838
H	−5.67653431	−0.94870008	+2.53349368
H	−4.74649090	−2.24091269	+1.79499206
H	−5.26037817	−0.86001212	+0.82913241
H	−1.60662252	+1.03255204	+2.24344895

51

* E = +1.291 kcal/mol ; (5) 016_315_062_006_321_064_182_306_141

C	+0.00000000	+0.00000000	+0.00000000
C	+0.00000000	+0.00000000	+1.33850885
C	+1.28332887	+0.00000000	+2.05030773
C	+1.62439467	+0.66167181	+3.16101268

C	+0.83625395	+1.61802038	+3.94282604
C	+0.24913390	+2.70824143	+3.42708648
C	+0.33692430	+3.10165900	+1.98932648
H	+0.56709070	+4.16383748	+1.91176120
C	−0.54467360	+3.65374327	+4.28224401
C	−0.29922448	+3.49895302	+5.76866243
C	−0.30629759	+2.04252927	+6.14227306
C	+0.81703499	+1.33223430	+5.42313792
C	−1.21791051	+0.02336771	−0.86560732
C	−2.51394901	+0.20572848	−0.08383137
C	−2.44535911	−0.58546563	+1.20544888
C	−1.31716744	−0.00057284	+2.05899066
C	−3.61522779	−0.41779278	+2.17709475
C	−3.00975223	−0.86528337	+3.52153552
C	−1.48084644	−0.73240851	+3.38256166
O	−0.17597625	+1.95136444	+7.54014451
H	−0.14415418	+1.03164518	+7.79481011
C	−2.23204577	−2.06543734	+0.89901525
C	−4.89894215	−1.13141220	+1.81610106
H	−1.26204960	+1.60618560	+5.82740653
H	+0.73991139	+0.25435627	+5.58122465
H	+1.76380876	+1.64062245	+5.87441238
H	−1.05177695	+4.03107960	+6.34638651
H	+0.67151261	+3.91544333	+6.03868591
H	−0.32736719	+4.67712602	+3.97609291
H	−1.60661556	+3.50682167	+4.06109179
H	−0.61348992	+2.94871727	+1.47336126
H	+1.09286508	+2.54085492	+1.45269782
H	+2.61335457	+0.46009469	+3.56033019
H	+2.05458039	−0.60959100	+1.59501601
H	+0.95283865	−0.01671643	−0.51614832
H	−1.25435385	−0.90847401	−1.43686320
H	−1.11294146	+0.81079567	−1.61295471
H	−2.65323346	+1.26114724	+0.15983752
H	−3.36478726	−0.09685935	−0.69537048
H	−3.82530167	+0.65401978	+2.23067297
H	−3.29427643	−1.89551056	+3.73484646
H	−3.39704644	−0.26539955	+4.34248601
H	−0.99882063	−1.70797342	+3.34181350
H	−1.03509080	−0.19240872	+4.21087813

H −2.33876672 −2.68207588 +1.78895789
H −1.24379982 −2.25196275 +0.48719077
H −2.97027039 −2.40869725 +0.17556196
H −5.67956494 −0.91485712 +2.54291689
H −4.75962700 −2.21102213 +1.79891566
H −5.26653246 −0.82419473 +0.83794737
H −1.59657888 +1.04092008 +2.25087567
51
* E = +1.306 kcal/mol ; (6) 349_042_297_007_321_063_289_135_140
C +0.00000000 +0.00000000 +0.00000000
C +0.00000000 +0.00000000 +1.33791065
C +1.27734941 +0.00000000 +2.06260735
C +1.61383513 +0.67131917 +3.16840241
C +0.85866882 +1.72400791 +3.87028294
C +0.72832051 +1.72160774 +5.20533660
C +1.20019792 +0.59324822 +6.06214441
H +0.41246029 +0.28643287 +6.75106258
C +0.06980055 +2.84055786 +5.95652582
C −0.18948082 +4.07705162 +5.11872480
C −0.70762224 +3.67433449 +3.75372524
C +0.35480199 +2.87209790 +3.03308458
C −1.22182333 +0.00989185 −0.86086915
C −2.51793457 +0.18376234 −0.07607855
C −2.44373153 −0.60380745 +1.21567205
C −1.31408308 −0.01316406 +2.06276902
C −3.60914558 −0.42870723 +2.19189213
C −2.99793217 −0.86328626 +3.53875899
C −1.46849066 −0.74744529 +3.38750023
O −1.91113244 +2.94489921 +3.86594946
H −1.69935121 +2.08186059 +4.22610137
C −2.22824975 −2.08392447 +0.91373218
C −4.89348021 −1.14710789 +1.84305848
H −0.96270428 +4.55621687 +3.17087029
H +1.19416400 +3.52260373 +2.77766943
H −0.03453769 +2.50535263 +2.08598041
H +0.73398655 +4.64145053 +4.98837067
H −0.90561751 +4.72796883 +5.61532429
H −0.86945335 +2.47349705 +6.38133671
H +0.68648160 +3.09312394 +6.82077025
H +2.04692153 +0.90041781 +6.67773931

H +1.49230489 −0.27646743 +5.48272511
H +2.60183435 +0.47066004 +3.56467893
H +2.04163277 −0.63886580 +1.63703321
H +0.95214255 −0.00760392 −0.51741569
H −1.25045411 −0.92426623 −1.42889115
H −1.12671466 +0.79557866 −1.61137479
H −2.66641315 +1.23772784 +0.16527815
H −3.36655513 −0.12853567 −0.68583080
H −3.81832539 +0.64292480 +2.23897624
H −3.28997670 −1.88700777 +3.77248325
H −3.37533315 −0.24575929 +4.35174155
H −1.00087452 −1.72940278 +3.33390541
H −0.99766321 −0.22580750 +4.21646359
H −2.33917254 −2.69906063 +1.80433678
H −1.23761275 −2.27040136 +0.50652037
H −2.96277201 −2.42942768 +0.18749720
H −5.67062968 −0.92453070 +2.57177580
H −4.75431028 −2.22696414 +1.83355957
H −5.26614758 −0.84724185 +0.86453142
H −1.60670886 +1.02493052 +2.24663295
51
* E = +1.415 kcal/mol ; (7) 017_314_062_006_321_064_060_306_141
C +0.00000000 +0.00000000 +0.00000000
C +0.00000000 +0.00000000 +1.33852109
C +1.28355980 +0.00000000 +2.05009905
C +1.62504554 +0.66078652 +3.16103202
C +0.83703598 +1.61633479 +3.94407330
C +0.24886772 +2.70499126 +3.42622041
C +0.33471556 +3.09870132 +1.98839242
H +0.56479959 +4.16098412 +1.91031689
C −0.54424566 +3.65000312 +4.28271001
C −0.26882371 +3.50628963 +5.76545143
C −0.27577217 +2.04814122 +6.15360179
C +0.82398123 +1.32513193 +5.42234447
C −1.21781440 +0.02174594 −0.86585032
C −2.51417042 +0.20228791 −0.08420406
C −2.44440765 −0.58837797 +1.20532498
C −1.31726102 −0.00168482 +2.05910896
C −3.61480293 −0.42229026 +2.17657957
C −3.00902251 −0.86875629 +3.52109027

C −1.48031813 −0.73243761 +3.38345248
O −0.05625774 +1.85164163 +7.52943871
H −0.74467832 +2.29119553 +8.02368355
C −2.22879161 −2.06810836 +0.89941161
C −4.89738108 −1.13773986 +1.81505795
H −1.23845341 +1.61021152 +5.86228168
H +0.72753520 +0.25351247 +5.59146429
H +1.77878556 +1.61621731 +5.86757068
H −1.01009266 +4.06037690 +6.34304658
H +0.70895690 +3.92081154 +6.01236092
H −0.34347927 +4.67343463 +3.96563078
H −1.60837090 +3.48928462 +4.08230288
H −0.61546538 +2.94510350 +1.47206090
H +1.09083531 +2.53815021 +1.45177704
H +2.61288198 +0.45727338 +3.56173728
H +2.05420056 −0.61054798 +1.59510069
H +0.95289128 −0.01626269 −0.51608943
H −1.25305700 −0.91024930 −1.43695425
H −1.11392815 +0.80922665 −1.61333788
H −2.65506466 +1.25759962 +0.15923385
H −3.36453890 −0.10174178 −0.69575748
H −3.82639002 +0.64937498 +2.22952620
H −3.29127738 −1.89959716 +3.73419816
H −3.39798811 −0.27059176 +4.34252542
H −0.99646955 −1.70705023 +3.34428002
H −1.03680384 −0.19241902 +4.21276080
H −2.33363415 −2.68435107 +1.78980886
H −1.24044986 −2.25304582 +0.48713355
H −2.96684694 −2.41301681 +0.17651994
H −5.67867437 −0.92239226 +2.54157672
H −4.75639001 −2.21712270 +1.79826833
H −5.26506967 −0.83139415 +0.83662608
H −1.59793389 +1.03982828 +2.24953023
51
* E = +1.507 kcal/mol ; (8) 012_317_063_008_322_062_185_231_033
C +0.00000000 +0.00000000 +0.00000000
C +0.00000000 +0.00000000 +1.33847629
C +1.22833907 +0.00000000 +2.13160865
C +2.40537394 +0.57027410 +1.84890884
C +2.73138576 +1.46422483 +0.72378136

C	+3.78225452	+1.24141334	−0.07488496
C	+4.65313338	+0.03399406	+0.04216915
H	+5.66488720	+0.31343360	+0.33924397
C	+4.15638859	+2.17318825	−1.18857454
C	+3.44128399	+3.50782283	−1.14086860
C	+1.98888717	+3.30206519	−0.80350811
C	+1.87343621	+2.69154257	+0.57251215
C	−1.22762568	−0.01972710	−0.85377219
C	−2.52905886	+0.14672350	−0.07473815
C	−2.44915492	−0.60509487	+1.23834810
C	−1.31662852	+0.00878753	+2.05806159
C	−3.61296375	−0.40509236	+2.21141261
C	−2.99788539	−0.78665249	+3.57203616
C	−1.47031915	−0.64928318	+3.42173012
O	+1.34672500	+4.55192524	−0.88107983
H	+0.44168158	+4.45735274	−0.59186463
C	−2.22235342	−2.09094228	+0.98007066
C	−4.89331881	−1.14338636	+1.89071323
H	+1.55059455	+2.60965920	−1.53320229
H	+0.83664967	+2.43755543	+0.78895778
H	+2.17323323	+3.44029477	+1.30956494
H	+3.53019200	+4.03242609	−2.08964278
H	+3.87780906	+4.14866329	−0.37444870
H	+5.23682624	+2.32686757	−1.17515765
H	+3.95068171	+1.67152504	−2.13881037
H	+4.74085495	−0.46502670	−0.92351073
H	+4.27232397	−0.68939010	+0.75542488
H	+3.21098243	+0.37868529	+2.54738273
H	+1.17010888	−0.55114839	+3.06199684
H	+0.95054308	−0.02025975	−0.51767156
H	−1.24320640	−0.96252918	−1.40758957
H	−1.14722317	+0.75334232	−1.61989282
H	−2.69923743	+1.20373753	+0.14002193
H	−3.37006858	−0.19434957	−0.67952904
H	−3.83081000	+0.66655689	+2.21940241
H	−3.27084799	−1.80914577	+3.83167855
H	−3.38662668	−0.15498630	+4.36751732
H	−0.98863020	−1.62655562	+3.43440837
H	−1.02229413	−0.06328776	+4.22043708
H	−2.30250237	−2.67652492	+1.89389639

H	−1.23824072	−2.27526324	+0.55648307
H	−2.96781783	−2.47126267	+0.28326433
H	−5.67130329	−0.90181027	+2.61237684
H	−4.74511686	−2.22158609	+1.91909593
H	−5.26906875	−0.88152612	+0.90255166
H	−1.58870702	+1.06199277	+2.20071429

51

* E = +1.516 kcal/mol ; (9) 353_038_297_007_322_062_284_208_044

C	+0.00000000	+0.00000000	+0.00000000
C	+0.00000000	+0.00000000	+1.33637647
C	+1.23712557	+0.00000000	+2.11350540
C	+2.34942569	+0.71887839	+1.91849669
C	+2.57522744	+1.84705849	+1.00686739
C	+3.79707331	+2.10035318	+0.50231377
C	+4.98537878	+1.21659835	+0.69843250
H	+5.81964020	+1.78079754	+1.11650009
C	+4.09963688	+3.31802805	−0.32178849
C	+2.89061761	+4.15497846	−0.69005106
C	+1.92394048	+4.20334777	+0.47414770
C	+1.43055801	+2.80163985	+0.75979675
C	−1.22777287	−0.03249081	−0.85334601
C	−2.53039244	+0.11006345	−0.06982549
C	−2.43714664	−0.64662210	+1.23996844
C	−1.31261380	−0.01920911	+2.05979809
C	−3.60049732	−0.46777665	+2.21822075
C	−2.97479379	−0.84405667	+3.57586946
C	−1.44953225	−0.68714967	+3.41991444
O	+2.52114381	+4.78624413	+1.60979746
H	+3.10703802	+4.13645241	+1.99891071
C	−2.18936015	−2.12766396	+0.97415386
C	−4.87059414	−1.22472759	+1.89991952
H	+1.07843488	+4.84384763	+0.23343707
H	+0.81787441	+2.46811704	−0.07586362
H	+0.77866074	+2.81177977	+1.63197636
H	+2.37840917	+3.72139491	−1.54901626
H	+3.19886451	+5.16102763	−0.96573173
H	+4.81359838	+3.92935903	+0.23841027
H	+4.63768516	+3.01320989	−1.22065167
H	+5.32377541	+0.83495734	−0.26534746
H	+4.79325630	+0.36087792	+1.33402251

H	+3.17850653	+0.47770262	+2.56972878
H	+1.25401420	−0.69716964	+2.94299260
H	+0.95313638	−0.00697674	−0.51484755
H	−1.23044859	−0.97066601	−1.41495215
H	−1.16117374	+0.74909847	−1.61241688
H	−2.71494908	+1.16341384	+0.14984664
H	−3.36721088	−0.24090866	−0.67483075
H	−3.83482758	+0.60022925	+2.23020108
H	−3.23405823	−1.87028228	+3.83500980
H	−3.36821097	−0.21835411	+4.37375163
H	−0.95706899	−1.65909358	+3.42376734
H	−1.00261125	−0.10060615	+4.21878141
H	−2.25795353	−2.71911890	+1.88512174
H	−1.20428601	−2.29552620	+0.54563618
H	−2.93181548	−2.51543809	+0.27820134
H	−5.64989520	−0.99609228	+2.62439412
H	−4.70655476	−2.30071560	+1.92587444
H	−5.25316773	−0.96656443	+0.91338236
H	−1.59986914	+1.02901656	+2.20888782

51

* E = +1.574 kcal/mol ; (10) 009_320_063_010_321_062_184_154_316

C	+0.00000000	+0.00000000	+0.00000000
C	+0.00000000	+0.00000000	+1.33646373
C	+1.23406650	+0.00000000	+2.11905297
C	+2.34719435	−0.72456025	+1.94889406
C	+2.58426958	−1.87134314	+1.06653342
C	+3.81837674	−2.16716223	+0.62247236
C	+5.02442960	−1.31916172	+0.86065392
H	+5.43298961	−0.98132081	−0.09225073
C	+4.11959123	−3.40904648	−0.16291737
C	+2.89921209	−4.18954976	−0.60343461
C	+1.88187685	−4.21055084	+0.50350863
C	+1.42864663	−2.79539219	+0.77801242
C	−1.22780346	+0.04856432	−0.85248382
C	−2.50677261	+0.35151156	−0.07573065
C	−2.49053372	−0.36837982	+1.25793714
C	−1.30401386	+0.15994459	+2.06016769
C	−3.62614941	−0.04362124	+2.23144615
C	−3.03965461	−0.45373644	+3.59701729
C	−1.50663596	−0.45445207	+3.43694478

O +0.81255856 −5.02678315 +0.08908028
H +0.12505521 −5.00354888 +0.75131292
C −2.40360950 −1.87498380 +1.03509027
C −4.97022877 −0.66876500 +1.93141887
H +2.34403254 −4.62807228 +1.40512074
H +0.73673276 −2.76783696 +1.62251541
H +0.85870440 −2.46308672 −0.08973417
H +3.16724932 −5.20575712 −0.88386582
H +2.43827101 −3.72451228 −1.47506232
H +4.72991073 −3.14559832 −1.02810267
H +4.76228082 −4.04243845 +0.45638635
H +5.81057461 −1.89947073 +1.34506752
H +4.82899432 −0.43712533 +1.45792251
H +3.16862702 −0.47123292 +2.60453755
H +1.24726750 +0.71318009 +2.93620497
H +0.95139226 −0.04821470 −0.51539488
H −1.32502153 −0.91105435 −1.36964882
H −1.08892488 +0.78398108 −1.64536822
H −2.57835898 +1.42511444 +0.10875090
H −3.37731811 +0.07078583 −0.66974429
H −3.74572679 +1.04323443 +2.21870178
H −3.40176983 −1.44287307 +3.87668939
H −3.36692209 +0.22432324 +4.38198619
H −1.10477344 −1.46691036 +3.46516968
H −1.00323172 +0.10370101 +4.22234847
H −2.51385056 −2.42797480 +1.96622005
H −1.45259355 −2.15768906 +0.59115532
H −3.19722648 −2.20265549 +0.36534165
H −5.71924889 −0.34044846 +2.64949400
H −4.92150684 −1.75512075 +1.98471378
H −5.32522945 −0.39638778 +0.93844049
H −1.47612557 +1.23715262 +2.17563755
51
* E = +1.576 kcal/mol ; (11) 012_315_063_008_321_062_182_054_043
C +0.00000000 +0.00000000 +0.00000000
C +0.00000000 +0.00000000 +1.33529105
C +1.22906403 +0.00000000 +2.13103959
C +2.34667939 +0.70834128 +1.93998209
C +2.65765397 +1.71531853 +0.91738202
C +1.91186291 +2.79804922 +0.66080546

C	+0.64957457	+3.13214641	+1.37960176
H	−0.21803435	+2.87485254	+0.76816669
C	+2.29309215	+3.78922380	−0.39977110
C	+3.40469869	+3.31882079	−1.31337787
C	+4.50051558	+2.69366623	−0.49471210
C	+3.96173591	+1.46798756	+0.20653440
C	−1.22897009	+0.00333719	−0.85210532
C	−2.52187237	+0.20694528	−0.06742752
C	−2.46015708	−0.55434055	+1.24150349
C	−1.31133486	+0.02656032	+2.06150989
C	−3.61506873	−0.33286557	+2.22051896
C	−3.00362025	−0.73933999	+3.57606660
C	−1.47333972	−0.64074830	+3.41969642
O	+5.56477275	+2.37818634	−1.35972714
H	+6.25805907	+1.94785725	−0.86402118
C	−2.27064146	−2.04386401	+0.97378244
C	−4.91403166	−1.03818803	+1.90019026
H	+4.83214650	+3.41888924	+0.25688168
H	+4.69316815	+1.09563442	+0.92854043
H	+3.84084735	+0.67433470	−0.53514529
H	+3.80528289	+4.14276543	−1.89974335
H	+3.03357430	+2.57097821	−2.01488676
H	+1.40469224	+4.03083671	−0.98598531
H	+2.57786897	+4.72589344	+0.08826040
H	+0.59972703	+4.20335718	+1.57393153
H	+0.55646182	+2.60066660	+2.32144915
H	+3.17044864	+0.49181776	+2.61270440
H	+1.21817080	−0.66168680	+2.98929503
H	+0.95030754	−0.01871325	−0.51749554
H	−1.27154673	−0.93964504	−1.40404786
H	−1.13144421	+0.77389136	−1.61854595
H	−2.65466408	+1.26828552	+0.15402539
H	−3.37622209	−0.10276012	−0.67049601
H	−3.80713328	+0.74352172	+2.23690105
H	−3.30221972	−1.75601850	+3.83045419
H	−3.37212781	−0.10286216	+4.37736930
H	−1.01756592	−1.63047558	+3.42069404
H	−1.00535293	−0.07422751	+4.22107713
H	−2.36405826	−2.63315246	+1.88394614
H	−1.29195257	−2.24921670	+0.54729501

H	−3.02659615	−2.40102381	+0.27594381
H	−5.68323630	−0.78312887	+2.62666929
H	−4.79167586	−2.11983553	+1.92051821
H	−5.28709838	−0.76050909	+0.91533021
H	−1.55793003	+1.08501129	+2.21588607

51

* E = +1.580 kcal/mol ; (12) 349_042_297_008_321_062_290_059_045

C	+0.00000000	+0.00000000	+0.00000000
C	+0.00000000	+0.00000000	+1.33557302
C	+1.23541276	+0.00000000	+2.12345128
C	+2.32349376	+0.75132027	+1.93815336
C	+2.56702272	+1.79744766	+0.93367029
C	+1.82043436	+2.90181876	+0.79430242
C	+0.59061121	+3.17662865	+1.59296332
H	−0.30244258	+3.00453282	+0.98832372
C	+2.14498240	+3.95907876	−0.22193477
C	+3.50769077	+3.80738539	−0.86855449
C	+3.76481369	+2.35203324	−1.19561119
C	+3.79050783	+1.54895204	+0.08644632
C	−1.22857322	−0.01469444	−0.85310695
C	−2.52521045	+0.16319643	−0.06748805
C	−2.44905469	−0.59656023	+1.24149864
C	−1.31162548	+0.00586520	+2.06175776
C	−3.60791190	−0.39571725	+2.22066327
C	−2.98892134	−0.78877076	+3.57692523
C	−1.46060319	−0.66399939	+3.41990446
O	+2.78682850	+1.85018223	−2.07978553
H	+1.96177637	+1.78853196	−1.59703908
C	−2.23261637	−2.08242507	+0.97412910
C	−4.89338911	−1.12557327	+1.90099429
H	+4.70975978	+2.24137873	−1.72202000
H	+4.69454669	+1.77884120	+0.65527270
H	+3.84506396	+0.48979881	−0.16466383
H	+4.28461151	+4.15407406	−0.18688184
H	+3.56832642	+4.41145161	−1.77116754
H	+1.36515182	+3.95630559	−0.99021681
H	+2.06429387	+4.93739520	+0.25296404
H	+0.56630907	+4.21847065	+1.91002121
H	+0.51924834	+2.54298673	+2.47062639
H	+3.17256892	+0.54558453	+2.58245081

H	+1.25473066	−0.69550427	+2.95433324
H	+0.95225934	−0.03349412	−0.51578586
H	−1.25286382	−0.95751716	−1.40599121
H	−1.14754926	+0.75781496	−1.61954046
H	−2.68022098	+1.22149140	+0.15324168
H	−3.37254768	−0.16418395	−0.67097321
H	−3.82023915	+0.67688510	+2.23557132
H	−3.26973618	−1.80986582	+3.83368479
H	−3.36813703	−0.15696491	+4.37690563
H	−0.98818774	−1.64580019	+3.42080005
H	−1.00123207	−0.08939882	+4.22058331
H	−2.31723244	−2.67346325	+1.88399425
H	−1.25005443	−2.27119587	+0.54891514
H	−2.98101568	−2.45287678	+0.27516051
H	−5.66758185	−0.88328828	+2.62651022
H	−4.75122602	−2.20471382	+1.92344625
H	−5.27086510	−0.85661751	+0.91541353
H	−1.57837254	+1.05892525	+2.21590414

51

* E = +1.602 kcal/mol ; (13) 012_314_063_008_321_062_060_054_043

C	+0.00000000	+0.00000000	+0.00000000
C	+0.00000000	+0.00000000	+1.33526947
C	+1.22933604	+0.00000000	+2.13070829
C	+2.34655567	+0.70888332	+1.93999127
C	+2.65662011	+1.71827468	+0.91922709
C	+1.90698782	+2.79916821	+0.66642689
C	+0.64429552	+3.13043761	+1.38594007
H	−0.22389380	+2.87175482	+0.77574717
C	+2.28596158	+3.79288263	−0.39261761
C	+3.38606902	+3.31042517	−1.31466685
C	+4.49332832	+2.68672750	−0.50037365
C	+3.96371734	+1.47242260	+0.21565184
C	−1.22902214	+0.00330755	−0.85207633
C	−2.52179931	+0.20761090	−0.06733467
C	−2.46035645	−0.55406790	+1.24141373
C	−1.31132124	+0.02620959	+2.06152194
C	−3.61512671	−0.33262403	+2.22059394
C	−3.00358501	−0.73975673	+3.57594146
C	−1.47328127	−0.64188891	+3.41930628
O	+5.57624345	+2.25971791	−1.29159551

H	+5.93886225	+3.00931811	−1.75837666
C	−2.27128816	−2.04356028	+0.97318038
C	−4.91431604	−1.03750100	+1.90016934
H	+4.83736982	+3.41424360	+0.24356456
H	+4.70498263	+1.12056745	+0.93341131
H	+3.85379001	+0.66810165	−0.51560511
H	+3.76775891	+4.13764072	−1.91471112
H	+3.00467565	+2.55837374	−2.00616381
H	+1.39545590	+4.04415481	−0.97153563
H	+2.58467553	+4.72573158	+0.09470372
H	+0.59236238	+4.20136906	+1.58193237
H	+0.55365911	+2.59811894	+2.32754627
H	+3.17167473	+0.49102524	+2.61019679
H	+1.21960617	−0.66374258	+2.98738104
H	+0.95044299	−0.01923289	−0.51722364
H	−1.27212193	−0.93989376	−1.40361821
H	−1.13161054	+0.77359372	−1.61887989
H	−2.65380236	+1.26900643	+0.15451832
H	−3.37647809	−0.10136713	−0.67036635
H	−3.80692024	+0.74382180	+2.23737726
H	−3.30269979	−1.75633384	+3.83015465
H	−3.37162849	−0.10326973	+4.37746777
H	−1.01796993	−1.63180309	+3.41949492
H	−1.00473817	−0.07615062	+4.22088867
H	−2.36489466	−2.63307955	+1.88316328
H	−1.29259738	−2.24903596	+0.54678726
H	−3.02734553	−2.40028088	+0.27520294
H	−5.68338830	−0.78251397	+2.62683256
H	−4.79220862	−2.11917928	+1.92011806
H	−5.28741039	−0.75941924	+0.91541681
H	−1.55794683	+1.08455484	+2.21669609

51

* E = +1.666 kcal/mol ; (14) 345_049_298_006_321_064_060_310_141

C	+0.00000000	+0.00000000	+0.00000000
C	+0.00000000	+0.00000000	+1.33896031
C	+1.28640534	+0.00000000	+2.04441413
C	+1.64081913	+0.64883813	+3.15972913
C	+0.86821484	+1.60254330	+3.95596169
C	+0.23735276	+2.66700334	+3.43875519
C	+0.32705107	+3.08781326	+2.00977434

H	+0.48819189	+4.16507526	+1.96131319
C	−0.56514319	+3.59072147	+4.30253375
C	−0.87949304	+3.01481911	+5.66545380
C	+0.38304867	+2.46422007	+6.29324394
C	+0.93204706	+1.34787722	+5.43808080
C	−1.21634369	+0.01441878	−0.86786191
C	−2.51527445	+0.18506937	−0.08861021
C	−2.44143200	−0.60310293	+1.20216173
C	−1.31910970	−0.00750481	+2.05649494
C	−3.61481854	−0.44044933	+2.17038687
C	−3.01019866	−0.87893283	+3.51767607
C	−1.48222623	−0.73145389	+3.38419186
O	+1.38494012	+3.45523783	+6.37965308
H	+1.06581739	+4.17901728	+6.91449042
C	−2.21641355	−2.08201258	+0.90006899
C	−4.89292639	−1.16377869	+1.80861916
H	+0.16975027	+2.07370331	+7.29136715
H	+0.40932560	+0.42073567	+5.67989789
H	+1.97310288	+1.18452956	+5.72035802
H	−1.60211390	+2.20237230	+5.57271732
H	−1.33258757	+3.77107466	+6.30796709
H	−0.01500727	+4.52842809	+4.41081661
H	−1.48950293	+3.84621579	+3.78120955
H	−0.59764925	+2.88464855	+1.46578427
H	+1.13357375	+2.59064330	+1.48320256
H	+2.63579617	+0.44338458	+3.54067905
H	+2.05592974	−0.59977827	+1.57326601
H	+0.95340222	−0.01280765	−0.51519882
H	−1.24388620	−0.91756724	−1.43953120
H	−1.11677681	+0.80291433	−1.61487165
H	−2.66510964	+1.23943337	+0.15349053
H	−3.36231004	−0.12653348	−0.70103929
H	−3.83210021	+0.63022351	+2.21945001
H	−3.28570911	−1.91139675	+3.73215591
H	−3.40660459	−0.28131786	+4.33608203
H	−0.98961868	−1.70205135	+3.35544745
H	−1.04648372	−0.17760881	+4.20800285
H	−2.31557975	−2.69633603	+1.79254517
H	−1.22712365	−2.26094809	+0.48732189
H	−2.95282165	−2.43448981	+0.17913030

H	−5.67676152	−0.95028549	+2.53298296
H	−4.74612147	−2.24246199	+1.79522902
H	−5.26032844	−0.86228007	+0.82858239
H	−1.60713041	+1.03233507	+2.24223625

51

* E = +1.669 kcal/mol ; (15) 351_042_297_010_321_062_286_305_336

C	+0.00000000	+0.00000000	+0.00000000
C	+0.00000000	+0.00000000	+1.33767530
C	+1.20468774	+0.00000000	+2.16789917
C	+2.45936453	−0.40333883	+1.92885818
C	+3.07863190	−1.03053011	+0.75593855
C	+2.65202966	−2.16013946	+0.16999665
C	+1.45758132	−2.92783541	+0.61765580
H	+1.68064422	−3.99452736	+0.64163401
C	+3.35294843	−2.75827499	−1.01536625
C	+4.42671249	−1.87549919	−1.61832880
C	+5.23898513	−1.23117943	−0.51477985
C	+4.33811943	−0.33288896	+0.30382601
C	−1.22575523	+0.07253237	−0.85419923
C	−2.50517913	+0.37928149	−0.08226411
C	−2.50015881	−0.35689992	+1.24155990
C	−1.31264402	+0.15179682	+2.05379019
C	−3.63872792	−0.03191460	+2.21117307
C	−3.06508892	−0.46560498	+3.57383050
C	−1.53114001	−0.47707614	+3.42361442
O	+5.85365181	−2.20476590	+0.29830561
H	+5.17549436	−2.60186808	+0.84548990
C	−2.42550732	−1.86197936	+1.00539088
C	−4.98710243	−0.63900129	+1.89393430
H	+6.05655290	−0.65023743	−0.93485145
H	+4.07911117	+0.55921711	−0.26984231
H	+4.88822743	+0.01875709	+1.17732576
H	+3.97275169	−1.08922461	−2.22215358
H	+5.07371790	−2.45817909	−2.27018798
H	+3.78437158	−3.71815404	−0.71715821
H	+2.60519319	−3.01046554	−1.76934979
H	+0.63878734	−2.79128034	−0.09125707
H	+1.10260343	−2.61609059	+1.59433696
H	+3.17125858	−0.18302382	+2.71769005
H	+1.04611015	+0.42763172	+3.15065770

H	+0.94482280	−0.06313159	−0.52235028
H	−1.33148340	−0.87847071	−1.38531485
H	−1.07322999	+0.81607395	−1.63736366
H	−2.56747632	+1.45114853	+0.11497801
H	−3.37547913	+0.11392381	−0.68371980
H	−3.74674261	+1.05624130	+2.21151724
H	−3.43649715	−1.45559296	+3.83748158
H	−3.39123519	+0.20410107	+4.36635500
H	−1.13883412	−1.49328272	+3.44457252
H	−1.03473907	+0.06899454	+4.22176552
H	−2.56805493	−2.42369851	+1.92636011
H	−1.46365362	−2.15173121	+0.59125807
H	−3.20296743	−2.17385959	+0.30949289
H	−5.73722811	−0.31321823	+2.61199687
H	−4.94994103	−1.72644407	+1.93201374
H	−5.33325855	−0.34872876	+0.90289472
H	−1.47892570	+1.22841049	+2.18303311

51

* E = +1.691 kcal/mol ; (16) 017_315_062_006_321_064_298_306_141

C	+0.00000000	+0.00000000	+0.00000000
C	+0.00000000	+0.00000000	+1.33852628
C	+1.28376889	+0.00000000	+2.04959822
C	+1.62567950	+0.66201544	+3.15973818
C	+0.83756116	+1.61922718	+3.94054777
C	+0.25006747	+2.70810438	+3.42222467
C	+0.33829584	+3.09947107	+1.98391852
H	+0.56455170	+4.16241820	+1.90456318
C	−0.54773397	+3.65295214	+4.27438762
C	−0.29402282	+3.50699290	+5.76108667
C	−0.29458698	+2.04922185	+6.14996830
C	+0.81610943	+1.32975983	+5.42019163
C	−1.21797648	+0.02242479	−0.86551766
C	−2.51412357	+0.20306501	−0.08352240
C	−2.44439628	−0.58792562	+1.20582032
C	−1.31700492	−0.00163109	+2.05958819
C	−3.61448446	−0.42195542	+2.17751291
C	−3.00865589	−0.86932120	+3.52165160
C	−1.47998391	−0.73304629	+3.38360219
O	−0.21579569	+1.85861679	+7.54086154
H	+0.60848274	+2.22585782	+7.85599620

C	−2.22904774	−2.06761624	+0.89946605
C	−4.89732498	−1.13687366	+1.81594869
H	−1.25031168	+1.60903507	+5.86210223
H	+0.72717477	+0.25774089	+5.59135166
H	+1.77740566	+1.62458460	+5.85629105
H	−1.04353680	+4.04395976	+6.33881033
H	+0.67983027	+3.93600017	+6.01115788
H	−0.34250985	+4.67646633	+3.96020075
H	−1.60993788	+3.49466262	+4.06316405
H	−0.61035676	+2.94156929	+1.46629216
H	+1.09726050	+2.54027192	+1.44989272
H	+2.61376218	+0.45844146	+3.56016056
H	+2.05404604	−0.61162722	+1.59540282
H	+0.95282096	−0.01677435	−0.51625032
H	−1.25337370	−0.90944656	−1.43678804
H	−1.11384721	+0.81000299	−1.61287214
H	−2.65491507	+1.25834598	+0.16008295
H	−3.36461668	−0.10067233	−0.69498402
H	−3.82590848	+0.64968360	+2.23118460
H	−3.29055157	−1.90038292	+3.73392755
H	−3.39725970	−0.27221104	+4.34380064
H	−0.99608826	−1.70764335	+3.34374240
H	−1.03655465	−0.19391078	+4.21351226
H	−2.33418389	−2.68401578	+1.78969296
H	−1.24084195	−2.25294620	+0.48697575
H	−2.96732394	−2.41204269	+0.17659475
H	−5.67808476	−0.92162182	+2.54298138
H	−4.75668194	−2.21629738	+1.79857745
H	−5.26535480	−0.82982714	+0.83785476
H	−1.59787684	+1.03979210	+2.25026676

51

* E = +1.717 kcal/mol ; (17) 014_316_063_008_322_062_058_230_036

C	+0.00000000	+0.00000000	+0.00000000
C	+0.00000000	+0.00000000	+1.33760678
C	+1.23178555	+0.00000000	+2.12530382
C	+2.38720663	+0.61809775	+1.85687840
C	+2.67271918	+1.57200611	+0.76963485
C	+3.73141668	+1.42341305	−0.03585944
C	+4.64309162	+0.24205611	+0.02191805
H	+5.64429795	+0.53829825	+0.33829070

C	+4.07597933	+2.42421065	−1.09792930
C	+3.33604872	+3.73835327	−0.95288990
C	+1.88074503	+3.47989309	−0.64168898
C	+1.76645103	+2.76930546	+0.68016620
C	−1.22832065	−0.00882674	−0.85289134
C	−2.52781967	+0.16721733	−0.07199455
C	−2.45323600	−0.58566911	+1.24098724
C	−1.31443011	+0.01820788	+2.05910072
C	−3.61330384	−0.37392989	+2.21632893
C	−2.99958904	−0.76033105	+3.57631663
C	−1.47092670	−0.63843718	+3.42297017
O	+1.13235846	+4.66786127	−0.54132986
H	+1.20595869	+5.15496976	−1.35901819
C	−2.24096731	−2.07360183	+0.98260245
C	−4.90152550	−1.09984235	+1.89875164
H	+1.45844339	+2.83576460	−1.42288250
H	+0.73526222	+2.47205716	+0.84846983
H	+2.01715573	+3.47807930	+1.47251357
H	+3.43607795	+4.33064014	−1.86371180
H	+3.75758195	+4.32665334	−0.13734265
H	+5.15344054	+2.59773575	−1.08806461
H	+3.86545451	+1.98068492	−2.07577965
H	+4.75033777	−0.20317847	−0.96795022
H	+4.28409201	−0.52983083	+0.69449635
H	+3.20604080	+0.42456140	+2.53928477
H	+1.19618951	−0.58952989	+3.03333248
H	+0.95084328	−0.02220451	−0.51706551
H	−1.25185023	−0.94936371	−1.41041341
H	−1.14213828	+0.76843942	−1.61392262
H	−2.68756735	+1.22556763	+0.14233098
H	−3.37209154	−0.16723990	−0.67609757
H	−3.82051460	+0.69966803	+2.22364708
H	−3.28259668	−1.77972776	+3.83773925
H	−3.38032897	−0.12361385	+4.37167250
H	−0.99931431	−1.62072273	+3.43450148
H	−1.01484773	−0.05678294	+4.22031286
H	−2.32561776	−2.65892459	+1.89624629
H	−1.25918581	−2.26705092	+0.55756676
H	−2.99093986	−2.44665068	+0.28660668
H	−5.67613361	−0.84867292	+2.62085140

H	−4.76476024	−2.17953437	+1.92925979
H	−5.27564944	−0.83595715	+0.91050168
H	−1.57607324	+1.07391918	+2.20156338

51

* E = +1.730 kcal/mol ; (18) 344_045_300_009_322_062_058_123_324

C	+0.00000000	+0.00000000	+0.00000000
C	+0.00000000	+0.00000000	+1.33749000
C	+1.23226086	+0.00000000	+2.12641307
C	+2.38096881	−0.62673002	+1.85170875
C	+2.61057107	−1.58063920	+0.74949144
C	+3.55416675	−1.39734576	−0.17832672
C	+4.41223493	−0.17716564	−0.23630222
H	+4.33536331	+0.29552906	−1.21653996
C	+3.77354836	−2.37499105	−1.29144708
C	+3.12430671	−3.72333057	−1.05706834
C	+1.71441459	−3.55517169	−0.53075949
C	+1.74233467	−2.80868388	+0.77774408
C	−1.22715151	+0.01613898	−0.85164958
C	−2.52200153	+0.24855059	−0.07683065
C	−2.47058280	−0.48132691	+1.25020043
C	−1.31205402	+0.10147357	+2.05807644
C	−3.62164451	−0.22077967	+2.22517835
C	−3.01904355	−0.61375061	+3.58766008
C	−1.48819004	−0.52443940	+3.43436211
O	+0.91455581	−2.79963207	−1.41924360
H	+0.89846998	−3.23396838	−2.26955103
C	−2.31054269	−1.97824150	+1.00658107
C	−4.93523002	−0.90496157	+1.91870895
H	+1.25595632	−4.53492562	−0.37324891
H	+2.09720489	−3.47864086	+1.56364808
H	+0.72762059	−2.52919960	+1.04838719
H	+3.69399817	−4.29155139	−0.32121273
H	+3.12450905	−4.31193795	−1.97586825
H	+3.38366961	−1.93055399	−2.21115498
H	+4.84452051	−2.50296853	−1.45763901
H	+5.46362533	−0.43155266	−0.09552151
H	+4.13353403	+0.55952000	+0.51033990
H	+3.21303152	−0.44936431	+2.52266725
H	+1.20017759	+0.59314802	+3.03338185
H	+0.94456302	−0.04489865	−0.52355325

H	−1.26469252	−0.93893968	−1.38273181
H	−1.11614587	+0.77224654	−1.62987026
H	−2.65069221	+1.31538916	+0.11692120
H	−3.37640686	−0.07216435	−0.67458090
H	−3.79220689	+0.85948240	+2.22161874
H	−3.32405673	−1.62643493	+3.85090450
H	−3.38891324	+0.03271377	+4.38050988
H	−1.02860080	−1.51155661	+3.46659542
H	−1.02432661	+0.06326510	+4.22282718
H	−2.33693853	−2.54732595	+1.93412822
H	−1.37511563	−2.20295596	+0.50020350
H	−3.12159475	−2.34141462	+0.37691274
H	−5.70188907	−0.61002504	+2.63284585
H	−4.83864169	−1.98801675	+1.97263570
H	−5.29662827	−0.64866729	+0.92362509
H	−1.53861718	+1.16840619	+2.17470098

51

* E = +1.838 kcal/mol ; (19) 014_316_062_007_322_062_297_230_036

C	+0.00000000	+0.00000000	+0.00000000
C	+0.00000000	+0.00000000	+1.33775186
C	+1.23158049	+0.00000000	+2.12558416
C	+2.39081650	+0.61048147	+1.85477451
C	+2.68401161	+1.55668746	+0.76308587
C	+3.74683579	+1.40359013	−0.03633301
C	+4.65611643	+0.22102596	+0.03331392
H	+5.65572990	+0.51785321	+0.35398800
C	+4.09891513	+2.39672182	−1.10313324
C	+3.35860963	+3.71326999	−0.98012400
C	+1.90150771	+3.46378081	−0.67110202
C	+1.77945825	+2.75518839	+0.65697528
C	−1.22774263	−0.00956567	−0.85338145
C	−2.52796521	+0.16192092	−0.07275642
C	−2.45179417	−0.59117891	+1.23996603
C	−1.31484831	+0.01540268	+2.05883928
C	−3.61281470	−0.38299291	+2.21493410
C	−2.99870976	−0.76836836	+3.57502435
C	−1.47032969	−0.64211559	+3.42244048
O	+1.13404270	+4.64249236	−0.70679393
H	+1.44534579	+5.23227034	−0.02211811
C	−2.23542960	−2.07846387	+0.98123921

C	−4.89892831	−1.11220758	+1.89646744
H	+1.47714639	+2.82921506	−1.45033495
H	+0.74656718	+2.46219888	+0.82326346
H	+2.03265553	+3.45774189	+1.45847183
H	+3.44948070	+4.29856241	−1.89282597
H	+3.78969720	+4.30528436	−0.16858904
H	+5.17676209	+2.56777458	−1.08808380
H	+3.89411897	+1.94564186	−2.07854018
H	+4.76874998	−0.22856977	−0.95385816
H	+4.29219262	−0.54726115	+0.70728956
H	+3.20786228	+0.41487037	+2.53877138
H	+1.19322451	−0.58450015	+3.03673737
H	+0.95088105	−0.02231703	−0.51705639
H	−1.24836121	−0.94902710	−1.41274584
H	−1.14276212	+0.76938382	−1.61264762
H	−2.69137216	+1.21973959	+0.14138675
H	−3.37078865	−0.17487855	−0.67743707
H	−3.82299274	+0.69005439	+2.22255438
H	−3.27882833	−1.78872884	+3.83570592
H	−3.38168802	−0.13331714	+4.37066012
H	−0.99602303	−1.62309240	+3.43372476
H	−1.01627474	−0.05978729	+4.22053061
H	−2.31862796	−2.66438193	+1.89464692
H	−1.25320206	−2.26922945	+0.55608194
H	−2.98430314	−2.45317750	+0.28501835
H	−5.67449796	−0.86358692	+2.61840794
H	−4.75917400	−2.19152285	+1.92638829
H	−5.27333431	−0.84873616	+0.90825457
H	−1.57985801	+1.07034321	+2.20129654

51

* E = +1.860 kcal/mol ; (20) 012_315_063_008_321_062_298_053_043

C	+0.00000000	+0.00000000	+0.00000000
C	+0.00000000	+0.00000000	+1.33523544
C	+1.22908237	+0.00000000	+2.13112467
C	+2.34568064	+0.71056945	+1.94237575
C	+2.65585061	+1.72107288	+0.92283508
C	+1.90653170	+2.80158583	+0.66722675
C	+0.64231264	+3.13101796	+1.38487407
H	−0.22416225	+2.87080347	+0.77297810
C	+2.28634391	+3.79712824	−0.38973222

C	+3.39610797	+3.32737395	−1.30744937
C	+4.50132582	+2.70061665	−0.49315520
C	+3.96661430	+1.48126744	+0.22190096
C	−1.22869383	+0.00395891	−0.85253826
C	−2.52143747	+0.20921877	−0.06794204
C	−2.46075196	−0.55248554	+1.24084674
C	−1.31146119	+0.02692275	+2.06120064
C	−3.61548303	−0.33000785	+2.21985876
C	−3.00447170	−0.73751636	+3.57533181
C	−1.47405248	−0.64087120	+3.41904109
O	+5.63955783	+2.39716973	−1.26146373
H	+5.39991338	+1.75277340	−1.92537290
C	−2.27291311	−2.04215133	+0.97277575
C	−4.91518638	−1.03385295	+1.89930001
H	+4.84737353	+3.42372910	+0.24535421
H	+4.70701437	+1.12507431	+0.93856854
H	+3.84586998	+0.66955754	−0.50426361
H	+3.79086158	+4.15218715	−1.89706230
H	+3.00844063	+2.58151857	−2.00651233
H	+1.39676821	+4.04557592	−0.97139163
H	+2.57850993	+4.73039430	+0.10025542
H	+0.58859390	+4.20188164	+1.58001845
H	+0.55084317	+2.59890768	+2.32649186
H	+3.16972564	+0.49411569	+2.61442278
H	+1.21898398	−0.66395722	+2.98760784
H	+0.95075287	−0.01927071	−0.51679701
H	−1.27257102	−0.93932724	−1.40392583
H	−1.13086470	+0.77430360	−1.61925953
H	−2.65264563	+1.27071692	+0.15372351
H	−3.37616225	−0.09913221	−0.67115942
H	−3.80640167	+0.74657852	+2.23652054
H	−3.30441290	−1.75380475	+3.82964050
H	−3.37209685	−0.10064343	+4.37669836
H	−1.01951969	−1.63114997	+3.41950922
H	−1.00530828	−0.07531952	+4.22061695
H	−2.36679467	−2.63143182	+1.88286070
H	−1.29449522	−2.24861651	+0.54616441
H	−3.02947785	−2.39835262	+0.27510317
H	−5.68409203	−0.77803852	+2.62580724
H	−4.79408612	−2.11563495	+1.91950633

H	−5.28796628	−0.75557388	+0.91449345
H	−1.55719264	+1.08546401	+2.21616290

51

* E = +1.930 kcal/mol ; (21) 010_319_063_011_320_062_060_154_315

C	+0.00000000	+0.00000000	+0.00000000
C	+0.00000000	+0.00000000	+1.33633561
C	+1.23433745	+0.00000000	+2.11803750
C	+2.34633291	−0.72655540	+1.95058523
C	+2.58441448	−1.87534656	+1.07024272
C	+3.82030173	−2.16831686	+0.62967986
C	+5.02607685	−1.32057079	+0.87089717
H	+5.43925207	−0.98440606	−0.08070706
C	+4.12443349	−3.40935577	−0.15595196
C	+2.89949781	−4.17194381	−0.61582264
C	+1.87209223	−4.20430106	+0.48883607
C	+1.42656716	−2.79658719	+0.78431405
C	−1.22821181	+0.04555105	−0.85181121
C	−2.50567335	+0.35322493	−0.07478063
C	−2.49074101	−0.36617612	+1.25922632
C	−1.30324741	+0.16071319	+2.06090835
C	−3.62499317	−0.03589844	+2.23262105
C	−3.03935025	−0.44673253	+3.59844090
C	−1.50642546	−0.45423968	+3.43731769
O	+0.72034122	−4.93020048	+0.12886702
H	+0.96719100	−5.83004134	−0.07269283
C	−2.40853478	−1.87320468	+1.03888632
C	−4.97097460	−0.65749937	+1.93376189
H	+2.32251617	−4.63831986	+1.38860631
H	+0.73927892	−2.79133952	+1.62915639
H	+0.84861316	−2.45561242	−0.07425895
H	+3.17677573	−5.18250058	−0.91896178
H	+2.44724946	−3.68870840	−1.48219491
H	+4.74879813	−3.14853148	−1.01189108
H	+4.75123403	−4.05351022	+0.46873927
H	+5.81018712	−1.89944724	+1.36063412
H	+4.82794947	−0.43737330	+1.46544410
H	+3.16682787	−0.47380085	+2.60756109
H	+1.24832399	+0.71417297	+2.93438207
H	+0.95148783	−0.04787897	−0.51541055
H	−1.32705966	−0.91736020	−1.36240199

H	−1.08954986	+0.77672113	−1.64879095
H	−2.57400319	+1.42745994	+0.10817251
H	−3.37721444	+0.07390969	−0.66808097
H	−3.74141384	+1.05144779	+2.21903539
H	−3.40481347	−1.43422872	+3.87918285
H	−3.36331795	+0.23389230	+4.38271580
H	−1.10994455	−1.46862343	+3.46312244
H	−1.00014898	+0.10126999	+4.22292761
H	−2.52782129	−2.42648235	+1.96823478
H	−1.45741002	−2.16835007	+0.60443670
H	−3.19931953	−2.19764262	+0.36410374
H	−5.71861706	−0.32738888	+2.65260468
H	−4.92420549	−1.74387812	+1.98644900
H	−5.32598332	−0.38402530	+0.94101805
H	−1.47328821	+1.23835831	+2.17699526

51

* E = +2.128 kcal/mol ; (22) 010_319_063_011_321_062_296_154_315

C	+0.00000000	+0.00000000	+0.00000000
C	+0.00000000	+0.00000000	+1.33646953
C	+1.23441744	+0.00000000	+2.11851515
C	+2.34442930	−0.72959826	+1.95275108
C	+2.57749660	−1.88268957	+1.07683313
C	+3.81020401	−2.17989851	+0.63022999
C	+5.01620319	−1.32924376	+0.85951450
H	+5.42940377	−1.00716816	−0.09684855
C	+4.11147221	−3.42753453	−0.14583034
C	+2.88899015	−4.20954524	−0.58053796
C	+1.86631123	−4.22726417	+0.52874942
C	+1.42004739	−2.81067180	+0.80692503
C	−1.22841269	+0.05414370	−0.85162489
C	−2.50439794	+0.36608520	−0.07369650
C	−2.49239578	−0.35621684	+1.25865425
C	−1.30265954	+0.16336100	+2.06151040
C	−3.62559005	−0.02600312	+2.23307635
C	−3.04069299	−0.44265249	+3.59753339
C	−1.50781353	−0.45526365	+3.43598988
O	+0.77514968	−5.06687671	+0.23950049
H	+0.31261476	−4.71836642	−0.52087703
C	−2.41446767	−1.86306467	+1.03490390
C	−4.97322984	−0.64297531	+1.93202916

H	+2.31652234	−4.65123777	+1.42630103
H	+0.73228031	−2.79238407	+1.65147241
H	+0.84357911	−2.46494022	−0.05500442
H	+3.15728894	−5.22642443	−0.85916443
H	+2.43842701	−3.74006244	−1.45868163
H	+4.72556541	−3.17236252	−1.01093370
H	+4.74915732	−4.06016112	+0.47927326
H	+5.79988523	−1.90179450	+1.35708281
H	+4.81829912	−0.43765462	+1.44152955
H	+3.16632425	−0.47708479	+2.60815169
H	+1.24955097	+0.71719770	+2.93206129
H	+0.95158842	−0.04897603	−0.51530239
H	−1.33417686	−0.90594834	−1.36689993
H	−1.08601661	+0.78699387	−1.64631899
H	−2.56688247	+1.44008284	+0.11177957
H	−3.37760979	+0.09310239	−0.66747585
H	−3.73886765	+1.06161582	+2.22252158
H	−3.40968278	−1.42934015	+3.87635131
H	−3.36183724	+0.23719374	+4.38350951
H	−1.11479740	−1.47110526	+3.45892635
H	−0.99925847	+0.09583927	+4.22307190
H	−2.53878099	−2.41908910	+1.96177849
H	−1.45900770	−2.15423902	+0.60659579
H	−3.20228733	−2.18352602	+0.35462087
H	−5.71978022	−0.31332854	+2.65206567
H	−4.92995310	−1.72969329	+1.98138156
H	−5.32787518	−0.36495303	+0.94042198
H	−1.46827411	+1.24127128	+2.18063915

51

* E = +2.320 kcal/mol ; (23) 341_048_299_008_321_062_185_056_041

C	+0.00000000	+0.00000000	+0.00000000
C	+0.00000000	+0.00000000	+1.33559009
C	+1.22747987	+0.00000000	+2.13452259
C	+2.35706425	+0.68238255	+1.92168710
C	+2.68727379	+1.63188645	+0.85036807
C	+1.98882612	+2.73619490	+0.56030584
C	+0.72906873	+3.12592828	+1.25648110
H	−0.13594539	+2.92151458	+0.62157422
C	+2.40588020	+3.65765672	−0.54604829
C	+3.85099133	+3.49286534	−0.96504876

C	+4.16497054	+2.03254544	−1.16904291
C	+3.95917472	+1.27628116	+0.12595708
C	−1.22874121	+0.00984141	−0.85246859
C	−2.52115801	+0.22371453	−0.06950942
C	−2.46571879	−0.53405292	+1.24132564
C	−1.31243470	+0.03963776	+2.05977440
C	−3.61941575	−0.29936825	+2.21897720
C	−3.01255455	−0.70583932	+3.57648826
C	−1.48150711	−0.62089796	+3.42034481
O	+3.29756049	+1.57332456	−2.18415526
H	+3.41493896	+0.63286494	−2.29632725
C	−2.28877572	−2.02594693	+0.97852846
C	−4.92423167	−0.99453143	+1.90014770
H	+5.20220393	+1.91533092	−1.49401396
H	+4.81668812	+1.44782876	+0.78252522
H	+3.96637665	+0.20267720	−0.08023950
H	+4.51379741	+3.88298368	−0.19278771
H	+4.04917851	+4.04530249	−1.88113827
H	+1.75785593	+3.47714353	−1.40681981
H	+2.22472748	+4.68850454	−0.24081651
H	+0.72641171	+4.19433637	+1.46907786
H	+0.58744431	+2.58457988	+2.18612439
H	+3.17795744	+0.47564204	+2.60193122
H	+1.20202639	−0.63222103	+3.01432366
H	+0.94869440	−0.01343346	−0.52038153
H	−1.27781202	−0.93447423	−1.40193846
H	−1.12351426	+0.77771117	−1.62020699
H	−2.64717549	+1.28642195	+0.14868867
H	−3.37707994	−0.08213426	−0.67247286
H	−3.80215140	+0.77867877	+2.23105729
H	−3.32007145	−1.71896855	+3.83462926
H	−3.37608195	−0.06306498	+4.37507102
H	−1.03406212	−1.61448359	+3.42506346
H	−1.00924761	−0.05553613	+4.22004383
H	−2.39163884	−2.61198190	+1.88982233
H	−1.30997477	−2.24092988	+0.55715819
H	−3.04459691	−2.37822853	+0.27805236
H	−5.69176670	−0.73042175	+2.62520130
H	−4.81114143	−2.07713742	+1.92412109
H	−5.29411551	−0.71691523	+0.91405776

H −1.54951734 +1.10110961 +2.20912165

51

* E = +2.361 kcal/mol ; (24) 348_043_298_007_322_062_192_207_043

C +0.00000000 +0.00000000 +0.00000000

C +0.00000000 +0.00000000 +1.33653476

C +1.23596584 +0.00000000 +2.11616692

C +2.35727074 +0.70613315 +1.91984291

C +2.60706658 +1.83074232 +1.01185392

C +3.83733845 +2.07965684 +0.53333094

C +5.02156573 +1.19944144 +0.76251376

H +5.83148873 +1.76605262 +1.22332133

C +4.15965410 +3.30478487 −0.26573200

C +2.95174279 +4.08666427 −0.73571420

C +1.93989510 +4.17945606 +0.37868293

C +1.47326125 +2.78827423 +0.74741810

C −1.22706828 −0.03538757 −0.85456854

C −2.53069954 +0.10395872 −0.07214108

C −2.43649259 −0.65305579 +1.23728628

C −1.31398836 −0.02328055 +2.05815843

C −3.60133306 −0.47803213 +2.21447898

C −2.97588828 −0.85321076 +3.57250055

C −1.45093717 −0.69266536 +3.41762938

O +2.57755395 +4.83776985 +1.45073883

H +2.04356741 +4.75015987 +2.23681773

C −2.18449731 −2.13330508 +0.97086381

C −4.86907323 −1.23828696 +1.89463010

H +1.07728647 +4.76604507 +0.05071123

H +0.83114601 +2.42619291 −0.05433492

H +0.82994935 +2.82892493 +1.62960907

H +2.48198382 +3.59156465 −1.58511256

H +3.24701468 +5.08283470 −1.05838891

H +4.78170756 +3.94706621 +0.36189606

H +4.78129516 +3.02599406 −1.11783820

H +5.40328153 +0.83448760 −0.19145304

H +4.81133214 +0.33335119 +1.37814425

H +3.17928236 +0.45685052 +2.57693383

H +1.24536831 −0.69269486 +2.94959310

H +0.95361598 −0.00476022 −0.51400047

H −1.22754101 −0.97381737 −1.41580278

H −1.16232332 +0.74588354 −1.61414541

H −2.71787590 +1.15685868 +0.14807783
H −3.36650855 −0.24845641 −0.67777880
H −3.83875806 +0.58938390 +2.22662332
H −3.23300179 −1.88011217 +3.83111207
H −3.37166413 −0.22888673 +4.37040844
H −0.95609355 −1.66331365 +3.42086997
H −1.00553563 −0.10640644 +4.21759920
H −2.25225479 −2.72540798 +1.88149385
H −1.19846545 −2.29787031 +0.54340298
H −2.92522688 −2.52272052 +0.27397054
H −5.64975079 −1.01237826 +2.61852465
H −4.70190608 −2.31379093 +1.92007752
H −5.25139117 −0.98071905 +0.90783489
H −1.60536646 +1.02396796 +2.20784967
51
* E = +2.383 kcal/mol ; (25) 342_047_299_007_320_064_059_131_134
C +0.00000000 +0.00000000 +0.00000000
C +0.00000000 +0.00000000 +1.33698730
C +1.26784469 +0.00000000 +2.08233203
C +1.58042975 +0.76903830 +3.12847008
C +0.79640340 +1.89933575 +3.66304515
C +0.50608929 +2.01227364 +4.96368334
C +0.83789363 +0.95663554 +5.96554814
H −0.05514991 +0.66946417 +6.52266656
C −0.22707421 +3.19581307 +5.51438204
C −0.20398974 +4.39597358 +4.59190198
C −0.55473272 +3.98015379 +3.17939739
C +0.44839141 +2.97529370 +2.66932311
C −1.22628866 +0.04869913 −0.85526819
C −2.50884057 +0.28180339 −0.06251294
C −2.46187241 −0.51465227 +1.22479056
C −1.30829623 +0.02588748 +2.07039231
C −3.61300032 −0.30697248 +2.21123292
C −3.00714628 −0.78199489 +3.54760301
C −1.47560593 −0.71780505 +3.38730276
O −1.82636783 +3.36379166 +3.13030358
H −2.48354380 +3.97384881 +3.45846444
C −2.30419232 −2.00054292 +0.91201758
C −4.92633247 −0.97169388 +1.86329158
H −0.54856890 +4.85136651 +2.51951051

H	+1.36349349	+3.50050283	+2.38732366
H	+0.06907899	+2.53147628	+1.75072561
H	+0.79120579	+4.84146625	+4.58112780
H	−0.89034539	+5.16628764	+4.94686188
H	−1.25981397	+2.89881071	+5.71375530
H	+0.19747078	+3.46265110	+6.48341488
H	+1.55565268	+1.32706986	+6.69880876
H	+1.24289383	+0.06048538	+5.50614649
H	+2.54329520	+0.59482281	+3.59366771
H	+2.01550082	−0.70774002	+1.74565118
H	+0.95042818	−0.02491382	−0.52015074
H	−1.29855554	−0.88713178	−1.41662498
H	−1.10477699	+0.82541891	−1.61146709
H	−2.60297634	+1.34050781	+0.18566681
H	−3.37485650	+0.01313519	−0.66919764
H	−3.77855298	+0.77151317	+2.27225765
H	−3.33564824	−1.79793532	+3.76790320
H	−3.35263822	−0.16157717	+4.37166971
H	−1.04292387	−1.71552917	+3.32461919
H	−0.98860332	−0.20744388	+4.21160305
H	−2.44029637	−2.61663507	+1.79834497
H	−1.32056041	−2.22288621	+0.50581771
H	−3.05000410	−2.31247936	+0.18172929
H	−5.68967947	−0.73167138	+2.60137952
H	−4.82719542	−2.05577292	+1.83793513
H	−5.29494856	−0.64538166	+0.89149191
H	−1.55243892	+1.07112336	+2.28598020

51

* E = +2.462 kcal/mol ; (26) 342_047_299_008_321_062_059_059_040

C	+0.00000000	+0.00000000	+0.00000000
C	+0.00000000	+0.00000000	+1.33608276
C	+1.22885937	+0.00000000	+2.13296787
C	+2.35832006	+0.67681760	+1.90694512
C	+2.66928798	+1.60800867	+0.81314878
C	+1.98513499	+2.72633722	+0.54858096
C	+0.76411730	+3.14822075	+1.29356582
H	−0.12851941	+2.96920686	+0.68991810
C	+2.36542692	+3.62564032	−0.58842536
C	+3.77403525	+3.40203541	−1.09549520
C	+4.03307753	+1.92451863	−1.28977982

C	+3.88356605	+1.19712568	+0.02305296
C	−1.23167711	+0.00272570	−0.84847529
C	−2.52556471	+0.20478638	−0.06476898
C	−2.46207219	−0.55094864	+1.24684596
C	−1.31174839	+0.03299249	+2.06242963
C	−3.61615239	−0.32451662	+2.22590331
C	−3.00400639	−0.72443429	+3.58290100
C	−1.47401760	−0.62724984	+3.42404448
O	+3.09615455	+1.35263562	−2.18001007
H	+3.15122533	+1.79824325	−3.02266638
C	−2.27344910	−2.04155033	+0.98503395
C	−4.91583421	−1.03060586	+1.90994909
H	+5.04423459	+1.76543591	−1.67314828
H	+4.78307737	+1.34695468	+0.62497114
H	+3.83219486	+0.12882617	−0.18648294
H	+4.49729964	+3.78623874	−0.37554120
H	+3.93694268	+3.94615328	−2.02714265
H	+1.65046982	+3.46866787	−1.40065321
H	+2.24348952	+4.66495629	−0.28182163
H	+0.79670831	+4.21537933	+1.51126940
H	+0.64226649	+2.60523157	+2.22491085
H	+3.18664931	+0.47845257	+2.58023800
H	+1.20506628	−0.62569214	+3.01744612
H	+0.94555723	−0.01330939	−0.52654972
H	−1.27256451	−0.94048609	−1.40030973
H	−1.13324813	+0.77224392	−1.61555893
H	−2.66245279	+1.26632646	+0.15292274
H	−3.37921239	−0.11012033	−0.66651914
H	−3.80763503	+0.75206453	+2.23696669
H	−3.30291539	−1.73980011	+3.84248492
H	−3.37159915	−0.08383727	+4.38145031
H	−1.01857938	−1.61718943	+3.42918809
H	−1.00506271	−0.05738225	+4.22256080
H	−2.36717846	−2.62750232	+1.89742517
H	−1.29472010	−2.24818784	+0.55951136
H	−3.02925187	−2.40104461	+0.28816606
H	−5.68474296	−0.77129937	+2.63535291
H	−4.79399241	−2.11221295	+1.93582832
H	−5.28889892	−0.75796335	+0.92363058
H	−1.55758072	+1.09257818	+2.21113790

51

* E = +2.480 kcal/mol ; (27) 341_049_299_008_320_063_180_130_133

C	+0.00000000	+0.00000000	+0.00000000
C	+0.00000000	+0.00000000	+1.33668830
C	+1.26365031	+0.00000000	+2.09018405
C	+1.57094910	+0.78569243	+3.12501488
C	+0.79198929	+1.93455748	+3.62813944
C	+0.47164808	+2.06938355	+4.91992018
C	+0.76660320	+1.02244245	+5.94221979
H	−0.14225564	+0.75535469	+6.48327967
C	−0.24706419	+3.27615462	+5.43795468
C	−0.15203085	+4.46967279	+4.51181648
C	−0.48870778	+4.05709968	+3.10026252
C	+0.49126230	+3.00831351	+2.61642218
C	−1.22610733	+0.04033413	−0.85656726
C	−2.51057425	+0.27007515	−0.06608423
C	−2.46333445	−0.51977867	+1.22525453
C	−1.30907396	+0.01973799	+2.06878186
C	−3.61523869	−0.31282095	+2.21089656
C	−3.00777136	−0.78022659	+3.54958433
C	−1.47622924	−0.71803986	+3.38845062
O	−1.81375173	+3.56849217	+3.13165418
H	−2.06659830	+3.29367238	+2.25308341
C	−2.30248292	−2.00620700	+0.91626822
C	−4.92690415	−0.98190197	+1.86538736
H	−0.43649746	+4.92626817	+2.43936553
H	+1.42683175	+3.50038999	+2.33849181
H	+0.12578150	+2.55661383	+1.69239503
H	+0.86124915	+4.87108216	+4.52007025
H	−0.82459281	+5.26109498	+4.83556389
H	−1.29673589	+3.01813863	+5.59327955
H	+0.15007606	+3.53402431	+6.42083323
H	+1.47137739	+1.39599526	+6.68625626
H	+1.17230690	+0.11493828	+5.50581864
H	+2.52751176	+0.61203590	+3.60357078
H	+2.00633770	−0.72165369	+1.77307317
H	+0.95047424	−0.02192571	−0.51976840
H	−1.29533515	−0.89801040	−1.41362725
H	−1.10801547	+0.81352336	−1.61677581
H	−2.61134800	+1.33134907	+0.17440607

H	−3.37579984	−0.00142339	−0.67203638
H	−3.78876431	+0.76529752	+2.26960708
H	−3.33658555	−1.79463233	+3.77523096
H	−3.35238534	−0.15481917	+4.36980481
H	−1.04307821	−1.71542328	+3.32776429
H	−0.98639035	−0.20393187	+4.20858852
H	−2.43745933	−2.61931059	+1.80455682
H	−1.31845507	−2.22731614	+0.51070842
H	−3.04797748	−2.32134381	+0.18734949
H	−5.68991030	−0.73984505	+2.60273714
H	−4.82520840	−2.06567355	+1.84510016
H	−5.29677094	−0.66065737	+0.89243657
H	−1.55099200	+1.06504290	+2.29167760

51

* E = +2.642 kcal/mol ; (28) 016_314_063_009_321_062_182_302_336

C	+0.00000000	+0.00000000	+0.00000000
C	+0.00000000	+0.00000000	+1.33766660
C	+1.20521703	+0.00000000	+2.16829580
C	+2.45641771	−0.40621670	+1.92102919
C	+3.06941611	−1.01656945	+0.73455233
C	+2.67542338	−2.16023653	+0.16024301
C	+1.48181184	−2.93706996	+0.59605029
H	+1.73321792	−3.99196068	+0.70281750
C	+3.40728805	−2.74897508	−1.01055961
C	+4.79277659	−2.17548986	−1.21837712
C	+4.73987229	−0.67608712	−1.12210736
C	+4.29502965	−0.27343144	+0.26537938
C	−1.22660549	+0.05695851	−0.85416119
C	−2.51062206	+0.34471222	−0.08262058
C	−2.49448071	−0.39043315	+1.24165010
C	−1.31449964	+0.13747577	+2.05333804
C	−3.63797305	−0.08192514	+2.21041399
C	−3.05831099	−0.50442133	+3.57405680
C	−1.52433434	−0.49102496	+3.42475726
O	+6.01814165	−0.17309434	−1.42757688
H	+6.01330365	+0.77740777	−1.33682303
C	−2.39545601	−1.89422191	+1.00636075
C	−4.97658492	−0.71043487	+1.89345629
H	+4.01074452	−0.30610724	−1.85205828
H	+4.09282904	+0.79937657	+0.29501254

H	+5.12436417	−0.44794994	+0.95624825
H	+5.19779852	−2.46899904	−2.18431199
H	+5.47576210	−2.54446140	−0.45274865
H	+3.46821696	−3.82978560	−0.88068436
H	+2.80153584	−2.60101979	−1.91018957
H	+0.69600070	−2.87354315	−0.15974793
H	+1.06903265	−2.57661317	+1.53175988
H	+3.17468368	−0.19759192	+2.70825429
H	+1.04801201	+0.42025205	+3.15437423
H	+0.94509465	−0.05678581	−0.52245239
H	−1.31854964	−0.89553383	−1.38512707
H	−1.08489711	+0.80277552	−1.63724372
H	−2.58961993	+1.41562178	+0.11402385
H	−3.37667306	+0.06550305	−0.68398244
H	−3.76281118	+1.00447820	+2.20912726
H	−3.41368018	−1.50005367	+3.83859651
H	−3.39609970	+0.16077715	+4.36553903
H	−1.11523151	−1.50051580	+3.44931705
H	−1.03753669	+0.06548009	+4.22164536
H	−2.52721136	−2.45785036	+1.92785764
H	−1.42962668	−2.16800417	+0.59043511
H	−3.16894870	−2.21918033	+0.31196160
H	−5.73214429	−0.39591001	+2.61087033
H	−4.92192125	−1.79710028	+1.93254937
H	−5.32679620	−0.42677624	+0.90192435
H	−1.49618356	+1.21192470	+2.17949046

51

* E = +2.644 kcal/mol ; (29) 345_047_298_009_321_062_188_306_337

C	+0.00000000	+0.00000000	+0.00000000
C	+0.00000000	+0.00000000	+1.33768285
C	+1.20380295	+0.00000000	+2.16937353
C	+2.46239690	−0.39085723	+1.92806940
C	+3.09361882	−1.00522256	+0.75534667
C	+2.67770530	−2.12655477	+0.15243470
C	+1.48452794	−2.90643271	+0.57992262
H	+1.72209811	−3.97001007	+0.60251312
C	+3.40948524	−2.71314584	−1.01575845
C	+4.40063055	−1.76626971	−1.65709808
C	+5.24335935	−1.11436965	−0.58928524
C	+4.35667132	−0.30271785	+0.33119856

C	−1.22560873	+0.06751713	−0.85476081
C	−2.50799073	+0.36226545	−0.08318430
C	−2.49689467	−0.37558560	+1.23962687
C	−1.31432986	+0.14320882	+2.05290123
C	−3.63929103	−0.06203436	+2.20837097
C	−3.06308206	−0.49162308	+3.57108527
C	−1.52894722	−0.48797096	+3.42239655
O	+5.89911093	−2.15961861	+0.09389202
H	+6.34622370	−1.80661050	+0.85940576
C	−2.40776137	−1.87969823	+1.00162218
C	−4.98171927	−0.68125820	+1.88927579
H	+5.98442912	−0.45391888	−1.04728569
H	+4.10583638	+0.64182240	−0.15731554
H	+4.92238345	−0.01772771	+1.22329516
H	+3.87853224	−0.98510229	−2.20966150
H	+5.03769252	−2.29847639	−2.36022647
H	+3.93171262	−3.61006125	−0.67712917
H	+2.68094856	−3.04868474	−1.75579918
H	+0.67305341	−2.77752158	−0.13918851
H	+1.11364084	−2.60417266	+1.55371494
H	+3.16729580	−0.17512253	+2.72529374
H	+1.03965296	+0.41339726	+3.15731708
H	+0.94503387	−0.06440149	−0.52157680
H	−1.32408991	−0.88250570	−1.38906758
H	−1.07801009	+0.81472288	−1.63550252
H	−2.57985218	+1.43327192	+0.11578738
H	−3.37584837	+0.09009901	−0.68525406
H	−3.75732592	+1.02511815	+2.20964337
H	−3.42507935	−1.48558665	+3.83301987
H	−3.39678765	+0.17384352	+4.36411949
H	−1.12660864	−1.50020932	+3.44348594
H	−1.03883763	+0.06270905	+4.22129811
H	−2.54690448	−2.44385268	+1.92166334
H	−1.44231449	−2.16006392	+0.58919537
H	−3.18109497	−2.19788875	+0.30391029
H	−5.73557334	−0.36350800	+2.60710664
H	−4.93419037	−1.76835363	+1.92593338
H	−5.32967156	−0.39308738	+0.89823037
H	−1.48978987	+1.21842032	+2.18193470

51

* E = +2.719 kcal/mol ; (30) 017_313_063_010_321_062_060_302_336

C	+0.00000000	+0.00000000	+0.00000000
C	+0.00000000	+0.00000000	+1.33756816
C	+1.20579215	+0.00000000	+2.16739702
C	+2.45419307	−0.41467688	+1.92145026
C	+3.06171571	−1.03898444	+0.73913275
C	+2.65811441	−2.18608699	+0.17848035
C	+1.45988607	−2.95125636	+0.62225666
H	+1.70432583	−4.00669629	+0.74038768
C	+3.38697964	−2.79143012	−0.98580408
C	+4.78531034	−2.24142857	−1.17349797
C	+4.75305770	−0.73479451	−1.10071599
C	+4.28748551	−0.30186466	+0.26484762
C	−1.22664843	+0.06055741	−0.85396469
C	−2.50908548	+0.35479003	−0.08213735
C	−2.49632221	−0.38037851	+1.24215943
C	−1.31368937	+0.14164580	+2.05366920
C	−3.63796282	−0.06663363	+2.21145993
C	−3.05972024	−0.49234275	+3.57477685
C	−1.52575601	−0.48700637	+3.42460802
O	+6.01755718	−0.15214128	−1.30694055
H	+6.33811516	−0.39542772	−2.17261608
C	−2.40478202	−1.88459930	+1.00661988
C	−4.97974040	−0.68858067	+1.89490244
H	+4.04485380	−0.36207907	−1.84984120
H	+4.09161647	+0.76955265	+0.25664971
H	+5.11022596	−0.45286534	+0.96845426
H	+5.19782673	−2.57092844	−2.12812951
H	+5.44743771	−2.60964740	−0.38933091
H	+3.42638835	−3.87316697	−0.85528686
H	+2.79600332	−2.63147019	−1.89328704
H	+0.67338743	−2.89046938	−0.13309266
H	+1.05030857	−2.57826403	+1.55446156
H	+3.17585765	−0.20133306	+2.70406862
H	+1.05217868	+0.43031821	+3.14971819
H	+0.94525531	−0.05641336	−0.52220188
H	−1.32319604	−0.89210049	−1.38390332
H	−1.08212194	+0.80516781	−1.63766356
H	−2.58237297	+1.42610393	+0.11443155
H	−3.37677952	+0.08000736	−0.68323319

H −3.75759052 +1.02033868 +2.21051919
H −3.42031438 −1.48606339 +3.83951468
H −3.39348592 +0.17466653 +4.36645441
H −1.12196954 −1.49868281 +3.44787868
H −1.03531205 +0.06613597 +4.22156606
H −2.53933220 −2.44778498 +1.92799426
H −1.44022995 −2.16280596 +0.59073641
H −3.17986564 −2.20556714 +0.31209390
H −5.73350216 −0.37052357 +2.61266838
H −4.93034935 −1.77552152 +1.93377219
H −5.32893065 −0.40301092 +0.90354492
H −1.49062219 +1.21677040 +2.18094273
51
* E = +2.973 kcal/mol ; (31) 017_313_062_010_321_062_297_302_335
C +0.00000000 +0.00000000 +0.00000000
C +0.00000000 +0.00000000 +1.33758278
C +1.20607401 +0.00000000 +2.16689199
C +2.45329254 −0.41859131 +1.92149963
C +3.05753725 −1.04678357 +0.73953213
C +2.65341693 −2.19515218 +0.18174451
C +1.45634211 −2.95855421 +0.63167997
H +1.70111852 −4.01353616 +0.75272788
C +3.37546149 −2.80287715 −0.98549099
C +4.76882186 −2.24802310 −1.19696227
C +4.73783890 −0.74163825 −1.12126330
C +4.28051396 −0.31039632 +0.25366238
C −1.22659415 +0.06169542 −0.85379994
C −2.50859900 +0.35744983 −0.08185753
C −2.49666176 −0.37753256 +1.24257198
C −1.31333579 +0.14303576 +2.05397269
C −3.63777116 −0.06219523 +2.21197973
C −3.05991711 −0.48842368 +3.57533050
C −1.52596068 −0.48544556 +3.42488399
O +5.96691215 −0.15301702 −1.46879511
H +6.63158996 −0.43576745 −0.84287608
C −2.40716927 −1.88190778 +1.00721287
C −4.98035017 −0.68251800 +1.89568221
H +4.03218031 −0.36856437 −1.86374931
H +4.08873132 +0.76186788 +0.25068078
H +5.09941141 −0.46592976 +0.96554265

H	+5.17325743	−2.56200619	−2.15699553
H	+5.43744259	−2.62689505	−0.41955993
H	+3.41973265	−3.88387855	−0.84996340
H	+2.77476524	−2.65022392	−1.88757705
H	+0.66877714	−2.90057027	−0.12259300
H	+1.04863186	−2.58203293	+1.56325501
H	+3.17553135	−0.20548154	+2.70386789
H	+1.05378376	+0.43310647	+3.14821800
H	+0.94500137	−0.05622611	−0.52262922
H	−1.32387855	−0.89088792	−1.38363500
H	−1.08101849	+0.80588571	−1.63761464
H	−2.58071701	+1.42888149	+0.11443648
H	−3.37656347	+0.08352860	−0.68288284
H	−3.75604733	+1.02491701	+2.21087939
H	−3.42191977	−1.48152722	+3.84041988
H	−3.39248430	+0.17931569	+4.36688602
H	−1.12383937	−1.49781388	+3.44787114
H	−1.03452938	+0.06683208	+4.22187472
H	−2.54258862	−2.44490940	+1.92857760
H	−1.44305661	−2.16150601	+0.59126572
H	−3.18262210	−2.20181852	+0.31265643
H	−5.73359552	−0.36342836	+2.61351762
H	−4.93232900	−1.76951326	+1.93464112
H	−5.32931922	−0.39658731	+0.90437095
H	−1.48899241	+1.21836037	+2.18130464

51

* E = +3.073 kcal/mol ; (32) 344_048_299_009_321_062_059_305_336

C	+0.00000000	+0.00000000	+0.00000000
C	+0.00000000	+0.00000000	+1.33759523
C	+1.20396793	+0.00000000	+2.16961865
C	+2.45933524	−0.40103084	+1.93036716
C	+3.08365976	−1.02680925	+0.75950261
C	+2.66135391	−2.15364323	+0.17341432
C	+1.46919605	−2.92781289	+0.61440843
H	+1.70776354	−3.99074287	+0.65838139
C	+3.38963804	−2.75605757	−0.98881095
C	+4.36245525	−1.80673356	−1.65347088
C	+5.21817951	−1.12560778	−0.60771995
C	+4.34524889	−0.32604539	+0.32825142
C	−1.22565489	+0.06835924	−0.85485694

C	−2.50705088	+0.36738772	−0.08322180
C	−2.49812276	−0.37060163	+1.23951509
C	−1.31408257	+0.14448394	+2.05292573
C	−3.63946952	−0.05439078	+2.20863179
C	−3.06403160	−0.48635892	+3.57100021
C	−1.52993540	−0.48777659	+3.42168454
O	+5.91324868	−2.06991335	+0.17827808
H	+6.47098585	−2.59974194	−0.38728194
C	−2.41344048	−1.87487682	+1.00094888
C	−4.98367135	−0.66978336	+1.88953024
H	+5.93328542	−0.45168986	−1.08647948
H	+4.08844681	+0.62409247	−0.14440797
H	+4.93484707	−0.07353992	+1.21012487
H	+3.82145651	−1.04029227	−2.20964312
H	+4.98584352	−2.33835122	−2.37393016
H	+3.91915679	−3.64443991	−0.63625399
H	+2.66092200	−3.11401490	−1.71799868
H	+0.65720117	−2.81399253	−0.10672632
H	+1.09857166	−2.60807131	+1.58261033
H	+3.16847290	−0.18840361	+2.72401940
H	+1.04232166	+0.42118611	+3.15469040
H	+0.94523896	−0.06388129	−0.52125166
H	−1.32688835	−0.88225250	−1.38765902
H	−1.07688278	+0.81423475	−1.63667758
H	−2.57513945	+1.43863683	+0.11578777
H	−3.37595740	+0.09817163	−0.68517429
H	−3.75457433	+1.03306793	+2.21051106
H	−3.42934901	−1.47913554	+3.83288855
H	−3.39510817	+0.18006586	+4.36434019
H	−1.13093534	−1.50136884	+3.44109794
H	−1.03736346	+0.06004220	+4.22096763
H	−2.55466758	−2.43903691	+1.92066142
H	−1.44856218	−2.15762711	+0.58895875
H	−3.18742812	−2.19040345	+0.30269774
H	−5.73649436	−0.35034361	+2.60770739
H	−4.93910409	−1.75703168	+1.92565896
H	−5.33106784	−0.38017545	+0.89868841
H	−1.48701679	+1.21996075	+2.18341503

51

* E = +3.134 kcal/mol ; (33) 344_047_298_007_322_062_060_224_039

C	+0.00000000	+0.00000000	+0.00000000
C	+0.00000000	+0.00000000	+1.33690827
C	+1.23409123	+0.00000000	+2.12174500
C	+2.37663456	+0.65148319	+1.88014464
C	+2.65339317	+1.67835688	+0.86024363
C	+3.77312038	+1.64969889	+0.12679588
C	+4.79258176	+0.56286542	+0.21610609
H	+5.77754059	+0.98077309	+0.42798807
C	+4.11211351	+2.73121423	−0.85165907
C	+2.93628343	+3.61801725	−1.19970780
C	+2.21269710	+4.03572901	+0.06195678
C	+1.67407904	+2.81991407	+0.77500003
C	−1.22747641	−0.02780326	−0.85404525
C	−2.53018082	+0.12426127	−0.07270610
C	−2.44244342	−0.62916690	+1.23905598
C	−1.31461123	−0.00676621	+2.05827126
C	−3.60632402	−0.44057311	+2.21462403
C	−2.98554565	−0.81742084	+3.57422437
C	−1.45920150	−0.66993548	+3.42026493
O	+3.08524735	+4.69672308	+0.95443488
H	+3.43191465	+5.47941546	+0.53157434
C	−2.20354057	−2.11251847	+0.97773962
C	−4.88054260	−1.19036130	+1.89586969
H	+1.37701005	+4.69576263	−0.18520982
H	+0.76075129	+2.49519861	+0.27861424
H	+1.38477188	+3.11271312	+1.78324545
H	+2.23477722	+3.08005896	−1.83821893
H	+3.26865825	+4.49231630	−1.76141014
H	+4.91733265	+3.33468918	−0.42501794
H	+4.52284715	+2.28259975	−1.75725431
H	+4.87523541	+0.03966251	−0.73729630
H	+4.56135488	−0.17631729	+0.97492257
H	+3.19486624	+0.43996145	+2.55762790
H	+1.21462231	−0.63169368	+3.00172521
H	+0.95258490	−0.00862095	−0.51513878
H	−1.23562020	−0.96802209	−1.41246684
H	−1.15609612	+0.75042187	−1.61606907
H	−2.70916432	+1.17924548	+0.14431866
H	−3.36870966	−0.22348279	−0.67738637
H	−3.83396007	+0.62897010	+2.22356642

H −3.25146941 −1.84154991 +3.83514214
H −3.37671584 −0.18767269 +4.37011250
H −0.97216364 −1.64459701 +3.42814584
H −1.01092577 −0.08380909 +4.21866972
H −2.27835033 −2.70099326 +1.89016154
H −1.21802932 −2.28693024 +0.55323599
H −2.94624795 −2.49761316 +0.28050486
H −5.65987823 −0.95526023 +2.61830720
H −4.72290921 −2.26723496 +1.92481620
H −5.25956655 −0.93250876 +0.90786017
H −1.59510725 +1.04364997 +2.20397887
51
* E = +3.143 kcal/mol ; (34) 013_314_064_007_321_063_183_130_131
C +0.00000000 +0.00000000 +0.00000000
C +0.00000000 +0.00000000 +1.33589226
C +1.26403340 +0.00000000 +2.09179326
C +1.58481514 +0.81325833 +3.09943278
C +0.81978813 +1.99404109 +3.54663366
C +0.48643391 +2.18729201 +4.82872667
C +0.81359997 +1.21226588 +5.91064270
H −0.09877254 +0.83907953 +6.37767836
C −0.26085054 +3.40434128 +5.28543837
C −0.85455056 +4.20644024 +4.14712608
C +0.17321944 +4.37560449 +3.06217787
C +0.53103325 +3.02437742 +2.48509987
C −1.22589739 +0.00628775 −0.85765704
C −2.52173888 +0.17874519 −0.07070348
C −2.44307692 −0.60433227 +1.22348851
C −1.31302197 −0.00918900 +2.06390509
C −3.60498463 −0.43640336 +2.20531588
C −2.98315346 −0.86406010 +3.55042791
C −1.45567650 −0.73204351 +3.39440443
O −0.36246072 +5.23499900 +2.08439471
H +0.25522838 +5.31134156 +1.36026756
C −2.22074922 −2.08427579 +0.92493338
C −4.88620881 −1.16407168 +1.86393255
H +1.06995774 +4.82685048 +3.50197717
H +1.40749121 +3.10939113 +1.83975795
H −0.28512173 +2.70254350 +1.83636261
H −1.19483215 +5.18140499 +4.48890295

H	−1.71735779	+3.68869329	+3.72621048
H	−1.04616498	+3.09829929	+5.97792368
H	+0.41613642	+4.03034387	+5.87436318
H	+1.39037822	+1.69515846	+6.70033750
H	+1.37220115	+0.35550778	+5.54838843
H	+2.53629367	+0.64187412	+3.58872245
H	+1.99062695	−0.75014518	+1.80466847
H	+0.95061984	−0.00734607	−0.52002257
H	−1.25508025	−0.92875832	−1.42397034
H	−1.13552544	+0.79127359	−1.60941289
H	−2.67243679	+1.23366232	+0.16641988
H	−3.37046958	−0.13692626	−0.67865494
H	−3.82367966	+0.63399548	+2.24929495
H	−3.26446202	−1.89118109	+3.78238376
H	−3.36236365	−0.25198218	+4.36597179
H	−0.97671328	−1.70915219	+3.35096230
H	−0.99112842	−0.18723179	+4.21028474
H	−2.31770810	−2.69618802	+1.81926509
H	−1.23304806	−2.26449497	+0.50818281
H	−2.95999648	−2.43755424	+0.20734605
H	−5.66175019	−0.94613666	+2.59579586
H	−4.73859809	−2.24274731	+1.85640931
H	−5.26520978	−0.86955837	+0.88618908
H	−1.60125852	+1.03098299	+2.25526032

51

* E = +3.330 kcal/mol ; (35) 014_313_064_007_321_063_061_129_131

C	+0.00000000	+0.00000000	+0.00000000
C	+0.00000000	+0.00000000	+1.33577337
C	+1.26511841	+0.00000000	+2.08944420
C	+1.58906566	+0.81227110	+3.09666616
C	+0.82338812	+1.99034329	+3.55149460
C	+0.48791405	+2.17016371	+4.83479512
C	+0.81648701	+1.18834004	+5.91018695
H	−0.09456923	+0.81107154	+6.37669562
C	−0.26689873	+3.38021426	+5.29765235
C	−0.88356754	+4.16238917	+4.15719048
C	+0.14197142	+4.36033980	+3.06737809
C	+0.54348117	+3.02549934	+2.49389904
C	−1.22551614	+0.01590372	−0.85776418
C	−2.51995149	+0.19682608	−0.07051958

C −2.44673353 −0.58844481 +1.22261653
C −1.31264652 −0.00223347 +2.06390780
C −3.60744533 −0.41433925 +2.20471169
C −2.98848765 −0.84871005 +3.54900350
C −1.46011649 −0.72704660 +3.39278777
O −0.34517244 +5.13600126 +1.99802874
H −0.57738887 +6.00407490 +2.32014580
C −2.23455969 −2.06950650 +0.92189678
C −4.89369311 −1.13253073 +1.86204363
H +1.02613015 +4.84014370 +3.50258567
H +1.42852278 +3.14595978 +1.87098781
H −0.24622062 +2.69508890 +1.81789161
H −1.25716112 +5.12359058 +4.51266372
H −1.73170771 +3.61934565 +3.73814918
H −1.04025531 +3.06915300 +6.00125546
H +0.40814224 +4.02097543 +5.87303145
H +1.39527552 +1.66544320 +6.70211108
H +1.37494996 +0.33465752 +5.54053539
H +2.54442756 +0.64367978 +3.57926247
H +1.99304056 −0.74689241 +1.79724385
H +0.95053300 −0.00910387 −0.51991553
H −1.26146642 −0.91769582 −1.42612545
H −1.12866764 +0.80239072 −1.60695398
H −2.66201336 +1.25265451 +0.16758059
H −3.37118281 −0.11190779 −0.67863536
H −3.81870866 +0.65753062 +2.25039258
H −3.27704314 −1.87426715 +3.77910174
H −3.36335082 −0.23560191 +4.36590338
H −0.98817248 −1.70744884 +3.34682439
H −0.99144064 −0.18756614 +4.20977333
H −2.33635088 −2.68244328 +1.81503300
H −1.24778015 −2.25545908 +0.50552778
H −2.97563667 −2.41636403 +0.20302887
H −5.66790136 −0.91084971 +2.59427663
H −4.75333730 −2.21216091 +1.85241982
H −5.27045112 −0.83366921 +0.88474595
H −1.59361544 +1.03973659 +2.25695281
51
* E = +3.561 kcal/mol ; (36) 014_313_063_007_321_063_294_129_131
C +0.00000000 +0.00000000 +0.00000000

C	+0.00000000	+0.00000000	+1.33587299
C	+1.26423469	+0.00000000	+2.09163847
C	+1.58644239	+0.81546956	+3.09676281
C	+0.82080091	+1.99690489	+3.54251240
C	+0.47582556	+2.18473537	+4.82220896
C	+0.79705946	+1.20784131	+5.90429117
H	−0.11691193	+0.83752898	+6.37045841
C	−0.27795094	+3.39904905	+5.27551790
C	−0.87456190	+4.19242950	+4.13158668
C	+0.16065358	+4.37827078	+3.04888760
C	+0.55248418	+3.03251664	+2.48100438
C	−1.22566536	+0.00791174	−0.85801475
C	−2.52132318	+0.18363016	−0.07157495
C	−2.44493626	−0.59843046	+1.22324963
C	−1.31315996	−0.00657489	+2.06357974
C	−3.60664579	−0.42799354	+2.20480777
C	−2.98583287	−0.85722243	+3.54991467
C	−1.45803115	−0.72941859	+3.39371482
O	−0.25915563	+5.26467115	+2.04048076
H	−0.99992369	+4.87575653	+1.57851623
C	−2.22615013	−2.07899048	+0.92478694
C	−4.88970451	−1.15225791	+1.86310002
H	+1.03968763	+4.85202992	+3.48624263
H	+1.43723507	+3.14166911	+1.85547805
H	−0.23739721	+2.68758239	+1.80722620
H	−1.23304321	+5.16169140	+4.47165606
H	−1.72981382	+3.65300368	+3.71542609
H	−1.06051761	+3.09176925	+5.97064394
H	+0.39457210	+4.03454055	+5.85923188
H	+1.37485150	+1.68826931	+6.69478078
H	+1.35371590	+0.34978450	+5.54203748
H	+2.53989674	+0.64735012	+3.58326138
H	+1.99074455	−0.74989558	+1.80393291
H	+0.95045970	−0.00732159	−0.51997832
H	−1.25736885	−0.92765430	−1.42317168
H	−1.13301878	+0.79143937	−1.61096455
H	−2.66994843	+1.23929442	+0.16529581
H	−3.37107485	−0.12946753	−0.67937335
H	−3.82298036	+0.64314470	+2.24904883
H	−3.27007611	−1.88348951	+3.78178379

H	−3.36340065	−0.24438448	+4.36567118
H	−0.98173332	−1.70770714	+3.34949646
H	−0.99155914	−0.18638503	+4.20949193
H	−2.32564138	−2.69058484	+1.81898142
H	−1.23843779	−2.26154129	+0.50923210
H	−2.96550483	−2.43034988	+0.20641828
H	−5.66473857	−0.93291115	+2.59505394
H	−4.74454826	−2.23119795	+1.85526417
H	−5.26800701	−0.85679582	+0.88536452
H	−1.59828154	+1.03479327	+2.25649435

51

* E = +3.629 kcal/mol ; (37) 008_321_063_004_324_062_183_158_147

C	+0.00000000	+0.00000000	+0.00000000
C	+0.00000000	+0.00000000	+1.33974497
C	+1.28941467	+0.00000000	+2.04042453
C	+1.68929215	+0.53104048	+3.20506481
C	+1.03973937	+1.48745050	+4.10204275
C	+1.38540936	+1.57633065	+5.39883224
C	+2.32703014	+0.64212922	+6.08484877
H	+1.82373064	+0.16177073	+6.92441048
C	+0.86757706	+2.65649841	+6.30215103
C	−0.22538480	+3.51760698	+5.70324423
C	+0.07136050	+3.79107884	+4.25462523
C	+0.07798374	+2.47943674	+3.50281101
C	−1.20963893	−0.10989762	−0.86962420
C	−2.52700612	−0.13136420	−0.10027140
C	−2.35619898	−0.87905027	+1.20620617
C	−1.32237293	−0.12012465	+2.04388917
C	−3.54746114	−0.83288620	+2.16646756
C	−2.90730632	−1.13969162	+3.53263125
C	−1.41276775	−0.78942605	+3.40689213
O	−0.92154383	+4.66311487	+3.77103677
H	−0.77602459	+4.81931926	+2.84039310
C	−1.94456332	−2.32253332	+0.94181369
C	−4.72143507	−1.72370311	+1.82644662
H	+1.05743481	+4.26158505	+4.17291155
H	+0.33281422	+2.63728373	+2.45398550
H	−0.94333879	+2.10110022	+3.52455216
H	−0.32399840	+4.45430955	+6.24726490
H	−1.18901387	+3.01069447	+5.75765791

H	+0.52263699	+2.20816196	+7.23516930
H	+1.71968994	+3.28174169	+6.58609217
H	+3.17286482	+1.18855979	+6.50411695
H	+2.70711144	−0.14434234	+5.44474419
H	+2.70465595	+0.28101581	+3.47889874
H	+2.05507288	−0.55523343	+1.51213715
H	+0.95215921	+0.04660169	−0.51542961
H	−1.11044188	−1.02320792	−1.46328353
H	−1.21199008	+0.70107799	−1.59933305
H	−2.84224357	+0.88939589	+0.12388127
H	−3.30865414	−0.57751898	−0.71619630
H	−3.89824812	+0.20291692	+2.17471611
H	−3.04013528	−2.19324022	+3.77830376
H	−3.39356671	−0.57726845	+4.32671259
H	−0.79272159	−1.68427225	+3.42720698
H	−1.06057847	−0.15159284	+4.21274978
H	−1.93609807	−2.91274203	+1.85615054
H	−0.95186223	−2.38199399	+0.50304614
H	−2.64564852	−2.79412724	+0.25478906
H	−5.53391050	−1.57979592	+2.53615962
H	−4.44213884	−2.77547585	+1.85955376
H	−5.11209068	−1.51028570	+0.83233195
H	−1.74190987	+0.88363312	+2.15089292

51

* E = +3.791 kcal/mol ; (38) 345_044_298_011_321_061_293_065_259

C	+0.00000000	+0.00000000	+0.00000000
C	+0.00000000	+0.00000000	+1.33249000
C	+1.24820803	+0.00000000	+2.12629583
C	+1.82675731	−1.04089423	+2.72397157
C	+1.40447681	−2.45154870	+2.73566059
C	+1.35521783	−3.24097576	+1.65275418
C	+1.71204861	−2.79101161	+0.27638630
H	+0.82934516	−2.74583242	−0.36374869
C	+0.96389723	−4.68985530	+1.74190635
C	+0.98249158	−5.25319991	+3.14893674
C	+0.33820891	−4.27224217	+4.10362394
C	+1.13960989	−2.98811514	+4.12191051
C	−1.22763558	+0.02366289	−0.85674890
C	−2.51083300	+0.32098280	−0.08907779
C	−2.49082554	−0.37532155	+1.25609884

C	−1.30832763	+0.16959274	+2.05285269
C	−3.63371265	−0.02916209	+2.21374512
C	−3.05631226	−0.39705908	+3.59423658
C	−1.52327431	−0.40422824	+3.44265838
O	−1.00266058	−4.02786321	+3.74303420
H	−1.00160808	−3.56079089	+2.90617426
C	−2.39240862	−1.88764232	+1.07459320
C	−4.97273803	−0.66736779	+1.91792080
H	+0.29181068	−4.69089059	+5.10608715
H	+2.09101547	−3.15395754	+4.63361508
H	+0.60302005	−2.24361468	+4.70842100
H	+2.01142111	−5.42678054	+3.46473989
H	+0.46107849	−6.20726728	+3.18347182
H	−0.03085033	−4.81270403	+1.30078768
H	+1.62577747	−5.27124218	+1.10006506
H	+2.39856549	−3.50447487	−0.17913884
H	+2.17605738	−1.81209522	+0.27549568
H	+2.69125135	−0.82788652	+3.34576762
H	+1.70515138	+0.97106284	+2.28416380
H	+0.94474682	−0.05847454	−0.52446272
H	−1.31048637	−0.94695104	−1.35543722
H	−1.09610741	+0.74693010	−1.66199806
H	−2.59716659	+1.39611864	+0.07917007
H	−3.37689012	+0.02022487	−0.68003066
H	−3.75684362	+1.05651859	+2.16818953
H	−3.41492539	−1.37819597	+3.90438385
H	−3.38911573	+0.30540571	+4.35514730
H	−1.13614126	−1.41820700	+3.50327604
H	−1.01670044	+0.17154592	+4.21314748
H	−2.61336375	−2.41153534	+2.00331654
H	−1.39695422	−2.18271517	+0.74202140
H	−3.11415896	−2.23286627	+0.33575410
H	−5.72654555	−0.33166077	+2.62747834
H	−4.91829473	−1.75235181	+1.99048606
H	−5.32547840	−0.41365486	+0.91914124
H	−1.47730261	+1.25010680	+2.13817573

51

* E = +3.961 kcal/mol ; (39) 010_320_063_004_324_062_060_158_148

C	+0.00000000	+0.00000000	+0.00000000
C	+0.00000000	+0.00000000	+1.33964729

C	+1.29020389	+0.00000000	+2.03764003
C	+1.69315081	+0.52318329	+3.20456281
C	+1.04629296	+1.47015914	+4.11450004
C	+1.39388270	+1.53555525	+5.41189011
C	+2.33345255	+0.58853785	+6.08313575
H	+1.82915302	+0.09441931	+6.91421077
C	+0.87831131	+2.60166968	+6.33287253
C	−0.23429403	+3.44896243	+5.75011204
C	+0.05576932	+3.75989459	+4.30214250
C	+0.08897579	+2.47086789	+3.52359775
C	−1.21015078	−0.10292888	−0.86967608
C	−2.52730173	−0.11972586	−0.09995467
C	−2.35848388	−0.87040262	+1.20495945
C	−1.32209466	−0.11633369	+2.04399627
C	−3.54965488	−0.82205591	+2.16505763
C	−2.91069766	−1.13406663	+3.53048321
C	−1.41452589	−0.78962207	+3.40498165
O	−0.94045150	+4.56564670	+3.71878846
H	−0.99512004	+5.39121166	+4.19496490
C	−1.95228613	−2.31503572	+0.93801014
C	−4.72647964	−1.70829690	+1.82287464
H	+1.03121586	+4.25275968	+4.22450351
H	+0.35186495	+2.66695150	+2.48615328
H	−0.92966068	+2.08556158	+3.51930755
H	−0.35061123	+4.36643029	+6.32866035
H	−1.18599648	+2.91883786	+5.79473999
H	+0.55030455	+2.14253645	+7.26674776
H	+1.72474353	+3.23767713	+6.61030927
H	+3.18070276	+1.12567427	+6.51181322
H	+2.71211909	−0.18746666	+5.42964804
H	+2.71102990	+0.27505323	+3.47075441
H	+2.05672659	−0.54742127	+1.50238742
H	+0.95218008	+0.04536244	−0.51543289
H	−1.11498940	−1.01525142	−1.46563280
H	−1.20845003	+0.71044209	−1.59660792
H	−2.83713695	+0.90210064	+0.12582434
H	−3.31099271	−0.56189982	−0.71626546
H	−3.89662913	+0.21490461	+2.17530155
H	−3.04782193	−2.18752351	+3.77449437
H	−3.39447550	−0.57088727	+4.32566381

H	−0.79855103	−1.68740254	+3.42194956
H	−1.05929995	−0.15640198	+4.21313763
H	−1.94770137	−2.90746875	+1.85100660
H	−0.95915005	−2.37738637	+0.50066617
H	−2.65424238	−2.78220699	+0.24878526
H	−5.53882243	−1.56343449	+2.53262489
H	−4.45059846	−2.76108375	+1.85343346
H	−5.11595675	−1.49121731	+0.82909984
H	−1.73739504	+0.88887455	+2.15241484

51

* E = +4.289 kcal/mol ; (40) 009_320_062_004_324_062_298_158_148

C	+0.00000000	+0.00000000	+0.00000000
C	+0.00000000	+0.00000000	+1.33960653
C	+1.28955328	+0.00000000	+2.03903296
C	+1.69061733	+0.52535523	+3.20554911
C	+1.04223990	+1.47505079	+4.11152729
C	+1.38444536	+1.54366073	+5.41024247
C	+2.31856294	+0.59401475	+6.08538595
H	+1.81432307	+0.11353937	+6.92433885
C	+0.87317408	+2.61536985	+6.32706068
C	−0.21973206	+3.48464792	+5.73817816
C	+0.07411816	+3.78270366	+4.28845035
C	+0.09428941	+2.48323806	+3.51602666
C	−1.20935129	−0.10643493	−0.87013180
C	−2.52700166	−0.12796827	−0.10136835
C	−2.35691082	−0.87647000	+1.20469211
C	−1.32264309	−0.11879246	+2.04305078
C	−3.54874930	−0.83055526	+2.16416530
C	−2.90953499	−1.13865504	+3.53044588
C	−1.41468577	−0.78860923	+3.40570849
O	−0.83616210	+4.70160888	+3.73596553
H	−1.71398394	+4.32537475	+3.77355830
C	−1.94623604	−2.32016123	+0.93972818
C	−4.72313561	−1.72029934	+1.82278605
H	+1.04708833	+4.26703034	+4.20838835
H	+0.35546114	+2.66939856	+2.47621289
H	−0.92514958	+2.09116242	+3.51551941
H	−0.32125723	+4.41198295	+6.29805380
H	−1.17992984	+2.96527454	+5.79482714
H	+0.53267074	+2.15949415	+7.25814013

H	+1.72572288	+3.23952761	+6.61177152
H	+3.17329887	+1.12723743	+6.50376197
H	+2.68567764	−0.19193355	+5.43715418
H	+2.70790550	+0.27760054	+3.47448741
H	+2.05629302	−0.54881108	+1.50568746
H	+0.95212967	+0.04699236	−0.51520752
H	−1.11097783	−1.01820117	−1.46626549
H	−1.21007072	+0.70688250	−1.59705286
H	−2.84140812	+0.89303495	+0.12279324
H	−3.30862420	−0.57348619	−0.71775911
H	−3.89921578	+0.20561686	+2.17205622
H	−3.04270525	−2.19240143	+3.77485210
H	−3.39663586	−0.57802723	+4.32555910
H	−0.79517542	−1.68374876	+3.42561398
H	−1.06171791	−0.15242282	+4.21264832
H	−1.93827601	−2.91119921	+1.85356430
H	−0.95353984	−2.37966310	+0.50115751
H	−2.64741563	−2.79071925	+0.25213738
H	−5.53624614	−1.57672744	+2.53188392
H	−4.44430583	−2.77215979	+1.85534026
H	−5.11261397	−1.50602276	+0.82845512
H	−1.74108607	+0.88588702	+2.14861093

51

* E = +4.299 kcal/mol ; (41) 047_333_335_007_321_064_294_310_141

C	+0.00000000	+0.00000000	+0.00000000
C	+0.00000000	+0.00000000	+1.33840539
C	+1.27863842	+0.00000000	+2.05736598
C	+1.60638163	+0.66262952	+3.17316293
C	+0.81764803	+1.65061499	+3.90359708
C	+0.21569946	+2.72105230	+3.35661356
C	+0.28419493	+3.11193336	+1.92083464
H	+0.46506929	+4.18328374	+1.83799934
C	−0.59464587	+3.58875673	+4.27689768
C	+0.12290057	+3.94524165	+5.58541211
C	+1.14777216	+2.89686697	+6.00243334
C	+0.78298549	+1.54505024	+5.40633225
C	−1.21913144	+0.00965554	−0.86448745
C	−2.51662407	+0.17920381	−0.08194106
C	−2.43937093	−0.61153533	+1.20722598
C	−1.31672658	−0.01491559	+2.05968683

C	−3.60929682	−0.45530122	+2.18106608
C	−2.99657933	−0.89472447	+3.52537681
C	−1.46917842	−0.75052926	+3.38235358
O	+2.45101348	+3.26290995	+5.61096136
H	+2.47450830	+3.25816777	+4.65304329
C	−2.21173442	−2.08932200	+0.90070874
C	−4.88637471	−1.18222189	+1.82306766
H	+1.19303219	+2.82906816	+7.08607138
H	−0.21217115	+1.25431319	+5.75456242
H	+1.47942199	+0.78540306	+5.75344127
H	−0.61786590	+4.07558689	+6.37243484
H	+0.64869722	+4.89250687	+5.48930633
H	−0.91359749	+4.49337352	+3.76427784
H	−1.51044571	+3.04329081	+4.52108691
H	−0.65651230	+2.91143143	+1.40301766
H	+1.06499211	+2.57838781	+1.38994602
H	+2.57960436	+0.44849069	+3.60184774
H	+2.04919340	−0.62315228	+1.61988975
H	+0.95257064	−0.00943784	−0.51682229
H	−1.24623942	−0.92408051	−1.43319834
H	−1.12343499	+0.79625354	−1.61394452
H	−2.66627751	+1.23328772	+0.16196746
H	−3.36470131	−0.13143312	−0.69322476
H	−3.83060565	+0.61439739	+2.23349879
H	−3.27251410	−1.92645860	+3.74238876
H	−3.38658561	−0.29607395	+4.34585776
H	−0.98151484	−1.72292604	+3.33469041
H	−1.01947450	−0.21457275	+4.21120020
H	−2.31329673	−2.70659197	+1.79078099
H	−1.22150042	−2.26684824	+0.48955617
H	−2.94624909	−2.43972710	+0.17687963
H	−5.66815438	−0.97149871	+2.55038213
H	−4.73725287	−2.26053644	+1.80830365
H	−5.25814863	−0.88068889	+0.84470586
H	−1.61022410	+1.02248930	+2.25157932

51

* E = +4.690 kcal/mol ; (42) 337_049_300_010_323_061_059_062_254

C	+0.00000000	+0.00000000	+0.00000000
C	+0.00000000	+0.00000000	+1.33272706
C	+1.25685884	+0.00000000	+2.11438610

C	+1.78131140	−1.02302580	+2.78996014
C	+1.25707209	−2.39303113	+2.90043535
C	+1.09196886	−3.22534599	+1.86482599
C	+1.47967913	−2.90390394	+0.46101023
H	+0.60779298	−2.84686081	−0.19228119
C	+0.45590461	−4.57223130	+2.04283311
C	+0.54068376	−5.09371759	+3.46125686
C	+0.08314970	−4.03378457	+4.43762865
C	+0.94973721	−2.80360849	+4.31815857
C	−1.22702718	+0.00600558	−0.85788416
C	−2.52349944	+0.24844402	−0.09001179
C	−2.47970492	−0.43679590	+1.26126026
C	−1.31245618	+0.15178405	+2.05062855
C	−3.63192920	−0.10996106	+2.21503174
C	−3.04605600	−0.43724349	+3.60079525
C	−1.51471020	−0.40279371	+3.44963961
O	−1.24580482	−3.63433911	+4.16632143
H	−1.81833176	−4.39675649	+4.21982384
C	−2.33262107	−1.94468673	+1.09310668
C	−4.95265286	−0.79082000	+1.93234049
H	+0.14518616	−4.41185617	+5.46122674
H	+1.89189971	−2.98209543	+4.84265617
H	+0.46147036	−1.98649285	+4.84723070
H	+1.57007373	−5.36340726	+3.69974656
H	−0.05711322	−5.99961618	+3.57433559
H	−0.59160266	−4.49466715	+1.73850955
H	+0.91647011	−5.28432708	+1.35842886
H	+2.11548589	−3.69840629	+0.06875101
H	+2.01363021	−1.96421205	+0.38905073
H	+2.66695071	−0.81846476	+3.38414890
H	+1.77890607	+0.94868884	+2.17405117
H	+0.94613854	−0.05715569	−0.52397782
H	−1.27792719	−0.95534104	−1.37800452
H	−1.11664333	+0.75002800	−1.64774788
H	−2.65800253	+1.31975132	+0.07211177
H	−3.37409650	−0.09221186	−0.68215280
H	−3.78698097	+0.97106545	+2.15170320
H	−3.36877523	−1.42789691	+3.92145039
H	−3.40487977	+0.26350068	+4.35189406
H	−1.11583457	−1.40877966	+3.51967914

H	−1.02453262	+0.20003600	+4.21053892
H	−2.35380346	−2.46411151	+2.05007398
H	−1.38732768	−2.19225019	+0.61701501
H	−3.13876733	−2.33770285	+0.47467355
H	−5.71965741	−0.45074844	+2.62586065
H	−4.86970164	−1.87087859	+2.04197793
H	−5.30517211	−0.58072942	+0.92314883
H	−1.50666753	+1.22988212	+2.11300665

51

* E = +4.749 kcal/mol ; (43) 337_049_300_010_323_061_181_061_253

C	+0.00000000	+0.00000000	+0.00000000
C	+0.00000000	+0.00000000	+1.33272075
C	+1.25699429	+0.00000000	+2.11466404
C	+1.77373573	−1.01582798	+2.80767669
C	+1.24076849	−2.37993292	+2.94331432
C	+1.05597245	−3.22696327	+1.92242686
C	+1.44218656	−2.93202513	+0.51224029
H	+0.57035108	−2.88883884	−0.14178053
C	+0.39457351	−4.55808615	+2.12449950
C	+0.46952816	−5.06005589	+3.55064498
C	+0.03210220	−3.97857444	+4.50515956
C	+0.93201631	−2.76739948	+4.36742686
C	−1.22657990	+0.01312694	−0.85851785
C	−2.52242305	+0.26186379	−0.09134178
C	−2.48253236	−0.42860733	+1.25735994
C	−1.31342460	+0.15516751	+2.04762428
C	−3.63337915	−0.10231967	+2.21253055
C	−3.04803372	−0.44523503	+3.59508853
C	−1.51603376	−0.40773464	+3.44307831
O	−1.30961122	−3.67797976	+4.18045380
H	−1.62661632	−2.98702704	+4.75832077
C	−2.34196666	−1.93638485	+1.08166664
C	−4.95758569	−0.77441917	+1.92620056
H	+0.08355872	−4.34778857	+5.53274867
H	+1.87205536	−2.96473030	+4.89061536
H	+0.48425121	−1.92041836	+4.89167391
H	+1.49353652	−5.34270027	+3.79476510
H	−0.15694359	−5.93993122	+3.68113061
H	−0.65286354	−4.46731077	+1.82642253
H	+0.84092784	−5.28933926	+1.45095265

H +2.07614244 −3.73575372 +0.13635505
H +1.97795514 −1.99516538 +0.42015752
H +2.66336641 −0.80717905 +3.39459937
H +1.79015087 +0.94352328 +2.15370122
H +0.94625886 −0.06117632 −0.52332685
H −1.28236071 −0.94718318 −1.37961474
H −1.11152094 +0.75755076 −1.64722451
H −2.65087500 +1.33353696 +0.07333949
H −3.37433206 −0.07274399 −0.68473658
H −3.78206310 +0.98008114 +2.16067231
H −3.38167120 −1.43889224 +3.89837045
H −3.40594946 +0.24637862 +4.35489800
H −1.10375568 −1.41053434 +3.48683857
H −1.02325081 +0.18748569 +4.20837662
H −2.37603539 −2.46877527 +2.03117579
H −1.39444065 −2.18550509 +0.61186518
H −3.14439144 −2.32038539 +0.45323671
H −5.72243399 −0.43671228 +2.62318846
H −4.87970959 −1.85576287 +2.02499843
H −5.30944455 −0.55256470 +0.91954770
H −1.50478662 +1.23315463 +2.11671725
51
* E = +4.877 kcal/mol ; (44) 313_034_016_010_321_062_176_147_320
C +0.00000000 +0.00000000 +0.00000000
C +0.00000000 +0.00000000 +1.33745096
C +1.23259833 +0.00000000 +2.12308914
C +2.37076185 −0.66674690 +1.89180348
C +2.61843082 −1.70770278 +0.89482309
C +3.80028315 −1.87366017 +0.28182525
C +4.98056214 −0.97708929 +0.42921973
H +5.25719733 −0.55751652 −0.53887563
C +3.92205867 −3.00645134 −0.69370519
C +3.36106757 −4.32251167 −0.15774616
C +2.15791277 −4.12489889 +0.75242826
C +1.56242301 −2.73504883 +0.59641318
C −1.22754010 +0.05513309 −0.85287292
C −2.50808301 +0.35024340 −0.07532737
C −2.49003946 −0.37701992 +1.25405036
C −1.30583954 +0.15191408 +2.05947797
C −3.62738078 −0.06103397 +2.22849009

C	−3.04087161	−0.47524586	+3.59241701
C	−1.50777813	−0.46680330	+3.43480378
O	+1.22580995	−5.13481997	+0.43444153
H	+0.48534569	−5.07602825	+1.03446584
C	−2.39897382	−1.88161712	+1.02138337
C	−4.96958528	−0.68855229	+1.92503225
H	+2.46922298	−4.23360281	+1.79421635
H	+0.71885209	−2.61750657	+1.27307919
H	+1.17248541	−2.63789359	−0.42021432
H	+4.13704484	−4.86667100	+0.37507390
H	+3.04298863	−4.95507817	−0.98372089
H	+3.38084465	−2.73212625	−1.60341588
H	+4.95928437	−3.13900007	−0.99507150
H	+5.84757469	−1.53558910	+0.78333201
H	+4.80240422	−0.14788611	+1.10520285
H	+3.20269612	−0.43185194	+2.54282166
H	+1.22050822	+0.65598291	+2.98618717
H	+0.95042590	−0.04471384	−0.51675807
H	−1.32340422	−0.89889057	−1.38002908
H	−1.08778978	+0.79864973	−1.63814646
H	−2.58297976	+1.42254674	+0.11516681
H	−3.37752454	+0.07039008	−0.67140943
H	−3.75021570	+1.02552547	+2.22073705
H	−3.39791323	−1.46793346	+3.86590314
H	−3.37315599	+0.19649118	+4.38069597
H	−1.10061227	−1.47690820	+3.46262612
H	−1.00967641	+0.09317109	+4.22230203
H	−2.49344684	−2.44135109	+1.95017096
H	−1.45357118	−2.15665091	+0.56079044
H	−3.20048468	−2.20860467	+0.36077144
H	−5.72025035	−0.36346237	+2.64284932
H	−4.91881596	−1.77492376	+1.97598877
H	−5.32387676	−0.41455343	+0.93223732
H	−1.48227860	+1.22806091	+2.17796324

51

* E = +4.978 kcal/mol ; (45) 016_313_063_009_322_062_183_240_265

C	+0.00000000	+0.00000000	+0.00000000
C	+0.00000000	+0.00000000	+1.33279000
C	+1.24442958	+0.00000000	+2.12976531
C	+1.86893494	−1.06103738	+2.63666947

C	+1.52417758	−2.48799673	+2.45116513
C	+1.21712244	−3.26682358	+3.49509583
C	+1.08794730	−2.74456862	+4.88809573
H	+1.87422980	−3.14961687	+5.52663839
C	+0.97439723	−4.73969699	+3.35087911
C	+1.47019634	−5.30291582	+2.03595995
C	+1.02746827	−4.41099187	+0.90856428
C	+1.65292307	−3.04229262	+1.05913374
C	−1.22987118	+0.02444948	−0.85428601
C	−2.51981933	+0.27235110	−0.07664873
C	−2.47577404	−0.44537110	+1.25730503
C	−1.30879247	+0.12633726	+2.05855052
C	−3.62401744	−0.15479026	+2.22647233
C	−3.02655751	−0.52684325	+3.59778606
C	−1.49441284	−0.48002610	+3.44028645
O	+1.40052166	−5.02280247	−0.30344018
H	+1.16885146	−4.44770177	−1.02957332
C	−2.32703567	−1.94796627	+1.04450775
C	−4.94255381	−0.83337080	+1.92899073
H	−0.06268037	−4.31225506	+0.95534311
H	+1.20488858	−2.34176771	+0.35515662
H	+2.70991953	−3.11606427	+0.78997544
H	+1.09797079	−6.31230871	+1.87534882
H	+2.55950306	−5.35175404	+2.02952055
H	+1.45239760	−5.25743476	+4.18383152
H	−0.09592066	−4.93444013	+3.46538654
H	+0.13966988	−3.05972754	+5.32546218
H	+1.13012985	−1.66087221	+4.93211383
H	+2.73898778	−0.86532211	+3.25347857
H	+1.64615342	+0.97675908	+2.37749980
H	+0.94834458	−0.04842130	−0.52129661
H	−1.29557141	−0.92875826	−1.38681424
H	−1.11376056	+0.77743605	−1.63439314
H	−2.63693433	+1.34168064	+0.10902429
H	−3.37786686	−0.04352327	−0.67129510
H	−3.78453063	+0.92668841	+2.20152769
H	−3.35603676	−1.52382140	+3.89002502
H	−3.37896547	+0.15036508	+4.37268142
H	−1.06115055	−1.47749205	+3.47521545
H	−1.00622329	+0.10275662	+4.21756991

H −2.41948916 −2.49957068 +1.97816315
H −1.35675002 −2.19052755 +0.61779556
H −3.09816975 −2.31425126 +0.36834530
H −5.70534103 −0.52749565 +2.64248915
H −4.85212810 −1.91651234 +1.99261240
H −5.30544700 −0.58398476 +0.93277289
H −1.51109964 +1.19952845 +2.16166102
51
* E = +5.009 kcal/mol ; (46) 312_055_347_008_321_062_183_215_039
C +0.00000000 +0.00000000 +0.00000000
C +0.00000000 +0.00000000 +1.33788562
C +1.23594962 +0.00000000 +2.11719730
C +2.37054905 +0.66788786 +1.87136372
C +2.61904585 +1.70206673 +0.86549720
C +3.78735016 +1.84247695 +0.22267834
C +4.95579831 +0.92782837 +0.34697772
H +5.84041571 +1.47546255 +0.67392227
C +3.90353451 +2.99626506 −0.72707618
C +2.68286121 +3.04850422 −1.63626720
C +1.38233956 +3.10465084 −0.84769204
C +1.59822057 +2.78106985 +0.62627540
C −1.22771281 −0.04133227 −0.85368299
C −2.52990683 +0.11174281 −0.07265958
C −2.44048103 −0.63807552 +1.24109578
C −1.31450527 −0.01139899 +2.05954355
C −3.60492456 −0.45127834 +2.21622499
C −2.98285466 −0.82360555 +3.57664359
C −1.45667725 −0.67307513 +3.42238597
O +0.85782094 +4.40943311 −0.99428945
H +0.06698246 +4.48858268 −0.46453785
C −2.19632690 −2.12131727 +0.98353276
C −4.87641113 −1.20608110 +1.89854335
H +0.67349295 +2.38745856 −1.26626326
H +0.65216895 +2.54102087 +1.10391575
H +1.96867710 +3.70097396 +1.08788623
H +2.69004311 +2.16314218 −2.26897340
H +2.71457267 +3.91651775 −2.29090521
H +3.97729643 +3.93402228 −0.16887270
H +4.81195447 +2.91633669 −1.32103969
H +5.20229430 +0.49175659 −0.62193646

H	+4.78088080	+0.11137200	+1.03962103
H	+3.20423481	+0.44368813	+2.52452531
H	+1.22964006	−0.64455934	+2.98771062
H	+0.95279360	−0.01307251	−0.51487464
H	−1.23141574	−0.98790463	−1.40110135
H	−1.16134632	+0.72814634	−1.62494511
H	−2.71083572	+1.16724588	+0.14095589
H	−3.36795962	−0.23885322	−0.67609302
H	−3.83629770	+0.61752557	+2.22315539
H	−3.24632819	−1.84760992	+3.84013384
H	−3.37524868	−0.19286784	+4.37109520
H	−0.96741813	−1.64655402	+3.43104657
H	−1.00923026	−0.08501890	+4.21985086
H	−2.27404545	−2.70824527	+1.89665327
H	−1.20840826	−2.29459534	+0.56390937
H	−2.93453768	−2.50972319	+0.28346618
H	−5.65605471	−0.97397404	+2.62152941
H	−4.71432037	−2.28228166	+1.92736539
H	−5.25723494	−0.94974173	+0.91088911
H	−1.59971933	+1.03820176	+2.20503410

51

* E = +5.076 kcal/mol ; (47) 017_313_063_009_322_062_060_241_266

C	+0.00000000	+0.00000000	+0.00000000
C	+0.00000000	+0.00000000	+1.33259000
C	+1.24409611	+0.00000000	+2.12969656
C	+1.87993831	−1.06387471	+2.61584622
C	+1.54450318	−2.49015992	+2.40606496
C	+1.23329777	−3.28358998	+3.43761950
C	+1.09283750	−2.78379120	+4.83782174
H	+1.87537606	−3.19661137	+5.47614006
C	+0.99803725	−4.75519233	+3.26942589
C	+1.52822659	−5.29193821	+1.95633393
C	+1.09009215	−4.39006966	+0.82769083
C	+1.68448302	−3.01648283	+1.00548438
C	−1.22981835	+0.01439816	−0.85404715
C	−2.52222777	+0.24983671	−0.07660371
C	−2.47108481	−0.46649090	+1.25782363
C	−1.30957458	+0.11743486	+2.05849427
C	−3.62202304	−0.18655065	+2.22692631
C	−3.02090120	−0.55166690	+3.59850416

C	−1.48938664	−0.49072780	+3.44010090
O	+1.50782992	−4.85654715	−0.43355941
H	+1.13394861	−5.72127429	−0.58751333
C	−2.30776369	−1.96779936	+1.04534746
C	−4.93390056	−0.87823093	+1.93012502
H	−0.00233891	−4.30782213	+0.85188668
H	+1.22445861	−2.32449654	+0.30527207
H	+2.74181248	−3.06489991	+0.73355808
H	+1.17601854	−6.31125355	+1.79192485
H	+2.61815405	−5.32323205	+1.96981875
H	+1.45919320	−5.28439420	+4.10466659
H	−0.07387331	−4.95675581	+3.35462954
H	+0.14179848	−3.10658375	+5.26374941
H	+1.13245244	−1.70082543	+4.89800575
H	+2.74953370	−0.87251135	+3.23468019
H	+1.63593965	+0.97600677	+2.39592471
H	+0.94759783	−0.04748878	−0.52215787
H	−1.28371685	−0.93923212	−1.38646636
H	−1.12038936	+0.76887751	−1.63366371
H	−2.65084724	+1.31798348	+0.10867558
H	−3.37701928	−0.07547221	−0.67098135
H	−3.79330043	+0.89325965	+2.20130201
H	−3.34110216	−1.55140962	+3.89187087
H	−3.37924470	+0.12305592	+4.37292970
H	−1.04732234	−1.48440929	+3.47350904
H	−1.00591129	+0.09553175	+4.21780014
H	−2.39332521	−2.51938195	+1.97989387
H	−1.33695778	−2.20154112	+0.61511470
H	−3.07745244	−2.34101431	+0.37123423
H	−5.69984748	−0.57935159	+2.64327946
H	−4.83254427	−1.96039775	+1.99453847
H	−5.29892967	−0.63338843	+0.93356054
H	−1.52247954	+1.18861132	+2.16110920

51

* E = +5.120 kcal/mol ; (48) 316_062_333_007_320_064_183_309_141

C	+0.00000000	+0.00000000	+0.00000000
C	+0.00000000	+0.00000000	+1.33819585
C	+1.27544267	+0.00000000	+2.06344273
C	+1.58870204	+0.66660026	+3.18069470
C	+0.77753814	+1.64839178	+3.89733570

C	+0.19408223	+2.71585178	+3.33381970
C	+0.31488458	+3.11638154	+1.90502097
H	+0.48922451	+4.19020285	+1.83951194
C	−0.63995235	+3.58546001	+4.22738014
C	−1.50257148	+2.70928715	+5.11953143
C	−0.65913558	+1.82485157	+6.01436182
C	+0.71656910	+1.53343082	+5.39945332
C	−1.22071701	+0.02608431	−0.86276104
C	−2.51364904	+0.22420336	−0.07874088
C	−2.45323799	−0.57123529	+1.20852552
C	−1.31582195	−0.00269690	+2.05877110
C	−3.61458619	−0.38900777	+2.18899402
C	−3.00433976	−0.84102910	+3.53161810
C	−1.47523405	−0.74867710	+3.37308486
O	−0.51589379	+2.50229905	+7.24668337
H	+0.04728168	+1.99098503	+7.82406911
C	−2.26144037	−2.05313010	+0.89708969
C	−4.90940424	−1.08723775	+1.83793126
H	−1.18911152	+0.88588720	+6.18847211
H	+1.07363492	+0.55668672	+5.72240394
H	+1.39629506	+2.27618513	+5.82617589
H	−2.12784410	+2.08258556	+4.48303987
H	−2.16055549	+3.29846242	+5.75437808
H	−0.00324657	+4.22263641	+4.84808555
H	−1.26004051	+4.25325695	+3.63197851
H	−0.60479917	+2.91262635	+1.35104043
H	+1.12236285	+2.59734496	+1.40063833
H	+2.56370516	+0.46542087	+3.61215181
H	+2.05194890	−0.61748958	+1.62835000
H	+0.95204188	−0.01552331	−0.51760132
H	−1.26701995	−0.90903751	−1.42797488
H	−1.11197662	+0.80803342	−1.61528261
H	−2.63737146	+1.28106024	+0.16773581
H	−3.36939169	−0.06481515	−0.69004343
H	−3.81145455	+0.68550892	+2.24252669
H	−3.31398594	−1.86051831	+3.76090945
H	−3.36443873	−0.22139667	+4.35136499
H	−1.02126469	−1.73620337	+3.30643988
H	−0.99611122	−0.23927063	+4.20263245
H	−2.38659065	−2.67194347	+1.78313711

H −1.27201477 −2.25331861 +0.49389213
H −2.99824427 −2.38163491 +0.16529465
H −5.68186674 −0.86145786 +2.57064399
H −4.78334008 −2.16848113 +1.81945906
H −5.28036036 −0.77528951 +0.86253651
H −1.59106990 +1.03780295 +2.26273904
51
* E = +5.254 kcal/mol ; (49) 016_313_062_009_322_062_297_241_266
C +0.00000000 +0.00000000 +0.00000000
C +0.00000000 +0.00000000 +1.33261000
C +1.24373580 +0.00000000 +2.13027865
C +1.87751286 −1.06401324 +2.61925177
C +1.54022274 −2.48987499 +2.41013160
C +1.22959963 −3.28519540 +3.44050905
C +1.09045970 −2.78554703 +4.84087546
H +1.87250309 −3.20025896 +5.47848852
C +0.98947519 −4.75599282 +3.27214857
C +1.50579254 −5.29893963 +1.95575931
C +1.07380078 −4.39464326 +0.82697188
C +1.67437164 −3.01850060 +1.00868811
C −1.22967174 +0.01401316 −0.85421205
C −2.52183396 +0.25116205 −0.07687756
C −2.47157990 −0.46479801 +1.25782620
C −1.30957495 +0.11824063 +2.05848269
C −3.62223551 −0.18274178 +2.22671409
C −3.02175862 −0.54800702 +3.59853475
C −1.49012876 −0.48893912 +3.44044057
O +1.37429975 −4.92674021 −0.44046295
H +2.32318980 −5.00600054 −0.52340344
C −2.31041853 −1.96631833 +1.04584448
C −4.93490981 −0.87297544 +1.93016992
H −0.01294394 −4.31351489 +0.84451887
H +1.21965398 −2.32400638 +0.30732269
H +2.73771129 −3.05777552 +0.74591820
H +1.14346921 −6.30954370 +1.77915597
H +2.59797676 −5.34084975 +1.97778848
H +1.45193212 −5.28656031 +4.10586753
H −0.08259772 −4.95327385 +3.36260331
H +0.13925645 −3.10776454 +5.26659003
H +1.13150703 −1.70271302 +4.90192325

H	+2.74561178	−0.87271826	+3.24029159
H	+1.63619732	+0.97590060	+2.39598520
H	+0.94780345	−0.04633304	−0.52211668
H	−1.28428124	−0.94022667	−1.38534190
H	−1.11972211	+0.76767079	−1.63456380
H	−2.64942194	+1.31955230	+0.10782801
H	−3.37667117	−0.07376224	−0.67123471
H	−3.79206644	+0.89730550	+2.20060052
H	−3.34307567	−1.54728367	+3.89212039
H	−3.37937516	+0.12742015	+4.37266823
H	−1.04954891	−1.48324279	+3.47461222
H	−1.00616325	+0.09724420	+4.21793431
H	−2.39573783	−2.51762123	+1.98054310
H	−1.34094814	−2.20182487	+0.61374359
H	−3.08078197	−2.33943897	+0.37272509
H	−5.70042367	−0.57290800	+2.64329043
H	−4.83484960	−1.95520324	+1.99473993
H	−5.29966766	−0.62797716	+0.93357720
H	−1.52128041	+1.18969664	+2.16065989

51

* E = +5.305 kcal/mol ; (50) 313_061_338_008_321_062_066_218_038

C	+0.00000000	+0.00000000	+0.00000000
C	+0.00000000	+0.00000000	+1.33805000
C	+1.23374787	+0.00000000	+2.12127362
C	+2.37619325	+0.64894595	+1.86297955
C	+2.62880251	+1.64471025	+0.81888445
C	+3.77560289	+1.71499843	+0.12909950
C	+4.91423064	+0.76268945	+0.24976266
H	+5.83387250	+1.29145890	+0.50266784
C	+3.88120692	+2.80077498	−0.89923787
C	+2.60698162	+2.80881145	−1.73216881
C	+1.38076091	+3.08506201	−0.87073668
C	+1.62936355	+2.74592338	+0.59183566
C	−1.22818085	−0.03961046	−0.85317004
C	−2.52972291	+0.12274413	−0.07313295
C	−2.44441759	−0.62497367	+1.24203013
C	−1.31500440	−0.00261728	+2.05891483
C	−3.60777478	−0.42887375	+2.21665916
C	−2.98811937	−0.80197795	+3.57789029
C	−1.46121154	−0.65998295	+3.42348346

O	+1.02658567	+4.45398546	−0.88158179
H	+0.74173743	+4.69763849	−1.75938920
C	−2.20887324	−2.11019252	+0.98815645
C	−4.88384122	−1.17663647	+1.90060636
H	+0.53918743	+2.49319638	−1.23766171
H	+0.69005080	+2.54539179	+1.09449333
H	+2.03329912	+3.65925244	+1.03591424
H	+2.50497726	+1.83369804	−2.20799754
H	+2.65903849	+3.55100960	−2.52897657
H	+4.00419096	+3.77634641	−0.42057501
H	+4.75338563	+2.64806474	−1.53211474
H	+5.09652634	+0.26173582	−0.70208158
H	+4.74256617	−0.00477041	+0.99719371
H	+3.20725464	+0.43585052	+2.52326110
H	+1.21598179	−0.62344955	+3.00671927
H	+0.95247606	−0.01435138	−0.51603839
H	−1.23615180	−0.98819926	−1.39733956
H	−1.15933775	+0.72715128	−1.62700365
H	−2.70327453	+1.17971472	+0.13787354
H	−3.36953751	−0.22496072	−0.67597411
H	−3.83239317	+0.64124717	+2.22120837
H	−3.25729883	−1.82403996	+3.84338366
H	−3.37710924	−0.16736010	+4.37091367
H	−0.97701890	−1.63604143	+3.43527645
H	−1.01138435	−0.07181593	+4.21950712
H	−2.28909676	−2.69417181	+1.90299483
H	−1.22208949	−2.28998350	+0.56852675
H	−2.94978031	−2.49640234	+0.28963759
H	−5.66215393	−0.93792398	+2.62291490
H	−4.72854912	−2.25380701	+1.93201194
H	−5.26295741	−0.92019869	+0.91228451
H	−1.59306406	+1.04907596	+2.20068574

51

* E = +5.488 kcal/mol ; (51) 343_046_299_316_031_018_192_121_339

C	+0.00000000	+0.00000000	+0.00000000
C	+0.00000000	+0.00000000	+1.34098686
C	+1.18676117	+0.00000000	+2.18784641
C	+2.43867959	−0.37339916	+1.89413156
C	+2.91805782	−1.03374711	+0.66640762
C	+3.85315805	−0.49366100	−0.12147817

C	+4.43147379	+0.86121309	+0.11885324
H	+4.32005098	+1.48317018	−0.76996821
C	+4.35702484	−1.18978198	−1.34771906
C	+4.03118666	−2.66831692	−1.38450824
C	+2.58708243	−2.89294098	−1.00799802
C	+2.33819700	−2.39507576	+0.39869420
C	−1.29765279	+0.12405570	−0.73230244
C	−2.43029702	−0.75060812	−0.16567916
C	−2.30968477	−0.97019353	+1.34384948
C	−1.36639961	+0.08016371	+1.95340309
C	−3.57557489	−0.68826743	+2.16034479
C	−3.04585471	−0.49229313	+3.58844110
C	−1.57017418	−0.07227807	+3.45242310
O	+1.80571644	−2.19309679	−1.95248183
H	+0.90368532	−2.15501260	−1.64012224
C	−1.79827447	−2.37894605	+1.61750989
C	−4.68560304	−1.70903378	+2.04976584
H	+2.35191887	−3.95955237	−1.05827409
H	+2.75477818	−3.11021877	+1.11160570
H	+1.26596015	−2.37459504	+0.59825793
H	+4.65722099	−3.21126984	−0.67647520
H	+4.22433752	−3.07647169	−2.37441716
H	+3.92203402	−0.70081962	−2.22197333
H	+5.43552422	−1.04332307	−1.42553067
H	+5.50138392	+0.79578620	+0.32033694
H	+3.95435166	+1.37520749	+0.94718061
H	+3.18270001	−0.20163535	+2.66337689
H	+1.02745699	+0.39561399	+3.18295721
H	+0.92577931	−0.00009931	−0.55801633
H	−1.16466901	−0.07332422	−1.79380492
H	−1.61190005	+1.17080287	−0.66795120
H	−3.38521738	−0.28228574	−0.40615163
H	−2.42973356	−1.71391210	−0.67397196
H	−3.96047602	+0.27089220	+1.80335954
H	−3.13117686	−1.42337873	+4.14840389
H	−3.63548005	+0.24481607	+4.12878218
H	−0.91025495	−0.84120566	+3.85173859
H	−1.34678122	+0.84429026	+3.99355112
H	−1.71770631	−2.59297662	+2.68178574
H	−0.81609349	−2.52029387	+1.17297773

H −2.46762303 −3.11723031 +1.17997696
H −5.56304398 −1.39074313 +2.60943131
H −4.37666791 −2.67380665 +2.44848081
H −4.99022980 −1.85504618 +1.01400913
H −1.79302895 +1.04939495 +1.66457666
51
* E = +5.549 kcal/mol ; (52) 049_318_352_007_322_062_188_212_038
C +0.00000000 +0.00000000 +0.00000000
C +0.00000000 +0.00000000 +1.33763283
C +1.23412086 +0.00000000 +2.11992739
C +2.37900924 +0.64810696 +1.86477908
C +2.64300728 +1.65821132 +0.84222339
C +3.83304539 +1.80369916 +0.24183374
C +5.01327029 +0.91657612 +0.43322894
H +5.86171656 +1.48396124 +0.81742178
C +3.97550033 +2.94522080 −0.71762156
C +3.48136563 +4.25670904 −0.11268134
C +2.18627855 +4.08950090 +0.67469366
C +1.60528170 +2.69201289 +0.50515883
C −1.22663178 −0.02317931 −0.85462703
C −2.52950559 +0.12880959 −0.07379499
C −2.44344683 −0.62692204 +1.23693053
C −1.31546543 −0.00637508 +2.05760390
C −3.60773417 −0.43905071 +2.21217849
C −2.98788680 −0.81827881 +3.57148609
C −1.46153558 −0.67004905 +3.41922578
O +2.49825782 +4.34598725 +2.02767997
H +1.74088304 +4.13526227 +2.57019357
C −2.20557747 −2.11004972 +0.97356654
C −4.88238228 −1.18735977 +1.89176289
H +1.44550150 +4.81814255 +0.33536227
H +1.25931996 +2.58655879 −0.52652613
H +0.72359961 +2.57985623 +1.13285121
H +3.36022632 +5.00028958 −0.89759470
H +4.22294367 +4.64253320 +0.58253081
H +5.00961935 +3.05063196 −1.04009896
H +3.39754686 +2.72722069 −1.62090697
H +5.32817810 +0.49416552 −0.52171231
H +4.81654158 +0.08990261 +1.10722723
H +3.20927882 +0.42486049 +2.52195731

H	+1.21878502	−0.62904158	+3.00161871
H	+0.95207207	−0.01755215	−0.51566739
H	−1.23586845	−0.96141266	−1.41627101
H	−1.15282472	+0.75778329	−1.61350227
H	−2.70772947	+1.18366002	+0.14463198
H	−3.36797589	−0.21705642	−0.67949635
H	−3.83476498	+0.63067646	+2.22255348
H	−3.25352400	−1.84297762	+3.83015308
H	−3.38014707	−0.19051275	+4.36841453
H	−0.97371945	−1.64422550	+3.42710292
H	−1.01416767	−0.08453812	+4.21870055
H	−2.27861445	−2.69959129	+1.88545576
H	−1.22129647	−2.28504475	+0.54650809
H	−2.95036645	−2.49366589	+0.27778631
H	−5.66188846	−0.95250331	+2.61405612
H	−4.72570880	−2.26436935	+1.91942953
H	−5.26064710	−0.92785694	+0.90392422
H	−1.59756821	+1.04408386	+2.20376882

51

* E = +5.605 kcal/mol ; (53) 311_057_344_010_321_062_067_144_319

C	+0.00000000	+0.00000000	+0.00000000
C	+0.00000000	+0.00000000	+1.33704604
C	+1.23474863	+0.00000000	+2.11995298
C	+2.36378703	−0.68356150	+1.89692513
C	+2.59784960	−1.74866389	+0.91997690
C	+3.76349118	−1.90687604	+0.27594271
C	+4.93759794	−0.99660079	+0.37881139
H	+5.20151026	−0.61018696	−0.60633675
C	+3.89294886	−3.10503225	−0.61511626
C	+3.44115873	−4.34081170	+0.14752358
C	+2.01129046	−4.19837671	+0.66520270
C	+1.52036651	−2.75668671	+0.63486947
C	−1.22776257	+0.05508304	−0.85236747
C	−2.50647562	+0.35609664	−0.07390304
C	−2.49058374	−0.37167716	+1.25513900
C	−1.30459299	+0.15349914	+2.06042764
C	−3.62597139	−0.05086838	+2.23029493
C	−3.04018492	−0.46865420	+3.59364684
C	−1.50711877	−0.46803420	+3.43427501
O	+1.08645935	−4.91748187	−0.12713226

H	+1.24929686	−5.85278016	−0.02944292
C	−2.40570006	−1.87646273	+1.02221651
C	−4.97074413	−0.67312721	+1.92721064
H	+1.96323226	−4.56744338	+1.69168828
H	+0.68976220	−2.64181866	+1.32369250
H	+1.11252548	−2.60211117	−0.36692438
H	+4.12034938	−4.48999225	+0.98569188
H	+3.50389967	−5.23038850	−0.47876771
H	+3.27559992	−2.98290188	−1.50921377
H	+4.91932992	−3.22141854	−0.95836425
H	+5.81394033	−1.53286284	+0.74552981
H	+4.75638792	−0.14511851	+1.02587810
H	+3.20134637	−0.44658307	+2.54035616
H	+1.23244794	+0.67134725	+2.97147463
H	+0.95066844	−0.04790596	−0.51633865
H	−1.32599543	−0.90097585	−1.37518392
H	−1.08694009	+0.79571197	−1.64026833
H	−2.57652969	+1.42895356	+0.11608300
H	−3.37739321	+0.07949108	−0.66941534
H	−3.74459506	+1.03628502	+2.22339880
H	−3.40161292	−1.45984323	+3.86664584
H	−3.36830371	+0.20454540	+4.38256387
H	−1.10599575	−1.48054919	+3.45739523
H	−1.00490092	+0.08708299	+4.22274658
H	−2.50610125	−2.43729946	+1.94939243
H	−1.46157851	−2.16093926	+0.56574224
H	−3.20691460	−2.19867070	+0.35881404
H	−5.71988730	−0.34635942	+2.64598609
H	−4.92311341	−1.75967084	+1.97630724
H	−5.32454603	−0.39663351	+0.93487300
H	−1.47824666	+1.22996087	+2.18158337

51

* E = +5.676 kcal/mol ; (54) 351_042_297_314_033_016_287_302_350

C	+0.00000000	+0.00000000	+0.00000000
C	+0.00000000	+0.00000000	+1.34051443
C	+1.16712346	+0.00000000	+2.21437688
C	+2.47421434	−0.17886469	+1.97141797
C	+3.20270851	−0.50592029	+0.74248734
C	+2.99097053	−1.60035520	−0.00597666
C	+1.95965337	−2.62577528	+0.30947066

H	+2.36872669	−3.62750176	+0.17911724
C	+3.79116777	−1.88854877	−1.24313484
C	+4.69331799	−0.75282395	−1.68239022
C	+5.37102511	−0.14042975	−0.47438690
C	+4.31977832	+0.45982261	+0.43292714
C	−1.29198064	+0.15185129	−0.73418697
C	−2.42334235	−0.73569487	−0.18904602
C	−2.32231410	−0.95181452	+1.32240983
C	−1.37378276	+0.08908644	+1.94141326
C	−3.59577369	−0.65205778	+2.11983694
C	−3.08479439	−0.45095317	+3.55395058
C	−1.59917057	−0.06167542	+3.43774247
O	+6.14429927	−1.09733653	+0.21344356
H	+5.54449754	−1.68285004	+0.67651022
C	−1.82767536	−2.36504458	+1.60401915
C	−4.71357352	−1.66329939	+2.00094108
H	+6.07470827	+0.62868899	−0.78358154
H	+3.90862791	+1.36498378	−0.01790207
H	+4.79206144	+0.77333220	+1.36459229
H	+4.10962583	+0.01963629	−2.18387282
H	+5.43785235	−1.11279576	−2.38885147
H	+4.38472624	−2.79105309	−1.07156731
H	+3.10261308	−2.15621596	−2.04672757
H	+1.11176103	−2.53730760	−0.37353627
H	+1.57844782	−2.52697114	+1.32056782
H	+3.12823039	+0.00961336	+2.81673916
H	+0.93323250	+0.24438687	+3.24213996
H	+0.92222674	−0.03990465	−0.55884737
H	−1.15657252	−0.02551003	−1.79838148
H	−1.60613748	+1.19686079	−0.64446548
H	−3.38000422	−0.27998740	−0.44664115
H	−2.39705911	−1.69834901	−0.69709698
H	−3.96713072	+0.30797732	+1.75092115
H	−3.19705696	−1.37348257	+4.12313829
H	−3.66752784	+0.30411245	+4.07673223
H	−0.96473649	−0.84773958	+3.84461797
H	−1.36247091	+0.84807767	+3.98478059
H	−1.76637827	−2.57966069	+2.66967166
H	−0.84147399	−2.51382901	+1.17105789
H	−2.49847903	−3.09680523	+1.15769036

H	−5.59518063	−1.33388667	+2.54753839
H	−4.41821562	−2.62841312	+2.40893303
H	−5.00616149	−1.81274389	+0.96220419
H	−1.78856410	+1.06300060	+1.65109876

51

* E = +5.677 kcal/mol ; (55) 040_298_037_009_322_062_058_136_326

C	+0.00000000	+0.00000000	+0.00000000
C	+0.00000000	+0.00000000	+1.33799547
C	+1.22473460	+0.00000000	+2.13471263
C	+2.40312111	−0.57535664	+1.86467285
C	+2.72356132	−1.47823073	+0.75119898
C	+3.83777119	−1.36596039	+0.01933808
C	+4.85695477	−0.29135890	+0.18530973
H	+4.93220112	+0.30974705	−0.72191374
C	+4.05600890	−2.34142450	−1.10010942
C	+2.73693942	−2.59177344	−1.80702354
C	+1.72752790	−3.21979259	−0.86693776
C	+1.82304174	−2.66946193	+0.55487800
C	−1.22554738	+0.02882011	−0.85374069
C	−2.51870814	+0.27231023	−0.07989816
C	−2.47507481	−0.45800378	+1.24733591
C	−1.31283880	+0.11565636	+2.05537469
C	−3.62543778	−0.18155024	+2.21864763
C	−3.03056035	−0.57646798	+3.58357223
C	−1.49844564	−0.50221476	+3.43417066
O	+0.40334558	−2.99159412	−1.31225251
H	+0.30455390	−3.35204066	−2.19123908
C	−2.32900042	−1.95718085	+1.00845553
C	−4.94548330	−0.85287572	+1.91183042
H	+1.90347822	−4.29804045	−0.83126029
H	+2.19100423	−3.45703763	+1.21479806
H	+0.81617567	−2.44330650	+0.88986855
H	+2.86957170	−3.21843940	−2.68962956
H	+2.33346303	−1.63425541	−2.13813056
H	+4.78986266	−1.94708808	−1.80096923
H	+4.46887356	−3.28297644	−0.72444660
H	+5.84497333	−0.72098822	+0.35578674
H	+4.62791009	+0.37941165	+1.00656002
H	+3.20725696	−0.37389077	+2.56136151
H	+1.16368636	+0.56235339	+3.05932225

H	+0.94763204	−0.04030591	−0.52145031
H	−1.27299550	−0.92675402	−1.38188285
H	−1.10618306	+0.78476969	−1.63108917
H	−2.63981157	+1.34011961	+0.11372058
H	−3.37429385	−0.04217574	−0.67914506
H	−3.78454988	+0.90053276	+2.21141211
H	−3.34526577	−1.58577048	+3.84814684
H	−3.39631169	+0.07535590	+4.37399541
H	−1.04855999	−1.49336053	+3.47271576
H	−1.03224949	+0.08541711	+4.22134965
H	−2.36589788	−2.52129857	+1.93875187
H	−1.39734108	−2.20113436	+0.50358845
H	−3.14421733	−2.31326220	+0.37984682
H	−5.70990424	−0.54844403	+2.62443737
H	−4.85962120	−1.93667032	+1.96814206
H	−5.30304070	−0.59518510	+0.91572782
H	−1.52740798	+1.18560035	+2.16725315

51

* E = +5.690 kcal/mol ; (56) 039_298_038_008_322_062_172_133_328

C	+0.00000000	+0.00000000	+0.00000000
C	+0.00000000	+0.00000000	+1.33856297
C	+1.22244423	+0.00000000	+2.13933333
C	+2.41270977	−0.54500578	+1.85887316
C	+2.75446759	−1.40886138	+0.71997609
C	+3.84287809	−1.22461392	−0.03469192
C	+4.80930110	−0.10337963	+0.13821545
H	+4.83108303	+0.52493807	−0.75316368
C	+4.08658208	−2.16239207	−1.17998165
C	+2.77153764	−2.48765739	−1.86082144
C	+1.82490896	−3.18514829	−0.91370373
C	+1.90791433	−2.63899059	+0.51877511
C	−1.22479733	+0.03382528	−0.85499272
C	−2.52004125	+0.27024988	−0.08223692
C	−2.47535183	−0.45839055	+1.24563186
C	−1.31431588	+0.11592718	+2.05488410
C	−3.62853970	−0.19153566	+2.21540241
C	−3.03304144	−0.58457275	+3.58061928
C	−1.50107169	−0.50384588	+3.43295986
O	+0.53066464	−3.03445732	−1.46080036
H	−0.08418835	−3.57144955	−0.96575179

C	−2.31870607	−1.95669087	+1.00854584
C	−4.94293511	−0.87178865	+1.90372225
H	+2.07674749	−4.24885259	−0.89213625
H	+2.31629811	−3.41365921	+1.17182681
H	+0.89891125	−2.44854441	+0.87534671
H	+2.91317246	−3.09698370	−2.75075505
H	+2.29659348	−1.55785824	−2.17458948
H	+4.77288516	−1.70733310	−1.89217526
H	+4.57343102	−3.07999871	−0.83442638
H	+5.82165396	−0.48643460	+0.27277664
H	+4.56681932	+0.53172571	+0.98381229
H	+3.21001748	−0.34813353	+2.56494856
H	+1.14751523	+0.53639601	+3.07807450
H	+0.94604906	−0.04222654	−0.52335022
H	−1.26970922	−0.91219390	−1.40086246
H	−1.10394976	+0.79733279	−1.62434643
H	−2.64606083	+1.33711336	+0.11214695
H	−3.37390467	−0.04707676	−0.68227714
H	−3.79456127	+0.88927901	+2.20890751
H	−3.34429940	−1.59492611	+3.84562752
H	−3.40223270	+0.06557310	+4.37063414
H	−1.04683076	−1.49318744	+3.47194961
H	−1.03844772	+0.08437989	+4.22159671
H	−2.37468399	−2.52420067	+1.93556519
H	−1.36348878	−2.17534123	+0.53721864
H	−3.10921915	−2.31792735	+0.35234309
H	−5.71041810	−0.57761650	+2.61713559
H	−4.84894606	−1.95534538	+1.95485702
H	−5.30183016	−0.61176683	+0.90882170
H	−1.53026346	+1.18535451	+2.16649304

51

* E = +5.723 kcal/mol ; (57) 313_056_345_008_321_062_292_215_040

C	+0.00000000	+0.00000000	+0.00000000
C	+0.00000000	+0.00000000	+1.33769290
C	+1.23681774	+0.00000000	+2.11575788
C	+2.36444636	+0.67989343	+1.87259469
C	+2.59446785	+1.72144215	+0.86907348
C	+3.75467342	+1.87311632	+0.21438577
C	+4.93190965	+0.96757932	+0.32243420
H	+5.81649798	+1.52056312	+0.64051795

C	+3.84916472	+3.02636092	−0.73884593
C	+2.61201770	+3.06122078	−1.62727566
C	+1.31823362	+3.13162089	−0.81562843
C	+1.55892178	+2.79062728	+0.65018091
C	−1.22800950	−0.03497626	−0.85324287
C	−2.52912171	+0.12342565	−0.07148211
C	−2.44247244	−0.62763595	+1.24180762
C	−1.31375965	−0.00569502	+2.06002068
C	−3.60536935	−0.43600686	+2.21787246
C	−2.98424505	−0.81143382	+3.57787815
C	−1.45754741	−0.66664497	+3.42301354
O	+0.67963534	+4.38466608	−0.92846009
H	+1.21515703	+5.04543341	−0.49211889
C	−2.20483802	−2.11187969	+0.98387766
C	−4.88016801	−1.18545489	+1.90079104
H	+0.59620840	+2.43112134	−1.22586592
H	+0.61844286	+2.55111279	+1.13345657
H	+1.93162655	+3.70305765	+1.13156939
H	+2.61217973	+2.15964201	−2.23767723
H	+2.63383819	+3.90618515	−2.31335822
H	+3.93238271	+3.96536191	−0.18084553
H	+4.74941666	+2.95274379	−1.34590146
H	+5.17026619	+0.53457724	−0.64991401
H	+4.77066879	+0.14888563	+1.01565928
H	+3.20342019	+0.46069082	+2.52061614
H	+1.23655992	−0.65110076	+2.98148465
H	+0.95302070	−0.01560728	−0.51482560
H	−1.23527648	−0.98039561	−1.40275615
H	−1.15941861	+0.73701376	−1.62160189
H	−2.70387409	+1.17974051	+0.14094590
H	−3.36848753	−0.22403014	−0.67491512
H	−3.83202246	+0.63363822	+2.22500198
H	−3.25144109	−1.83463025	+3.84097028
H	−3.37381560	−0.17947765	+4.37276244
H	−0.97200047	−1.64207331	+3.43188101
H	−1.00777512	−0.07990027	+4.22022473
H	−2.28339258	−2.69898655	+1.89688860
H	−1.21843655	−2.28918789	+0.56222312
H	−2.94583388	−2.49692951	+0.28486627
H	−5.65843890	−0.95032436	+2.62431882

H −4.72251002 −2.26238510 +1.92901311
H −5.26036559 −0.92691704 +0.91349210
H −1.59358780 +1.04526647 +2.20428837
51
* E = +5.790 kcal/mol ; (58) 317_062_333_007_320_064_294_309_141
C +0.00000000 +0.00000000 +0.00000000
C +0.00000000 +0.00000000 +1.33819917
C +1.27562633 +0.00000000 +2.06312599
C +1.58908922 +0.66533093 +3.18090650
C +0.77784298 +1.64641570 +3.89875723
C +0.19547712 +2.71402105 +3.33393455
C +0.31662307 +3.11593577 +1.90550729
H +0.48976048 +4.19002034 +1.83970600
C −0.64016677 +3.58316913 +4.22693054
C −1.50038080 +2.70408518 +5.12030542
C −0.65471901 +1.81533213 +6.02028456
C +0.71847929 +1.52822754 +5.39996256
C −1.22076695 +0.02477584 −0.86266237
C −2.51398331 +0.22089976 −0.07861192
C −2.45238758 −0.57418865 +1.20881823
C −1.31573136 −0.00391381 +2.05900376
C −3.61403798 −0.39342025 +2.18917907
C −3.00332978 −0.84440142 +3.53187399
C −1.47430917 −0.74962152 +3.37365804
O −0.50445973 +2.35786677 +7.31363436
H +0.00504346 +3.16466844 +7.25484699
C −2.25842399 −2.05586744 +0.89777018
C −4.90786449 −1.09348640 +1.83815292
H −1.18149115 +0.88040606 +6.19240412
H +1.07761060 +0.55758640 +5.73259153
H +1.41148060 +2.26282599 +5.82415733
H −2.11962256 +2.07849530 +4.47619500
H −2.17165121 +3.28673826 +5.74837815
H +0.00192329 +4.23095736 +4.83536179
H −1.26103623 +4.25124940 +3.63279879
H −0.60167550 +2.91034970 +1.35000058
H +1.12556943 +2.59795078 +1.40246331
H +2.56293128 +0.46265422 +3.61386775
H +2.05154944 −0.61865244 +1.62873809
H +0.95201399 −0.01559716 −0.51765520

H	−1.26564646	−0.91041483	−1.42782886
H	−1.11305767	+0.80679290	−1.61527105
H	−2.63961156	+1.27763481	+0.16762652
H	−3.36923037	−0.06962352	−0.68985344
H	−3.81247904	+0.68090643	+2.24232944
H	−3.31097523	−1.86441222	+3.76113353
H	−3.36504326	−0.22605250	+4.35181028
H	−1.01890589	−1.73642488	+3.30743930
H	−0.99570537	−0.24057007	+4.20363448
H	−2.38234508	−2.67457674	+1.78401366
H	−1.26889204	−2.25486783	+0.49427057
H	−2.99501984	−2.38561049	+0.16634691
H	−5.68055197	−0.86880672	+2.57093796
H	−4.78022734	−2.17451910	+1.81990758
H	−5.27933736	−0.78222691	+0.86272838
H	−1.59262553	+1.03645870	+2.26194040

51

* E = +5.917 kcal/mol ; (59) 347_044_297_318_028_019_291_131_337

C	+0.00000000	+0.00000000	+0.00000000
C	+0.00000000	+0.00000000	+1.33843643
C	+1.19089196	+0.00000000	+2.17715340
C	+2.43924676	−0.40597347	+1.90877252
C	+2.96444218	−1.13526084	+0.74092152
C	+4.05917969	−0.71470036	+0.09026900
C	+4.74007704	+0.57870083	+0.39682158
H	+4.91805915	+1.14073924	−0.52032155
C	+4.69930948	−1.51134689	−1.00841259
C	+4.19381669	−2.93738845	−1.10427159
C	+2.68621478	−2.96146856	−0.96243550
C	+2.29915505	−2.44465192	+0.40655749
C	−1.28800054	+0.10726228	−0.74699337
C	−2.43267117	−0.74462615	−0.16638168
C	−2.30656606	−0.97029067	+1.34127489
C	−1.36548415	+0.08036017	+1.95257004
C	−3.57077424	−0.69471350	+2.16253028
C	−3.03817625	−0.50452269	+3.59003828
C	−1.56502639	−0.07601881	+3.45195003
O	+2.06981082	−2.21129581	−1.98597269
H	+2.26832281	−1.28748143	−1.83358994
C	−1.78917001	−2.37850866	+1.60619123

C −4.67995435 −1.71621906 +2.05072513
H +2.31123951 −3.97415901 −1.09033627
H +2.56559762 −3.18110726 +1.16728497
H +1.21956327 −2.32949986 +0.45390659
H +4.62834201 −3.53973381 −0.30616327
H +4.48887263 −3.38271975 −2.05187461
H +4.54815084 −0.99424581 −1.96105421
H +5.78025741 −1.50563138 −0.85876669
H +5.71846707 +0.40317060 +0.84671814
H +4.16082507 +1.20683321 +1.06531725
H +3.17145919 −0.20203035 +2.68109444
H +1.04302527 +0.43759551 +3.15671532
H +0.92932437 −0.00114098 −0.54988335
H −1.14128446 −0.13083491 −1.79742771
H −1.59540677 +1.15766196 −0.72014328
H −3.37948502 −0.25508468 −0.39649108
H −2.45435053 −1.70583604 −0.67659459
H −3.95816616 +0.26560980 +1.81115395
H −3.11657859 −1.43962185 +4.14424671
H −3.62995359 +0.22608213 +4.13693743
H −0.89987276 −0.84197292 +3.84836402
H −1.34584241 +0.84092749 +3.99434445
H −1.69259579 −2.59307567 +2.66916652
H −0.81564801 −2.52003907 +1.14424499
H −2.46457585 −3.11653899 +1.17793392
H −5.55662245 −1.40044478 +2.61314438
H −4.36932029 −2.68189243 +2.44578576
H −4.98606851 −1.85926200 +1.01506144
H −1.79303149 +1.04983504 +1.66655700
51
* E = +5.924 kcal/mol ; (60) 312_037_014_005_324_063_175_155_146
C +0.00000000 +0.00000000 +0.00000000
C +0.00000000 +0.00000000 +1.33967177
C +1.28628767 +0.00000000 +2.04657949
C +1.65388246 +0.55783861 +3.20883231
C +0.93814732 +1.50377013 +4.05707106
C +1.15085183 +1.61699323 +5.37880786
C +2.02546188 +0.73180871 +6.19632811
H +1.44045350 +0.25052958 +6.98113766
C +0.39090998 +2.68634405 +6.10415706

C	+0.45961590	+4.03872454	+5.39760496
C	+0.43099252	+3.91089085	+3.88110979
C	+0.00379882	+2.51425272	+3.45446176
C	−1.21215495	−0.08527260	−0.86918443
C	−2.52761088	−0.06208237	−0.09746759
C	−2.37733421	−0.81775671	+1.20653861
C	−1.32294799	−0.09301514	+2.04919286
C	−3.56714857	−0.74824388	+2.16621822
C	−2.93470356	−1.08743512	+3.52785480
C	−1.43143318	−0.77390234	+3.40655957
O	−0.44881037	+4.89983553	+3.39425957
H	−0.42746621	+4.89408036	+2.43964287
C	−2.00305096	−2.27069773	+0.93469885
C	−4.76465132	−1.60359993	+1.81728130
H	+1.43204997	+4.08631154	+3.48059460
H	+0.00794728	+2.44528075	+2.36869566
H	−1.02487102	+2.36085532	+3.79178865
H	+1.35696316	+4.56969778	+5.70562417
H	−0.38857989	+4.65456244	+5.68877832
H	−0.65548276	+2.37610824	+6.17718880
H	+0.74583152	+2.78145734	+7.12830835
H	+2.79949945	+1.31203628	+6.69961106
H	+2.50067140	−0.05218191	+5.61738875
H	+2.65805640	+0.32111981	+3.53439542
H	+2.05821578	−0.56578242	+1.53947029
H	+0.95289963	+0.03437226	−0.51501976
H	−1.14054212	−1.00612953	−1.45507952
H	−1.19170260	+0.71942468	−1.60548030
H	−2.80703761	+0.96867779	+0.12883578
H	−3.32562182	−0.48074220	−0.71175906
H	−3.88951839	+0.29667341	+2.18523567
H	−3.09468728	−2.14014417	+3.76062041
H	−3.40642796	−0.52283471	+4.32917443
H	−0.83338837	−1.68373409	+3.42175083
H	−1.06787604	−0.14917285	+4.21688535
H	−2.01624611	−2.86730936	+1.84465712
H	−1.00962357	−2.35419805	+0.50155015
H	−2.71275301	−2.71839169	+0.24046482
H	−5.57247900	−1.44656871	+2.52951349
H	−4.51299537	−2.66261877	+1.83789209

H	−5.15039919	−1.36856676	+0.82615149
H	−1.71285971	+0.92071581	+2.17533450

51

* E = +5.958 kcal/mol ; (61) 013_317_063_313_033_016_185_233_030

C	+0.00000000	+0.00000000	+0.00000000
C	+0.00000000	+0.00000000	+1.33976157
C	+1.20118932	+0.00000000	+2.16600382
C	+2.40249669	+0.52070243	+1.88689732
C	+2.78278720	+1.34960826	+0.72960677
C	+3.82407343	+1.03985397	−0.05210007
C	+4.62838144	−0.20643194	+0.11991062
H	+5.65226325	+0.03082929	+0.41204613
C	+4.25325257	+1.90700099	−1.19761552
C	+3.61330478	+3.28000079	−1.20245609
C	+2.14958411	+3.16756954	−0.86959295
C	+1.99172143	+2.61374296	+0.52613582
C	−1.30212694	+0.01551677	−0.73156118
C	−2.32235405	−1.00013523	−0.18596867
C	−2.19401123	−1.21162740	+1.32420318
C	−1.36779859	−0.07362151	+1.95026473
C	−3.49191522	−1.06220925	+2.12481468
C	−3.00833670	−0.80086025	+3.55929050
C	−1.57330377	−0.25313799	+3.44606489
O	+1.57912810	+4.44778231	−0.99609970
H	+0.66660581	+4.41278750	−0.71719878
C	−1.53720867	−2.55903919	+1.59761284
C	−4.48320961	−2.19796037	+2.00788904
H	+1.67749807	+2.47546850	−1.57812846
H	+0.94186940	+2.42114692	+0.74294473
H	+2.32436582	+3.37204191	+1.23890826
H	+3.73658293	+3.76476195	−2.16841516
H	+4.08065590	+3.92245995	−0.45581986
H	+5.34047886	+2.00101404	−1.18373297
H	+4.02328077	+1.38280232	−2.12994588
H	+4.69350371	−0.74944871	−0.82353809
H	+4.20565024	−0.87742413	+0.86036959
H	+3.18427739	+0.33960291	+2.61475450
H	+1.10734942	−0.50335138	+3.11903915
H	+0.92816416	+0.00067526	−0.55387867
H	−1.15293362	−0.14015148	−1.79698740

H	−1.73580390	+1.01587100	−0.63273947
H	−3.32588564	−0.65656030	−0.43903285
H	−2.18578469	−1.95023117	−0.69945796
H	−3.97382841	−0.15284813	+1.75533571
H	−3.02163957	−1.72772599	+4.13231595
H	−3.67141859	−0.11150847	+4.07711861
H	−0.86500191	−0.97071252	+3.85514673
H	−1.43009224	+0.67457442	+3.99447000
H	−1.45762117	−2.77346019	+2.66231280
H	−0.53745660	−2.58776010	+1.16928500
H	−2.11486144	−3.36148941	+1.14279286
H	−5.39539503	−1.97604335	+2.55850842
H	−4.07376190	−3.12191149	+2.41265011
H	−4.75992516	−2.37928877	+0.96990672
H	−1.88966464	+0.84984051	+1.66723226

51

* E = +6.013 kcal/mol ; (62) 048_310_003_008_321_062_179_051_041

C	+0.00000000	+0.00000000	+0.00000000
C	+0.00000000	+0.00000000	+1.33613050
C	+1.23145136	+0.00000000	+2.12871830
C	+2.36052688	+0.68610472	+1.91727754
C	+2.66232575	+1.67521560	+0.88206550
C	+1.92793405	+2.74905799	+0.56664404
C	+0.64646781	+3.14004350	+1.21044522
H	−0.20072679	+2.89349844	+0.56596456
C	+2.44643216	+3.60197852	−0.55259424
C	+2.82378114	+2.74141326	−1.75333184
C	+3.71820095	+1.56416193	−1.37466360
C	+3.95419357	+1.51566578	+0.12956320
C	−1.23343247	+0.00424554	−0.84565417
C	−2.52456618	+0.21316360	−0.06009859
C	−2.46305492	−0.54639293	+1.24971852
C	−1.30991580	+0.03149140	+2.06558294
C	−3.61486091	−0.31817919	+2.23129283
C	−3.00201607	−0.72340643	+3.58654322
C	−1.47187137	−0.63180292	+3.42569662
O	+3.06291240	+0.39583393	−1.82632040
H	+3.60210850	−0.36605497	−1.62348883
C	−2.28043838	−2.03707116	+0.98407177
C	−4.91778574	−1.01855063	+1.91608798

H	+4.68461962	+1.64717516	−1.87800671
H	+4.64656423	+2.32304029	+0.38596731
H	+4.45564376	+0.58443638	+0.39175659
H	+3.29239773	+3.34928091	−2.52433738
H	+1.91898272	+2.31876585	−2.18531205
H	+1.70447772	+4.34152463	−0.84710528
H	+3.32283289	+4.16568775	−0.21673854
H	+0.61962076	+4.21540524	+1.38419496
H	+0.49648910	+2.62742417	+2.15608021
H	+3.18795311	+0.46736365	+2.58472464
H	+1.21771973	−0.64860286	+2.99708270
H	+0.94583984	−0.00821759	−0.52644831
H	−1.27899708	−0.93963648	−1.39596083
H	−1.13373797	+0.77123461	−1.61554936
H	−2.65296514	+1.27524628	+0.16123874
H	−3.38189405	−0.09378172	−0.66067095
H	−3.80241282	+0.75906060	+2.24532512
H	−3.30476165	−1.73792578	+3.84492327
H	−3.36576851	−0.08278815	+4.38685222
H	−1.02005266	−1.62342566	+3.42781009
H	−0.99931951	−0.06557941	+4.22472596
H	−2.37602212	−2.62504185	+1.89497299
H	−1.30265682	−2.24634172	+0.55765488
H	−3.03800769	−2.39186723	+0.28669913
H	−5.68434014	−0.75781654	+2.64347534
H	−4.80025820	−2.10068808	+1.93943237
H	−5.29157577	−0.74223785	+0.93108573
H	−1.55441835	+1.09098833	+2.21812351

51

* E = +6.092 kcal/mol ; (63) 310_042_007_008_321_062_177_051_043

C	+0.00000000	+0.00000000	+0.00000000
C	+0.00000000	+0.00000000	+1.33551121
C	+1.23123943	+0.00000000	+2.12849409
C	+2.34683423	+0.71203269	+1.93310890
C	+2.63058507	+1.73914703	+0.92994318
C	+1.87724440	+2.81241857	+0.65524553
C	+0.58393482	+3.14693712	+1.30756607
H	−0.25511064	+2.90627255	+0.65025365
C	+2.38256969	+3.72354582	−0.42420891
C	+3.84552441	+4.10558476	−0.21790040

C	+4.70696731	+2.92872377	+0.22614624
C	+3.92902212	+1.62155822	+0.18154059
C	−1.22873583	+0.00192629	−0.85257088
C	−2.52155374	+0.20497440	−0.06793782
C	−2.46022122	−0.55710857	+1.24029742
C	−1.31190295	+0.02367775	+2.06103714
C	−3.61540963	−0.33459433	+2.21882614
C	−3.00494570	−0.74139390	+3.57469049
C	−1.47434664	−0.64389101	+3.41897928
O	+5.83586069	+2.89036227	−0.61972667
H	+6.42047393	+2.19243374	−0.33173359
C	−2.27215797	−2.04691254	+0.97354995
C	−4.91494873	−1.03856497	+1.89780954
H	+5.03349741	+3.08783641	+1.25646879
H	+4.53595613	+0.81173681	+0.58639013
H	+3.74274023	+1.37793871	−0.86830372
H	+3.90848212	+4.90469988	+0.51674096
H	+4.26792327	+4.48994164	−1.14369068
H	+2.28138812	+3.21515533	−1.38728458
H	+1.77137163	+4.62099037	−0.49000615
H	+0.52966477	+4.21349631	+1.52248733
H	+0.44231696	+2.59418097	+2.23154255
H	+3.18049646	+0.48715962	+2.59079945
H	+1.22824623	−0.67141498	+2.97924306
H	+0.95074477	−0.01878477	−0.51680041
H	−1.27072654	−0.94154526	−1.40382007
H	−1.13194981	+0.77221629	−1.61935642
H	−2.65368001	+1.26622794	+0.15464036
H	−3.37611160	−0.10366143	−0.67131016
H	−3.80650930	+0.74196830	+2.23490211
H	−3.30443754	−1.75778828	+3.82914445
H	−3.37331293	−0.10444690	+4.37569072
H	−1.01922322	−1.63390997	+3.41975496
H	−1.00612625	−0.07775995	+4.22048969
H	−2.37071873	−2.63547991	+1.88365691
H	−1.29204295	−2.25476903	+0.55184556
H	−3.02564017	−2.40317798	+0.27259732
H	−5.68441040	−0.78225436	+2.62357004
H	−4.79410406	−2.12037076	+1.91856239
H	−5.28687686	−0.76071717	+0.91256204

H	−1.56090253	+1.08147174	+2.21535937

51

* E = +6.130 kcal/mol ; (64) 349_042_297_314_032_017_290_058_050

C	+0.00000000	+0.00000000	+0.00000000
C	+0.00000000	+0.00000000	+1.33540359
C	+1.21444058	+0.00000000	+2.15097914
C	+2.26177098	+0.81857384	+2.02996281
C	+2.44641850	+1.93901889	+1.09577667
C	+1.62629045	+2.99583304	+1.01376634
C	+0.37421610	+3.14052384	+1.81351806
H	−0.50071005	+2.95241966	+1.18792062
C	+1.88419126	+4.13118478	+0.06496424
C	+3.25917994	+4.11238658	−0.57290228
C	+3.61917056	+2.70035424	−0.98246352
C	+3.69074893	+1.82598194	+0.25028169
C	−1.30426198	+0.01398205	−0.72964571
C	−2.32988087	−0.99337371	−0.17437146
C	−2.18733844	−1.21513753	+1.33304341
C	−1.36215124	−0.07567284	+1.95480962
C	−3.47602675	−1.07722480	+2.15079110
C	−2.97531107	−0.82376884	+3.58106951
C	−1.54985067	−0.25387993	+3.45264506
O	+2.68575286	+2.18602275	−1.90642528
H	+1.86361725	+2.03576943	−1.43822623
C	−1.51947994	−2.55981100	+1.59225707
C	−4.46420172	−2.21628569	+2.03909088
H	+4.57383547	+2.68709215	−1.50283569
H	+4.57220674	+2.08362410	+0.84221965
H	+3.82065211	+0.78998799	−0.06202921
H	+4.00480657	+4.47025316	+0.13736912
H	+3.28485921	+4.77212238	−1.43741048
H	+1.11289109	+4.11862960	−0.71178265
H	+1.73135221	+5.07209617	+0.59456360
H	+0.28105756	+4.15730088	+2.19323146
H	+0.33451766	+2.45168158	+2.65071959
H	+3.11550139	+0.62082568	+2.67036592
H	+1.26131225	−0.74931596	+2.93224357
H	+0.93045010	−0.00445669	−0.55210575
H	−1.15861477	−0.15063787	−1.79415071
H	−1.73164161	+1.01780355	−0.63708757

H	−3.33189592	−0.63536793	−0.41293277
H	−2.21250418	−1.94253793	−0.69427695
H	−3.96637574	−0.16732036	+1.79404714
H	−2.96701485	−1.75711629	+4.14360277
H	−3.64098532	−0.15021042	+4.11602920
H	−0.82235573	−0.95421739	+3.85816095
H	−1.41765028	+0.67980798	+3.99438604
H	−1.41835618	−2.77435417	+2.65525632
H	−0.52848570	−2.58377413	+1.14395224
H	−2.10146408	−3.36483366	+1.14771297
H	−5.37066446	−2.00097717	+2.60166953
H	−4.04668162	−3.14096721	+2.43381428
H	−4.75240532	−2.39273000	+1.00343985
H	−1.88705897	+0.84702045	+1.67471419

51

* E = +6.139 kcal/mol ; (65) 052_311_001_007_321_064_177_310_141

C	+0.00000000	+0.00000000	+0.00000000
C	+0.00000000	+0.00000000	+1.33836174
C	+1.27867182	+0.00000000	+2.05791266
C	+1.60778291	+0.66522720	+3.17147089
C	+0.82851630	+1.65827961	+3.90320165
C	+0.22970229	+2.72953296	+3.36242674
C	+0.28917883	+3.12628778	+1.92903510
H	+0.49774497	+4.19286000	+1.84953615
C	−0.47740102	+3.64072887	+4.32085730
C	+0.45485735	+4.04432927	+5.45936884
C	+1.16516979	+2.84845548	+6.08629558
C	+0.76917489	+1.54585459	+5.40057491
C	−1.21900845	+0.01314844	−0.86489104
C	−2.51604407	+0.18773906	−0.08256468
C	−2.44191288	−0.60325313	+1.20660340
C	−1.31686946	−0.01114379	+2.05886208
C	−3.61105170	−0.44283526	+2.18075387
C	−2.99945020	−0.88499075	+3.52481436
C	−1.47158984	−0.74774872	+3.38056772
O	+2.55076969	+3.08996478	+5.96253249
H	+3.03300346	+2.38098937	+6.38283016
C	−2.22046193	−2.08194797	+0.89979029
C	−4.89085690	−1.16508026	+1.82298468
H	+0.90918179	+2.77362274	+7.14611574

H	−0.24718324	+1.29539378	+5.71853708
H	+1.40182835	+0.73250584	+5.75672680
H	−0.09343047	+4.60535587	+6.21311726
H	+1.23078109	+4.70034734	+5.07150792
H	−0.84738273	+4.52839840	+3.81253539
H	−1.35382026	+3.13317566	+4.73468694
H	−0.66217378	+2.95138851	+1.42102581
H	+1.05228188	+2.57774676	+1.38810391
H	+2.58749151	+0.46005988	+3.59001432
H	+2.05031988	−0.61952668	+1.61714869
H	+0.95266062	−0.00980263	−0.51662680
H	−1.24998855	−0.92025758	−1.43405010
H	−1.12070829	+0.79976438	−1.61402600
H	−2.66115149	+1.24236135	+0.16150828
H	−3.36538892	−0.11959659	−0.69392679
H	−3.82821596	+0.62760486	+2.23372740
H	−3.28037316	−1.91539082	+3.74224986
H	−3.38609962	−0.28396678	+4.34520856
H	−0.98842011	−1.72238685	+3.33118965
H	−1.01839772	−0.21411421	+4.20888782
H	−2.32553476	−2.69930652	+1.78946826
H	−1.23043592	−2.26305779	+0.48979612
H	−2.95557387	−2.42900980	+0.17488364
H	−5.67153002	−0.95268390	+2.55107819
H	−4.74501933	−2.24385830	+1.80673739
H	−5.26224359	−0.86124352	+0.84516778
H	−1.60475913	+1.02737732	+2.25269628

51

* E = +6.218 kcal/mol ; (66) 013_316_063_314_032_017_058_233_031

C	+0.00000000	+0.00000000	+0.00000000
C	+0.00000000	+0.00000000	+1.33914180
C	+1.20379694	+0.00000000	+2.16158471
C	+2.39449977	+0.54547961	+1.88608401
C	+2.75216633	+1.40234178	+0.74127277
C	+3.79410950	+1.12228406	−0.05057526
C	+4.61785518	−0.11513196	+0.09131491
H	+5.63979084	+0.12980440	+0.38433291
C	+4.20417719	+2.01857558	−1.18049463
C	+3.55375897	+3.38637929	−1.13564806
C	+2.08633709	+3.25210054	−0.80242580

C	+1.93497278	+2.65358122	+0.56992710
C	−1.30056619	+0.03066119	−0.73341297
C	−2.34038071	−0.96410004	−0.18533754
C	−2.20638684	−1.18867231	+1.32215787
C	−1.36662050	−0.06359516	+1.95255514
C	−3.49893238	−1.03333022	+2.13028055
C	−3.00593433	−0.78862301	+3.56440020
C	−1.56726736	−0.25125802	+3.44813875
O	+1.42162043	+4.49263291	−0.79212184
H	+1.51750330	+4.90703523	−1.64675030
C	−1.56133749	−2.54451017	+1.58177148
C	−4.50216590	−2.15811701	+2.00887326
H	+1.61468701	+2.57940010	−1.52964477
H	+0.88735939	+2.44586647	+0.76785058
H	+2.24618986	+3.39856831	+1.30556185
H	+3.68665118	+3.89879970	−2.08964747
H	+4.01910627	+4.00655981	−0.36905678
H	+5.29084472	+2.12009660	−1.17927104
H	+3.96364006	+1.51937400	−2.12397635
H	+4.68619781	−0.63813635	−0.86330866
H	+4.20761720	−0.80706558	+0.81941827
H	+3.18423933	+0.36501198	+2.60542785
H	+1.12180160	−0.52207586	+3.10579706
H	+0.92902768	+0.00029658	−0.55243324
H	−1.15172500	−0.13367760	−1.79768731
H	−1.71406304	+1.03977826	−0.64113947
H	−3.33715732	−0.59411387	−0.42762998
H	−2.23219541	−1.91442645	−0.70535003
H	−3.97328793	−0.11612925	+1.77080118
H	−3.02361475	−1.72058370	+4.12913708
H	−3.66080178	−0.09863045	+4.09176129
H	−0.86250094	−0.97673526	+3.84958548
H	−1.41457748	+0.67208447	+4.00122624
H	−1.47623594	−2.76653168	+2.64453878
H	−0.56496110	−2.58010114	+1.14624001
H	−2.15014995	−3.33824593	+1.12586714
H	−5.40984173	−1.93118999	+2.56494950
H	−4.10053319	−3.08929806	+2.40490941
H	−4.78488538	−2.32863100	+0.97064567
H	−1.87817318	+0.86786561	+1.67755784

51

* E = +6.221 kcal/mol ; (67) 343_046_299_316_030_018_058_124_341

C	+0.00000000	+0.00000000	+0.00000000
C	+0.00000000	+0.00000000	+1.34015090
C	+1.18576844	+0.00000000	+2.18680536
C	+2.45029190	−0.32907036	+1.89183852
C	+2.97569998	−0.92111941	+0.64893352
C	+3.95570487	−0.33932457	−0.05069365
C	+4.52324772	+0.99658184	+0.29781202
H	+4.47334288	+1.66456735	−0.56292799
C	+4.53341908	−0.96487535	−1.28234175
C	+4.21189363	−2.43843903	−1.41639843
C	+2.74390871	−2.68301168	−1.13982872
C	+2.40693304	−2.26087577	+0.26866408
C	−1.29787294	+0.08533530	−0.73374104
C	−2.40741548	−0.81660928	−0.16263712
C	−2.27759017	−1.03402868	+1.34598684
C	−1.36790360	+0.04661428	+1.95399841
C	−3.55107575	−0.79464528	+2.16369052
C	−3.02790792	−0.57849414	+3.59111632
C	−1.56650281	−0.11161626	+3.45332743
O	+1.92745797	−1.92223788	−2.00738349
H	+2.13728521	−2.14632744	−2.91148951
C	−1.71645802	−2.42465468	+1.61609039
C	−4.62614826	−1.85249367	+2.05591192
H	+2.51089980	−3.74434467	−1.25813212
H	+2.77789311	−3.01475011	+0.96553945
H	+1.32524483	−2.24526372	+0.38120949
H	+4.79631595	−3.01735765	−0.70071351
H	+4.48171439	−2.80082485	−2.40966390
H	+4.15662296	−0.42082315	−2.15219649
H	+5.61466094	−0.81777783	−1.28710176
H	+5.57723906	+0.91388708	+0.56687509
H	+3.99309435	+1.47140696	+1.11671596
H	+3.18024677	−0.16025964	+2.67485795
H	+1.01581864	+0.35785701	+3.19447587
H	+0.91828576	−0.01609275	−0.56803248
H	−1.15098360	−0.12505714	−1.78994900
H	−1.64119996	+1.12360887	−0.67986513
H	−3.37478408	−0.37160542	−0.39884891

H	−2.38112521	−1.77843137	−0.67200030
H	−3.96879083	+0.15046884	+1.80590190
H	−3.08270072	−1.51073568	+4.15319894
H	−3.64092336	+0.14041557	+4.13027110
H	−0.88366555	−0.86031798	+3.85247800
H	−1.37105261	+0.81129251	+3.99481293
H	−1.62433952	−2.63696666	+2.68004499
H	−0.73294818	−2.53124816	+1.16488664
H	−2.36241876	−3.18473352	+1.18053485
H	−5.51327725	−1.56410818	+2.61681334
H	−4.28330313	−2.80579948	+2.45461366
H	−4.92660318	−2.00972165	+1.02057303
H	−1.82446170	+1.00191768	+1.66510221

51

* E = +6.277 kcal/mol ; (68) 012_315_063_314_032_017_182_053_047

C	+0.00000000	+0.00000000	+0.00000000
C	+0.00000000	+0.00000000	+1.33514030
C	+1.20796041	+0.00000000	+2.15818565
C	+2.29063358	+0.77141789	+2.02148412
C	+2.54756715	+1.85009101	+1.05860746
C	+1.73788001	+2.89820939	+0.85956457
C	+0.45632611	+3.11743835	+1.59016087
H	−0.39327837	+2.86048184	+0.95422651
C	+2.05891206	+3.96733510	−0.14280907
C	+3.19438575	+3.61332443	−1.07906966
C	+4.32746921	+3.01104307	−0.29438328
C	+3.86378786	+1.71944342	+0.33952958
C	−1.30345077	+0.03579045	−0.72965750
C	−2.35012129	−0.94925220	−0.17533883
C	−2.21223537	−1.17405472	+1.33194614
C	−1.36358119	−0.05280075	+1.95434064
C	−3.49841432	−1.00988855	+2.14878860
C	−2.99340566	−0.76726232	+3.57945779
C	−1.55568309	−0.22937550	+3.45196835
O	+5.40661642	+2.80612310	−1.17405896
H	+6.12506999	+2.39131457	−0.70148801
C	−1.57301094	−2.53267433	+1.59079283
C	−4.50967058	−2.12838743	+2.03576662
H	+4.61736789	+3.71318923	+0.49540719
H	+4.61669160	+1.35365836	+1.04265078

H	+3.78995173	+0.96071330	−0.44365680
H	+3.54431138	+4.48980599	−1.61992335
H	+2.86629572	+2.88364813	−1.81998874
H	+1.15729425	+4.18861348	−0.71668910
H	+2.28937284	+4.89057501	+0.39679415
H	+0.35063592	+4.16958822	+1.85439754
H	+0.38422482	+2.52067832	+2.49400671
H	+3.11930169	+0.56212193	+2.69028100
H	+1.21873130	−0.71156483	+2.97514926
H	+0.92848662	+0.01157770	−0.55299838
H	−1.16053194	−0.13175958	−1.79415804
H	−1.70959559	+1.04893015	−0.63859683
H	−3.34453289	−0.57044475	−0.41378557
H	−2.25296451	−1.90087465	−0.69510345
H	−3.96960152	−0.08993561	+1.79203005
H	−3.00626240	−1.70055863	+4.14205742
H	−3.64442055	−0.07896185	+4.11374705
H	−0.84473179	−0.94730341	+3.85604435
H	−1.40181631	+0.69997586	+3.99538452
H	−1.47815459	−2.75049632	+2.65370388
H	−0.58179151	−2.57605166	+1.14451528
H	−2.17120951	−3.32478820	+1.14444690
H	−5.41225036	−1.89483389	+2.59734503
H	−4.11146207	−3.06149597	+2.43062839
H	−4.80015536	−2.29865175	+0.99968024
H	−1.86988854	+0.88153427	+1.67657607

51

* E = +6.289 kcal/mol ; (69) 013_314_063_314_032_017_060_053_047

C	+0.00000000	+0.00000000	+0.00000000
C	+0.00000000	+0.00000000	+1.33513976
C	+1.20807098	+0.00000000	+2.15817634
C	+2.29107736	+0.77081628	+2.02174461
C	+2.54902092	+1.84977646	+1.05911644
C	+1.73827686	+2.89737603	+0.86226126
C	+0.45764019	+3.11881429	+1.59386667
H	−0.39393327	+2.86589250	+0.95887197
C	+2.05924733	+3.96610842	−0.14067550
C	+3.18199207	+3.59479122	−1.08620983
C	+4.32549489	+2.99342563	−0.30553719
C	+3.86745788	+1.71557214	+0.34699538

C	−1.30348856	+0.03517511	−0.72976111
C	−2.34984316	−0.95032170	−0.17556169
C	−2.21124592	−1.17580966	+1.33156172
C	−1.36365167	−0.05388678	+1.95423821
C	−3.49748133	−1.01346158	+2.14864972
C	−2.99260119	−0.77089543	+3.57933078
C	−1.55558297	−0.23108231	+3.45184573
O	+5.42835750	+2.66972733	−1.11788605
H	+5.74689336	+3.46179844	−1.54500394
C	−1.57030813	−2.53379113	+1.58940283
C	−4.50769973	−2.13286366	+2.03513871
H	+4.63024124	+3.69993324	+0.47489261
H	+4.62843357	+1.37072560	+1.04740563
H	+3.80292065	+0.94465546	−0.42448793
H	+3.51475843	+4.47204116	−1.64275213
H	+2.84122095	+2.85913787	−1.81546566
H	+1.15517207	+4.19813181	−0.70639544
H	+2.30700146	+4.88664002	+0.39616062
H	+0.35511298	+4.17069598	+1.86103346
H	+0.38523883	+2.52089480	+2.49683141
H	+3.12050910	+0.56039483	+2.68881639
H	+1.21927343	−0.71262184	+2.97419043
H	+0.92859907	+0.01116318	−0.55281085
H	−1.16037841	−0.13263730	−1.79424074
H	−1.71036964	+1.04807341	−0.63897735
H	−3.34445884	−0.57151773	−0.41329462
H	−2.25278684	−1.90169860	−0.69580686
H	−3.96966280	−0.09380168	+1.79237395
H	−3.00410966	−1.70448418	+4.14147677
H	−3.64449138	−0.08376441	+4.11409485
H	−0.84372036	−0.94812361	+3.85577913
H	−1.40284621	+0.69831906	+3.99548972
H	−1.47459607	−2.75198984	+2.65214846
H	−0.57925590	−2.57571829	+1.14266797
H	−2.16774698	−3.32641938	+1.14293714
H	−5.41044388	−1.90047021	+2.59696381
H	−4.10863703	−3.06585650	+2.42940136
H	−4.79814235	−2.30281762	+0.99897796
H	−1.87083029	+0.88005632	+1.67663529

51

* E = +6.354 kcal/mol ; (70) 013_317_062_314_032_017_297_232_031

C	+0.00000000	+0.00000000	+0.00000000
C	+0.00000000	+0.00000000	+1.33915686
C	+1.20461019	+0.00000000	+2.16042015
C	+2.39467817	+0.54724442	+1.88479567
C	+2.75032869	+1.40826752	+0.74256129
C	+3.79566341	+1.13819099	−0.04845582
C	+4.62525143	−0.09556666	+0.09125517
H	+5.64497466	+0.15388508	+0.38798412
C	+4.20379620	+2.03968059	−1.17494545
C	+3.54520083	+3.40409611	−1.13609460
C	+2.07818769	+3.26681090	−0.80348919
C	+1.92603474	+2.65578021	+0.56894423
C	−1.29978819	+0.02980414	−0.73455198
C	−2.34134813	−0.96276405	−0.18545064
C	−2.20520769	−1.19050970	+1.32126545
C	−1.36631559	−0.06557586	+1.95311295
C	−3.49709496	−1.03874355	+2.13112211
C	−3.00300551	−0.79668840	+3.56525584
C	−1.56555934	−0.25617045	+3.44859665
O	+1.38558816	+4.48648146	−0.91332991
H	+1.74400580	+5.10148135	−0.27547710
C	−1.55789459	−2.54597036	+1.57720205
C	−4.49902607	−2.16450775	+2.00818498
H	+1.60703801	+2.60918645	−1.53546244
H	+0.87873879	+2.44319949	+0.76432026
H	+2.23584473	+3.38945337	+1.32109762
H	+3.66318365	+3.92084179	−2.08630023
H	+4.01882570	+4.02147865	−0.36835172
H	+5.29010062	+2.14544217	−1.17089192
H	+3.96826452	+1.54103579	−2.11977165
H	+4.69920865	−0.61361582	−0.86553270
H	+4.21673822	−0.79289377	+0.81511955
H	+3.18567315	+0.36436925	+2.60219519
H	+1.12463278	−0.52486019	+3.10329105
H	+0.92928271	+0.00022958	−0.55202313
H	−1.14981262	−0.13637973	−1.79823423
H	−1.71186710	+1.03967583	−0.64546355
H	−3.33733400	−0.58904335	−0.42497711
H	−2.23758691	−1.91256685	−0.70725773

H	−3.97299460	−0.12134749	+1.77421477
H	−3.01798282	−1.73012449	+4.12760471
H	−3.65868434	−0.10946171	+4.09522419
H	−0.85899288	−0.98087510	+3.84827768
H	−1.41438366	+0.66646818	+4.00340959
H	−1.47015116	−2.76979361	+2.63939877
H	−0.56249332	−2.57982168	+1.13934145
H	−2.14677554	−3.33956813	+1.12122415
H	−5.40647941	−1.93988730	+2.56554254
H	−4.09591318	−3.09605771	+2.40183127
H	−4.78245963	−2.33302350	+0.96985986
H	−1.87923333	+0.86562576	+1.67969031

51

* E = +6.495 kcal/mol ; (71) 052_313_358_007_321_064_057_310_141

C	+0.00000000	+0.00000000	+0.00000000
C	+0.00000000	+0.00000000	+1.33837404
C	+1.27816829	+0.00000000	+2.05923286
C	+1.60517536	+0.66511425	+3.17327675
C	+0.82194634	+1.65682412	+3.90237783
C	+0.22960001	+2.73078558	+3.36049041
C	+0.29094992	+3.12711469	+1.92678704
H	+0.49844870	+4.19390660	+1.84486483
C	−0.47638078	+3.64363369	+4.31843229
C	+0.44861705	+4.04016291	+5.46768861
C	+1.18836049	+2.83928001	+6.06851209
C	+0.76603161	+1.54342464	+5.40054935
C	−1.21890079	+0.01108210	−0.86526931
C	−2.51627343	+0.18486204	−0.08333570
C	−2.44166358	−0.60540324	+1.20623930
C	−1.31728059	−0.01192570	+2.05836959
C	−3.61119622	−0.44555741	+2.17996511
C	−2.99953157	−0.88639699	+3.52441347
C	−1.47176424	−0.74767952	+3.38063593
O	+2.58530850	+2.93093228	+5.88646557
H	+2.90171829	+3.74720414	+6.26617892
C	−2.21870159	−2.08402002	+0.90021882
C	−4.89031876	−1.16905753	+1.82219417
H	+0.97347749	+2.76576480	+7.13734470
H	−0.25162642	+1.30987952	+5.72392215
H	+1.40363604	+0.73896552	+5.75877323

H	−0.11891179	+4.56566097	+6.23424267
H	+1.19372450	+4.73740366	+5.08626176
H	−0.84312017	+4.53364188	+3.81153552
H	−1.35458944	+3.13834116	+4.73018747
H	−0.65860225	+2.94983691	+1.41610095
H	+1.05582752	+2.57819036	+1.38859072
H	+2.58245821	+0.46502611	+3.59848362
H	+2.05012541	−0.62012476	+1.61990126
H	+0.95278421	−0.00929723	−0.51641837
H	−1.24907990	−0.92289601	−1.43358283
H	−1.12163235	+0.79715006	−1.61516703
H	−2.66233320	+1.23948427	+0.16036518
H	−3.36530626	−0.12347637	−0.69471035
H	−3.82931395	+0.62477465	+2.23219467
H	−3.27950079	−1.91697199	+3.74228688
H	−3.38713957	−0.28548571	+4.34447908
H	−0.98747772	−1.72174611	+3.33219655
H	−1.01927139	−0.21347050	+4.20895338
H	−2.32307431	−2.70090774	+1.79029923
H	−1.22836344	−2.26412223	+0.49055180
H	−2.95335862	−2.43231235	+0.17539867
H	−5.67151301	−0.95677515	+2.54980007
H	−4.74361329	−2.24773210	+1.80689219
H	−5.26150017	−0.86635354	+0.84391989
H	−1.60608880	+1.02657036	+2.25133993

51

* E = +6.542 kcal/mol ; (72) 012_315_063_314_032_017_297_053_047

C	+0.00000000	+0.00000000	+0.00000000
C	+0.00000000	+0.00000000	+1.33512227
C	+1.20760382	+0.00000000	+2.15887591
C	+2.29015655	+0.77204299	+2.02488224
C	+2.54950980	+1.85144851	+1.06322685
C	+1.74006324	+2.89933331	+0.86217409
C	+0.45764382	+3.12052725	+1.59068122
H	−0.39198879	+2.86782648	+0.95310613
C	+2.06358588	+3.96902437	−0.13894562
C	+3.19598710	+3.60933517	−1.07803265
C	+4.33633440	+3.00443681	−0.29589010
C	+3.87181980	+1.72192737	+0.35537542
C	−1.30313858	+0.03614439	−0.73033194

C	−2.35068883	−0.94813692	−0.17617337
C	−2.21207434	−1.17429920	+1.33081599
C	−1.36379915	−0.05316288	+1.95402345
C	−3.49823039	−1.01136145	+2.14792882
C	−2.99328292	−0.76975544	+3.57870600
C	−1.55595626	−0.23064637	+3.45158152
O	+5.48829411	+2.80480567	−1.07751726
H	+5.28400034	+2.18234093	−1.77339548
C	−1.57206748	−2.53282042	+1.58809199
C	−4.50933781	−2.12989296	+2.03378627
H	+4.64280016	+3.70708244	+0.47897156
H	+4.63121540	+1.37235699	+1.05530961
H	+3.79697854	+0.94255869	−0.41121114
H	+3.54319156	+4.48383724	−1.62438368
H	+2.84887843	+2.87972843	−1.81448252
H	+1.16134258	+4.19874624	−0.70861649
H	+2.30487186	+4.88960096	+0.40030514
H	+0.35461769	+4.17240877	+1.85715430
H	+0.38275076	+2.52276475	+2.49350289
H	+3.11788408	+0.56278048	+2.69449811
H	+1.21796094	−0.71252648	+2.97495300
H	+0.92886571	+0.01086003	−0.55246691
H	−1.15999771	−0.13214271	−1.79476768
H	−1.70900506	+1.04945604	−0.64010622
H	−3.34473319	−0.56773974	−0.41355175
H	−2.25522720	−1.89948790	−0.69676062
H	−3.96966099	−0.09118497	+1.79202411
H	−3.00526811	−1.70353531	+4.14048449
H	−3.64477653	−0.08251020	+4.11373523
H	−0.84463279	−0.94819645	+3.85555151
H	−1.40283090	+0.69852396	+3.99545968
H	−1.47645584	−2.75146545	+2.65072845
H	−0.58105885	−2.57542067	+1.14127086
H	−2.17009709	−3.32485179	+1.14140394
H	−5.41189544	−1.89689195	+2.59560463
H	−4.11126086	−3.06343440	+2.42773275
H	−4.79992426	−2.29911543	+0.99755079
H	−1.87002773	+0.88131098	+1.67659832

51

* E = +6.569 kcal/mol ; (73) 310_056_347_005_324_063_068_153_147

C	+0.00000000	+0.00000000	+0.00000000
C	+0.00000000	+0.00000000	+1.33948359
C	+1.28749613	+0.00000000	+2.04368041
C	+1.66317614	+0.55301317	+3.20554216
C	+0.96294463	+1.49866617	+4.06872760
C	+1.17526702	+1.57653069	+5.39256791
C	+2.04064056	+0.66503985	+6.19048472
H	+1.45706954	+0.19467859	+6.98276770
C	+0.48623890	+2.68810710	+6.12327015
C	+0.73829431	+3.99763099	+5.38945093
C	+0.28773886	+3.92753869	+3.93079240
C	+0.00531832	+2.49841381	+3.48713599
C	−1.21267871	−0.07902444	−0.86895670
C	−2.52764971	−0.05031598	−0.09652353
C	−2.38023877	−0.80933335	+1.20598519
C	−1.32220467	−0.09072719	+2.04928032
C	−3.56858616	−0.73476912	+2.16720504
C	−2.93658044	−1.07923804	+3.52779272
C	−1.43120892	−0.77673728	+3.40383727
O	−0.92200186	+4.62578096	+3.71341871
H	−0.77108307	+5.56105182	+3.82836182
C	−2.01423726	−2.26386058	+0.93174532
C	−4.77141002	−1.58300448	+1.81885924
H	+1.06475035	+4.34965907	+3.29067652
H	−0.00513596	+2.45575168	+2.40275424
H	−1.01141553	+2.28505633	+3.82701414
H	+1.80448001	+4.21399779	+5.43551508
H	+0.22128559	+4.82190074	+5.87964339
H	−0.59022832	+2.50092372	+6.17135583
H	+0.83766946	+2.75188263	+7.15131624
H	+2.83953135	+1.22075485	+6.68345803
H	+2.48396498	−0.12756740	+5.59803092
H	+2.67001604	+0.31281395	+3.52074593
H	+2.05793510	−0.56299883	+1.53097861
H	+0.95280801	+0.03374991	−0.51522624
H	−1.14557589	−0.99932335	−1.45639915
H	−1.18853433	+0.72733871	−1.60325416
H	−2.80072496	+0.98146843	+0.13179155
H	−3.32817686	−0.46400922	−0.71105276
H	−3.88503751	+0.31171177	+2.18822167

```
H   −3.10415648   −2.13074305   +3.76105971
H   −3.40232614   −0.51051697   +4.32971154
H   −0.84035700   −1.69144794   +3.41237133
H   −1.06026990   −0.15953828   +4.21648661
H   −2.03256730   −2.86232997   +1.84051492
H   −1.02047885   −2.35226396   +0.50023925
H   −2.72541736   −2.70585301   +0.23531230
H   −5.57746290   −1.42177374   +2.53225226
H   −4.52636113   −2.64366033   +1.83814810
H   −5.15689305   −1.34459169   +0.82841465
H   −1.70873904   +0.92355833   +2.17797411
51
* E =  +6.628 kcal/mol ; (74) 346_047_298_314_033_016_188_302_350
C   +0.00000000   +0.00000000   +0.00000000
C   +0.00000000   +0.00000000   +1.34059625
C   +1.16629485   +0.00000000   +2.21551091
C   +2.47508886   −0.16720278   +1.97161205
C   +3.21116231   −0.48274983   +0.74493416
C   +3.00930653   −1.56814154   −0.01455181
C   +1.98366407   −2.60306238   +0.28554605
H   +2.40842377   −3.59868557   +0.15880932
C   +3.83687778   −1.84609300   −1.23180896
C   +4.65020850   −0.66051479   −1.70497863
C   +5.35447768   −0.02611179   −0.53120181
C   +4.32955776   +0.48430185   +0.45932657
C   −1.29225618   +0.15090974   −0.73410189
C   −2.42119435   −0.74098956   −0.19097993
C   −2.31844751   −0.96037926   +1.31979985
C   −1.37457287   +0.08367544   +1.94096932
C   −3.59338454   −0.66890658   +2.11781112
C   −3.08377920   −0.46792227   +3.55234893
C   −1.60013282   −0.07115309   +3.43696434
O   +6.17734933   −1.02613795   +0.02736381
H   +6.54878126   −0.70965837   +0.84737652
C   −1.81606121   −2.37173195   +1.59725103
C   −4.70601820   −1.68560328   +1.99679557
H   +5.97177946   +0.80950238   −0.87145887
H   +3.91631339   +1.42649262   +0.09223984
H   +4.82660053   +0.74046453   +1.39974973
H   +4.00301425   +0.08696469   −2.16376590
```

H	+5.37769348	−0.97039531	−2.45208487
H	+4.50530769	−2.67941427	−1.00723838
H	+3.18084383	−2.19297436	−2.03220382
H	+1.14288315	−2.52465239	−0.40770678
H	+1.59007996	−2.51176790	+1.29261812
H	+3.12412043	+0.01556236	+2.82283775
H	+0.92955422	+0.23134116	+3.24564641
H	+0.92244545	−0.04228893	−0.55800301
H	−1.15623920	−0.02428982	−1.79861067
H	−1.60998578	+1.19471704	−0.64246533
H	−3.37934385	−0.28716849	−0.44670866
H	−2.39255345	−1.70239265	−0.70128200
H	−3.96964342	+0.28997404	+1.75077524
H	−3.19149583	−1.39215982	+4.11971466
H	−3.67049178	+0.28320725	+4.07648284
H	−0.96222637	−0.85512974	+3.84229375
H	−1.36767138	+0.83853177	+3.98600947
H	−1.75182953	−2.58863423	+2.66231485
H	−0.82974356	−2.51400892	+1.16225677
H	−2.48379907	−3.10590102	+1.15018594
H	−5.58948883	−1.36188603	+2.54388745
H	−4.40560918	−2.64996681	+2.40288418
H	−4.99749918	−1.83458311	+0.95766902
H	−1.79340173	+1.05639555	+1.65224911

51

* E = +6.677 kcal/mol ; (75) 050_312_001_007_321_062_056_214_040

C	+0.00000000	+0.00000000	+0.00000000
C	+0.00000000	+0.00000000	+1.33710577
C	+1.23531754	+0.00000000	+2.11784642
C	+2.36362199	+0.67915911	+1.87732798
C	+2.59976196	+1.71773639	+0.87505384
C	+3.77566323	+1.88038004	+0.25187639
C	+4.96519223	+0.99657186	+0.39983631
H	+5.82202173	+1.55846297	+0.77400923
C	+3.89447359	+3.04838482	−0.67910432
C	+3.41702614	+4.32571313	+0.00221207
C	+2.06279075	+4.14811202	+0.69787020
C	+1.53486496	+2.73064666	+0.55844163
C	−1.22750342	−0.01595013	−0.85377479
C	−2.52809124	+0.14899229	−0.07146177

C	−2.44813519	−0.60756545	+1.23927192
C	−1.31401095	+0.00255035	+2.05877570
C	−3.60899417	−0.40882827	+2.21640215
C	−2.99048972	−0.79464286	+3.57472503
C	−1.46290122	−0.66055171	+3.42011041
O	+2.14619280	+4.40678730	+2.08320381
H	+2.49236154	+5.28560590	+2.21913521
C	−2.22449679	−2.09284598	+0.97561701
C	−4.89146097	−1.14447941	+1.89780271
H	+1.32760126	+4.82549360	+0.25575346
H	+1.17514867	+2.60161779	−0.46492094
H	+0.67671516	+2.61891951	+1.21291781
H	+3.37950629	+5.14290589	−0.71674887
H	+4.14772382	+4.59956709	+0.76247115
H	+4.92122029	+3.16806977	−1.02033399
H	+3.29316988	+2.87068736	−1.57576529
H	+5.25884283	+0.59157065	−0.56945496
H	+4.78652606	+0.15754062	+1.06354327
H	+3.19982599	+0.46357683	+2.52943556
H	+1.23200986	−0.64834113	+2.98565327
H	+0.95241266	−0.01905862	−0.51521919
H	−1.24587754	−0.95555247	−1.41305207
H	−1.14860888	+0.76298128	−1.61429967
H	−2.69487711	+1.20554762	+0.14733543
H	−3.37070276	−0.18845620	−0.67636658
H	−3.82536366	+0.66300521	+2.22775155
H	−3.26573351	−1.81691481	+3.83333348
H	−3.37530982	−0.16314351	+4.37232366
H	−0.98383081	−1.63919052	+3.42692646
H	−1.00890351	−0.07820620	+4.21793191
H	−2.30449119	−2.68174312	+1.88735260
H	−1.24123204	−2.27697249	+0.55002410
H	−2.97168479	−2.46940248	+0.27845747
H	−5.66720679	−0.90295047	+2.62201681
H	−4.74516477	−2.22305882	+1.92375196
H	−5.26917140	−0.88011159	+0.91099506
H	−1.58450054	+1.05543099	+2.20692305

51

* E = +6.759 kcal/mol ; (76) 016_314_063_314_032_017_182_299_350

C	+0.00000000	+0.00000000	+0.00000000

C	+0.00000000	+0.00000000	+1.34039747
C	+1.16836025	+0.00000000	+2.21416271
C	+2.47372280	−0.16915010	+1.96062362
C	+3.19364690	−0.46253462	+0.71651358
C	+3.02429750	−1.56352843	−0.02766594
C	+2.00188834	−2.60348147	+0.27213518
H	+2.44163013	−3.59865762	+0.21595528
C	+3.85218561	−1.82581123	−1.25170540
C	+5.10112741	−0.97409182	−1.33938052
C	+4.76373332	+0.45699590	−1.02406634
C	+4.25629916	+0.55775290	+0.39640756
C	−1.29182899	+0.14820252	−0.73574141
C	−2.42588497	−0.73629399	−0.19031520
C	−2.31710245	−0.96427007	+1.31830737
C	−1.37397203	+0.07905639	+1.94235436
C	−3.59009830	−0.68127318	+2.12284937
C	−3.07599925	−0.48846871	+3.55663079
C	−1.59535810	−0.08233816	+3.43846759
O	+5.92188563	+1.23150813	−1.22146485
H	+5.73596928	+2.14050804	−0.99593173
C	−1.80948628	−2.37592921	+1.58464991
C	−4.70098193	−1.69953042	+1.99901354
H	+3.97411752	+0.78761578	−1.70845428
H	+3.85568045	+1.55694949	+0.57857011
H	+5.10538002	+0.43853764	+1.07484419
H	+5.55028206	−1.03761788	−2.32800936
H	+5.84589842	−1.31689779	−0.62062600
H	+4.11989362	−2.88236267	−1.27644832
H	+3.22666169	−1.66687843	−2.13586128
H	+1.19401279	−2.56948519	−0.46285187
H	+1.55906872	−2.46907915	+1.25298719
H	+3.13504000	+0.00602215	+2.80391984
H	+0.93559422	+0.22757359	+3.24593572
H	+0.92220969	−0.03938732	−0.55884827
H	−1.15457597	−0.03248811	−1.79914841
H	−1.60579685	+1.19359803	−0.65026890
H	−3.38090713	−0.27164743	−0.43788545
H	−2.41027024	−1.69536879	−0.70549428
H	−3.96968845	+0.27922248	+1.76351842
H	−3.17596763	−1.41789494	+4.11688514

H	−3.66508326	+0.25486930	+4.08910110
H	−0.95160370	−0.86343312	+3.84005884
H	−1.36702239	+0.82703168	+3.98974504
H	−1.73424465	−2.59780169	+2.64799482
H	−0.82752266	−2.51490004	+1.13879391
H	−2.48010189	−3.10927509	+1.14064210
H	−5.58355123	−1.38058386	+2.55029536
H	−4.39767770	−2.66555317	+2.39895430
H	−4.99505261	−1.84320932	+0.95988117
H	−1.79487011	+1.05228571	+1.65838610

51

* E = +6.772 kcal/mol ; (77) 342_048_299_315_027_023_187_142_138

C	+0.00000000	+0.00000000	+0.00000000
C	+0.00000000	+0.00000000	+1.33766962
C	+1.25625083	+0.00000000	+2.09574457
C	+1.58502835	+0.68490741	+3.19728509
C	+0.84155600	+1.75694125	+3.87258624
C	+0.81161650	+1.86156966	+5.20807891
C	+1.39500373	+0.83891547	+6.12555036
H	+0.64075625	+0.51226738	+6.84245597
C	+0.12508611	+2.99610676	+5.90231367
C	−0.13295645	+4.18699593	+5.00490271
C	−0.74363476	+3.72605763	+3.70490598
C	+0.20792586	+2.79448242	+2.98610405
C	−1.29002561	−0.00127916	−0.75232190
C	−2.32893324	−0.99677356	−0.20023148
C	−2.13317548	−1.28734780	+1.28561890
C	−1.36316361	−0.13116553	+1.95917092
C	−3.41305950	−1.27665652	+2.12637858
C	−2.89757947	−1.10843749	+3.55896935
C	−1.53789842	−0.39517193	+3.44994794
O	−1.95667904	+3.08309890	+4.03672752
H	−2.32075921	+2.67805302	+3.25215100
C	−1.38409518	−2.60340801	+1.45678159
C	−4.35097676	−2.44756194	+1.93743519
H	−0.95073270	+4.58802442	+3.06527457
H	+0.99674663	+3.38522921	+2.51411483
H	−0.31184812	+2.31363987	+2.15741671
H	+0.80140560	+4.70191436	+4.78218396
H	−0.79435582	+4.89884403	+5.49417905

H	−0.82306308	+2.63206208	+6.30434694
H	+0.72125279	+3.29907298	+6.76462549
H	+2.21414046	+1.26125494	+6.70910675
H	+1.75668665	−0.04202666	+5.60677154
H	+2.56248963	+0.46790930	+3.60902194
H	+2.01867193	−0.65921355	+1.69881368
H	+0.93140376	+0.04494071	−0.54841918
H	−1.11963186	−0.18120418	−1.81080955
H	−1.71619181	+1.00444785	−0.68899117
H	−3.32599957	−0.59120644	−0.37513132
H	−2.27415500	−1.92712274	−0.76307752
H	−3.95087193	−0.36572447	+1.84669037
H	−2.78522634	−2.08575922	+4.02845039
H	−3.60495541	−0.55425250	+4.17208334
H	−0.72939611	−1.02595947	+3.81474598
H	−1.50278698	+0.51756208	+4.03524973
H	−1.20731992	−2.84482320	+2.50346569
H	−0.42146417	−2.56409171	+0.95304427
H	−1.95227719	−3.42053517	+1.01642708
H	−5.26109164	−2.31177869	+2.51857053
H	−3.89161696	−3.37864526	+2.26448932
H	−4.63994225	−2.56307526	+0.89328961
H	−1.93601598	+0.77272328	+1.70429272

51

* E = +6.850 kcal/mol ; (78) 048_308_006_008_321_062_059_053_042

C	+0.00000000	+0.00000000	+0.00000000
C	+0.00000000	+0.00000000	+1.33591329
C	+1.23592585	+0.00000000	+2.12134024
C	+2.35246523	+0.70221364	+1.90245185
C	+2.61878073	+1.71054014	+0.87500487
C	+1.87330882	+2.79116329	+0.61465895
C	+0.60853098	+3.15757798	+1.30513179
H	−0.25596813	+2.91617423	+0.68161410
C	+2.35015190	+3.67501219	−0.49989586
C	+2.67947098	+2.83615801	−1.72903794
C	+3.63459177	+1.68129152	−1.40959872
C	+3.88994346	+1.56933471	+0.08366133
C	−1.23419283	+0.00548881	−0.84490406
C	−2.52488134	+0.21010443	−0.05685213
C	−2.46012783	−0.55433890	+1.25004599

C	−1.30856144	+0.02481046	+2.06716396
C	−3.61180106	−0.33480634	+2.23372516
C	−2.99544330	−0.74380785	+3.58643163
C	−1.46577331	−0.64626289	+3.42392086
O	+3.10495682	+0.43668691	−1.82101724
H	+2.94283918	+0.45931999	−2.76145402
C	−2.27223604	−2.04329428	+0.97809358
C	−4.91225364	−1.03906222	+1.91694144
H	+4.59367080	+1.83874372	−1.90895723
H	+4.59291270	+2.35859385	+0.36287700
H	+4.37933351	+0.61832811	+0.27613998
H	+3.08060443	+3.46391754	−2.52361931
H	+1.75075269	+2.40059510	−2.09740160
H	+1.59725147	+4.42034506	−0.74891977
H	+3.24060341	+4.22992859	−0.18812067
H	+0.57661977	+4.22857198	+1.50332879
H	+0.49173044	+2.62354221	+2.24351738
H	+3.19539912	+0.48547481	+2.55045415
H	+1.23883108	−0.66284162	+2.97885472
H	+0.94850989	−0.01797916	−0.52185699
H	−1.28033700	−0.93687727	−1.39798602
H	−1.13875199	+0.77670388	−1.61173865
H	−2.65417304	+1.27123662	+0.16863427
H	−3.38261252	−0.09582521	−0.65744968
H	−3.80367237	+0.74161330	+2.25292862
H	−3.29440561	−1.76044616	+3.84086544
H	−3.36033146	−0.10786603	+4.38995460
H	−1.01048506	−1.63622832	+3.41980651
H	−0.99361222	−0.08266330	+4.22498374
H	−2.36671008	−2.63523018	+1.88647229
H	−1.29328515	−2.24780812	+0.55205957
H	−3.02826687	−2.39775846	+0.27879956
H	−5.67881965	−0.78525291	+2.64676731
H	−4.79013553	−2.12079185	+1.93463888
H	−5.28872619	−0.75940555	+0.93387262
H	−1.55738257	+1.08231371	+2.22555135

51

* E = +6.869 kcal/mol ; (79) 017_313_063_314_032_017_060_299_350

C	+0.00000000	+0.00000000	+0.00000000
C	+0.00000000	+0.00000000	+1.34027252

C	+1.16858716	+0.00000000	+2.21367131
C	+2.47356224	−0.17185903	+1.96054707
C	+3.19449861	−0.47291320	+0.71874656
C	+3.01846439	−1.57642873	−0.02027162
C	+1.98816646	−2.60858903	+0.27921279
H	+2.42036014	−3.60730320	+0.22555040
C	+3.85133629	−1.84940401	−1.23867941
C	+5.11934937	−1.02309799	−1.29784412
C	+4.80209585	+0.42126909	−0.99708741
C	+4.26077488	+0.54338267	+0.40342801
C	−1.29127122	+0.15174242	−0.73605667
C	−2.42911858	−0.72768944	−0.19022936
C	−2.32026007	−0.95733570	+1.31811220
C	−1.37360638	+0.08243048	+1.94252917
C	−3.59189730	−0.67126604	+2.12375282
C	−3.07620408	−0.48173580	+3.55741642
C	−1.59454462	−0.07962924	+3.43862532
O	+5.93434475	+1.25377548	−1.07334003
H	+6.29484798	+1.21379722	−1.95639147
C	−1.81706787	−2.37088778	+1.58284434
C	−4.70614743	−1.68580078	+1.99942919
H	+4.03718174	+0.76740325	−1.70178713
H	+3.86646758	+1.54758072	+0.55149360
H	+5.09508626	+0.43940767	+1.10187236
H	+5.58790418	−1.11678720	−2.27849952
H	+5.83891936	−1.37780748	−0.55938007
H	+4.09619457	−2.91130202	−1.26978026
H	+3.24239034	−1.66739878	−2.13003104
H	+1.18042603	−2.56988578	−0.45583139
H	+1.54565136	−2.46904000	+1.25949248
H	+3.13540073	+0.00853407	+2.80214694
H	+0.93666432	+0.23314008	+3.24438647
H	+0.92252006	−0.03911705	−0.55840964
H	−1.15452272	−0.03051459	−1.79931053
H	−1.60103216	+1.19848756	−0.65191341
H	−3.38213115	−0.25822901	−0.43655215
H	−2.41877125	−1.68655461	−0.70601764
H	−3.96865040	+0.29084721	+1.76579792
H	−3.17846257	−1.41161945	+4.11654122
H	−3.66281584	+0.26258820	+4.09124752

H	−0.95243247	−0.86270700	+3.83903375
H	−1.36334258	+0.82866222	+3.99042052
H	−1.74122116	−2.59369016	+2.64595482
H	−0.83612319	−2.51274508	+1.13572963
H	−2.49070967	−3.10168242	+1.13914454
H	−5.58742716	−1.36455701	+2.55146212
H	−4.40580985	−2.65321736	+2.39826276
H	−5.00117784	−1.82747373	+0.96027344
H	−1.79151578	+1.05728116	+1.65968074

51

* E = +6.912 kcal/mol ; (80) 312_047_001_010_321_062_178_309_335

C	+0.00000000	+0.00000000	+0.00000000
C	+0.00000000	+0.00000000	+1.33764711
C	+1.20343070	+0.00000000	+2.16982201
C	+2.45705194	−0.41441938	+1.93923672
C	+3.06921303	−1.08521389	+0.79373027
C	+2.63294861	−2.19130985	+0.17654905
C	+1.39893907	−2.94130051	+0.52036985
H	+1.60409632	−4.01080016	+0.56101396
C	+3.48463726	−2.71301384	−0.94291964
C	+3.88665670	−1.60203992	−1.90572794
C	+4.39045645	−0.35338409	−1.18771577
C	+4.39349641	−0.54606458	+0.32236890
C	−1.22572910	+0.07274180	−0.85432487
C	−2.50528207	+0.38044619	−0.08280907
C	−2.50054701	−0.35508321	+1.24141148
C	−1.31267356	+0.15401720	+2.05321194
C	−3.63882198	−0.02942222	+2.21112751
C	−3.06511858	−0.46211807	+3.57401762
C	−1.53125159	−0.47343246	+3.42363702
O	+5.68799666	−0.08194537	−1.67253400
H	+6.01578953	+0.71544188	−1.26177030
C	−2.42515045	−1.86028050	+1.00537904
C	−4.98766505	−0.63600912	+1.89477434
H	+3.73579125	+0.48931742	−1.42201642
H	+4.62950045	+0.39578557	+0.81579541
H	+5.19983404	−1.24593262	+0.55875422
H	+3.03216734	−1.34848041	−2.52940590
H	+4.67732928	−1.94126078	−2.57108253
H	+4.38598106	−3.16992464	−0.52400075

H	+2.96674955	−3.50323442	−1.48273068
H	+0.63709395	−2.79205169	−0.24851545
H	+0.97579588	−2.62360067	+1.46829974
H	+3.16769853	−0.18269969	+2.72611638
H	+1.04676622	+0.44609795	+3.14474090
H	+0.94513309	−0.06563290	−0.52178364
H	−1.33227155	−0.87889923	−1.38428079
H	−1.07343216	+0.81546383	−1.63832261
H	−2.56745220	+1.45246923	+0.11392883
H	−3.37556720	+0.11490296	−0.68423814
H	−3.74649943	+1.05878920	+2.21079205
H	−3.43651149	−1.45190401	+3.83850967
H	−3.39137039	+0.20820757	+4.36603286
H	−1.13828259	−1.48932621	+3.44592212
H	−1.03470249	+0.07360430	+4.22104544
H	−2.56817644	−2.42239817	+1.92611281
H	−1.46297772	−2.14949301	+0.59126465
H	−3.20198358	−2.17245534	+0.30885367
H	−5.73731263	−0.30953135	+2.61308243
H	−4.95096538	−1.72345299	+1.93330331
H	−5.33418138	−0.34600578	+0.90377487
H	−1.47838896	+1.23089795	+2.18125572

51

* E = +6.930 kcal/mol ; (81) 312_036_014_005_323_063_298_154_147

C	+0.00000000	+0.00000000	+0.00000000
C	+0.00000000	+0.00000000	+1.33951507
C	+1.28778550	+0.00000000	+2.04221920
C	+1.66140297	+0.54586698	+3.20808982
C	+0.94925730	+1.47482878	+4.07875527
C	+1.17212015	+1.55893788	+5.40056610
C	+2.05517976	+0.65750559	+6.19112145
H	+1.47906401	+0.16103247	+6.97309624
C	+0.41402484	+2.60637085	+6.15898866
C	+0.45973790	+3.97490475	+5.48095001
C	+0.42948707	+3.88555964	+3.95409245
C	+0.01146453	+2.49560438	+3.49881132
C	−1.21267509	−0.07255925	−0.86927730
C	−2.52773116	−0.03398314	−0.09749434
C	−2.38635109	−0.79457914	+1.20477286
C	−1.32303832	−0.08452270	+2.04870955

C	−3.57454306	−0.71261934	+2.16576724
C	−2.94493854	−1.06354846	+3.52589620
C	−1.43759567	−0.77096560	+3.40264610
O	−0.37086645	+4.88931686	+3.37703439
H	−1.27232044	+4.77453967	+3.67273587
C	−2.03240531	−2.25177443	+0.92929087
C	−4.78335282	−1.55177194	+1.81620528
H	+1.42328899	+4.07424962	+3.55664234
H	+0.01183728	+2.46545688	+2.41357557
H	−1.01634075	+2.31398438	+3.83512213
H	+1.34761797	+4.51644575	+5.79847566
H	−0.39141248	+4.57164579	+5.80905608
H	−0.62790953	+2.28194160	+6.24153327
H	+0.78191179	+2.68233549	+7.18024162
H	+2.83603177	+1.22680085	+6.69641586
H	+2.52272251	−0.11500285	+5.59106967
H	+2.67042425	+0.31235993	+3.52063441
H	+2.06106105	−0.55268031	+1.52287886
H	+0.95285556	+0.03104757	−0.51503171
H	−1.15157229	−0.99411169	−1.45532970
H	−1.18251152	+0.73263409	−1.60448053
H	−2.79275922	+1.00008760	+0.13057930
H	−3.33130002	−0.44111628	−0.71229960
H	−3.88395330	+0.33624075	+2.18737226
H	−3.11956596	−2.11431436	+3.75688916
H	−3.40765825	−0.49445056	+4.32950408
H	−0.85308167	−1.68961415	+3.41064542
H	−1.06219713	−0.15759343	+4.21624084
H	−2.05589945	−2.85161773	+1.83690878
H	−1.03975062	−2.34843820	+0.49771087
H	−2.74723659	−2.68649932	+0.23214680
H	−5.58834333	−1.38657012	+2.52988428
H	−4.54526617	−2.61400164	+1.83364229
H	−5.16712911	−1.30921922	+0.82616736
H	−1.70259186	+0.93273183	+2.17857299

51

* E = +6.993 kcal/mol ; (82) 342_047_299_313_033_016_184_059_032

C	+0.00000000	+0.00000000	+0.00000000
C	+0.00000000	+0.00000000	+1.33804900
C	+1.19435978	+0.00000000	+2.17866002

C	+2.38972275	+0.55312153	+1.94452821
C	+2.85062255	+1.32959203	+0.78569148
C	+2.29591534	+2.46957255	+0.35852227
C	+1.08375813	+3.08130443	+0.97356600
H	+0.22302872	+2.95617998	+0.31360447
C	+2.82667624	+3.20102716	−0.83653350
C	+4.23533114	+2.80361432	−1.22215993
C	+4.36067143	+1.30129550	−1.24414211
C	+4.07406306	+0.74104398	+0.13234149
C	−1.30262433	+0.01911260	−0.73065825
C	−2.32770014	−0.99043829	−0.18589029
C	−2.20724073	−1.19344774	+1.32616800
C	−1.36987221	−0.06116491	+1.94727744
C	−3.50596569	−1.02340242	+2.12108786
C	−3.02438079	−0.75802364	+3.55559165
C	−1.58164320	−0.23039199	+3.44359431
O	+3.43048433	+0.83303731	−2.19803851
H	+3.43501481	−0.12140907	−2.19879096
C	−1.56775524	−2.54681362	+1.61084001
C	−4.51049413	−2.14796252	+2.00846369
H	+5.37127982	+1.01545952	−1.54757976
H	+4.94649236	+0.89484106	+0.77337203
H	+3.95625838	−0.34337683	+0.06198023
H	+4.94939725	+3.19708202	−0.49890826
H	+4.49429234	+3.21101892	−2.19715882
H	+2.15443751	+3.01278522	−1.67709889
H	+2.78264471	+4.27420425	−0.64887303
H	+1.22683650	+4.15256185	+1.11225492
H	+0.83360204	+2.63418310	+1.92938107
H	+3.15941809	+0.35693188	+2.68494560
H	+1.09076215	−0.52203687	+3.12108061
H	+0.92379794	+0.02895707	−0.55958481
H	−1.15363096	−0.13605736	−1.79616457
H	−1.72898808	+1.02282073	−0.63197855
H	−3.32953230	−0.64690216	−0.44613443
H	−2.19054951	−1.94407050	−0.69297546
H	−3.97576835	−0.11100404	+1.74377555
H	−3.05261903	−1.68032684	+4.13555528
H	−3.67998733	−0.05576224	+4.06565745
H	−0.88398487	−0.95484047	+3.85902961

H	−1.42791406	+0.69832309	+3.98764792
H	−1.49622097	−2.75679408	+2.67708842
H	−0.56610443	−2.58806793	+1.18802766
H	−2.15158097	−3.34533055	+1.15679345
H	−5.42220300	−1.91111655	+2.55375391
H	−4.11387726	−3.07391543	+2.42149849
H	−4.78512400	−2.33338212	+0.97062366
H	−1.88176918	+0.86629732	+1.65888421

51

* E = +7.029 kcal/mol ; (83) 341_049_300_314_029_021_059_145_141

C	+0.00000000	+0.00000000	+0.00000000
C	+0.00000000	+0.00000000	+1.33835033
C	+1.26083780	+0.00000000	+2.08620762
C	+1.60001579	+0.63758066	+3.21342565
C	+0.86916232	+1.67161572	+3.95650347
C	+0.91928966	+1.74074938	+5.29367134
C	+1.56737383	+0.70444606	+6.15062307
H	+0.86712750	+0.36595407	+6.91530028
C	+0.27289908	+2.85577418	+6.05476015
C	−0.00926492	+4.07242806	+5.20084596
C	−0.70454472	+3.65899200	+3.92253263
C	+0.16799117	+2.71550029	+3.13112152
C	−1.29348329	−0.01928226	−0.74487777
C	−2.29502569	−1.05613105	−0.20055685
C	−2.10573024	−1.32444776	+1.29133070
C	−1.36281268	−0.14542170	+1.95623883
C	−3.39135380	−1.32532536	+2.12335549
C	−2.88883068	−1.12775481	+3.55684066
C	−1.54263592	−0.38904875	+3.44884498
O	−1.91208886	+2.97674307	+4.19764254
H	−2.48358030	+3.54404151	+4.71083928
C	−1.33356917	−2.62371785	+1.48530844
C	−4.30520104	−2.51686006	+1.94485626
H	−0.91759376	+4.53798481	+3.30908737
H	+0.91889630	+3.29213348	+2.58761270
H	−0.44794340	+2.24453884	+2.36913077
H	+0.92434223	+4.57166114	+4.93976882
H	−0.61314640	+4.79694450	+5.74934899
H	−0.65677460	+2.48459252	+6.49389421
H	+0.91042449	+3.12981158	+6.89687096

H	+2.42835706	+1.11623131	+6.67936077
H	+1.88773704	−0.16840119	+5.59283575
H	+2.58907339	+0.41417359	+3.59236634
H	+2.03128446	−0.62514951	+1.65089307
H	+0.93010663	+0.04851212	−0.55053107
H	−1.12785126	−0.17524218	−1.80801442
H	−1.74962811	+0.97092495	−0.65564751
H	−3.30642809	−0.69700218	−0.39395310
H	−2.18868237	−1.98712698	−0.75529014
H	−3.94287848	−0.42810459	+1.82855797
H	−2.76246518	−2.09673292	+4.04060007
H	−3.61129030	−0.57708926	+4.15539139
H	−0.72624656	−0.99625088	+3.83623857
H	−1.54188384	+0.54054149	+4.00697204
H	−1.16198812	−2.84710017	+2.53694479
H	−0.36674386	−2.57090291	+0.99027965
H	−1.88039948	−3.45825071	+1.05012159
H	−5.22088984	−2.39216393	+2.52002369
H	−3.82898016	−3.43471926	+2.28539138
H	−4.58682988	−2.65130015	+0.90083371
H	−1.94783183	+0.74621793	+1.69661123

51

* E = +7.063 kcal/mol ; (84) 345_047_299_314_033_016_059_302_350

C	+0.00000000	+0.00000000	+0.00000000
C	+0.00000000	+0.00000000	+1.34058223
C	+1.16588176	+0.00000000	+2.21648890
C	+2.47479251	−0.16480052	+1.97295270
C	+3.21141239	−0.47363450	+0.74455720
C	+3.01344799	−1.56032725	−0.01266584
C	+1.99413503	−2.60168995	+0.28730054
H	+2.42558630	−3.59543484	+0.16682190
C	+3.84557899	−1.83518445	−1.22756995
C	+4.63304516	−0.63563367	−1.70813483
C	+5.33792168	+0.02326071	−0.54209476
C	+4.32274224	+0.50346989	+0.46583931
C	−1.29252745	+0.14864174	−0.73426789
C	−2.41944635	−0.74610927	−0.19180702
C	−2.31669122	−0.96497571	+1.31904685
C	−1.37505688	+0.08102652	+1.94041994
C	−3.59256851	−0.67595988	+2.11640486

C	−3.08408798	−0.47338857	+3.55110933
C	−1.60109664	−0.07406788	+3.43633041
O	+6.18502922	−0.88814437	+0.12438337
H	+6.83596942	−1.21949947	−0.49053162
C	−1.81140966	−2.37521990	+1.59691897
C	−4.70299677	−1.69504872	+1.99512394
H	+5.92279423	+0.87851214	−0.89019966
H	+3.89687856	+1.44898694	+0.12490327
H	+4.84520662	+0.72633352	+1.39667538
H	+3.96572244	+0.09564469	−2.16561099
H	+5.35091543	−0.93054251	−2.47489149
H	+4.52541177	−2.65832948	−0.99517031
H	+3.19816023	−2.20141809	−2.02624701
H	+1.15417008	−2.53198748	−0.40802716
H	+1.59802662	−2.50928956	+1.29319991
H	+3.12544795	+0.01298192	+2.82339590
H	+0.92819008	+0.22769541	+3.24716242
H	+0.92261666	−0.04094482	−0.55777647
H	−1.15607976	−0.02578431	−1.79890958
H	−1.61285735	+1.19163242	−0.64227457
H	−3.37873969	−0.29488485	−0.44797313
H	−2.38805132	−1.70756123	−0.70192952
H	−3.97066584	+0.28199930	+1.74882971
H	−3.19052107	−1.39752290	+4.11890732
H	−3.67243571	+0.27697314	+4.07453049
H	−0.96204815	−0.85682532	+3.84209344
H	−1.37033946	+0.83603031	+3.98534652
H	−1.74736032	−2.59190522	+2.66203131
H	−0.82439762	−2.51503741	+1.16266035
H	−2.47719523	−3.11093055	+1.14940368
H	−5.58744488	−1.37307088	+2.54168573
H	−4.40071696	−2.65864134	+2.40166982
H	−4.99362600	−1.84498070	+0.95587453
H	−1.79565749	+1.05291183	+1.65141509

51

* E = +7.097 kcal/mol ; (85) 342_047_299_314_032_017_059_061_037

C	+0.00000000	+0.00000000	+0.00000000
C	+0.00000000	+0.00000000	+1.33752338
C	+1.20324952	+0.00000000	+2.16625942
C	+2.35883836	+0.63129641	+1.93758801

C	+2.71882958	+1.49572240	+0.80418226
C	+2.08915982	+2.63237127	+0.48991547
C	+0.89248395	+3.14612959	+1.21651629
H	−0.00545954	+3.00364054	+0.61151776
C	+2.50068964	+3.45468680	−0.69271831
C	+3.88814543	+3.13247419	−1.20493501
C	+4.06801250	+1.63515175	−1.32380371
C	+3.90441246	+0.98634864	+0.02794947
C	−1.30450070	+0.03079962	−0.72762195
C	−2.34419675	−0.96262259	−0.17856292
C	−2.21102605	−1.18200385	+1.32981211
C	−1.36599103	−0.05762225	+1.95390385
C	−3.50189083	−1.01881276	+2.13876132
C	−3.00598758	−0.77104799	+3.57139646
C	−1.56601597	−0.23781679	+3.45075147
O	+3.08595479	+1.06623658	−2.16639796
H	+3.14173649	+1.46826842	−3.03066348
C	−1.57041474	−2.53906260	+1.59382788
C	−4.50986573	−2.13999812	+2.02301893
H	+5.06218752	+1.40344714	−1.71442405
H	+4.81752802	+1.12670923	+0.61126493
H	+3.80101854	−0.08807843	−0.12263373
H	+4.64151899	+3.51558459	−0.51601503
H	+4.06419229	+3.61786667	−2.16618920
H	+1.76664742	+3.29502061	−1.48749567
H	+2.43656590	+4.51281272	−0.43704514
H	+0.98661335	+4.21571714	+1.40288832
H	+0.73477955	+2.63911682	+2.16216361
H	+3.16429817	+0.44223747	+2.64041608
H	+1.14383985	−0.58341643	+3.07638727
H	+0.92249713	+0.01909505	−0.56315770
H	−1.15871653	−0.13055175	−1.79264936
H	−1.71747797	+1.04061110	−0.63166662
H	−3.34164452	−0.59531184	−0.42312167
H	−2.23396310	−1.91450458	−0.69538836
H	−3.97331618	−0.10103231	+1.77671032
H	−3.02515912	−1.70150003	+4.13866648
H	−3.65836217	−0.07808115	+4.09816898
H	−0.86178457	−0.96190307	+3.85559865
H	−1.41091544	+0.68793283	+3.99955573

H	−1.48369147	−2.75778736	+2.65726853
H	−0.57529023	−2.57861199	+1.15594049
H	−2.16273286	−3.33251353	+1.14181932
H	−5.41581743	−1.90782334	+2.57990684
H	−4.11100021	−3.07136575	+2.42143163
H	−4.79476501	−2.31295919	+0.98579249
H	−1.87569005	+0.87460693	+1.67586111

51

* E = +7.131 kcal/mol ; (86) 017_313_062_314_032_017_297_299_350

C	+0.00000000	+0.00000000	+0.00000000
C	+0.00000000	+0.00000000	+1.34026847
C	+1.16887990	+0.00000000	+2.21313896
C	+2.47356302	−0.17488586	+1.96021764
C	+3.19325717	−0.47923064	+0.71852853
C	+3.01646039	−1.58291192	−0.02014648
C	+1.98494688	−2.61305092	+0.28206561
H	+2.41513909	−3.61248822	+0.22707448
C	+3.84444682	−1.85792042	−1.24137288
C	+5.10849338	−1.02660022	−1.31964013
C	+4.79489959	+0.41733390	−1.01386890
C	+4.25952135	+0.53624886	+0.39521254
C	−1.29093918	+0.15252852	−0.73627827
C	−2.43031103	−0.72457321	−0.18978315
C	−2.32110013	−0.95512534	+1.31836591
C	−1.37331575	+0.08346841	+1.94294225
C	−3.59213920	−0.66829336	+2.12469499
C	−3.07567836	−0.48015256	+3.55826187
C	−1.59377235	−0.07904777	+3.43907083
O	+5.89340014	+1.26930709	−1.22630121
H	+6.59693032	+1.01936656	−0.62957364
C	−1.81923192	−2.36931327	+1.58221906
C	−4.70745112	−1.68162908	+2.00019625
H	+4.03437466	+0.76610452	−1.71263580
H	+3.87076047	+1.54170385	+0.54984758
H	+5.08933669	+0.42289224	+1.10233269
H	+5.56771750	−1.10491712	−2.30287627
H	+5.83444490	−1.39231298	−0.58873751
H	+4.09186770	−2.91937465	−1.26927023
H	+3.22907772	−1.68244610	−2.12936232
H	+1.17630758	−2.57251328	−0.45174743

H	+1.54436402	−2.47257536	+1.26306862
H	+3.13531067	+0.00526914	+2.80213762
H	+0.93754495	+0.23517915	+3.24355254
H	+0.92234368	−0.03895857	−0.55868849
H	−1.15414513	−0.03066730	−1.79929490
H	−1.59873355	+1.19989090	−0.65307505
H	−3.38233193	−0.25276118	−0.43527601
H	−2.42264785	−1.68320381	−0.70601318
H	−3.96806620	+0.29440545	+1.76749286
H	−3.17834649	−1.41035072	+4.11677612
H	−3.66146493	+0.26423548	+4.09288338
H	−0.95206955	−0.86279612	+3.83891874
H	−1.36181362	+0.82887988	+3.99118404
H	−1.74294402	−2.59263384	+2.64519875
H	−0.83878955	−2.51220737	+1.13435578
H	−2.49396223	−3.09917498	+1.13869192
H	−5.58821026	−1.35974473	+2.55266306
H	−4.40802921	−2.64957791	+2.39842344
H	−5.00297395	−1.82240783	+0.96107622
H	−1.79035448	+1.05882003	+1.66062542

51

* E = +7.229 kcal/mol ; (87) 314_059_340_010_321_062_067_309_336

C	+0.00000000	+0.00000000	+0.00000000
C	+0.00000000	+0.00000000	+1.33762043
C	+1.20319672	+0.00000000	+2.16996208
C	+2.45808560	−0.40980966	+1.93810202
C	+3.07545957	−1.07382943	+0.79075095
C	+2.64187697	−2.18375278	+0.17859467
C	+1.40492790	−2.93098018	+0.51821987
H	+1.60848009	−4.00091001	+0.55769851
C	+3.47347591	−2.69077759	−0.96232393
C	+3.83069896	−1.52860352	−1.87546567
C	+4.61051434	−0.44726957	−1.13423063
C	+4.42407204	−0.54841341	+0.37375149
C	−1.22567350	+0.06917199	−0.85480403
C	−2.50653246	+0.37267333	−0.08378024
C	−2.49955249	−0.36155818	+1.24113866
C	−1.31337474	+0.15205476	+2.05251413
C	−3.63923273	−0.03791419	+2.20973516
C	−3.06496747	−0.46648407	+3.57368161

C	−1.53094522	−0.47269753	+3.42435833
O	+6.00362810	−0.56072916	−1.34401736
H	+6.19996022	−0.37648760	−2.25978496
C	−2.41972445	−1.86674616	+1.00691555
C	−4.98588737	−0.64959355	+1.89386830
H	+4.27672417	+0.53515513	−1.47458345
H	+4.63537139	+0.41306431	+0.83320936
H	+5.20145322	−1.23537626	+0.71828592
H	+2.90583886	−1.10644151	−2.26752461
H	+4.41577719	−1.86554203	−2.73107141
H	+4.39124331	−3.15432503	−0.58892301
H	+2.93697960	−3.46051928	−1.51389258
H	+0.64404649	−2.77991249	−0.25155908
H	+0.98073398	−2.61430082	+1.46593517
H	+3.16710817	−0.17803845	+2.72637197
H	+1.04528160	+0.44250168	+3.14626926
H	+0.94584647	−0.06314850	−0.52056789
H	−1.32949383	−0.88278781	−1.38486505
H	−1.07589351	+0.81267492	−1.63864453
H	−2.57275919	+1.44459553	+0.11213705
H	−3.37575169	+0.10337435	−0.68515957
H	−3.75042041	+1.04994171	+2.20751743
H	−3.43308894	−1.45721164	+3.83927908
H	−3.39417887	+0.20382206	+4.36449235
H	−1.13415226	−1.48700207	+3.44994941
H	−1.03694544	+0.07839186	+4.22052263
H	−2.55835606	−2.42788035	+1.92894901
H	−1.45769519	−2.15339536	+0.59063147
H	−3.19760700	−2.18242258	+0.31310169
H	−5.73676228	−0.32518622	+2.61183984
H	−4.94502321	−1.73686206	+1.93347509
H	−5.33342958	−0.36190273	+0.90253630
H	−1.48177565	+1.22878096	+2.17814778

51

* E = +7.317 kcal/mol ; (88) 051_310_004_010_321_062_177_308_336

C	+0.00000000	+0.00000000	+0.00000000
C	+0.00000000	+0.00000000	+1.33771612
C	+1.20343081	+0.00000000	+2.17000457
C	+2.45939769	−0.40217435	+1.93132086
C	+3.07192760	−1.05128900	+0.77412962

C	+2.65122712	−2.16288224	+0.15735949
C	+1.43065085	−2.93236148	+0.50643089
H	+1.65231778	−3.99823996	+0.55263928
C	+3.51232204	−2.67097489	−0.95999951
C	+4.96272486	−2.78527986	−0.50539799
C	+5.46981872	−1.50882014	+0.15731903
C	+4.36499752	−0.46518489	+0.27899307
C	−1.22573132	+0.06525320	−0.85483122
C	−2.50767095	+0.36370501	−0.08393205
C	−2.49807917	−0.37120589	+1.24052429
C	−1.31412608	+0.14702758	+2.05248331
C	−3.63941548	−0.05272762	+2.20858315
C	−3.06383443	−0.47904864	+3.57287802
C	−1.52979268	−0.47965567	+3.42379390
O	+5.96384816	−1.88218556	+1.42625089
H	+6.34039570	−1.11686034	+1.85550691
C	−2.41158549	−1.87586370	+1.00516048
C	−4.98311114	−0.67030734	+1.89157193
H	+6.28494932	−1.07905981	−0.43049054
H	+4.20971979	−0.01911788	−0.70766222
H	+4.69928204	+0.34814262	+0.92321773
H	+5.60155786	−3.06641255	−1.33981415
H	+5.04009849	−3.57445079	+0.23909564
H	+3.15712490	−3.63724104	−1.31183245
H	+3.45114447	−1.99076034	−1.81536571
H	+0.66377923	−2.79821942	−0.26037627
H	+1.00562215	−2.61673875	+1.45398057
H	+3.17219618	−0.18192884	+2.71945678
H	+1.04375596	+0.42930845	+3.15182406
H	+0.94534570	−0.06122505	−0.52162221
H	−1.32554977	−0.88669574	−1.38552996
H	−1.07819776	+0.80961318	−1.63829008
H	−2.57785139	+1.43530624	+0.11246081
H	−3.37587184	+0.09148280	−0.68551433
H	−3.75528753	+1.03467641	+2.20649662
H	−3.42826838	−1.47114990	+3.83847073
H	−3.39575303	+0.19011435	+4.36355198
H	−1.12964007	−1.49264408	+3.44837167
H	−1.03787483	+0.07234619	+4.22064654
H	−2.55077547	−2.43864685	+1.92609072

H	−1.44709362	−2.15782418	+0.59143260
H	−3.18606088	−2.19393381	+0.30862422
H	−5.73624661	−0.34922361	+2.60866823
H	−4.93733216	−1.75738386	+1.93118297
H	−5.33069317	−0.38429365	+0.89977162
H	−1.48730198	+1.22293992	+2.17867247

51

* E = +7.321 kcal/mol ; (89) 045_299_027_006_322_063_310_152_149

C	+0.00000000	+0.00000000	+0.00000000
C	+0.00000000	+0.00000000	+1.33975566
C	+1.28489401	+0.00000000	+2.04346311
C	+1.66025764	+0.51492592	+3.22289808
C	+0.97159856	+1.41633751	+4.14912456
C	+1.21711725	+1.40743218	+5.46854341
C	+2.07262210	+0.41849025	+6.18194898
H	+1.48884087	−0.11198839	+6.93538718
C	+0.55171169	+2.45522480	+6.31043653
C	−0.90670157	+2.57647580	+5.91047834
C	−1.05308319	+2.99234442	+4.45646442
C	+0.15274151	+2.56005348	+3.60813499
C	−1.21593820	−0.04586117	−0.86671378
C	−2.52686653	+0.04034238	−0.09213246
C	−2.40973548	−0.72961363	+1.20711911
C	−1.32073645	−0.05927187	+2.04925350
C	−3.58995481	−0.60315144	+2.17366815
C	−2.96782328	−0.98681470	+3.52899665
C	−1.44919491	−0.75700840	+3.39624603
O	−2.28552005	+2.55319234	+3.93026862
H	−2.39562796	+1.62528778	+4.13442942
C	−2.10816225	−2.19738873	+0.92548695
C	−4.83300321	−1.39165779	+1.82751690
H	−1.11893667	+4.07657157	+4.39803875
H	+0.82920852	+3.41643846	+3.53898578
H	−0.17773360	+2.37766354	+2.59091466
H	−1.45278150	+3.26279469	+6.55396181
H	−1.36710130	+1.59279123	+6.03375621
H	+0.63532927	+2.20466583	+7.36615898
H	+1.05416775	+3.41981376	+6.18202961
H	+2.87949873	+0.92280888	+6.71541553
H	+2.50649446	−0.32460548	+5.52242178

H +2.67897911 +0.28419825 +3.50595230
H +2.06540426 −0.52471426 +1.50586741
H +0.95295144 +0.01851859 −0.51554125
H −1.18439674 −0.97325575 −1.44590631
H −1.16292611 +0.75252291 −1.60801836
H −2.75205712 +1.08248208 +0.14055833
H −3.34621781 −0.33520303 −0.70616733
H −3.85432445 +0.45786107 +2.20218267
H −3.18330025 −2.02997979 +3.75915505
H −3.40992493 −0.40940685 +4.34126772
H −0.90602256 −1.70053215 +3.38844765
H −1.03051341 −0.17639040 +4.21529047
H −2.16029654 −2.80210418 +1.82882235
H −1.11579961 −2.32690428 +0.50096337
H −2.83278026 −2.60225476 +0.22040876
H −5.62678482 −1.19877503 +2.54668554
H −4.63704969 −2.46264810 +1.83619130
H −5.21219311 −1.12675471 +0.84162068
H −1.67046177 +0.96604342 +2.18710074
51
* E = +7.490 kcal/mol ; (90) 041_298_034_006_322_063_055_147_148
C +0.00000000 +0.00000000 +0.00000000
C +0.00000000 +0.00000000 +1.33996372
C +1.29141175 +0.00000000 +2.03489605
C +1.67287864 +0.52984313 +3.20489112
C +0.96436230 +1.43285821 +4.11702314
C +1.12492857 +1.37900262 +5.44633074
C +1.91814486 +0.34731592 +6.17156412
H +1.27216142 −0.23000792 +6.83467406
C +0.40757563 +2.38969872 +6.29145557
C −0.99862297 +2.58421447 +5.75771445
C −0.97370465 +3.12295984 +4.34020976
C +0.21024332 +2.59636593 +3.52730320
C −1.21671923 −0.03331735 −0.86613301
C −2.52374519 +0.07533703 −0.08864933
C −2.41675150 −0.69923072 +1.20865269
C −1.31886036 −0.04934324 +2.05532637
C −3.59485247 −0.55917613 +2.17534585
C −2.97784396 −0.94254888 +3.53333371
C −1.45590220 −0.75416469 +3.39646655

O	−2.15610794	+2.77798430	+3.64452463
H	−2.91214200	+3.08428006	+4.14124638
C	−2.13761546	−2.17083186	+0.92290743
C	−4.84537271	−1.33624554	+1.82869814
H	−0.89811777	+4.21269761	+4.37712233
H	+0.93217098	+3.40722391	+3.40936171
H	−0.13470461	+2.37592410	+2.52255741
H	−1.58141069	+3.24392771	+6.40114550
H	−1.49921226	+1.61555224	+5.73723523
H	+0.37560125	+2.05852229	+7.32781907
H	+0.94526526	+3.34348819	+6.29174297
H	+2.67207970	+0.81672350	+6.80482069
H	+2.41246813	−0.35237632	+5.50699873
H	+2.69241075	+0.30742919	+3.49264958
H	+2.06710633	−0.53300624	+1.49825910
H	+0.95297268	+0.01127627	−0.51610804
H	−1.19991399	−0.96419225	−1.44054604
H	−1.15284831	+0.76071308	−1.61146650
H	−2.72768640	+1.12109010	+0.14759234
H	−3.35161507	−0.28339226	−0.70183562
H	−3.84634884	+0.50419719	+2.20233925
H	−3.22504353	−1.97523743	+3.78083319
H	−3.38787806	−0.32666321	+4.33176340
H	−0.93489351	−1.71075051	+3.38429729
H	−1.03572051	−0.17412905	+4.21126270
H	−2.20628501	−2.77584908	+1.82491047
H	−1.14427059	−2.31590326	+0.50525830
H	−2.86383458	−2.56222544	+0.21149553
H	−5.63820305	−1.13479864	+2.54701000
H	−4.66076686	−2.40929112	+1.84022940
H	−5.22171633	−1.07072887	+0.84149268
H	−1.65856132	+0.97394522	+2.22165048

51

* E = +7.653 kcal/mol ; (91) 042_299_032_007_322_063_166_146_149

C	+0.00000000	+0.00000000	+0.00000000
C	+0.00000000	+0.00000000	+1.34020117
C	+1.29031359	+0.00000000	+2.03621739
C	+1.66688822	+0.52010264	+3.21203728
C	+0.95552199	+1.40780864	+4.13732483
C	+1.10686479	+1.32579005	+5.46622533

C	+1.88798984	+0.27338020	+6.17410098
H	+1.23268715	−0.31330107	+6.81950117
C	+0.39327105	+2.32715659	+6.32432162
C	−1.01370433	+2.53725135	+5.80046614
C	−0.99924307	+3.08322570	+4.39075021
C	+0.21040001	+2.59315066	+3.57879346
C	−1.21659570	−0.03134084	−0.86654031
C	−2.52176069	+0.09471818	−0.08930714
C	−2.42544204	−0.67850718	+1.20947448
C	−1.31804700	−0.04630155	+2.05650770
C	−3.60211101	−0.52277694	+2.17544886
C	−2.99026356	−0.89809211	+3.53869073
C	−1.46474899	−0.75318274	+3.39482492
O	−2.23284782	+2.70951304	+3.81090409
H	−2.36960259	+3.21032980	+3.00980095
C	−2.16489732	−2.15375056	+0.92447364
C	−4.85873843	−1.29180578	+1.83286691
H	−0.95089653	+4.17413251	+4.43520633
H	+0.93138374	+3.41289954	+3.52002668
H	−0.09356665	+2.40951244	+2.55141815
H	−1.59432602	+3.19370097	+6.44476138
H	−1.52686299	+1.57598430	+5.77056452
H	+0.36191749	+1.98308478	+7.35638900
H	+0.93760249	+3.27750294	+6.33601061
H	+2.63954919	+0.72427962	+6.82331941
H	+2.38274856	−0.41523927	+5.49823189
H	+2.68752291	+0.29968286	+3.49792285
H	+2.06957114	−0.52258475	+1.49461973
H	+0.95316814	+0.00928007	−0.51568624
H	−1.20899053	−0.96755274	−1.43221336
H	−1.14625562	+0.75482064	−1.61963600
H	−2.71305384	+1.14426905	+0.14398516
H	−3.35519166	−0.25277577	−0.70121325
H	−3.84847738	+0.54212592	+2.19726110
H	−3.26556784	−1.91755647	+3.80975610
H	−3.37630390	−0.24785449	+4.32083040
H	−0.96892259	−1.72282803	+3.37294933
H	−1.02581360	−0.18870739	+4.21011122
H	−2.25019235	−2.75738997	+1.82576520
H	−1.17028822	−2.31298753	+0.51478694

H	−2.89134508	−2.53415906	+0.20742878
H	−5.64957879	−1.07715234	+2.54921266
H	−4.68368680	−2.36627504	+1.85315508
H	−5.23226229	−1.03089543	+0.84323507
H	−1.64674186	+0.97868047	+2.23335491

51

* E = +7.667 kcal/mol ; (92) 051_310_003_010_321_062_055_308_336

C	+0.00000000	+0.00000000	+0.00000000
C	+0.00000000	+0.00000000	+1.33772489
C	+1.20329642	+0.00000000	+2.17043885
C	+2.45875025	−0.40347204	+1.93205410
C	+3.06872397	−1.05553217	+0.77522558
C	+2.64800174	−2.16984322	+0.16363238
C	+1.42504237	−2.93633122	+0.51204591
H	+1.64210256	−4.00325530	+0.56034619
C	+3.51415765	−2.68583950	−0.94647042
C	+4.96285680	−2.79792736	−0.48155242
C	+5.46387374	−1.51323678	+0.18695140
C	+4.36432360	−0.47059553	+0.28365052
C	−1.22558986	+0.06601778	−0.85517627
C	−2.50716866	+0.36660193	−0.08447356
C	−2.49887264	−0.36818969	+1.24003336
C	−1.31419277	+0.14806743	+2.05212118
C	−3.63982777	−0.04805541	+2.20798770
C	−3.06498445	−0.47530581	+3.57232237
C	−1.53094521	−0.47863559	+3.42322203
O	+5.90229021	−1.74207692	+1.50951466
H	+6.56422999	−2.42948420	+1.50986564
C	−2.41477330	−1.87298998	+1.00465928
C	−4.98447518	−0.66355858	+1.89090027
H	+6.28877265	−1.08938237	−0.39116234
H	+4.21584325	−0.03522858	−0.70765233
H	+4.70733868	+0.33276310	+0.93074917
H	+5.60496197	−3.07562856	−1.31612572
H	+5.02208844	−3.60073541	+0.25257078
H	+3.15944871	−3.65296945	−1.29685759
H	+3.46446215	−2.00969271	−1.80528782
H	+0.65824681	−2.80058938	−0.25460427
H	+1.00070770	−2.61744120	+1.45889003
H	+3.17633750	−0.18391631	+2.71531965

H	+1.04448570	+0.43234427	+3.15105601
H	+0.94554507	−0.06062584	−0.52134355
H	−1.32701201	−0.88594619	−1.38564557
H	−1.07725589	+0.81002178	−1.63883973
H	−2.57543824	+1.43832094	+0.11192512
H	−3.37586270	+0.09586422	−0.68609004
H	−3.75404952	+1.03950942	+2.20592229
H	−3.43133256	−1.46671693	+3.83800023
H	−3.39568360	+0.19456122	+4.36292801
H	−1.13256217	−1.49233828	+3.44739082
H	−1.03778059	+0.07214591	+4.22007856
H	−2.55478151	−2.43544446	+1.92568998
H	−1.45060014	−2.15613728	+0.59098650
H	−3.18974852	−2.18990342	+0.30810871
H	−5.73717152	−0.34133563	+2.60797038
H	−4.94034439	−1.75072276	+1.93052857
H	−5.33156966	−0.37703755	+0.89906052
H	−1.48582723	+1.22417545	+2.17876553

51

* E = +7.683 kcal/mol ; (93) 312_052_354_010_321_062_293_309_335

C	+0.00000000	+0.00000000	+0.00000000
C	+0.00000000	+0.00000000	+1.33759833
C	+1.20378260	+0.00000000	+2.16902416
C	+2.45642483	−0.41753301	+1.93931776
C	+3.06619704	−1.09543517	+0.79662958
C	+2.62509574	−2.20341961	+0.18584729
C	+1.38595012	−2.94472149	+0.52958378
H	+1.58355795	−4.01565747	+0.57080648
C	+3.46725850	−2.72570444	−0.94046008
C	+3.84549449	−1.59914922	−1.89347959
C	+4.46248817	−0.39596292	−1.17130058
C	+4.40178685	−0.57134078	+0.34082842
C	−1.22557932	+0.07506392	−0.85430478
C	−2.50425122	+0.38626813	−0.08267743
C	−2.50145334	−0.34881063	+1.24181326
C	−1.31212161	+0.15713929	+2.05332964
C	−3.63862434	−0.01949757	+2.21159151
C	−3.06597582	−0.45325181	+3.57461721
C	−1.53213786	−0.46906236	+3.42406037
O	+5.78221492	−0.13141473	−1.58917173

H	+6.34528303	−0.85250392	−1.31245213
C	−2.43067635	−1.85429582	+1.00633514
C	−4.98922126	−0.62244525	+1.89579651
H	+3.91691965	+0.50526278	−1.43776084
H	+4.65297904	+0.36969395	+0.82212862
H	+5.18439271	−1.28720824	+0.62058690
H	+2.94964987	−1.28624781	−2.42682026
H	+4.54841779	−1.94494548	−2.64972719
H	+4.37354696	−3.18711976	−0.53406903
H	+2.94345468	−3.51296512	−1.47903680
H	+0.62455629	−2.79033534	−0.23876555
H	+0.96535829	−2.62331512	+1.47737943
H	+3.16854638	−0.18207595	+2.72351278
H	+1.04920903	+0.45168337	+3.14168573
H	+0.94529778	−0.06476423	−0.52147793
H	−1.33468696	−0.87652130	−1.38390178
H	−1.07148073	+0.81722046	−1.63843688
H	−2.56348048	+1.45849809	+0.11362684
H	−3.37526796	+0.12286196	−0.68397733
H	−3.74321627	+1.06898627	+2.21078937
H	−3.44022432	−1.44183229	+3.83961064
H	−3.39013438	+0.21839291	+4.36635578
H	−1.14205386	−1.48607809	+3.44689518
H	−1.03385164	+0.07702353	+4.22102352
H	−2.57423550	−2.41562305	+1.92748472
H	−1.46986488	−2.14642509	+0.59109688
H	−3.20929189	−2.16444918	+0.31089501
H	−5.73771304	−0.29370813	+2.61426792
H	−4.95549283	−1.70998543	+1.93461700
H	−5.33527498	−0.33171961	+0.90485144
H	−1.47472622	+1.23450497	+2.18103819

51

* E = +10.034 kcal/mol ; (94) 045_327_343_314_037_012_289_077_332

C	+0.00000000	+0.00000000	+0.00000000
C	+0.00000000	+0.00000000	+1.34198135
C	+1.15452359	+0.00000000	+2.23270412
C	+2.39263869	−0.47571314	+2.05512965
C	+2.98645359	−1.13651323	+0.88671945
C	+3.38971452	−0.52285551	−0.23159827
C	+3.28403530	+0.94113451	−0.48228137

H	+2.75419422	+1.14201230	−1.41442907
C	+4.08587532	−1.36266308	−1.26084077
C	+3.39094626	−2.70080832	−1.52037904
C	+2.71382038	−3.28233807	−0.27736873
C	+3.25294404	−2.61799838	+0.98153920
C	−1.30659469	+0.00942910	−0.72604935
C	−2.31423018	−1.00733573	−0.16919392
C	−2.23420219	−1.13629296	+1.35575056
C	−1.37922709	+0.00467349	+1.93478363
C	−3.54129777	−0.88392762	+2.11526021
C	−3.07265480	−0.57060480	+3.54490196
C	−1.61209080	−0.09319072	+3.43286665
O	+1.31352996	−3.14928299	−0.33611282
H	+1.09079413	−2.22154730	−0.22072502
C	−1.65051436	−2.49363225	+1.72805211
C	−4.58131830	−1.97861623	+2.03689437
H	+2.88586741	−4.35455602	−0.22836996
H	+4.32634140	−2.81395253	+1.06091439
H	+2.77108606	−3.04899463	+1.85471523
H	+4.11717111	−3.40732839	−1.91850893
H	+2.62043637	−2.58605007	−2.27953745
H	+4.19749283	−0.81032285	−2.19189312
H	+5.10203907	−1.55512081	−0.90392114
H	+4.27778508	+1.37699307	−0.59288440
H	+2.76952196	+1.45440187	+0.32459490
H	+3.04242116	−0.43976075	+2.92403451
H	+0.94444767	+0.38874512	+3.22071821
H	+0.91438756	+0.05675149	−0.56663307
H	−1.15836007	−0.15556960	−1.79049012
H	−1.73867459	+1.01133022	−0.63552863
H	−3.31845698	−0.71462758	−0.47800232
H	−2.12168557	−1.97647458	−0.62589150
H	−3.97376268	+0.02536053	+1.68844576
H	−3.13813219	−1.46527461	+4.16353686
H	−3.71226076	+0.17388669	+4.01373924
H	−0.93456180	−0.81800676	+3.88117886
H	−1.44276848	+0.85389324	+3.94058140
H	−1.62499184	−2.65131941	+2.80512171
H	−0.63943915	−2.60665686	+1.34471853
H	−2.24916841	−3.29166149	+1.29314010

H	−5.49334740	−1.68569069	+2.55372296
H	−4.22319819	−2.89665951	+2.49934782
H	−4.84479639	−2.20385468	+1.00410143
H	−1.86736381	+0.92694245	+1.59582722

51

* E = +10.725 kcal/mol ; (95) 048_310_003_314_032_017_179_051_041

C	+0.00000000	+0.00000000	+0.00000000
C	+0.00000000	+0.00000000	+1.33653674
C	+1.20744674	+0.00000000	+2.15948333
C	+2.33532314	+0.69515949	+1.97330843
C	+2.64489031	+1.69087058	+0.94649280
C	+1.90668632	+2.76105990	+0.62933998
C	+0.61352395	+3.13822236	+1.25898027
H	−0.22062115	+2.90147910	+0.59466134
C	+2.43057115	+3.62322554	−0.48032890
C	+2.82388739	+2.77056265	−1.68147986
C	+3.72292117	+1.59776993	−1.30049720
C	+3.94461148	+1.54190337	+0.20589203
C	−1.30600207	+0.03087041	−0.72543334
C	−2.35052881	−0.95502309	−0.17074900
C	−2.21161748	−1.17779507	+1.33660686
C	−1.36275210	−0.05577368	+1.95868625
C	−3.49815817	−1.01491481	+2.15253973
C	−2.99418932	−0.77201553	+3.58331001
C	−1.55562490	−0.23614664	+3.45633195
O	+3.08049230	+0.42755906	−1.76570765
H	+3.62188407	−0.33207965	−1.56032177
C	−1.57052245	−2.53562874	+1.59571001
C	−4.50900347	−2.13374679	+2.03879866
H	+4.69372454	+1.69044851	−1.79361601
H	+4.63044228	+2.35119299	+0.47357364
H	+4.44824149	+0.61172771	+0.46770979
H	+3.29476372	+3.38486295	−2.44598405
H	+1.92552551	+2.34378633	−2.12265158
H	+1.68706939	+4.35989519	−0.77813742
H	+3.30049102	+4.19008462	−0.13322981
H	+0.58075508	+4.21072725	+1.44910699
H	+0.44789452	+2.61017058	+2.19324271
H	+3.15142604	+0.48148779	+2.65601069
H	+1.18316292	−0.65409640	+3.02266231

H	+0.92504592	+0.02505198	−0.55940776
H	−1.16270693	−0.13580636	−1.79000683
H	−1.71523713	+1.04302859	−0.63434335
H	−3.34631800	−0.57929711	−0.40897913
H	−2.25150356	−1.90716763	−0.68939516
H	−3.96966708	−0.09521147	+1.79554336
H	−3.00895063	−1.70470507	+4.14701335
H	−3.64453269	−0.08212567	+4.11657421
H	−0.84676006	−0.95746376	+3.85822225
H	−1.40010604	+0.69045604	+4.00366056
H	−1.47877799	−2.75543843	+2.65853108
H	−0.57756895	−2.57454294	+1.15278513
H	−2.16486513	−3.32850071	+1.14542780
H	−5.41163606	−1.90127772	+2.60090397
H	−4.11021794	−3.06717657	+2.43247147
H	−4.79953840	−2.30300549	+1.00253804
H	−1.87093357	+0.87856746	+1.68300413

51

* E = +10.783 kcal/mol ; (96) 310_041_008_314_031_018_177_051_047

C	+0.00000000	+0.00000000	+0.00000000
C	+0.00000000	+0.00000000	+1.33532308
C	+1.20959578	+0.00000000	+2.15623352
C	+2.29191629	+0.77250327	+2.01371658
C	+2.52519147	+1.86319110	+1.06604463
C	+1.71002359	+2.90107616	+0.83737027
C	+0.39254578	+3.12486413	+1.49113502
H	−0.42268767	+2.87012807	+0.80993351
C	+2.16282351	+3.89045024	−0.19545335
C	+3.60178102	+4.34614013	+0.03068424
C	+4.52872341	+3.20412356	+0.43157761
C	+3.83057758	+1.85680095	+0.32135632
C	−1.30253219	+0.04015626	−0.73124037
C	−2.35571076	−0.93772512	−0.17680962
C	−2.21623446	−1.16729757	+1.32951953
C	−1.36364057	−0.05047468	+1.95443465
C	−3.50080628	−1.00188619	+2.14857512
C	−2.99328854	−0.76671959	+3.57943778
C	−1.55477752	−0.23077674	+3.45189556
O	+5.66473568	+3.27119422	−0.40283441
H	+6.28833859	+2.59792490	−0.13896568

C	−1.58009141	−2.52841303	+1.58305347
C	−4.51695495	−2.11566745	+2.03253070
H	+4.83651221	+3.33613791	+1.47145783
H	+4.48306976	+1.06644619	+0.69255255
H	+3.66252136	+1.65132242	−0.73968187
H	+3.61910720	+5.11864664	+0.79563333
H	+4.00244160	+4.78925603	−0.87835941
H	+2.08943908	+3.42275994	−1.18146889
H	+1.50113800	+4.75352610	−0.22034685
H	+0.27273409	+4.17405233	+1.75860527
H	+0.27232490	+2.51797545	+2.38395632
H	+3.12941522	+0.55718293	+2.66950642
H	+1.22676583	−0.71727013	+2.96804359
H	+0.92908966	+0.01000294	−0.55215114
H	−1.15867985	−0.13049038	−1.79516197
H	−1.70381092	+1.05557540	−0.64386884
H	−3.34755717	−0.55006830	−0.41167315
H	−2.26797752	−1.88899940	−0.69889997
H	−3.96880274	−0.07866068	+1.79606814
H	−3.00673200	−1.70253500	+4.13784661
H	−3.64221737	−0.07976491	+4.11798414
H	−0.84423340	−0.95063941	+3.85339509
H	−1.39894065	+0.69684904	+3.99763817
H	−1.48430060	−2.74984409	+2.64511514
H	−0.58956843	−2.57251634	+1.13525488
H	−2.18050963	−3.31774760	+1.13482360
H	−5.41791077	−1.87996434	+2.59583782
H	−4.12286860	−3.05203342	+2.42382778
H	−4.80933035	−2.28094070	+0.99617485
H	−1.86637187	+0.88679217	+1.67945603

51

* E = +11.061 kcal/mol ; (97) 313_047_000_314_032_017_178_306_349

C	+0.00000000	+0.00000000	+0.00000000
C	+0.00000000	+0.00000000	+1.34069468
C	+1.16552320	+0.00000000	+2.21662433
C	+2.47473428	−0.18140642	+1.97986967
C	+3.20352580	−0.55180434	+0.77068884
C	+2.97748015	−1.62155669	−0.00494099
C	+1.90839011	−2.62568107	+0.21824343
H	+2.30403641	−3.63412219	+0.10124086

C	+3.90746785	−1.81702923	−1.16538710
C	+4.10178804	−0.52329221	−1.94695536
C	+4.39443303	+0.67352873	−1.04621429
C	+4.41461934	+0.27187969	+0.42235247
C	−1.28918046	+0.16978325	−0.73591395
C	−2.43747397	−0.69660681	−0.19155846
C	−2.33247029	−0.92774011	+1.31691787
C	−1.37330859	+0.09987451	+1.94175578
C	−3.60084162	−0.62622331	+2.12176066
C	−3.08333743	−0.44464180	+3.55592728
C	−1.59730032	−0.05865587	+3.43780757
O	+5.63861313	+1.20786385	−1.44548289
H	+5.83254530	+1.97952584	−0.91732863
C	−1.84767766	−2.34765792	+1.58106921
C	−4.72774127	−1.62649858	+1.99618308
H	+3.61795724	+1.42927727	−1.18345728
H	+4.47620767	+1.16034834	+1.04906338
H	+5.32926451	−0.30454197	+0.58667457
H	+3.20734212	−0.33015701	−2.53545031
H	+4.92889137	−0.61948925	−2.64658550
H	+4.87646883	−2.16346409	−0.79404839
H	+3.53795738	−2.59907161	−1.82564763
H	+1.10550788	−2.50970254	−0.51466253
H	+1.46678307	−2.52964887	+1.20526987
H	+3.12429607	+0.02277360	+2.82513901
H	+0.92980576	+0.26003124	+3.24015967
H	+0.92240200	−0.04824005	−0.55811498
H	−1.15472293	−0.01205122	−1.79953095
H	−1.58635179	+1.22008054	−0.64951886
H	−3.38479972	−0.21629336	−0.43887975
H	−2.43722313	−1.65541249	−0.70752157
H	−3.96534435	+0.34089724	+1.76462767
H	−3.19565194	−1.37448641	+4.11316948
H	−3.66186218	+0.30493604	+4.09121410
H	−0.96327047	−0.84776136	+3.83941817
H	−1.35729369	+0.84773443	+3.98905432
H	−1.77043307	−2.57082716	+2.64398480
H	−0.87075334	−2.50284160	+1.12992033
H	−2.53317281	−3.06924409	+1.14042292
H	−5.60511458	−1.29457017	+2.54810092

H	−4.43973737	−2.59789803	+2.39440847
H	−5.02412331	−1.76365591	+0.95681180
H	−1.77854046	+1.07982739	+1.65794942

51

* E = +11.370 kcal/mol ; (98) 314_059_340_314_032_017_067_306_349

C	+0.00000000	+0.00000000	+0.00000000
C	+0.00000000	+0.00000000	+1.34062120
C	+1.16555553	+0.00000000	+2.21661788
C	+2.47449781	−0.18106671	+1.97914072
C	+3.20377972	−0.55134969	+0.76951380
C	+2.97781082	−1.62939967	+0.00567315
C	+1.90468041	−2.62834958	+0.23327656
H	+2.29689298	−3.63848291	+0.11869806
C	+3.88334164	−1.82076490	−1.17444078
C	+4.02884192	−0.49961471	−1.91281656
C	+4.60537584	+0.59053409	−1.01461419
C	+4.43737213	+0.25629402	+0.46178090
C	−1.28857299	+0.17197130	−0.73665960
C	−2.43995607	−0.69038212	−0.19234485
C	−2.33394833	−0.92468568	+1.31544642
C	−1.37324417	+0.10040411	+1.94193699
C	−3.60145392	−0.62381423	+2.12183260
C	−3.08274203	−0.44698117	+3.55612062
C	−1.59650962	−0.06175215	+3.43779344
O	+5.99717775	+0.74992107	−1.20221621
H	+6.15951258	+1.09057953	−2.07898698
C	−1.85035143	−2.34558747	+1.57637636
C	−4.72964886	−1.62233790	+1.99388332
H	+4.10470014	+1.53655345	−1.23016304
H	+4.47180569	+1.16908842	+1.04959333
H	+5.32410980	−0.32277963	+0.73239794
H	+3.04381350	−0.19586533	−2.26614748
H	+4.66372060	−0.60805700	−2.79200966
H	+4.86887152	−2.16377940	−0.84609875
H	+3.49021988	−2.58937483	−1.83719917
H	+1.10068459	−2.51219725	−0.49866327
H	+1.46500379	−2.52837458	+1.22067904
H	+3.12426286	+0.02452611	+2.82386467
H	+0.93004951	+0.26121932	+3.23987399
H	+0.92299153	−0.04839815	−0.55715596

H	−1.15420268	−0.01143479	−1.80010687
H	−1.58297672	+1.22317387	−0.65169307
H	−3.38549070	−0.20524970	−0.43718414
H	−2.44510549	−1.64831549	−0.70996248
H	−3.96487091	+0.34482947	+1.76769682
H	−3.19534910	−1.37841228	+4.11066215
H	−3.66027472	+0.30144890	+4.09408943
H	−0.96257267	−0.85241044	+3.83648383
H	−1.35526705	+0.84291617	+3.99124533
H	−1.77308139	−2.57075700	+2.63883661
H	−0.87355744	−2.50028412	+1.12491899
H	−2.53643778	−3.06581412	+1.13438746
H	−5.60636845	−1.29110130	+2.54726997
H	−4.44251334	−2.59527198	+2.38896847
H	−5.02671045	−1.75603653	+0.95423244
H	−1.77730367	+1.08162889	+1.66073604

51

* E = +11.461 kcal/mol ; (99) 051_310_003_314_032_017_177_306_350

C	+0.00000000	+0.00000000	+0.00000000
C	+0.00000000	+0.00000000	+1.34055225
C	+1.16621120	+0.00000000	+2.21567707
C	+2.47463746	−0.17868179	+1.97372343
C	+3.19864775	−0.54235891	+0.76012067
C	+2.97813556	−1.61551013	−0.01161678
C	+1.91191222	−2.62194291	+0.21532044
H	+2.30974267	−3.63053939	+0.10874622
C	+3.91999756	−1.81495133	−1.16053347
C	+5.36603956	−1.74952513	−0.68196485
C	+5.64503641	−0.51752398	+0.17309368
C	+4.38289575	+0.30865924	+0.39428118
C	−1.28960947	+0.16497100	−0.73632821
C	−2.43484178	−0.70548704	−0.19214365
C	−2.32904606	−0.93631195	+1.31626011
C	−1.37365147	+0.09448093	+1.94170430
C	−3.59858094	−0.63973790	+2.12115913
C	−3.08180493	−0.45741751	+3.55544033
C	−1.59694494	−0.06669541	+3.43762748
O	+6.16889705	−0.98715185	+1.39739782
H	+6.40562060	−0.24192533	+1.94548725
C	−1.83886116	−2.35443213	+1.58072661

C	−4.72213951	−1.64367105	+1.99449793
H	+6.39026482	+0.11631470	−0.31418797
H	+4.16816769	+0.85645526	−0.52764164
H	+4.56977115	+1.06757502	+1.15408440
H	+6.04748568	−1.79319255	−1.52877719
H	+5.57350701	−2.61540887	−0.05734979
H	+3.73462694	−2.76819778	−1.65087636
H	+3.75236912	−1.04185308	−1.91676249
H	+1.10946923	−2.51299898	−0.51936497
H	+1.46779015	−2.51808265	+1.20037015
H	+3.12982060	+0.01692731	+2.81658254
H	+0.93184569	+0.25276040	+3.24128811
H	+0.92260880	−0.04534893	−0.55791475
H	−1.15419160	−0.01683930	−1.79984591
H	−1.59073690	+1.21421968	−0.65046015
H	−3.38403269	−0.22872351	−0.43935723
H	−2.43107043	−1.66437089	−0.70799775
H	−3.96630912	+0.32644464	+1.76472442
H	−3.19137958	−1.38784563	+4.11227959
H	−3.66272409	+0.29016001	+4.09099363
H	−0.96039176	−0.85450504	+3.83769030
H	−1.35941107	+0.83959213	+3.99010907
H	−1.76325801	−2.57757704	+2.64376274
H	−0.86010093	−2.50524718	+1.13210121
H	−2.51990544	−3.07895181	+1.13801332
H	−5.60093241	−1.31498183	+2.54614651
H	−4.43118353	−2.61438160	+2.39225709
H	−5.01746483	−1.78116312	+0.95484764
H	−1.78263807	+1.07328134	+1.65920123

51

* E = +11.598 kcal/mol ; (100) 048_307_007_314_031_018_059_053_043

C	+0.00000000	+0.00000000	+0.00000000
C	+0.00000000	+0.00000000	+1.33604293
C	+1.21337608	+0.00000000	+2.15051610
C	+2.31972884	+0.72694813	+1.96446736
C	+2.57814999	+1.75840892	+0.95794802
C	+1.81334986	+2.82796995	+0.71045685
C	+0.53427859	+3.15666721	+1.39475181
H	−0.31700003	+2.93310856	+0.74747226
C	+2.28037185	+3.73996961	−0.38552399

C	+2.63421834	+2.92550534	−1.62397873
C	+3.62063810	+1.79432759	−1.31666116
C	+3.85939831	+1.65632291	+0.17746548
C	−1.30643454	+0.03595198	−0.72505308
C	−2.35657680	−0.94433129	−0.16907043
C	−2.20973332	−1.17814606	+1.33565273
C	−1.36077916	−0.05829961	+1.96127683
C	−3.49261687	−1.02456189	+2.15917402
C	−2.98178205	−0.79170603	+3.58913652
C	−1.54642315	−0.24817865	+3.45865259
O	+3.13617430	+0.54410457	−1.76523000
H	+2.99014256	+0.58377536	−2.70774447
C	−1.56383119	−2.53622990	+1.58123333
C	−4.50176831	−2.14460625	+2.04210237
H	+4.58127454	+1.99322513	−1.79794837
H	+4.54418193	+2.45246069	+0.48113754
H	+4.36477564	+0.71099387	+0.35627052
H	+3.01800729	+3.57286966	−2.41141396
H	+1.71808299	+2.46792448	−1.99700350
H	+1.51386623	+4.47330844	−0.62868373
H	+3.15693323	+4.30802729	−0.05841995
H	+0.48906997	+4.21968752	+1.63059264
H	+0.40698058	+2.58831007	+2.31117901
H	+3.15584166	+0.51651204	+2.62311441
H	+1.21535406	−0.68054662	+2.99332932
H	+0.92863597	+0.01412894	−0.55423487
H	−1.16643600	−0.13361919	−1.78994922
H	−1.71100974	+1.05011397	−0.63511317
H	−3.35030555	−0.55716679	−0.39759219
H	−2.27053040	−1.89414920	−0.69423379
H	−3.96793608	−0.10301535	+1.81196445
H	−2.98903386	−1.72945705	+4.14452412
H	−3.63244769	−0.10971571	+4.13209885
H	−0.83165898	−0.96792664	+3.85274559
H	−1.39281643	+0.67611723	+4.01045405
H	−1.46438589	−2.76247004	+2.64198851
H	−0.57384756	−2.57053603	+1.13156687
H	−2.15968549	−3.32744713	+1.13001789
H	−5.40186315	−1.91835852	+2.61079743
H	−4.09895850	−3.08015666	+2.42646370

H	−4.79748905	−2.30660233	+1.00612878
H	−1.87133611	+0.87686800	+1.69284232

51

* E = +11.815 kcal/mol ; (101) 051_311_001_314_032_017_055_305_350

C	+0.00000000	+0.00000000	+0.00000000
C	+0.00000000	+0.00000000	+1.34058164
C	+1.16573820	+0.00000000	+2.21657411
C	+2.47422574	−0.17883020	+1.97553725
C	+3.19779199	−0.54411401	+0.76236229
C	+2.97852705	−1.61927732	−0.00675154
C	+1.90931797	−2.62359053	+0.21700093
H	+2.30301665	−3.63391940	+0.10963206
C	+3.92734673	−1.82393082	−1.14922215
C	+5.37220115	−1.75905331	−0.66219923
C	+5.64166304	−0.53163582	+0.21568230
C	+4.38794974	+0.30345215	+0.40521432
C	−1.28974261	+0.16472485	−0.73636529
C	−2.43415142	−0.70699542	−0.19274528
C	−2.32937712	−0.93618540	+1.31603042
C	−1.37398097	+0.09482444	+1.94103687
C	−3.59930132	−0.63847304	+2.11987856
C	−3.08328003	−0.45477712	+3.55428097
C	−1.59810908	−0.06510065	+3.43694906
O	+6.06687459	−0.89272529	+1.51282973
H	+6.83248130	−1.45886472	+1.44972406
C	−1.83982032	−2.35417503	+1.58236624
C	−4.72306066	−1.64225247	+1.99359093
H	+6.40746138	+0.09623545	−0.24640281
H	+4.18853245	+0.84272159	−0.52391582
H	+4.58307566	+1.05293623	+1.16801503
H	+6.05383765	−1.78399179	−1.51105772
H	+5.57031711	−2.64774376	−0.06401791
H	+3.74240044	−2.77658127	−1.64119785
H	+3.77127943	−1.05022655	−1.90669566
H	+1.10665057	−2.51130500	−0.51699842
H	+1.46532808	−2.51915691	+1.20213445
H	+3.13349850	+0.01753106	+2.81435610
H	+0.93116516	+0.25497544	+3.24157149
H	+0.92277906	−0.04427974	−0.55777569
H	−1.15431667	−0.01644083	−1.80004382

H	−1.59188699	+1.21365789	−0.64999541
H	−3.38397908	−0.23214706	−0.44134779
H	−2.42823928	−1.66642759	−0.70766221
H	−3.96656500	+0.32741298	+1.76217585
H	−3.19391760	−1.38447595	+4.11218299
H	−3.66409879	+0.29381868	+4.08854739
H	−0.96209041	−0.85289374	+3.83782692
H	−1.36012422	+0.84139130	+3.98880478
H	−1.76535385	−2.57616414	+2.64572715
H	−0.86055004	−2.50527188	+1.13492918
H	−2.52054125	−3.07902447	+1.13959617
H	−5.60214739	−1.31276655	+2.54432820
H	−4.43258410	−2.61260323	+2.39262860
H	−5.01775296	−1.78084424	+0.95388378
H	−1.78268488	+1.07349806	+1.65764037

51

* E = +11.845 kcal/mol ; (102) 313_052_353_314_032_017_293_306_349

C	+0.00000000	+0.00000000	+0.00000000
C	+0.00000000	+0.00000000	+1.34060907
C	+1.16585549	+0.00000000	+2.21600938
C	+2.47447417	−0.18589138	+1.98010005
C	+3.20188169	−0.56559937	+0.77286566
C	+2.97057193	−1.63956399	+0.00435913
C	+1.89448657	−2.63548347	+0.22977051
H	+2.28219041	−3.64708102	+0.11306964
C	+3.89097846	−1.83868275	−1.16303028
C	+4.06308480	−0.53714255	−1.93561102
C	+4.46636102	+0.63699687	−1.03640052
C	+4.42539738	+0.24398524	+0.43523594
C	−1.28817106	+0.17415268	−0.73661971
C	−2.44182517	−0.68460144	−0.19136466
C	−2.33619442	−0.91879790	+1.31649760
C	−1.37268297	+0.10396150	+1.94230347
C	−3.60260794	−0.61414267	+2.12323906
C	−3.08289277	−0.43808541	+3.55727056
C	−1.59579488	−0.05644461	+3.43833215
O	+5.72753537	+1.16850133	−1.37299661
H	+6.40009378	+0.51756080	−1.17925716
C	−1.85664156	−2.34102298	+1.57768246
C	−4.73365009	−1.60955277	+1.99624325

H	+3.78106642	+1.46520619	−1.19409508
H	+4.50194995	+1.13719201	+1.04870139
H	+5.32037618	−0.35653630	+0.63906038
H	+3.12295173	−0.30898929	−2.43481067
H	+4.81032903	−0.64227033	−2.72048445
H	+4.86459084	−2.18862733	−0.80349541
H	+3.51407640	−2.61942468	−1.82074970
H	+1.09083656	−2.51397307	−0.50145613
H	+1.45535633	−2.53499688	+1.21740924
H	+3.12491410	+0.02286260	+2.82336417
H	+0.93134222	+0.26699410	+3.23801574
H	+0.92260021	−0.04824977	−0.55784542
H	−1.15419140	−0.01017755	−1.79987644
H	−1.57919505	+1.22630484	−0.65244202
H	−3.38587696	−0.19668227	−0.43618165
H	−2.45007406	−1.64264709	−0.70871841
H	−3.96350676	+0.35529968	+1.76880974
H	−3.19752027	−1.36908326	+4.11212066
H	−3.65837829	+0.31188000	+4.09524456
H	−0.96354009	−0.84809889	+3.83783454
H	−1.35254305	+0.84827714	+3.99082786
H	−1.77869734	−2.56595647	+2.64016201
H	−0.88099707	−2.49920937	+1.12492104
H	−2.54556926	−3.05927878	+1.13694792
H	−5.60924530	−1.27530311	+2.54956256
H	−4.44935838	−2.58303377	+2.39205663
H	−5.03139192	−1.74310420	+0.95678552
H	−1.77406710	+1.08606433	+1.66052964

51

* E = +13.736 kcal/mol ; (103) 048_310_003_313_033_017_058_073_345

C	+0.00000000	+0.00000000	+0.00000000
C	+0.00000000	+0.00000000	+1.34024217
C	+1.16058356	+0.00000000	+2.22292963
C	+2.45333682	−0.25834053	+1.98877816
C	+3.14072424	−0.60068981	+0.73954995
C	+3.39095266	+0.24151876	−0.26755727
C	+2.95686563	+1.66367193	−0.31974318
H	+2.31833293	+1.84354860	−1.18638061
C	+4.16122477	−0.31333648	−1.42850306
C	+3.56324923	−1.64326830	−1.87529779

C	+3.35095857	−2.61733592	−0.70987153
C	+3.72030772	−1.98378661	+0.61987773
C	−1.30257648	−0.00062512	−0.73126490
C	−2.31182861	−1.02676388	−0.19006562
C	−2.17565042	−1.23118811	+1.31951565
C	−1.37782209	−0.06720735	+1.93777178
C	−3.47888723	−1.11166819	+2.11559313
C	−3.00692254	−0.83221052	+3.54906986
C	−1.58937050	−0.24211560	+3.43414115
O	+2.00059943	−3.01446748	−0.59806973
H	+1.71950735	−3.41224065	−1.41882498
C	−1.48088127	−2.56002993	+1.59278423
C	−4.44187323	−2.27176854	+2.00064805
H	+3.97200338	−3.50578271	−0.84854884
H	+4.81096604	−1.94124080	+0.68362567
H	+3.37260823	−2.63284016	+1.41852026
H	+4.18251549	−2.09424218	−2.64931824
H	+2.58771645	−1.44490355	−2.31911806
H	+4.16935144	+0.38985363	−2.25922625
H	+5.20691324	−0.46372933	−1.14351029
H	+3.81818523	+2.32460150	−0.42395338
H	+2.40443011	+1.94738831	+0.57079180
H	+3.09425228	−0.27631510	+2.86496526
H	+0.92033726	+0.19802494	+3.25886601
H	+0.91693075	+0.00404822	−0.56239349
H	−1.14758219	−0.15162715	−1.79699062
H	−1.74586471	+0.99529087	−0.63284590
H	−3.31985516	−0.69433838	−0.44156094
H	−2.16489609	−1.97687749	−0.70094831
H	−3.98221166	−0.21568657	+1.74148097
H	−2.99116197	−1.75793581	+4.12408123
H	−3.69147135	−0.16244538	+4.06509627
H	−0.85851861	−0.93514826	+3.84674225
H	−1.47933105	+0.69292615	+3.97906945
H	−1.38109191	−2.76446033	+2.65792700
H	−0.48692563	−2.57073210	+1.14996800
H	−2.04835929	−3.37851043	+1.15295706
H	−5.36174631	−2.06950126	+2.54639651
H	−4.01110608	−3.18319463	+2.41157976
H	−4.70939914	−2.46523650	+0.96235487

H	−1.91358768	+0.84271094	+1.63947750

S3.2. Cartesian Coordinates of TS(+) Conformers

```
51
 * E =  +0.000  kcal/mol ; (1) 334_047_298_022_316_062_278
 C   +0.00000000   +0.00000000   +0.00000000
 C   +0.00000000   +0.00000000   +1.40967937
 C   +1.17478891   +0.00000000   +2.14884293
 C   +2.51584943   +0.06749202   +1.76108514
 C   +3.18933941   +0.52002403   +0.62308776
 C   +2.70731898   +1.43541076   −0.32701732
 C   +1.51466411   +2.13494030   −0.13714319
 H   +0.51250792   +1.20191037   −0.27032383
 C   +3.41187595   +1.61081745   −1.64879847
 C   +4.88765714   +1.27281951   −1.62272699
 C   +5.10807112   −0.04570148   −0.91195297
 C   +4.63672371   +0.07196211   +0.51978969
 C   −1.25942103   −0.25355985   −0.79738668
 C   −2.54809526   +0.02530905   −0.03608435
 C   −2.46601648   −0.54684988   +1.36553575
 C   −1.32313410   +0.15860523   +2.09210036
 C   −3.63045625   −0.19650019   +2.29399190
 C   −3.02978947   −0.37780673   +3.69922559
 C   −1.50222061   −0.25740870   +3.54435406
 O   +4.45218140   −1.10158340   −1.57802533
 H   +3.53114398   −1.09724445   −1.31752904
 C   −2.24288462   −2.05383038   +1.32001937
 C   −4.91661007   −0.96099683   +2.07431735
 H   +6.16442519   −0.30544422   −0.92852764
 H   +5.27691710   +0.78880434   +1.03807265
 H   +4.76652479   −0.88789005   +1.01373032
 H   +5.44513514   +2.05012341   −1.09926105
 H   +5.27566544   +1.21668581   −2.63752154
 H   +2.92336035   +0.96450371   −2.38442234
 H   +3.26458939   +2.62753788   −2.00666093
 H   +1.28625495   +2.92874562   −0.83694369
 H   +1.20620114   +2.32104088   +0.88691192
 H   +3.18660402   −0.32447681   +2.51917495
 H   +1.04048994   −0.16095614   +3.21117589
 H   +0.88805689   −0.46076007   −0.43238405
 H   −1.25183276   −1.29866536   −1.11296798
```

H	−1.23537331	+0.32617297	−1.71951704
H	−2.71534095	+1.10231281	+0.03696403
H	−3.39346728	−0.39103748	−0.58557433
H	−3.83544264	+0.86722940	+2.14290301
H	−3.30094823	−1.35536042	+4.09724439
H	−3.43024939	+0.35689554	+4.39397750
H	−1.01578351	−1.21534237	+3.72534164
H	−1.06660553	+0.45643343	+4.23914697
H	−2.33295616	−2.50296928	+2.30731478
H	−1.25151706	−2.29629952	+0.94280449
H	−2.97762269	−2.53227389	+0.67408812
H	−5.69534985	−0.61279289	+2.75013070
H	−4.77825577	−2.02549283	+2.25658550
H	−5.28599280	−0.83940561	+1.05704685
H	−1.56782771	+1.22830189	+2.04239027

51

* E = +0.012 kcal/mol ; (2) 006_315_066_023_316_062_183

C	+0.00000000	+0.00000000	+0.00000000
C	+0.00000000	+0.00000000	+1.41311574
C	+1.17480821	+0.00000000	+2.15014701
C	+2.51411581	+0.05844437	+1.75546454
C	+3.18029764	+0.49277405	+0.60556845
C	+2.70766007	+1.42061438	−0.33411900
C	+1.52534577	+2.13251584	−0.14143349
H	+0.50040186	+1.18132144	−0.27447583
C	+3.45695108	+1.68149648	−1.61783992
C	+4.64548494	+0.77353415	−1.84595960
C	+5.42869146	+0.66632743	−0.56679690
C	+4.58397196	−0.05452803	+0.45702503
C	−1.26383127	−0.26236856	−0.79053776
C	−2.55114827	+0.01818143	−0.02751452
C	−2.46532670	−0.54732447	+1.37639729
C	−1.32174141	+0.16385653	+2.09640009
C	−3.62851142	−0.19430681	+2.30543281
C	−3.02480663	−0.36709943	+3.71024277
C	−1.49802945	−0.24295851	+3.55182372
O	+6.62125072	−0.02962837	−0.83720316
H	+7.10594783	−0.15371641	−0.02398402
C	−2.23954695	−2.05401241	+1.33806338
C	−4.91358743	−0.96263002	+2.09276200

H	+5.65832105	+1.67722739	−0.21056961
H	+5.07149398	−0.03463134	+1.43421340
H	+4.54561897	−1.10304721	+0.15525440
H	+5.27954223	+1.15907294	−2.64106645
H	+4.32634178	−0.22736006	−2.13904250
H	+2.75834436	+1.60339548	−2.45127709
H	+3.79526230	+2.71948171	−1.61327044
H	+1.30147141	+2.92869091	−0.84063431
H	+1.20453237	+2.31018330	+0.87970305
H	+3.18803367	−0.33260873	+2.51137861
H	+1.04289503	−0.14726684	+3.21485137
H	+0.88303591	−0.47370833	−0.42849250
H	−1.25551898	−1.30944979	−1.09902786
H	−1.24479572	+0.31121319	−1.71663481
H	−2.71983064	+1.09527569	+0.04151784
H	−3.39742307	−0.40172262	−0.57312351
H	−3.83596178	+0.86819384	+2.14919530
H	−3.29222452	−1.34383824	+4.11296379
H	−3.42636920	+0.36934442	+4.40256363
H	−1.00831437	−1.19821315	+3.73817822
H	−1.06323969	+0.47658554	+4.24125151
H	−2.32699195	−2.49820466	+2.32789134
H	−1.24829495	−2.29571992	+0.96035631
H	−2.97438791	−2.53721005	+0.69573278
H	−5.69137618	−0.61296574	+2.76896553
H	−4.77230369	−2.02593073	+2.27983703
H	−5.28581059	−0.84686742	+1.07584943
H	−1.56767731	+1.23300494	+2.03944659

51

* E = +0.055 kcal/mol ; (3) 007_314_066_023_316_062_060

C	+0.00000000	+0.00000000	+0.00000000
C	+0.00000000	+0.00000000	+1.41305529
C	+1.17489921	+0.00000000	+2.15005336
C	+2.51432587	+0.05771556	+1.75617105
C	+3.18217390	+0.49135622	+0.60674556
C	+2.70833814	+1.41974649	−0.33146164
C	+1.52551843	+2.13189541	−0.14146621
H	+0.49994875	+1.18160712	−0.27525665
C	+3.46020758	+1.68263613	−1.61296421
C	+4.63222104	+0.75417226	−1.84585008

C	+5.42712481	+0.64210099	−0.56779727
C	+4.58534607	−0.05672175	+0.46617704
C	−1.26364839	−0.26348648	−0.79050250
C	−2.55119265	+0.01624779	−0.02753698
C	−2.46492776	−0.54909791	+1.37641313
C	−1.32188075	+0.16305767	+2.09632890
C	−3.62842563	−0.19699683	+2.30538632
C	−3.02455539	−0.36922863	+3.71019800
C	−1.49788214	−0.24389127	+3.55174652
O	+6.60169302	−0.11845205	−0.71656881
H	+7.16244254	+0.29442524	−1.36942566
C	−2.23790771	−2.05560525	+1.33812357
C	−4.91286849	−0.96640296	+2.09274029
H	+5.67575933	+1.65021743	−0.21604450
H	+5.08657348	−0.02107611	+1.43263121
H	+4.54963289	−1.10999428	+0.18263608
H	+5.25264886	+1.13226319	−2.65925745
H	+4.29553902	−0.24319012	−2.13134439
H	+2.76087832	+1.62531748	−2.44740896
H	+3.81825473	+2.71415866	−1.59835139
H	+1.30326104	+2.92841699	−0.84089138
H	+1.20381579	+2.31044297	+0.87924739
H	+3.18803523	−0.33367040	+2.51174728
H	+1.04277031	−0.14766453	+3.21467710
H	+0.88343932	−0.47344820	−0.42808977
H	−1.25474614	−1.31065713	−1.09874901
H	−1.24522956	+0.30998178	−1.71675459
H	−2.72052908	+1.09325365	+0.04149815
H	−3.39724651	−0.40423953	−0.57309950
H	−3.83676019	+0.86533436	+2.14909208
H	−3.29121785	−1.34615755	+4.11297525
H	−3.42672948	+0.36692949	+4.40248405
H	−1.00736028	−1.19870516	+3.73811229
H	−1.06358873	+0.47595498	+4.24114396
H	−2.32526917	−2.49987053	+2.32791702
H	−1.24627749	−2.29643250	+0.96087154
H	−2.97215741	−2.53943164	+0.69555296
H	−5.69095085	−0.61742074	+2.76897529
H	−4.77064494	−2.02957811	+2.27981871
H	−5.28521628	−0.85098924	+1.07581680

H −1.56881363 +1.23200039 +2.03938676
51
* E = +0.141 kcal/mol ; (4) 330_050_299_022_316_062_190
C +0.00000000 +0.00000000 +0.00000000
C +0.00000000 +0.00000000 +1.40994973
C +1.17507451 +0.00000000 +2.14858438
C +2.51464941 +0.06050160 +1.75416558
C +3.17897558 +0.50199828 +0.60789859
C +2.70322659 +1.42456673 −0.33475476
C +1.51642599 +2.13219627 −0.13709637
H +0.50980171 +1.20317823 −0.27160534
C +3.40920627 +1.59751076 −1.65282015
C +4.89650913 +1.32221509 −1.59404588
C +5.14417086 −0.00596099 −0.92316010
C +4.61395593 +0.02717489 +0.49238336
C −1.26106168 −0.25240910 −0.79534996
C −2.54918808 +0.02718687 −0.03330172
C −2.46636802 −0.54473680 +1.36835070
C −1.32245629 +0.16053589 +2.09358056
C −3.63000222 −0.19472914 +2.29790090
C −3.02803601 −0.37633796 +3.70254180
C −1.50066817 −0.25582173 +3.54590055
O +4.49091858 −0.98196671 −1.70505144
H +4.48530066 −1.81232681 −1.23454318
C −2.24338772 −2.05175465 +1.32240970
C −4.91638197 −0.95924540 +2.07939405
H +6.21722958 −0.21425989 −0.89663254
H +5.26244000 +0.68398150 +1.07744246
H +4.71403492 −0.96399790 +0.93547380
H +5.40957812 +2.09884702 −1.02714850
H +5.31768827 +1.31315579 −2.59720599
H +2.96619500 +0.90304194 −2.36964555
H +3.22925634 +2.60069861 −2.03346975
H +1.29125035 +2.93103147 −0.83219647
H +1.21121154 +2.31512589 +0.88857799
H +3.18819677 −0.33498696 +2.50893225
H +1.04262399 −0.15528230 +3.21214188
H +0.88765139 −0.45853715 −0.43480360
H −1.25458932 −1.29736480 −1.11172047
H −1.23752334 +0.32720751 −1.71757104

H	−2.71550689	+1.10436348	+0.04027412
H	−3.39576395	−0.38838956	−0.58180338
H	−3.83519027	+0.86901037	+2.14722985
H	−3.29897981	−1.35409115	+4.10047623
H	−3.42794367	+0.35811502	+4.39798890
H	−1.01389705	−1.21381527	+3.72604369
H	−1.06423260	+0.45767818	+4.24058727
H	−2.33268222	−2.50115740	+2.30975693
H	−1.25212604	−2.29290312	+0.94419953
H	−2.97855020	−2.53004665	+0.67672790
H	−5.69464685	−0.61143918	+2.75606290
H	−4.77759105	−2.02381681	+2.26116186
H	−5.28659014	−0.83749182	+1.06242148
H	−1.56775410	+1.23016005	+2.04445094

51

* E = +0.318 kcal/mol ; (5) 007_315_066_023_316_062_296

C	+0.00000000	+0.00000000	+0.00000000
C	+0.00000000	+0.00000000	+1.41313107
C	+1.17480944	+0.00000000	+2.15024637
C	+2.51417565	+0.05944377	+1.75628445
C	+3.18115206	+0.49421940	+0.60684836
C	+2.70807778	+1.42091757	−0.33351549
C	+1.52531372	+2.13264966	−0.14268089
H	+0.50028859	+1.18105777	−0.27487457
C	+3.45883717	+1.68239257	−1.61601479
C	+4.63751351	+0.76200747	−1.84959084
C	+5.43367195	+0.64924243	−0.57258836
C	+4.58800970	−0.04757706	+0.46702254
C	−1.26380562	−0.26263995	−0.79057720
C	−2.55115793	+0.01767021	−0.02749675
C	−2.46521068	−0.54789583	+1.37638748
C	−1.32176692	+0.16346755	+2.09639247
C	−3.62843852	−0.19516225	+2.30547609
C	−3.02462536	−0.36789602	+3.71024302
C	−1.49789357	−0.24322881	+3.55186658
O	+6.66892513	+0.00113486	−0.74974386
H	+6.50681325	−0.90035677	−1.02300744
C	−2.23915181	−2.05454179	+1.33801647
C	−4.91336762	−0.96373050	+2.09281301
H	+5.69044537	+1.65115755	−0.22762499

H	+5.08929214	−0.01123010	+1.43359563
H	+4.53885881	−1.10641964	+0.19337535
H	+5.26729531	+1.13903464	−2.65252583
H	+4.29336509	−0.23402926	−2.14104599
H	+2.75861940	+1.62134583	−2.44953807
H	+3.81357494	+2.71484771	−1.60420463
H	+1.30155918	+2.92813423	−0.84269485
H	+1.20424543	+2.31150346	+0.87814530
H	+3.18861806	−0.32931857	+2.51270767
H	+1.04278265	−0.14726242	+3.21490368
H	+0.88321375	−0.47369011	−0.42824948
H	−1.25552847	−1.30976685	−1.09901736
H	−1.24498682	+0.31103910	−1.71665117
H	−2.71994612	+1.09472365	+0.04155952
H	−3.39736138	−0.40230220	−0.57315652
H	−3.83610110	+0.86729715	+2.14929501
H	−3.29171668	−1.34474477	+4.11289811
H	−3.42640732	+0.36833767	+4.40262793
H	−1.00782699	−1.19825677	+3.73844142
H	−1.06336407	+0.47656292	+4.24115277
H	−2.32643618	−2.49872311	+2.32784742
H	−1.24781440	−2.29603354	+0.96032922
H	−2.97394361	−2.53789625	+0.69573597
H	−5.69114532	−0.61423234	+2.76909260
H	−4.77191193	−2.02701136	+2.27987873
H	−5.28573512	−0.84797713	+1.07594806
H	−1.56782187	+1.23257319	+2.03939324

51

* E = +0.593 kcal/mol ; (6) 044_312_359_023_316_062_284

C	+0.00000000	+0.00000000	+0.00000000
C	+0.00000000	+0.00000000	+1.40838136
C	+1.17072833	+0.00000000	+2.15715609
C	+2.51215873	+0.08533407	+1.77650538
C	+3.15959961	+0.58900310	+0.64665946
C	+2.66992580	+1.49882531	−0.29892375
C	+1.45792227	+2.17794840	−0.16350050
H	+0.49194443	+1.22132216	−0.28579130
C	+3.52640817	+1.68859398	−1.51575671
C	+4.04462921	+0.36147381	−2.05710938
C	+4.64720349	−0.53464938	−0.96315879

C	+4.58115704	+0.16167642	+0.39000968
C	−1.25361151	−0.27514088	−0.79892312
C	−2.54769574	−0.01361981	−0.04105889
C	−2.45920829	−0.58160512	+1.36169730
C	−1.32726361	+0.14136496	+2.08835423
C	−3.62992025	−0.24632606	+2.28782239
C	−3.02862596	−0.41554397	+3.69428547
C	−1.50274562	−0.27521652	+3.54090776
O	+4.00274556	−1.78566439	−0.90156505
H	+3.15696365	−1.66253065	−0.46801550
C	−2.21466818	−2.08536849	+1.31968747
C	−4.90394794	−1.03083031	+2.06805894
H	+5.68234591	−0.76990490	−1.19718066
H	+5.24927498	+1.02511979	+0.37420393
H	+4.93719733	−0.51987308	+1.15779910
H	+4.77008828	+0.54068705	−2.84835931
H	+3.22315962	−0.19055359	−2.51088339
H	+2.97206997	+2.22477192	−2.28327689
H	+4.37506802	+2.32774339	−1.25509642
H	+1.24188332	+2.95948116	−0.88113640
H	+1.12162153	+2.38006831	+0.84962623
H	+3.19343268	−0.31643167	+2.51877533
H	+1.03054493	−0.17856428	+3.21578442
H	+0.89762267	−0.43370776	−0.43994424
H	−1.22965259	−1.32080758	−1.11195411
H	−1.23586870	+0.30226094	−1.72281607
H	−2.73079458	+1.06096421	+0.02945625
H	−3.38586292	−0.44361856	−0.59104210
H	−3.85061010	+0.81385840	+2.13398632
H	−3.28712991	−1.39546888	+4.09487151
H	−3.43962563	+0.31578650	+4.38642408
H	−1.00452176	−1.22675215	+3.72358016
H	−1.07703346	+0.44478254	+4.23548331
H	−2.30007471	−2.53370694	+2.30776551
H	−1.21943908	−2.31480855	+0.94465618
H	−2.94137846	−2.57554246	+0.67350558
H	−5.68839300	−0.69432100	+2.74318772
H	−4.74883519	−2.09287753	+2.25118436
H	−5.27458387	−0.91589825	+1.05047166
H	−1.58640453	+1.20742045	+2.03666498

51

* E = +0.821 kcal/mol ; (7) 328_052_299_022_316_062_061

C	+0.00000000	+0.00000000	+0.00000000
C	+0.00000000	+0.00000000	+1.41003987
C	+1.17589489	+0.00000000	+2.14740897
C	+2.51410474	+0.05736120	+1.74981412
C	+3.17636113	+0.49637524	+0.60140189
C	+2.70076786	+1.42684299	−0.33238802
C	+1.51351199	+2.13380113	−0.13480691
H	+0.50489407	+1.20264711	−0.27281034
C	+3.41676149	+1.61464689	−1.64242659
C	+4.90949567	+1.38471052	−1.54849958
C	+5.18358817	+0.04349941	−0.90343765
C	+4.60738895	+0.00712067	+0.48915478
C	−1.26121852	−0.25532821	−0.79443344
C	−2.54954375	+0.02235007	−0.03195992
C	−2.46499429	−0.54930226	+1.36969808
C	−1.32224904	+0.15854426	+2.09436301
C	−3.62900522	−0.20183866	+2.29969400
C	−3.02599679	−0.38230207	+3.70405118
C	−1.49895509	−0.25867308	+3.54662403
O	+4.58049319	−1.00628836	−1.62989141
H	+4.94352279	−1.02704305	−2.51274866
C	−2.23856553	−2.05582815	+1.32357957
C	−4.91381481	−0.96916738	+2.08173206
H	+6.26135175	−0.12819383	−0.83938496
H	+5.24214044	+0.62221243	+1.13087750
H	+4.68208826	−1.01337421	+0.85569161
H	+5.37865182	+2.16483874	−0.94811288
H	+5.36348331	+1.43128126	−2.53957400
H	+3.00312004	+0.90614489	−2.36408550
H	+3.21397871	+2.61059773	−2.03032023
H	+1.29077183	+2.93738915	−0.82526366
H	+1.20495624	+2.31066603	+0.89093360
H	+3.18879949	−0.34397141	+2.50028423
H	+1.04492400	−0.15624163	+3.21103593
H	+0.88796877	−0.45990724	−0.43294583
H	−1.25282567	−1.30052129	−1.10984043
H	−1.23966997	+0.32387013	−1.71710433
H	−2.71763105	+1.09930371	+0.04166477

H	−3.39579035	−0.39479049	−0.57989291
H	−3.83660149	+0.86149831	+2.14924841
H	−3.29472948	−1.36067306	+4.10199160
H	−3.42713476	+0.35126987	+4.39976939
H	−1.01011686	−1.21571071	+3.72598960
H	−1.06350843	+0.45537686	+4.24137936
H	−2.32731362	−2.50561006	+2.31079498
H	−1.24663458	−2.29490185	+0.94593977
H	−2.97250184	−2.53562077	+0.67756050
H	−5.69249644	−0.62339187	+2.75900793
H	−4.77238913	−2.03347008	+2.26300722
H	−5.28487040	−0.84796736	+1.06497925
H	−1.56999931	+1.22767645	+2.04545545

51

* E = +0.883 kcal/mol ; (8) 304_039_015_023_316_062_175

C	+0.00000000	+0.00000000	+0.00000000
C	+0.00000000	+0.00000000	+1.40844850
C	+1.17201607	+0.00000000	+2.15516468
C	+2.51163835	+0.07624151	+1.76823702
C	+3.15148637	+0.55958026	+0.62627978
C	+2.66657554	+1.48895599	−0.30167855
C	+1.46046727	+2.17458068	−0.14811443
H	+0.48697447	+1.22285370	−0.28392268
C	+3.51786994	+1.68553056	−1.52078181
C	+4.94474693	+2.08585776	−1.14865684
C	+5.45444287	+1.33981894	+0.07834429
C	+4.57798833	+0.13853569	+0.38979944
C	−1.25548219	−0.27359765	−0.79707973
C	−2.54890730	−0.01042412	−0.03816907
C	−2.45948210	−0.57667999	+1.36508200
C	−1.32621622	+0.14679445	+2.08924962
C	−3.62935889	−0.23945907	+2.29158388
C	−3.02717715	−0.40741282	+3.69763307
C	−1.50161298	−0.26561373	+3.54318296
O	+6.78867980	+0.96755547	−0.18590778
H	+7.16457459	+0.56918878	+0.59610317
C	−2.21520020	−2.08048400	+1.32427484
C	−4.90435206	−1.02301317	+2.07392710
H	+5.42623764	+2.00446238	+0.94549221
H	+4.96164423	−0.39417302	+1.25956769

H	+4.64043351	−0.55564090	−0.45226508
H	+4.99630524	+3.15818586	−0.97705944
H	+5.62045143	+1.85941222	−1.97075111
H	+3.55402940	+0.75170482	−2.08683282
H	+3.07836147	+2.43122690	−2.17823325
H	+1.24768452	+2.97308573	−0.84742886
H	+1.12675308	+2.35522638	+0.86993766
H	+3.19414694	−0.32230438	+2.51159410
H	+1.03368744	−0.16692301	+3.21604286
H	+0.89724606	−0.43762317	−0.43548151
H	−1.23325199	−1.31962691	−1.10952891
H	−1.23869050	+0.30325717	−1.72133445
H	−2.73140314	+1.06436004	+0.03147507
H	−3.38809018	−0.44047990	−0.58673840
H	−3.84921562	+0.82073827	+2.13671083
H	−3.28436391	−1.38765212	+4.09849216
H	−3.43880085	+0.32350037	+4.38991138
H	−1.00205944	−1.21588415	+3.72884655
H	−1.07668515	+0.45706180	+4.23550100
H	−2.30225135	−2.52836085	+2.31247443
H	−1.21919016	−2.30892276	+0.95079768
H	−2.94069632	−2.57112984	+0.67698904
H	−5.68796699	−0.68487722	+2.74926274
H	−4.75005039	−2.08496253	+2.25838485
H	−5.27570163	−0.90908644	+1.05647879
H	−1.58384477	+1.21309137	+2.03443113

51

* E = +1.380 kcal/mol ; (9) 304_040_014_023_316_062_063

C	+0.00000000	+0.00000000	+0.00000000
C	+0.00000000	+0.00000000	+1.40840568
C	+1.17204612	+0.00000000	+2.15514194
C	+2.51189639	+0.07541692	+1.76897155
C	+3.15277924	+0.55823879	+0.62722085
C	+2.66676543	+1.48714960	−0.30012529
C	+1.46084844	+2.17393111	−0.14812794
H	+0.48653503	+1.22326917	−0.28424856
C	+3.51815201	+1.68413394	−1.51877946
C	+4.94350031	+2.08525197	−1.13970832
C	+5.46140481	+1.31557898	+0.07927987
C	+4.57653938	+0.12868417	+0.39299214

C	−1.25526410	−0.27451452	−0.79711420
C	−2.54894225	−0.01221937	−0.03832417
C	−2.45913887	−0.57828956	+1.36497357
C	−1.32639525	+0.14606601	+2.08910731
C	−3.62934243	−0.24183392	+2.29133126
C	−3.02713446	−0.40915550	+3.69744095
C	−1.50165643	−0.26637472	+3.54304944
O	+6.76629480	+0.81905510	−0.11987597
H	+7.36847886	+1.54961795	−0.24030080
C	−2.21376607	−2.08191431	+1.32429616
C	−4.90372786	−1.02639208	+2.07366329
H	+5.45305526	+1.97095548	+0.95354806
H	+4.97106102	−0.39814906	+1.25793831
H	+4.63359437	−0.56793700	−0.44750913
H	+4.98074970	+3.15656890	−0.94940315
H	+5.60965597	+1.87932065	−1.97632258
H	+3.55990003	+0.75014926	−2.08388265
H	+3.07934111	+2.42924179	−2.17760883
H	+1.24918368	+2.97251415	−0.84781617
H	+1.12674610	+2.35556142	+0.86964016
H	+3.19447348	−0.32306097	+2.51188525
H	+1.03348697	−0.16724215	+3.21593981
H	+0.89751172	−0.43733644	−0.43526264
H	−1.23234031	−1.32057238	−1.10944016
H	−1.23893457	+0.30225365	−1.72147957
H	−2.73220465	+1.06245777	+0.03129951
H	−3.38783073	−0.44292612	−0.58687812
H	−3.84999761	+0.81819065	+2.13629646
H	−3.28370098	−1.38949761	+4.09845215
H	−3.43931788	+0.32159482	+4.38958036
H	−1.00145798	−1.21626373	+3.72878085
H	−1.07717339	+0.45655911	+4.23535477
H	−2.30056228	−2.52975088	+2.31252880
H	−1.21752952	−2.30966092	+0.95104849
H	−2.93884951	−2.57315692	+0.67697798
H	−5.68768778	−0.68877243	+2.74887577
H	−4.74859892	−2.08818566	+2.25830884
H	−5.27504846	−0.91295142	+1.05613632
H	−1.58487907	+1.21217930	+2.03419784

51

* E = +1.521 kcal/mol ; (10) 044_307_008_023_316_062_182

C	+0.00000000	+0.00000000	+0.00000000
C	+0.00000000	+0.00000000	+1.40845161
C	+1.17403229	+0.00000000	+2.15169047
C	+2.50970157	+0.07766186	+1.75287897
C	+3.13950021	+0.58033937	+0.61460258
C	+2.65062363	+1.51677055	−0.30273142
C	+1.43825105	+2.19119103	−0.14963005
H	+0.47578044	+1.22553995	−0.28263738
C	+3.49398068	+1.72763738	−1.52562115
C	+3.97231571	+0.40209336	−2.09942813
C	+4.67888216	−0.46031006	−1.05581573
C	+4.55320475	+0.15072863	+0.33590193
C	−1.25516277	−0.28031991	−0.79493111
C	−2.54905521	−0.02011105	−0.03600683
C	−2.45764646	−0.58364399	+1.36842659
C	−1.32538161	+0.14303245	+2.09094331
C	−3.62747348	−0.24767249	+2.29540150
C	−3.02373466	−0.41136373	+3.70151517
C	−1.49832075	−0.26916546	+3.54496688
O	+4.08403211	−1.73706445	−1.12364773
H	+4.47868331	−2.30641300	−0.46638047
C	−2.21010638	−2.08702720	+1.33011787
C	−4.90053248	−1.03506303	+2.08020122
H	+5.74197208	−0.54559071	−1.29588624
H	+5.22944434	+1.00831613	+0.37905649
H	+4.90442526	−0.56145300	+1.08146661
H	+4.61330562	+0.56555989	−2.96283368
H	+3.11165417	−0.16844130	−2.44314573
H	+2.93415063	+2.28517504	−2.27347600
H	+4.35804332	+2.34831780	−1.26919338
H	+1.21722130	+2.98577281	−0.85130314
H	+1.10302549	+2.37208421	+0.86783700
H	+3.19903967	−0.33368664	+2.48311014
H	+1.04206323	−0.17596833	+3.21204577
H	+0.89946028	−0.43383862	−0.43580240
H	−1.22814205	−1.32682169	−1.10500684
H	−1.24141886	+0.29399727	−1.72089904
H	−2.73456634	+1.05432908	+0.03221061
H	−3.38758425	−0.45347575	−0.58312697

H −3.85030163 +0.81164261 +2.13856477
H −3.28032901 −1.39047243 +4.10556822
H −3.43490326 +0.32157983 +4.39200105
H −0.99825768 −1.21938127 +3.72967508
H −1.07229990 +0.45356514 +4.23660529
H −2.29294005 −2.53283578 +2.31970100
H −1.21489928 −2.31409330 +0.95376008
H −2.93694960 −2.58045667 +0.68643103
H −5.68476628 −0.69803963 +2.75545227
H −4.74294128 −2.09628716 +2.26621138
H −5.27267266 −0.92403691 +1.06270165
H −1.58654342 +1.20861281 +2.03631227
51
* E = +1.626 kcal/mol ; (11) 304_036_019_023_316_062_299
C +0.00000000 +0.00000000 +0.00000000
C +0.00000000 +0.00000000 +1.40834386
C +1.17208458 +0.00000000 +2.15497296
C +2.51195025 +0.07714129 +1.76939160
C +3.15261219 +0.55966512 +0.62742321
C +2.66704667 +1.48717716 −0.30188189
C +1.46116904 +2.17375058 −0.14915880
H +0.48717705 +1.22341393 −0.28351074
C +3.50819623 +1.67283019 −1.53037598
C +4.94870489 +2.05260134 −1.18308018
C +5.44776761 +1.35394948 +0.08206857
C +4.58416194 +0.14702177 +0.40416018
C −1.25547025 −0.27268360 −0.79736563
C −2.54878923 −0.00905827 −0.03842935
C −2.45967064 −0.57594260 +1.36459058
C −1.32618610 +0.14673079 +2.08917887
C −3.62939694 −0.23868182 +2.29126568
C −3.02724598 −0.40802412 +3.69715535
C −1.50164131 −0.26646506 +3.54287965
O +6.81430988 +1.02786409 +0.00590086
H +6.94933296 +0.42820489 −0.72580595
C −2.21599382 −2.07983222 +1.32310547
C −4.90485711 −1.02129760 +2.07299205
H +5.37997376 +2.03725716 +0.92537079
H +4.98158020 −0.36070728 +1.27951686
H +4.64449367 −0.56574317 −0.42641381

H	+5.03848360	+3.13005508	−1.06774987
H	+5.60242050	+1.77676726	−2.01120664
H	+3.52071523	+0.73602236	−2.09335697
H	+3.06846573	+2.42054572	−2.18540780
H	+1.24820915	+2.97101234	−0.84982648
H	+1.12926585	+2.35735640	+0.86891194
H	+3.19470174	−0.31714777	+2.51433269
H	+1.03359681	−0.16653124	+3.21584856
H	+0.89736499	−0.43757562	−0.43534105
H	−1.23376821	−1.31866456	−1.11007653
H	−1.23836067	+0.30449628	−1.72145092
H	−2.73063997	+1.06580098	+0.03158216
H	−3.38816086	−0.43842296	−0.58724109
H	−3.84861184	+0.82176339	+2.13716368
H	−3.28463727	−1.38856634	+4.09711848
H	−3.43869160	+0.32233534	+4.39009561
H	−1.00224788	−1.21689027	+3.72807191
H	−1.07658960	+0.45572110	+4.23557851
H	−2.30342676	−2.52811413	+2.31106109
H	−1.21995149	−2.30852204	+0.94983709
H	−2.94152906	−2.56988766	+0.67540672
H	−5.68825713	−0.68310158	+2.74853055
H	−4.75129527	−2.08346590	+2.25678701
H	−5.27614020	−0.90644244	+1.05561366
H	−1.58337523	+1.21315302	+2.03496886

51

* E = +2.275 kcal/mol ; (12) 042_300_020_023_316_062_055

C	+0.00000000	+0.00000000	+0.00000000
C	+0.00000000	+0.00000000	+1.40803154
C	+1.17344569	+0.00000000	+2.15216237
C	+2.51130797	+0.08021671	+1.76104534
C	+3.15056831	+0.58119522	+0.62741182
C	+2.66059626	+1.50974764	−0.29781931
C	+1.44733316	+2.18549992	−0.15416979
H	+0.48372513	+1.22638377	−0.28422304
C	+3.49479052	+1.69707159	−1.53203800
C	+3.98184834	+0.34834275	−2.03872885
C	+4.83771887	−0.36481196	−0.99317577
C	+4.58912015	+0.19724737	+0.39896428
C	−1.25452199	−0.27817488	−0.79656080

C	−2.54847923	−0.01883363	−0.03747834
C	−2.45727453	−0.58587703	+1.36552746
C	−1.32602479	+0.14013472	+2.09033532
C	−3.62788251	−0.25359841	+2.29274358
C	−3.02446687	−0.42062723	+3.69860919
C	−1.49910880	−0.27657418	+3.54313882
O	+4.56001248	−1.74667602	−0.94567209
H	+4.66307279	−2.11958716	−1.81838386
C	−2.20836715	−2.08898938	+1.32358242
C	−4.89989608	−1.04197064	+2.07488151
H	+5.89670257	−0.22242006	−1.22498733
H	+5.22142922	+1.08029039	+0.51282070
H	+4.92632617	−0.53814735	+1.12360605
H	+4.52398341	+0.45302611	−2.97786647
H	+3.10980439	−0.27726607	−2.23066902
H	+2.91847693	+2.21369700	−2.29665004
H	+4.35440982	+2.33732001	−1.31105619
H	+1.23001083	+2.97479215	−0.86316499
H	+1.11278791	+2.37799873	+0.86146857
H	+3.19525436	−0.32944142	+2.49632354
H	+1.03890104	−0.17846066	+3.21169769
H	+0.89964941	−0.43468663	−0.43408841
H	−1.22920843	−1.32427698	−1.10856965
H	−1.24062842	+0.29853949	−1.72120984
H	−2.73305129	+1.05557312	+0.03320984
H	−3.38717253	−0.45033761	−0.58585412
H	−3.85188496	+0.80589477	+2.13879016
H	−3.28027552	−1.40109486	+4.09982153
H	−3.43666328	+0.30995974	+4.39097105
H	−0.99809313	−1.22671944	+3.72527241
H	−1.07407069	+0.44454261	+4.23699864
H	−2.29292185	−2.53744796	+2.31175382
H	−1.21200605	−2.31452701	+0.94936286
H	−2.93350200	−2.58134747	+0.67707517
H	−5.68458860	−0.70815887	+2.75118703
H	−4.74087127	−2.10355676	+2.25756450
H	−5.27218482	−0.92824113	+1.05770985
H	−1.58837794	+1.20554284	+2.03876015

51

* E = +3.318 kcal/mol ; (13) 006_315_066_313_042_008_184

C	+0.00000000	+0.00000000	+0.00000000
C	+0.00000000	+0.00000000	+1.41262775
C	+1.14807935	+0.00000000	+2.18714868
C	+2.50013260	+0.05059633	+1.83737922
C	+3.21481919	+0.43788252	+0.69862981
C	+2.79150860	+1.33930953	−0.28531585
C	+1.61001809	+2.07124962	−0.15608271
H	+0.59997209	+1.14451782	−0.30152356
C	+3.58677573	+1.54682216	−1.54965858
C	+4.76806896	+0.61461789	−1.70638604
C	+5.50480505	+0.53866887	−0.39774950
C	+4.61424760	−0.13486467	+0.61970690
C	−1.34788792	−0.08654030	−0.67144103
C	−2.24539798	−1.17695222	−0.07515015
C	−2.17286849	−1.22462434	+1.45631808
C	−1.37423195	−0.02031588	+1.99423053
C	−3.49808248	−1.00160770	+2.19223189
C	−3.06338173	−0.59243550	+3.60873558
C	−1.62364639	−0.05740527	+3.49083809
O	+6.69490256	−0.18328359	−0.60323315
H	+7.14871268	−0.28790139	+0.23029537
C	−1.52143497	−2.53111253	+1.89174757
C	−4.48060146	−2.15041203	+2.16151290
H	+5.73794584	+1.55744650	−0.06714964
H	+5.06785952	−0.08726769	+1.61218311
H	+4.57051937	−1.19266213	+0.35319899
H	+5.43539460	+0.96399644	−2.49090332
H	+4.44331717	−0.39066184	−1.97742083
H	+2.91490457	+1.45056050	−2.40319108
H	+3.93987577	+2.57979809	−1.56845584
H	+1.42390074	+2.84857459	−0.88723386
H	+1.26524654	+2.29393068	+0.84844018
H	+3.14291554	−0.32147832	+2.62903191
H	+0.98396134	−0.14761368	+3.24653953
H	+0.81980959	−0.54862355	−0.46266281
H	−1.22965875	−0.24359383	−1.74105089
H	−1.85341515	+0.87447132	−0.56376399
H	−3.27151414	−1.01055688	−0.40636275
H	−1.94656915	−2.14161011	−0.48319948
H	−3.97019200	−0.13824299	+1.71476862

H	−3.09973080	−1.45546860	+4.27315909
H	−3.74225584	+0.14608783	+4.02922083
H	−0.92803060	−0.73354825	+3.98420685
H	−1.49529948	+0.91848602	+3.95271652
H	−1.49427023	−2.64350254	+2.97438036
H	−0.49863290	−2.58621237	+1.52320397
H	−2.06794526	−3.38023437	+1.48563682
H	−5.41053754	−1.88011358	+2.65826092
H	−4.07982807	−3.02488694	+2.67131280
H	−4.72431760	−2.43964313	+1.13985804
H	−1.89569764	+0.86481051	+1.60996335

51

* E = +3.367 kcal/mol ; (14) 007_314_066_313_042_008_060

C	+0.00000000	+0.00000000	+0.00000000
C	+0.00000000	+0.00000000	+1.41257107
C	+1.14820848	+0.00000000	+2.18699773
C	+2.50028100	+0.04996330	+1.83777283
C	+3.21632897	+0.43670219	+0.69945730
C	+2.79169928	+1.33913206	−0.28267558
C	+1.60977319	+2.07110742	−0.15578517
H	+0.59914878	+1.14494206	−0.30207282
C	+3.58954825	+1.54939028	−1.54466096
C	+4.75438404	+0.59705558	−1.70646995
C	+5.50264182	+0.51500650	−0.39832599
C	+4.61483304	−0.13675105	+0.62805954
C	−1.34788255	−0.08769622	−0.67138702
C	−2.24475336	−1.17854597	−0.07490894
C	−2.17228785	−1.22579259	+1.45658496
C	−1.37425622	−0.02096289	+1.99417530
C	−3.49766027	−1.00325392	+2.19235090
C	−3.06319080	−0.59346447	+3.60876587
C	−1.62359498	−0.05807961	+3.49079631
O	+6.66937454	−0.26739040	−0.48135504
H	+7.25877673	+0.11550193	−1.12720443
C	−1.52024847	−2.53185756	+1.89235329
C	−4.47959416	−2.15257506	+2.16190667
H	+5.75509428	+1.53029737	−0.07075293
H	+5.08272939	−0.07486339	+1.60980756
H	+4.57284107	−1.19849373	+0.37921110
H	+5.40868580	+0.93925665	−2.50915472

H	+4.41205379	−0.40401935	−1.97147375
H	+2.91741956	+1.47415055	−2.40002225
H	+3.96173637	+2.57605910	−1.55201678
H	+1.42510004	+2.84896504	−0.88683601
H	+1.26414094	+2.29424148	+0.84835588
H	+3.14305535	−0.32262611	+2.62885876
H	+0.98400128	−0.14800150	+3.24631999
H	+0.82034541	−0.54826795	−0.46228637
H	−1.22970727	−0.24492357	−1.74103176
H	−1.85412488	+0.87298834	−0.56390508
H	−3.27099552	−1.01296916	−0.40623974
H	−1.94520009	−2.14313107	−0.48263506
H	−3.97021225	−0.14025097	+1.71463397
H	−3.09936079	−1.45627039	+4.27349616
H	−3.74233729	+0.14501657	+4.02892060
H	−0.92774394	−0.73397920	+3.98410204
H	−1.49543851	+0.91783704	+3.95266155
H	−1.49332452	−2.64410865	+2.97499097
H	−0.49726232	−2.58638285	+1.52427593
H	−2.06617054	−3.38135203	+1.48618584
H	−5.40966514	−1.88268628	+2.65865211
H	−4.07830277	−3.02672185	+2.67185531
H	−4.72317839	−2.44216782	+1.14030688
H	−1.89636907	+0.86382549	+1.60991395

51

* E = +3.413 kcal/mol ; (15) 334_046_298_313_041_009_278

C	+0.00000000	+0.00000000	+0.00000000
C	+0.00000000	+0.00000000	+1.40951666
C	+1.14783635	+0.00000000	+2.18605161
C	+2.50097452	+0.05660229	+1.84125237
C	+3.22185085	+0.46049115	+0.71285286
C	+2.79163522	+1.35183716	−0.28024856
C	+1.60203015	+2.07440483	−0.15174767
H	+0.60973067	+1.16660924	−0.29817038
C	+3.54340364	+1.47632439	−1.58118077
C	+5.00788216	+1.09953633	−1.49917015
C	+5.17101810	−0.20009319	−0.73971248
C	+4.66113501	−0.02208987	+0.67250706
C	−1.34399394	−0.08576962	−0.67656452
C	−2.24431970	−1.17578762	−0.08289822

C	−2.17230148	−1.22770612	+1.44867056
C	−1.37509494	−0.02423311	+1.98995075
C	−3.49784815	−1.00761132	+2.18499225
C	−3.06364247	−0.60204701	+3.60271526
C	−1.62473669	−0.06450750	+3.48638738
O	+4.50503730	−1.25924645	−1.39033346
H	+3.57756544	−1.22041069	−1.15659517
C	−1.52003438	−2.53494911	+1.88053666
C	−4.47961638	−2.15691346	+2.15094710
H	+6.21980106	−0.48841938	−0.71637797
H	+5.30224630	+0.69757101	+1.18560175
H	+4.75319289	−0.96709099	+1.20228354
H	+5.57096090	+1.87855932	−0.98436908
H	+5.42414270	+0.99980552	−2.49931608
H	+3.05974049	+0.82604693	−2.31669969
H	+3.43574753	+2.48761310	−1.96758744
H	+1.41282941	+2.85174205	−0.88148034
H	+1.26949408	+2.30263730	+0.85618243
H	+3.14102086	−0.31860332	+2.63350018
H	+0.98193218	−0.15964028	+3.24330570
H	+0.82790830	−0.53255787	−0.46707057
H	−1.22242660	−0.24226894	−1.74574842
H	−1.84918351	+0.87550237	−0.56970451
H	−3.26968181	−1.00504352	−0.41395722
H	−1.94841939	−2.14009464	−0.49365829
H	−3.97052596	−0.14321402	+1.70999118
H	−3.09837539	−1.46701975	+4.26462331
H	−3.74357454	+0.13417155	+4.02543130
H	−0.92839662	−0.74061316	+3.97877411
H	−1.49792501	+0.91066914	+3.95013823
H	−1.49374386	−2.65078814	+2.96277844
H	−0.49688029	−2.58893899	+1.51276467
H	−2.06553471	−3.38323331	+1.47148307
H	−5.40967657	−1.88838355	+2.64835944
H	−4.07858264	−3.03262388	+2.65838907
H	−4.72315712	−2.44339222	+1.12849093
H	−1.89663605	+0.86140012	+1.60697983

51

* E = +3.579 kcal/mol ; (16) 330_050_299_313_042_008_191

C	+0.00000000	+0.00000000	+0.00000000

C	+0.00000000	+0.00000000	+1.40978989
C	+1.14849776	+0.00000000	+2.18516626
C	+2.50004831	+0.05190396	+1.83220548
C	+3.20971363	+0.45052631	+0.69635871
C	+2.78201276	+1.35300861	−0.28447600
C	+1.59526517	+2.07816925	−0.14545626
H	+0.60215712	+1.16979552	−0.29822778
C	+3.53684128	+1.48715430	−1.57890279
C	+5.01684947	+1.19405623	−1.45862449
C	+5.22129739	−0.12199350	−0.75061394
C	+4.63922142	−0.05188392	+0.64338490
C	−1.34507477	−0.08838839	−0.67486615
C	−2.24338972	−1.17850838	−0.07868492
C	−2.17450213	−1.22406484	+1.45337075
C	−1.37504939	−0.02046178	+1.99054548
C	−3.50053247	−0.99790837	+2.18688896
C	−3.06712347	−0.58702839	+3.60349981
C	−1.62614327	−0.05496987	+3.48680352
O	+4.58538632	−1.10666804	−1.53601666
H	+4.54737090	−1.92532041	−1.04687670
C	−1.52609240	−2.53103686	+1.89184064
C	−4.48496355	−2.14508682	+2.15685931
H	+6.28974629	−0.34306852	−0.67904100
H	+5.27757763	+0.60425859	+1.24019619
H	+4.70492692	−1.03611137	+1.10804919
H	+5.51876488	+1.97648547	−0.88973296
H	+5.47514459	+1.15748115	−2.44475450
H	+3.11022481	+0.78282412	−2.29619198
H	+3.38487164	+2.48387051	−1.98771054
H	+1.40664009	+2.86359368	−0.86658546
H	+1.26311754	+2.29583018	+0.86501021
H	+3.14457199	−0.32832932	+2.61940488
H	+0.98543402	−0.15557403	+3.24366505
H	+0.83004066	−0.52731175	−0.46851833
H	−1.22376362	−0.24753607	−1.74371550
H	−1.85256162	+0.87206188	−0.57027734
H	−3.26893688	−1.01336538	−0.41244000
H	−1.94291276	−2.14351413	−0.48466001
H	−3.97070696	−0.13463577	+1.70742165
H	−3.10628529	−1.44871385	+4.26957322

```
H   −3.74521115   +0.15367023   +4.02151510
H   −0.93226777   −0.73162273   +3.98204619
H   −1.49643586   +0.92136056   +3.94740415
H   −1.50376234   −2.64343988   +2.97462033
H   −0.50177336   −2.58627259   +1.52749944
H   −2.07144506   −3.37986646   +1.48346326
H   −5.41515996   −1.87240419   +2.65189148
H   −4.08640566   −3.01943073   +2.66870097
H   −4.72769674   −2.43562091   +1.13532919
H   −1.89571480   +0.86449923   +1.60464710
51
 * E =  +3.622 kcal/mol ; (17) 007_315_066_313_042_008_296
C   +0.00000000   +0.00000000   +0.00000000
C   +0.00000000   +0.00000000   +1.41263497
C   +1.14804930   +0.00000000   +2.18729024
C   +2.50014200   +0.05147278   +1.83819400
C   +3.21556178   +0.43926983   +0.69990520
C   +2.79176797   +1.33968567   −0.28454075
C   +1.60992766   +2.07142198   −0.15703491
H   +0.59983101   +1.14424082   −0.30175791
C   +3.58833471   +1.54788643   −1.54769075
C   +4.75945554   +0.60290692   −1.71013582
C   +5.50948295   +0.52087102   −0.40324880
C   +4.61791727   −0.12778594   +0.62941879
C   −1.34790628   −0.08638489   −0.67149013
C   −2.24585462   −1.17636909   −0.07508336
C   −2.17354496   −1.22372788   +1.45640213
C   −1.37423372   −0.01983580   +1.99418258
C   −3.49869339   −0.99969293   +2.19214852
C   −3.06383477   −0.59045862   +3.60859664
C   −1.62367940   −0.05655671   +3.49078089
O   +6.73944001   −0.15131907   −0.51610199
H   +6.57215667   −1.05912367   −0.76417868
C   −1.52307576   −2.53054850   +1.89224806
C   −4.48195612   −2.14786345   +2.16156922
H   +5.77053051   +1.52980436   −0.08280441
H   +5.08606019   −0.06422432   +1.61111509
H   +4.56222116   −1.19480844   +0.39116596
H   +5.42254052   +0.94340332   −2.50250546
H   +4.40936472   −0.39697207   −1.98053779
```

H	+2.91498897	+1.46902455	−2.40185425
H	+3.95780741	+2.57526387	−1.55837139
H	+1.42392224	+2.84808621	−0.88890551
H	+1.26491680	+2.29518239	+0.84714178
H	+3.14346416	−0.31840933	+2.63024563
H	+0.98375548	−0.14745423	+3.24664760
H	+0.81988043	−0.54873217	−0.46250858
H	−1.22984244	−0.24347279	−1.74114520
H	−1.85307486	+0.87480334	−0.56391035
H	−3.27184777	−1.00969022	−0.40649886
H	−1.94738316	−2.14128078	−0.48287478
H	−3.97018636	−0.13613936	+1.71442731
H	−3.10096700	−1.45321968	+4.27331304
H	−3.74217401	+0.14872536	+4.02873611
H	−0.92858759	−0.73311942	+3.98429015
H	−1.49459596	+0.91926958	+3.95252548
H	−1.49625058	−2.64270015	+2.97488879
H	−0.50014695	−2.58628541	+1.52407641
H	−2.07004788	−3.37940655	+1.48618469
H	−5.41173278	−1.87680164	+2.65816826
H	−4.08187512	−3.02248882	+2.67165374
H	−4.72583396	−2.43716425	+1.13996358
H	−1.89522208	+0.86553732	+1.60983441

51

* E = +3.976 kcal/mol ; (18) 044_313_358_313_042_008_284

C	+0.00000000	+0.00000000	+0.00000000
C	+0.00000000	+0.00000000	+1.40827179
C	+1.14331791	+0.00000000	+2.19433021
C	+2.49653579	+0.07928772	+1.85704025
C	+3.19134483	+0.54296289	+0.73720536
C	+2.75001055	+1.43062866	−0.24915196
C	+1.53972837	+2.12613902	−0.17542122
H	+0.58544673	+1.18963570	−0.31382483
C	+3.65378978	+1.58214450	−1.43640405
C	+4.18542647	+0.23996306	−1.92612247
C	+4.71674186	−0.64422351	−0.78662880
C	+4.61462741	+0.08767388	+0.54509961
C	−1.34122077	−0.11320218	−0.67786935
C	−2.22140117	−1.21782042	−0.08152925
C	−2.15908921	−1.25405957	+1.45083716

C	−1.37703486	−0.03749814	+1.98521780
C	−3.49124548	−1.03897365	+2.17676166
C	−3.06967634	−0.61426885	+3.59281554
C	−1.63390625	−0.06741778	+3.48061704
O	+4.03422721	−1.87473479	−0.72107032
H	+3.17617833	−1.71616224	−0.32467118
C	−1.49800124	−2.55078180	+1.90053432
C	−4.46133569	−2.19835301	+2.14922201
H	+5.75326421	−0.91410131	−0.97203400
H	+5.29756337	+0.93956967	+0.53213483
H	+4.93046688	−0.57950145	+1.34252249
H	+4.95593129	+0.39955221	−2.67805461
H	+3.38528515	−0.31184363	−2.41650611
H	+3.13422352	+2.10346462	−2.23777162
H	+4.49591286	+2.22243835	−1.15763167
H	+1.36176071	+2.89189067	−0.92032790
H	+1.17542361	+2.36555317	+0.81979237
H	+3.14818330	−0.30708970	+2.63320496
H	+0.97168559	−0.17965608	+3.24737172
H	+0.84088267	−0.50442590	−0.47431940
H	−1.21423002	−0.27185388	−1.74610792
H	−1.86508859	+0.83876032	−0.57613183
H	−3.24797026	−1.07420742	−0.42165062
H	−1.89960380	−2.17835829	−0.48143682
H	−3.96955521	−0.18448175	+1.68960039
H	−3.10287846	−1.47194241	+4.26419512
H	−3.75778036	+0.12176970	+4.00241731
H	−0.93620512	−0.73426601	+3.98343107
H	−1.51687998	+0.91255693	+3.93678140
H	−1.47993824	−2.65660537	+2.98399077
H	−0.47132416	−2.59839039	+1.54184357
H	−2.03143479	−3.40813454	+1.49457327
H	−5.39683418	−1.93397249	+2.63858130
H	−4.05446372	−3.06469522	+2.66797999
H	−4.69621197	−2.49793000	+1.12850239
H	−1.90724992	+0.83902356	+1.59377298

51

* E = +4.261 kcal/mol ; (19) 329_052_299_313_042_008_061

C	+0.00000000	+0.00000000	+0.00000000
C	+0.00000000	+0.00000000	+1.40986386

C	+1.14971984	+0.00000000	+2.18351024
C	+2.49969170	+0.04891772	+1.82625000
C	+3.20617125	+0.44553483	+0.68768838
C	+2.77757082	+1.35665237	−0.28365640
C	+1.59049903	+2.08107521	−0.14335820
H	+0.59577928	+1.17021331	−0.29954300
C	+3.54029531	+1.50530636	−1.57138022
C	+5.02479954	+1.25465291	−1.42028681
C	+5.25466424	−0.07593656	−0.73738670
C	+4.63063662	−0.07287806	+0.63536902
C	−1.34534146	−0.09346130	−0.67394299
C	−2.23875906	−1.18730268	−0.07725895
C	−2.16905188	−1.23222877	+1.45479988
C	−1.37456635	−0.02492181	+1.99127554
C	−3.49579063	−1.01155213	+2.18865616
C	−3.06362523	−0.59857230	+3.60503582
C	−1.62501811	−0.06024262	+3.48763075
O	+4.65967703	−1.13187669	−1.46169263
H	+5.05240371	−1.17746039	−2.33080105
C	−1.51459320	−2.53623941	+1.89311847
C	−4.47526245	−2.16299804	+2.15926138
H	+6.32662172	−0.26309084	−0.63242816
H	+5.25510901	+0.54265532	+1.28666749
H	+4.67277063	−1.08714259	+1.02383922
H	+5.48509728	+2.04062086	−0.82068766
H	+5.51387367	+1.27234794	−2.39549052
H	+3.14001190	+0.78797049	−2.29202363
H	+3.36706263	+2.49569267	−1.98687051
H	+1.40343541	+2.87153675	−0.85945019
H	+1.25563373	+2.29223902	+0.86758939
H	+3.14615777	−0.33773299	+2.60855559
H	+0.98886222	−0.15701837	+3.24215349
H	+0.83118304	−0.52783979	−0.46620975
H	−1.22426621	−0.25244386	−1.74293851
H	−1.85678291	+0.86492012	−0.56912689
H	−3.26523277	−1.02674712	−0.41061020
H	−1.93390621	−2.15102076	−0.48293235
H	−3.96991928	−0.15036825	+1.70922823
H	−3.09865807	−1.46041680	+4.27114097
H	−3.74484772	+0.13916324	+4.02329504

H	−0.92798934	−0.73373800	+3.98264701
H	−1.49942291	+0.91675670	+3.94800804
H	−1.49109930	−2.64826267	+2.97592001
H	−0.49038770	−2.58764104	+1.52812535
H	−2.05673233	−3.38747487	+1.48543373
H	−5.40646742	−1.89438275	+2.65469701
H	−4.07248970	−3.03549333	+2.67092928
H	−4.71707487	−2.45477140	+1.13784793
H	−1.89917428	+0.85774030	+1.60531163

51

* E = +4.360 kcal/mol ; (20) 304_039_015_313_042_008_175

C	+0.00000000	+0.00000000	+0.00000000
C	+0.00000000	+0.00000000	+1.40842148
C	+1.14440166	+0.00000000	+2.19280703
C	+2.49640888	+0.06915742	+1.85020115
C	+3.18459179	+0.50994023	+0.71826428
C	+2.74850197	+1.41575563	−0.25296184
C	+1.54493380	+2.12052256	−0.16214583
H	+0.58217515	+1.19041610	−0.31241775
C	+3.64489550	+1.56394626	−1.44577467
C	+5.06372481	+1.95484425	−1.03506040
C	+5.51756690	+1.24056095	+0.23215685
C	+4.61259114	+0.06172425	+0.54835285
C	−1.34246470	−0.11015211	−0.67660732
C	−2.22382076	−1.21321562	−0.08013200
C	−2.16100261	−1.24912549	+1.45245839
C	−1.37657864	−0.03374870	+1.98654579
C	−3.49237756	−1.03287360	+2.17925930
C	−3.06944718	−0.60804599	+3.59503193
C	−1.63214418	−0.06545852	+3.48209159
O	+6.85487071	+0.84208932	+0.02850595
H	+7.19639430	+0.46365386	+0.83577139
C	−1.50068503	−2.54660349	+1.90124293
C	−4.46352995	−2.19145013	+2.15293725
H	+5.46780652	+1.93240508	+1.07678050
H	+4.95647791	−0.44789345	+1.44809046
H	+4.69565844	−0.66006848	−0.26831785
H	+5.12616584	+3.03117006	−0.89511846
H	+5.76454951	+1.69258992	−1.82484107
H	+3.68670329	+0.61312385	−1.98243542

H	+3.24036370	+2.29578393	−2.14020036
H	+1.37043170	+2.90197468	−0.89104361
H	+1.18377341	+2.34158409	+0.83848072
H	+3.14846603	−0.31269661	+2.62870671
H	+0.97388586	−0.16723876	+3.24820556
H	+0.84004243	−0.50874830	−0.46985219
H	−1.21689920	−0.26877894	−1.74510090
H	−1.86527001	+0.84237136	−0.57448105
H	−3.25054561	−1.06885786	−0.41972097
H	−1.90316032	−2.17447374	−0.47942916
H	−3.97052217	−0.17823164	+1.69227076
H	−3.10546534	−1.46475247	+4.26758810
H	−3.75536192	+0.13059342	+4.00372805
H	−0.93567121	−0.73562809	+3.98229258
H	−1.51147602	+0.91321387	+3.94016551
H	−1.48289760	−2.65317229	+2.98465904
H	−0.47405082	−2.59329236	+1.54227560
H	−2.03495846	−3.40330157	+1.49475657
H	−5.39840381	−1.92622193	+2.64312148
H	−4.05688497	−3.05799940	+2.67158386
H	−4.69957805	−2.49109489	+1.13247798
H	−1.90627963	+0.84382290	+1.59661574

51

* E = +4.856 kcal/mol ; (21) 304_040_014_313_042_008_063

C	+0.00000000	+0.00000000	+0.00000000
C	+0.00000000	+0.00000000	+1.40839532
C	+1.14442795	+0.00000000	+2.19280536
C	+2.49661540	+0.06829089	+1.85092123
C	+3.18575801	+0.50850952	+0.71919611
C	+2.74855906	+1.41407422	−0.25130512
C	+1.54529278	+2.11997178	−0.16190945
H	+0.58163901	+1.19090268	−0.31262901
C	+3.64516609	+1.56299631	−1.44356393
C	+5.06230940	+1.95470360	−1.02587961
C	+5.52356028	+1.21729346	+0.23510383
C	+4.61096014	+0.05231058	+0.55194000
C	−1.34239387	−0.11136888	−0.67658871
C	−2.22265422	−1.21528928	−0.08006419
C	−2.15997642	−1.25084550	+1.45255682
C	−1.37664494	−0.03466084	+1.98641273

C	−3.49164957	−1.03557952	+2.17908974
C	−3.06927041	−0.61001042	+3.59480919
C	−1.63237092	−0.06633793	+3.48193322
O	+6.82805437	+0.69846940	+0.10090060
H	+7.44405259	+1.41721526	−0.02074453
C	−1.49855890	−2.54764476	+1.90166758
C	−4.46175276	−2.19504600	+2.15296371
H	+5.49145326	+1.89998258	+1.08766785
H	+4.96585437	−0.45187528	+1.44704027
H	+4.68912705	−0.67145472	−0.26353284
H	+5.11029797	+3.03093865	−0.86864928
H	+5.75455568	+1.71156574	−1.83071863
H	+3.69250215	+0.61209310	−1.97929493
H	+3.24143673	+2.29435932	−2.13918716
H	+1.37191304	+2.90163038	−0.89097273
H	+1.18376066	+2.34175336	+0.83844589
H	+3.14879034	−0.31352041	+2.62895253
H	+0.97370032	−0.16751156	+3.24812525
H	+0.84036501	−0.50844304	−0.46964876
H	−1.21674999	−0.27000154	−1.74510578
H	−1.86620447	+0.84063301	−0.57449365
H	−3.24952844	−1.07218145	−0.41981206
H	−1.90079284	−2.17625172	−0.47910624
H	−3.97051394	−0.18148511	+1.69180523
H	−3.10469976	−1.46656311	+4.26759101
H	−3.75586801	+0.12818952	+4.00319906
H	−0.93541588	−0.73584637	+3.98228195
H	−1.51248744	+0.91249053	+3.93988806
H	−1.48094251	−2.65412020	+2.98508423
H	−0.47176045	−2.59341896	+1.54311391
H	−2.03188237	−3.40490874	+1.49510529
H	−5.39692279	−1.93058799	+2.64302843
H	−4.05429819	−3.06107602	+2.67182963
H	−4.69741109	−2.49518038	+1.13254832
H	−1.90715687	+0.84235793	+1.59627502

51

* E = +5.000 kcal/mol ; (22) 044_306_009_313_042_008_181

C	+0.00000000	+0.00000000	+0.00000000
C	+0.00000000	+0.00000000	+1.40819298
C	+1.14711010	+0.00000000	+2.18832743

C	+2.49542345	+0.07177476	+1.83383818
C	+3.17461651	+0.53051880	+0.70453573
C	+2.73482183	+1.44110868	−0.25918764
C	+1.52546381	+2.13622509	−0.16976235
H	+0.57225461	+1.19342954	−0.31273240
C	+3.62352604	+1.59929913	−1.45735356
C	+4.10790542	+0.24875888	−1.96380152
C	+4.77647034	−0.57573091	−0.86672701
C	+4.59425645	+0.07880064	+0.49878793
C	−1.34263023	−0.11722370	−0.67500640
C	−2.21871392	−1.22473725	−0.07778678
C	−2.15135966	−1.26390885	+1.45432790
C	−1.37518652	−0.04339392	+1.98856109
C	−3.48253640	−1.05967017	+2.18488886
C	−3.05949328	−0.63573410	+3.60060992
C	−1.62839690	−0.07749438	+3.48461692
O	+4.19190618	−1.85783042	−0.91826213
H	+4.56758693	−2.40546580	−0.23212167
C	−1.47918285	−2.55714300	+1.89776182
C	−4.44522890	−2.22523576	+2.15726219
H	+5.84871058	−0.66144170	−1.06202180
H	+5.27582594	+0.93204081	+0.54522201
H	+4.90699591	−0.61265137	+1.28012951
H	+4.77475821	+0.37236626	−2.81413409
H	+3.25259235	−0.32885599	−2.30908863
H	+3.09635736	+2.13372226	−2.24483970
H	+4.48410580	+2.22167732	−1.19296371
H	+1.34290248	+2.91183503	−0.90335846
H	+1.16473775	+2.36158311	+0.83002901
H	+3.15450317	−0.32154924	+2.60088186
H	+0.98401191	−0.17742955	+3.24329243
H	+0.84443934	−0.50397416	−0.46817952
H	−1.21722171	−0.27602567	−1.74348573
H	−1.87050506	+0.83264171	−0.57270538
H	−3.24704233	−1.08277869	−0.41381564
H	−1.89596079	−2.18383273	−0.48031568
H	−3.96790682	−0.20705564	+1.70141693
H	−3.08375674	−1.49580999	+4.26945791
H	−3.75202293	+0.09359985	+4.01495740
H	−0.92388033	−0.73954122	+3.98434473

H	−1.51762199	+0.90266947	+3.94205410
H	−1.45427601	−2.66523900	+2.98098982
H	−0.45457810	−2.59571850	+1.53233282
H	−2.00942383	−3.41709958	+1.49281392
H	−5.38101406	−1.96868993	+2.65041318
H	−4.03070603	−3.09040082	+2.67201805
H	−4.68111691	−2.52335487	+1.13631722
H	−1.91160518	+0.83032336	+1.59904045

51

* E = +5.100 kcal/mol ; (23) 304_036_018_313_042_008_299

C	+0.00000000	+0.00000000	+0.00000000
C	+0.00000000	+0.00000000	+1.40829602
C	+1.14456130	+0.00000000	+2.19250645
C	+2.49666594	+0.06937163	+1.85081621
C	+3.18536183	+0.50849394	+0.71837446
C	+2.74899630	+1.41274474	−0.25396475
C	+1.54617027	+2.11925602	−0.16372161
H	+0.58229760	+1.19093633	−0.31210636
C	+3.63543710	+1.54955977	−1.45620084
C	+5.06841220	+1.91836127	−1.06880242
C	+5.51212132	+1.24924414	+0.23264538
C	+4.61717541	+0.06677676	+0.56030248
C	−1.34229021	−0.10924856	−0.67697754
C	−2.22450178	−1.21189616	−0.08092799
C	−2.16114665	−1.24884209	+1.45155112
C	−1.37656731	−0.03383567	+1.98642261
C	−3.49232495	−1.03307377	+2.17887099
C	−3.06895999	−0.60962285	+3.59488992
C	−1.63186116	−0.06652555	+3.48199125
O	+6.87452562	+0.89832560	+0.21286595
H	+7.02341645	+0.27454919	−0.49562226
C	−1.50075120	−2.54662935	+1.89931887
C	−4.46366289	−2.19147775	+2.15169078
H	+5.42805791	+1.95841198	+1.05281022
H	+4.97485891	−0.41920100	+1.46467187
H	+4.69526960	−0.67289034	−0.24487625
H	+5.17034011	+2.99729586	−0.98073213
H	+5.74654137	+1.60975527	−1.86508961
H	+3.65316669	+0.59630870	−1.99079691
H	+3.23106135	+2.28386923	−2.14813031

H	+1.37182877	+2.89943127	−0.89401717
H	+1.18728559	+2.34374142	+0.83692199
H	+3.14907680	−0.30841669	+2.63057314
H	+0.97399520	−0.16643485	+3.24797552
H	+0.84029362	−0.50855702	−0.46967695
H	−1.21675788	−0.26750327	−1.74555827
H	−1.86447817	+0.84355830	−0.57448339
H	−3.25116719	−1.06615583	−0.42007332
H	−1.90505428	−2.17325146	−0.48098152
H	−3.97044825	−0.17789707	+1.69277523
H	−3.10443269	−1.46702956	+4.26655866
H	−3.75492583	+0.12836375	+4.00463962
H	−0.93505558	−0.73664036	+3.98174568
H	−1.51141484	+0.91190341	+3.94058228
H	−1.48224460	−2.65352919	+2.98265994
H	−0.47430505	−2.59345640	+1.53982221
H	−2.03548010	−3.40306188	+1.49286305
H	−5.39833759	−1.92650964	+2.64237646
H	−4.05708088	−3.05857154	+2.66946160
H	−4.70008727	−2.49014429	+1.13102015
H	−1.90609589	+0.84409558	+1.59703036

51

* E = +5.745 kcal/mol ; (24) 042_300_020_313_042_008_055

C	+0.00000000	+0.00000000	+0.00000000
C	+0.00000000	+0.00000000	+1.40789970
C	+1.14655577	+0.00000000	+2.18885203
C	+2.49621053	+0.07394521	+1.83957661
C	+3.18086589	+0.53591578	+0.71500862
C	+2.73717617	+1.44485724	−0.24891192
C	+1.52536983	+2.13680281	−0.16436404
H	+0.57352097	+1.19735965	−0.31243099
C	+3.61562534	+1.59154051	−1.45728254
C	+4.10338476	+0.22532874	−1.91354115
C	+4.91135643	−0.47265351	−0.82049263
C	+4.62107811	+0.12655772	+0.54786598
C	−1.34171380	−0.12173277	−0.67615929
C	−2.21543028	−1.23020660	−0.07718743
C	−2.15110991	−1.26424682	+1.45526255
C	−1.37585053	−0.04222443	+1.98680205
C	−3.48369067	−1.05742878	+2.18259667

C	−3.06292049	−0.62857053	+3.59769394
C	−1.63109252	−0.07200975	+3.48250124
O	+4.61360243	−1.84938420	−0.74969302
H	+4.74355046	−2.24498155	−1.60870686
C	−1.47993018	−2.55574313	+1.90519889
C	−4.44621568	−2.22320536	+2.15772684
H	+5.97980213	−0.34950037	−1.01724116
H	+5.26187984	+1.00321930	+0.66363341
H	+4.92159524	−0.59569148	+1.30133335
H	+4.68077820	+0.30074356	−2.83428714
H	+3.23107522	−0.39437318	−2.12243869
H	+3.07261455	+2.09561918	−2.25402999
H	+4.47481740	+2.22737086	−1.22203729
H	+1.34511308	+2.91228683	−0.89886731
H	+1.16267537	+2.36336202	+0.83451391
H	+3.15175295	−0.32098178	+2.60796790
H	+0.98148346	−0.18012484	+3.24301666
H	+0.84600319	−0.50162164	−0.46747557
H	−1.21550246	−0.28232353	−1.74442780
H	−1.87217556	+0.82694208	−0.57653050
H	−3.24351598	−1.09320802	−0.41602147
H	−1.88843280	−2.18972389	−0.47554112
H	−3.96846543	−0.20657699	+1.69539468
H	−3.08917807	−1.48604994	+4.26977702
H	−3.75563103	+0.10298973	+4.00780788
H	−0.92784555	−0.73333882	+3.98478514
H	−1.52009814	+0.90933734	+3.93729130
H	−1.46021024	−2.66020770	+2.98883514
H	−0.45330014	−2.59508691	+1.54552009
H	−2.00791795	−3.41735860	+1.50077347
H	−5.38299587	−1.96470215	+2.64796430
H	−4.03264150	−3.08612988	+2.67692362
H	−4.68010549	−2.52560949	+1.13756093
H	−1.91174542	+0.83054120	+1.59439628

S3.3. Cartesian Coordinates of TS(−) Conformers

```
51
 * E =  +0.000  kcal/mol ; (1) 055_326_338_357_328_062_290
 C    +0.00000000    +0.00000000    +0.00000000
 C    +0.00000000    +0.00000000    +1.41005690
 C    +1.16787057    +0.00000000    +2.16206706
 C    +2.51453071    −0.05098181    +1.79173410
 C    +3.18225990    −0.49342619    +0.65053439
 C    +2.72926130    −1.42149391    −0.29876780
 C    +1.53948002    −2.13421606    −0.16045242
 H    +0.55729160    −1.17771140    −0.27872936
 C    +3.58844198    −1.57203747    −1.51853208
 C    +5.03618125    −1.91834170    −1.15848975
 C    +5.48146564    −1.26752185    +0.15097066
 C    +4.61292370    −0.05657181    +0.45631340
 C    −1.27412383    +0.14345898    −0.81293326
 C    −2.56359311    +0.22232400    +0.00457057
 C    −2.45273177    −0.59134787    +1.27668281
 C    −1.32515612    +0.01292131    +2.11038232
 C    −3.61762992    −0.44107182    +2.25969603
 C    −3.00090283    −0.85568872    +3.60606837
 C    −1.48139880    −0.64584151    +3.47412363
 O    +5.43904732    −2.17860515    +1.22421367
 H    +4.51865773    −2.35979793    +1.42064527
 C    −2.20828707    −2.06190013    +0.95924461
 C    −4.88931754    −1.18349418    +1.91528655
 H    +6.52454871    −0.97043481    +0.08150680
 H    +4.68412716    +0.65307014    −0.37224491
 H    +4.97892339    +0.45175172    +1.34511583
 H    +5.68972413    −1.60963772    −1.97284005
 H    +5.15536412    −2.99342435    −1.04441847
 H    +3.17295875    −2.32138918    −2.18752426
 H    +3.58514743    −0.62656310    −2.06699218
 H    +1.19398568    −2.33073017    +0.85136835
 H    +1.33773730    −2.92074481    −0.87631500
 H    +3.17928595    +0.34194364    +2.55334976
 H    +1.01813219    +0.16025670    +3.22277222
 H    +0.87375399    +0.48190460    −0.43757863
 H    −1.33801450    −0.69911080    −1.50305196
```

H	−1.19404100	+1.02612562	−1.44591206
H	−2.76222028	+1.25875167	+0.28406960
H	−3.40563549	−0.10592535	−0.60586505
H	−3.84745230	+0.62739457	+2.30160099
H	−3.22949142	−1.90096332	+3.81310118
H	−3.42633470	−0.28163069	+4.42598874
H	−0.94615377	−1.59443445	+3.49732488
H	−1.07670559	−0.03709012	+4.27876093
H	−2.20482994	−2.67297902	+1.86002899
H	−1.25681857	−2.21569609	+0.45622715
H	−2.99190928	−2.44381357	+0.30689848
H	−5.67179010	−0.96673080	+2.63990152
H	−4.73139207	−2.26072699	+1.91919908
H	−5.26489129	−0.90178719	+0.93253350
H	−1.60099989	+1.06739930	+2.23627170

51

* E = +0.073 kcal/mol ; (2) 000_039_294_357_328_062_285

C	+0.00000000	+0.00000000	+0.00000000
C	+0.00000000	+0.00000000	+1.41501449
C	+1.17102545	+0.00000000	+2.15666808
C	+2.51755128	−0.03132992	+1.77881265
C	+3.21161280	−0.41714476	+0.62983763
C	+2.77259360	−1.33799104	−0.33678615
C	+1.61069725	−2.08321258	−0.15955400
H	+0.57323485	−1.13224695	−0.27051178
C	+3.52917264	−1.54585424	−1.62769807
C	+4.77254855	−0.69205793	−1.78046459
C	+5.49027156	−0.60007618	−0.44959541
C	+4.60953140	+0.15672601	+0.51719406
C	−1.28185312	+0.11724198	−0.80831271
C	−2.57038784	+0.16672305	+0.01299651
C	−2.43776189	−0.63932392	+1.28791905
C	−1.32334368	−0.00542923	+2.11713356
C	−3.60546987	−0.51132149	+2.27041017
C	−2.97923269	−0.90188246	+3.61949064
C	−1.46502913	−0.65720297	+3.48575331
O	+5.82573881	−1.87688708	+0.04349462
H	+5.03006535	−2.28834054	+0.38241928
C	−2.15743169	−2.10485518	+0.97691125
C	−4.85859943	−1.28690762	+1.93118583

H +6.43742473 −0.07851432 −0.56567272
H +4.55669101 +1.19809911 +0.19611026
H +5.07353367 +0.16167949 +1.50335255
H +4.51197720 +0.31614313 −2.10356948
H +5.42614182 −1.11967572 −2.53731329
H +3.80868831 −2.59762812 −1.69736954
H +2.84830610 −1.36981329 −2.46135333
H +1.28321914 −2.28207085 +0.85610448
H +1.39796010 −2.86511399 −0.87799177
H +3.17173043 +0.35352169 +2.55464310
H +1.02798132 +0.13912956 +3.22132738
H +0.85356548 +0.52380045 −0.43121275
H −1.32939639 −0.72674959 −1.49764358
H −1.22442829 +1.00142276 −1.44137821
H −2.79413167 +1.19874938 +0.28980000
H −3.40585529 −0.18400002 −0.59407564
H −3.86107885 +0.55154469 +2.30544591
H −3.18276785 −1.95089245 +3.83373423
H −3.41820388 −0.33249633 +4.43553916
H −0.90786788 −1.59294814 +3.51544027
H −1.07431484 −0.03320779 +4.28567190
H −2.14013722 −2.71177014 +1.88042699
H −1.20136142 −2.23749134 +0.47620324
H −2.93037725 −2.50893125 +0.32519926
H −5.64592324 −1.08506948 +2.65485327
H −4.67394800 −2.35985129 +1.94181546
H −5.24130184 −1.02081567 +0.94685000
H −1.62088769 +1.04423443 +2.23439656
51
* E = +0.081 kcal/mol ; (3) 000_040_294_309_034_018_285
C +0.00000000 +0.00000000 +0.00000000
C +0.00000000 +0.00000000 +1.40984289
C +1.15457485 +0.00000000 +2.17658610
C +2.50240203 −0.01833236 +1.80987250
C +3.20330742 −0.35678835 +0.64655662
C +2.78767482 −1.25552539 −0.34617039
C +1.64394649 −2.04175389 −0.19020454
H +0.57722173 −1.16578280 −0.30732731
C +3.54829163 −1.40740310 −1.64181930
C +4.76604999 −0.51435783 −1.77300141

C	+5.48506602	−0.44068971	−0.44175510
C	+4.58504863	+0.25829479	+0.55036775
C	−1.33075406	+0.20270745	−0.67326408
C	−2.42740438	−0.73201037	−0.15350640
C	−2.28305493	−1.03830728	+1.34139946
C	−1.36715321	−0.00295957	+2.01337028
C	−3.55315016	−0.84246226	+2.17699112
C	−3.02631611	−0.71839864	+3.61326281
C	−1.56655379	−0.24095008	+3.50066128
O	+5.86219886	−1.72082096	+0.01089948
H	+5.08075790	−2.16746564	+0.33794232
C	−1.72840153	−2.44524817	+1.52167533
C	−4.63468693	−1.88486191	+2.00493135
H	+6.41511691	+0.11350792	−0.54413514
H	+4.50225549	+1.30815501	+0.26528069
H	+5.05079897	+0.24298445	+1.53562784
H	+4.47529747	+0.49488139	−2.06571880
H	+5.42934785	−0.90063695	−2.54351693
H	+3.85724744	−2.44881788	−1.73999940
H	+2.86108551	−1.22948911	−2.47011546
H	+1.34939930	−2.28325721	+0.82595024
H	+1.47396835	−2.82662424	−0.91712698
H	+3.15051497	+0.35056015	+2.59815492
H	+0.99639409	+0.13957099	+3.23850077
H	+0.84291096	+0.51378567	−0.46089274
H	−1.23064906	+0.09894040	−1.75161641
H	−1.63505459	+1.23862703	−0.50009871
H	−3.39949255	−0.28142282	−0.35650883
H	−2.40089404	−1.66408668	−0.71695010
H	−3.96517502	+0.12566592	+1.87894383
H	−3.07652190	−1.68591165	+4.11235146
H	−3.63918891	−0.03852265	+4.20065302
H	−0.88025735	−1.00751097	+3.85792368
H	−1.37171245	+0.65307315	+4.08824213
H	−1.60214895	−2.71276345	+2.56913726
H	−0.76249293	−2.54162978	+1.03283466
H	−2.39601899	−3.17614727	+1.06940444
H	−5.52124855	−1.62219142	+2.57882859
H	−4.30095053	−2.86191647	+2.35000346
H	−4.93369126	−1.98030037	+0.96164263

H	−1.81801221	+0.97005973	+1.77697908

51

* E = +0.245 kcal/mol ; (4) 055_327_338_309_034_018_290

C	+0.00000000	+0.00000000	+0.00000000
C	+0.00000000	+0.00000000	+1.40536874
C	+1.15063792	+0.00000000	+2.18292545
C	+2.49963098	−0.03356402	+1.82521973
C	+3.17914921	−0.42245085	+0.66816969
C	+2.75563163	−1.32721207	−0.31254073
C	+1.58785412	−2.08862501	−0.19762515
H	+0.56793512	−1.21046670	−0.31825736
C	+3.61788092	−1.40950643	−1.53656654
C	+5.07633621	−1.72276748	−1.19061200
C	+5.50240099	−1.10568643	+0.14132745
C	+4.59610642	+0.06508986	+0.49071210
C	−1.32289227	+0.22324003	−0.67943404
C	−2.43876957	−0.68977628	−0.16146544
C	−2.30602590	−0.99471958	+1.33471289
C	−1.36921047	+0.02146765	+2.00631657
C	−3.57335910	−0.76759256	+2.16700265
C	−3.04656328	−0.65292658	+3.60413553
C	−1.57566220	−0.21058419	+3.49350989
O	+5.48991992	−2.05601549	+1.18079058
H	+4.57585692	−2.27585056	+1.36709692
C	−1.78579105	−2.41385513	+1.52144613
C	−4.67796673	−1.78543024	+1.99454878
H	+6.53538553	−0.77306701	+0.08288229
H	+4.64394282	+0.80717213	−0.31070730
H	+4.94683631	+0.55142351	+1.39776206
H	+5.71839723	−1.36436976	−1.99365186
H	+5.22974599	−2.79692873	−1.11542503
H	+3.22456027	−2.14817562	−2.23040228
H	+3.58443424	−0.44675138	−2.05312900
H	+1.27689565	−2.33440056	+0.81411686
H	+1.43266066	−2.87582391	−0.92471257
H	+3.15523774	+0.34363877	+2.60225921
H	+0.98415417	+0.15796203	+3.24093571
H	+0.86012566	+0.47463101	−0.46874237
H	−1.21962575	+0.11364474	−1.75691587
H	−1.61003205	+1.26530036	−0.51200720

H −3.40168601 −0.22233223 −0.36990960
H −2.42642571 −1.62362465 −0.72240955
H −3.96230188 +0.20913079 +1.86600491
H −3.12107435 −1.61761968 +4.10561330
H −3.64406999 +0.04298358 +4.18855992
H −0.90778442 −0.99280933 +3.85182552
H −1.36083332 +0.67896828 +4.08090602
H −1.67075587 −2.68072862 +2.57033868
H −0.82065990 −2.53487135 +1.03697141
H −2.46871538 −3.13006233 +1.06854629
H −5.55946830 −1.50190034 +2.56629537
H −4.36697134 −2.76924841 +2.34164642
H −4.97702017 −1.87573687 +0.95080606
H −1.79974520 +1.00334813 +1.76872397
51
* E = +0.512 kcal/mol ; (5) 029_311_062_358_328_062_183
C +0.00000000 +0.00000000 +0.00000000
C +0.00000000 +0.00000000 +1.41150311
C +1.17085423 +0.00000000 +2.15563321
C +2.51835375 −0.03575715 +1.78186390
C +3.21584512 −0.44240556 +0.64376853
C +2.77146129 −1.35656487 −0.32367850
C +1.59769969 −2.08695731 −0.15097206
H +0.58463560 −1.15532048 −0.26860013
C +3.50159752 −1.49337874 −1.63421629
C +4.97799844 −1.16810817 −1.55137116
C +5.16844971 +0.14976386 −0.85229326
C +4.64641000 +0.05135704 +0.56258536
C −1.27776323 +0.11316499 −0.81232910
C −2.56742914 +0.17351130 +0.00620699
C −2.44092031 −0.62935870 +1.28378703
C −1.32441981 +0.00140846 +2.11294891
C −3.60909547 −0.49339033 +2.26457725
C −2.98606624 −0.88469392 +3.61505145
C −1.47046162 −0.64833135 +3.48204560
O +6.54141368 +0.45674319 −0.87768853
H +6.69205456 +1.26887659 −0.39868146
C −2.16716913 −2.09701141 +0.97688548
C −4.86583302 −1.26314766 +1.92531820
H +4.60464101 +0.91882078 −1.39305194

H	+4.72785064	+1.02252552	+1.05003480
H	+5.30626008	−0.63179203	+1.10299880
H	+5.42047929	−1.12587425	−2.54425902
H	+5.51091688	−1.93549088	−0.98925378
H	+3.36052305	−2.49815306	−2.02640213
H	+3.03338788	−0.81906544	−2.35808910
H	+1.28011909	−2.29006447	+0.86766463
H	+1.38415443	−2.87174024	−0.86542444
H	+3.16948116	+0.35703708	+2.55683388
H	+1.02588028	+0.15067749	+3.21853585
H	+0.86113748	+0.50675712	−0.43600296
H	−1.32658970	−0.73691877	−1.49440833
H	−1.21619184	+0.99136732	−1.45354029
H	−2.78512710	+1.20787870	+0.27918765
H	−3.40396318	−0.17435815	−0.60106624
H	−3.85937994	+0.57081337	+2.29743430
H	−3.19555841	−1.93220285	+3.83101605
H	−3.42279582	−0.31137026	+4.42958876
H	−0.91829230	−1.58695464	+3.51323062
H	−1.07713740	−0.02560398	+4.28171412
H	−2.15815669	−2.70241594	+1.88150720
H	−1.20888567	−2.23520672	+0.48208898
H	−2.93880053	−2.49794904	+0.32160843
H	−5.65272107	−1.05698784	+2.64828651
H	−4.68621656	−2.33694361	+1.93671535
H	−5.24652623	−0.99594616	+0.94049592
H	−1.61827264	+1.05213549	+2.22930151

51

* E = +0.623 kcal/mol ; (6) 030_309_062_358_328_062_060

C	+0.00000000	+0.00000000	+0.00000000
C	+0.00000000	+0.00000000	+1.41148464
C	+1.17106510	+0.00000000	+2.15539780
C	+2.51838720	−0.03409796	+1.78150423
C	+3.21685484	−0.44006906	+0.64362050
C	+2.77176332	−1.35739714	−0.32028556
C	+1.59703265	−2.08690860	−0.14926488
H	+0.58329756	−1.15559730	−0.26943938
C	+3.50810758	−1.50199300	−1.62613085
C	+4.98933413	−1.20501123	−1.51887773
C	+5.19107092	+0.12366466	−0.83209040

C	+4.64566267	+0.05845304	+0.57012504
C	−1.27779008	+0.11442265	−0.81221823
C	−2.56738373	+0.17550661	+0.00644497
C	−2.44130779	−0.62761687	+1.28391147
C	−1.32434201	+0.00228621	+2.11303324
C	−3.60937911	−0.49112072	+2.26475491
C	−2.98650670	−0.88292833	+3.61514865
C	−1.47075446	−0.64751808	+3.48206020
O	+6.54967338	+0.47467110	−0.72364825
H	+6.93131658	+0.52598040	−1.59720138
C	−2.16847462	−2.09539299	+0.97674945
C	−4.86658217	−1.26012242	+1.92546486
H	+4.64978290	+0.89641057	−1.39071874
H	+4.72968953	+1.04145132	+1.02547409
H	+5.29951036	−0.60820594	+1.13745685
H	+5.44158196	−1.19628491	−2.51169940
H	+5.49654027	−1.97538093	−0.93724977
H	+3.35167401	−2.50176304	−2.02525287
H	+3.06366888	−0.81369849	−2.35196380
H	+1.27728471	−2.28755235	+0.86923463
H	+1.38548615	−2.87408818	−0.86170713
H	+3.16933138	+0.36141369	+2.55500688
H	+1.02613265	+0.15142872	+3.21820897
H	+0.86146263	+0.50681040	−0.43540616
H	−1.32750807	−0.73545127	−1.49458113
H	−1.21575331	+0.99289406	−1.45305278
H	−2.78423455	+1.20998384	+0.27965117
H	−3.40429073	−0.17164932	−0.60078319
H	−3.85906251	+0.57321072	+2.29775208
H	−3.19671229	−1.93032173	+3.83106719
H	−3.42281275	−0.30935931	+4.42974744
H	−0.91914432	−1.58647596	+3.51312781
H	−1.07692276	−0.02512555	+4.28170227
H	−2.15987707	−2.70089604	+1.88131582
H	−1.21016638	−2.23396980	+0.48208519
H	−2.94032717	−2.49577628	+0.32135255
H	−5.65334391	−1.05357283	+2.64847292
H	−4.68756838	−2.33403103	+1.93679357
H	−5.24714510	−0.99263969	+0.94065249
H	−1.61746367	+1.05319259	+2.22963403

51

* E = +0.712 kcal/mol ; (7) 028_311_062_309_034_018_183

C	+0.00000000	+0.00000000	+0.00000000
C	+0.00000000	+0.00000000	+1.40664980
C	+1.15363175	+0.00000000	+2.17687511
C	+2.50260844	−0.02119754	+1.81511512
C	+3.20851805	−0.38043517	+0.66270085
C	+2.79057909	−1.27699826	−0.32844356
C	+1.63434963	−2.04852848	−0.17706282
H	+0.58945690	−1.18905901	−0.30535537
C	+3.52995200	−1.36865108	−1.63744104
C	+4.99611113	−1.00328056	−1.54237950
C	+5.14666225	+0.30381745	−0.81447190
C	+4.62346119	+0.15978866	+0.59605290
C	−1.32793564	+0.19951241	−0.67753998
C	−2.42619830	−0.73271231	−0.15615552
C	−2.28638477	−1.03209744	+1.34064463
C	−1.36819445	+0.00328237	+2.00909179
C	−3.55732047	−0.82766954	+2.17284260
C	−3.03238079	−0.69808914	+3.60952854
C	−1.56984499	−0.22930911	+3.49680480
O	+6.51020286	+0.65092326	−0.82815610
H	+6.63565720	+1.45670089	−0.33147381
C	−1.73767696	−2.44030827	+1.52923322
C	−4.64205795	−1.86729109	+2.00398538
H	+4.56252586	+1.06794089	−1.34045177
H	+4.67251862	+1.12307778	+1.10306598
H	+5.30259826	−0.51279762	+1.12566829
H	+5.44044393	−0.92725297	−2.53243009
H	+5.54868194	−1.76723169	−0.99477456
H	+3.41953483	−2.37008714	−2.04773293
H	+3.04490896	−0.69632469	−2.35216204
H	+1.34483608	−2.28803072	+0.84162520
H	+1.46705645	−2.84044091	−0.89631072
H	+3.14663210	+0.35737719	+2.60275404
H	+0.99208382	+0.15010785	+3.23692482
H	+0.84972327	+0.49680543	−0.46636403
H	−1.22528485	+0.09047636	−1.75513973
H	−1.63327780	+1.23642731	−0.51053862
H	−3.39781473	−0.28338869	−0.36431773

H	−2.39747456	−1.66735698	−0.71521373
H	−3.96553870	+0.14031581	+1.86910092
H	−3.08928007	−1.66216981	+4.11453241
H	−3.64252108	−0.01109664	+4.19152263
H	−0.88785292	−0.99879256	+3.85612292
H	−1.37045483	+0.66511858	+4.08231423
H	−1.61829549	−2.70419024	+2.57847317
H	−0.76936801	−2.54162154	+1.04635156
H	−2.40555427	−3.17056064	+1.07620478
H	−5.52904402	−1.59878300	+2.57457333
H	−4.31233198	−2.84370387	+2.35468757
H	−4.93907609	−1.96707568	+0.96051716
H	−1.81667853	+0.97655301	+1.76921965

51

* E = +0.793 kcal/mol ; (8) 030_310_062_358_328_062_297

C	+0.00000000	+0.00000000	+0.00000000
C	+0.00000000	+0.00000000	+1.41139145
C	+1.17100936	+0.00000000	+2.15542553
C	+2.51825705	−0.03636772	+1.78148334
C	+3.21532917	−0.44354128	+0.64318755
C	+2.77019928	−1.35867140	−0.32279474
C	+1.59565402	−2.08823794	−0.15057214
H	+0.58314994	−1.15628942	−0.26850202
C	+3.50103297	−1.49661513	−1.63253872
C	+4.98214960	−1.19266798	−1.53983552
C	+5.19104667	+0.12930993	−0.84276686
C	+4.64460368	+0.05527622	+0.56461160
C	−1.27756613	+0.11416658	−0.81242192
C	−2.56725476	+0.17541379	+0.00603438
C	−2.44138545	−0.62719298	+1.28385532
C	−1.32433683	+0.00276962	+2.11284680
C	−3.60943224	−0.48992413	+2.26463401
C	−2.98677099	−0.88132590	+3.61523315
C	−1.47096356	−0.64619062	+3.48224352
O	+6.53719762	+0.53649656	−0.85078871
H	+7.05319729	−0.09839671	−0.35643473
C	−2.16900126	−2.09519675	+0.97739069
C	−4.86683535	−1.25870025	+1.92562251
H	+4.66183255	+0.90671144	−1.39452315
H	+4.73332302	+1.03401230	+1.02856751

H	+5.28858904	−0.62292226	+1.13582576
H	+5.43281321	−1.16364320	−2.52978212
H	+5.48916629	−1.97701225	−0.97169794
H	+3.34455322	−2.49526929	−2.03457283
H	+3.05012059	−0.80736701	−2.35318226
H	+1.27756458	−2.29058146	+0.86808572
H	+1.38182531	−2.87335815	−0.86456197
H	+3.16962971	+0.35837887	+2.55514350
H	+1.02617851	+0.15228298	+3.21810249
H	+0.86159117	+0.50606405	−0.43580876
H	−1.32691711	−0.73572594	−1.49471730
H	−1.21507773	+0.99248065	−1.45333443
H	−2.78430561	+1.20997936	+0.27865433
H	−3.40394070	−0.17209871	−0.60121960
H	−3.85879928	+0.57448314	+2.29715640
H	−3.19711900	−1.92860610	+3.83151010
H	−3.42303991	−0.30739208	+4.42957355
H	−0.91955116	−1.58526164	+3.51396922
H	−1.07722703	−0.02332749	+4.28158548
H	−2.16022295	−2.70024593	+1.88226212
H	−1.21101730	−2.23437173	+0.48225728
H	−2.94120505	−2.49569266	+0.32252340
H	−5.65356396	−1.05150530	+2.64846057
H	−4.68821464	−2.33266488	+1.93749870
H	−5.24724667	−0.99155127	+0.94068060
H	−1.61693414	+1.05386994	+2.22870662

51

* E = +0.824 kcal/mol ; (9) 029_309_062_309_034_018_060

C	+0.00000000	+0.00000000	+0.00000000
C	+0.00000000	+0.00000000	+1.40660644
C	+1.15384762	+0.00000000	+2.17664833
C	+2.50264263	−0.01962417	+1.81481428
C	+3.20949261	−0.37829044	+0.66266262
C	+2.79079915	−1.27817752	−0.32497998
C	+1.63351688	−2.04864586	−0.17507859
H	+0.58799585	−1.18939103	−0.30611270
C	+3.53685418	−1.37803404	−1.62921284
C	+5.00882277	−1.04271822	−1.50943580
C	+5.17107830	+0.27606865	−0.79427027
C	+4.62256244	+0.16630196	+0.60394342

C	−1.32775634	+0.20144035	−0.67744361
C	−2.42755624	−0.72908053	−0.15615440
C	−2.28753485	−1.02971224	+1.34035617
C	−1.36811674	+0.00423779	+2.00923371
C	−3.55801353	−0.82476192	+2.17311591
C	−3.03247640	−0.69684779	+3.60971175
C	−1.56956553	−0.22926906	+3.49685046
O	+6.51885875	+0.66268050	−0.67258782
H	+6.90179714	+0.74436763	−1.54325397
C	−1.74004548	−2.43857498	+1.52760887
C	−4.64388269	−1.86313348	+2.00376417
H	+4.61045619	+1.04505132	−1.33884240
H	+4.67373664	+1.14255483	+1.07826421
H	+5.29467505	−0.49005126	+1.16182056
H	+5.46472296	−1.00094404	−2.49974557
H	+5.53446195	−1.81095579	−0.94154850
H	+3.41019945	−2.37464017	−2.04653955
H	+3.07623178	−0.69046565	−2.34571806
H	+1.34164108	−2.28528068	+0.84365357
H	+1.46813879	−2.84309271	−0.89200978
H	+3.14647417	+0.36170904	+2.60105035
H	+0.99233195	+0.15084905	+3.23660420
H	+0.85015256	+0.49673530	−0.46575049
H	−1.22545452	+0.09232860	−1.75512348
H	−1.63152372	+1.23879495	−0.51036930
H	−3.39845012	−0.27774954	−0.36345849
H	−2.40097561	−1.66347382	−0.71579774
H	−3.96533628	+0.14388600	+1.87028464
H	−3.09008810	−1.66131144	+4.11393579
H	−3.64181862	−0.00979560	+4.19248337
H	−0.88808271	−0.99956988	+3.85535952
H	−1.36914211	+0.66452962	+4.08291596
H	−1.62005236	−2.70313974	+2.57660429
H	−0.77216583	−2.54034493	+1.04395092
H	−2.40895980	−3.16795417	+1.07464475
H	−5.53042855	−1.59417197	+2.57484208
H	−4.31505636	−2.84016173	+2.35361787
H	−4.94131484	−1.96180755	+0.96029234
H	−1.81569453	+0.97817031	+1.77028071

51

* E = +0.855 kcal/mol ; (10) 352_046_295_357_328_062_190

C	+0.00000000	+0.00000000	+0.00000000
C	+0.00000000	+0.00000000	+1.41469463
C	+1.17119007	+0.00000000	+2.15624031
C	+2.51715698	−0.03179417	+1.77573064
C	+3.21175823	−0.42866086	+0.63135009
C	+2.77627055	−1.35235589	−0.33030268
C	+1.60847376	−2.08783588	−0.15738600
H	+0.57655567	−1.13288267	−0.27249515
C	+3.55257171	−1.58155437	−1.60012114
C	+4.69918794	−0.61746325	−1.81465802
C	+5.48284984	−0.48589554	−0.53159396
C	+4.60325356	+0.15659260	+0.51696835
C	−1.28164764	+0.11852541	−0.80836717
C	−2.56981538	+0.17109440	+0.01320451
C	−2.43859257	−0.63657182	+1.28722989
C	−1.32339243	−0.00515638	+2.11724529
C	−3.60638179	−0.50860696	+2.26949984
C	−2.98084416	−0.90197176	+3.61812629
C	−1.46611483	−0.66013906	+3.48426365
O	+5.88845041	−1.78712390	−0.17021880
H	+6.25180173	−1.77240948	+0.71198455
C	−2.15975907	−2.10199220	+0.97422939
C	−4.86032199	−1.28232906	+1.92888597
H	+6.36293238	+0.14284119	−0.69040553
H	+4.53511384	+1.22064307	+0.28383814
H	+5.09188088	+0.10520592	+1.49342488
H	+4.33320899	+0.36970653	−2.09710122
H	+5.34308884	−0.97193639	−2.61657960
H	+3.95158483	−2.59461380	−1.57494194
H	+2.86413775	−1.54037589	−2.44474362
H	+1.27745235	−2.28403541	+0.85775650
H	+1.39809272	−2.87228210	−0.87378113
H	+3.17068930	+0.36228352	+2.54855665
H	+1.02912645	+0.14186370	+3.22081387
H	+0.85432516	+0.52252171	−0.43131150
H	−1.33125818	−0.72648801	−1.49651095
H	−1.22384820	+1.00175237	−1.44286109
H	−2.79043027	+1.20350619	+0.29145638
H	−3.40679978	−0.17670710	−0.59366614

H	−3.86085230	+0.55450477	+2.30608064
H	−3.18656313	−1.95086999	+3.83111041
H	−3.41868147	−0.33262254	+4.43491975
H	−0.91081887	−1.59707874	+3.51085516
H	−1.07360939	−0.03917924	+4.28572632
H	−2.14542130	−2.71053923	+1.87674578
H	−1.20256183	−2.23470414	+0.47580068
H	−2.93190300	−2.50377046	+0.32003766
H	−5.64763874	−1.08122030	+2.65285781
H	−4.67644216	−2.35544496	+1.93736103
H	−5.24256083	−1.01410025	+0.94492420
H	−1.62072569	+1.04434452	+2.23723880

51

* E = +0.892 kcal/mol ; (11) 352_046_295_309_034_018_190

C	+0.00000000	+0.00000000	+0.00000000
C	+0.00000000	+0.00000000	+1.40962559
C	+1.15430314	+0.00000000	+2.17685052
C	+2.50209100	−0.01874435	+1.80896525
C	+3.20499723	−0.36747094	+0.65055755
C	+2.79321240	−1.26729132	−0.33917378
C	+1.64426321	−2.04512625	−0.18800071
H	+0.58134020	−1.16612611	−0.30908209
C	+3.57437629	−1.43899033	−1.61454903
C	+4.70175008	−0.44606684	−1.79710196
C	+5.47895580	−0.33634960	−0.50807631
C	+4.58240401	+0.25388017	+0.55655716
C	−1.33061809	+0.20578572	−0.67287696
C	−2.42829328	−0.72856466	−0.15436144
C	−2.28376910	−1.03725450	+1.34003880
C	−1.36744419	−0.00308546	+2.01310538
C	−3.55377442	−0.84261158	+2.17595493
C	−3.02677249	−0.72112726	+3.61236209
C	−1.56709010	−0.24328452	+3.50009046
O	+5.91237717	−1.63877064	−0.18542032
H	+6.27064678	−1.64377051	+0.69897291
C	−1.72906890	−2.44448286	+1.51764849
C	−4.63566032	−1.88440339	+2.00222405
H	+6.34521335	+0.31635852	−0.64463058
H	+4.48961358	+1.32272940	+0.35609075
H	+5.06986384	+0.18423646	+1.53248262

H	+4.31644405	+0.54133018	−2.05162113
H	+5.35499205	−0.76357073	−2.60691845
H	+3.99220827	−2.44482289	−1.62276031
H	+2.88501684	−1.38368096	−2.45789513
H	+1.34621770	−2.28493124	+0.82759981
H	+1.47726788	−2.83198941	−0.91348524
H	+3.14863179	+0.35795301	+2.59588776
H	+0.99624547	+0.14139326	+3.23866603
H	+0.84366948	+0.51259897	−0.46088501
H	−1.23077350	+0.10233690	−1.75138489
H	−1.63510386	+1.24171372	−0.49932776
H	−3.40023408	−0.27688129	−0.35625274
H	−2.40268768	−1.65994837	−0.71903652
H	−3.96561696	+0.12616371	+1.87966025
H	−3.07673892	−1.68973990	+4.10946444
H	−3.63975826	−0.04254283	+4.20127685
H	−0.88044767	−1.01020027	+3.85594049
H	−1.37230288	+0.64990882	+4.08905871
H	−1.60232637	−2.71385445	+2.56462999
H	−0.76332085	−2.53938029	+1.02838095
H	−2.39686935	−3.17463321	+1.06429032
H	−5.52228986	−1.62267181	+2.57656873
H	−4.30197334	−2.86214861	+2.34543581
H	−4.93448326	−1.97797006	+0.95869361
H	−1.81863785	+0.97027847	+1.77835629

51

* E = +0.997 kcal/mol ; (12) 029_310_062_309_034_018_297

C	+0.00000000	+0.00000000	+0.00000000
C	+0.00000000	+0.00000000	+1.40655368
C	+1.15373627	+0.00000000	+2.17673994
C	+2.50252051	−0.02187120	+1.81496457
C	+3.20821345	−0.38178629	+0.66251643
C	+2.78965412	−1.27936132	−0.32728080
C	+1.63245083	−2.04985298	−0.17650451
H	+0.58817799	−1.18996602	−0.30526837
C	+3.53014875	−1.37244607	−1.63528465
C	+5.00160660	−1.02860042	−1.53024323
C	+5.17044625	+0.28327927	−0.80433775
C	+4.62175487	+0.16326256	+0.59895594
C	−1.32763879	+0.20060740	−0.67769887

C	−2.42728056	−0.72980078	−0.15596679
C	−2.28766644	−1.02927118	+1.34082209
C	−1.36805517	+0.00487544	+2.00915293
C	−3.55819204	−0.82309651	+2.17321826
C	−3.03290823	−0.69419332	+3.60982599
C	−1.56973600	−0.22742005	+3.49690436
O	+6.50427823	+0.72917491	−0.79833087
H	+7.03688238	+0.09870385	−0.31608347
C	−1.74081258	−2.43820979	+1.52946144
C	−4.64441851	−1.86118520	+2.00452324
H	+4.62089780	+1.05696835	−1.34123792
H	+4.67784634	+1.13481665	+1.08265091
H	+5.28453276	−0.50546338	+1.15966435
H	+5.45490139	−0.96587855	−2.51743218
H	+5.52839042	−1.81030039	−0.97665848
H	+3.40424166	−2.36811293	−2.05521985
H	+3.06226854	−0.68484540	−2.34675592
H	+1.34211669	−2.28839207	+0.84220547
H	+1.46488485	−2.84220716	−0.89520214
H	+3.14663865	+0.35874219	+2.60143862
H	+0.99225531	+0.15173368	+3.23655120
H	+0.85002523	+0.49626659	−0.46626430
H	−1.22503389	+0.09110922	−1.75524612
H	−1.63131177	+1.23804476	−0.51116135
H	−3.39817062	−0.27886753	−0.36395999
H	−2.40030652	−1.66456377	−0.71493965
H	−3.96506636	+0.14544038	+1.86949326
H	−3.09110647	−1.65815797	+4.11491955
H	−3.64198632	−0.00628866	+4.19183242
H	−0.88874674	−0.99786403	+3.85610629
H	−1.36901086	+0.66673992	+4.08232366
H	−1.62137361	−2.70192425	+2.57874017
H	−0.77283349	−2.54094797	+1.04616517
H	−2.40988023	−3.16766315	+1.07689720
H	−5.53094648	−1.59134314	+2.57518263
H	−4.31610040	−2.83805839	+2.35528326
H	−4.94171113	−1.96059352	+0.96109855
H	−1.81512606	+0.97877069	+1.76930538

51

* E = +1.503 kcal/mol ; (13) 317_057_346_357_328_062_182

C	+0.00000000	+0.00000000	+0.00000000
C	+0.00000000	+0.00000000	+1.40973032
C	+1.16831466	+0.00000000	+2.16087744
C	+2.51449692	−0.05725710	+1.78967360
C	+3.18536776	−0.52390597	+0.66071391
C	+2.73005899	−1.44334466	−0.29254794
C	+1.53119375	−2.14228084	−0.16888101
H	+0.55845441	−1.17838085	−0.28198749
C	+3.59651686	−1.59770465	−1.50755346
C	+4.07162723	−0.23999394	−2.00073200
C	+4.82624310	+0.52784427	−0.92099032
C	+4.61543584	−0.10117318	+0.45309670
C	−1.27412885	+0.14625866	−0.81263289
C	−2.56311223	+0.22861151	+0.00531809
C	−2.45393634	−0.58516349	+1.27747282
C	−1.32501161	+0.01717439	+2.11064841
C	−3.61857859	−0.43261676	+2.26039073
C	−3.00245775	−0.84686106	+3.60722617
C	−1.48240238	−0.64175247	+3.47408669
O	+6.19429044	+0.50825847	−1.27175290
H	+6.69903222	+0.95236262	−0.59339725
C	−2.21149968	−2.05610112	+0.96000835
C	−4.89127386	−1.17368576	+1.91666636
H	+4.47504163	+1.56171098	−0.89901726
H	+4.93540902	+0.58688074	+1.23360312
H	+5.27391445	−0.97193101	+0.49550327
H	+3.20482006	+0.33535365	−2.32062175
H	+4.73075516	−0.33898375	−2.85997245
H	+4.46402174	−2.21686927	−1.26364892
H	+3.05454068	−2.12550205	−2.28939695
H	+1.18396188	−2.35044703	+0.83997256
H	+1.32585914	−2.91891528	−0.89491631
H	+3.17737530	+0.34624364	+2.54790616
H	+1.02012620	+0.17107323	+3.22027244
H	+0.87557240	+0.47912122	−0.43769403
H	−1.34083178	−0.69675830	−1.50213242
H	−1.19317236	+1.02826204	−1.44647854
H	−2.75886447	+1.26556570	+0.28504611
H	−3.40641393	−0.09745571	−0.60470134
H	−3.84702835	+0.63619651	+2.30129737

H	−3.23418958	−1.89109215	+3.81618400
H	−3.42587169	−0.27002294	+4.42632085
H	−0.95016937	−1.59207913	+3.49549721
H	−1.07490009	−0.03500070	+4.27886930
H	−2.20788396	−2.66722485	+1.86084345
H	−1.26037835	−2.21048841	+0.45644131
H	−2.99600650	−2.43726748	+0.30823226
H	−5.67361510	−0.95521561	+2.64097051
H	−4.73464378	−2.25111210	+1.92160965
H	−5.26626444	−0.89251316	+0.93352730
H	−1.59917635	+1.07209908	+2.23689622

51

* E = +1.506 kcal/mol ; (14) 350_047_296_357_328_062_060

C	+0.00000000	+0.00000000	+0.00000000
C	+0.00000000	+0.00000000	+1.41473142
C	+1.17146434	+0.00000000	+2.15594902
C	+2.51735866	−0.03058775	+1.77600937
C	+3.21332980	−0.42584772	+0.63184244
C	+2.77659712	−1.35008678	−0.32723418
C	+1.60894644	−2.08674617	−0.15773135
H	+0.57494057	−1.13266490	−0.27350984
C	+3.55641275	−1.58002867	−1.59424134
C	+4.67147064	−0.58370133	−1.82217465
C	+5.47826709	−0.43055759	−0.55013569
C	+4.60490380	+0.16241111	+0.52628411
C	−1.28165053	+0.12015582	−0.80827555
C	−2.56972086	+0.17347384	+0.01344287
C	−2.43900766	−0.63494401	+1.28702739
C	−1.32337828	−0.00473271	+2.11735605
C	−3.60674193	−0.50691035	+2.26933203
C	−2.98139752	−0.90152102	+3.61767936
C	−1.46647535	−0.66073978	+3.48385972
O	+5.94455856	−1.67723727	−0.08190948
H	+6.50067789	−2.07686475	−0.74728485
C	−2.16101386	−2.10036773	+0.97325526
C	−4.86113184	−1.27971662	+1.92821384
H	+6.32677729	+0.23752270	−0.71826579
H	+4.52861880	+1.23405902	+0.33907262
H	+5.11357198	+0.05246090	+1.48361646
H	+4.26884895	+0.39240984	−2.09532226

H +5.30499591 −0.90740395 −2.64900287
H +3.97836183 −2.58447326 −1.55472469
H +2.86719011 −1.57300188 −2.43897378
H +1.27719027 −2.28473164 +0.85677529
H +1.40047458 −2.87114333 −0.87491467
H +3.17131670 +0.36126658 +2.54914411
H +1.02943833 +0.14122451 +3.22061870
H +0.85476578 +0.52257467 −0.43071050
H −1.33231599 −0.72453374 −1.49683681
H −1.22331566 +1.00372396 −1.44231303
H −2.78925915 +1.20594124 +0.29235070
H −3.40717390 −0.17318241 −0.59350882
H −3.86061687 +0.55631795 +2.30657446
H −3.18792047 −1.95039223 +3.83008920
H −3.41878298 −0.33231160 +4.43482389
H −0.91180727 −1.59805126 +3.50967719
H −1.07336647 −0.04076615 +4.28574057
H −2.14705942 −2.70928816 +1.87551709
H −1.20371787 −2.23324285 +0.47504363
H −2.93334579 −2.50139878 +0.31876971
H −5.64834676 −1.07867762 +2.65233206
H −4.67779477 −2.35294075 +1.93601721
H −5.24324962 −1.01067202 +0.94440263
H −1.62029994 +1.04480528 +2.23830573
51
* E = +1.568 kcal/mol ; (15) 351_047_296_309_034_018_060
C +0.00000000 +0.00000000 +0.00000000
C +0.00000000 +0.00000000 +1.40966050
C +1.15438961 +0.00000000 +2.17685995
C +2.50232171 −0.01790717 +1.81036982
C +3.20712730 −0.36459781 +0.65250534
C +2.79430930 −1.26424192 −0.33558321
C +1.64568561 −2.04340603 −0.18840366
H +0.58053615 −1.16548728 −0.31001464
C +3.57903543 −1.43590604 −1.60838999
C +4.67937780 −0.41564678 −1.79960360
C +5.47577865 −0.28712410 −0.51836184
C +4.58533240 +0.25772593 +0.56914467
C −1.33069299 +0.20681294 −0.67260434
C −2.42882933 −0.72724612 −0.15444197

C	−2.28391150	−1.03701720	+1.33969473
C	−1.36745551	−0.00325805	+2.01322152
C	−3.55377750	−0.84311518	+2.17596468
C	−3.02650662	−0.72242162	+3.61233948
C	−1.56681456	−0.24465070	+3.50006729
O	+5.96029432	−1.53925988	−0.08462619
H	+6.52642076	−1.90969185	−0.75839786
C	−1.72893571	−2.44427755	+1.51610886
C	−4.63557704	−1.88494376	+2.00181413
H	+6.31357849	+0.40019912	−0.66068211
H	+4.48630351	+1.33228896	+0.41141661
H	+5.09158071	+0.13238325	+1.52587100
H	+4.26277143	+0.56162241	−2.04629897
H	+5.32288059	−0.70441208	−2.63163287
H	+4.01470901	−2.43534927	−1.60512317
H	+2.89022407	−1.40855839	−2.45335057
H	+1.34696381	−2.28562390	+0.82635573
H	+1.48074822	−2.82978144	−0.91507617
H	+3.14897772	+0.35571330	+2.59818596
H	+0.99594013	+0.14049481	+3.23873219
H	+0.84374307	+0.51327279	−0.46030101
H	−1.23126100	+0.10389683	−1.75127381
H	−1.63477813	+1.24275761	−0.49844975
H	−3.40060189	−0.27481922	−0.35565339
H	−2.40404114	−1.65832599	−0.71972591
H	−3.96581929	+0.12578850	+1.88032665
H	−3.07647830	−1.69128216	+4.10897689
H	−3.63938777	−0.04409845	+4.20169256
H	−0.88009424	−1.01179126	+3.85517576
H	−1.37173253	+0.64803643	+4.08967275
H	−1.60170323	−2.71428508	+2.56284438
H	−0.76322077	−2.53848390	+1.02665626
H	−2.39677090	−3.17426520	+1.06246247
H	−5.52211239	−1.62373814	+2.57657098
H	−4.30164379	−2.86285519	+2.34432071
H	−4.93465354	−1.97789641	+0.95827835
H	−1.81885121	+0.97026809	+1.77934651

51

* E = +1.727 kcal/mol ; (16) 318_061_339_357_328_062_067

C	+0.00000000	+0.00000000	+0.00000000

C	+0.00000000	+0.00000000	+1.40956063
C	+1.16834749	+0.00000000	+2.16064414
C	+2.51497994	−0.05637860	+1.79066994
C	+3.18898093	−0.52096757	+0.66260785
C	+2.73260298	−1.44117461	−0.28948815
C	+1.53342004	−2.14030410	−0.16774572
H	+0.56047836	−1.17931866	−0.28161994
C	+3.59203582	−1.58674251	−1.51059611
C	+4.05959637	−0.21623327	−1.97204013
C	+4.90163696	+0.47329617	−0.90018191
C	+4.62663539	−0.11245356	+0.47729889
C	−1.27397992	+0.14486490	−0.81301973
C	−2.56316149	+0.22669588	+0.00469986
C	−2.45354755	−0.58664297	+1.27709890
C	−1.32518581	+0.01667064	+2.11027497
C	−3.61860417	−0.43477872	+2.25961925
C	−3.00248866	−0.84798400	+3.60675792
C	−1.48250688	−0.64209613	+3.47380014
O	+6.28550468	+0.28803253	−1.11509541
H	+6.54414782	+0.74311618	−1.91319582
C	−2.21004185	−2.05749135	+0.95995326
C	−4.89052743	−1.17720876	+1.91588208
H	+4.67655837	+1.54193623	−0.89385115
H	+4.94548080	+0.58657549	+1.24456825
H	+5.27398700	−0.98859497	+0.55516954
H	+3.18282378	+0.39185622	−2.19319580
H	+4.64036373	−0.28630242	−2.89146266
H	+4.46446179	−2.20514361	−1.28236325
H	+3.04332887	−2.10204647	−2.29610810
H	+1.18708092	−2.35097180	+0.84093319
H	+1.32962473	−2.91667732	−0.89461221
H	+3.17653859	+0.34698776	+2.54988847
H	+1.01970780	+0.17096594	+3.22000254
H	+0.87565703	+0.47925362	−0.43742949
H	−1.34017675	−0.69879987	−1.50188658
H	−1.19392837	+1.02658953	−1.44748322
H	−2.75951148	+1.26360790	+0.28417281
H	−3.40624008	−0.10007083	−0.60531789
H	−3.84809061	+0.63382567	+2.30007681
H	−3.23369731	−1.89223564	+3.81624123

H	−3.42637792	−0.27093976	+4.42547041
H	−0.94975916	−1.59211346	+3.49549691
H	−1.07538540	−0.03499356	+4.27847703
H	−2.20597027	−2.66835602	+1.86095902
H	−1.25874784	−2.21131058	+0.45652357
H	−2.99435954	−2.43939306	+0.30833842
H	−5.67330676	−0.95904456	+2.63981738
H	−4.73288872	−2.25449004	+1.92156507
H	−5.26549914	−0.89702037	+0.93243366
H	−1.60017426	+1.07141296	+2.23636278

51

* E = +1.790 kcal/mol ; (17) 317_057_346_309_034_018_182

C	+0.00000000	+0.00000000	+0.00000000
C	+0.00000000	+0.00000000	+1.40535005
C	+1.15020228	+0.00000000	+2.18311878
C	+2.49943917	−0.03860456	+1.82652027
C	+3.18431361	−0.44946492	+0.68130545
C	+2.75978395	−1.34388293	−0.30553986
C	+1.58490171	−2.09494524	−0.20669846
H	+0.57124820	−1.20979666	−0.32132163
C	+3.63236240	−1.42738838	−1.52330817
C	+4.06813695	−0.03941984	−1.96603029
C	+4.79240257	+0.71325844	−0.85532764
C	+4.60137088	+0.02446330	+0.49255333
C	−1.32287702	+0.22806439	−0.67824656
C	−2.44040102	−0.68314225	−0.16060068
C	−2.30872166	−0.98755076	+1.33576531
C	−1.36909246	+0.02650494	+2.00654937
C	−3.57533409	−0.75695227	+2.16797264
C	−3.04794859	−0.64340267	+3.60511763
C	−1.57620444	−0.20404619	+3.49392748
O	+6.16101694	+0.75649891	−1.20165218
H	+6.64760989	+1.19358571	−0.50570678
C	−1.79169253	−2.40782101	+1.52260693
C	−4.68294858	−1.77158163	+1.99596575
H	+4.40403254	+1.73213871	−0.79456184
H	+4.89992964	+0.69120845	+1.29955097
H	+5.28633012	−0.82657253	+0.50162907
H	+3.18606682	+0.51991910	−2.27228433
H	+4.73508233	−0.08862743	−2.82354299

H	+4.51754528	−2.02931269	−1.30017550
H	+3.10737081	−1.94204396	−2.32529921
H	+1.27197830	−2.35534273	+0.80066712
H	+1.42754709	−2.87074022	−0.94590848
H	+3.15155355	+0.34852255	+2.60212538
H	+0.98377026	+0.16746982	+3.23980342
H	+0.86152358	+0.47320296	−0.46828356
H	−1.22101622	+0.11902749	−1.75602255
H	−1.60857831	+1.27043867	−0.50978987
H	−3.40269562	−0.21455767	−0.36968711
H	−2.42886325	−1.61732049	−0.72103804
H	−3.96169449	+0.22083634	+1.86704760
H	−3.12434350	−1.60809218	+4.10639600
H	−3.64401004	+0.05362465	+4.18978325
H	−0.90947483	−0.98705916	+3.85274909
H	−1.35976647	+0.68567152	+4.08058621
H	−1.67584558	−2.67469990	+2.57145606
H	−0.82734551	−2.53082764	+1.03705746
H	−2.47708481	−3.12245422	+1.07080340
H	−5.56379369	−1.48516765	+2.56737010
H	−4.37488149	−2.75613867	+2.34358825
H	−4.98197244	−1.86155087	+0.95217542
H	−1.79737749	+1.00938298	+1.76873148

51

* E = +2.020 kcal/mol ; (18) 318_061_339_309_034_018_067

C	+0.00000000	+0.00000000	+0.00000000
C	+0.00000000	+0.00000000	+1.40522694
C	+1.14990473	+0.00000000	+2.18343657
C	+2.49988759	−0.03779293	+1.82914346
C	+3.18853491	−0.44625356	+0.68532097
C	+2.76300680	−1.34070905	−0.30120434
C	+1.58822766	−2.09255092	−0.20493532
H	+0.57386610	−1.21088277	−0.32085760
C	+3.62781025	−1.41357705	−1.52526298
C	+4.05598767	−0.01340122	−1.93234809
C	+4.87601503	+0.65753574	−0.83233244
C	+4.61454693	+0.01090721	+0.52032425
C	−1.32281954	+0.22732708	−0.67858299
C	−2.43980646	−0.68480284	−0.16128566
C	−2.30791855	−0.98940480	+1.33502544

C	−1.36940252	+0.02548423	+2.00605278
C	−3.57495031	−0.76043841	+2.16697877
C	−3.04798680	−0.64658221	+3.60420539
C	−1.57672135	−0.20549423	+3.49336729
O	+6.26493994	+0.51899724	−1.04979277
H	+6.51260626	+1.01207364	−1.82858076
C	−1.78928970	−2.40915160	+1.52148747
C	−4.68128489	−1.77639889	+1.99451958
H	+4.62128890	+1.71843890	−0.78538577
H	+4.91160744	+0.68834394	+1.31515013
H	+5.28657238	−0.84873613	+0.56584312
H	+3.16238716	+0.57738048	−2.13201186
H	+4.63995699	−0.03099447	−2.85225114
H	+4.51773298	−2.01497818	−1.31974354
H	+3.09481984	−1.91358427	−2.33128063
H	+1.27607568	−2.35609942	+0.80186556
H	+1.43315482	−2.86766564	−0.94546779
H	+3.15008747	+0.34882165	+2.60637556
H	+0.98238593	+0.16721212	+3.23999582
H	+0.86148919	+0.47337318	−0.46817237
H	−1.22083190	+0.11824636	−1.75641422
H	−1.60964616	+1.26944858	−0.51022282
H	−3.40248761	−0.21690702	−0.37028373
H	−2.42750499	−1.61888983	−0.72189772
H	−3.96236767	+0.21698141	+1.86616050
H	−3.12339649	−1.61145115	+4.10530202
H	−3.64497583	+0.04963832	+4.18890565
H	−0.90911193	−0.98766430	+3.85230242
H	−1.36141241	+0.68440168	+4.08013779
H	−1.67314104	−2.67621400	+2.57025514
H	−0.82471228	−2.53069545	+1.03605903
H	−2.47379279	−3.12450137	+1.06942101
H	−5.56258436	−1.49134776	+2.56592043
H	−4.37188480	−2.76063963	+2.34186828
H	−4.98003341	−1.86647831	+0.95064287
H	−1.79867882	+1.00800100	+1.76842144

51

* E = +2.213 kcal/mol ; (19) 056_320_346_357_328_062_190

C	+0.00000000	+0.00000000	+0.00000000
C	+0.00000000	+0.00000000	+1.41016597

C	+1.16733222	+0.00000000	+2.16230560
C	+2.51468386	−0.05292971	+1.79305554
C	+3.18558317	−0.50395882	+0.65901275
C	+2.73386654	−1.42457207	−0.29346988
C	+1.54235768	−2.13290408	−0.16199181
H	+0.56140971	−1.17679089	−0.28120711
C	+3.60908444	−1.58844203	−1.49797199
C	+5.03459251	−1.97539528	−1.10536775
C	+5.52281531	−1.23075692	+0.13268697
C	+4.61001927	−0.05908999	+0.46030496
C	−1.27445738	+0.14261305	−0.81266306
C	−2.56396896	+0.21979322	+0.00492089
C	−2.45194505	−0.59465531	+1.27640829
C	−1.32550890	+0.01117020	+2.11053080
C	−3.61754410	−0.44748968	+2.25898353
C	−3.00042655	−0.86192451	+3.60522692
C	−1.48122268	−0.64970953	+3.47334251
O	+5.54126689	−2.16880244	+1.18641856
H	+5.68370027	−1.71454466	+2.01431824
C	−2.20445450	−2.06443969	+0.95766228
C	−4.88750278	−1.19243830	+1.91351310
H	+6.53613443	−0.85677272	−0.03304343
H	+4.67176167	+0.66019052	−0.36107304
H	+4.97106380	+0.46746173	+1.34453066
H	+5.70178908	−1.79385467	−1.94540850
H	+5.08371728	−3.03669458	−0.87527690
H	+3.19351100	−2.33007293	−2.17565796
H	+3.64361306	−0.64450835	−2.04895336
H	+1.19843024	−2.33243890	+0.84963778
H	+1.34321278	−2.91921800	−0.87887566
H	+3.17541260	+0.34721519	+2.55542936
H	+1.01708836	+0.16146278	+3.22290574
H	+0.87312102	+0.48312664	−0.43757247
H	−1.33805432	−0.70016572	−1.50273652
H	−1.19633792	+1.02542120	−1.44584119
H	−2.76351181	+1.25583630	+0.28553273
H	−3.40603506	−0.10883884	−0.60548029
H	−3.84969499	+0.62047746	+2.30164248
H	−3.22751742	−1.90772799	+3.81151236
H	−3.42701932	−0.28899777	+4.42545207

H	−0.94444206	−1.59743819	+3.49479313
H	−1.07730304	−0.04155287	+4.27890528
H	−2.20141532	−2.67657780	+1.85776313
H	−1.25162827	−2.21571010	+0.45657957
H	−2.98643014	−2.44692330	+0.30355649
H	−5.67080861	−0.97848874	+2.63815656
H	−4.72675545	−2.26927406	+1.91606869
H	−5.26335203	−0.91057659	+0.93087915
H	−1.60413610	+1.06484709	+2.23777232

51

* E = +2.232 kcal/mol ; (20) 318_058_344_357_328_062_292

C	+0.00000000	+0.00000000	+0.00000000
C	+0.00000000	+0.00000000	+1.40968237
C	+1.16845492	+0.00000000	+2.16070436
C	+2.51456672	−0.05790338	+1.79004815
C	+3.18556031	−0.52364677	+0.66058538
C	+2.72913830	−1.44363894	−0.29154307
C	+1.53017349	−2.14270459	−0.16858104
H	+0.55729023	−1.17884282	−0.28136342
C	+3.59194973	−1.59629667	−1.50979862
C	+4.06983440	−0.23518133	−1.99266136
C	+4.85262746	+0.51694837	−0.91248461
C	+4.61734168	−0.10261971	+0.46144663
C	−1.27400465	+0.14658128	−0.81273240
C	−2.56298246	+0.22960540	+0.00517161
C	−2.45418192	−0.58376945	+1.27761913
C	−1.32490273	+0.01814236	+2.11058344
C	−3.61867421	−0.43016497	+2.26056178
C	−3.00275942	−0.84423141	+3.60752966
C	−1.48259341	−0.63991420	+3.47439952
O	+6.23261274	+0.58032655	−1.19324476
H	+6.60510477	−0.29691234	−1.12014173
C	−2.21269605	−2.05499173	+0.96071827
C	−4.89182712	−1.17061131	+1.91719894
H	+4.53705882	+1.55659248	−0.89573309
H	+4.94614920	+0.58640522	+1.23379028
H	+5.26421120	−0.98455881	+0.52811469
H	+3.19726365	+0.34784171	−2.28355981
H	+4.70048342	−0.31964944	−2.87565887
H	+4.45259699	−2.22932616	−1.27005597

H	+3.04590334	−2.11761826	−2.29316457
H	+1.18303669	−2.35137685	+0.84017979
H	+1.32438174	−2.91893521	−0.89495237
H	+3.17756260	+0.34554657	+2.54789947
H	+1.02028025	+0.17210439	+3.21988882
H	+0.87574489	+0.47891157	−0.43750004
H	−1.34097183	−0.69649717	−1.50213507
H	−1.19246483	+1.02850860	−1.44657251
H	−2.75836034	+1.26671818	+0.28441630
H	−3.40630834	−0.09637855	−0.60482148
H	−3.84648152	+0.63877923	+2.30105603
H	−3.23499515	−1.88826430	+3.81689126
H	−3.42584818	−0.26687320	+4.42639275
H	−0.95084754	−1.59051072	+3.49648609
H	−1.07487898	−0.03283050	+4.27879047
H	−2.20886173	−2.66566219	+1.86185613
H	−1.26198728	−2.21020380	+0.45657284
H	−2.99780547	−2.43605256	+0.30961862
H	−5.67398101	−0.95121857	+2.64140246
H	−4.73595083	−2.24814727	+1.92276712
H	−5.26670197	−0.88969771	+0.93394510
H	−1.59807429	+1.07336069	+2.23623095

51

* E = +2.445 kcal/mol ; (21) 056_320_347_309_034_018_190

C	+0.00000000	+0.00000000	+0.00000000
C	+0.00000000	+0.00000000	+1.40549618
C	+1.14996758	+0.00000000	+2.18337886
C	+2.49958655	−0.03501265	+1.82671320
C	+3.18246908	−0.43154807	+0.67611938
C	+2.76042710	−1.32868202	−0.30780333
C	+1.59173197	−2.08695839	−0.19967815
H	+0.57155377	−1.20941401	−0.32081769
C	+3.64166039	−1.42614901	−1.51497645
C	+5.07652219	−1.78631112	−1.13214282
C	+5.54378835	−1.06362125	+0.12697673
C	+4.59248849	+0.06487352	+0.49434037
C	−1.32326577	+0.22475314	−0.67853062
C	−2.43825346	−0.69017884	−0.16195898
C	−2.30508515	−0.99685451	+1.33386462
C	−1.36964250	+0.02004064	+2.00616420

C −3.57289147 −0.77268668 +2.16616212
C −3.04644368 −0.65858311 +3.60343417
C −1.57623208 −0.21388286 +3.49309564
O +5.59979081 −2.03269949 +1.15094436
H +5.73791606 −1.59998468 +1.99102827
C −1.78229531 −2.41530178 +1.51856745
C −4.67590814 −1.79209093 +1.99259191
H +6.54262386 −0.64908472 −0.03012470
H +4.62999613 +0.81434229 −0.30112882
H +4.93676690 +0.57190772 +1.39644596
H +5.73920182 −1.56437867 −1.96603097
H +5.15310220 −2.85191921 −0.93136297
H +3.24832268 −2.15596403 −2.21830247
H +3.65078469 −0.46377567 −2.03425107
H +1.28268036 −2.33635551 +0.81159170
H +1.43985942 −2.87366661 −0.92803809
H +3.15099685 +0.34897487 +2.60492324
H +0.98281365 +0.15878299 +3.24129041
H +0.85997258 +0.47529666 −0.46846279
H −1.22053254 +0.11643261 −1.75630007
H −1.61211005 +1.26624469 −0.50979416
H −3.40196405 −0.22380396 −0.36973285
H −2.42450083 −1.62341386 −0.72394010
H −3.96343403 +0.20375072 +1.86617894
H −3.11951983 −1.62399173 +4.10384565
H −3.64522011 +0.03578150 +4.18856100
H −0.90704824 −0.99548425 +3.85032389
H −1.36277360 +0.67539939 +4.08154222
H −1.66799169 −2.68408616 +2.56709326
H −0.81606652 −2.53297805 +1.03561547
H −2.46324085 −3.13202455 +1.06334892
H −5.55804723 −1.51083182 +2.56460661
H −4.36309172 −2.77580075 +2.33839689
H −4.97455858 −1.88170187 +0.94865339
H −1.80205538 +1.00147095 +1.76967455
51
* E = +2.520 kcal/mol ; (22) 318_058_344_309_034_018_292
C +0.00000000 +0.00000000 +0.00000000
C +0.00000000 +0.00000000 +1.40529850
C +1.15028460 +0.00000000 +2.18303766

C	+2.49955675	−0.03937350	+1.82728784
C	+3.18473538	−0.44918758	+0.68169233
C	+2.75904939	−1.34382542	−0.30440068
C	+1.58412841	−2.09513161	−0.20646286
H	+0.57031591	−1.21027785	−0.32069772
C	+3.62737686	−1.42464090	−1.52583239
C	+4.06573432	−0.03308033	−1.95652345
C	+4.82159227	+0.70193139	−0.84597121
C	+4.60389080	+0.02206822	+0.50201140
C	−1.32268778	+0.22801186	−0.67852966
C	−2.44076417	−0.68230117	−0.16049274
C	−2.30907500	−0.98647720	+1.33590545
C	−1.36900390	+0.02729706	+2.00650765
C	−3.57550652	−0.75520301	+2.16819294
C	−3.04796694	−0.64168226	+3.60526411
C	−1.57612492	−0.20267193	+3.49396843
O	+6.19935207	+0.82066052	−1.11948012
H	+6.59944129	−0.04658999	−1.08136020
C	−1.79265881	−2.40694950	+1.52299992
C	−4.68356031	−1.76938198	+1.99637197
H	+4.47240879	+1.72934742	−0.78987324
H	+4.91134224	+0.69000193	+1.30115593
H	+5.27674633	−0.84210633	+0.53507581
H	+3.17722575	+0.53392069	−2.23032189
H	+4.70180270	−0.06644895	−2.83907082
H	+4.50608642	−2.04105334	−1.30892914
H	+3.09734908	−1.93164663	−2.32940444
H	+1.27140580	−2.35651378	+0.80068761
H	+1.42642397	−2.87030711	−0.94629356
H	+3.15162892	+0.34758628	+2.60271286
H	+0.98370800	+0.16848374	+3.23949510
H	+0.86159483	+0.47306901	−0.46819652
H	−1.22076720	+0.11857621	−1.75625572
H	−1.60758416	+1.27063231	−0.51052428
H	−3.40267862	−0.21298184	−0.36953399
H	−2.43012435	−1.61660432	−0.72075876
H	−3.96142405	+0.22270836	+1.86714747
H	−3.12456364	−1.60628835	+4.10665768
H	−3.64376994	+0.05555319	+4.18989802
H	−0.90957488	−0.98566160	+3.85317616

H	−1.35951677	+0.68723588	+4.08021519
H	−1.67647510	−2.67348483	+2.57188921
H	−0.82858846	−2.53068297	+1.03704455
H	−2.47860664	−3.12140456	+1.07176906
H	−5.56424138	−1.48241810	+2.56771934
H	−4.37598111	−2.75400359	+2.34424982
H	−4.98268418	−1.85942988	+0.95261575
H	−1.79657886	+1.01036067	+1.76834517

51

* E = +2.851 kcal/mol ; (23) 056_317_350_357_328_062_057

C	+0.00000000	+0.00000000	+0.00000000
C	+0.00000000	+0.00000000	+1.41032551
C	+1.16757633	+0.00000000	+2.16263766
C	+2.51451453	−0.04692587	+1.79338524
C	+3.18415070	−0.49083065	+0.65576616
C	+2.73505135	−1.41727862	−0.29135988
C	+1.54591928	−2.13059267	−0.16095610
H	+0.55907799	−1.17459443	−0.28207679
C	+3.61958333	−1.59421753	−1.48704935
C	+5.03477473	−1.98489382	−1.06483904
C	+5.53547489	−1.17603106	+0.13799983
C	+4.60081586	−0.02408892	+0.44915524
C	−1.27460872	+0.14495908	−0.81241278
C	−2.56396281	+0.22203473	+0.00548326
C	−2.45218377	−0.59410500	+1.27587634
C	−1.32547305	+0.01013761	+2.11069958
C	−3.61772638	−0.44822408	+2.25868639
C	−3.00051394	−0.86454600	+3.60433198
C	−1.48121210	−0.65308363	+3.47238559
O	+5.59614374	−1.95462504	+1.31235317
H	+6.15707061	−2.71158406	+1.16114162
C	−2.20509598	−2.06353848	+0.95501437
C	−4.88778653	−1.19260245	+1.91226611
H	+6.52871075	−0.77372087	−0.07596743
H	+4.64268098	+0.68340273	−0.38280638
H	+4.96257450	+0.50329276	+1.32837483
H	+5.71049175	−1.86577190	−1.91040861
H	+5.04570242	−3.04011017	−0.79430306
H	+3.20945006	−2.34144592	−2.16219529
H	+3.67179566	−0.65739776	−2.04806148

H	+1.19903106	−2.32734069	+0.85024056
H	+1.35082576	−2.91981403	−0.87599353
H	+3.17830870	+0.34309797	+2.55699673
H	+1.01711747	+0.15730797	+3.22380752
H	+0.87309535	+0.48462711	−0.43645701
H	−1.33930268	−0.69681230	−1.50372049
H	−1.19617241	+1.02871910	−1.44426638
H	−2.76258834	+1.25783989	+0.28765673
H	−3.40654624	−0.10508153	−0.60513044
H	−3.84981646	+0.61968940	+2.30279020
H	−3.22832069	−1.91044921	+3.80953390
H	−3.42661368	−0.29225429	+4.42528292
H	−0.94493260	−1.60112250	+3.49190598
H	−1.07644812	−0.04678141	+4.27883620
H	−2.20242140	−2.67685091	+1.85434487
H	−1.25210119	−2.21410990	+0.45399548
H	−2.98715397	−2.44482994	+0.30022742
H	−5.67107209	−0.97963765	+2.63725404
H	−4.72705224	−2.26946562	+1.91335593
H	−5.26365893	−0.90941330	+0.92999565
H	−1.60410897	+1.06360132	+2.24002176

51

* E = +3.072 kcal/mol ; (24) 056_317_350_309_034_018_057

C	+0.00000000	+0.00000000	+0.00000000
C	+0.00000000	+0.00000000	+1.40561000
C	+1.15020176	+0.00000000	+2.18372279
C	+2.49946793	−0.02938118	+1.82741026
C	+3.18134047	−0.41949747	+0.67400228
C	+2.76162332	−1.32179416	−0.30537242
C	+1.59508236	−2.08424948	−0.19855479
H	+0.56969961	−1.20680824	−0.32150019
C	+3.65317659	−1.43303412	−1.50359827
C	+5.07665816	−1.80068486	−1.08968362
C	+5.55499738	−1.01208296	+0.13576723
C	+4.58345928	+0.09758657	+0.48572047
C	−1.32341089	+0.22617876	−0.67822338
C	−2.43885393	−0.68872596	−0.16251084
C	−2.30477180	−0.99788127	+1.33267682
C	−1.36971539	+0.01853942	+2.00624631
C	−3.57245726	−0.77638079	+2.16584228

C	−3.04558506	−0.66421704	+3.60305659
C	−1.57597172	−0.21759870	+3.49293066
O	+5.64758779	−1.82357946	+1.28556895
H	+6.23302508	−2.55614897	+1.10923835
C	−1.78055673	−2.41619798	+1.51440721
C	−4.67473696	−1.79638046	+1.99088972
H	+6.53290578	−0.56945127	−0.06901624
H	+4.60240247	+0.83503117	−0.32066534
H	+4.92872286	+0.60518753	+1.38299388
H	+5.75076870	−1.64330893	−1.93026894
H	+5.11168836	−2.86204636	−0.84675039
H	+3.26499428	−2.16766745	−2.20506395
H	+3.68219065	−0.47685905	−2.03281172
H	+1.28311969	−2.33094591	+0.81252849
H	+1.44669373	−2.87342368	−0.92520840
H	+3.15406374	+0.34439297	+2.60663372
H	+0.98274667	+0.15483626	+3.24214974
H	+0.85974306	+0.47723663	−0.46736932
H	−1.22099288	+0.11873785	−1.75617929
H	−1.61201808	+1.26756619	−0.50855516
H	−3.40241691	−0.22123345	−0.36868599
H	−2.42639701	−1.62114444	−0.72598348
H	−3.96394590	+0.20022805	+1.86764214
H	−3.11738427	−1.63064381	+4.10174878
H	−3.64504433	+0.02832935	+4.18968011
H	−0.90565627	−0.99861477	+3.84918517
H	−1.36335517	+0.67115970	+4.08239546
H	−1.66426612	−2.68647114	+2.56234069
H	−0.81490173	−2.53186696	+1.02982095
H	−2.46159989	−3.13279558	+1.05900311
H	−5.55701633	−1.51674286	+2.56352444
H	−4.36106561	−2.78037586	+2.33515749
H	−4.97349522	−1.88464465	+0.94683927
H	−1.80284784	+1.00005932	+1.77118057

S3.4. Cartesian Coordinates for the β-cyclodextrin dimer

O	−6.98400	−4.37500	−4.32500
H	−6.64800	−3.78900	−5.02500
C	−6.21100	−5.55200	−4.28800
H	−6.10600	−6.01800	−5.28300
H	−6.74800	−6.25800	−3.64900
C	−4.81200	−5.35200	−3.67800
H	−4.91900	−4.84000	−2.71600
O	−4.31200	−6.69900	−3.45900
C	−3.05100	−6.78700	−2.81900
O	−3.08500	−6.19600	−1.54400
H	−2.83800	−7.85900	−2.74200
C	−1.98200	−6.10100	−3.68000
O	−0.75100	−6.20300	−2.98800
H	−0.06400	−5.61800	−3.37900
H	−1.92500	−6.64800	−4.63200
C	−2.38100	−4.66100	−3.99100
O	−1.44600	−4.15000	−4.93000
H	−1.78800	−3.29100	−5.28300
H	−2.35900	−4.07400	−3.06000
C	−3.80400	−4.59900	−4.56500
H	−3.79500	−5.05500	−5.56600
O	−4.13900	−3.20600	−4.67200
C	−4.52800	−2.66900	−5.91500
O	−5.88700	−2.27400	−5.90500
C	−6.23800	−1.23800	−4.94700
C	−7.76200	−1.08000	−5.08900
O	−8.34600	−0.31700	−4.05800
H	−8.13100	+0.62200	−4.19600
H	−7.99200	−0.68000	−6.09100
H	−8.20100	−2.08000	−5.02500
H	−6.01000	−1.58700	−3.93500
H	−4.45200	−3.43000	−6.69900
C	−3.64400	−1.46600	−6.27200
O	−2.29900	−1.90600	−6.24900
H	−1.66600	−1.15500	−6.28200
H	−3.91900	−1.15100	−7.28900
C	−3.90900	−0.29200	−5.33200
O	−3.18600	+0.82600	−5.83200

H	−3.47700	+1.63300	−5.34200
H	−3.56800	−0.55800	−4.32100
C	−5.41200	+0.02200	−5.26800
H	−5.72700	+0.42400	−6.24200
O	−5.57800	+1.03000	−4.25400
C	−6.18700	+2.26100	−4.57100
O	−7.44400	+2.39600	−3.93300
C	−7.42700	+2.39200	−2.48000
C	−8.91100	+2.47100	−2.07800
O	−9.13800	+2.20700	−0.71200
H	−8.84200	+2.97000	−0.18300
H	−9.31800	+3.44700	−2.39000
H	−9.44300	+1.69700	−2.63700
H	−7.01600	+1.44300	−2.12100
H	−6.40000	+2.31500	−5.64400
C	−5.27100	+3.42500	−4.16400
O	−4.02600	+3.22600	−4.80700
H	−3.32500	+3.81500	−4.45200
H	−5.74000	+4.35100	−4.53100
C	−5.15700	+3.52600	−2.64500
O	−4.44000	+4.71600	−2.34100
H	−4.49900	+4.87800	−1.36700
H	−4.62200	+2.64300	−2.27000
C	−6.54800	+3.55900	−1.99100
H	−7.03300	+4.51300	−2.24300
O	−6.32900	+3.48500	−0.57400
C	−6.81800	+4.48800	+0.28900
O	−7.86700	+4.00100	+1.10400
C	−7.53300	+2.89400	+1.98700
C	−8.85200	+2.55700	+2.70600
O	−8.83600	+1.30900	+3.36000
H	−8.25900	+1.35300	+4.14300
H	−9.11400	+3.38500	+3.38600
H	−9.63400	+2.49700	+1.94400
H	−7.23000	+2.02900	+1.38900
H	−7.25500	+5.30900	−0.29000
C	−5.67700	+5.01600	+1.17500
O	−4.64600	+5.48100	+0.31800
H	−3.79400	+5.55600	+0.80000
H	−6.08000	+5.85200	+1.76500

C	−5.20500	+3.92700	+2.13200
O	−4.25400	+4.49500	+3.02600
H	−4.06000	+3.82900	+3.73100
H	−4.74700	+3.13000	+1.53900
C	−6.37500	+3.31200	+2.91300
H	−6.73500	+4.04500	+3.64900
O	−5.83300	+2.16700	+3.59200
C	−5.94400	+2.04100	+4.98900
O	−6.81500	+0.98200	+5.34100
C	−6.41200	−0.34400	+4.89700
C	−7.57600	−1.26200	+5.31200
O	−7.49700	−2.55300	+4.75200
H	−6.80600	−3.06000	+5.21300
H	−7.64100	−1.28300	+6.41300
H	−8.49800	−0.81200	+4.93500
H	−6.32700	−0.35400	+3.80500
H	−6.39400	+2.94300	+5.41800
C	−4.55800	+1.80300	+5.61000
O	−3.71400	+2.86100	+5.19200
H	−2.77500	+2.67100	+5.41400
H	−4.68500	+1.83000	+6.70200
C	−4.01500	+0.42400	+5.24200
O	−2.83900	+0.21100	+6.01000
H	−2.56900	−0.73600	+5.92200
H	−3.78600	+0.40000	+4.16600
C	−5.05200	−0.66900	+5.54000
H	−5.18000	−0.73900	+6.63100
O	−4.50100	−1.89900	+5.04100
C	−4.33800	−2.99800	+5.91100
O	−5.21200	−4.05900	+5.56900
C	−5.02300	−4.63700	+4.24900
C	−6.14600	−5.68000	+4.12200
O	−6.30300	−6.18300	+2.81400
H	−5.56700	−6.79000	+2.61600
H	−5.97800	−6.48100	+4.86200
H	−7.08700	−5.18500	+4.38100
H	−5.17000	−3.86300	+3.48900
H	−4.61300	−2.71500	+6.93300
C	−2.88500	−3.49500	+5.88100
O	−2.05000	−2.39800	+6.19800

H	−1.10400	−2.60200	+6.02500
H	−2.79500	−4.27300	+6.65400
C	−2.55100	−4.12900	+4.53500
O	−1.26000	−4.71400	+4.64000
H	−1.09600	−5.26300	+3.83300
H	−2.56400	−3.34700	+3.76300
C	−3.59100	−5.19500	+4.16100
H	−3.48900	−6.04500	+4.85000
O	−3.27400	−5.61400	+2.82400
C	−2.99200	−6.96500	+2.53900
O	−4.02000	−7.55400	+1.76700
H	−2.95100	−7.54800	+3.46600
C	−1.65200	−7.07200	+1.79400
O	−0.66300	−6.46100	+2.60400
H	+0.16700	−6.30700	+2.10100
H	−1.43200	−8.14300	+1.67500
C	−1.75000	−6.45000	+0.40500
O	−0.54400	−6.74500	−0.29100
H	−0.65500	−6.45700	−1.23100
H	−1.88200	−5.36500	+0.50900
C	−2.95900	−7.00500	−0.36400
H	−2.74200	−8.04500	−0.64700
C	−4.25700	−6.95600	+0.46100
H	−4.57500	−5.91700	+0.58700
C	−5.42900	−7.76600	−0.12200
H	−5.08700	−8.79400	−0.32800
H	−6.20200	−7.82700	+0.64900
O	−6.03300	−7.16400	−1.24300
H	−5.43600	−7.22800	−2.01100
O	+5.41900	+0.37300	−4.79200
H	+5.17000	−0.55400	−4.95400
C	+4.72400	+1.21200	−5.68500
H	+4.82600	+0.88900	−6.73500
H	+5.18000	+2.20300	−5.59900
C	+3.22900	+1.36500	−5.34700
H	+3.13500	+1.60200	−4.28400
O	+2.78500	+2.50300	−6.13700
C	+1.43500	+2.88300	−5.94300
O	+1.18700	+3.20600	−4.59500
H	+1.27900	+3.75700	−6.58500

C	+0.50800	+1.73900	−6.37200
O	−0.82600	+2.17300	−6.16500
H	−1.46000	+1.42300	−6.18900
H	+0.68100	+1.55300	−7.44200
C	+0.85100	+0.46800	−5.60100
O	+0.08400	−0.59400	−6.15300
H	+0.42000	−1.44900	−5.78500
H	+0.60100	+0.62200	−4.54400
C	+2.35100	+0.14900	−5.69900
H	+2.57200	−0.16900	−6.72800
O	+2.59100	−0.94200	−4.79400
C	+3.18100	−2.13100	−5.26800
O	+4.47700	−2.31400	−4.72800
C	+4.55300	−2.46900	−3.28400
C	+6.06100	−2.55000	−2.98700
O	+6.37400	−2.45200	−1.61700
H	+6.13600	−3.28600	−1.17300
H	+6.46700	−3.46700	−3.44800
H	+6.53700	−1.69700	−3.47800
H	+4.14200	−1.57900	−2.79400
H	+3.32600	−2.07300	−6.35200
C	+2.29600	−3.34000	−4.92700
O	+1.00500	−3.07800	−5.44500
H	+0.33600	−3.70800	−5.09400
H	+2.73400	−4.21200	−5.43400
C	+2.29700	−3.62700	−3.42900
O	+1.61900	−4.86100	−3.23300
H	+1.75700	−5.15200	−2.29800
H	+1.78500	−2.81200	−2.90100
C	+3.73300	−3.71000	−2.89000
H	+4.21400	−4.60800	−3.30500
O	+3.62100	−3.83700	−1.46400
C	+4.20300	−4.93900	−0.80600
O	+5.29300	−4.53900	+0.00300
C	+4.98700	−3.61300	+1.08300
C	+6.35300	−3.34400	+1.74400
O	+6.34500	−2.27200	+2.65700
H	+5.86700	−2.53400	+3.46400
H	+6.71600	−4.28200	+2.19700
H	+7.05200	−3.07000	+0.95000

H	+4.60200	−2.67700	+0.66500
H	+4.62500	−5.64000	−1.53600
C	+3.15100	−5.65200	+0.05800
O	+2.07700	−6.02400	−0.78800
H	+1.28600	−6.28800	−0.26900
H	+3.62600	−6.55100	+0.47700
C	+2.71600	−4.75700	+1.21000
O	+1.85500	−5.51500	+2.05100
H	+1.70300	−5.01100	+2.88700
H	+2.19000	−3.88700	+0.79500
C	+3.93300	−4.25500	+2.00300
H	+4.38200	−5.11000	+2.52800
O	+3.43600	−3.30600	+2.96200
C	+3.66100	−3.51600	+4.34000
O	+4.56900	−2.57100	+4.87100
C	+4.16900	−1.17100	+4.83100
C	+5.34600	−0.44200	+5.51000
O	+5.35200	+0.95500	+5.34300
H	+4.58500	+1.35600	+5.79000
H	+5.37400	−0.73800	+6.57400
H	+6.26400	−0.80900	+5.04400
H	+4.09000	−0.83200	+3.79300
H	+4.13500	−4.49100	+4.49900
C	+2.33300	−3.45300	+5.10700
O	+1.46000	−4.41000	+4.53300
H	+0.53300	−4.27100	+4.82700
H	+2.54800	−3.72100	+6.15200
C	+1.76800	−2.04100	+5.08200
O	+0.63800	−1.99800	+5.94900
H	+0.36600	−1.05300	+6.05200
H	+1.47400	−1.79300	+4.05500
C	+2.81300	−1.01600	+5.54700
H	+2.95600	−1.13700	+6.63100
O	+2.22000	+0.26200	+5.27600
C	+2.09500	+1.23600	+6.27900
O	+2.95600	+2.33300	+6.02900
C	+2.74800	+2.94200	+4.72500
C	+3.90700	+3.92700	+4.52900
O	+4.00500	+4.37900	+3.18500
H	+3.30300	+5.03800	+3.03700

H	+3.82300	+4.77200	+5.22900
H	+4.83600	+3.39400	+4.74600
H	+2.83700	+2.17700	+3.95000
H	+2.39500	+0.83300	+7.25300
C	+0.64100	+1.72700	+6.33500
O	−0.18300	+0.59700	+6.56100
H	−1.12500	+0.80400	+6.37300
H	+0.56400	+2.42600	+7.18000
C	+0.27600	+2.49000	+5.06100
O	−0.99100	+3.10500	+5.27700
H	−1.16400	+3.72900	+4.53200
H	+0.22700	+1.78100	+4.22300
C	+1.33500	+3.55100	+4.72800
H	+1.28700	+4.34700	+5.48400
O	+0.98100	+4.09000	+3.44000
C	+0.75500	+5.47300	+3.31000
O	+1.77200	+6.09200	+2.53800
C	+1.85500	+5.72600	+1.13500
C	+3.03200	+6.56300	+0.60300
O	+3.49000	+6.15900	−0.66600
H	+2.85200	+6.44900	−1.34200
H	+2.75300	+7.62900	+0.63100
H	+3.86900	+6.42900	+1.29500
H	+2.10300	+4.66200	+1.04100
H	+0.81000	+5.96000	+4.29000
C	−0.62300	+5.73600	+2.67900
O	−1.59200	+5.06800	+3.46300
H	−2.47000	+5.06700	+3.02100
H	−0.79400	+6.82300	+2.71900
C	−0.64700	+5.31100	+1.21600
O	−1.90500	+5.69200	+0.67700
H	−1.86800	+5.59900	−0.30600
H	−0.51000	+4.22100	+1.15700
C	+0.50100	+5.98800	+0.45600
H	+0.31000	+7.07100	+0.42500
O	+0.48800	+5.45500	−0.87500
C	+0.35700	+6.32200	−1.97900
O	+1.55300	+6.37700	−2.73500
H	+0.17900	+7.34900	−1.64000
C	−0.80300	+5.85300	−2.86600

O	−1.96300	+5.82300	−2.05600
H	−2.71300	+5.37000	−2.50200
H	−0.92300	+6.59400	−3.67000
C	−0.47400	+4.50700	−3.50300
O	−1.50700	+4.20500	−4.43200
H	−1.23100	+3.42000	−4.96500
H	−0.42200	+3.74200	−2.71500
C	+0.88700	+4.55500	−4.21300
H	+0.79000	+5.18600	−5.10800
C	+1.99700	+5.11600	−3.30800
H	+2.21300	+4.40600	−2.50300
C	+3.31300	+5.46800	−4.02900
H	+3.08600	+6.12200	−4.88700
H	+3.92500	+6.04400	−3.33000
O	+4.07700	+4.34400	−4.39600
H	+3.65300	+3.88800	−5.14500

S3.5. Cartesian Coordinates of encapsulated PreD Conformers

```
345
 * E = 0.000  kcal/mol
 C   +0.51026   −0.92303   −0.33129
 C   +0.80229   −0.40724   +0.86779
 C   −0.19448   −0.03195   +1.86903
 C   −1.49200   +0.29746   +1.78058
 C   −2.41312   +0.46995   +0.65344
 C   −2.17483   +1.20386   −0.44268
 C   −0.91222   +1.95298   −0.68708
 H   −1.13869   +2.94820   −1.07234
 C   −3.20271   +1.37365   −1.51962
 C   −4.37036   +0.41566   −1.42274
 C   −4.86217   +0.35760   +0.00698
 C   −3.75980   −0.16223   +0.89958
 C   +1.52434   −1.36629   −1.33673
 C   +2.95301   −1.40756   −0.80252
 C   +3.20236   −0.21504   +0.09799
 C   +2.24501   −0.32159   +1.28183
 C   +4.53995   −0.18220   +0.84084
 C   +4.25429   +0.76808   +2.02019
 C   +2.72545   +0.77924   +2.21869
 O   −5.19984   +1.64884   +0.47423
 H   −6.02995   +1.93301   +0.09113
 C   +3.00248   +1.08120   −0.68256
 C   +5.75419   +0.20073   +0.02534
 H   −5.72448   −0.30338   +0.09174
 H   −3.68415   −1.24538   +0.78391
 H   −4.04959   +0.01323   +1.93728
 H   −4.06517   −0.58658   −1.72593
 H   −5.16952   +0.72413   −2.09495
 H   −3.56507   +2.40450   −1.47659
 H   −2.71394   +1.27234   −2.49147
 H   −0.30811   +1.44968   −1.44529
 H   −0.30315   +2.04909   +0.20600
 H   −1.98552   +0.42475   +2.73947
 H   +0.19477   −0.04809   +2.88100
 H   −0.52856   −1.02159   −0.61663
 H   +1.47175   −0.69502   −2.20094
```

H	+1.23892	−2.34510	−1.72694
H	+3.10107	−2.31978	−0.22016
H	+3.65954	−1.43950	−1.63378
H	+4.69776	−1.18863	+1.23861
H	+4.62151	+1.76862	+1.79251
H	+4.77679	+0.44617	+2.91757
H	+2.29428	+1.73888	+1.93193
H	+2.44269	+0.60316	+3.25487
H	+3.32429	+1.95052	−0.11223
H	+1.95811	+1.23163	−0.94589
H	+3.58587	+1.06042	−1.60301
H	+6.65542	+0.15149	+0.63139
H	+5.67160	+1.21773	−0.35551
H	+5.89231	−0.46699	−0.82435
H	+2.48741	−1.27041	+1.77724
O	−6.26258	−5.07972	−1.65165
H	−5.52127	−5.43124	−2.20351
C	−6.19042	−5.68249	−0.35990
H	−6.14741	−6.80173	−0.44739
H	−7.11888	−5.42115	+0.19191
C	−4.98021	−5.18135	+0.43361
H	−4.93763	−4.06231	+0.42859
O	−5.24342	−5.63449	+1.82361
C	−4.22347	−5.18621	+2.77038
O	−4.17363	−3.72924	+2.73392
H	−4.55050	−5.54521	+3.78300
C	−2.87962	−5.77979	+2.36238
O	−1.88219	−5.32728	+3.30163
H	−0.98242	−5.49115	+2.90981
H	−2.95339	−6.90444	+2.41360
C	−2.50668	−5.37044	+0.93873
O	−1.29017	−6.07241	+0.58854
H	−1.17918	−6.02330	−0.40702
H	−2.33287	−4.25955	+0.89067
C	−3.63343	−5.75262	−0.03016
H	−3.69590	−6.86911	−0.12542
O	−3.29562	−5.16806	−1.34726
C	−3.13274	−6.12806	−2.43276
O	−4.16280	−5.86446	−3.43595
C	−4.10544	−4.47870	−3.96817

C	−5.29619	−4.39467	−4.92772
O	−5.59320	−3.04528	−5.28554
H	−4.86289	−2.70384	−5.85816
H	−5.08749	−5.01050	−5.84413
H	−6.19330	−4.82150	−4.42989
H	−4.23393	−3.76670	−3.11368
H	−3.30621	−7.17485	−2.06497
C	−1.76383	−5.97143	−3.08546
O	−0.75476	−6.24282	−2.09025
H	+0.12718	−5.94765	−2.44410
H	−1.67394	−6.72597	−3.91895
C	−1.59072	−4.56742	−3.66213
O	−0.32383	−4.53743	−4.36204
H	−0.31424	−3.72532	−4.95132
H	−1.58369	−3.80926	−2.83017
C	−2.73931	−4.25015	−4.62944
H	−2.64437	−4.87539	−5.55659
O	−2.62514	−2.81949	−4.99026
C	−2.43463	−2.54641	−6.40977
O	−3.58538	−1.78312	−6.88861
C	−3.78138	−0.51057	−6.14722
C	−5.06194	+0.07865	−6.74582
O	−5.57714	+1.14396	−5.94782
H	−4.96407	+1.91630	−6.02250
H	−4.86210	+0.43028	−7.79402
H	−5.83684	−0.71616	−6.79689
H	−3.92442	−0.75165	−5.06322
H	−2.41854	−3.50253	−6.99855
C	−1.17252	−1.71952	−6.62683
O	−0.04573	−2.48691	−6.15283
H	+0.73770	−1.88048	−6.06323
H	−1.05137	−1.52758	−7.73163
C	−1.26426	−0.38181	−5.89476
O	−0.08994	+0.38764	−6.24555
H	−0.25677	+1.34403	−5.99112
H	−1.28887	−0.55371	−4.78249
C	−2.53416	+0.36675	−6.32183
H	−2.44055	+0.70404	−7.38812
O	−2.65954	+1.55151	−5.44346
C	−2.64416	+2.84000	−6.12742

O	−3.93681	+3.48784	−5.91419
C	−4.25076	+3.68168	−4.47502
C	−5.66101	+4.27851	−4.47184
O	−6.25290	+4.22139	−3.17374
H	−5.78764	+4.87101	−2.58978
H	−5.62602	+5.33757	−4.84433
H	−6.30292	+3.69544	−5.16674
H	−4.24460	+2.67975	−3.97440
H	−2.53802	+2.69745	−7.23629
C	−1.54637	+3.73293	−5.55944
O	−0.27869	+3.07684	−5.77398
H	+0.40867	+3.52024	−5.20746
H	−1.55490	+4.71335	−6.11702
C	−1.77187	+4.00211	−4.07264
O	−0.76779	+4.95126	−3.64197
H	−1.06003	+5.33369	−2.76085
H	−1.66525	+3.04442	−3.48914
C	−3.17584	+4.57910	−3.84666
H	−3.23798	+5.61736	−4.26807
O	−3.39392	+4.63035	−2.38424
C	−3.66584	+5.95617	−1.83688
O	−5.01563	+5.95160	−1.27857
C	−5.18868	+4.92416	−0.21804
C	−6.67254	+4.99828	+0.14960
O	−7.08623	+3.83989	+0.87849
H	−6.66798	+3.87930	+1.77508
H	−6.87532	+5.92950	+0.74301
H	−7.27787	+5.05094	−0.78108
H	−4.94001	+3.92176	−0.65196
H	−3.66037	+6.72719	−2.65369
C	−2.66969	+6.29066	−0.73156
O	−1.34492	+6.26101	−1.30624
H	−0.67845	+6.23394	−0.56789
H	−2.88623	+7.33175	−0.35532
C	−2.77857	+5.30605	+0.43181
O	−1.90833	+5.77799	+1.48902
H	−2.16280	+5.29809	+2.33338
H	−2.45198	+4.28207	+0.09914
C	−4.22659	+5.23619	+0.93532
H	−4.51105	+6.20125	+1.43405

O	−4.29044	+4.13619	+1.91599
C	−4.71511	+4.50733	+3.25672
O	−5.98131	+3.83504	+3.54238
C	−5.89014	+2.34986	+3.45211
C	−7.31645	+1.88082	+3.75466
O	−7.51620	+0.51424	+3.39026
H	−6.98276	−0.05094	+4.00155
H	−7.54567	+2.03481	+4.84450
H	−8.03359	+2.49732	+3.17010
H	−5.58432	+2.06862	+2.40630
H	−4.91974	+5.61083	+3.31803
C	−3.67414	+4.06157	+4.27838
O	−2.43076	+4.73313	+3.97568
H	−1.69682	+4.26262	+4.45312
H	−4.01863	+4.36753	+5.30798
C	−3.49098	+2.54635	+4.23712
O	−2.57107	+2.18350	+5.29709
H	−2.63924	+1.19269	+5.43591
H	−3.06382	+2.24150	+3.24274
C	−4.83851	+1.84478	+4.44755
H	−5.18812	+1.98827	+5.50499
O	−4.61933	+0.39958	+4.19924
C	−4.89988	−0.47962	+5.32579
O	−6.02082	−1.34911	+4.96702
H	−5.23149	+0.11392	+6.21962
C	−3.68560	−1.34806	+5.63479
O	−2.58886	−0.47697	+5.98279
H	−1.74613	−1.00469	+5.95436
H	−3.92816	−2.00925	+6.51603
C	−3.31948	−2.22555	+4.43854
O	−2.24550	−3.10264	+4.85450
H	−2.17659	−3.84733	+4.18561
H	−2.97561	−1.58163	+3.58087
C	−4.53550	−3.05265	+4.00032
H	−4.77385	−3.82601	+4.77794
C	−5.75543	−2.15625	+3.74892
H	−5.54084	−1.45651	+2.90093
C	−7.06279	−2.91580	+3.50590
H	−7.19249	−3.70808	+4.29179
H	−7.91504	−2.20826	+3.59381

O	−7.10309	−3.49194	+2.20014
H	−6.45076	−4.23446	+2.16544
O	+6.84111	−4.72305	+0.36190
H	+6.12154	−5.22178	+0.82192
C	+6.79823	−5.03799	−1.02912
H	+6.85361	−6.14977	−1.18600
H	+7.69155	−4.58386	−1.50928
C	+5.53563	−4.48628	−1.69808
H	+5.38278	−3.40974	−1.43049
O	+5.82489	−4.57033	−3.15300
C	+4.74786	−4.01951	−3.97198
O	+4.52410	−2.63653	−3.56378
H	+5.09940	−4.06529	−5.03757
C	+3.48891	−4.85023	−3.74810
O	+2.43513	−4.29529	−4.56592
H	+1.56439	−4.65221	−4.24506
H	+3.69182	−5.91029	−4.07410
C	+3.08550	−4.84332	−2.27521
O	+1.97704	−5.76245	−2.11767
H	+1.85025	−5.92242	−1.13490
H	+2.76236	−3.80844	−1.97699
C	+4.25948	−5.27988	−1.38764
H	+4.44332	−6.38063	−1.50603
O	+3.86952	−4.99490	+0.01157
C	+3.81069	−6.15398	+0.89362
O	+4.81560	−5.98305	+1.94116
C	+4.61887	−4.73882	+2.72996
C	+5.80973	−4.71071	+3.69239
O	+5.95757	−3.43392	+4.31251
H	+5.20618	−3.30292	+4.94197
H	+5.68867	−5.51564	+4.46701
H	+6.74004	−4.92481	+3.12365
H	+4.65439	−3.86601	+2.02906
H	+4.08655	−7.08946	+0.33662
C	+2.43499	−6.25928	+1.54150
O	+1.45438	−6.41910	+0.49412
H	+0.55309	−6.24825	+0.87961
H	+2.41826	−7.17085	+2.20584
C	+2.12873	−5.02285	+2.38550
O	+0.87003	−5.25911	+3.05953

H	+0.79418	−4.60372	+3.81562
H	+2.04343	−4.11575	+1.72417
C	+3.24617	−4.80154	+3.41352
H	+3.23103	−5.62332	+4.17787
O	+2.98007	−3.50826	+4.08251
C	+2.79624	−3.58455	+5.52779
O	+3.86671	−2.82395	+6.16863
C	+3.88747	−1.39363	+5.76685
C	+5.11313	−0.82374	+6.48694
O	+5.45732	+0.47325	+6.00172
H	+4.76299	+1.11233	+6.29656
H	+4.91979	−0.79381	+7.59322
H	+5.98052	−1.49613	+6.31255
H	+4.01685	−1.33777	+4.65569
H	+2.90518	−4.64457	+5.88289
C	+1.45358	−2.98675	+5.93386
O	+0.40880	−3.73870	+5.28231
H	−0.43859	−3.22051	+5.34259
H	+1.34028	−3.08854	+7.05180
C	+1.36885	−1.50799	+5.56066
O	+0.12379	−0.99713	+6.09298
H	+0.16846	+0.00545	+6.08384
H	+1.38126	−1.38915	+4.44052
C	+2.55478	−0.74604	+6.16642
H	+2.45845	−0.70998	+7.28395
O	+2.51133	+0.63117	+5.62755
C	+2.34674	+1.68679	+6.62180
O	+3.54604	+2.52088	+6.60675
C	+3.81779	+3.11302	+5.27115
C	+5.12779	+3.88333	+5.46309
O	+5.72375	+4.23642	+4.21552
H	+5.15234	+4.91081	+3.77212
H	+4.93930	+4.80007	+6.08569
H	+5.84778	+3.24185	+6.01547
H	+3.95167	+2.27959	+4.53528
H	+2.27463	+1.24925	+7.65361
C	+1.14085	+2.55621	+6.28423
O	−0.03809	+1.72402	+6.31359
H	−0.78409	+2.21718	+5.87660
H	+1.04615	+3.36041	+7.06926

C	+1.30432	+3.21036	+4.91392
O	+0.18814	+4.11266	+4.72521
H	+0.40207	+4.70871	+3.94712
H	+1.29586	+2.42216	+4.11029
C	+2.62768	+3.98559	+4.84966
H	+2.56721	+4.90371	+5.49241
O	+2.82616	+4.38659	+3.43953
C	+2.90395	+5.82153	+3.20062
O	+4.23188	+6.12382	+2.66875
C	+4.53571	+5.36499	+1.42807
C	+5.97861	+5.75285	+1.09192
O	+6.55119	+4.87218	+0.12568
H	+6.10626	+5.02818	−0.74364
H	+6.00990	+6.81529	+0.72709
H	+6.59387	+5.69352	+2.01533
H	+4.46230	+4.27047	+1.65526
H	+2.80728	+6.39117	+4.16356
C	+1.85674	+6.24308	+2.17626
O	+0.55364	+5.94502	+2.72003
H	−0.11898	+5.99770	+1.98811
H	+1.94227	+7.35486	+2.00608
C	+2.07274	+5.51623	+0.84969
O	+1.12599	+6.05641	−0.10262
H	+1.43115	+5.80154	−1.02369
H	+1.89271	+4.41283	+0.98228
C	+3.50722	+5.74204	+0.35312
H	+3.64132	+6.81306	+0.04529
O	+3.70508	+4.86736	−0.82432
C	+4.05461	+5.55772	−2.06003
O	+5.38508	+5.11609	−2.47269
C	+5.46795	+3.64812	−2.68602
C	+6.94125	+3.40368	−3.02539
O	+7.27334	+2.01686	−2.97346
H	+6.83153	+1.56136	−3.73157
H	+7.16990	+3.82762	−4.04049
H	+7.57636	+3.93584	−2.28498
H	+5.18938	+3.13300	−1.73076
H	+4.12670	+6.66516	−1.88750
C	+3.05846	+5.21711	−3.16272
O	+1.75007	+5.65283	−2.73690

H	+1.06781	+5.22856	−3.32328
H	+3.34934	+5.77833	−4.09708
C	+3.05994	+3.71889	−3.46144
O	+2.18350	+3.50179	−4.59324
H	+2.37430	+2.58890	−4.96291
H	+2.67855	+3.14783	−2.56968
C	+4.48353	+3.25240	−3.79505
H	+4.80899	+3.68776	−4.77703
O	+4.45197	+1.77599	−3.90261
C	+4.83530	+1.24058	−5.20461
O	+6.05480	+0.45291	−5.03994
H	+5.08959	+2.07357	−5.91372
C	+3.73727	+0.33956	−5.75867
O	+2.54105	+1.13179	−5.90987
H	+1.76831	+0.52011	−6.04582
H	+4.06024	−0.04000	−6.77077
C	+3.48672	−0.85288	−4.83737
O	+2.52076	−1.71284	−5.48802
H	+2.54976	−2.60995	−5.03910
H	+3.07603	−0.49782	−3.85024
C	+4.79696	−1.61478	−4.59853
H	+5.12487	−2.12273	−5.54406
C	+5.89688	−0.67507	−4.08592
H	+5.60158	−0.25894	−3.08821
C	+7.29206	−1.30400	−4.02095
H	+7.51605	−1.82618	−4.99026
H	+8.04570	−0.49823	−3.88813
O	+7.41601	−2.20673	−2.92309
H	+6.87037	−3.00916	−3.11279

345

* E = 1.328 kcal/mol

C	−0.48930	−0.82441	+0.63539
C	−0.64997	−0.56679	−0.66820
C	+0.44715	−0.42515	−1.62609
C	+1.76339	−0.24276	−1.45560
C	+2.59049	−0.11575	−0.24769
C	+2.46014	+0.83773	+0.68388
C	+1.37687	+1.86066	+0.66579
H	+1.79574	+2.86061	+0.78800
C	+3.40087	+0.93247	+1.84973

C	+4.69196	+0.16410	+1.65157
C	+4.38958	−1.22044	+1.13334
C	+3.70548	−1.12605	−0.20770
C	−1.60317	−0.99237	+1.61690
C	−2.98711	−1.07863	+0.98354
C	−3.08450	−0.10636	−0.17384
C	−2.04979	−0.52136	−1.21684
C	−4.35948	−0.17799	−1.01590
C	−3.92936	+0.46319	−2.34867
C	−2.39358	+0.35032	−2.41793
O	+5.54433	−1.99512	+0.93275
H	+5.93621	−2.24196	+1.77116
C	−2.85358	+1.31944	+0.31811
C	−5.60090	+0.43842	−0.41203
H	+3.71522	−1.72406	+1.83791
H	+3.32414	−2.10884	−0.48804
H	+4.46299	−0.87504	−0.95574
H	+5.24857	+0.10991	+2.58589
H	+5.32565	+0.66960	+0.92338
H	+3.61801	+1.98369	+2.04465
H	+2.88330	+0.57648	+2.74762
H	+0.68789	+1.70252	+1.49950
H	+0.79616	+1.83177	−0.24978
H	+2.35384	−0.26445	−2.36796
H	+0.12973	−0.50227	−2.65900
H	+0.51179	−0.90123	+1.03430
H	−1.56879	−0.15149	+2.31822
H	−1.41033	−1.87388	+2.23106
H	−3.15669	−2.08881	+0.60474
H	−3.75351	−0.88694	+1.73627
H	−4.55414	−1.23954	−1.19119
H	−4.24130	+1.50750	−2.37987
H	−4.41076	−0.02615	−3.19190
H	−1.91996	+1.32850	−2.32909
H	−2.05635	−0.08102	−3.35872
H	−3.06373	+2.05415	−0.45720
H	−1.82539	+1.46956	+0.64184
H	−3.51088	+1.53559	+1.16004
H	−6.45967	+0.29036	−1.06193
H	−5.48040	+1.51170	−0.26958

H	−5.83869	−0.00441	+0.55447
H	−2.31002	−1.54827	−1.50201
O	−5.74943	−4.32687	−3.81205
H	−4.97488	−4.30029	−4.42627
C	−5.60013	−5.45017	−2.94442
H	−5.43215	−6.38945	−3.53789
H	−6.54775	−5.57262	−2.37716
C	−4.44800	−5.25289	−1.95506
H	−4.52786	−4.25845	−1.44656
O	−4.65220	−6.31742	−0.93822
C	−3.68559	−6.24741	+0.15609
O	−3.80107	−4.93617	+0.78289
H	−3.96546	−7.05821	+0.88119
C	−2.28437	−6.44628	−0.41035
O	−1.34106	−6.36738	+0.67947
H	−0.42854	−6.25852	+0.29702
H	−2.22689	−7.47239	−0.87556
C	−1.96719	−5.39848	−1.47637
O	−0.68050	−5.73594	−2.04643
H	−0.58525	−5.24012	−2.91341
H	−1.92113	−4.37518	−1.00937
C	−3.04878	−5.41388	−2.56477
H	−2.98846	−6.36905	−3.15102
O	−2.78268	−4.27037	−3.46655
C	−2.54021	−4.62326	−4.86108
O	−3.59695	−4.02847	−5.67645
C	−3.65925	−2.54858	−5.56086
C	−4.86459	−2.15990	−6.42208
O	−5.25511	−0.80443	−6.20670
H	−4.56329	−0.21380	−6.59394
H	−4.62560	−2.33530	−7.50570
H	−5.72477	−2.81078	−6.15502
H	−3.83498	−2.28275	−4.48717
H	−2.61674	−5.73450	−5.00520
C	−1.19329	−4.08261	−5.32832
O	−0.16249	−4.67031	−4.50810
H	+0.68079	−4.15934	−4.64274
H	−1.03508	−4.39423	−6.40111
C	−1.14930	−2.55789	−5.24435
O	+0.10797	−2.12815	−5.81813

H	+0.04386	−1.14553	−6.01078
H	−1.21009	−2.22695	−4.16939
C	−2.32280	−1.95643	−6.02886
H	−2.18147	−2.13360	−7.12800
O	−2.32560	−0.50016	−5.76525
C	−2.14875	+0.34779	−6.93977
O	−3.36288	+1.13978	−7.12153
C	−3.69167	+1.96715	−5.93199
C	−5.01046	+2.65183	−6.30359
O	−5.65239	+3.22009	−5.16277
H	−5.10827	+3.98015	−4.84009
H	−4.82129	+3.43800	−7.08442
H	−5.69928	+1.89828	−6.74242
H	−3.83224	+1.28497	−5.05515
H	−2.03383	−0.27814	−7.86507
C	−0.97116	+1.29574	−6.74182
O	+0.22190	+0.50064	−6.57248
H	+0.94074	+1.08063	−6.20337
H	−0.86303	+1.93410	−7.66517
C	−1.19340	+2.19904	−5.53062
O	−0.10484	+3.15316	−5.49066
H	−0.35676	+3.87977	−4.84612
H	−1.19097	+1.58248	−4.58933
C	−2.53495	+2.93556	−5.65045
H	−2.47377	+3.71772	−6.45302
O	−2.78714	+3.58940	−4.34689
C	−2.89883	+5.04163	−4.38530
O	−4.25056	+5.40767	−3.96573
C	−4.58714	+4.90363	−2.60901
C	−6.04910	+5.31320	−2.40797
O	−6.63981	+4.63764	−1.29834
H	−6.23638	+4.98885	−0.46663
H	−6.11476	+6.42693	−2.27414
H	−6.62784	+5.04654	−3.31830
H	−4.48374	+3.78769	−2.60949
H	−2.77630	+5.42161	−5.43499
C	−1.89722	+5.67503	−3.42643
O	−0.56996	+5.30326	−3.85429
H	+0.06905	+5.50458	−3.11854
H	−2.00340	+6.79675	−3.48103

C	−2.15333	+5.21944	−1.99053
O	−1.24606	+5.95143	−1.13257
H	−1.58701	+5.88747	−0.19116
H	−1.95887	+4.11423	−1.89657
C	−3.60705	+5.51527	−1.59844
H	−3.76598	+6.62390	−1.52465
O	−3.83670	+4.90003	−0.27211
C	−4.24193	+5.82435	+0.78096
O	−5.58163	+5.45175	+1.22932
C	−5.65483	+4.05591	+1.73240
C	−7.13638	+3.85627	+2.06561
O	−7.45233	+2.47983	+2.26989
H	−7.02808	+2.18278	+3.11214
H	−7.40428	+4.46286	+2.97279
H	−7.75131	+4.22612	+1.21717
H	−5.33604	+3.36468	+0.90997
H	−4.32006	+6.87045	+0.37980
C	−3.28569	+5.73930	+1.96555
O	−1.96729	+6.10392	+1.50433
H	−1.30262	+5.82505	+2.18961
H	−3.61967	+6.47584	+2.75181
C	−3.27858	+4.33528	+2.56749
O	−2.45299	+4.37530	+3.75670
H	−2.63822	+3.54544	+4.28903
H	−2.84544	+3.60486	+1.82941
C	−4.70724	+3.90683	+2.93039
H	−5.07962	+4.51198	+3.79918
O	−4.65355	+2.47838	+3.31753
C	−5.08240	+2.18733	+4.68123
O	−6.27897	+1.35091	+4.62155
C	−6.06065	+0.06915	+3.90252
C	−7.44046	−0.59601	+3.90281
O	−7.50036	−1.69322	+2.99291
H	−6.94605	−2.42945	+3.35124
H	−7.69739	−0.93056	+4.94422
H	−8.20159	+0.15048	+3.58929
H	−5.73024	+0.29931	+2.85654
H	−5.38210	+3.13176	+5.21003
C	−3.99195	+1.43571	+5.43656
O	−2.81714	+2.27296	+5.47543

H	−2.03870	+1.71767	+5.74943
H	−4.34774	+1.24769	+6.49040
C	−3.68281	+0.09692	+4.76883
O	−2.73153	−0.60142	+5.60761
H	−2.72263	−1.56615	+5.33124
H	−3.23537	+0.27091	+3.74991
C	−4.96753	−0.72999	+4.62521
H	−5.32755	−1.06045	+5.63551
O	−4.63175	−1.92041	+3.81226
C	−4.84567	−3.20778	+4.46579
O	−5.88088	−3.93027	+3.73041
H	−5.23728	−3.06070	+5.50807
C	−3.56470	−4.03494	+4.44859
O	−2.54994	−3.30482	+5.17289
H	−1.66298	−3.69772	+4.95530
H	−3.76047	−5.01447	+4.97163
C	−3.10767	−4.31052	+3.01767
O	−1.97640	−5.21246	+3.08372
H	−1.80913	−5.56191	+2.15762
H	−2.79454	−3.34744	+2.52883
C	−4.23985	−4.94288	+2.19669
H	−4.41370	−6.00088	+2.52788
C	−5.53596	−4.12767	+2.29866
H	−5.38438	−3.12182	+1.83051
C	−6.76534	−4.82306	+1.70631
H	−6.81904	−5.88051	+2.08381
H	−7.68136	−4.29499	+2.04809
O	−6.75167	−4.79789	+0.27971
H	−6.01021	−5.36890	−0.03997
O	+7.34514	−3.84390	−1.43195
H	+6.60711	−4.46472	−1.21531
C	+7.26242	−3.49320	−2.81074
H	+7.36185	−4.40714	−3.46091
H	+8.11765	−2.82236	−3.04303
C	+5.95455	−2.76951	−3.14844
H	+5.74497	−1.95391	−2.41025
O	+6.22550	−2.14950	−4.47310
C	+5.09856	−1.38635	−5.00142
O	+4.76468	−0.33231	−4.04801
H	+5.44360	−0.94280	−5.97397

C	+3.91441	−2.32665	−5.19152
O	+2.81300	−1.56538	−5.73117
H	+1.97947	−2.09768	−5.62514
H	+4.20510	−3.12958	−5.92836
C	+3.52498	−2.97786	−3.86716
O	+2.46949	−3.93112	−4.13951
H	+2.39415	−4.54363	−3.34768
H	+3.15366	−2.19385	−3.14878
C	+4.73489	−3.69626	−3.25622
H	+4.98667	−4.60608	−3.86389
O	+4.33790	−4.11221	−1.89379
C	+4.40813	−5.54315	−1.62484
O	+5.38950	−5.75911	−0.56445
C	+5.06048	−4.98849	+0.66438
C	+6.24977	−5.21768	+1.59852
O	+6.22397	−4.29915	+2.69943
H	+5.50529	−4.58717	+3.31552
H	+6.25377	−6.27482	+1.97472
H	+7.19294	−5.05044	+1.03636
H	+4.98933	−3.89867	+0.39903
H	+4.77953	−6.09924	−2.52725
C	+3.05290	−6.05880	−1.15107
O	+2.08944	−5.83262	−2.20268
H	+1.17757	−5.92296	−1.81600
H	+3.13552	−7.16699	−0.95744
C	+2.61962	−5.35842	+0.13601
O	+1.39979	−5.99302	+0.59004
H	+1.25813	−5.73977	+1.54946
H	+2.42881	−4.26707	−0.06685
C	+3.71464	−5.49209	+1.20212
H	+3.79951	−6.56101	+1.53479
O	+3.30653	−4.65570	+2.35537
C	+3.13279	−5.37320	+3.61068
O	+4.12553	−4.87620	+4.56480
C	+4.00698	−3.41732	+4.81155
C	+5.16883	−3.09950	+5.75753
O	+5.41059	−1.69504	+5.84084
H	+4.67060	−1.28146	+6.35033
H	+4.95701	−3.52523	+6.77469
H	+6.09240	−3.58402	+5.37320

H	+4.13219	−2.88100	+3.83476
H	+3.34486	−6.46756	+3.47513
C	+1.73948	−5.13074	+4.18177
O	+0.77067	−5.62275	+3.23263
H	−0.12827	−5.28697	+3.49482
H	+1.64044	−5.70880	+5.14545
C	+1.51091	−3.64709	+4.46623
O	+0.22015	−3.52008	+5.10900
H	+0.17089	−2.61008	+5.52845
H	+1.51519	−3.06569	+3.50224
C	+2.61725	−3.11424	+5.38627
H	+2.51251	−3.55817	+6.41154
O	+2.45429	−1.64590	+5.46925
C	+2.20738	−1.12149	+6.80738
O	+3.32708	−0.25786	+7.17694
C	+3.52733	+0.86876	+6.22939
C	+4.76661	+1.59243	+6.76504
O	+5.30277	+2.50385	+5.80723
H	+4.67606	+3.26252	+5.70937
H	+4.50882	+2.12649	+7.71937
H	+5.55152	+0.84123	+6.99901
H	+3.72364	+0.44467	+5.21124
H	+2.18527	−1.95378	+7.56090
C	+0.92339	−0.30034	+6.82460
O	−0.16889	−1.17045	+6.46049
H	−0.96213	−0.61188	+6.24000
H	+0.75537	+0.08815	+7.87001
C	+1.02038	+0.88158	+5.86172
O	−0.18180	+1.67143	+6.01807
H	−0.02228	+2.56858	+5.59730
H	+1.09674	+0.50851	+4.80187
C	+2.25688	+1.72851	+6.19150
H	+2.11147	+2.25579	+7.17142
O	+2.40029	+2.73041	+5.11243
C	+2.33371	+4.12354	+5.53982
O	+3.62187	+4.75215	+5.25627
C	+3.99681	+4.67336	+3.82071
C	+5.38673	+5.31391	+3.75748
O	+6.04890	+5.01925	+2.52850
H	+5.58559	+5.49842	+1.79770

H	+5.29835	+6.42459	+3.90420
H	+6.00876	+4.91152	+4.58583
H	+4.04431	+3.59327	+3.52601
H	+2.18318	+4.18999	+6.65068
C	+1.24549	+4.86206	+4.76913
O	−0.01948	+4.23195	+5.06412
H	−0.69264	+4.54442	+4.40134
H	+1.21648	+5.93336	+5.12012
C	+1.52736	+4.83371	+3.26806
O	+0.52466	+5.65169	+2.61972
H	+0.84421	+5.85668	+1.69037
H	+1.46101	+3.77634	+2.88623
C	+2.92928	+5.38873	+2.98187
H	+2.95231	+6.49329	+3.17922
O	+3.21223	+5.14071	+1.55071
C	+3.47787	+6.33289	+0.75210
O	+4.84620	+6.25334	+0.24746
C	+5.09007	+5.04022	−0.57598
C	+6.57798	+5.11994	−0.93026
O	+7.06507	+3.88413	−1.45091
H	+6.66228	+3.73778	−2.34208
H	+6.74674	+5.95364	−1.66473
H	+7.15696	+5.35600	−0.01151
H	+4.87876	+4.13825	+0.05262
H	+3.42717	+7.25499	+1.39128
C	+2.51751	+6.40430	−0.42940
O	+1.17241	+6.47361	+0.09064
H	+0.53629	+6.27089	−0.64708
H	+2.73603	+7.34233	−1.01640
C	+2.68271	+5.19258	−1.34491
O	+1.83020	+5.39890	−2.49617
H	+2.11974	+4.76167	−3.21538
H	+2.37089	+4.25639	−0.80249
C	+4.14666	+5.06166	−1.78626
H	+4.41643	+5.90558	−2.47542
O	+4.27415	+3.77830	−2.51110
C	+4.71401	+3.88608	−3.89741
O	+6.00219	+3.20855	−4.02578
H	+4.89128	+4.96054	−4.17282
C	+3.71158	+3.21723	−4.83102

O	+2.44484	+3.89571	−4.69514
H	+1.73978	+3.33812	−5.12136
H	+4.07837	+3.32895	−5.89188
C	+3.56665	+1.73118	−4.50845
O	+2.69190	+1.15522	−5.50823
H	+2.79012	+0.15703	−5.46994
H	+3.11368	+1.60043	−3.48618
C	+4.94120	+1.04975	−4.54795
H	+5.32182	+1.01623	−5.60334
C	+5.94648	+1.77366	−3.64192
H	+5.61193	+1.70514	−2.57582
C	+7.39292	+1.29225	−3.79206
H	+7.66589	+1.25655	−4.88189
H	+8.07005	+2.02184	−3.29777
O	+7.59646	+0.02019	−3.18014
H	+7.10738	−0.66306	−3.70082

345

* E = 1.414 kcal/mol

C	+0.76709	−0.61995	−0.57887
C	+1.01645	−0.81444	+0.72107
C	−0.01691	−0.93674	+1.74803
C	−1.29728	−0.54155	+1.78910
C	−2.13137	+0.20023	+0.83714
C	−1.81821	+1.38833	+0.29447
C	−0.55286	+2.12175	+0.57431
H	−0.75881	+3.18053	+0.73616
C	−2.73290	+2.09548	−0.66425
C	−3.92642	+1.27795	−1.11568
C	−4.50121	+0.52928	+0.06686
C	−3.47061	−0.44984	+0.58565
C	+1.81217	−0.54738	−1.64440
C	+3.20590	−0.95612	−1.17882
C	+3.46111	−0.42093	+0.21544
C	+2.43625	−1.05572	+1.15109
C	+4.75560	−0.86985	+0.89696
C	+4.45229	−0.64572	+2.39121
C	+2.91692	−0.63636	+2.53415
O	−4.89257	+1.42517	+1.08348
H	−4.10212	+1.76543	+1.50613
C	+3.36526	+1.10224	+0.21714

C	+6.03034	−0.20920	+0.42272
H	−5.40678	+0.00070	−0.21449
H	−3.35429	−1.27881	−0.11530
H	−3.83794	−0.88696	+1.51661
H	−3.62479	+0.55615	−1.87502
H	−4.68173	+1.92413	−1.55514
H	−3.07169	+3.02687	−0.19901
H	−2.14523	+2.41334	−1.52850
H	+0.11783	+2.05840	−0.28504
H	−0.02190	+1.72700	+1.43438
H	−1.84841	−0.87576	+2.66254
H	+0.31776	−1.47067	+2.63027
H	−0.25687	−0.49076	−0.90171
H	+1.83668	+0.47657	−2.03368
H	+1.50071	−1.15936	−2.49303
H	+3.27974	−2.04563	−1.15440
H	+3.95336	−0.60012	−1.88976
H	+4.84096	−1.94556	+0.71976
H	+4.87570	+0.30249	+2.72125
H	+4.91138	−1.41692	+3.00405
H	+2.54673	+0.35880	+2.78275
H	+2.57021	−1.30733	+3.31791
H	+3.69982	+1.52742	+1.16152
H	+2.34407	+1.43848	+0.05181
H	+3.99195	+1.52102	−0.57018
H	+6.89225	−0.62398	+0.93966
H	+6.01820	+0.86234	+0.61588
H	+6.18521	−0.35816	−0.64526
H	+2.60349	−2.13827	+1.08636
O	−6.22386	−3.11588	−4.52331
H	−5.44262	−3.16461	−5.12716
C	−6.26336	−4.30437	−3.73451
H	−6.22684	−5.21620	−4.39011
H	−7.22999	−4.31928	−3.18671
C	−5.11530	−4.35315	−2.72195
H	−5.05982	−3.39938	−2.13793
O	−5.49526	−5.44976	−1.79453
C	−4.54885	−5.61923	−0.69372
O	−4.47447	−4.36359	+0.04551
H	−4.96145	−6.43632	−0.04312

C	−3.18570	−5.98479	−1.27066
O	−2.25974	−6.14875	−0.17628
H	−1.33441	−6.13692	−0.54163
H	−3.28101	−6.96295	−1.82458
C	−2.69301	−4.91104	−2.23875
O	−1.46057	−5.38862	−2.82891
H	−1.26838	−4.83111	−3.64031
H	−2.50205	−3.95083	−1.68309
C	−3.74216	−4.66707	−3.33147
H	−3.80979	−5.56170	−4.00567
O	−3.29108	−3.49635	−4.11649
C	−3.04906	−3.74908	−5.53235
O	−3.98909	−2.94337	−6.30892
C	−3.87697	−1.49082	−6.01793
C	−4.98044	−0.85047	−6.86539
O	−5.24062	+0.49474	−6.46658
H	−4.46123	+1.05239	−6.70992
H	−4.69589	−0.89382	−7.95173
H	−5.91858	−1.43211	−6.73754
H	−4.07344	−1.33339	−4.92701
H	−3.26263	−4.82212	−5.78543
C	−1.62957	−3.34307	−5.91228
O	−0.70818	−4.15285	−5.15197
H	+0.20240	−3.76113	−5.23776
H	−1.47890	−3.54444	−7.01165
C	−1.39647	−1.85832	−5.63905
O	−0.07519	−1.52703	−6.12947
H	−0.01018	−0.52776	−6.19386
H	−1.45209	−1.65931	−4.53244
C	−2.45786	−1.01646	−6.36045
H	−2.28962	−1.05480	−7.46940
O	−2.30307	+0.37802	−5.88987
C	−1.99577	+1.35691	−6.92601
O	−3.09548	+2.31688	−6.99130
C	−3.33776	+3.00374	−5.69623
C	−4.56041	+3.88709	−5.96179
O	−5.13046	+4.37859	−4.74958
H	−4.50990	+5.04007	−4.35586
H	−4.27264	+4.73729	−6.63772
H	−5.33458	+3.28539	−6.48467

H	−3.56936	+2.22789	−4.92298
H	−1.94183	+0.86382	−7.93350
C	−0.71159	+2.10407	−6.58503
O	+0.36483	+1.14415	−6.52802
H	+1.14754	+1.56893	−6.08484
H	−0.49886	+2.85050	−7.40332
C	−0.84871	+2.84564	−5.25628
O	+0.35982	+3.61808	−5.06309
H	+0.18056	+4.30587	−4.35459
H	−0.96715	+2.10712	−4.41471
C	−2.07120	+3.77265	−5.29552
H	−1.88718	+4.61901	−6.00920
O	−2.25483	+4.31570	−3.93064
C	−2.17893	+5.76918	−3.82413
O	−3.47640	+6.26752	−3.37474
C	−3.89502	+5.70179	−2.06610
C	−5.28667	+6.29652	−1.82824
O	−5.97885	+5.62260	−0.77937
H	−5.53795	+5.83580	+0.07926
H	−5.19359	+7.39349	−1.60222
H	−5.88772	+6.19220	−2.75718
H	−3.94985	+4.58738	−2.16320
H	−1.99344	+6.22904	−4.83180
C	−1.11624	+6.17532	−2.80931
O	+0.15691	+5.66985	−3.26482
H	+0.80387	+5.71223	−2.51053
H	−1.07253	+7.30146	−2.76445
C	−1.44655	+5.63374	−1.41953
O	−0.45956	+6.16273	−0.50247
H	−0.80467	+6.03985	+0.43206
H	−1.39557	+4.50854	−1.42472
C	−2.85517	+6.07791	−1.00191
H	−2.86853	+7.18488	−0.81818
O	−3.18943	+5.37022	+0.25540
C	−3.45062	+6.23488	+1.40476
O	−4.83584	+6.04806	+1.82263
C	−5.16206	+4.64871	+2.20957
C	−6.64350	+4.71158	+2.59819
O	−7.24676	+3.42182	+2.63105
H	−6.79985	+2.87611	+3.32341

H	−6.74673	+5.22670	+3.59419
H	−7.18824	+5.32332	+1.84674
H	−5.00973	+3.98788	+1.31875
H	−3.35033	+7.31478	+1.11304
C	−2.52981	+5.87025	+2.56318
O	−1.16532	+6.06711	+2.13549
H	−0.56166	+5.61027	+2.78114
H	−2.74901	+6.55772	+3.42986
C	−2.75738	+4.42525	+2.99930
O	−1.92871	+4.17932	+4.16029
H	−2.26861	+3.35135	+4.61543
H	−2.46394	+3.72439	+2.16687
C	−4.23428	+4.20616	+3.35048
H	−4.48761	+4.75381	+4.29690
O	−4.40696	+2.75273	+3.56546
C	−4.91611	+2.36438	+4.87943
O	−6.20924	+1.71697	+4.70358
C	−6.15676	+0.51701	+3.82064
C	−7.61220	+0.04321	+3.76502
O	−7.82205	−0.91618	+2.72900
H	−7.32122	−1.73769	+2.95459
H	−7.91042	−0.38200	+4.76304
H	−8.26931	+0.91529	+3.55969
H	−5.79602	+0.83168	+2.80321
H	−5.09519	+3.27313	+5.51484
C	−3.95463	+1.38311	+5.53974
O	−2.67713	+2.03895	+5.69895
H	−1.99025	+1.34560	+5.88913
H	−4.35733	+1.10544	+6.55566
C	−3.80929	+0.12126	+4.69286
O	−2.96926	−0.80416	+5.42466
H	−3.07784	−1.71284	+5.01363
H	−3.32384	+0.37780	+3.70954
C	−5.18468	−0.50459	+4.42824
H	−5.60206	−0.93065	+5.37952
O	−4.97556	−1.60308	+3.45455
C	−5.35944	−2.93122	+3.91814
O	−6.45826	−3.41126	+3.08243
H	−5.75431	−2.88439	+4.96835
C	−4.19043	−3.90219	+3.79203

O	−3.10817	−3.41566	+4.61377
H	−2.27831	−3.90309	+4.36271
H	−4.51921	−4.91160	+4.17314
C	−3.74110	−4.04169	+2.33830
O	−2.72623	−5.07309	+2.29537
H	−2.61055	−5.35525	+1.33941
H	−3.30858	−3.06713	+1.97522
C	−4.93207	−4.43165	+1.45222
H	−5.25628	−5.48042	+1.68532
C	−6.10397	−3.45831	+1.64102
H	−5.79819	−2.43023	+1.31823
C	−7.40185	−3.88113	+0.94699
H	−7.62997	−4.95323	+1.19365
H	−8.23763	−3.25867	+1.33199
O	−7.32980	−3.69503	−0.46643
H	−6.69873	−4.35898	−0.83831
O	+6.68059	−4.57217	−1.80114
H	+5.92103	−5.20112	−1.72630
C	+6.74895	−4.09610	−3.14457
H	+6.80486	−4.95511	−3.86742
H	+7.68365	−3.50437	−3.24960
C	+5.55162	−3.20690	−3.49358
H	+5.39000	−2.43078	−2.70257
O	+5.95790	−2.52036	−4.74714
C	+4.95768	−1.56448	−5.21657
O	+4.71784	−0.59307	−4.15430
H	+5.39475	−1.06141	−6.12046
C	+3.67439	−2.31945	−5.54599
O	+2.69675	−1.36113	−6.00753
H	+1.79863	−1.78669	−5.97922
H	+3.88965	−3.05631	−6.37178
C	+3.15341	−3.07063	−4.32268
O	+2.02208	−3.86990	−4.74560
H	+1.81435	−4.51971	−4.00941
H	+2.82343	−2.33435	−3.53917
C	+4.24522	−3.97498	−3.73445
H	+4.42445	−4.85283	−4.41018
O	+3.74618	−4.45596	−2.42631
C	+3.59913	−5.90132	−2.30508
O	+4.51813	−6.36906	−1.26923

C	+4.27574	−5.72625	+0.04868
C	+5.38509	−6.28037	+0.94726
O	+5.49797	−5.54202	+2.16333
H	+4.70030	−5.72805	+2.71741
H	+5.19177	−7.36656	+1.15999
H	+6.35565	−6.20741	+0.41112
H	+4.38036	−4.61784	−0.07353
H	+3.90588	−6.41132	−3.25755
C	+2.17417	−6.25912	−1.89872
O	+1.27894	−5.78334	−2.92646
H	+0.35192	−5.79582	−2.56531
H	+2.09123	−7.38182	−1.82689
C	+1.81786	−5.64617	−0.54517
O	+0.50551	−6.13487	−0.17975
H	+0.37639	−5.97455	+0.80225
H	+1.79882	−4.52340	−0.62357
C	+2.85141	−6.06645	+0.50824
H	+2.76263	−7.16615	+0.71431
O	+2.55053	−5.31235	+1.74600
C	+2.24420	−6.13248	+2.91291
O	+3.27048	−5.89315	+3.92552
C	+3.35188	−4.47062	+4.34629
C	+4.52423	−4.43933	+5.33123
O	+4.93197	−3.10399	+5.62662
H	+4.22929	−2.67570	+6.17438
H	+4.24063	−4.98359	+6.27214
H	+5.39019	−4.97008	+4.88042
H	+3.57344	−3.84510	+3.44406
H	+2.30356	−7.22374	+2.65383
C	+0.88349	−5.76087	+3.49216
O	−0.11735	−5.98840	+2.47796
H	−0.95768	−5.53383	+2.75541
H	+0.67493	−6.43214	+4.37460
C	+0.85767	−4.30667	+3.95903
O	−0.41885	−4.08151	+4.60306
H	−0.35595	−3.23072	+5.13149
H	+0.96604	−3.61512	+3.07665
C	+2.00341	−4.05196	+4.94739
H	+1.81500	−4.60370	+5.90623
O	+2.03453	−2.59744	+5.21862

C	+1.80564	−2.21511	+6.60858
O	+3.01555	−1.56989	+7.11319
C	+3.40989	−0.37798	+6.31784
C	+4.70602	+0.10324	+6.97724
O	+5.41918	+1.01324	+6.14044
H	+4.90024	+1.85226	+6.07091
H	+4.47329	+0.57663	+7.96983
H	+5.36331	−0.77271	+7.16622
H	+3.60087	−0.70388	+5.26402
H	+1.63927	−3.12465	+7.24583
C	+0.64766	−1.22854	+6.70505
O	−0.54518	−1.88149	+6.22149
H	−1.24123	−1.18878	+6.06127
H	+0.50278	−0.94713	+7.78754
C	+0.93914	+0.03205	+5.89391
O	−0.14557	+0.96210	+6.12667
H	+0.15142	+1.86809	+5.81387
H	+0.99439	−0.22172	+4.79816
C	+2.27075	+0.65043	+6.34063
H	+2.16305	+1.09367	+7.36618
O	+2.59502	+1.72337	+5.37503
C	+2.72519	+3.05892	+5.94327
O	+4.09600	+3.51620	+5.72381
C	+4.47472	+3.52137	+4.28724
C	+5.94703	+3.94277	+4.28746
O	+6.56683	+3.69838	+3.02526
H	+6.19175	+4.33068	+2.36398
H	+6.02965	+5.02828	+4.56553
H	+6.49206	+3.35360	+5.05595
H	+4.35997	+2.48145	+3.88657
H	+2.56981	+3.03477	+7.05516
C	+1.76910	+4.02309	+5.25064
O	+0.42057	+3.56298	+5.48101
H	−0.18607	+4.02891	+4.84509
H	+1.89388	+5.04744	+5.70595
C	+2.06725	+4.10145	+3.75432
O	+1.20250	+5.11623	+3.19107
H	+1.56643	+5.37152	+2.29160
H	+1.85615	+3.10815	+3.26786
C	+3.53903	+4.47493	+3.53134

H	+3.71869	+5.53436	+3.85544
O	+3.80715	+4.35583	+2.08085
C	+4.24551	+5.58302	+1.42615
O	+5.59837	+5.37328	+0.91493
C	+5.68587	+4.24089	−0.04307
C	+7.17642	+4.15294	−0.38364
O	+7.49586	+2.92859	−1.04365
H	+7.10053	+2.94952	−1.94970
H	+7.47241	+5.03282	−1.01658
H	+7.76603	+4.19855	+0.55715
H	+5.34232	+3.30942	+0.47588
H	+4.31228	+6.42433	+2.16711
C	+3.32626	+5.92339	+0.25859
O	+1.99497	+6.12741	+0.77722
H	+1.35348	+6.11744	+0.01716
H	+3.68800	+6.87970	−0.21787
C	+3.33494	+4.81095	−0.78836
O	+2.53327	+5.26138	−1.90645
H	+2.74541	+4.67935	−2.69562
H	+2.89096	+3.87127	−0.35565
C	+4.77386	+4.53184	−1.24299
H	+5.16716	+5.40484	−1.82856
O	+4.73646	+3.33619	−2.11527
C	+5.21312	+3.54554	−3.47815
O	+6.40346	+2.72300	−3.68128
H	+5.53513	+4.61155	−3.62373
C	+4.14815	+3.13118	−4.48775
O	+2.97842	+3.94594	−4.26366
H	+2.20812	+3.53827	−4.74325
H	+4.54186	+3.32650	−5.52667
C	+3.80996	+1.64718	−4.35505
O	+2.88961	+1.31197	−5.42080
H	+2.87067	+0.31294	−5.51412
H	+3.32454	+1.45101	−3.35736
C	+5.08675	+0.80411	−4.47248
H	+5.48633	+0.85291	−5.52001
C	+6.14999	+1.27448	−3.47061
H	+5.77332	+1.12747	−2.42529
C	+7.52525	+0.62419	−3.64769
H	+7.82831	+0.66844	−4.72842

H	+8.27480	+1.19796	−3.06136
O	+7.53633	−0.72300	−3.17710
H	+7.00804	−1.27739	−3.80249

345

* E = 1.910 kcal/mol

C	+0.86310	−1.34684	+0.06917
C	+0.75399	−0.16973	−0.55681
C	+1.86680	+0.54736	−1.17508
C	+3.19393	+0.48715	−0.99167
C	+4.05670	−0.26919	−0.08043
C	+3.94046	−0.30232	+1.25502
C	+2.85006	+0.37702	+2.00705
H	+3.25391	+0.87542	+2.88954
C	+4.94869	−0.99101	+2.12258
C	+5.91536	−1.88732	+1.37813
C	+6.40479	−1.19239	+0.13099
C	+5.22787	−0.90949	−0.77768
C	−0.27758	−2.09556	+0.68229
C	−1.65128	−1.50621	+0.37598
C	−1.58397	+0.00693	+0.37137
C	−0.61705	+0.42225	−0.73341
C	−2.84593	+0.74413	−0.08222
C	−2.30571	+2.12093	−0.51336
C	−0.80643	+1.93163	−0.81753
O	+7.03296	−0.00162	+0.54264
H	+7.21886	+0.54463	−0.22214
C	−1.13523	+0.51944	+1.73566
C	−3.98025	+0.81848	+0.91604
H	+7.11807	−1.82871	−0.39632
H	+4.91225	−1.83578	−1.26511
H	+5.55443	−0.25769	−1.59173
H	+5.42812	−2.81749	+1.08577
H	+6.75599	−2.14394	+2.01746
H	+5.50710	−0.21645	+2.65183
H	+4.42422	−1.56142	+2.89309
H	+2.12215	−0.35582	+2.36490
H	+2.31242	+1.10213	+1.40340
H	+3.76469	+1.09965	−1.68495
H	+1.55020	+1.24928	−1.93860
H	+1.84250	−1.79374	+0.17244

H	−0.12058	−2.12631	+1.76600
H	−0.23877	−3.13928	+0.36531
H	−1.98624	−1.84302	−0.60768
H	−2.38173	−1.86959	+1.10086
H	−3.20744	+0.22165	−0.97198
H	−2.44971	+2.84726	+0.28764
H	−2.85076	+2.50371	−1.37328
H	−0.18488	+2.43651	−0.07828
H	−0.52937	+2.33484	−1.78920
H	−1.21190	+1.60342	+1.80596
H	−0.10351	+0.24902	+1.94403
H	−1.76151	+0.09773	+2.52137
H	−4.84650	+1.31018	+0.47966
H	−3.69327	+1.38681	+1.80062
H	−4.29367	−0.17328	+1.24022
H	−1.02952	+0.00956	−1.66235
O	−6.99587	−2.75556	−3.62542
H	−6.28841	−2.81505	−4.31365
C	−6.99065	−3.96262	−2.86428
H	−7.05919	−4.85768	−3.54031
H	−7.89097	−3.96015	−2.21301
C	−5.74049	−4.07468	−1.98670
H	−5.58066	−3.13373	−1.40159
O	−6.05886	−5.17064	−1.03475
C	−5.00661	−5.38531	−0.04344
O	−4.80942	−4.14204	+0.69265
H	−5.37503	−6.19561	+0.64147
C	−3.72503	−5.78780	−0.76447
O	−2.69397	−5.98666	+0.22581
H	−1.81350	−6.01137	−0.23699
H	−3.90808	−6.75935	−1.30782
C	−3.30407	−4.72401	−1.77734
O	−2.16005	−5.24000	−2.49858
H	−2.05339	−4.70300	−3.33964
H	−3.02016	−3.77360	−1.24322
C	−4.45794	−4.43828	−2.74738
H	−4.63562	−5.33058	−3.40459
O	−4.05277	−3.28771	−3.58566
C	−4.01213	−3.54766	−5.02079
O	−4.99431	−2.68459	−5.67276

C	−4.75031	−1.23929	−5.42911
C	−5.91850	−0.54046	−6.13105
O	−6.01483	+0.83400	−5.76041
H	−5.24246	+1.31803	−6.14299
H	−5.80456	−0.64340	−7.24426
H	−6.86717	−1.04057	−5.84003
H	−4.78203	−1.05619	−4.32508
H	−4.32338	−4.60447	−5.23909
C	−2.63060	−3.23369	−5.58415
O	−1.67045	−4.08923	−4.92949
H	−0.75811	−3.73404	−5.10763
H	−2.63359	−3.45879	−6.68952
C	−2.27347	−1.76314	−5.37686
O	−1.01036	−1.52422	−6.04234
H	−0.89151	−0.53239	−6.13591
H	−2.17205	−1.54333	−4.27747
C	−3.36553	−0.86744	−5.97672
H	−3.35491	−0.94670	−7.09619
O	−3.05617	+0.52490	−5.58229
C	−2.80817	+1.44597	−6.68573
O	−3.84381	+2.47646	−6.66542
C	−3.89042	+3.22466	−5.38241
C	−5.06367	+4.19328	−5.55958
O	−5.46177	+4.77839	−4.32086
H	−4.74444	+5.38515	−4.01301
H	−4.78573	+4.98941	−6.30278
H	−5.93360	+3.63657	−5.96961
H	−4.09619	+2.49747	−4.55649
H	−2.90341	+0.91704	−7.67185
C	−1.44915	+2.11764	−6.52607
O	−0.43071	+1.09604	−6.56483
H	+0.42330	+1.49052	−6.24128
H	−1.29419	+2.82727	−7.38888
C	−1.37984	+2.90014	−5.21646
O	−0.10964	+3.59440	−5.18928
H	−0.15317	+4.29144	−4.46855
H	−1.44506	+2.19351	−4.34239
C	−2.53600	+3.90675	−5.14371
H	−2.37566	+4.73014	−5.88937
O	−2.53155	+4.47528	−3.77783

C	−2.32551	+5.91663	−3.70016
O	−3.52745	+6.51975	−3.12800
C	−3.86304	+5.96421	−1.79136
C	−5.17804	+6.65285	−1.41405
O	−5.82812	+5.99259	−0.32873
H	−5.29215	+6.13120	+0.49074
H	−4.98232	+7.73066	−1.16244
H	−5.86345	+6.62839	−2.28834
H	−4.01003	+4.85901	−1.89529
H	−2.20402	+6.35581	−4.72659
C	−1.13996	+6.23547	−2.79639
O	+0.04106	+5.64032	−3.37611
H	+0.76377	+5.63784	−2.69266
H	−1.00779	+7.35470	−2.75488
C	−1.37376	+5.70553	−1.38275
O	−0.26926	+6.15587	−0.56165
H	−0.53640	+6.05318	+0.40021
H	−1.40351	+4.58066	−1.39605
C	−2.70062	+6.24253	−0.82867
H	−2.61496	+7.34350	−0.62817
O	−2.96310	+5.53281	+0.44358
C	−3.08594	+6.38751	+1.61877
O	−4.43619	+6.23924	+2.15700
C	−4.75935	+4.83941	+2.53644
C	−6.22229	+4.90226	+2.98533
O	−6.79600	+3.60120	+3.10458
H	−6.39059	+3.15070	+3.88567
H	−6.29438	+5.45750	+3.95939
H	−6.80850	+5.46710	+2.22895
H	−4.64854	+4.19170	+1.62970
H	−2.97433	+7.46827	+1.33443
C	−2.07935	+5.97268	+2.68561
O	−0.75506	+6.11564	+2.13097
H	−0.11041	+5.63537	+2.71787
H	−2.18642	+6.66403	+3.57075
C	−2.32513	+4.53639	+3.14608
O	−1.40825	+4.26745	+4.23256
H	−1.73973	+3.46184	+4.73051
H	−2.13294	+3.81914	+2.29784
C	−3.77560	+4.38430	+3.62314

H	−3.93375	+4.97709	+4.56297
O	−4.00035	+2.94922	+3.90843
C	−4.38298	+2.64215	+5.28334
O	−5.72458	+2.06454	+5.27163
C	−5.82107	+0.83206	+4.44738
C	−7.30047	+0.44423	+4.53147
O	−7.64743	−0.54220	+3.56088
H	−7.20266	−1.39179	+3.80108
H	−7.53544	+0.07962	+5.56832
H	−7.92246	+1.34505	+4.34051
H	−5.53479	+1.08661	+3.39515
H	−4.44736	+3.58261	+5.89379
C	−3.41887	+1.63176	+5.89491
O	−2.09919	+2.21638	+5.90106
H	−1.43195	+1.49647	+6.06071
H	−3.73783	+1.42639	+6.95710
C	−3.42713	+0.32314	+5.10812
O	−2.58242	−0.61679	+5.81430
H	−2.76103	−1.53222	+5.44429
H	−3.01978	+0.49701	+4.07278
C	−4.85644	−0.22531	+5.00179
H	−5.20612	−0.58893	+6.00429
O	−4.81485	−1.36237	+4.05512
C	−5.20416	−2.65412	+4.60791
O	−6.41220	−3.10219	+3.91768
H	−5.47330	−2.55605	+5.69377
C	−4.10236	−3.68313	+4.38056
O	−2.91602	−3.23557	+5.07177
H	−2.13845	−3.75376	+4.73193
H	−4.43358	−4.66998	+4.81435
C	−3.82268	−3.85799	+2.88940
O	−2.85867	−4.92836	+2.74658
H	−2.85504	−5.21441	+1.78460
H	−3.39620	−2.90395	+2.46869
C	−5.11587	−4.20576	+2.13962
H	−5.44604	−5.24535	+2.40384
C	−6.23035	−3.19578	+2.44575
H	−5.93862	−2.18537	+2.06114
C	−7.61132	−3.59907	+1.92046
H	−7.82452	−4.66671	+2.20017

H	−8.38304	−2.96207	+2.40356
O	−7.71457	−3.41830	+0.50888
H	−7.12506	−4.07476	+0.06241
O	+6.06067	−4.80663	−2.46515
H	+5.29169	−5.39009	−2.24783
C	+5.96400	−4.42261	−3.83703
H	+5.85208	−5.32683	−4.49442
H	+6.91227	−3.91538	−4.11731
C	+4.79457	−3.46218	−4.07122
H	+4.79807	−2.64401	−3.30559
O	+5.06820	−2.85976	−5.40170
C	+4.08287	−1.84174	−5.76481
O	+4.07144	−0.83151	−4.71623
H	+4.42077	−1.40000	−6.74048
C	+2.71232	−2.50030	−5.87249
O	+1.75431	−1.48408	−6.24052
H	+0.84018	−1.84090	−6.07668
H	+2.74837	−3.28577	−6.68087
C	+2.32185	−3.15735	−4.54943
O	+1.07998	−3.87015	−4.76399
H	+0.95637	−4.51096	−4.00169
H	+2.17736	−2.37016	−3.75892
C	+3.41582	−4.13523	−4.09992
H	+3.43240	−5.03028	−4.77701
O	+3.07365	−4.56688	−2.72659
C	+2.89050	−6.00177	−2.55036
O	+3.90548	−6.47962	−1.61265
C	+3.83247	−5.80173	−0.29238
C	+5.02423	−6.36300	+0.48800
O	+5.28802	−5.59867	+1.66450
H	+4.56364	−5.77172	+2.31500
H	+4.83384	−7.43850	+0.74951
H	+5.92934	−6.32540	−0.15568
H	+3.94849	−4.69955	−0.45317
H	+3.06930	−6.54529	−3.51685
C	+1.50867	−6.29770	−1.97732
O	+0.52041	−5.80434	−2.90639
H	−0.35776	−5.76489	−2.44057
H	+1.39381	−7.41530	−1.87404
C	+1.33041	−5.65160	−0.60414

O	+0.05095	−6.08466	−0.08348
H	+0.04369	−5.91631	+0.90544
H	+1.34168	−4.52992	−0.70028
C	+2.46179	−6.09557	+0.33207
H	+2.36553	−7.19104	+0.55668
O	+2.32451	−5.32624	+1.58880
C	+2.12522	−6.13158	+2.78886
O	+3.26954	−5.93085	+3.67488
C	+3.45153	−4.51468	+4.08594
C	+4.71963	−4.53897	+4.94477
O	+5.21653	−3.22521	+5.19474
H	+4.59250	−2.76475	+5.80775
H	+4.50995	−5.07334	+5.91061
H	+5.50946	−5.10742	+4.40798
H	+3.60568	−3.89358	+3.16659
H	+2.11288	−7.22484	+2.53149
C	+0.85481	−5.70747	+3.51760
O	−0.26347	−5.90110	+2.62612
H	−1.05287	−5.42628	+3.00153
H	+0.72392	−6.36704	+4.42333
C	+0.93653	−4.25135	+3.97126
O	−0.25288	−3.97228	+4.74788
H	−0.10209	−3.11675	+5.25015
H	+0.97333	−3.56986	+3.07519
C	+2.19379	−4.03850	+4.82509
H	+2.08884	−4.57567	+5.80493
O	+2.31742	−2.58555	+5.07390
C	+2.25060	−2.18314	+6.47417
O	+3.53464	−1.59208	+6.84493
C	+3.90074	−0.43002	+5.99434
C	+5.27998	−0.00589	+6.50838
O	+5.93918	+0.86983	+5.59542
H	+5.45965	+1.73420	+5.58088
H	+5.17467	+0.47845	+7.51734
H	+5.91410	−0.91014	+6.63194
H	+3.96351	−0.77652	+4.93068
H	+2.10714	−3.07802	+7.13732
C	+1.15684	−1.14063	+6.67342
O	−0.10788	−1.74240	+6.32460
H	−0.78898	−1.02277	+6.23585

H	+1.13652	−0.84012	+7.76020
C	+1.42372	+0.09520	+5.81638
O	+0.41092	+1.07708	+6.14076
H	+0.71302	+1.96523	+5.78311
H	+1.35815	−0.17356	+4.72472
C	+2.82128	+0.65384	+6.11795
H	+2.83956	+1.11256	+7.14209
O	+3.10071	+1.69842	+5.10827
C	+3.33430	+3.03826	+5.63562
O	+4.69510	+3.43740	+5.28701
C	+4.95460	+3.40178	+3.82323
C	+6.42606	+3.80949	+3.69696
O	+6.96739	+3.46706	+2.42329
H	+6.49903	+3.99035	+1.72703
H	+6.52860	+4.91351	+3.88938
H	+7.01705	+3.27627	+4.47239
H	+4.80006	+2.35320	+3.46195
H	+3.27712	+3.03823	+6.75715
C	+2.35818	+4.02637	+5.00857
O	+1.01934	+3.63429	+5.38042
H	+0.37493	+4.09780	+4.78080
H	+2.56963	+5.05604	+5.41708
C	+2.51749	+4.04459	+3.49004
O	+1.64998	+5.07980	+2.96872
H	+1.94782	+5.28988	+2.03398
H	+2.21958	+3.04736	+3.06133
C	+3.97377	+4.34584	+3.11276
H	+4.21710	+5.41477	+3.35468
O	+4.08510	+4.13844	+1.65156
C	+4.56206	+5.28577	+0.89204
O	+5.82462	+4.91554	+0.25426
C	+5.67873	+3.74485	−0.64797
C	+7.11051	+3.40351	−1.06448
O	+7.16690	+2.15212	−1.75979
H	+6.78939	+2.29584	−2.66173
H	+7.52939	+4.22277	−1.70497
H	+7.74393	+3.31959	−0.15628
H	+5.22697	+2.90185	−0.06649
H	+4.79190	+6.14558	+1.57731
C	+3.55570	+5.67581	−0.18653

O	+2.30121	+5.97701	+0.46140
H	+1.58575	+6.00716	−0.22885
H	+3.93303	+6.60225	−0.70852
C	+3.37496	+4.56741	−1.22499
O	+2.52773	+5.09968	−2.27085
H	+2.60962	+4.50541	−3.07466
H	+2.88216	+3.67128	−0.75465
C	+4.74123	+4.15511	−1.79234
H	+5.19011	+5.00241	−2.37521
O	+4.52919	+3.00260	−2.69735
C	+4.87884	+3.23656	−4.09596
O	+6.00039	+2.36594	−4.44382
H	+5.24042	+4.28994	−4.23913
C	+3.70024	+2.91138	−5.00814
O	+2.60785	+3.79052	−4.66746
H	+1.77221	+3.42652	−5.06526
H	+4.01005	+3.10241	−6.07584
C	+3.28165	+1.45081	−4.86138
O	+2.23461	+1.18936	−5.82681
H	+2.11577	+0.19431	−5.88627
H	+2.89338	+1.26629	−3.82116
C	+4.47853	+0.52924	−5.12767
H	+4.73357	+0.52985	−6.22055
C	+5.69622	+0.92218	−4.28215
H	+5.46215	+0.73110	−3.20302
C	+6.99359	+0.21453	−4.68540
H	+7.11595	+0.25129	−5.80165
H	+7.85591	+0.75153	−4.23364
O	+7.02472	−1.13434	−4.21938
H	+6.34613	−1.65638	−4.71532

345

* E = 1.965 kcal/mol

C	−0.34030	−0.50586	−0.96327
C	−0.76704	+0.74314	−0.74205
C	+0.08449	+1.83439	−0.26773
C	+1.28826	+1.83330	+0.31945
C	+2.18884	+0.73904	+0.70786
C	+1.89456	−0.23523	+1.57862
C	+0.54938	−0.40460	+2.19668
H	+0.63835	−0.54457	+3.27467

C	+2.90419	−1.27099	+1.98086
C	+4.33104	−0.91640	+1.61113
C	+4.37301	−0.40453	+0.19312
C	+3.55760	+0.86472	+0.09000
C	−1.19228	−1.62067	−1.47600
C	−2.56605	−1.18066	−1.96878
C	−3.12405	−0.11330	−1.04997
C	−2.18412	+1.08818	−1.10792
C	−4.43182	+0.54823	−1.48826
C	−4.41909	+1.88178	−0.71694
C	−2.94865	+2.16730	−0.35200
O	+5.67605	−0.23167	−0.29950
H	+6.14450	+0.41774	+0.22458
C	−3.25698	−0.66076	+0.36823
C	−5.69404	−0.26069	−1.29292
H	+3.93051	−1.15755	−0.46190
H	+3.46663	+1.15029	−0.95844
H	+4.10227	+1.68066	+0.57916
H	+4.98470	−1.77882	+1.71427
H	+4.70308	−0.13766	+2.28033
H	+2.82508	−1.44055	+3.05576
H	+2.62687	−2.22494	+1.51988
H	+0.06160	−1.29921	+1.80173
H	−0.10525	+0.43807	+2.00205
H	+1.72491	+2.81550	+0.48057
H	−0.33442	+2.81781	−0.44564
H	+0.68873	−0.75408	−0.74600
H	−1.30442	−2.36035	−0.67568
H	−0.65505	−2.14733	−2.26684
H	−2.48008	−0.76588	−2.97539
H	−3.23112	−2.04322	−2.03453
H	−4.32536	+0.76554	−2.55446
H	−5.03056	+1.80044	+0.18204
H	−4.85255	+2.68194	−1.31151
H	−2.78158	+2.07367	+0.72159
H	−2.64276	+3.17325	−0.63363
H	−3.79860	+0.02419	+1.01822
H	−2.28355	−0.84066	+0.82050
H	−3.80570	−1.60227	+0.35597
H	−6.55760	+0.27436	−1.68005

H	−5.87856	−0.45816	−0.23774
H	−5.64053	−1.21785	−1.81017
H	−2.16955	+1.40478	−2.15819
O	−6.25982	−3.65472	−3.90594
H	−5.47716	−3.89334	−4.46131
C	−6.40125	−4.63322	−2.87712
H	−6.41594	−5.66821	−3.31409
H	−7.37811	−4.46334	−2.37536
C	−5.28180	−4.52935	−1.83684
H	−5.16310	−3.47149	−1.48577
O	−5.76714	−5.35230	−0.69900
C	−4.86284	−5.33258	+0.44858
O	−4.71001	−3.95077	+0.89130
H	−5.35209	−5.95072	+1.24855
C	−3.51710	−5.91195	+0.02666
O	−2.63525	−5.87384	+1.16858
H	−1.70254	−6.02025	+0.85509
H	−3.66957	−6.98450	−0.28775
C	−2.92304	−5.12936	−1.14315
O	−1.71580	−5.81178	−1.55809
H	−1.46816	−5.47899	−2.47197
H	−2.67394	−4.08084	−0.81535
C	−3.92486	−5.07347	−2.30412
H	−4.04688	−6.09297	−2.75708
O	−3.36590	−4.15143	−3.31779
C	−3.12856	−4.73466	−4.63437
O	−3.99183	−4.05682	−5.59873
C	−3.76880	−2.58830	−5.63531
C	−4.80945	−2.07265	−6.63366
O	−4.95630	−0.65510	−6.56006
H	−4.12304	−0.23358	−6.88549
H	−4.52287	−2.38799	−7.67376
H	−5.79481	−2.53042	−6.40075
H	−3.96160	−2.17641	−4.61192
H	−3.42555	−5.81772	−4.64530
C	−1.67542	−4.53668	−5.05213
O	−0.83611	−5.20413	−4.08407
H	+0.09750	−4.88072	−4.19773
H	−1.52297	−5.01056	−6.06384
C	−1.32369	−3.05263	−5.13374

O	+0.01583	−2.94940	−5.67314
H	+0.17340	−1.98973	−5.92182
H	−1.35303	−2.59935	−4.10460
C	−2.31503	−2.30685	−6.03764
H	−2.14387	−2.58476	−7.11128
O	−2.05820	−0.85992	−5.85775
C	−1.63740	−0.14471	−7.05660
O	−2.65837	+0.85148	−7.37622
C	−2.88667	+1.81854	−6.27107
C	−4.03227	+2.70082	−6.77539
O	−4.59442	+3.48856	−5.72648
H	−3.93169	+4.17170	−5.45846
H	−3.66422	+3.35728	−7.60981
H	−4.83617	+2.05174	−7.18429
H	−3.19745	+1.24704	−5.35960
H	−1.58647	−0.84211	−7.93549
C	−0.31191	+0.56869	−6.81589
O	+0.68559	−0.42453	−6.49732
H	+1.47256	+0.03685	−6.09979
H	−0.01340	+1.10163	−7.76442
C	−0.43455	+1.59226	−5.68824
O	+0.82372	+2.30262	−5.60974
H	+0.67543	+3.13918	−5.07674
H	−0.63878	+1.06876	−4.71289
C	−1.57785	+2.56995	−5.99122
H	−1.30664	+3.21721	−6.86710
O	−1.75923	+3.42114	−4.79336
C	−1.57187	+4.85072	−5.01341
O	−2.83947	+5.52729	−4.74559
C	−3.33131	+5.31097	−3.36061
C	−4.68590	+6.02497	−3.32977
O	−5.44385	+5.67264	−2.17303
H	−5.01428	+6.07950	−1.38093
H	−4.52800	+7.13630	−3.37590
H	−5.27159	+5.72822	−4.22625
H	−3.45837	+4.21048	−3.19652
H	−1.32508	+5.05488	−6.08989
C	−0.50824	+5.40726	−4.07297
O	+0.73304	+4.72381	−4.34232
H	+1.36766	+4.91104	−3.59900

H	−0.37962	+6.50715	−4.28916
C	−0.91615	+5.23493	−2.61073
O	+0.08284	+5.89865	−1.79969
H	−0.29862	+6.03040	−0.88107
H	−0.95523	+4.14091	−2.34557
C	−2.29489	+5.86507	−2.37349
H	−2.22829	+6.98174	−2.46306
O	−2.70752	+5.50933	−0.99753
C	−2.93848	+6.63753	−0.10127
O	−4.34596	+6.63294	+0.28996
C	−4.75691	+5.36232	+0.94167
C	−6.25502	+5.54186	+1.20518
O	−6.88986	+4.30214	+1.51617
H	−6.56361	+3.99267	+2.39692
H	−6.40683	+6.28406	+2.03552
H	−6.73720	+5.95248	+0.29208
H	−4.57911	+4.52274	+0.22258
H	−2.75773	+7.60913	−0.63455
C	−2.08115	+6.50512	+1.15243
O	−0.69428	+6.52015	+0.75157
H	−0.14069	+6.19796	+1.51271
H	−2.28263	+7.38915	+1.82293
C	−2.41110	+5.21831	+1.90605
O	−1.65904	+5.23042	+3.14349
H	−2.03839	+4.51874	+3.74052
H	−2.11084	+4.32627	+1.28891
C	−3.91476	+5.14545	+2.20612
H	−4.18622	+5.89958	+2.99182
O	−4.18843	+3.78421	+2.71837
C	−4.73279	+3.72113	+4.06870
O	−6.06985	+3.13492	+3.99655
C	−6.07298	+1.78123	+3.38378
C	−7.55458	+1.39712	+3.33064
O	−7.77798	+0.26603	+2.48967
H	−7.39800	−0.53292	+2.93134
H	−7.93078	+1.19785	+4.37050
H	−8.13486	+2.25022	+2.91799
H	−5.64548	+1.85832	+2.35056
H	−4.85912	+4.75283	+4.49405
C	−3.85678	+2.84100	+4.95283

O	−2.54183	+3.43296	+5.01388
H	−1.90995	+2.76399	+5.39231
H	−4.29962	+2.81539	+5.98991
C	−3.78605	+1.41622	+4.40540
O	−3.03799	+0.62149	+5.35614
H	−3.21760	−0.34664	+5.16280
H	−3.26173	+1.41226	+3.40889
C	−5.20205	+0.84907	+4.23738
H	−5.67302	+0.68988	+5.24367
O	−5.07434	−0.45344	+3.54650
C	−5.59011	−1.60205	+4.28312
O	−6.70144	−2.17323	+3.52584
H	−6.01202	−1.27941	+5.27271
C	−4.50812	−2.66400	+4.44483
O	−3.41713	−2.08360	+5.19102
H	−2.61882	−2.66835	+5.09243
H	−4.93665	−3.52894	+5.02834
C	−4.02560	−3.16857	+3.08618
O	−3.09966	−4.25512	+3.32892
H	−2.97505	−4.75377	+2.46718
H	−3.50198	−2.33981	+2.53377
C	−5.21567	−3.66442	+2.25326
H	−5.62984	−4.60697	+2.70021
C	−6.31181	−2.59463	+2.15545
H	−5.91882	−1.69987	+1.60711
C	−7.62094	−3.08311	+1.52817
H	−7.92920	−4.05302	+2.00460
H	−8.41969	−2.33750	+1.73046
O	−7.50680	−3.23156	+0.11356
H	−6.90938	−3.99680	−0.07421
O	+6.41603	−5.25423	−0.34299
H	+5.61611	−5.80516	−0.15924
C	+6.54884	−5.10297	−1.75628
H	+6.55683	−6.10669	−2.26230
H	+7.52631	−4.61456	−1.95672
C	+5.42966	−4.23890	−2.34434
H	+5.32050	−3.28472	−1.76953
O	+5.90359	−3.91072	−3.71331
C	+4.99480	−3.00733	−4.41727
O	+4.82685	−1.80817	−3.60952

H	+5.48121	−2.76282	−5.39956
C	+3.65573	−3.71342	−4.59861
O	+2.76763	−2.82296	−5.30730
H	+1.84380	−3.18653	−5.24786
H	+3.81592	−4.64436	−5.21453
C	+3.06249	−4.10270	−3.24558
O	+1.86001	−4.86881	−3.50031
H	+1.61027	−5.34400	−2.65296
H	+2.80509	−3.17599	−2.66053
C	+4.06893	−4.94284	−2.44719
H	+4.18688	−5.95317	−2.92164
O	+3.51467	−5.10588	−1.08383
C	+3.27523	−6.48186	−0.66774
O	+4.13076	−6.76948	+0.48234
C	+3.89700	−5.84137	+1.61820
C	+4.93925	−6.25358	+2.66167
O	+5.06528	−5.27857	+3.69646
H	+4.24146	−5.29337	+4.24290
H	+4.66705	−7.25489	+3.09251
H	+5.92794	−6.35524	+2.16505
H	+4.08210	−4.79522	+1.26428
H	+3.57646	−7.19967	−1.47707
C	+1.82018	−6.66633	−0.25188
O	+0.98498	−6.38437	−1.39451
H	+0.04773	−6.26942	−1.08207
H	+1.66720	−7.73866	+0.06280
C	+1.46664	−5.74873	+0.91741
O	+0.11420	−6.06486	+1.32408
H	−0.03517	−5.67792	+2.23763
H	+1.52490	−4.67199	+0.59261
C	+2.44010	−5.98161	+2.08073
H	+2.26902	−6.99729	+2.52633
O	+2.16038	−4.94696	+3.10234
C	+1.75419	−5.45646	+4.40769
O	+2.76477	−5.06769	+5.38899
C	+2.95491	−3.59687	+5.47486
C	+4.09193	−3.42484	+6.48653
O	+4.62320	−2.10064	+6.46533
H	+3.94814	−1.48545	+6.84432
H	+3.72480	−3.68788	+7.51531

H	+4.91370	−4.12742	+6.22991
H	+3.26078	−3.21916	+4.46541
H	+1.73120	−6.57922	+4.40330
C	+0.41271	−4.86162	+4.82112
O	−0.57323	−5.25012	+3.84176
H	−1.38700	−4.69228	+3.97060
H	+0.12456	−5.28728	+5.82519
C	+0.49643	−3.34022	+4.93148
O	−0.77545	−2.87045	+5.43764
H	−0.65193	−1.93095	+5.76835
H	+0.69224	−2.89043	+3.91749
C	+1.62856	−2.94484	+5.88886
H	+1.36205	−3.23509	+6.93962
O	+1.77558	−1.47401	+5.81009
C	+1.56791	−0.75939	+7.06570
O	+2.81996	−0.10358	+7.43483
C	+3.30575	+0.84100	+6.39585
C	+4.64250	+1.34720	+6.94588
O	+5.40410	+2.02420	+5.94671
H	+4.94933	+2.87433	+5.72699
H	+4.45760	+2.02032	+7.82646
H	+5.23967	+0.48032	+7.30176
H	+3.45768	+0.27144	+5.44393
H	+1.32908	−1.47908	+7.89409
C	+0.48533	+0.30169	+6.90307
O	−0.74164	−0.36322	+6.53345
H	−1.37926	+0.31625	+6.18505
H	+0.34026	+0.82346	+7.89239
C	+0.88086	+1.33216	+5.84735
O	−0.13163	+2.36669	+5.84614
H	+0.23299	+3.15577	+5.34473
H	+0.92628	+0.84325	+4.83429
C	+2.25202	+1.93578	+6.18009
H	+2.17513	+2.58995	+7.08880
O	+2.65769	+2.75680	+5.01804
C	+2.86339	+4.17345	+5.29142
O	+4.26845	+4.48822	+5.03719
C	+4.68433	+4.15778	+3.64982
C	+6.18372	+4.46716	+3.62024
O	+6.81357	+3.90945	+2.46644

H	+6.51945	+4.41992	+1.67178
H	+6.34283	+5.57833	+3.65905
H	+6.66298	+4.02394	+4.51943
H	+4.50229	+3.06651	+3.47757
H	+2.67585	+4.39979	+6.37532
C	+1.99690	+5.02544	+4.37042
O	+0.61367	+4.70760	+4.63362
H	+0.05751	+5.07718	+3.89599
H	+2.17860	+6.11293	+4.60799
C	+2.34139	+4.76982	+2.90405
O	+1.57527	+5.70369	+2.10565
H	+1.97358	+5.72339	+1.18472
H	+2.06624	+3.71511	+2.62299
C	+3.84265	+4.98298	+2.66698
H	+4.09546	+6.07282	+2.75484
O	+4.13404	+4.52260	+1.29071
C	+4.68267	+5.53455	+0.39463
O	+6.02885	+5.11746	+0.00395
C	+6.03509	+3.78146	−0.64377
C	+7.51295	+3.45338	−0.86886
O	+7.69376	+2.06133	−1.13351
H	+7.35274	+1.87223	−2.04296
H	+7.92056	+4.07313	−1.71124
H	+8.08711	+3.70629	+0.04859
H	+5.57809	+3.04586	+0.06809
H	+4.79367	+6.51670	+0.92757
C	+3.82161	+5.65751	−0.85797
O	+2.49554	+6.05945	−0.45127
H	+1.87184	+5.90735	−1.21116
H	+4.26280	+6.45541	−1.52175
C	+3.77266	+4.33823	−1.62797
O	+3.05966	+4.58221	−2.86348
H	+3.24179	+3.81310	−3.48300
H	+3.22818	+3.56079	−1.02118
C	+5.19591	+3.84698	−1.92593
H	+5.68547	+4.52315	−2.67605
O	+5.08864	+2.48428	−2.48972
C	+5.62581	+2.32917	−3.84051
O	+6.75638	+1.41066	−3.79203
H	+6.03266	+3.30868	−4.21198

C	+4.55873	+1.77073	−4.77433
O	+3.44946	+2.69470	−4.80177
H	+2.66144	+2.22555	−5.18689
H	+4.99484	+1.68691	−5.81151
C	+4.10225	+0.39060	−4.31118
O	+3.15215	−0.10827	−5.28410
H	+3.06596	−1.09890	−5.15267
H	+3.60949	+0.46680	−3.30163
C	+5.30906	−0.55055	−4.22275
H	+5.70066	−0.76786	−5.25288
C	+6.41568	+0.03227	−3.33327
H	+6.06224	+0.07814	−2.26703
C	+7.75170	−0.71194	−3.43787
H	+8.00190	−0.88538	−4.52208
H	+8.55508	−0.07496	−3.00765
O	+7.74309	−1.94485	−2.72153
H	+7.06880	−2.54249	−3.12728

345

* E = 2.795 kcal/mol

C	+0.41918	−0.83017	−0.59980
C	+0.82436	−0.74433	+0.67216
C	−0.07578	−0.65440	+1.82047
C	−1.35108	−0.25899	+1.93910
C	−2.33622	+0.27215	+0.99042
C	−2.15768	+1.32631	+0.18170
C	−0.86679	+2.05808	+0.05208
H	−1.03706	+3.13477	+0.07284
C	−3.27578	+1.85843	−0.66719
C	−4.65061	+1.42879	−0.20149
C	−4.67872	−0.05535	+0.04692
C	−3.67666	−0.40560	+1.12951
C	+1.33255	−0.96937	−1.77535
C	+2.78722	−1.24400	−1.40518
C	+3.17650	−0.42235	−0.19318
C	+2.29287	−0.86705	+0.96907
C	+4.55829	−0.69343	+0.40622
C	+4.41683	−0.16842	+1.84810
C	+2.90902	−0.15893	+2.16894
O	−5.98692	−0.39471	+0.42074
H	−6.10120	−1.34426	+0.49772

C	+3.01505	+1.06486	−0.49583
C	+5.74207	−0.12117	−0.33993
H	−4.39908	−0.57008	−0.87995
H	−3.52652	−1.48731	+1.16169
H	−4.11406	−0.12838	+2.09386
H	−5.41132	+1.69208	−0.93135
H	−4.90494	+1.93484	+0.73091
H	−3.21561	+2.94788	−0.68022
H	−3.11250	+1.54744	−1.70415
H	−0.39244	+1.83138	−0.90603
H	−0.16100	+1.79705	+0.83342
H	−1.77690	−0.40744	+2.92806
H	+0.37748	−0.99395	+2.74494
H	−0.64001	−0.78151	−0.81449
H	+1.26920	−0.05304	−2.37217
H	+0.95534	−1.75561	−2.43211
H	+2.91129	−2.30183	−1.16285
H	+3.43350	−1.03322	−2.25905
H	+4.67055	−1.78050	+0.44442
H	+4.83398	+0.83576	+1.92395
H	+4.97615	−0.78617	+2.54604
H	+2.52660	+0.85909	+2.24980
H	+2.68454	−0.65620	+3.11082
H	+3.42846	+1.68662	+0.29615
H	+1.96853	+1.33741	−0.61337
H	+3.53804	+1.31986	−1.41749
H	+6.67414	−0.40469	+0.14251
H	+5.70600	+0.96713	−0.36653
H	+5.78049	−0.48236	−1.36703
H	+2.49843	−1.93595	+1.10783
O	−6.73578	−3.72341	−2.91649
H	−6.05216	−3.95603	−3.59183
C	−6.67750	−4.68058	−1.86137
H	−6.74457	−5.72547	−2.26901
H	−7.55797	−4.51789	−1.20254
C	−5.39898	−4.53681	−1.02902
H	−5.24483	−3.47190	−0.71589
O	−5.67057	−5.35072	+0.18431
C	−4.57793	−5.34158	+1.15626
O	−4.37009	−3.96928	+1.60887

H	−4.91484	−5.98832	+2.01044
C	−3.31810	−5.89140	+0.49694
O	−2.25062	−5.85555	+1.46631
H	−1.38553	−5.97004	+0.98794
H	−3.51042	−6.96223	+0.19718
C	−2.94562	−5.08874	−0.74779
O	−1.81402	−5.74498	−1.36698
H	−1.72365	−5.39152	−2.30148
H	−2.66651	−4.03709	−0.46017
C	−4.13168	−5.05537	−1.72002
H	−4.30853	−6.07820	−2.14660
O	−3.77924	−4.12271	−2.81254
C	−3.73269	−4.71041	−4.14682
O	−4.76820	−4.08028	−4.96267
C	−4.61959	−2.60367	−5.04054
C	−5.81888	−2.14827	−5.87779
O	−6.03946	−0.74308	−5.76820
H	−5.29132	−0.26895	−6.20770
H	−5.66365	−2.44315	−6.95146
H	−6.73217	−2.66614	−5.51364
H	−4.67987	−2.18814	−4.00284
H	−3.97905	−5.80529	−4.10630
C	−2.37655	−4.45107	−4.79228
O	−1.36556	−5.09460	−3.98809
H	−0.47180	−4.76965	−4.28128
H	−2.37373	−4.90530	−5.82437
C	−2.10752	−2.95150	−4.90269
O	−0.86435	−2.78252	−5.62533
H	−0.80688	−1.82579	−5.92335
H	−2.01331	−2.50337	−3.87444
C	−3.25475	−2.26185	−5.65391
H	−3.22968	−2.54780	−6.73894
O	−3.04182	−0.80343	−5.52643
C	−2.87685	−0.08117	−6.78197
O	−3.97284	+0.87708	−6.90659
C	−4.04007	+1.82322	−5.76278
C	−5.28260	+2.67207	−6.04752
O	−5.68743	+3.41806	−4.90055
H	−5.01020	+4.11631	−4.72326
H	−5.08117	+3.35719	−6.91537

H	−6.12148	+2.00156	−6.33278
H	−4.17153	+1.23077	−4.82203
H	−2.96119	−0.78048	−7.65656
C	−1.55705	+0.68112	−6.78061
O	−0.48445	−0.27859	−6.66611
H	+0.34866	+0.20395	−6.41625
H	−1.45581	+1.23472	−7.75786
C	−1.51260	+1.68456	−5.62950
O	−0.28981	+2.44671	−5.76418
H	−0.36968	+3.26746	−5.19203
H	−1.51452	+1.13864	−4.64489
C	−2.72889	+2.61846	−5.69560
H	−2.64048	+3.29983	−6.58304
O	−2.72765	+3.42775	−4.45608
C	−2.63327	+4.87021	−4.65384
O	−3.85551	+5.48622	−4.14261
C	−4.08919	+5.20389	−2.70245
C	−5.43821	+5.86462	−2.40349
O	−5.97273	+5.43221	−1.15347
H	−5.41748	+5.80845	−0.42695
H	−5.32297	+6.98231	−2.42033
H	−6.16157	+5.58646	−3.19986
H	−4.15153	+4.09421	−2.56512
H	−2.58725	+5.11600	−5.74876
C	−1.44048	+5.43723	−3.89239
O	−0.24191	+4.81671	−4.40361
H	+0.50320	+4.99649	−3.76931
H	−1.38976	+6.54851	−4.07937
C	−1.57892	+5.19188	−2.39081
O	−0.47068	+5.85670	−1.73936
H	−0.69076	+5.94748	−0.76432
H	−1.54009	+4.08712	−2.17486
C	−2.91265	+5.76124	−1.88920
H	−2.89648	+6.88195	−1.94642
O	−3.06689	+5.34671	−0.47621
C	−3.17907	+6.44268	+0.48316
O	−4.49467	+6.37514	+1.11210
C	−4.73764	+5.09967	+1.83571
C	−6.17195	+5.23847	+2.35554
O	−6.69080	+3.99679	+2.82703

H	−6.20099	+3.73896	+3.64570
H	−6.20276	+6.01540	+3.16753
H	−6.82459	+5.58924	+1.52731
H	−4.65751	+4.25497	+1.10436
H	−3.13191	+7.43229	−0.04613
C	−2.10761	+6.32606	+1.56125
O	−0.81588	+6.38084	+0.91933
H	−0.12690	+6.06836	+1.56529
H	−2.20868	+7.20337	+2.26318
C	−2.26100	+5.03176	+2.35736
O	−1.28538	+5.06331	+3.42683
H	−1.53473	+4.35775	+4.09557
H	−2.06737	+4.14550	+1.69146
C	−3.67925	+4.93373	+2.93487
H	−3.82311	+5.70609	+3.73656
O	−3.82005	+3.58410	+3.52432
C	−4.08687	+3.54949	+4.95722
O	−5.39836	+2.93963	+5.16505
C	−5.50549	+1.57397	+4.58699
C	−6.94931	+1.16078	+4.89095
O	−7.36194	+0.03756	+4.11564
H	−6.81641	−0.74595	+4.37227
H	−7.05378	+0.94460	+5.99033
H	−7.62613	+2.00801	+4.64858
H	−5.33229	+1.64071	+3.48282
H	−4.14857	+4.59033	+5.37467
C	−3.04000	+2.70445	+5.67440
O	−1.75019	+3.32542	+5.48899
H	−1.04514	+2.66842	+5.73537
H	−3.28518	+2.68087	+6.77498
C	−3.03792	+1.27966	+5.12700
O	−2.10809	+0.49977	+5.91833
H	−2.28922	−0.47013	+5.73716
H	−2.70817	+1.28557	+4.05119
C	−4.44129	+0.66752	+5.22272
H	−4.69592	+0.45351	+6.29471
O	−4.39265	−0.60582	+4.46878
C	−4.74334	−1.80318	+5.22161
O	−5.94340	−2.38201	+4.61709
H	−5.00779	−1.54315	+6.28138

C	−3.61379	−2.82665	+5.14708
O	−2.42945	−2.22525	+5.71494
H	−1.64173	−2.77384	+5.45525
H	−3.90269	−3.72712	+5.76149
C	−3.35153	−3.27024	+3.70699
O	−2.39982	−4.35993	+3.75579
H	−2.40202	−4.81145	+2.86001
H	−2.91857	−2.41457	+3.11640
C	−4.65605	−3.73576	+3.04422
H	−5.00458	−4.69443	+3.51152
C	−5.74065	−2.65840	+3.17304
H	−5.39914	−1.71283	+2.68198
C	−7.11349	−3.05549	+2.63204
H	−7.42374	−4.05062	+3.04333
H	−7.86495	−2.30102	+2.94675
O	−7.09247	−3.09253	+1.19927
H	−6.65272	−3.93529	+0.92466
O	+6.38221	−5.01903	−1.60041
H	+5.64087	−5.60160	−1.30217
C	+6.28476	−4.84425	−3.01323
H	+6.25432	−5.83953	−3.53466
H	+7.19778	−4.31102	−3.35499
C	+5.05113	−4.02323	−3.40036
H	+4.99075	−3.08550	−2.79123
O	+5.29190	−3.64667	−4.81717
C	+4.24098	−2.78758	−5.35697
O	+4.14323	−1.59977	−4.51498
H	+4.55754	−2.50923	−6.39784
C	+2.92455	−3.55705	−5.34578
O	+1.89763	−2.69291	−5.88029
H	+1.00908	−3.07396	−5.64820
H	+3.02882	−4.46301	−6.00878
C	+2.56625	−4.00631	−3.93074
O	+1.39097	−4.84789	−4.02126
H	+1.27668	−5.30978	−3.13755
H	+2.33842	−3.10727	−3.29485
C	+3.72558	−4.78962	−3.29893
H	+3.81785	−5.79806	−3.78258
O	+3.39986	−4.96114	−1.86484
C	+3.26090	−6.34134	−1.41595

O	+4.30887	−6.61180	−0.43366
C	+4.25179	−5.70090	+0.73945
C	+5.46657	−6.10011	+1.58222
O	+5.74674	−5.13632	+2.59648
H	+5.02690	−5.17577	+3.27322
H	+5.29430	−7.11365	+2.03575
H	+6.35755	−6.17123	+0.92203
H	+4.35021	−4.64677	+0.37437
H	+3.43174	−7.05305	−2.26784
C	+1.90216	−6.55385	−0.75856
O	+0.87806	−6.27612	−1.73725
H	+0.01095	−6.16969	−1.26142
H	+1.82293	−7.63209	−0.43641
C	+1.73727	−5.65552	+0.46614
O	+0.48187	−6.00585	+1.09546
H	+0.48638	−5.63198	+2.02639
H	+1.71834	−4.57464	+0.15219
C	+2.89935	−5.88333	+1.44184
H	+2.82912	−6.91091	+1.88780
O	+2.77433	−4.87480	+2.51820
C	+2.61119	−5.41817	+3.86216
O	+3.76809	−5.02126	+4.66203
C	+3.93287	−3.54735	+4.75125
C	+5.22761	−3.36320	+5.54852
O	+5.71149	−2.02301	+5.46994
H	+5.10278	−1.43877	+5.98553
H	+5.05779	−3.66067	+6.61854
H	+6.00850	−4.03401	+5.13010
H	+4.04338	−3.13901	+3.71435
H	+2.61407	−6.54104	+3.83608
C	+1.34788	−4.87016	+4.51733
O	+0.21601	−5.26118	+3.71279
H	−0.57796	−4.73847	+4.00779
H	+1.24895	−5.32980	+5.54288
C	+1.41139	−3.35040	+4.65960
O	+0.23912	−2.93307	+5.40017
H	+0.39802	−2.00032	+5.73474
H	+1.41066	−2.86603	+3.64266
C	+2.68528	−2.94734	+5.41363
H	+2.61733	−3.27342	+6.48532

O	+2.77790	−1.47185	+5.35672
C	+2.78033	−0.79512	+6.64958
O	+4.05883	−0.10572	+6.80376
C	+4.32320	+0.86615	+5.71125
C	+5.71671	+1.41696	+6.02856
O	+6.27462	+2.11869	+4.91830
H	+5.75531	+2.94811	+4.77607
H	+5.66266	+2.08353	+6.93192
H	+6.39417	+0.57061	+6.27277
H	+4.32764	+0.31198	+4.73817
H	+2.71623	−1.54136	+7.48631
C	+1.65729	+0.23366	+6.71287
O	+0.40090	−0.46265	+6.56820
H	−0.30955	+0.20540	+6.37061
H	+1.68669	+0.73944	+7.72033
C	+1.81984	+1.28598	+5.61807
O	+0.79956	+2.29214	+5.82418
H	+1.03557	+3.08957	+5.26260
H	+1.68523	+0.81001	+4.60688
C	+3.21305	+1.92565	+5.69666
H	+3.28437	+2.57998	+6.60577
O	+3.37813	+2.75452	+4.48224
C	+3.58468	+4.17750	+4.71879
O	+4.91056	+4.53595	+4.21830
C	+5.08376	+4.22308	+2.77604
C	+6.54069	+4.59393	+2.48385
O	+6.98061	+4.06541	+1.23336
H	+6.51541	+4.54939	+0.50724
H	+6.65779	+5.71132	+2.50470
H	+7.18871	+4.17046	+3.28105
H	+4.91660	+3.12495	+2.62872
H	+3.58730	+4.40087	+5.81938
C	+2.53911	+4.99600	+3.97017
O	+1.23703	+4.63619	+4.47828
H	+0.54519	+4.97795	+3.85022
H	+2.72508	+6.09043	+4.17000
C	+2.62411	+4.74157	+2.46613
O	+1.68594	+5.63447	+1.82032
H	+1.92072	+5.68197	+0.84592
H	+2.35057	+3.67282	+2.24076

C	+4.04899	+5.01813	+1.96795
H	+4.26919	+6.11707	+2.02816
O	+4.11131	+4.58430	+0.55451
C	+4.45646	+5.62954	−0.40204
O	+5.72493	+5.27322	−1.03409
C	+5.67693	+3.96102	−1.72914
C	+7.10622	+3.75676	−2.24015
O	+7.32888	+2.41336	−2.66672
H	+6.81110	+2.25788	−3.49450
H	+7.31465	+4.47745	−3.07665
H	+7.81980	+3.97722	−1.41735
H	+5.40607	+3.17168	−0.98128
H	+4.62717	+6.60665	+0.12471
C	+3.38415	+5.74336	−1.47972
O	+2.13757	+6.08992	−0.83990
H	+1.39554	+5.92341	−1.48085
H	+3.67587	+6.56763	−2.19245
C	+3.24701	+4.43651	−2.25866
O	+2.30353	+4.66838	−3.33197
H	+2.40009	+3.92312	−3.99665
H	+2.86126	+3.62424	−1.58181
C	+4.60913	+4.01971	−2.82995
H	+4.92112	+4.73427	−3.63727
O	+4.44923	+2.66708	−3.41029
C	+4.72673	+2.56713	−4.83909
O	+5.87754	+1.68622	−5.02536
H	+5.02244	+3.56778	−5.25442
C	+3.52989	+1.97694	−5.57636
O	+2.40427	+2.85755	−5.37945
H	+1.57462	+2.38205	−5.65466
H	+3.77343	+1.92757	−6.67677
C	+3.21429	+0.56934	−5.07321
O	+2.14972	+0.03856	−5.89810
H	+2.12395	−0.95666	−5.77221
H	+2.87875	+0.61021	−3.99846
C	+4.46104	−0.31823	−5.18303
H	+4.70152	−0.51219	−6.26195
C	+5.66117	+0.32386	−4.47383
H	+5.44643	+0.41420	−3.37773
C	+6.99578	−0.39049	−4.70761

H	+7.12733	−0.59692	−5.80415
H	+7.82408	+0.27717	−4.38666
O	+7.08816	−1.59850	−3.95370
H	+6.46541	−2.26048	−4.34327

345

* E = 4.703 kcal/mol

C	−0.61625	−0.76722	−0.29568
C	−0.78390	+0.46127	−0.80577
C	+0.24612	+1.47528	−0.98132
C	+1.51389	+1.56979	−0.54447
C	+2.33593	+0.73764	+0.33517
C	+1.98576	+0.32047	+1.56140
C	+0.69402	+0.66671	+2.21209
H	+0.85949	+0.92250	+3.25921
C	+2.90846	−0.49655	+2.41265
C	+4.10750	−1.05070	+1.67369
C	+4.72305	+0.03648	+0.82009
C	+3.71991	+0.49475	−0.21240
C	−1.76579	−1.71901	−0.29615
C	−3.08118	−1.09525	+0.19771
C	−3.21828	+0.37554	−0.20133
C	−2.18341	+0.72592	−1.28365
C	−4.50948	+0.74789	−0.93709
C	−4.15554	+2.07145	−1.63207
C	−2.62119	+2.09925	−1.76394
O	+5.05563	+1.16366	+1.60628
H	+5.81354	+0.96853	+2.15729
C	−3.02271	+1.25656	+1.02526
C	−5.76516	+0.80846	−0.09723
H	+5.61180	−0.33187	+0.30770
H	+3.67352	−0.24005	−1.01852
H	+4.08923	+1.41793	−0.66339
H	+3.80680	−1.87374	+1.02433
H	+4.83558	−1.44905	+2.37865
H	+3.24173	+0.13108	+3.24350
H	+2.33809	−1.31008	+2.86741
H	+0.00345	−0.18029	+2.19799
H	+0.19971	+1.49749	+1.71850
H	+2.06876	+2.40270	−0.96588
H	−0.05843	+2.28451	−1.63334

H	+0.34790	−1.09713	+0.05980
H	−1.53400	−2.61232	+0.28045
H	−1.91913	−2.06074	−1.32592
H	−3.91412	−1.67962	−0.19579
H	−3.13037	−1.18211	+1.28303
H	−4.65071	−0.01622	−1.70650
H	−4.50399	+2.91304	−1.03405
H	−4.64941	+2.15465	−2.59672
H	−2.19297	+2.88227	−1.13783
H	−2.29328	+2.30074	−2.78178
H	−3.13566	+2.31596	+0.79875
H	−2.03244	+1.10356	+1.44886
H	−3.75262	+1.00633	+1.79367
H	−6.63408	+1.01219	−0.71811
H	−5.70471	+1.59863	+0.64952
H	−5.94405	−0.13343	+0.42167
H	−2.37800	+0.02331	−2.10463
O	−7.51508	−3.34630	−0.87004
H	−6.91182	−3.97587	−1.33661
C	−7.58231	−3.71661	+0.50615
H	−7.86251	−4.79985	+0.61185
H	−8.38282	−3.11181	+0.98387
C	−6.25870	−3.45459	+1.23138
H	−5.89007	−2.41698	+1.02262
O	−6.60994	−3.54775	+2.67175
C	−5.48004	−3.25491	+3.55051
O	−4.99135	−1.91578	+3.24111
H	−5.87403	−3.29840	+4.60134
C	−4.38790	−4.29166	+3.30962
O	−3.27936	−3.97854	+4.18043
H	−2.47767	−4.47821	+3.86966
H	−4.79213	−5.31031	+3.57566
C	−3.94327	−4.29500	+1.84800
O	−3.02035	−5.39773	+1.67177
H	−2.90764	−5.54946	+0.68637
H	−3.42436	−3.32680	+1.60675
C	−5.15169	−4.47012	+0.91735
H	−5.54826	−5.51703	+0.99647
O	−4.67386	−4.23103	−0.46375
C	−4.82889	−5.35322	−1.38268

O	−5.75671	−4.95794	−2.44022
C	−5.30427	−3.75248	−3.18191
C	−6.44323	−3.46494	−4.16455
O	−6.32510	−2.16715	−4.74585
H	−5.53702	−2.16057	−5.34313
H	−6.46170	−4.25634	−4.96233
H	−7.41142	−3.50923	−3.62113
H	−5.18745	−2.91164	−2.45161
H	−5.29674	−6.23002	−0.85945
C	−3.48890	−5.71522	−2.01369
O	−2.58113	−6.08635	−0.95313
H	−1.65437	−6.08039	−1.31363
H	−3.63933	−6.59860	−2.69845
C	−2.92492	−4.54694	−2.82044
O	−1.73365	−5.01727	−3.49541
H	−1.49015	−4.34156	−4.19636
H	−2.65861	−3.70007	−2.12819
C	−3.95386	−4.05216	−3.84640
H	−4.07998	−4.81173	−4.66280
O	−3.42463	−2.79530	−4.42394
C	−3.18721	−2.82137	−5.86299
O	−4.07955	−1.85224	−6.49557
C	−3.87465	−0.46352	−6.00670
C	−4.94959	+0.34943	−6.73426
O	−5.09986	+1.65253	−6.17260
H	−4.29361	+2.18227	−6.38908
H	−4.69640	+0.41614	−7.82692
H	−5.92416	−0.17679	−6.64382
H	−4.04520	−0.44884	−4.90023
H	−3.45756	−3.82532	−6.28811
C	−1.74401	−2.44277	−6.17646
O	−0.87721	−3.39930	−5.53108
H	+0.04918	−3.03618	−5.53093
H	−1.59273	−2.50168	−7.29296
C	−1.42756	−1.02317	−5.70804
O	−0.08674	−0.70901	−6.15361
H	+0.03345	+0.28616	−6.10415
H	−1.47947	−0.96644	−4.58490
C	−2.43316	−0.03621	−6.31548
H	−2.27781	+0.03639	−7.42468

O	−2.18032	+1.28565	−5.69954
C	−1.81300	+2.34797	−6.62996
O	−2.84963	+3.37724	−6.58564
C	−3.04557	+3.94099	−5.22510
C	−4.19935	+4.93442	−5.39114
O	−4.74381	+5.32816	−4.13216
H	−4.06886	+5.86714	−3.65056
H	−3.84519	+5.83180	−5.96800
H	−5.01081	+4.45340	−5.97856
H	−3.33717	+3.10739	−4.53619
H	−1.79400	+1.96039	−7.68392
C	−0.48237	+2.97646	−6.23019
O	+0.53414	+1.95279	−6.28223
H	+1.33474	+2.27785	−5.78926
H	−0.23015	+3.78693	−6.97292
C	−0.56126	+3.58579	−4.83222
O	+0.68638	+4.27660	−4.58318
H	+0.55848	+4.86277	−3.77854
H	−0.70232	+2.77088	−4.06787
C	−1.73262	+4.57374	−4.74383
H	−1.50194	+5.49685	−5.33907
O	−1.88447	+4.93606	−3.31702
C	−1.70812	+6.34933	−3.00520
O	−2.97262	+6.86223	−2.48162
C	−3.43533	+6.12707	−1.27585
C	−4.79166	+6.75836	−0.94853
O	−5.53312	+5.96658	−0.02094
H	−5.08385	+6.01149	+0.85893
H	−4.63891	+7.79739	−0.54869
H	−5.38838	+6.83919	−1.88246
H	−3.55571	+5.04626	−1.54428
H	−1.48673	+6.93562	−3.93725
C	−0.62817	+6.53136	−1.94469
O	+0.61465	+6.02237	−2.47343
H	+1.26230	+5.93255	−1.72264
H	−0.51567	+7.63323	−1.73111
C	−1.00075	+5.80802	−0.65148
O	+0.00844	+6.13148	+0.33381
H	−0.35009	+5.88344	+1.23701
H	−1.02105	+4.69501	−0.82588

C	−2.38209	+6.27076	−0.16849
H	−2.32906	+7.33520	+0.18345
O	−2.76373	+5.39952	+0.96590
C	−3.00001	+6.08885	+2.22888
O	−4.39912	+5.89248	+2.60367
C	−4.76174	+4.45850	+2.74084
C	−6.26393	+4.47690	+3.03761
O	−6.84578	+3.18117	+2.89646
H	−6.52103	+2.61082	+3.63591
H	−6.44033	+4.87920	+4.07169
H	−6.76717	+5.15820	+2.31837
H	−4.55606	+3.94576	+1.76619
H	−2.85567	+7.19604	+2.10768
C	−2.11062	+5.51666	+3.32743
O	−0.73564	+5.71000	+2.93451
H	−0.15121	+5.16318	+3.52613
H	−2.30667	+6.08584	+4.28149
C	−2.40531	+4.03575	+3.56271
O	−1.61935	+3.61370	+4.70295
H	−1.98355	+2.73527	+5.02226
H	−2.11279	+3.43644	+2.65599
C	−3.90039	+3.83226	+3.84595
H	−4.16629	+4.27072	+4.84430
O	−4.14945	+2.37317	+3.87143
C	−4.66696	+1.84706	+5.12863
O	−6.00138	+1.30199	+4.88590
H	−4.79230	+2.67189	+5.88052
C	−3.76774	+0.73185	+5.65085
O	−2.45569	+1.28689	+5.88041
H	−1.80774	+0.54049	+5.99288
H	−4.18795	+0.35810	+6.62881
C	−3.70142	−0.43059	+4.66130
O	−2.93340	−1.48946	+5.28218
H	−3.10309	−2.33623	+4.77146
H	−3.19505	−0.10016	+3.71141
C	−5.11670	−0.92655	+4.33573
H	−5.56581	−1.42487	+5.23556
C	−6.01172	+0.22629	+3.86074
H	−5.60968	+0.64537	+2.90244
C	−7.49275	−0.13556	+3.71472

H	−7.84313	−0.67432	+4.63639
H	−8.08612	+0.79947	+3.62261
O	−7.73202	−0.92434	+2.54981
H	−7.34207	−1.82154	+2.69376
O	+5.19464	−6.28576	+1.48007
H	+4.38127	−6.50143	+1.99945
C	+5.04098	−6.80532	+0.15971
H	+4.77587	−7.89677	+0.19205
H	+6.01790	−6.70935	−0.36110
C	+3.97627	−6.03976	−0.63155
H	+4.14799	−4.93554	−0.56188
O	+4.19606	−6.46027	−2.03941
C	+3.30894	−5.77806	−2.97985
O	+3.52405	−4.34134	−2.85195
H	+3.59912	−6.13185	−4.00542
C	+1.86659	−6.13594	−2.63941
O	+1.00053	−5.46306	−3.57725
H	+0.06685	−5.51056	−3.23663
H	+1.73672	−7.25092	−2.74996
C	+1.52698	−5.73381	−1.20537
O	+0.18970	−6.21148	−0.92430
H	+0.05455	−6.18821	+0.06949
H	+1.56027	−4.61340	−1.10286
C	+2.53071	−6.36385	−0.23029
H	+2.37729	−7.47471	−0.18566
O	+2.26939	−5.77989	+1.10414
C	+1.90065	−6.73462	+2.14272
O	+2.93020	−6.70457	+3.17966
C	+3.11323	−5.35277	+3.76809
C	+4.27263	−5.52516	+4.75378
O	+4.80074	−4.26732	+5.17362
H	+4.13153	−3.82278	+5.75031
H	+3.93031	−6.12613	+5.63921
H	+5.08995	−6.09017	+4.25623
H	+3.39318	−4.64521	+2.94675
H	+1.88994	−7.78043	+1.73368
C	+0.56400	−6.35147	+2.76763
O	−0.44180	−6.38957	+1.73321
H	−1.26597	−5.94649	+2.07208
H	+0.30641	−7.10886	+3.56256

C	+0.63564	−4.96421	+3.40400
O	−0.62362	−4.72936	+4.07758
H	−0.50127	−3.95565	+4.70492
H	+0.79276	−4.18438	+2.60752
C	+1.79336	−4.90370	+4.40995
H	+1.55814	−5.53933	+5.30445
O	+1.93004	−3.49250	+4.83517
C	+1.75597	−3.25074	+6.26324
O	+3.01412	−2.73421	+6.79796
C	+3.45720	−1.48804	+6.12115
C	+4.81410	−1.17427	+6.75770
O	+5.53481	−0.19878	+6.00446
H	+5.08395	+0.67537	+6.11439
H	+4.66586	−0.82689	+7.81549
H	+5.42375	−2.10288	+6.78774
H	+3.57344	−1.70254	+5.02800
H	+1.55015	−4.21271	+6.80486
C	+0.65997	−2.21717	+6.49818
O	−0.57205	−2.74289	+5.96021
H	−1.23117	−2.00123	+5.88616
H	+0.54244	−2.06106	+7.60899
C	+1.01457	−0.88453	+5.84066
O	−0.00498	+0.07277	+6.21226
H	+0.34337	+0.99162	+6.00723
H	+1.03480	−1.00369	+4.72075
C	+2.38979	−0.40384	+6.32382
H	+2.33555	−0.10605	+7.40446
O	+2.74829	+0.77795	+5.50799
C	+2.97358	+2.01145	+6.25643
O	+4.37191	+2.39938	+6.08992
C	+4.73829	+2.61197	+4.66496
C	+6.24389	+2.88554	+4.69131
O	+6.81868	+2.75049	+3.38942
H	+6.49864	+3.50634	+2.83614
H	+6.44070	+3.91093	+5.10416
H	+6.73819	+2.14963	+5.36170
H	+4.51958	+1.67040	+4.09864
H	+2.81986	+1.83647	+7.35534
C	+2.07837	+3.12856	+5.72968
O	+0.70681	+2.69756	+5.87131

H	+0.12586	+3.29618	+5.32921
H	+2.24371	+4.04830	+6.36133
C	+2.39344	+3.46124	+4.27182
O	+1.61006	+4.62469	+3.90958
H	+1.98399	+4.99128	+3.05284
H	+2.11323	+2.59499	+3.61201
C	+3.88937	+3.75812	+4.10038
H	+4.15439	+4.72784	+4.60049
O	+4.14499	+3.86318	+2.65106
C	+4.67486	+5.13749	+2.19176
O	+6.01622	+4.91310	+1.65539
C	+6.04196	+3.93996	+0.52644
C	+7.52681	+3.84585	+0.16279
O	+7.80215	+2.71256	−0.66190
H	+7.38434	+2.86281	−1.54541
H	+7.85584	+4.79250	−0.34684
H	+8.12345	+3.74129	+1.09504
H	+5.65038	+2.95036	+0.89097
H	+4.79136	+5.85263	+3.05102
C	+3.78760	+5.70790	+1.08997
O	+2.46650	+5.91509	+1.63816
H	+1.82692	+6.01128	+0.88341
H	+4.21018	+6.69982	+0.75815
C	+3.73163	+4.75870	−0.10452
O	+2.96325	+5.40577	−1.14981
H	+3.13912	+4.91600	−2.00685
H	+3.23045	+3.79800	+0.19574
C	+5.14811	+4.45593	−0.60762
H	+5.59353	+5.37017	−1.08380
O	+5.02442	+3.38684	−1.62729
C	+5.50692	+3.73001	−2.95744
O	+6.64447	+2.86562	−3.27373
C	+6.30233	+1.42199	−3.20925
C	+7.62920	+0.70633	−3.47811
O	+7.55795	−0.67999	−3.14397
H	+6.97298	−1.13044	−3.80189
H	+7.91958	+0.83885	−4.55520
H	+8.42353	+1.16825	−2.85338
H	+5.93028	+1.19240	−2.17785
H	+5.89232	+4.78456	−2.98068

C	+4.41314	+3.49775	−3.99377
O	+3.29764	+4.35333	−3.66766
H	+2.50904	+4.05686	−4.19692
H	+4.81188	+3.77948	−5.01065
C	+3.98042	+2.03226	−4.01573
O	+3.03944	+1.87235	−5.10414
H	+2.94899	+0.89133	−5.29377
H	+3.48489	+1.76382	−3.04061
C	+5.19946	+1.12594	−4.23447
H	+5.59228	+1.25651	−5.27763
O	+4.74641	−0.27231	−4.05504
C	+4.91583	−1.13511	−5.21849
O	+5.85274	−2.20120	−4.86769
H	+5.38416	−0.56732	−6.06670
C	+3.58525	−1.76245	−5.61903
O	+2.67021	−0.70029	−5.96009
H	+1.74708	−1.07076	−5.97757
H	+3.75278	−2.41236	−6.52587
C	+3.02026	−2.62146	−4.48929
O	+1.82924	−3.27212	−4.99217
H	+1.59899	−4.02376	−4.36904
H	+2.75708	−1.97334	−3.60755
C	+4.05443	−3.67073	−4.06012
H	+4.19204	−4.42998	−4.87505
C	+5.39702	−3.01435	−3.71104
H	+5.26726	−2.33496	−2.83033
C	+6.54363	−4.00100	−3.47174
H	+6.57902	−4.74893	−4.30934
H	+7.50590	−3.44524	−3.47283
O	+6.41921	−4.66174	−2.21279
H	+5.64917	−5.28032	−2.25818

345

* E = 4.917 kcal/mol

C	−0.46366	−1.10918	−0.00012
C	−0.55410	−0.09342	−0.86731
C	+0.58834	+0.57169	−1.49320
C	+1.89996	+0.55158	−1.21849
C	+2.67541	−0.09288	−0.15960
C	+2.52634	+0.07966	+1.15985
C	+1.45269	+0.88538	+1.79756

H	+1.86655	+1.54797	+2.55895
C	+3.55463	−0.57330	+2.03378
C	+4.96412	−0.23432	+1.55036
C	+5.14202	−0.45985	+0.04500
C	+3.84696	−0.92950	−0.59588
C	−1.62958	−1.81858	+0.60977
C	−2.97665	−1.44336	+0.00210
C	−3.01195	+0.03992	−0.29931
C	−1.92194	+0.33085	−1.32686
C	−4.23943	+0.55108	−1.05531
C	−3.73726	+1.86858	−1.67518
C	−2.19984	+1.77359	−1.72931
O	+5.49169	+0.73382	−0.62987
H	+6.39458	+0.98005	−0.42427
C	−2.80930	+0.84057	+0.98305
C	−5.51277	+0.69700	−0.25325
H	+5.90847	−1.21188	−0.14069
H	+3.67562	−1.96937	−0.30572
H	+3.96566	−0.91849	−1.67736
H	+5.69563	−0.80579	+2.11714
H	+5.15122	+0.81984	+1.74764
H	+3.43247	−0.26436	+3.07087
H	+3.42069	−1.65945	+2.02053
H	+0.73031	+0.23579	+2.29974
H	+0.90553	+1.47783	+1.07039
H	+2.53166	+1.08100	−1.92569
H	+0.31740	+1.16600	−2.35743
H	+0.51524	−1.44847	+0.30729
H	−1.63552	−1.60634	+1.68427
H	−1.47154	−2.89663	+0.54018
H	−3.12535	−1.99110	−0.93103
H	−3.78402	−1.73611	+0.67517
H	−4.42303	−0.16214	−1.86337
H	−4.05090	+2.71423	−1.06223
H	−4.16875	+2.02658	−2.66074
H	−1.73491	+2.46200	−1.02306
H	−1.80776	+2.01911	−2.71443
H	−2.97434	+1.90549	+0.82780
H	−1.80224	+0.71854	+1.37734
H	−3.51600	+0.50920	+1.74316

H	−6.33513	+1.01298	−0.89016
H	−5.40237	+1.44430	+0.53176
H	−5.80090	−0.24327	+0.21532
H	−2.16564	−0.28294	−2.20298
O	−6.47657	−3.68390	−3.45307
H	−5.70827	−3.88232	−4.04317
C	−6.55106	−4.69442	−2.44835
H	−6.58073	−5.71624	−2.91543
H	−7.50005	−4.55038	−1.88857
C	−5.37544	−4.61057	−1.47034
H	−5.24927	−3.56545	−1.08856
O	−5.78152	−5.47872	−0.33481
C	−4.81176	−5.47033	+0.75811
O	−4.64758	−4.09477	+1.21564
H	−5.24557	−6.10820	+1.57448
C	−3.48742	−6.02551	+0.24490
O	−2.53729	−5.99384	+1.33243
H	−1.62425	−6.11391	+0.95701
H	−3.64588	−7.09465	−0.07705
C	−2.97034	−5.21644	−0.94329
O	−1.79805	−5.89455	−1.45620
H	−1.58932	−5.50736	−2.35843
H	−2.69051	−4.18034	−0.60564
C	−4.04270	−5.11795	−2.03689
H	−4.18239	−6.11596	−2.53073
O	−3.55560	−4.14029	−3.03631
C	−3.36486	−4.66663	−4.38300
O	−4.29022	−3.97780	−5.28042
C	−4.09273	−2.50477	−5.30023
C	−5.19804	−1.99347	−6.22882
O	−5.35018	−0.57739	−6.14254
H	−4.55146	−0.15142	−6.54019
H	−4.97558	−2.30417	−7.28560
H	−6.16275	−2.45996	−5.93463
H	−4.23430	−2.11458	−4.26029
H	−3.63572	−5.75598	−4.42266
C	−1.93866	−4.41696	−4.85997
O	−1.03491	−5.09542	−3.96217
H	−0.11726	−4.74286	−4.11653
H	−1.82582	−4.85069	−5.89534

C	−1.62747	−2.92228	−4.91112
O	−0.30789	−2.77368	−5.48656
H	−0.19567	−1.81546	−5.76132
H	−1.64170	−2.48909	−3.87225
C	−2.66666	−2.20045	−5.77914
H	−2.54488	−2.50190	−6.85340
O	−2.41589	−0.74751	−5.64830
C	−2.07971	−0.05994	−6.89009
O	−3.13144	+0.91413	−7.17454
C	−3.30355	+1.91295	−6.08825
C	−4.48488	+2.77019	−6.55275
O	−5.00338	+3.57611	−5.49541
H	−4.34033	+4.27730	−5.27978
H	−4.16879	+3.41063	−7.42034
H	−5.30025	+2.10208	−6.90449
H	−3.55584	+1.36879	−5.14246
H	−2.07347	−0.78121	−7.75105
C	−0.75145	+0.67703	−6.75741
O	+0.27768	−0.29447	−6.47736
H	+1.10212	+0.18695	−6.19552
H	−0.52406	+1.18408	−7.73913
C	−0.81300	+1.73335	−5.65537
O	+0.43664	+2.46336	−5.68311
H	+0.31467	+3.30953	−5.15761
H	−0.94107	+1.23712	−4.65216
C	−1.99036	+2.68594	−5.90426
H	−1.78391	+3.32589	−6.80279
O	−2.11501	+3.54764	−4.70795
C	−1.96902	+4.97893	−4.94853
O	−3.22893	+5.63322	−4.60281
C	−3.64080	+5.37341	−3.19909
C	−4.99775	+6.07111	−3.06770
O	−5.69868	+5.64750	−1.89876
H	−5.21551	+5.98179	−1.10322
H	−4.85132	+7.18553	−3.05976
H	−5.62299	+5.81831	−3.95086
H	−3.74930	+4.26745	−3.06246
H	−1.79167	+5.18116	−6.03884
C	−0.86179	+5.55299	−4.07198
O	+0.37470	+4.90022	−4.43193

H	+1.04833	+5.07395	−3.72027
H	−0.77187	+6.65794	−4.27881
C	−1.17433	+5.34325	−2.59150
O	−0.15767	+6.03601	−1.82807
H	−0.47514	+6.09754	−0.87838
H	−1.14930	+4.24510	−2.34771
C	−2.56043	+5.90760	−2.24921
H	−2.53526	+7.02933	−2.27712
O	−2.88418	+5.45955	−0.87530
C	−3.09829	+6.52584	+0.09506
O	−4.48097	+6.44994	+0.56300
C	−4.80960	+5.14432	+1.19197
C	−6.30218	+5.25342	+1.51701
O	−6.86353	+3.98720	+1.86013
H	−6.51058	+3.71843	+2.74370
H	−6.45370	+5.99213	+2.34983
H	−6.83984	+5.63561	+0.62281
H	−4.62321	+4.33187	+0.44257
H	−2.98232	+7.53170	−0.39107
C	−2.16338	+6.35964	+1.28795
O	−0.80518	+6.42375	+0.80525
H	−0.19679	+6.09391	+1.52042
H	−2.34147	+7.21166	+2.00585
C	−2.42184	+5.03916	+2.01250
O	−1.58217	+5.01876	+3.19132
H	−1.93658	+4.31728	+3.81489
H	−2.16002	+4.17248	+1.34255
C	−3.90020	+4.94413	+2.41235
H	−4.13435	+5.70797	+3.20070
O	−4.12043	+3.58898	+2.96575
C	−4.59629	+3.54935	+4.34445
O	−5.92821	+2.94906	+4.35524
C	−5.94966	+1.57942	+3.78021
C	−7.42838	+1.18191	+3.82126
O	−7.69018	+0.02798	+3.02405
H	−7.26702	−0.75466	+3.45544
H	−7.74234	+1.00712	+4.88615
H	−8.03964	+2.01932	+3.42137
H	−5.58092	+1.63252	+2.72341
H	−4.71311	+4.58971	+4.75125

C	−3.66915	+2.70271	+5.21031
O	−2.35768	+3.30546	+5.18246
H	−1.69719	+2.64685	+5.52735
H	−4.05847	+2.71149	+6.26887
C	−3.61111	+1.26039	+4.71085
O	−2.82840	+0.49740	+5.66156
H	−2.97891	−0.47619	+5.47186
H	−3.11657	+1.22692	+3.70116
C	−5.02537	+0.67301	+4.60432
H	−5.45122	+0.50870	+5.62964
O	−4.90442	−0.62937	+3.91025
C	−5.34738	−1.78918	+4.67497
O	−6.49600	−2.38055	+3.99150
H	−5.70771	−1.47798	+5.69218
C	−4.23539	−2.82911	+4.75757
O	−3.10798	−2.22531	+5.42762
H	−2.30690	−2.79506	+5.27581
H	−4.60325	−3.70440	+5.36639
C	−3.83885	−3.31813	+3.36566
O	−2.87681	−4.38756	+3.53209
H	−2.80777	−4.87956	+2.66021
H	−3.37171	−2.47416	+2.78511
C	−5.07136	−3.83275	+2.60970
H	−5.43530	−4.78822	+3.07249
C	−6.19253	−2.78477	+2.59426
H	−5.85416	−1.87887	+2.02777
C	−7.53165	−3.29484	+2.05324
H	−7.78493	−4.27685	+2.53740
H	−8.32990	−2.56955	+2.32065
O	−7.51408	−3.42404	+0.63244
H	−6.91561	−4.17370	+0.39193
O	+6.38924	−5.05058	−0.68982
H	+5.63029	−5.65028	−0.48417
C	+6.46025	−4.88241	−2.10576
H	+6.47452	−5.87997	−2.62202
H	+7.41511	−4.36513	−2.34138
C	+5.29358	−4.04382	−2.63574
H	+5.18425	−3.09820	−2.04483
O	+5.69894	−3.68906	−4.01990
C	+4.73610	−2.81263	−4.68820

O	+4.59521	−1.59764	−3.89747
H	+5.16752	−2.58012	−5.69869
C	+3.39986	−3.53956	−4.79219
O	+2.45953	−2.66264	−5.44723
H	+1.54213	−3.01498	−5.29302
H	+3.54185	−4.46625	−5.42005
C	+2.88673	−3.94773	−3.41223
O	+1.68711	−4.73406	−3.60779
H	+1.49243	−5.21975	−2.75228
H	+2.64751	−3.03168	−2.80371
C	+3.94869	−4.78105	−2.68293
H	+4.06765	−5.77724	−3.18589
O	+3.46947	−4.98784	−1.29737
C	+3.27548	−6.37930	−0.90678
O	+4.20550	−6.68480	+0.17932
C	+4.02281	−5.80122	+1.35940
C	+5.12898	−6.23227	+2.32704
O	+5.31350	−5.28248	+3.37631
H	+4.52442	−5.31482	+3.97147
H	+4.88909	−7.24586	+2.74826
H	+6.08632	−6.31532	+1.76891
H	+4.17240	−4.74045	+1.03317
H	+3.53835	−7.06825	−1.75377
C	+1.85155	−6.60417	−0.41011
O	+0.94344	−6.30277	−1.48998
H	+0.02197	−6.23820	−1.11989
H	+1.73832	−7.68913	−0.12289
C	+1.55114	−5.73238	+0.80787
O	+0.23378	−6.09298	+1.28773
H	+0.13275	−5.73191	+2.21836
H	+1.56534	−4.64560	+0.51526
C	+2.59831	−5.97863	+1.90211
H	+2.46976	−7.00716	+2.33233
O	+2.36742	−4.97257	+2.96244
C	+2.05424	−5.51434	+4.27949
O	+3.11589	−5.11726	+5.20214
C	+3.28656	−3.64361	+5.28247
C	+4.47816	−3.45467	+6.22587
O	+4.98859	−2.12299	+6.17333
H	+4.32523	−1.51590	+6.58486

H	+4.17586	−3.72178	+7.27456
H	+5.29367	−4.14569	+5.92202
H	+3.52604	−3.25893	+4.25827
H	+2.05473	−6.63708	+4.25644
C	+0.72990	−4.95306	+4.78458
O	−0.30911	−5.35671	+3.86780
H	−1.12297	−4.81480	+4.05226
H	+0.51657	−5.39043	+5.80206
C	+0.79081	−3.43091	+4.89758
O	−0.45135	−2.98750	+5.49362
H	−0.32553	−2.04252	+5.80663
H	+0.90666	−2.97469	+3.87505
C	+1.97715	−3.01447	+5.77750
H	+1.78393	−3.30406	+6.84445
O	+2.09409	−1.54240	+5.68352
C	+1.95095	−0.82316	+6.94401
O	+3.21292	−0.14364	+7.22911
C	+3.61685	+0.79567	+6.15113
C	+4.97957	+1.32604	+6.60652
O	+5.66947	+1.98948	+5.54799
H	+5.19801	+2.83594	+5.34891
H	+4.84317	+2.01517	+7.48345
H	+5.60690	+0.47203	+6.94129
H	+3.71441	+0.21789	+5.19578
H	+1.77599	−1.53974	+7.79084
C	+0.84352	+0.21943	+6.84001
O	−0.39476	−0.46682	+6.55866
H	−1.07342	+0.20415	+6.27658
H	+0.75491	+0.75181	+7.83032
C	+1.15576	+1.24233	+5.74908
O	+0.12708	+2.25934	+5.79779
H	+0.45316	+3.05630	+5.28193
H	+1.14915	+0.74194	+4.74020
C	+2.53375	+1.87159	+5.99464
H	+2.49973	+2.52621	+6.90563
O	+2.85339	+2.69826	+4.80962
C	+3.06876	+4.11592	+5.07939
O	+4.45142	+4.44330	+4.73958
C	+4.78179	+4.15080	+3.32048
C	+6.27236	+4.48284	+3.20697

O	+6.83691	+3.97077	+1.99977
H	+6.46870	+4.48744	+1.24046
H	+6.41825	+5.59494	+3.27092
H	+6.81322	+4.02064	+4.06069
H	+4.60265	+3.06144	+3.13226
H	+2.95088	+4.33117	+6.17538
C	+2.13410	+4.96783	+4.22793
O	+0.77560	+4.61961	+4.57120
H	+0.16943	+4.96866	+3.86372
H	+2.31135	+6.05417	+4.47435
C	+2.39077	+4.74503	+2.73877
O	+1.56075	+5.67871	+2.00792
H	+1.89882	+5.71938	+1.06364
H	+2.11708	+3.69007	+2.45629
C	+3.87067	+4.98922	+2.41387
H	+4.11301	+6.08027	+2.51815
O	+4.08230	+4.57148	+1.01170
C	+4.54869	+5.61672	+0.10930
O	+5.88019	+5.24755	−0.36727
C	+5.89239	+3.93140	−1.05981
C	+7.36998	+3.68992	−1.37933
O	+7.62122	+2.31958	−1.70364
H	+7.20457	+2.13368	−2.58298
H	+7.69484	+4.35760	−2.22152
H	+7.98245	+3.94693	−0.48820
H	+5.50732	+3.15400	−0.35273
H	+4.66415	+6.59038	+0.65763
C	+3.61307	+5.74455	−1.08816
O	+2.30440	+6.10053	−0.59343
H	+1.64181	+5.97275	−1.32494
H	+3.99334	+6.57064	−1.75565
C	+3.55866	+4.44281	−1.88625
O	+2.76336	+4.68606	−3.07269
H	+2.94486	+3.94284	−3.72208
H	+3.08194	+3.63197	−1.26934
C	+4.97498	+4.00956	−2.28719
H	+5.39590	+4.72095	−3.04741
O	+4.85800	+2.66459	−2.88441
C	+5.34708	+2.53464	−4.24782
O	+6.48890	+1.62141	−4.24354

H	+5.72845	+3.52031	−4.62982
C	+4.25349	+1.96087	−5.14328
O	+3.13146	+2.87169	−5.11103
H	+2.32979	+2.39774	−5.45783
H	+4.64438	+1.89179	−6.19890
C	+3.83105	+0.57094	−4.67116
O	+2.89410	+0.04270	−5.64177
H	+2.78985	−0.93904	−5.46421
H	+3.33208	+0.64629	−3.66547
C	+5.05150	−0.35193	−4.55838
H	+5.44518	−0.59900	−5.58044
C	+6.15012	+0.27914	−3.69367
H	+5.78258	+0.40716	−2.63706
C	+7.48173	−0.47664	−3.72322
H	+7.76415	−0.71120	−4.78534
H	+8.27860	+0.17157	−3.29824
O	+7.42859	−1.67343	−2.94455
H	+6.83486	−2.31561	−3.40601

345

* E = 5.180 kcal/mol

C	+0.60930	+1.21151	+0.61426
C	+0.23635	−0.04746	+0.87099
C	+1.08703	−1.05933	+1.49874
C	+2.41930	−1.17216	+1.60944
C	+3.51908	−0.35958	+1.09761
C	+3.71396	+0.02419	−0.17150
C	+2.80612	−0.26995	−1.30911
H	+3.37392	−0.66110	−2.15497
C	+4.98486	+0.76575	−0.44958
C	+6.19187	−0.00154	+0.08430
C	+5.96879	−0.54000	+1.49477
C	+4.63605	−0.05406	+2.05692
C	−0.26867	+2.25466	−0.00234
C	−1.72981	+1.83959	−0.14702
C	−1.82303	+0.37663	−0.52865
C	−1.19105	−0.43549	+0.59855
C	−3.22549	−0.23538	−0.54706
C	−2.94568	−1.74327	−0.40043
C	−1.55050	−1.87207	+0.24234
O	+5.97730	−1.94940	+1.40701

H	+6.13241	−2.33658	+2.27292
C	−1.12440	+0.13553	−1.86230
C	−4.09824	+0.11317	−1.73205
H	+6.77259	−0.21090	+2.15359
H	+4.70057	+1.02369	+2.23208
H	+4.45378	−0.51122	+3.02902
H	+7.07404	+0.63252	+0.04937
H	+6.38653	−0.86039	−0.55336
H	+5.10157	+0.94918	−1.51623
H	+4.94448	+1.75005	+0.02692
H	+2.30886	+0.64147	−1.65260
H	+2.03341	−0.98461	−1.04254
H	+2.76015	−2.00500	+2.21860
H	+0.52472	−1.85409	+1.97560
H	+1.62299	+1.51240	+0.84010
H	+0.14333	+2.50903	−0.98459
H	−0.19741	+3.17690	+0.57733
H	−2.24863	+1.98153	+0.80348
H	−2.22686	+2.47710	−0.88043
H	−3.72675	+0.11168	+0.36055
H	−2.96629	−2.22500	−1.37864
H	−3.71507	−2.22966	+0.19535
H	−0.82603	−2.28527	−0.45921
H	−1.55565	−2.52669	+1.11135
H	−1.28775	−0.87702	−2.22791
H	−0.05123	+0.28671	−1.78262
H	−1.50866	+0.82089	−2.61732
H	−5.08744	−0.32536	−1.62552
H	−3.67217	−0.26310	−2.66161
H	−4.22479	+1.19056	−1.83283
H	−1.76679	−0.19150	+1.50009
O	−6.36537	−4.93991	−2.24231
H	−5.63393	−5.11019	−2.88551
C	−6.17850	−5.78344	−1.10669
H	−6.08691	−6.85835	−1.42223
H	−7.08052	−5.69523	−0.46376
C	−4.94456	−5.37915	−0.29445
H	−4.94910	−4.27871	−0.08545
O	−5.10342	−6.10154	+0.99414
C	−4.04377	−5.78965	+1.95068

O	−4.04237	−4.34863	+2.17851
H	−4.29586	−6.33926	+2.89710
C	−2.70931	−6.23983	+1.36685
O	−1.67124	−5.93020	+2.32167
H	−0.79157	−5.98444	+1.86104
H	−2.74398	−7.35574	+1.20714
C	−2.43530	−5.55057	+0.03179
O	−1.22026	−6.12154	−0.51086
H	−1.16164	−5.86087	−1.47825
H	−2.29637	−4.44602	+0.19410
C	−3.60693	−5.77600	−0.93381
H	−3.63334	−6.84818	−1.26438
O	−3.37694	−4.90632	−2.10924
C	−3.24140	−5.60126	−3.38362
O	−4.34009	−5.18383	−4.25265
C	−4.36872	−3.71595	−4.48288
C	−5.62206	−3.49403	−5.33461
O	−5.98756	−2.11574	−5.38976
H	−5.31142	−1.63259	−5.92552
H	−5.45297	−3.90067	−6.36843
H	−6.46921	−4.05476	−4.88436
H	−4.46575	−3.20403	−3.49165
H	−3.34970	−6.71024	−3.24339
C	−1.92011	−5.23546	−4.04996
O	−0.84627	−5.65333	−3.18142
H	−0.00835	−5.20391	−3.47485
H	−1.84294	−5.79218	−5.02819
C	−1.84414	−3.73449	−4.32479
O	−0.61953	−3.48732	−5.05471
H	−0.69160	−2.58707	−5.49188
H	−1.83156	−3.16381	−3.35317
C	−3.05570	−3.29503	−5.15709
H	−2.99059	−3.73119	−6.18921
O	−3.01293	−1.81863	−5.24750
C	−2.93559	−1.27932	−6.60075
O	−4.13872	−0.48830	−6.84876
C	−4.31369	+0.61686	−5.87136
C	−5.65008	+1.25436	−6.26290
O	−6.13442	+2.13508	−5.25011
H	−5.54199	+2.92566	−5.21106

H	−5.53631	+1.79846	−7.23967
H	−6.40529	+0.45229	−6.40814
H	−4.36914	+0.17489	−4.84358
H	−2.93767	−2.10894	−7.35770
C	−1.71639	−0.37573	−6.74766
O	−0.53510	−1.16811	−6.50198
H	+0.23361	−0.55386	−6.35703
H	−1.68477	+0.01815	−7.80407
C	−1.78793	+0.80127	−5.77704
O	−0.66532	+1.66988	−6.06225
H	−0.83300	+2.55013	−5.61097
H	−1.72018	+0.42822	−4.71695
C	−3.10790	+1.56217	−5.96195
H	−3.10674	+2.10149	−6.94618
O	−3.20001	+2.54944	−4.86290
C	−3.26389	+3.94476	−5.28072
O	−4.55318	+4.49108	−4.86222
C	−4.77280	+4.39771	−3.39553
C	−6.18865	+4.94649	−3.19509
O	−6.69165	+4.65410	−1.89241
H	−6.18689	+5.19026	−1.23266
H	−6.19314	+6.05462	−3.38200
H	−6.86766	+4.47502	−3.93768
H	−4.71749	+3.31956	−3.09655
H	−3.23254	+4.02438	−6.40053
C	−2.15116	+4.75067	−4.62002
O	−0.88523	+4.20060	−5.04162
H	−0.17167	+4.55247	−4.44466
H	−2.22447	+5.82067	−4.96952
C	−2.27818	+4.71393	−3.09814
O	−1.26349	+5.59176	−2.55499
H	−1.49928	+5.78649	−1.59910
H	−2.11378	+3.66436	−2.72299
C	−3.67398	+5.19127	−2.67528
H	−3.78415	+6.28839	−2.88460
O	−3.79384	+4.95709	−1.21878
C	−4.03156	+6.14973	−0.41269
O	−5.33851	+6.01970	+0.22748
C	−5.44676	+4.80478	+1.07614
C	−6.89286	+4.82653	+1.58043

O	−7.27566	+3.57395	+2.14640
H	−6.77234	+3.44038	+2.98694
H	−7.01876	+5.65505	+2.32945
H	−7.57139	+5.03683	+0.72593
H	−5.26406	+3.90714	+0.43263
H	−4.08513	+7.06278	−1.06442
C	−2.96626	+6.28306	+0.66971
O	−1.68132	+6.41090	+0.02382
H	−0.97048	+6.24579	+0.69952
H	−3.17441	+7.21645	+1.26761
C	−2.98167	+5.07575	+1.60507
O	−2.03603	+5.33681	+2.67001
H	−2.21552	+4.68323	+3.41029
H	−2.67311	+4.15149	+1.04158
C	−4.38768	+4.87045	+2.18498
H	−4.62925	+5.69408	+2.90808
O	−4.37771	+3.57612	+2.90268
C	−4.67997	+3.64909	+4.32744
O	−5.91229	+2.90314	+4.57381
C	−5.82055	+1.48198	+4.14934
C	−7.21909	+0.91838	+4.41793
O	−7.41345	−0.34250	+3.77870
H	−6.84577	−1.01471	+4.22986
H	−7.38439	+0.83053	+5.52605
H	−7.97721	+1.62520	+4.01739
H	−5.58498	+1.45458	+3.05522
H	−4.88247	+4.71008	+4.63501
C	−3.55508	+3.02251	+5.14346
O	−2.34541	+3.76850	+4.89205
H	−1.57210	+3.23588	+5.22157
H	−3.81718	+3.10052	+6.23785
C	−3.36842	+1.55055	+4.77954
O	−2.37268	+1.00404	+5.67674
H	−2.43514	+0.00342	+5.63779
H	−3.01282	+1.45964	+3.71516
C	−4.69627	+0.79801	+4.93932
H	−4.96960	+0.73000	+6.02577
O	−4.49596	−0.56554	+4.39979
C	−4.71080	−1.64961	+5.35177
O	−5.83133	−2.46026	+4.88082

H	−5.01247	−1.24141	+6.35364
C	−3.47147	−2.53228	+5.44629
O	−2.37426	−1.71961	+5.91308
H	−1.52517	−2.21680	+5.76497
H	−3.66958	−3.35192	+6.19551
C	−3.14254	−3.16292	+4.09441
O	−2.03510	−4.07278	+4.29722
H	−1.97533	−4.67408	+3.49579
H	−2.84850	−2.36445	+3.35701
C	−4.36218	−3.92573	+3.56009
H	−4.55294	−4.83406	+4.19112
C	−5.60748	−3.02901	+3.52642
H	−5.44547	−2.18407	+2.80997
C	−6.91263	−3.76667	+3.21284
H	−6.99751	−4.67621	+3.86734
H	−7.77020	−3.09986	+3.44684
O	−7.00043	−4.12830	+1.83552
H	−6.33342	−4.83479	+1.65258
O	+6.88848	−4.31737	−0.91908
H	+6.15097	−4.81080	−0.48466
C	+6.70507	−4.36741	−2.33102
H	+6.72112	−5.43181	−2.69789
H	+7.55968	−3.83732	−2.80455
C	+5.39797	−3.69863	−2.76940
H	+5.27554	−2.69817	−2.28251
O	+5.57859	−3.49361	−4.23187
C	+4.43894	−2.84758	−4.87552
O	+4.22826	−1.54791	−4.24527
H	+4.71460	−2.72065	−5.95696
C	+3.20722	−3.72640	−4.69844
O	+2.09312	−3.08162	−5.35149
H	+1.25203	−3.50758	−5.03349
H	+3.39895	−4.72237	−5.19226
C	+2.91345	−3.95105	−3.21701
O	+1.79495	−4.86583	−3.12954
H	+1.77960	−5.25144	−2.20332
H	+2.64724	−2.97466	−2.72392
C	+4.14501	−4.55319	−2.52875
H	+4.31263	−5.60058	−2.89707
O	+3.86136	−4.58628	−1.07719

C	+3.91461	−5.90592	−0.46102
O	+4.97516	−5.90129	+0.54382
C	+4.76882	−4.88029	+1.60456
C	+6.01792	−5.00917	+2.48040
O	+6.11645	−3.94298	+3.43381
H	+5.40329	−4.06213	+4.11061
H	+6.01284	−5.99957	+3.00783
H	+6.92036	−4.97046	+1.83451
H	+4.72158	−3.86866	+1.12649
H	+4.19924	−6.68463	−1.21900
C	+2.59154	−6.23611	+0.22210
O	+1.54859	−6.22754	−0.77563
H	+0.66879	−6.21606	−0.31220
H	+2.66663	−7.26992	+0.66770
C	+2.28078	−5.23826	+1.33633
O	+1.07584	−5.69344	+1.99809
H	+1.00530	−5.21999	+2.87946
H	+2.11369	−4.21298	+0.90364
C	+3.45023	−5.18820	+2.32851
H	+3.52726	−6.16228	+2.88124
O	+3.16509	−4.10311	+3.29537
C	+3.03301	−4.52407	+4.68518
O	+4.11296	−3.90473	+5.45419
C	+4.10311	−2.42329	+5.35313
C	+5.33340	−1.97502	+6.14708
O	+5.68861	−0.62551	+5.84346
H	+4.99801	−0.02661	+6.22246
H	+5.14271	−2.09617	+7.24723
H	+6.19544	−2.62378	+5.87955
H	+4.20897	−2.14396	+4.27267
H	+3.17130	−5.63447	+4.77810
C	+1.69883	−4.06334	+5.26141
O	+0.64298	−4.69527	+4.50811
H	−0.21306	−4.22891	+4.70685
H	+1.63732	−4.39637	+6.33730
C	+1.57233	−2.54243	+5.19977
O	+0.34775	−2.17617	+5.87977
H	+0.37548	−1.18770	+6.05247
H	+1.52731	−2.20421	+4.12726
C	+2.77248	−1.88150	+5.89105

H	+2.70784	−2.03022	+7.00159
O	+2.70709	−0.43646	+5.58401
C	+2.59404	+0.44615	+6.73746
O	+3.78181	+1.29829	+6.77482
C	+3.95416	+2.09276	+5.53097
C	+5.27629	+2.83726	+5.73757
O	+5.76553	+3.39052	+4.51579
H	+5.17543	+4.13992	+4.25393
H	+5.14221	+3.64147	+6.51031
H	+6.03839	+2.12422	+6.11963
H	+4.02973	+1.38436	+4.66637
H	+2.59509	−0.14566	+7.69201
C	+1.35700	+1.32817	+6.61080
O	+0.19516	+0.47155	+6.57509
H	−0.57675	+1.00195	+6.23971
H	+1.29569	+1.99721	+7.51690
C	+1.42931	+2.19404	+5.35417
O	+0.28990	+3.08682	+5.37530
H	+0.46139	+3.82345	+4.71620
H	+1.38800	+1.54483	+4.43648
C	+2.73289	+3.00378	+5.34853
H	+2.70429	+3.78272	+6.15616
O	+2.83047	+3.66963	+4.03069
C	+2.88446	+5.12576	+4.07100
O	+4.17158	+5.55188	+3.52382
C	+4.38871	+5.08780	+2.12916
C	+5.80314	+5.56741	+1.79030
O	+6.30098	+4.94868	+0.60438
H	+5.80120	+5.30866	−0.16928
H	+5.80797	+6.68582	+1.68501
H	+6.48533	+5.30291	+2.62678
H	+4.33497	+3.96924	+2.11580
H	+2.85273	+5.49368	+5.13167
C	+1.76688	+5.72658	+3.22572
O	+0.50386	+5.29606	+3.77486
H	−0.21151	+5.48706	+3.11030
H	+1.83340	+6.85103	+3.28832
C	+1.89452	+5.30169	+1.76379
O	+0.87143	+6.00298	+1.01753
H	+1.10814	+5.95505	+0.04360

H	+1.73913	+4.19048	+1.66984
C	+3.28664	+5.66991	+1.23367
H	+3.38860	+6.78564	+1.16675
O	+3.41363	+5.08575	−0.11993
C	+3.64181	+6.04710	−1.19298
O	+4.95215	+5.77820	−1.78130
C	+5.07330	+4.40118	−2.32622
C	+6.51988	+4.32318	−2.82370
O	+6.91327	+2.98011	−3.10196
H	+6.42799	+2.66999	−3.90567
H	+6.64032	+4.96813	−3.73581
H	+7.19532	+4.72127	−2.03603
H	+4.89985	+3.67309	−1.49260
H	+3.68582	+7.09217	−0.78402
C	+2.57700	+5.90341	−2.27441
O	+1.29071	+6.17210	−1.67802
H	+0.58037	+5.85602	−2.29871
H	+2.77746	+6.66615	−3.08090
C	+2.60689	+4.50650	−2.89168
O	+1.65069	+4.49105	−3.97807
H	+1.84038	+3.68786	−4.54937
H	+2.31886	+3.73848	−2.12026
C	+4.01473	+4.19740	−3.41859
H	+4.24198	+4.84266	−4.30837
O	+4.02842	+2.77623	−3.82826
C	+4.31556	+2.53328	−5.23769
O	+5.56453	+1.77978	−5.32840
H	+4.48563	+3.50173	−5.78021
C	+3.20045	+1.70884	−5.87026
O	+1.97632	+2.47057	−5.79352
H	+1.21263	+1.86112	−5.98033
H	+3.45512	+1.52572	−6.95343
C	+3.04690	+0.36741	−5.15629
O	+2.05622	−0.40078	−5.88011
H	+2.12850	−1.35733	−5.58356
H	+2.69974	+0.53551	−4.09853
C	+4.38814	−0.37884	−5.13834
H	+4.64329	−0.73308	−6.17233
C	+5.51608	+0.49798	−4.57864
H	+5.31187	+0.72760	−3.50105

C	+6.91740	−0.09681	−4.74764
H	+7.04461	−0.47421	−5.79885
H	+7.67258	+0.70152	−4.58149
O	+7.16626	−1.13835	−3.80458
H	+6.58897	−1.91059	−4.02477

345

* E = 5.782 kcal/mol

C	−0.73956	−0.87359	+0.09667
C	−0.85824	+0.18375	−0.71932
C	+0.23041	+0.90700	−1.36547
C	+1.56300	+0.79912	−1.25409
C	+2.41424	−0.06030	−0.42241
C	+2.42914	−0.06692	+0.91777
C	+1.50327	+0.74976	+1.75056
H	+2.05862	+1.28547	+2.52134
C	+3.39374	−0.91982	+1.68926
C	+4.57705	−1.38600	+0.86617
C	+4.09710	−1.94401	−0.45069
C	+3.38935	−0.86817	−1.23741
C	−1.97139	−1.55732	+0.58624
C	−3.04518	−0.59107	+1.10901
C	−3.08277	+0.71781	+0.31798
C	−2.28492	+0.56946	−0.98955
C	−4.45076	+1.10825	−0.25064
C	−4.10399	+2.11031	−1.36065
C	−2.64909	+1.81928	−1.77336
O	+5.14732	−2.40153	−1.26486
H	+5.56581	−3.16768	−0.87291
C	−2.50230	+1.84242	+1.16501
C	−5.47303	+1.61629	+0.74011
H	+3.39041	−2.76021	−0.25430
H	+2.87573	−1.32075	−2.08644
H	+4.15000	−0.20856	−1.66509
H	+5.14830	−2.13472	+1.41311
H	+5.24643	−0.55118	+0.65986
H	+3.74130	−0.36170	+2.55947
H	+2.85450	−1.78151	+2.09793
H	+0.78051	+0.11257	+2.26652
H	+0.94388	+1.46559	+1.15748
H	+2.13345	+1.39865	−1.95886

H	−0.10305	+1.62588	−2.10270
H	+0.22655	−1.27414	+0.35883
H	−1.72954	−2.30035	+1.34334
H	−2.40141	−2.11521	−0.25280
H	−4.01419	−1.09044	+1.07004
H	−2.85250	−0.37700	+2.16017
H	−4.85123	+0.20472	−0.71828
H	−4.19996	+3.12920	−0.98566
H	−4.79130	+2.02556	−2.19840
H	−1.99874	+2.65158	−1.50365
H	−2.54153	+1.67397	−2.84630
H	−2.53062	+2.80301	+0.65228
H	−1.46715	+1.62612	+1.42460
H	−3.06116	+1.94940	+2.09362
H	−6.42145	+1.81306	+0.24633
H	−5.14864	+2.54657	+1.20510
H	−5.65825	+0.88894	+1.53032
H	−2.74738	−0.27783	−1.51227
O	−7.16067	−2.69016	−2.91556
H	−6.48846	−2.89052	−3.61267
C	−7.22137	−3.79332	−2.01247
H	−7.41131	−4.75116	−2.56895
H	−8.07811	−3.62545	−1.32511
C	−5.93664	−3.92354	−1.18907
H	−5.65932	−2.94178	−0.72764
O	−6.29378	−4.86854	−0.09939
C	−5.20737	−5.06448	+0.85810
O	−4.85009	−3.76683	+1.42113
H	−5.60637	−5.74460	+1.65799
C	−4.01610	−5.67935	+0.13129
O	−2.94593	−5.84376	+1.08792
H	−2.10012	−6.00382	+0.59013
H	−4.31845	−6.69146	−0.26361
C	−3.56305	−4.80196	−1.03447
O	−2.53678	−5.52647	−1.75408
H	−2.39146	−5.06143	−2.63156
H	−3.13842	−3.83706	−0.63996
C	−4.73982	−4.48556	−1.96796
H	−5.03995	−5.40550	−2.53620
O	−4.27347	−3.45119	−2.92013

C	−4.29497	−3.84084	−4.32571
O	−5.24747	−2.98254	−5.02714
C	−4.90505	−1.53862	−4.94139
C	−6.06236	−0.83845	−5.65974
O	−6.05969	+0.56870	−5.42257
H	−5.28715	+0.96453	−5.89596
H	−6.00822	−1.05426	−6.76105
H	−7.02363	−1.24764	−5.28109
H	−4.87198	−1.24614	−3.86114
H	−4.67509	−4.89206	−4.43632
C	−2.91651	−3.66776	−4.95394
O	−1.98684	−4.50561	−4.23557
H	−1.06114	−4.23533	−4.48300
H	−2.96778	−4.00906	−6.02816
C	−2.46743	−2.20741	−4.91979
O	−1.21901	−2.12072	−5.64692
H	−1.05542	−1.15670	−5.87158
H	−2.31279	−1.87581	−3.85518
C	−3.52784	−1.31793	−5.58203
H	−3.57303	−1.52953	−6.68332
O	−3.11598	+0.08862	−5.37212
C	−2.88997	+0.86058	−6.58961
O	−3.86243	+1.95063	−6.63038
C	−3.77965	+2.84250	−5.44546
C	−4.91193	+3.85213	−5.65617
O	−5.18969	+4.59231	−4.46813
H	−4.42015	+5.18317	−4.27754
H	−4.64790	+4.54424	−6.50145
H	−5.83643	+3.30598	−5.94221
H	−3.96197	+2.22821	−4.52662
H	−3.08177	+0.22899	−7.49828
C	−1.48613	+1.45658	−6.59811
O	−0.53668	+0.37018	−6.55611
H	+0.35472	+0.73693	−6.31047
H	−1.34695	+2.03367	−7.55715
C	−1.27918	+2.39814	−5.41350
O	+0.01605	+3.02529	−5.57250
H	+0.06562	+3.79493	−4.93058
H	−1.29919	+1.81070	−4.45315
C	−2.37974	+3.46818	−5.37818

H	−2.23510	+4.19575	−6.22037
O	−2.25130	+4.18149	−4.08727
C	−1.96220	+5.60724	−4.18274
O	−3.08861	+6.34367	−3.61193
C	−3.36195	+5.97364	−2.19885
C	−4.60642	+6.78667	−1.83046
O	−5.21234	+6.30959	−0.62950
H	−4.61347	+6.51898	+0.12922
H	−4.33423	+7.87262	−1.73250
H	−5.35291	+6.69706	−2.64869
H	−3.57955	+4.87585	−2.15652
H	−1.88501	+5.92447	−5.25733
C	−0.70312	+5.94850	−3.39373
O	+0.39949	+5.21614	−3.96900
H	+1.16365	+5.24244	−3.33204
H	−0.50444	+7.05456	−3.49164
C	−0.87211	+5.60058	−1.91563
O	+0.30805	+6.06555	−1.21856
H	+0.09929	+6.08883	−0.23766
H	−0.97246	+4.48511	−1.79278
C	−2.12219	+6.28747	−1.35012
H	−1.95829	+7.39634	−1.29319
O	−2.33078	+5.75369	+0.01530
C	−2.31978	+6.74774	+1.08242
O	−3.63824	+6.75869	+1.71252
C	−4.02171	+5.43436	+2.26510
C	−5.45004	+5.63918	+2.77759
O	−6.08843	+4.39611	+3.06834
H	−5.66264	+4.00705	+3.87134
H	−5.43484	+6.29834	+3.68718
H	−6.04593	+6.15525	+1.99452
H	−4.00305	+4.68330	+1.43368
H	−2.15963	+7.77664	+0.66149
C	−1.27376	+6.39603	+2.13509
O	+0.01778	+6.37134	+1.49039
H	+0.66891	+5.92781	+2.09768
H	−1.27686	+7.19944	+2.92683
C	−1.57559	+5.04926	+2.79110
O	−0.62247	+4.86659	+3.86590
H	−0.95285	+4.11849	+4.44684

H	−1.46005	+4.22229	+2.03693
C	−3.00739	+5.03499	+3.34546
H	−3.08567	+5.72299	+4.22866
O	−3.29884	+3.64927	+3.77851
C	−3.60466	+3.48453	+5.19482
O	−4.98121	+3.00903	+5.31732
C	−5.21387	+1.72156	+4.61243
C	−6.71205	+1.46057	+4.79330
O	−7.17854	+0.43602	+3.91616
H	−6.81302	−0.42950	+4.22428
H	−6.92311	+1.19174	+5.86354
H	−7.27234	+2.39160	+4.56072
H	−4.96339	+1.86088	+3.52950
H	−3.56119	+4.47355	+5.72526
C	−2.67104	+2.45816	+5.82753
O	−1.31836	+2.94031	+5.68627
H	−0.69049	+2.19411	+5.88323
H	−2.92096	+2.37264	+6.92430
C	−2.83059	+1.08598	+5.17358
O	−2.00956	+0.15130	+5.91432
H	−2.30230	−0.77753	+5.67297
H	−2.48742	+1.12690	+4.10168
C	−4.30048	+0.64741	+5.21824
H	−4.60433	+0.42572	+6.27563
O	−4.41301	−0.58735	+4.40823
C	−4.89645	−1.76314	+5.12434
O	−6.17441	−2.16566	+4.54180
H	−5.10158	−1.51305	+6.19987
C	−3.90827	−2.91668	+4.98723
O	−2.65075	−2.49400	+5.55807
H	−1.93767	−3.11820	+5.25724
H	−4.30096	−3.79995	+5.56808
C	−3.72967	−3.31670	+3.52381
O	−2.89363	−4.49935	+3.48810
H	−2.96282	−4.89646	+2.56924
H	−3.22668	−2.48178	+2.96324
C	−5.08835	−3.61652	+2.87528
H	−5.50649	−4.57455	+3.28369
C	−6.08020	−2.46508	+3.08961
H	−5.71205	−1.54770	+2.56193

C	−7.52109	−2.78607	+2.68054
H	−7.82242	−3.78006	+3.10983
H	−8.19859	−2.01454	+3.10549
O	−7.68366	−2.77991	+1.26322
H	−7.19747	−3.55446	+0.88673
O	+5.70859	−5.36849	−1.90056
H	+4.95127	−5.95988	−1.66570
C	+5.65516	−5.13109	−3.31278
H	+5.54036	−6.10061	−3.86655
H	+6.62025	−4.67448	−3.61935
C	+4.51299	−4.17732	−3.66707
H	+4.55645	−3.26584	−3.01067
O	+4.78400	−3.77025	−5.07213
C	+3.84871	−2.74936	−5.53704
O	+3.92782	−1.61252	−4.62835
H	+4.17756	−2.45593	−6.57020
C	+2.43970	−3.33360	−5.52834
O	+1.52528	−2.31163	−5.98102
H	+0.60089	−2.58736	−5.73905
H	+2.40449	−4.20472	−6.24405
C	+2.05729	−3.81958	−4.13108
O	+0.77089	−4.47667	−4.23269
H	+0.63793	−5.02575	−3.40453
H	+1.98288	−2.94301	−3.42743
C	+3.11294	−4.80177	−3.60709
H	+3.08030	−5.75484	−4.19968
O	+2.77222	−5.09559	−2.19515
C	+2.50128	−6.49530	−1.90014
O	+3.52412	−6.97558	−0.96935
C	+3.55979	−6.20032	+0.29587
C	+4.73319	−6.80283	+1.07416
O	+5.12251	−5.97080	+2.16700
H	+4.42254	−6.02432	+2.86369
H	+4.46022	−7.82978	+1.43696
H	+5.60580	−6.90484	+0.39338
H	+3.75830	−5.12508	+0.04773
H	+2.59719	−7.12254	−2.82664
C	+1.13194	−6.65136	−1.24681
O	+0.13391	−6.17203	−2.17205
H	−0.72612	−6.06723	−1.68318

H	+0.95287	−7.74640	−1.04314
C	+1.05962	−5.88191	+0.07120
O	−0.21980	−6.17788	+0.68029
H	−0.17725	−5.89621	+1.64195
H	+1.14178	−4.77634	−0.12424
C	+2.20113	−6.32045	+0.99799
H	+2.03202	−7.37402	+1.34527
O	+2.19388	−5.41254	+2.16660
C	+1.98381	−6.05941	+3.45642
O	+3.18381	−5.86664	+4.26849
C	+3.51763	−4.43380	+4.47404
C	+4.82235	−4.46300	+5.27604
O	+5.46883	−3.19093	+5.28041
H	+4.93269	−2.56758	+5.82995
H	+4.61335	−4.80492	+6.32567
H	+5.51586	−5.19738	+4.81255
H	+3.67859	−3.96060	+3.47156
H	+1.86114	−7.16821	+3.32795
C	+0.79527	−5.43270	+4.17612
O	−0.37829	−5.63544	+3.36111
H	−1.11327	−5.06264	+3.71066
H	+0.65432	−5.95545	+5.16546
C	+1.03156	−3.94416	+4.42686
O	−0.08041	−3.45347	+5.21213
H	+0.17996	−2.56484	+5.59920
H	+1.07918	−3.39220	+3.44643
C	+2.34903	−3.74111	+5.18760
H	+2.24926	−4.12423	+6.23766
O	+2.61680	−2.28619	+5.22035
C	+2.70378	−1.69731	+6.55216
O	+4.05568	−1.17496	+6.73645
C	+4.43039	−0.16522	+5.71291
C	+5.88235	+0.18835	+6.04896
O	+6.51501	+0.89483	+4.98313
H	+6.11290	+1.79624	+4.92041
H	+5.91464	+0.78974	+6.99764
H	+6.45209	−0.74976	+6.22321
H	+4.36185	−0.64325	+4.70184
H	+2.55442	−2.48157	+7.34190
C	+1.70896	−0.55065	+6.68928

O	+0.38136	−1.08686	+6.50615
H	−0.24968	−0.33333	+6.35130
H	+1.79428	−0.11881	+7.72763
C	+1.99814	+0.54604	+5.66598
O	+1.09621	+1.64479	+5.93698
H	+1.43975	+2.45541	+5.45511
H	+1.82086	+0.15577	+4.62458
C	+3.45374	+1.01597	+5.79122
H	+3.59441	+1.57576	+6.75368
O	+3.71760	+1.92495	+4.65320
C	+4.10578	+3.28398	+5.01802
O	+5.46701	+3.51750	+4.54282
C	+5.60435	+3.33624	+3.07420
C	+7.09770	+3.54657	+2.80654
O	+7.47132	+3.09242	+1.50659
H	+7.06412	+3.69239	+0.83412
H	+7.35513	+4.63287	+2.93631
H	+7.68468	+2.96840	+3.55231
H	+5.29831	+2.29032	+2.81764
H	+4.13412	+3.39890	+6.13491
C	+3.17350	+4.29833	+4.36526
O	+1.83354	+4.04302	+4.83924
H	+1.19164	+4.53595	+4.26056
H	+3.48760	+5.33354	+4.68394
C	+3.23468	+4.20436	+2.84163
O	+2.42973	+5.28253	+2.30749
H	+2.65702	+5.38594	+1.33519
H	+2.81810	+3.21515	+2.50265
C	+4.68538	+4.33476	+2.35761
H	+5.05058	+5.38433	+2.51498
O	+4.69346	+4.02455	+0.91101
C	+5.16110	+5.09949	+0.04398
O	+6.37430	+4.64849	−0.63440
C	+6.16512	+3.42105	−1.44643
C	+7.55634	+3.09924	−2.00060
O	+7.61640	+1.78709	−2.55641
H	+7.06985	+1.76772	−3.37993
H	+7.84266	+3.86877	−2.76821
H	+8.29682	+3.15478	−1.17370
H	+5.80726	+2.60502	−0.76879

H	+5.45202	+5.99851	+0.65131
C	+4.10649	+5.43667	−1.00380
O	+2.91454	+5.87688	−0.31892
H	+2.15597	+5.85912	−0.96239
H	+4.49466	+6.27779	−1.64762
C	+3.80724	+4.22934	−1.89059
O	+2.89329	+4.66722	−2.92461
H	+2.89798	+3.98003	−3.65586
H	+3.32966	+3.41130	−1.28239
C	+5.10575	+3.70695	−2.51937
H	+5.49685	+4.45090	−3.26332
O	+4.78047	+2.44303	−3.21760
C	+5.02851	+2.44560	−4.65629
O	+6.06067	+1.45561	−4.94930
H	+5.43906	+3.43871	−4.98323
C	+3.76165	+2.07406	−5.41799
O	+2.75318	+3.06741	−5.13530
H	+1.87364	+2.71966	−5.44405
H	+3.98937	+2.08267	−6.52255
C	+3.27793	+0.68323	−5.01615
O	+2.13905	+0.35602	−5.84856
H	+1.98292	−0.63352	−5.78297
H	+2.96986	+0.68291	−3.93251
C	+4.39635	−0.34520	−5.22805
H	+4.57405	−0.49662	−6.32623
C	+5.69644	+0.07630	−4.52805
H	+5.54618	+0.07097	−3.41834
C	+6.91962	−0.75891	−4.92100
H	+6.95068	−0.87167	−6.04073
H	+7.84078	−0.21851	−4.61298
O	+6.92876	−2.03450	−4.28549
H	+6.13472	−2.54348	−4.58173

345

* E = 6.041 kcal/mol

C	−0.92356	+1.10819	+0.41861
C	−1.32014	−0.12333	+0.75848
C	−0.45645	−1.10666	+1.40718
C	+0.87803	−1.22193	+1.47446
C	+1.96927	−0.45548	+0.87337
C	+2.12089	−0.15372	−0.42628

C	+1.14421	−0.48300	−1.49552
H	+1.65633	−0.87607	−2.37436
C	+3.33849	+0.63551	−0.81033
C	+4.63776	+0.11207	−0.18741
C	+4.41886	−0.56434	+1.16134
C	+3.11350	−0.09296	+1.78648
C	−1.79904	+2.14195	−0.21006
C	−3.27877	+1.77737	−0.23005
C	−3.44812	+0.30321	−0.53037
C	−2.76947	−0.48424	+0.58678
C	−4.86840	−0.25564	−0.42795
C	−4.62832	−1.76511	−0.23731
C	−3.19379	−1.92003	+0.30629
O	+4.40205	−1.97124	+1.04105
H	+3.63745	−2.20727	+0.51381
C	−2.84917	−0.02668	−1.89302
C	−5.80984	+0.07644	−1.56391
H	+5.24920	−0.35748	+1.82851
H	+3.14973	+0.98906	+1.94089
H	+2.99131	−0.55522	+2.76451
H	+5.33768	+0.93760	−0.08169
H	+5.10939	−0.61633	−0.84284
H	+3.43249	+0.68410	−1.89378
H	+3.18311	+1.66675	−0.47900
H	+0.61260	+0.41738	−1.81416
H	+0.39868	−1.19929	−1.16428
H	+1.23336	−2.00527	+2.13657
H	−1.00275	−1.86234	+1.96041
H	+0.10654	+1.39058	+0.58833
H	−1.44359	+2.31029	−1.23252
H	−1.65243	+3.09605	+0.29969
H	−3.72188	+1.98942	+0.74544
H	−3.80326	+2.39575	−0.95971
H	−5.29184	+0.14628	+0.49616
H	−4.73846	−2.28153	−1.19120
H	−5.36521	−2.19829	+0.43401
H	−2.53494	−2.37648	−0.43331
H	−3.15462	−2.55154	+1.19133
H	−3.07526	−1.04679	−2.19901
H	−1.76705	+0.08308	−1.89371

H	−3.25835	+0.63654	−2.65431
H	−6.80335	−0.31747	−1.36423
H	−5.47224	−0.35764	−2.50484
H	−5.90537	+1.15230	−1.70285
H	−3.27840	−0.19034	+1.51320
O	−6.26368	−4.56704	−2.53206
H	−5.61729	−4.49439	−3.27729
C	−5.90675	−5.69322	−1.73028
H	−5.82130	−6.61842	−2.36249
H	−6.72198	−5.86010	−0.99389
C	−4.59417	−5.45580	−0.97839
H	−4.60426	−4.45678	−0.47376
O	−4.57107	−6.50755	+0.07103
C	−3.41193	−6.38772	+0.95332
O	−3.41892	−5.05121	+1.53523
H	−3.53124	−7.16947	+1.75051
C	−2.14694	−6.59277	+0.12814
O	−1.01301	−6.48707	+1.01533
H	−0.18983	−6.37554	+0.46730
H	−2.16989	−7.62596	−0.32326
C	−2.05859	−5.55701	−0.99172
O	−0.91074	−5.89107	−1.80793
H	−0.99010	−5.38810	−2.67222
H	−1.92674	−4.52846	−0.55268
C	−3.33404	−5.58387	−1.84512
H	−3.37033	−6.52840	−2.45031
O	−3.26877	−4.42011	−2.75930
C	−3.30798	−4.74163	−4.18088
O	−4.51407	−4.14679	−4.75317
C	−4.56278	−2.67045	−4.59551
C	−5.93234	−2.27806	−5.15601
O	−6.26153	−0.92547	−4.83980
H	−5.68809	−0.33225	−5.38434
H	−5.94638	−2.43840	−6.26736
H	−6.70895	−2.93445	−4.70805
H	−4.49692	−2.42654	−3.50296
H	−3.39947	−5.85088	−4.33164
C	−2.08736	−4.17165	−4.89566
O	−0.90787	−4.74851	−4.29655
H	−0.11347	−4.22633	−4.58913

H	−2.13495	−4.47297	−5.98189
C	−2.04950	−2.64611	−4.80750
O	−0.93421	−2.19605	−5.61242
H	−1.05271	−1.21570	−5.78910
H	−1.90223	−2.32477	−3.73821
C	−3.36556	−2.05975	−5.33721
H	−3.45443	−2.24848	−6.43997
O	−3.33701	−0.59941	−5.09338
C	−3.41848	+0.23283	−6.28948
O	−4.66165	+0.99951	−6.23995
C	−4.75929	+1.88842	−5.05328
C	−6.14209	+2.53406	−5.18659
O	−6.54677	+3.17627	−3.97843
H	−5.98345	+3.97737	−3.84311
H	−6.13683	+3.26427	−6.04064
H	−6.88862	+1.74479	−5.42065
H	−4.70398	+1.25565	−4.13015
H	−3.47978	−0.40793	−7.20996
C	−2.24249	+1.20178	−6.35359
O	−1.02688	+0.42506	−6.38833
H	−0.25782	+1.02298	−6.18839
H	−2.32486	+1.80302	−7.30461
C	−2.24263	+2.15661	−5.16099
O	−1.17443	+3.11262	−5.36621
H	−1.31987	+3.88088	−4.73714
H	−2.06051	+1.58295	−4.20977
C	−3.59090	+2.88348	−5.06481
H	−3.69988	+3.60876	−5.91430
O	−3.59525	+3.62533	−3.78379
C	−3.73721	+5.07301	−3.89319
O	−4.98882	+5.46263	−3.24583
C	−5.06174	+5.02163	−1.82890
C	−6.45346	+5.45928	−1.36292
O	−6.83651	+4.80042	−0.15624
H	−6.25413	+5.11773	+0.57775
H	−6.47532	+6.57533	−1.22997
H	−7.19769	+5.19716	−2.14536
H	−4.97043	+3.90526	−1.80348
H	−3.82215	+5.38319	−4.96908
C	−2.58379	+5.77172	−3.18145

O	−1.35406	+5.38855	−3.83625
H	−0.59379	+5.61470	−3.23685
H	−2.72108	+6.88699	−3.27247
C	−2.54936	+5.38168	−1.70557
O	−1.51814	+6.16824	−1.06070
H	−1.64894	+6.09002	−0.06876
H	−2.30372	+4.28801	−1.61550
C	−3.90544	+5.64946	−1.03848
H	−4.06487	+6.75384	−0.91854
O	−3.85718	+5.01278	+0.29798
C	−4.05186	+5.91053	+1.43045
O	−5.27080	+5.50877	+2.12972
C	−5.22310	+4.10303	+2.60937
C	−6.61007	+3.85991	+3.21082
O	−6.84442	+2.47239	+3.44991
H	−6.25866	+2.17908	+4.19075
H	−6.71837	+4.44936	+4.16121
H	−7.38302	+4.22000	+2.49841
H	−5.05007	+3.43412	+1.72741
H	−4.22035	+6.96267	+1.07510
C	−2.87519	+5.81699	+2.39586
O	−1.67685	+6.19117	+1.68151
H	−0.89012	+5.90908	+2.22100
H	−3.04447	+6.54512	+3.24043
C	−2.73842	+4.40844	+2.97253
O	−1.69699	+4.45065	+3.97704
H	−1.76839	+3.61694	+4.53094
H	−2.45176	+3.68679	+2.15750
C	−4.06304	+3.95986	+3.60391
H	−4.26947	+4.55865	+4.53038
O	−3.91488	+2.53186	+3.96788
C	−4.06865	+2.23413	+5.38803
O	−5.23666	+1.37152	+5.55364
C	−5.12318	+0.08043	+4.82617
C	−6.46817	−0.60549	+5.08344
O	−6.65006	−1.74905	+4.24993
H	−6.02404	−2.45447	+4.54586
H	−6.54264	−0.89050	+6.16763
H	−7.28798	+0.11207	+4.86468
H	−4.98712	+0.29763	+3.73589

H	−4.28653	+3.17352	+5.96420
C	−2.84015	+1.51172	+5.93062
O	−1.69112	+2.36363	+5.74186
H	−0.87007	+1.81784	+5.87675
H	−2.99094	+1.33488	+7.03482
C	−2.63197	+0.16555	+5.23930
O	−1.52694	−0.49392	+5.90182
H	−1.53941	−1.46151	+5.63843
H	−2.38667	+0.32226	+4.15209
C	−3.90359	−0.68442	+5.35854
H	−4.06800	−0.98256	+6.42795
O	−3.69945	−1.90013	+4.53882
C	−3.73466	−3.16032	+5.27325
O	−4.87855	−3.93662	+4.79803
H	−3.90786	−2.97506	+6.36730
C	−2.46298	−3.96531	+5.02672
O	−1.34024	−3.19569	+5.50615
H	−0.50179	−3.61424	+5.17156
H	−2.52888	−4.92763	+5.61116
C	−2.29812	−4.29085	+3.54365
O	−1.14579	−5.15657	+3.40990
H	−1.16881	−5.56205	+2.49210
H	−2.12809	−3.34034	+2.96185
C	−3.55373	−4.99223	+3.00714
H	−3.61926	−6.03469	+3.41738
C	−4.82685	−4.20119	+3.33733
H	−4.80600	−3.21859	+2.79986
C	−6.12944	−4.95138	+3.04306
H	−6.06878	−5.99311	+3.46055
H	−6.96987	−4.43158	+3.55132
O	−6.41674	−4.98536	+1.64548
H	−5.74908	−5.56192	+1.19873
O	+6.96951	−3.66978	−2.53827
H	+6.38051	−4.41368	−2.26038
C	+6.68944	−3.34942	−3.89925
H	+6.75292	−4.26730	−4.54479
H	+7.46581	−2.63538	−4.24944
C	+5.30968	−2.70338	−4.06001
H	+5.17390	−1.86772	−3.32690
O	+5.33605	−2.12698	−5.42970

C	+4.11516	−1.40146	−5.77059
O	+3.93485	−0.32100	−4.80567
H	+4.27050	−0.98570	−6.80230
C	+2.93576	−2.36604	−5.71825
O	+1.73864	−1.62986	−6.04858
H	+0.94760	−2.17150	−5.78383
H	+3.09701	−3.17440	−6.48814
C	+2.81193	−3.00940	−4.33838
O	+1.74855	−3.98822	−4.40953
H	+1.83200	−4.59833	−3.61653
H	+2.55560	−2.22403	−3.57200
C	+4.13432	−3.68407	−3.95028
H	+4.31251	−4.58116	−4.60072
O	+4.01080	−4.11793	−2.54087
C	+4.15222	−5.55430	−2.31629
O	+5.33135	−5.77927	−1.48712
C	+5.27359	−5.09063	−0.16871
C	+6.60303	−5.46434	+0.49602
O	+6.92595	−4.59455	+1.57743
H	+6.23485	−4.68972	+2.27873
H	+6.56134	−6.53445	+0.84355
H	+7.41730	−5.38825	−0.25693
H	+5.21108	−3.98706	−0.34396
H	+4.33951	−6.08741	−3.28704
C	+2.92610	−6.10033	−1.59338
O	+1.77303	−5.87795	−2.43301
H	+0.95219	−6.00201	−1.88421
H	+3.06145	−7.20872	−1.43560
C	+2.75422	−5.41666	−0.23976
O	+1.64491	−6.04713	+0.44487
H	+1.70860	−5.80960	+1.41801
H	+2.53399	−4.32207	−0.39069
C	+4.03048	−5.56604	+0.59730
H	+4.15311	−6.63453	+0.92008
O	+3.84320	−4.71216	+1.78720
C	+3.97283	−5.37983	+3.07471
O	+5.11833	−4.80769	+3.77467
C	+5.00462	−3.33207	+3.94207
C	+6.33932	−2.93344	+4.57724
O	+6.50855	−1.51603	+4.61720

H	+5.86350	−1.14621	+5.26795
H	+6.40828	−3.36649	+5.61227
H	+7.17018	−3.36001	+3.97577
H	+4.87761	−2.86540	+2.92874
H	+4.19199	−6.47298	+2.93531
C	+2.71709	−5.14943	+3.90956
O	+1.59129	−5.68551	+3.17689
H	+0.75600	−5.32879	+3.57983
H	+2.82034	−5.70232	+4.88663
C	+2.51178	−3.66180	+4.19036
O	+1.38881	−3.53725	+5.09676
H	+1.38753	−2.60165	+5.45812
H	+2.28126	−3.12714	+3.22679
C	+3.77231	−3.04425	+4.81104
H	+3.92400	−3.43663	+5.85179
O	+3.55388	−1.57832	+4.87095
C	+3.54971	−1.00407	+6.21078
O	+4.70135	−0.11107	+6.33744
C	+4.69519	+0.98844	+5.33903
C	+6.00964	+1.73197	+5.59383
O	+6.31489	+2.64451	+4.53961
H	+5.67121	+3.39389	+4.57770
H	+5.95372	+2.26998	+6.57857
H	+6.83717	+0.99335	+5.65715
H	+4.68644	+0.53675	+4.31385
H	+3.68566	−1.80686	+6.98436
C	+2.27701	−0.19829	+6.44916
O	+1.14826	−1.08588	+6.30657
H	+0.32511	−0.53539	+6.21409
H	+2.30068	+0.20438	+7.50286
C	+2.16729	+0.97241	+5.47358
O	+1.01037	+1.75341	+5.85849
H	+1.06896	+2.64114	+5.39522
H	+2.03352	+0.58698	+4.42441
C	+3.43297	+1.83754	+5.54016
H	+3.47837	+2.37758	+6.52280
O	+3.34568	+2.82786	+4.44224
C	+3.33277	+4.22445	+4.86263
O	+4.53238	+4.87423	+4.33691
C	+4.62912	+4.79466	+2.85671

C	+5.97268	+5.45376	+2.53077
O	+6.38370	+5.18279	+1.19119
H	+5.78331	+5.66754	+0.57303
H	+5.90297	+6.56126	+2.70730
H	+6.74954	+5.04974	+3.21481
H	+4.63500	+3.71476	+2.56062
H	+3.39371	+4.30119	+5.98131
C	+2.10600	+4.94012	+4.30805
O	+0.92862	+4.29392	+4.83502
H	+0.14084	+4.59538	+4.30708
H	+2.12743	+6.01279	+4.65659
C	+2.09932	+4.91247	+2.78078
O	+0.97805	+5.71160	+2.33333
H	+1.11488	+5.92197	+1.36209
H	+1.98120	+3.85368	+2.41685
C	+3.41123	+5.49342	+2.23726
H	+3.45279	+6.59772	+2.43276
O	+3.42029	+5.25457	+0.77686
C	+3.49896	+6.45148	−0.05240
O	+4.74321	+6.39711	−0.81708
C	+4.84797	+5.17321	−1.65238
C	+6.23335	+5.27314	−2.29767
O	+6.64059	+4.03060	−2.86941
H	+6.07458	+3.84653	−3.65906
H	+6.23114	+6.08508	−3.07451
H	+6.97615	+5.55090	−1.51931
H	+4.78828	+4.28052	−0.97908
H	+3.55519	+7.37357	+0.58594
C	+2.32881	+6.49946	−1.02816
O	+1.10595	+6.56795	−0.26463
H	+0.34171	+6.38930	−0.87632
H	+2.42294	+7.42831	−1.66067
C	+2.32983	+5.27190	−1.93743
O	+1.26670	+5.44471	−2.90445
H	+1.41573	+4.78876	−3.64887
H	+2.14239	+4.34390	−1.32784
C	+3.68276	+5.14505	−2.65117
H	+3.79620	+5.96669	−3.40715
O	+3.69040	+3.83756	−3.34452
C	+3.85195	+3.90064	−4.79257

O	+5.10016	+3.22821	−5.14524
H	+3.95630	+4.96569	−5.13298
C	+2.69625	+3.18961	−5.48688
O	+1.47262	+3.86527	−5.12745
H	+0.70223	+3.29287	−5.38932
H	+2.84489	+3.26232	−6.60253
C	+2.64117	+1.71595	−5.08694
O	+1.59541	+1.09429	−5.86983
H	+1.72095	+0.09949	−5.82592
H	+2.40329	+1.62265	−3.98966
C	+3.99207	+1.04546	−5.37172
H	+4.16238	+0.98117	−6.47911
C	+5.13971	+1.81142	−4.69938
H	+5.00875	+1.78639	−3.58725
C	+6.54061	+1.33350	−5.09288
H	+6.60948	+1.24703	−6.21098
H	+7.28597	+2.08958	−4.76480
O	+6.87002	+0.09250	−4.47085
H	+6.31700	−0.61909	−4.87765

345

* E = 6.082 kcal/mol

C	−0.97029	−0.54507	+0.03822
C	−1.07629	+0.49328	−0.80303
C	+0.01734	+1.17620	−1.48019
C	+1.35064	+1.12155	−1.32898
C	+2.20886	+0.40245	−0.38485
C	+2.10944	+0.46302	+0.95456
C	+1.09362	+1.28412	+1.66689
H	+1.54369	+1.78305	+2.52560
C	+3.03511	−0.29612	+1.86105
C	+3.96187	−1.26175	+1.14989
C	+4.48906	−0.61645	−0.11337
C	+3.33450	−0.34837	−1.05425
C	−2.21139	−1.19799	+0.54836
C	−3.27958	−0.20524	+1.03555
C	−3.28220	+1.09314	+0.22694
C	−2.49489	+0.90124	−1.07975
C	−4.63979	+1.52262	−0.33769
C	−4.26669	+2.49251	−1.46927
C	−2.82191	+2.15069	−1.87923

O	+5.17814	+0.57747	+0.18452
H	+4.53541	+1.24502	+0.43021
C	−2.66452	+2.21048	+1.05602
C	−5.62905	+2.08635	+0.65733
H	+5.22048	−1.25675	−0.59684
H	+2.96089	−1.28720	−1.46732
H	+3.69868	+0.23498	−1.90267
H	+3.42606	−2.17239	+0.88125
H	+4.78542	−1.54241	+1.80101
H	+3.61909	+0.42436	+2.44263
H	+2.43179	−0.82693	+2.60132
H	+0.28825	+0.65439	+2.05256
H	+0.64235	+2.02922	+1.01923
H	+1.91368	+1.65966	−2.08520
H	−0.31161	+1.80981	−2.29450
H	−0.00767	−0.95473	+0.30365
H	−1.98085	−1.91804	+1.33098
H	−2.64216	−1.77949	−0.27413
H	−4.25435	−0.69216	+0.98488
H	−3.10419	+0.02145	+2.08709
H	−5.07886	+0.62668	−0.78490
H	−4.33174	+3.52162	−1.11750
H	−4.95766	+2.40853	−2.30377
H	−2.14351	+2.96268	−1.61659
H	−2.71546	+1.99164	−2.95031
H	−2.66228	+3.16463	+0.53079
H	−1.63807	+1.96670	+1.32030
H	−3.21964	+2.34766	+1.98229
H	−6.58183	+2.29386	+0.17640
H	−5.27050	+3.01995	+1.08796
H	−5.81668	+1.38830	+1.47317
H	−2.97877	+0.05694	−1.58797
O	−7.48776	−2.03453	−2.51368
H	−6.88345	−2.39517	−3.20837
C	−7.66866	−3.03120	−1.50854
H	−8.00964	−3.99885	−1.96696
H	−8.46823	−2.68191	−0.82055
C	−6.38563	−3.26675	−0.70620
H	−5.96145	−2.29678	−0.34053
O	−6.82963	−4.05725	+0.47080

C	−5.74767	−4.31587	+1.41818
O	−5.20239	−3.03447	+1.85299
H	−6.20660	−4.86358	+2.28465
C	−4.67351	−5.14677	+0.72439
O	−3.60429	−5.37308	+1.66906
H	−2.80273	−5.68644	+1.17097
H	−5.12087	−6.13830	+0.42691
C	−4.14698	−4.44072	−0.52395
O	−3.24779	−5.35495	−1.19593
H	−3.07565	−4.99554	−2.11692
H	−3.58776	−3.51032	−0.22645
C	−5.30361	−4.05478	−1.45661
H	−5.74428	−4.97440	−1.92495
O	−4.73982	−3.18721	−2.51646
C	−4.86352	−3.70296	−3.87554
O	−5.72286	−2.79853	−4.63676
C	−5.19500	−1.41100	−4.70882
C	−6.27504	−0.64162	−5.47517
O	−6.07577	+0.76942	−5.40027
H	−5.27360	+1.00177	−5.92906
H	−6.29187	−0.98253	−6.54561
H	−7.26732	−0.87124	−5.03078
H	−5.08965	−1.01740	−3.66586
H	−5.37922	−4.70081	−3.87180
C	−3.49831	−3.77299	−4.55038
O	−2.65765	−4.65501	−3.77688
H	−1.71466	−4.52637	−4.06676
H	−3.63194	−4.20311	−5.58479
C	−2.86394	−2.38794	−4.66830
O	−1.63740	−2.53823	−5.42169
H	−1.34939	−1.62895	−5.73373
H	−2.63517	−1.97961	−3.64446
C	−3.82215	−1.43432	−5.39469
H	−3.92810	−1.74029	−6.46928
O	−3.23302	−0.07793	−5.32883
C	−2.91969	+0.53103	−6.61737
O	−3.74276	+1.72917	−6.77396
C	−3.53770	+2.71797	−5.68427
C	−4.51924	+3.84857	−6.00645
O	−4.71243	+4.71430	−4.88817

H	−3.87029	+5.20359	−4.71673
H	−4.15155	+4.42731	−6.89717
H	−5.50483	+3.40916	−6.27184
H	−3.80480	+2.22976	−4.71249
H	−3.19827	−0.15885	−7.45859
C	−1.45172	+0.93856	−6.67458
O	−0.64478	−0.25007	−6.53813
H	+0.28933	+0.02438	−6.33355
H	−1.24831	+1.41072	−7.67850
C	−1.12184	+1.94353	−5.57320
O	+0.24832	+2.36921	−5.76553
H	+0.39600	+3.19494	−5.21450
H	−1.23017	+1.45618	−4.56351
C	−2.06506	+3.15121	−5.65662
H	−1.82073	+3.76831	−6.56164
O	−1.83925	+3.95941	−4.43727
C	−1.38329	+5.32606	−4.66272
O	−2.39843	+6.23750	−4.13860
C	−2.67310	+6.02052	−2.69442
C	−3.80768	+6.99736	−2.37300
O	−4.42166	+6.69690	−1.11967
H	−3.78075	+6.91043	−0.39744
H	−3.41526	+8.05015	−2.37929
H	−4.58617	+6.92093	−3.16224
H	−3.01155	+4.96228	−2.55396
H	−1.29895	+5.53883	−5.76221
C	−0.07138	+5.57870	−3.92802
O	+0.92327	+4.67446	−4.45403
H	+1.70250	+4.66862	−3.83531
H	+0.25321	+6.64072	−4.12564
C	−0.23845	+5.38036	−2.42220
O	+1.01063	+5.75399	−1.79405
H	+0.83756	+5.89035	−0.81539
H	−0.46883	+4.29985	−2.20011
C	−1.38177	+6.26085	−1.89979
H	−1.08763	+7.34272	−1.95177
O	−1.61520	+5.88565	−0.48664
C	−1.44590	+6.96450	+0.48045
O	−2.73012	+7.19849	+1.13803
C	−3.25144	+5.99348	+1.83284

C	−4.61986	+6.42670	+2.36604
O	−5.39390	+5.30837	+2.79994
H	−4.98526	+4.94662	+3.62424
H	−4.48419	+7.16268	+3.20381
H	−5.18075	+6.93689	+1.55373
H	−3.36248	+5.17137	+1.07986
H	−1.17655	+7.92241	−0.04031
C	−0.41477	+6.58230	+1.53674
O	+0.84418	+6.34584	+0.87184
H	+1.45337	+5.87591	+1.50202
H	−0.29793	+7.44565	+2.25339
C	−0.85501	+5.34486	+2.31733
O	+0.11539	+5.12968	+3.36982
H	−0.28378	+4.49337	+4.03486
H	−0.88200	+4.44907	+1.63589
C	−2.25036	+5.56954	+2.91656
H	−2.20160	+6.34282	+3.72852
O	−2.69571	+4.28163	+3.49450
C	−2.95300	+4.29142	+4.92958
O	−4.37051	+4.00610	+5.14601
C	−4.79507	+2.69868	+4.58130
C	−6.29956	+2.63911	+4.86175
O	−6.93817	+1.61599	+4.09856
H	−6.65096	+0.73707	+4.44920
H	−6.47458	+2.48136	+5.96040
H	−6.75865	+3.61158	+4.58164
H	−4.60019	+2.70914	+3.47802
H	−2.76510	+5.31250	+5.35787
C	−2.12479	+3.21796	+5.62714
O	−0.72910	+3.51351	+5.40934
H	−0.19251	+2.71249	+5.65373
H	−2.34276	+3.25646	+6.73322
C	−2.47346	+1.82765	+5.09841
O	−1.73085	+0.86359	+5.88263
H	−2.14336	−0.03940	+5.73684
H	−2.18057	+1.74315	+4.01438
C	−3.98056	+1.57550	+5.23796
H	−4.25469	+1.47535	+6.32172
O	−4.27638	+0.30384	+4.53948
C	−4.86072	−0.74383	+5.36970

O	−6.19999	−1.03733	+4.86500
H	−4.98966	−0.38462	+6.42592
C	−4.01909	−2.01358	+5.29931
O	−2.69934	−1.70011	+5.79415
H	−2.07812	−2.43168	+5.53323
H	−4.48820	−2.79479	+5.96374
C	−3.94792	−2.54898	+3.87017
O	−3.26057	−3.82317	+3.91235
H	−3.42298	−4.29025	+3.03929
H	−3.37250	−1.83061	+3.22308
C	−5.35888	−2.73383	+3.29450
H	−5.86918	−3.59633	+3.79968
C	−6.19882	−1.45740	+3.44012
H	−5.74613	−0.63457	+2.82870
C	−7.68182	−1.63252	+3.09892
H	−8.08116	−2.55107	+3.60841
H	−8.24791	−0.75782	+3.48529
O	−7.89517	−1.70556	+1.68995
H	−7.52307	−2.56005	+1.35895
O	+4.93761	−6.49416	−1.57971
H	+4.09014	−6.90184	−1.27483
C	+4.85656	−6.27686	−2.98767
H	+4.56933	−7.22453	−3.51877
H	+5.86534	−5.97967	−3.34649
C	+3.85714	−5.16973	−3.33575
H	+4.05394	−4.25094	−2.72670
O	+4.14233	−4.85844	−4.76015
C	+3.32694	−3.76198	−5.27894
O	+3.57821	−2.57766	−4.46485
H	+3.66054	−3.59133	−6.33758
C	+1.85848	−4.16483	−5.20260
O	+1.06231	−3.07758	−5.71841
H	+0.11597	−3.22347	−5.44893
H	+1.70231	−5.07653	−5.84838
C	+1.45444	−4.49658	−3.76753
O	+0.08987	−4.97803	−3.79997
H	−0.09876	−5.43658	−2.92791
H	+1.51728	−3.57289	−3.12709
C	+2.38360	−5.57611	−3.19678
H	+2.19817	−6.55488	−3.71359

O	+2.06242	−5.71400	−1.75867
C	+1.59735	−7.03339	−1.34629
O	+2.56519	−7.58587	−0.40073
C	+2.76646	−6.71802	+0.78826
C	+3.85488	−7.43146	+1.59590
O	+4.41393	−6.58146	+2.59656
H	+3.73076	−6.42314	+3.29365
H	+3.43201	−8.36400	+2.05904
H	+4.67410	−7.73564	+0.90954
H	+3.12513	−5.71703	+0.43778
H	+1.57016	−7.73537	−2.22241
C	+0.24296	−6.93004	−0.65409
O	−0.70801	−6.39429	−1.59825
H	−1.52908	−6.12753	−1.10367
H	−0.08422	−7.96445	−0.34596
C	+0.33093	−6.04330	+0.58667
O	−0.95589	−6.09051	+1.24833
H	−0.83800	−5.73997	+2.18100
H	+0.56473	−4.98428	+0.28498
C	+1.42987	−6.55855	+1.52590
H	+1.11712	−7.53439	+1.98358
O	+1.59922	−5.54893	+2.59451
C	+1.35814	−6.02789	+3.95057
O	+2.60230	−5.90830	+4.70748
C	+3.12298	−4.51723	+4.74124
C	+4.45007	−4.62953	+5.49750
O	+5.24400	−3.45320	+5.34861
H	+4.81229	−2.71712	+5.84808
H	+4.24993	−4.83777	+6.58334
H	+5.02789	−5.48623	+5.08890
H	+3.29435	−4.17880	+3.68784
H	+1.09185	−7.11883	+3.94386
C	+0.28715	−5.18203	+4.62975
O	−0.93461	−5.31279	+3.87247
H	−1.57293	−4.61092	+4.17260
H	+0.12051	−5.57823	+5.67241
C	+0.71910	−3.71882	+4.71495
O	−0.28986	−3.01176	+5.47446
H	+0.09846	−2.13384	+5.76693
H	+0.79440	−3.27989	+3.68089

C	+2.08162	−3.61189	+5.41337
H	+1.97741	−3.87722	+6.49883
O	+2.52721	−2.20508	+5.29492
C	+2.74307	−1.51213	+6.56096
O	+4.15499	−1.14960	+6.65410
C	+4.60798	−0.28201	+5.53618
C	+6.10473	−0.08605	+5.79734
O	+6.77373	+0.46050	+4.66278
H	+6.48282	+1.39779	+4.54473
H	+6.24662	+0.57197	+6.69728
H	+6.56418	−1.07238	+6.02336
H	+4.44021	−0.82876	+4.57323
H	+2.53278	−2.19667	+7.42605
C	+1.90306	−0.24157	+6.62297
O	+0.51271	−0.61737	+6.52622
H	−0.02331	+0.19516	+6.32186
H	+2.08218	+0.26144	+7.61643
C	+2.28232	+0.72253	+5.50028
O	+1.52697	+1.94062	+5.70118
H	+1.94261	+2.66107	+5.13951
H	+2.01793	+0.27176	+4.50244
C	+3.78848	+1.01564	+5.54348
H	+4.03360	+1.62642	+6.45241
O	+4.12463	+1.79259	+4.32797
C	+4.66950	+3.12662	+4.57347
O	+6.03635	+3.17220	+4.06503
C	+6.14272	+2.88593	+2.60891
C	+7.64562	+2.98265	+2.32361
O	+7.99760	+2.37875	+1.08271
H	+7.54453	+2.86300	+0.34988
H	+7.96091	+4.06337	+2.34601
H	+8.20094	+2.45487	+3.12900
H	+5.76579	+1.84829	+2.42200
H	+4.73484	+3.32563	+5.67687
C	+3.83779	+4.18150	+3.85351
O	+2.49067	+4.12413	+4.36939
H	+1.89374	+4.63184	+3.75658
H	+4.27496	+5.19767	+4.07247
C	+3.84896	+3.94537	+2.34532
O	+3.13116	+5.03877	+1.72536

H	+3.35654	+5.04376	+0.74684
H	+3.33662	+2.97060	+2.10780
C	+5.29160	+3.89652	+1.82642
H	+5.75179	+4.91892	+1.87619
O	+5.22155	+3.45943	+0.41426
C	+5.79138	+4.38774	−0.56136
O	+6.91980	+3.73940	−1.21577
C	+6.55768	+2.47116	−1.91084
C	+7.88951	+1.97783	−2.48455
O	+7.81575	+0.61770	−2.91157
H	+7.19646	+0.56143	−3.67970
H	+8.20516	+2.64251	−3.33580
H	+8.67005	+2.04518	−1.69664
H	+6.15247	+1.74548	−1.15402
H	+6.20000	+5.29707	−0.04381
C	+4.74660	+4.74793	−1.61115
O	+3.63362	+5.38328	−0.94438
H	+2.85641	+5.38637	−1.56464
H	+5.20242	+5.47420	−2.34360
C	+4.28490	+3.50082	−2.36070
O	+3.37477	+3.92706	−3.40375
H	+3.28639	+3.17732	−4.06448
H	+3.75132	+2.80658	−1.65234
C	+5.48868	+2.77232	−2.97111
H	+5.92220	+3.38248	−3.80811
O	+4.98104	+1.49073	−3.51639
C	+5.18583	+1.29524	−4.94658
O	+6.06855	+0.14530	−5.13214
H	+5.71380	+2.17890	−5.39535
C	+3.85980	+1.00902	−5.64283
O	+2.99285	+2.14680	−5.44949
H	+2.06621	+1.88135	−5.69488
H	+4.05374	+0.87489	−6.74576
C	+3.21144	−0.26054	−5.09302
O	+2.03474	−0.52780	−5.89246
H	+1.74634	−1.47210	−5.71408
H	+2.91368	−0.10414	−4.01797
C	+4.19267	−1.43742	−5.18264
H	+4.35510	−1.72052	−6.25633
C	+5.53150	−1.09380	−4.51480

H	+5.37232	−0.90446	−3.42232
C	+6.63617	−2.13206	−4.73037
H	+6.70610	−2.38753	−5.82217
H	+7.60940	−1.69494	−4.42064
O	+6.41468	−3.30823	−3.95237
H	+5.63522	−3.78851	−4.32526

345

* E = 6.089 kcal/mol

C	−0.16053	+0.46339	+1.04500
C	−0.80657	−0.58420	+0.51907
C	−0.15171	−1.74157	−0.08955
C	+1.09930	−1.94031	−0.53072
C	+2.26417	−1.06062	−0.62773
C	+2.32226	+0.12863	−1.24252
C	+1.17445	+0.81280	−1.89128
H	+1.46047	+1.18134	−2.87745
C	+3.66070	+0.80548	−1.27041
C	+4.29446	+0.78168	+0.11385
C	+4.37539	−0.63364	+0.69343
C	+3.56576	−1.62625	−0.12931
C	−0.82023	+1.63015	+1.70569
C	−2.31266	+1.44423	+1.95509
C	−2.94642	+0.73455	+0.77681
C	−2.30243	−0.64472	+0.66403
C	−4.41467	+0.33441	+0.93223
C	−4.57576	−0.79339	−0.10451
C	−3.16342	−1.34751	−0.37785
O	+5.68570	−1.14930	+0.69682
H	+6.22205	−0.72782	+1.37346
C	−2.75395	+1.55595	−0.49448
C	−5.43303	+1.44346	+0.79473
H	+3.98331	−0.62315	+1.71440
H	+3.41330	−2.53329	+0.45134
H	+4.20020	−1.89913	−0.97678
H	+3.70046	+1.41478	+0.77403
H	+5.29707	+1.20423	+0.08161
H	+4.31815	+0.29198	−1.97704
H	+3.56536	+1.83074	−1.62624
H	+0.86134	+1.68170	−1.30597
H	+0.31332	+0.15932	−1.99258

H	+1.31607	−2.95938	−0.83920
H	−0.79827	−2.60795	−0.16369
H	+0.91786	+0.49603	+0.98342
H	−0.65682	+2.51473	+1.08034
H	−0.30419	+1.85263	+2.64158
H	−2.46364	+0.83666	+2.85002
H	−2.78223	+2.41112	+2.14281
H	−4.51240	−0.09899	+1.93125
H	−5.02030	−0.40059	−1.01936
H	−5.25090	−1.56465	+0.25823
H	−2.82674	−1.09573	−1.38394
H	−3.12444	−2.43198	−0.29329
H	−3.32564	+1.15270	−1.32850
H	−1.70916	+1.58609	−0.79748
H	−3.09110	+2.57936	−0.33205
H	−6.43768	+1.06790	+0.97217
H	−5.41722	+1.87321	−0.20614
H	−5.25029	+2.24603	+1.50830
H	−2.50649	−1.14798	+1.61736
O	−6.97244	−4.27469	+0.30418
H	−6.55382	−4.60379	−0.52889
C	−6.49780	−5.06599	+1.39271
H	−6.63992	−6.15966	+1.17841
H	−7.10475	−4.81457	+2.28894
C	−5.02235	−4.78899	+1.69607
H	−4.83741	−3.68923	+1.79918
O	−4.79847	−5.43062	+3.01718
C	−3.44754	−5.21979	+3.53267
O	−3.22177	−3.78385	+3.65405
H	−3.41293	−5.71110	+4.54207
C	−2.44792	−5.84211	+2.56408
O	−1.12004	−5.61916	+3.08222
H	−0.46121	−5.81753	+2.36320
H	−2.64299	−6.95203	+2.51149
C	−2.59025	−5.24693	+1.16429
O	−1.69869	−5.97795	+0.28927
H	−1.96388	−5.78416	−0.65901
H	−2.30341	−4.15762	+1.17563
C	−4.03955	−5.38593	+0.67953
H	−4.28025	−6.46588	+0.49178

O	−4.15044	−4.63267	−0.58925
C	−4.53345	−5.42286	−1.75469
O	−5.82549	−4.93759	−2.23373
C	−5.80512	−3.49093	−2.57250
C	−7.24848	−3.17976	−2.97957
O	−7.49675	−1.77492	−3.01750
H	−6.98223	−1.38376	−3.76598
H	−7.46933	−3.64822	−3.97717
H	−7.94028	−3.62849	−2.23463
H	−5.51962	−2.91722	−1.65427
H	−4.68117	−6.49937	−1.47103
C	−3.50571	−5.26100	−2.86901
O	−2.23733	−5.75207	−2.38469
H	−1.51867	−5.43043	−2.99259
H	−3.83040	−5.88191	−3.75264
C	−3.39037	−3.79974	−3.29793
O	−2.50294	−3.74869	−4.44080
H	−2.59813	−2.84382	−4.86386
H	−2.96098	−3.18758	−2.45699
C	−4.76965	−3.24296	−3.67763
H	−5.11337	−3.69544	−4.64551
O	−4.61485	−1.78077	−3.84802
C	−4.92864	−1.26880	−5.17580
O	−6.07211	−0.36545	−5.05980
C	−5.82077	+0.76602	−4.12990
C	−7.15245	+1.52134	−4.09056
O	−7.19950	+2.45526	−3.01256
H	−6.57738	+3.19709	−3.21364
H	−7.32110	+2.04116	−5.07232
H	−7.97803	+0.79151	−3.94729
H	−5.57129	+0.34725	−3.12065
H	−5.24889	−2.10261	−5.85664
C	−3.74348	−0.49359	−5.74011
O	−2.62387	−1.39814	−5.84399
H	−1.79578	−0.86430	−5.98357
H	−4.01387	−0.11943	−6.76947
C	−3.39714	+0.70056	−4.85183
O	−2.34796	+1.44567	−5.51423
H	−2.31090	+2.36193	−5.10717
H	−3.03485	+0.34209	−3.84761

C	−4.63576	+1.58557	−4.65926
H	−4.90778	+2.08069	−5.62911
O	−4.28368	+2.62272	−3.66385
C	−4.39787	+3.99941	−4.13239
O	−5.42340	+4.67204	−3.33828
C	−5.12624	+4.66099	−1.88281
C	−6.34640	+5.33454	−1.24752
O	−6.38818	+5.13185	+0.16433
H	−5.65143	+5.64632	+0.57688
H	−6.34091	+6.43179	−1.49003
H	−7.26978	+4.89606	−1.68332
H	−5.04042	+3.59564	−1.54599
H	−4.75225	+4.02578	−5.19780
C	−3.07567	+4.73792	−3.95466
O	−2.07384	+4.05751	−4.74023
H	−1.17534	+4.36945	−4.44947
H	−3.19668	+5.79089	−4.34051
C	−2.66656	+4.78892	−2.48373
O	−1.48142	+5.61594	−2.38870
H	−1.35071	+5.85798	−1.42399
H	−2.43519	+3.75136	−2.11539
C	−3.79792	+5.38961	−1.63778
H	−3.90585	+6.48372	−1.86348
O	−3.42042	+5.21900	−0.21599
C	−3.28779	+6.45562	+0.54607
O	−4.30246	+6.46147	+1.59769
C	−4.19033	+5.30277	+2.52079
C	−5.37639	+5.47062	+3.47537
O	−5.61639	+4.28705	+4.23516
H	−4.87678	+4.17294	+4.88148
H	−5.19309	+6.34690	+4.15441
H	−6.29049	+5.68730	+2.88184
H	−4.29175	+4.36016	+1.92284
H	−3.50078	+7.34531	−0.10541
C	−1.90882	+6.54058	+1.19108
O	−0.92185	+6.52310	+0.13801
H	−0.03065	+6.33796	+0.53904
H	−1.83301	+7.51519	+1.75390
C	−1.68518	+5.38374	+2.16349
O	−0.41405	+5.60290	+2.82129

H	−0.37755	+5.01114	+3.63092
H	−1.65782	+4.40991	+1.59864
C	−2.81493	+5.33708	+3.20114
H	−2.74117	+6.21950	+3.89063
O	−2.63916	+4.09112	+3.98090
C	−2.43568	+4.27076	+5.41464
O	−3.56049	+3.65984	+6.11907
C	−3.72322	+2.21723	+5.80209
C	−4.98185	+1.80284	+6.57009
O	−5.47018	+0.53283	+6.14058
H	−4.82157	−0.16296	+6.41076
H	−4.76371	+1.79147	+7.67267
H	−5.78059	+2.55423	+6.39090
H	−3.87850	+2.11356	+4.69797
H	−2.44656	+5.36182	+5.68084
C	−1.14767	+3.58742	+5.86138
O	−0.04746	+4.19469	+5.14876
H	+0.74499	+3.59967	+5.22718
H	−1.01263	+3.76007	+6.96751
C	−1.19821	+2.08534	+5.58976
O	−0.00848	+1.49461	+6.16712
H	−0.13791	+0.49938	+6.18336
H	−1.21107	+1.90075	+4.48037
C	−2.45301	+1.46248	+6.21659
H	−2.35658	+1.44078	+7.33444
O	−2.54818	+0.07649	+5.70492
C	−2.48073	−0.97071	+6.71734
O	−3.75460	−1.68752	+6.71990
H	−2.36483	−0.52340	+7.74108
C	−1.36251	−1.95568	+6.39548
O	−0.11161	−1.23544	+6.39054
H	+0.57819	−1.81032	+5.96157
H	−1.33305	−2.74165	+7.20423
C	−1.59817	−2.63713	+5.04841
O	−0.56520	−3.63746	+4.88137
H	−0.85806	−4.26751	+4.15759
H	−1.53427	−1.88072	+4.21724
C	−2.98309	−3.29752	+5.03148
H	−3.00112	−4.16805	+5.73969
C	−4.08014	−2.28955	+5.40054

H	−4.11465	−1.47095	+4.63734
C	−5.46850	−2.90452	+5.59983
H	−5.39021	−3.80665	+6.26526
H	−6.12278	−2.16416	+6.10835
O	−6.07801	−3.24815	+4.35607
H	−5.58677	−4.01303	+3.96682
O	+5.86389	−5.13734	−2.83376
H	+5.31442	−5.68812	−2.22415
C	+5.27047	−5.16103	−4.13036
H	+5.12089	−6.21933	−4.47892
H	+5.97304	−4.66871	−4.83675
C	+3.93116	−4.41812	−4.16084
H	+4.02921	−3.39975	−3.70648
O	+3.64100	−4.26072	−5.61013
C	+2.41060	−3.51743	−5.87158
O	+2.53265	−2.19707	−5.26429
H	+2.32266	−3.43154	−6.98813
C	+1.23806	−4.28030	−5.26586
O	+0.02960	−3.53823	−5.53354
H	−0.69914	−3.90256	−4.96250
H	+1.16987	−5.28957	−5.76517
C	+1.42956	−4.48067	−3.76368
O	+0.33723	−5.30771	−3.29465
H	+0.58165	−5.66090	−2.38766
H	+1.40827	−3.48725	−3.23502
C	+2.77246	−5.17313	−3.49490
H	+2.73674	−6.23378	−3.86048
O	+2.98428	−5.16818	−2.03004
C	+3.09359	−6.48437	−1.41030
O	+4.42925	−6.61353	−0.83353
C	+4.73986	−5.55872	+0.16728
C	+6.17537	−5.87483	+0.59963
O	+6.78315	−4.78285	+1.28595
H	+6.28265	−4.61297	+2.12126
H	+6.17803	−6.79904	+1.24195
H	+6.78598	−6.09118	−0.30323
H	+4.68441	−4.56170	−0.33857
H	+3.00029	−7.29423	−2.18285
C	+2.06405	−6.63520	−0.29640
O	+0.74785	−6.52381	−0.87896

H	+0.09063	−6.36905	−0.14870
H	+2.17869	−7.65715	+0.16665
C	+2.27184	−5.57039	+0.77750
O	+1.34162	−5.84211	+1.85351
H	+1.63805	−5.31856	+2.65670
H	+2.06847	−4.54924	+0.34792
C	+3.71130	−5.61916	+1.30582
H	+3.86285	−6.54456	+1.92283
O	+3.88682	−4.42559	+2.16293
C	+4.25693	−4.69186	+3.54647
O	+5.57213	−4.09812	+3.78626
C	+5.58297	−2.64319	+3.48579
C	+7.04680	−2.22265	+3.62131
O	+7.24286	−0.88512	+3.14116
H	+6.89136	−0.26073	+3.82411
H	+7.37699	−2.30257	+4.68952
H	+7.68066	−2.90139	+3.01267
H	+5.23743	−2.49802	+2.42980
H	+4.36455	−5.79547	+3.72405
C	+3.25030	−4.05401	+4.49916
O	+1.95244	−4.63064	+4.23862
H	+1.26225	−4.05126	+4.65939
H	+3.55563	−4.29387	+5.55831
C	+3.20783	−2.53573	+4.32695
O	+2.34587	−2.00649	+5.36235
H	+2.50805	−1.01938	+5.42666
H	+2.79178	−2.27431	+3.31394
C	+4.62039	−1.95089	+4.46037
H	+4.98671	−2.06422	+5.51508
O	+4.53691	−0.50893	+4.12808
C	+4.91121	+0.39801	+5.20735
O	+6.10815	+1.13327	+4.79665
C	+5.90786	+1.92506	+3.55590
C	+7.27824	+2.55385	+3.28464
O	+7.36986	+3.06342	+1.95510
H	+6.79763	+3.86722	+1.88599
H	+7.47783	+3.36529	+4.03510
H	+8.06407	+1.77748	+3.40862
H	+5.62418	+1.22272	+2.72950
H	+5.19598	−0.17955	+6.12722

C	+3.79384	+1.39702	+5.48681
O	+2.62210	+0.66285	+5.89614
H	+1.84106	+1.27954	+5.88319
H	+4.12049	+2.07288	+6.32898
C	+3.49525	+2.24799	+4.25393
O	+2.50726	+3.23485	+4.63376
H	+2.49699	+3.95078	+3.93053
H	+3.08923	+1.59988	+3.42740
C	+4.77788	+2.93930	+3.77399
H	+5.09205	+3.72360	+4.51249
O	+4.47232	+3.58068	+2.47690
C	+4.64963	+5.02820	+2.43895
O	+5.70098	+5.33586	+1.47257
C	+5.40138	+4.82239	+0.11050
C	+6.63890	+5.19592	−0.71140
O	+6.68022	+4.49677	−1.95416
H	+5.95501	+4.83849	−2.53313
H	+6.65865	+6.30684	−0.88025
H	+7.55052	+4.92495	−0.13641
H	+5.28482	+3.70988	+0.16978
H	+5.00733	+5.40945	+3.43279
C	+3.35827	+5.70543	+1.99578
O	+2.33535	+5.39808	+2.96677
H	+1.44879	+5.63230	+2.58050
H	+3.52292	+6.82078	+1.97133
C	+2.94690	+5.22850	+0.60422
O	+1.78543	+5.99455	+0.20623
H	+1.66394	+5.88390	−0.78395
H	+2.68892	+4.13307	+0.63511
C	+4.09447	+5.45252	−0.38988
H	+4.23493	+6.55101	−0.57230
O	+3.70932	+4.79115	−1.65576
C	+3.61715	+5.66883	−2.81702
O	+4.61929	+5.24509	−3.79202
C	+4.45569	+3.82689	−4.20474
C	+5.63370	+3.57107	−5.14965
O	+5.83050	+2.17769	−5.38433
H	+5.07589	+1.83985	−5.92698
H	+5.46664	+4.11675	−6.11783
H	+6.56111	+3.97462	−4.68924

H	+4.53112	+3.18228	−3.29165
H	+3.87138	+6.72590	−2.53565
C	+2.23602	+5.56168	−3.45307
O	+1.25874	+5.98676	−2.47914
H	+0.35618	+5.70815	−2.79188
H	+2.19353	+6.25045	−4.34503
C	+1.95767	+4.13115	−3.91110
O	+0.68765	+4.13501	−4.60520
H	+0.62052	+3.28584	−5.13640
H	+1.90173	+3.44706	−3.01807
C	+3.07368	+3.65028	−4.84843
H	+3.02274	+4.20597	−5.82221
O	+2.84677	+2.20884	−5.09500
C	+2.64466	+1.84056	−6.49257
O	+3.73438	+0.95711	−6.89912
H	+2.70765	+2.74752	−7.15209
C	+1.32100	+1.10395	−6.66208
O	+0.25877	+1.98924	−6.24689
H	−0.56787	+1.45203	−6.11349
H	+1.18615	+0.85071	−7.75295
C	+1.30115	−0.18618	−5.84439
O	+0.06848	−0.87776	−6.15611
H	+0.15622	−1.82631	−5.84037
H	+1.33517	+0.05531	−4.74522
C	+2.50782	−1.06052	−6.21134
H	+2.39424	−1.45155	−7.25735
C	+3.82045	−0.27702	−6.07526
H	+3.97427	+0.02087	−5.00706
C	+5.05270	−1.00507	−6.62063
H	+4.83164	−1.40071	−7.64915
H	+5.89117	−0.28121	−6.70957
O	+5.47214	−2.05882	−5.75540
H	+4.79246	−2.77638	−5.78077

345

* E = 6.269 kcal/mol

C	−0.55775	−1.03140	−0.09373
C	−0.80261	+0.01585	−0.88957
C	+0.23489	+0.80592	−1.55429
C	+1.54374	+0.95620	−1.30427
C	+2.40085	+0.42482	−0.24316

C	+2.19364	+0.52214	+1.07623
C	+1.02030	+1.17498	+1.71166
H	+1.34242	+1.84117	+2.51327
C	+3.25211	−0.03619	+1.98211
C	+3.76092	−1.41133	+1.54690
C	+3.75899	−1.60163	+0.03162
C	+3.67097	−0.25923	−0.67776
C	−1.60516	−1.87109	+0.56106
C	−3.01937	−1.60715	+0.05834
C	−3.21825	−0.12135	−0.15789
C	−2.23249	+0.33186	−1.23214
C	−4.53676	+0.30349	−0.80648
C	−4.20556	+1.69538	−1.37871
C	−2.67212	+1.76038	−1.52410
O	+4.84107	−2.38397	−0.40871
H	+5.66303	−1.98928	−0.11972
C	−3.01020	+0.63025	+1.15357
C	−5.76087	+0.28074	+0.08082
H	+2.88164	−2.17675	−0.26287
H	+3.69220	−0.42013	−1.75312
H	+4.54818	+0.34304	−0.41865
H	+3.16869	−2.19850	+2.01083
H	+4.77941	−1.54723	+1.90923
H	+4.08905	+0.66721	+1.99743
H	+2.88768	−0.08301	+3.00741
H	+0.36736	+0.42512	+2.16533
H	+0.42723	+1.73816	+0.99786
H	+2.08991	+1.53772	−2.04139
H	−0.12537	+1.34341	−2.42386
H	+0.46783	−1.29661	+0.12038
H	−1.55606	−1.69493	+1.64134
H	−1.34864	−2.92496	+0.43996
H	−3.17415	−2.12356	−0.89145
H	−3.74662	−2.01166	+0.76400
H	−4.70565	−0.38169	−1.64150
H	−4.56305	+2.47301	−0.70322
H	−4.70966	+1.85741	−2.32840
H	−2.23446	+2.44858	−0.80028
H	−2.36647	+2.10252	−2.51106
H	−3.30170	+1.67595	+1.07320

H	−1.96939	+0.60376	+1.46918
H	−3.61614	+0.18266	+1.94083
H	−6.64995	+0.55018	−0.48381
H	−5.66806	+0.99050	+0.90184
H	−5.92802	−0.70744	+0.50715
H	−2.47715	−0.25195	−2.12830
O	−6.45946	−4.10699	−3.01643
H	−5.72754	−4.21139	−3.67333
C	−6.35315	−5.14814	−2.04614
H	−6.33127	−6.15479	−2.54564
H	−7.25857	−5.10904	−1.40316
C	−5.10854	−4.98126	−1.16936
H	−5.03711	−3.93487	−0.77677
O	−5.34047	−5.89671	−0.02223
C	−4.28174	−5.81992	+0.98208
O	−4.18946	−4.43940	+1.44561
H	−4.58899	−6.49836	+1.82278
C	−2.96748	−6.25967	+0.34575
O	−1.93099	−6.16299	+1.34737
H	−1.04666	−6.20345	+0.89357
H	−3.06755	−7.33427	+0.01888
C	−2.62340	−5.39660	−0.86683
O	−1.45286	−5.97250	−1.49437
H	−1.35300	−5.55442	−2.40129
H	−2.39586	−4.34662	−0.53288
C	−3.79278	−5.36305	−1.86036
H	−3.89282	−6.35594	−2.37355
O	−3.47642	−4.32132	−2.86378
C	−3.37245	−4.78976	−4.24055
O	−4.41915	−4.13774	−5.02544
C	−4.32463	−2.65502	−4.99551
C	−5.54486	−2.18585	−5.79296
O	−5.78240	−0.78884	−5.62342
H	−5.05743	−0.29139	−6.07568
H	−5.40363	−2.43424	−6.87961
H	−6.44373	−2.73197	−5.43447
H	−4.39246	−2.31741	−3.92994
H	−3.57211	−5.89348	−4.29847
C	−2.01459	−4.42400	−4.82915
O	−0.99241	−5.06675	−4.03881

H	−0.11501	−4.65079	−4.25839
H	−1.96460	−4.81392	−5.88666
C	−1.81162	−2.90969	−4.84833
O	−0.56439	−2.64906	−5.53414
H	−0.54456	−1.67802	−5.78409
H	−1.75845	−2.51404	−3.79582
C	−2.97335	−2.23384	−5.58905
H	−2.93243	−2.49025	−6.68093
O	−2.80995	−0.77107	−5.42534
C	−2.64558	−0.02224	−6.66635
O	−3.78259	+0.88456	−6.81124
C	−3.90836	+1.84547	−5.68532
C	−5.18585	+2.62749	−6.00513
O	−5.63144	+3.39466	−4.88744
H	−4.99810	+4.14055	−4.74624
H	−5.00952	+3.29033	−6.89494
H	−5.99183	+1.91025	−6.27114
H	−4.02494	+1.26579	−4.73404
H	−2.67757	−0.71372	−7.55081
C	−1.36144	+0.79974	−6.63675
O	−0.24957	−0.10650	−6.48581
H	+0.55954	+0.41890	−6.24235
H	−1.26344	+1.34526	−7.61950
C	−1.38433	+1.82323	−5.50283
O	−0.19126	+2.63408	−5.62466
H	−0.31607	+3.45831	−5.06599
H	−1.38587	+1.29721	−4.50650
C	−2.63696	+2.70249	−5.61511
H	−2.56211	+3.36629	−6.51686
O	−2.69469	+3.53886	−4.39582
C	−2.64476	+4.97984	−4.61942
O	−3.90516	+5.55956	−4.16113
C	−4.18698	+5.25968	−2.73326
C	−5.56129	+5.88633	−2.47938
O	−6.14772	+5.40271	−1.27142
H	−5.62103	+5.74249	−0.50642
H	−5.46815	+7.00624	−2.45408
H	−6.24099	+5.62230	−3.31791
H	−4.22972	+4.14795	−2.60821
H	−2.56736	+5.20736	−5.71640

C	−1.50263	+5.60159	−3.82355
O	−0.26397	+5.02753	−4.29357
H	+0.45019	+5.22807	−3.63079
H	−1.49203	+6.71296	−4.01434
C	−1.68018	+5.35034	−2.32750
O	−0.64115	+6.08227	−1.63313
H	−0.88601	+6.11399	−0.66062
H	−1.57819	+4.25092	−2.11061
C	−3.06106	+5.83294	−1.86230
H	−3.09639	+6.95473	−1.86382
O	−3.24723	+5.33877	−0.47939
C	−3.44405	+6.36868	+0.53252
O	−4.77579	+6.19488	+1.10952
C	−4.97104	+4.85063	+1.71189
C	−6.43437	+4.85028	+2.16345
O	−6.88780	+3.53579	+2.48471
H	−6.44497	+3.24787	+3.32063
H	−6.55863	+5.53792	+3.04322
H	−7.06792	+5.23868	+1.33733
H	−4.80003	+4.07784	+0.91823
H	−3.42917	+7.39037	+0.06642
C	−2.40529	+6.23053	+1.63978
O	−1.09782	+6.39788	+1.05190
H	−0.41437	+6.09023	+1.70643
H	−2.57652	+7.04645	+2.39969
C	−2.51969	+4.87324	+2.33264
O	−1.58625	+4.86958	+3.43898
H	−1.84579	+4.12847	+4.06354
H	−2.25893	+4.04886	+1.61108
C	−3.95149	+4.66662	+2.84450
H	−4.16666	+5.38319	+3.68115
O	−4.04109	+3.28011	+3.35443
C	−4.40540	+3.15752	+4.76186
O	−5.68657	+2.46073	+4.84758
C	−5.65562	+1.11055	+4.22839
C	−7.09491	+0.60487	+4.36577
O	−7.33188	−0.54570	+3.55589
H	−6.82813	−1.30652	+3.93678
H	−7.31489	+0.38169	+5.44489
H	−7.79253	+1.40466	+4.03634

H	−5.37465	+1.22109	+3.14922
H	−4.56424	+4.17158	+5.21781
C	−3.35543	+2.35240	+5.51971
O	−2.09632	+3.05113	+5.41899
H	−1.36704	+2.43339	+5.69379
H	−3.66036	+2.29379	+6.60405
C	−3.23489	+0.93691	+4.95818
O	−2.31828	+0.20682	+5.80901
H	−2.42031	−0.77068	+5.60661
H	−2.83021	+0.97441	+3.90883
C	−4.60841	+0.25172	+4.95044
H	−4.93664	+0.03894	+6.00242
O	−4.45742	−1.02589	+4.21763
C	−4.75623	−2.22673	+4.99043
O	−5.91444	−2.88538	+4.39047
H	−5.05239	−1.95698	+6.03967
C	−3.57359	−3.18851	+4.96217
O	−2.43739	−2.51956	+5.55043
H	−1.61470	−3.02725	+5.31710
H	−3.83093	−4.09536	+5.58147
C	−3.26057	−3.62996	+3.53388
O	−2.21254	−4.62592	+3.60237
H	−2.17545	−5.09913	+2.71797
H	−2.90715	−2.74650	+2.93163
C	−4.51229	−4.22391	+2.87398
H	−4.76885	−5.20761	+3.34928
C	−5.70064	−3.25704	+2.96779
H	−5.47445	−2.32546	+2.38712
C	−7.04205	−3.85569	+2.53373
H	−7.18826	−4.85295	+3.03057
H	−7.86283	−3.18627	+2.86997
O	−7.13175	−3.98216	+1.11549
H	−6.50628	−4.68980	+0.82225
O	+6.77229	−4.69401	−1.52821
H	+6.00584	−5.22462	−1.19737
C	+6.61395	−4.50271	−2.93316
H	+6.56912	−5.49320	−3.46577
H	+7.51128	−3.96253	−3.30614
C	+5.36289	−3.68236	−3.26310
H	+5.28144	−2.79087	−2.58899

O	+5.60145	−3.21561	−4.65676
C	+4.52278	−2.38587	−5.18315
O	+4.37423	−1.21973	−4.31693
H	+4.83744	−2.07159	−6.21487
C	+3.23399	−3.19968	−5.20392
O	+2.18150	−2.36634	−5.73611
H	+1.30729	−2.78552	−5.51336
H	+3.37940	−4.08950	−5.88174
C	+2.88262	−3.68753	−3.80151
O	+1.71517	−4.53969	−3.90486
H	+1.64828	−5.07023	−3.05587
H	+2.65354	−2.80916	−3.13664
C	+4.05361	−4.48119	−3.20943
H	+4.16493	−5.46134	−3.74670
O	+3.71246	−4.73164	−1.79535
C	+3.67968	−6.12482	−1.38704
O	+4.72196	−6.33300	−0.38255
C	+4.57186	−5.42411	+0.78640
C	+5.79999	−5.72475	+1.64960
O	+5.96646	−4.75737	+2.68818
H	+5.25226	−4.89811	+3.35669
H	+5.71591	−6.75704	+2.08473
H	+6.70832	−5.69970	+1.01011
H	+4.58996	−4.36282	+0.41803
H	+3.92392	−6.80223	−2.24950
C	+2.32521	−6.44951	−0.76619
O	+1.30859	−6.22506	−1.76766
H	+0.42146	−6.21962	−1.31855
H	+2.31186	−7.53543	−0.46189
C	+2.06980	−5.58145	+0.46588
O	+0.82754	−6.02921	+1.06093
H	+0.78818	−5.68067	+2.00018
H	+1.97825	−4.50086	+0.16179
C	+3.22800	−5.73278	+1.46142
H	+3.23554	−6.77307	+1.88359
O	+3.00105	−4.76592	+2.56197
C	+2.84597	−5.36076	+3.88440
O	+3.95232	−4.91346	+4.73011
C	+4.01939	−3.43686	+4.87247
C	+5.26221	−3.19990	+5.73565

O	+5.66982	−1.83211	+5.71180
H	+4.99771	−1.29530	+6.19935
H	+5.06055	−3.53433	+6.78914
H	+6.10011	−3.81321	+5.33994
H	+4.15334	−2.98886	+3.85474
H	+2.92510	−6.47939	+3.82673
C	+1.53447	−4.91554	+4.52135
O	+0.44899	−5.37045	+3.68752
H	−0.38212	−4.89787	+3.96172
H	+1.44640	−5.39552	+5.53851
C	+1.49453	−3.39733	+4.68219
O	+0.26978	−3.06460	+5.37780
H	+0.35110	−2.12478	+5.71930
H	+1.50528	−2.90277	+3.67024
C	+2.71060	−2.92579	+5.49016
H	+2.62032	−3.26685	+6.55552
O	+2.71719	−1.44586	+5.44945
C	+2.61315	−0.78524	+6.74563
O	+3.84000	−0.02403	+6.97262
C	+4.10084	+0.97672	+5.90624
C	+5.44825	+1.59535	+6.29030
O	+6.01667	+2.33522	+5.21036
H	+5.47352	+3.14901	+5.06593
H	+5.32020	+2.24962	+7.19473
H	+6.15676	+0.78217	+6.55783
H	+4.17669	+0.43767	+4.92723
H	+2.54962	−1.54257	+7.57227
C	+1.43050	+0.17666	+6.75840
O	+0.22620	−0.58838	+6.54123
H	−0.51460	+0.03850	+6.32186
H	+1.37761	+0.67155	+7.77045
C	+1.59143	+1.25063	+5.68409
O	+0.49741	+2.18604	+5.83361
H	+0.72946	+3.02063	+5.32650
H	+1.55239	+0.77880	+4.66144
C	+2.93440	+1.97310	+5.85594
H	+2.91662	+2.60232	+6.78499
O	+3.11156	+2.84781	+4.67534
C	+3.24486	+4.27116	+4.96644
O	+4.57008	+4.70630	+4.53137

C	+4.81162	+4.45632	+3.08678
C	+6.26029	+4.90022	+2.86377
O	+6.76843	+4.43553	+1.61370
H	+6.30543	+4.92018	+0.88649
H	+6.32646	+6.02015	+2.92665
H	+6.89732	+4.47744	+3.67019
H	+4.69904	+3.35882	+2.89486
H	+3.19600	+4.45443	+6.07331
C	+2.19034	+5.06912	+4.20783
O	+0.88907	+4.62206	+4.64584
H	+0.20826	+4.94010	+3.99392
H	+2.31147	+6.16127	+4.46167
C	+2.34490	+4.88564	+2.69922
O	+1.39863	+5.76997	+2.05318
H	+1.65936	+5.85047	+1.08741
H	+2.12076	+3.81835	+2.41921
C	+3.77490	+5.23406	+2.26487
H	+3.94820	+6.33863	+2.36236
O	+3.90637	+4.84102	+0.84454
C	+4.23199	+5.92360	−0.07619
O	+5.53643	+5.64113	−0.67190
C	+5.57128	+4.34144	−1.39139
C	+7.02513	+4.20687	−1.85353
O	+7.32039	+2.87991	−2.28825
H	+6.84671	+2.71740	−3.14116
H	+7.23151	+4.94701	−2.67310
H	+7.69901	+4.44636	−1.00296
H	+5.30846	+3.52938	−0.66676
H	+4.33809	+6.89508	+0.47740
C	+3.19229	+6.01141	−1.18788
O	+1.91044	+6.28746	−0.58473
H	+1.20026	+6.12860	−1.26352
H	+3.46957	+6.86338	−1.87316
C	+3.14218	+4.71709	−1.99820
O	+2.22951	+4.93042	−3.10167
H	+2.38486	+4.20530	−3.77771
H	+2.76945	+3.87243	−1.35424
C	+4.54095	+4.37472	−2.52836
H	+4.84778	+5.11947	−3.30992
O	+4.46048	+3.02944	−3.14017

C	+4.80053	+2.96890	−4.55780
O	+5.99267	+2.13769	−4.70943
H	+5.07264	+3.98859	−4.94198
C	+3.65937	+2.34637	−5.35471
O	+2.48897	+3.17469	−5.18516
H	+1.69221	+2.66346	−5.49020
H	+3.94748	+2.33212	−6.44507
C	+3.38049	+0.91712	−4.89350
O	+2.37876	+0.36162	−5.77806
H	+2.36954	−0.63446	−5.64998
H	+2.99293	+0.92421	−3.83565
C	+4.66584	+0.08133	−4.95594
H	+4.96366	−0.08673	−6.02491
C	+5.80573	+0.76002	−4.18510
H	+5.53763	+0.82819	−3.09964
C	+7.17483	+0.09775	−4.36522
H	+7.36735	−0.08112	−5.45752
H	+7.96278	+0.78644	−3.99086
O	+7.27127	−1.12185	−3.63068
H	+6.68693	−1.79425	−4.06092

345

* E = 6.384 kcal/mol

C	−0.80488	−0.81792	−0.01950
C	−0.98296	+0.29732	−0.74212
C	+0.06176	+1.11451	−1.34763
C	+1.39879	+1.06836	−1.25126
C	+2.29803	+0.20344	−0.47837
C	+2.32568	+0.11611	+0.85895
C	+1.36057	+0.82623	+1.74308
H	+1.88465	+1.31628	+2.56408
C	+3.33632	−0.73017	+1.57734
C	+4.51321	−1.14307	+0.71612
C	+4.01726	−1.63699	−0.61971
C	+3.30104	−0.51772	−1.34210
C	−1.99795	−1.59164	+0.43144
C	−3.10659	−0.72017	+1.04274
C	−3.20498	+0.65059	+0.37213
C	−2.42820	+0.64639	−0.95577
C	−4.59672	+1.04009	−0.13696
C	−4.30666	+2.14712	−1.16035

C	−2.85115	+1.94529	−1.62141
O	+5.02827	−2.20646	−1.41018
H	+5.70811	−1.55849	−1.59555
C	−2.64757	+1.71648	+1.30591
C	−5.61626	+1.42195	+0.91176
H	+3.31071	−2.45159	−0.44987
H	+2.80905	−0.91758	−2.22913
H	+4.04249	+0.20221	−1.70776
H	+5.09882	−1.91831	+1.20366
H	+5.16954	−0.28509	+0.55492
H	+3.68673	−0.18865	+2.45701
H	+2.83238	−1.61943	+1.97063
H	+0.65765	+0.12089	+2.19337
H	+0.77993	+1.56650	+1.20281
H	+1.93195	+1.73984	−1.91940
H	−0.31482	+1.86075	−2.03549
H	+0.18208	−1.19852	+0.19107
H	−1.71081	−2.38030	+1.12375
H	−2.41553	−2.10149	−0.44371
H	−4.05395	−1.25480	+0.96268
H	−2.91561	−0.59316	+2.10825
H	−4.97487	+0.16716	−0.67583
H	−4.42987	+3.12520	−0.69557
H	−5.00715	+2.11096	−1.99044
H	−2.22393	+2.77359	−1.29100
H	−2.75897	+1.89740	−2.70454
H	−2.71619	+2.71669	+0.87989
H	−1.60178	+1.51431	+1.53112
H	−3.19339	+1.72233	+2.24820
H	−6.58059	+1.62559	+0.45273
H	−5.31648	+2.31959	+1.45146
H	−5.76034	+0.62221	+1.63804
H	−2.86813	−0.17007	−1.54300
O	−7.08340	−2.57298	−3.10083
H	−6.41562	−2.70961	−3.81725
C	−7.12843	−3.74818	−2.29239
H	−7.31401	−4.65782	−2.92571
H	−7.98168	−3.64603	−1.58802
C	−5.83673	−3.93476	−1.49120
H	−5.56811	−2.99440	−0.94584

O	−6.17415	−4.97751	−0.48809
C	−5.07910	−5.24013	+0.44326
O	−4.73801	−3.99080	+1.11499
H	−5.46335	−5.99303	+1.18287
C	−3.88303	−5.77129	−0.33952
O	−2.80505	−6.00016	+0.59459
H	−1.95958	−6.11014	+0.08207
H	−4.17224	−6.75038	−0.81855
C	−3.45059	−4.79044	−1.42830
O	−2.42222	−5.43785	−2.21444
H	−2.28496	−4.89513	−3.04741
H	−3.03446	−3.85741	−0.95531
C	−4.63875	−4.41109	−2.32338
H	−4.93152	−5.28338	−2.96573
O	−4.19444	−3.29312	−3.18775
C	−4.21452	−3.56633	−4.62063
O	−5.18473	−2.67062	−5.24713
C	−4.86704	−1.23222	−5.04912
C	−6.03720	−0.50006	−5.71267
O	−6.05320	+0.88738	−5.37943
H	−5.28703	+1.32528	−5.82489
H	−5.98501	−0.63875	−6.82640
H	−6.99096	−0.94797	−5.35977
H	−4.83872	−1.02370	−3.94938
H	−4.57659	−4.61145	−4.81639
C	−2.84219	−3.31724	−5.23627
O	−1.89436	−4.19624	−4.59545
H	−0.97525	−3.87986	−4.80883
H	−2.89300	−3.56669	−6.33556
C	−2.41822	−1.85759	−5.07965
O	−1.17001	−1.69163	−5.79283
H	−1.01661	−0.70979	−5.92877
H	−2.27112	−1.61268	−3.99078
C	−3.49430	−0.93565	−5.66868
H	−3.53595	−1.05689	−6.78366
O	−3.11053	+0.45708	−5.34348
C	−2.87922	+1.32524	−6.49307
O	−3.87267	+2.39735	−6.46869
C	−3.82972	+3.19536	−5.21659
C	−4.97497	+4.20079	−5.36954

O	−5.30472	+4.81985	−4.12658
H	−4.55313	+5.40178	−3.85395
H	−4.69945	+4.97451	−6.13692
H	−5.87883	+3.66803	−5.73563
H	−4.02060	+2.50778	−4.35335
H	−3.04164	+0.76283	−7.45149
C	−1.48843	+1.94743	−6.42736
O	−0.51626	+0.88146	−6.45983
H	+0.36608	+1.24712	−6.18163
H	−1.34664	+2.60600	−7.33208
C	−1.32259	+2.79068	−5.16476
O	−0.03521	+3.44944	−5.24277
H	−0.01996	+4.17803	−4.55292
H	−1.35312	+2.12693	−4.25549
C	−2.44261	+3.83612	−5.06895
H	−2.29336	+4.63112	−5.84698
O	−2.35212	+4.44547	−3.72285
C	−2.11308	+5.88338	−3.69751
O	−3.26310	+6.52837	−3.06682
C	−3.52067	+6.02652	−1.69205
C	−4.79524	+6.75496	−1.25627
O	−5.37588	+6.15438	−0.09888
H	−4.78475	+6.32801	+0.67475
H	−4.56675	+7.83887	−1.06760
H	−5.54090	+6.70385	−2.07853
H	−3.69520	+4.92164	−1.74611
H	−2.04934	+6.29287	−4.74131
C	−0.86501	+6.19979	−2.88102
O	+0.26124	+5.55744	−3.51541
H	+1.02610	+5.55915	−2.87872
H	−0.70450	+7.31625	−2.88407
C	−1.01953	+5.72141	−1.43814
O	+0.14471	+6.16646	−0.70219
H	−0.06394	+6.10034	+0.27680
H	−1.08078	+4.59672	−1.41082
C	−2.29195	+6.31384	−0.81818
H	−2.16804	+7.41954	−0.67066
O	−2.47765	+5.66319	+0.49901
C	−2.49691	+6.56691	+1.64385
O	−3.81293	+6.48243	+2.27327

C	−4.14968	+5.10662	+2.72061
C	−5.58128	+5.22277	+3.25126
O	−6.17441	+3.93975	+3.44986
H	−5.73128	+3.50729	+4.22060
H	−5.58372	+5.81197	+4.20770
H	−6.19910	+5.77492	+2.51094
H	−4.11082	+4.42468	+1.83225
H	−2.37277	+7.63143	+1.30787
C	−1.43658	+6.16692	+2.66448
O	−0.14611	+6.23919	+2.02116
H	+0.52198	+5.77708	+2.59512
H	−1.46547	+6.90267	+3.51891
C	−1.69015	+4.76176	+3.20865
O	−0.72534	+4.52376	+4.26193
H	−1.02444	+3.71638	+4.77697
H	−1.55206	+4.00321	+2.38904
C	−3.11788	+4.65565	+3.76357
H	−3.21452	+5.26707	+4.69970
O	−3.36212	+3.23085	+4.08314
C	−3.64476	+2.93881	+5.48354
O	−5.00635	+2.41612	+5.57956
C	−5.21341	+1.18607	+4.77137
C	−6.70073	+0.86687	+4.94833
O	−7.15136	−0.09212	+3.99233
H	−6.75699	−0.96980	+4.22011
H	−6.88794	+0.50147	+5.99411
H	−7.29045	+1.79757	+4.80371
H	−4.98360	+1.42315	+3.70085
H	−3.62121	+3.88038	+6.09537
C	−2.67550	+1.88889	+6.01601
O	−1.33776	+2.41860	+5.90427
H	−0.68892	+1.67413	+6.02406
H	−2.90972	+1.70171	+7.10354
C	−2.80546	+0.57489	+5.24652
O	−1.94676	−0.39563	+5.89194
H	−2.21763	−1.30882	+5.57609
H	−2.48038	+0.71958	+4.17788
C	−4.26150	+0.09129	+5.27168
H	−4.54329	−0.22880	+6.30980
O	−4.35137	−1.07199	+4.35990

C	−4.78974	−2.31893	+4.97821
O	−6.06414	−2.70791	+4.37859
H	−4.98607	−2.16813	+6.07369
C	−3.77073	−3.42599	+4.72960
O	−2.51726	−3.01809	+5.31985
H	−1.79202	−3.59274	+4.95548
H	−4.12976	−4.36772	+5.23555
C	−3.60158	−3.69155	+3.23487
O	−2.73238	−4.84061	+3.08664
H	−2.80974	−5.16503	+2.14022
H	−3.13142	−2.79582	+2.74339
C	−4.96018	−3.97451	+2.57911
H	−5.34441	−4.97656	+2.90731
C	−5.98191	−2.87660	+2.90471
H	−5.64793	−1.90591	+2.45556
C	−7.41805	−3.20334	+2.48369
H	−7.68197	−4.24379	+2.81669
H	−8.11303	−2.49947	+2.99013
O	−7.60158	−3.06367	+1.07567
H	−7.09733	−3.78167	+0.61902
O	+5.89281	−5.51028	−2.60277
H	+5.05861	−5.97278	−2.34420
C	+5.73349	−4.97824	−3.91603
H	+5.52037	−5.80172	−4.65420
H	+6.69685	−4.50748	−4.21056
C	+4.62156	−3.92541	−3.98765
H	+4.71295	−3.18397	−3.14751
O	+4.89178	−3.23178	−5.28094
C	+3.92136	−2.20305	−5.63313
O	+3.95806	−1.15362	−4.61556
H	+4.24679	−1.79189	−6.62711
C	+2.53045	−2.82149	−5.70657
O	+1.58961	−1.78312	−6.05510
H	+0.67236	−2.10922	−5.85134
H	+2.52748	−3.60863	−6.51484
C	+2.15562	−3.46841	−4.37686
O	+0.86171	−4.09821	−4.54144
H	+0.73653	−4.74866	−3.78797
H	+2.09823	−2.68458	−3.57032
C	+3.20619	−4.51806	−3.99715

H	+3.14434	−5.39301	−4.69887
O	+2.88149	−4.96837	−2.62560
C	+2.61056	−6.39010	−2.47545
O	+3.63185	−6.96110	−1.59870
C	+3.69842	−6.27318	−0.28369
C	+4.87220	−6.94588	+0.43371
O	+5.28736	−6.20577	+1.58165
H	+4.57405	−6.25723	+2.26414
H	+4.58854	−7.99503	+0.72099
H	+5.73523	−7.00697	−0.26316
H	+3.91032	−5.18874	−0.46203
H	+2.69903	−6.92076	−3.46130
C	+1.24548	−6.59884	−1.82941
O	+0.24239	−6.04341	−2.70684
H	−0.61460	−5.98158	−2.20580
H	+1.06313	−7.70538	−1.71144
C	+1.19059	−5.93127	−0.45620
O	−0.08114	−6.27427	+0.14613
H	−0.02386	−6.07106	+1.12656
H	+1.26688	−4.81409	−0.57144
C	+2.34491	−6.43002	+0.42407
H	+2.17492	−7.50264	+0.70761
O	+2.34818	−5.59674	+1.64822
C	+2.16826	−6.32364	+2.89840
O	+3.37262	−6.14889	+3.70857
C	+3.67569	−4.72128	+3.98422
C	+5.00276	−4.75793	+4.74747
O	+5.60091	−3.46442	+4.82893
H	+5.05735	−2.90614	+5.43752
H	+4.83679	−5.18238	+5.77435
H	+5.70919	−5.42861	+4.21305
H	+3.79552	−4.18966	+3.00592
H	+2.07016	−7.42568	+2.70621
C	+0.97594	−5.77004	+3.67057
O	−0.20646	−5.94859	+2.86276
H	−0.94428	−5.40880	+3.25532
H	+0.86211	−6.35602	+4.62762
C	+1.18137	−4.29475	+4.01184
O	+0.06837	−3.88270	+4.84019
H	+0.31691	−3.02095	+5.28982

H	+1.20156	−3.68072	+3.06805
C	+2.50504	−4.10983	+4.76642
H	+2.43329	−4.57423	+5.78553
O	+2.73297	−2.65354	+4.90884
C	+2.82532	−2.17155	+6.28291
O	+4.16552	−1.62882	+6.49318
C	+4.49571	−0.51399	+5.56849
C	+5.94452	−0.15941	+5.91653
O	+6.53283	+0.69096	+4.93341
H	+6.10863	+1.58195	+4.99503
H	+5.98108	+0.32317	+6.93045
H	+6.54398	−1.09385	+5.96402
H	+4.42003	−0.89115	+4.51624
H	+2.71052	−3.02184	+7.00747
C	+1.80241	−1.06934	+6.53378
O	+0.48671	−1.61991	+6.31580
H	−0.16505	−0.87174	+6.24299
H	+1.89028	−0.73427	+7.60735
C	+2.04856	+0.12823	+5.61788
O	+1.11533	+1.16640	+5.99942
H	+1.42943	+2.03087	+5.59841
H	+1.87433	−0.16476	+4.54439
C	+3.49074	+0.62647	+5.78101
H	+3.62687	+1.08206	+6.79751
O	+3.71633	+1.66200	+4.74708
C	+4.05551	+2.98732	+5.25662
O	+5.40839	+3.31837	+4.81596
C	+5.55519	+3.29483	+3.33768
C	+7.03976	+3.58966	+3.10311
O	+7.43441	+3.28958	+1.76488
H	+7.00171	+3.93738	+1.15578
H	+7.25418	+4.66546	+3.34750
H	+7.64732	+2.95948	+3.78757
H	+5.29146	+2.26955	+2.97318
H	+4.07819	+2.98364	+6.37944
C	+3.09086	+4.03433	+4.71068
O	+1.75977	+3.68869	+5.15051
H	+1.10226	+4.21808	+4.62417
H	+3.37215	+5.03988	+5.13671
C	+3.15480	+4.10063	+3.18603

O	+2.31696	+5.20223	+2.76096
H	+2.53042	+5.39894	+1.80014
H	+2.76898	+3.13904	+2.74657
C	+4.59947	+4.32355	+2.71818
H	+4.92992	+5.36672	+2.96732
O	+4.61013	+4.14266	+1.24971
C	+5.03033	+5.30358	+0.47592
O	+6.25590	+4.95458	−0.24123
C	+6.08240	+3.77118	−1.12256
C	+7.47994	+3.51549	−1.69361
O	+7.57108	+2.22798	−2.30442
H	+7.04401	+2.24093	−3.14150
H	+7.74260	+4.32183	−2.43011
H	+8.22186	+3.55543	−0.86742
H	+5.74886	+2.90800	−0.49229
H	+5.28921	+6.16041	+1.15418
C	+3.95808	+5.68082	−0.54033
O	+2.75588	+6.02255	+0.18198
H	+1.99308	+6.02650	−0.45690
H	+4.31067	+6.58186	−1.12006
C	+3.69794	+4.53168	−1.51316
O	+2.77523	+5.01123	−2.52059
H	+2.80468	+4.37502	−3.29588
H	+3.24323	+3.65945	−0.96594
C	+5.01038	+4.08910	−2.17376
H	+5.37553	+4.88533	−2.87527
O	+4.71413	+2.85915	−2.94221
C	+4.98493	+2.93449	−4.37350
O	+6.03277	+1.96475	−4.69098
H	+5.39060	+3.94532	−4.64672
C	+3.73305	+2.58637	−5.17213
O	+2.70602	+3.54362	−4.83449
H	+1.83338	+3.20167	−5.16833
H	+3.97196	+2.67250	−6.27094
C	+3.26123	+1.16318	−4.87471
O	+2.16504	+0.87295	−5.77366
H	+2.01568	−0.12005	−5.76909
H	+2.90771	+1.09212	−3.80720
C	+4.40962	+0.16932	−5.09736
H	+4.65427	+0.10416	−6.19067

C	+5.65230	+0.58422	−4.29981
H	+5.41362	+0.58457	−3.20427
C	+6.89520	−0.26626	−4.57053
H	+7.03543	−0.40256	−5.67540
H	+7.79114	+0.25941	−4.17534
O	+6.79956	−1.53218	−3.91478
H	+6.14069	−2.08401	−4.40501

345

* E = 6.565 kcal/mol

C	−0.63455	−0.81162	−0.26737
C	−0.86207	+0.40083	−0.79280
C	+0.12260	+1.45424	−1.00246
C	+1.39155	+1.60651	−0.58559
C	+2.24589	+0.81474	+0.30027
C	+1.91124	+0.41591	+1.53714
C	+0.62749	+0.77066	+2.19887
H	+0.81574	+1.08310	+3.22673
C	+2.83899	−0.40145	+2.38122
C	+4.00728	−0.98177	+1.61465
C	+4.63482	+0.10933	+0.78191
C	+3.63139	+0.58742	−0.25144
C	−1.73773	−1.81670	−0.25210
C	−3.07699	−1.25316	+0.24800
C	−3.28470	+0.20551	−0.16206
C	−2.27776	+0.59563	−1.25740
C	−4.59929	+0.51112	−0.88638
C	−4.31495	+1.84464	−1.59386
C	−2.78462	+1.94476	−1.73818
O	+4.98738	+1.14277	+1.67908
H	+5.72822	+1.64505	+1.34541
C	−3.11917	+1.10452	+1.05579
C	−5.84651	+0.51819	−0.03190
H	+5.52373	−0.26510	+0.27556
H	+3.58998	−0.13241	−1.07262
H	+3.98724	+1.52105	−0.69520
H	+3.66771	−1.77903	+0.95288
H	+4.74046	−1.40119	+2.29853
H	+3.21053	+0.22765	+3.19376
H	+2.26560	−1.20151	+2.85582
H	−0.04382	−0.09049	+2.24511

H	+0.10436	+1.56669	+1.67903
H	+1.90993	+2.45042	−1.03106
H	−0.22474	+2.23709	−1.66542
H	+0.34539	−1.09255	+0.08628
H	−1.45950	−2.69488	+0.32713
H	−1.88204	−2.16838	−1.27969
H	−3.88415	−1.88098	−0.13202
H	−3.11213	−1.33258	+1.33434
H	−4.71274	−0.26439	−1.64887
H	−4.69945	+2.67281	−0.99917
H	−4.81919	+1.89647	−2.55551
H	−2.38898	+2.74696	−1.11510
H	−2.47487	+2.16174	−2.75836
H	−3.28945	+2.15524	+0.82462
H	−2.11778	+1.00556	+1.46884
H	−3.82664	+0.82223	+1.83375
H	−6.73174	+0.67309	−0.64383
H	−5.81603	+1.31697	+0.70725
H	−5.97179	−0.42640	+0.49721
H	−2.44789	−0.12016	−2.07260
O	−7.33456	−3.79136	−0.16353
H	−6.72383	−4.43816	−0.59549
C	−7.31962	−4.02319	+1.24424
H	−7.53759	−5.10218	+1.47037
H	−8.12799	−3.41180	+1.69980
C	−5.97995	−3.62645	+1.87215
H	−5.67802	−2.59754	+1.54667
O	−6.25906	−3.59550	+3.33082
C	−5.10787	−3.16136	+4.11884
O	−4.70958	−1.83603	+3.65835
H	−5.45004	−3.12189	+5.18781
C	−3.97209	−4.15969	+3.92182
O	−2.84455	−3.70822	+4.70225
H	−2.03152	−4.19783	+4.40471
H	−4.30693	−5.16716	+4.30247
C	−3.59599	−4.28228	+2.44607
O	−2.62052	−5.34641	+2.32881
H	−2.54715	−5.59203	+1.35878
H	−3.14617	−3.31582	+2.08599
C	−4.83504	−4.61231	+1.60184

H	−5.16930	−5.66404	+1.80601
O	−4.43594	−4.49323	+0.18090
C	−4.57306	−5.70956	−0.61232
O	−5.56882	−5.46897	−1.65460
C	−5.21799	−4.32050	−2.52958
C	−6.41456	−4.18876	−3.47631
O	−6.39484	−2.95014	−4.18452
H	−5.63470	−2.96155	−4.81688
H	−6.42550	−5.05470	−4.19269
H	−7.35318	−4.22828	−2.88297
H	−5.11471	−3.40738	−1.88929
H	−4.96824	−6.55034	+0.01877
C	−3.24662	−6.06766	−1.27380
O	−2.27152	−6.28714	−0.23067
H	−1.36392	−6.26445	−0.63589
H	−3.38153	−7.02072	−1.86135
C	−2.78431	−4.95798	−2.21630
O	−1.60239	−5.43293	−2.90491
H	−1.42498	−4.81244	−3.67326
H	−2.53121	−4.03736	−1.62127
C	−3.88450	−4.61376	−3.23006
H	−4.00558	−5.45131	−3.96706
O	−3.45189	−3.39058	−3.94495
C	−3.27395	−3.53957	−5.38496
O	−4.24884	−2.68669	−6.06189
C	−4.10655	−1.24780	−5.71703
C	−5.26007	−0.57075	−6.46319
O	−5.46463	+0.76973	−6.01900
H	−4.70300	+1.32086	−6.32498
H	−5.05979	−0.59529	−7.56852
H	−6.19573	−1.14012	−6.27562
H	−4.22812	−1.13517	−4.60967
H	−3.50087	−4.59291	−5.70230
C	−1.87035	−3.11275	−5.79993
O	−0.92230	−3.95143	−5.10696
H	−0.01961	−3.54044	−5.18646
H	−1.76152	−3.27241	−6.91157
C	−1.62078	−1.63831	−5.48524
O	−0.32079	−1.29502	−6.02144
H	−0.26153	−0.29499	−6.07985

H	−1.62954	−1.47387	−4.37150
C	−2.70825	−0.77414	−6.13698
H	−2.60588	−0.80625	−7.25422
O	−2.50718	+0.61559	−5.66841
C	−2.25192	+1.59716	−6.71747
O	−3.34623	+2.56578	−6.71827
C	−3.50792	+3.25611	−5.41279
C	−4.73049	+4.15696	−5.61194
O	−5.23524	+4.64585	−4.36991
H	−4.57722	+5.27770	−3.98817
H	−4.46478	+5.00918	−6.29485
H	−5.53746	+3.57022	−6.10121
H	−3.70913	+2.48377	−4.62678
H	−2.26086	+1.10627	−7.72760
C	−0.94379	+2.33652	−6.45621
O	+0.12783	+1.36945	−6.45852
H	+0.93007	+1.78563	−6.04308
H	−0.77657	+3.07682	−7.29062
C	−0.99164	+3.08473	−5.12580
O	+0.22277	+3.86581	−5.01489
H	+0.09409	+4.53086	−4.27451
H	−1.04702	+2.34822	−4.27597
C	−2.21457	+4.01087	−5.07588
H	−2.07125	+4.87556	−5.77687
O	−2.31520	+4.51611	−3.68871
C	−2.22026	+5.96284	−3.53520
O	−3.48734	+6.44907	−2.99320
C	−3.83875	+5.81183	−1.69728
C	−5.21398	+6.39122	−1.35350
O	−5.85063	+5.65577	−0.30908
H	−5.35379	+5.81067	+0.53189
H	−5.10820	+7.47312	−1.06837
H	−5.86439	+6.34011	−2.25303
H	−3.90298	+4.70480	−1.85346
H	−2.08729	+6.45915	−4.53394
C	−1.10232	+6.32242	−2.56307
O	+0.14378	+5.83913	−3.10967
H	+0.83344	+5.87265	−2.39313
H	−1.05384	+7.44514	−2.46827
C	−1.35693	+5.71414	−1.18489

O	−0.32823	+6.20715	−0.29398
H	−0.61475	+6.00795	+0.64679
H	−1.30102	+4.59070	−1.24568
C	−2.74294	+6.12604	−0.66956
H	−2.74832	+7.21917	−0.41515
O	−3.00541	+5.33739	+0.55579
C	−3.20838	+6.11999	+1.76942
O	−4.57262	+5.88965	+2.24113
C	−4.84992	+4.45868	+2.52920
C	−6.33296	+4.43214	+2.90989
O	−6.85060	+3.10207	+2.92478
H	−6.45812	+2.62279	+3.69536
H	−6.47534	+4.92327	+3.91028
H	−6.90991	+5.01563	+2.16059
H	−4.66916	+3.86726	+1.59490
H	−3.13034	+7.21838	+1.54793
C	−2.23025	+5.68963	+2.85725
O	−0.89054	+5.90878	+2.36752
H	−0.24990	+5.43425	+2.96335
H	−2.40109	+6.33347	+3.76782
C	−2.43442	+4.22449	+3.24065
O	−1.56117	+3.94519	+4.36079
H	−1.86725	+3.09084	+4.78830
H	−2.16684	+3.55832	+2.37351
C	−3.89802	+3.98261	+3.63498
H	−4.12827	+4.50661	+4.60043
O	−4.07121	+2.52395	+3.81766
C	−4.49246	+2.10526	+5.14935
O	−5.80692	+1.47491	+5.04040
H	−4.62220	+2.99616	+5.82087
C	−3.50935	+1.09386	+5.72850
O	−2.21842	+1.73053	+5.82933
H	−1.52927	+1.03015	+5.98667
H	−3.85963	+0.80156	+6.76026
C	−3.43047	−0.15797	+4.85614
O	−2.57486	−1.10936	+5.53397
H	−2.72259	−2.01012	+5.11772
H	−2.99187	+0.09979	+3.85183
C	−4.83139	−0.75328	+4.66103
H	−5.20354	−1.18440	+5.62819

C	−5.81513	+0.29935	+4.13164
H	−5.49392	+0.63667	+3.11252
C	−7.27913	−0.15056	+4.11584
H	−7.54265	−0.61584	+5.10421
H	−7.92966	+0.73954	+3.97640
O	−7.54218	−1.05834	+3.04659
H	−7.08582	−1.91369	+3.24153
O	+5.60606	−5.82919	+1.83803
H	+4.83026	−6.04326	+2.41251
C	+5.42722	−6.47002	+0.57576
H	+5.22947	−7.56765	+0.71312
H	+6.37339	−6.36568	+0.00231
C	+4.28556	−5.83805	−0.22631
H	+4.39480	−4.72430	−0.26243
O	+4.46854	−6.37195	−1.60086
C	+3.50104	−5.83289	−2.55432
O	+3.63687	−4.38053	−2.57396
H	+3.76498	−6.26771	−3.55562
C	+2.09907	−6.23470	−2.11031
O	+1.15334	−5.70170	−3.06108
H	+0.23960	−5.77514	−2.67408
H	+2.03054	−7.36057	−2.11210
C	+1.80019	−5.71938	−0.70366
O	+0.50539	−6.23857	−0.31717
H	+0.41119	−6.12928	+0.67577
H	+1.77428	−4.59408	−0.70432
C	+2.88083	−6.20299	+0.27302
H	+2.79504	−7.31205	+0.42214
O	+2.64683	−5.51623	+1.56265
C	+2.37453	−6.39225	+2.69652
O	+3.44534	−6.22273	+3.67611
C	+3.58346	−4.81888	+4.14305
C	+4.78514	−4.85547	+5.09222
O	+5.27414	−3.54616	+5.37887
H	+4.60746	−3.07459	+5.93648
H	+4.50188	−5.38926	+6.03976
H	+5.60710	−5.42980	+4.61308
H	+3.79825	−4.17092	+3.25557
H	+2.39987	−7.46930	+2.37936
C	+1.04822	−6.02006	+3.34926

O	+0.00215	−6.19999	+2.37128
H	−0.82820	−5.76599	+2.70660
H	+0.86559	−6.71487	+4.21865
C	+1.07538	−4.57970	+3.85802
O	−0.16481	−4.34445	+4.56643
H	−0.05454	−3.51527	+5.12147
H	+1.15937	−3.86728	+2.99041
C	+2.26935	−4.37544	+4.80047
H	+2.10260	−4.93601	+5.75838
O	+2.35328	−2.92680	+5.08920
C	+2.22864	−2.55935	+6.49579
O	+3.47763	−1.93035	+6.91796
C	+3.83196	−0.74400	+6.09519
C	+5.18119	−0.28473	+6.65649
O	+5.85528	+0.59573	+5.75943
H	+5.33479	+1.43452	+5.68943
H	+5.02724	+0.20714	+7.65611
H	+5.82969	−1.17343	+6.81421
H	+3.93737	−1.07254	+5.03010
H	+2.09863	−3.47416	+7.13410
C	+1.09059	−1.56228	+6.68225
O	−0.13828	−2.20128	+6.27257
H	−0.83239	−1.50059	+6.14406
H	+1.02070	−1.29022	+7.77429
C	+1.34226	−0.29934	+5.86182
O	+0.29489	+0.64925	+6.17734
H	+0.58798	+1.55045	+5.84661
H	+1.31029	−0.54831	+4.76451
C	+2.71225	+0.30029	+6.20670
H	+2.68865	+0.73790	+7.24012
O	+2.96950	+1.37776	+5.22479
C	+3.18915	+2.70341	+5.79519
O	+4.55269	+3.11889	+5.48331
C	+4.81628	+3.20521	+4.02075
C	+6.30890	+3.53234	+3.93497
O	+6.79115	+3.45507	+2.59003
H	+6.44384	+4.23895	+2.09756
H	+6.50015	+4.55403	+4.35907
H	+6.87776	+2.79877	+4.54532
H	+4.60042	+2.21054	+3.55317

H	+3.11926	+2.66178	+6.91599
C	+2.20646	+3.71246	+5.20900
O	+0.86899	+3.23646	+5.47546
H	+0.23081	+3.75003	+4.91131
H	+2.35655	+4.70101	+5.73197
C	+2.42047	+3.91127	+3.70873
O	+1.54735	+4.98883	+3.28879
H	+1.85522	+5.30448	+2.38797
H	+2.15730	+2.97010	+3.15209
C	+3.88555	+4.27286	+3.42966
H	+4.12475	+5.28367	+3.85624
O	+4.05594	+4.31139	+1.96038
C	+4.45424	+5.59508	+1.40542
O	+5.78653	+5.44672	+0.81177
C	+5.83119	+4.37785	−0.21725
C	+7.29910	+4.32094	−0.64917
O	+7.58982	+3.11388	−1.35546
H	+7.14570	+3.15253	−2.23876
H	+7.54670	+5.21702	−1.27926
H	+7.94603	+4.35236	+0.25391
H	+5.52392	+3.41296	+0.26272
H	+4.54980	+6.36903	+2.21356
C	+3.48539	+6.02511	+0.30848
O	+2.17930	+6.18998	+0.90045
H	+1.50277	+6.20559	+0.17188
H	+3.82977	+7.01276	−0.11366
C	+3.44108	+4.98558	−0.80931
O	+2.60189	+5.50545	−1.86929
H	+2.75093	+4.93761	−2.68257
H	+3.00489	+4.02629	−0.41551
C	+4.85114	+4.71197	−1.34815
H	+5.21689	+5.59068	−1.94240
O	+4.75261	+3.52771	−2.23230
C	+5.14257	+3.74751	−3.61940
O	+6.31127	+2.91575	−3.90334
C	+6.05683	+1.47147	−3.66626
C	+7.40745	+0.79956	−3.93023
O	+7.43471	−0.53944	−3.43647
H	+6.84737	−1.09512	−4.00615
H	+7.63231	+0.82043	−5.03060

H	+8.20525	+1.37238	−3.41027
H	+5.75024	+1.33817	−2.59698
H	+5.46289	+4.81223	−3.77696
C	+4.01052	+3.34540	−4.55757
O	+2.86601	+4.17239	−4.25816
H	+2.06821	+3.78029	−4.70584
H	+4.33698	+3.53508	−5.62041
C	+3.66485	+1.86529	−4.39819
O	+2.68066	+1.53802	−5.40741
H	+2.64256	+0.53875	−5.49156
H	+3.23772	+1.67850	−3.37329
C	+4.92302	+1.00771	−4.59035
H	+5.25393	+1.04723	−5.66198
O	+4.56464	−0.38521	−4.24065
C	+4.73446	−1.35881	−5.31379
O	+5.74677	−2.32799	−4.89911
H	+5.13207	−0.86091	−6.23862
C	+3.42649	−2.09490	−5.58153
O	+2.43676	−1.12415	−5.98189
H	+1.53664	−1.54240	−5.91299
H	+3.59146	−2.82501	−6.42561
C	+2.96269	−2.86201	−4.34466
O	+1.79122	−3.62378	−4.72091
H	+1.63284	−4.31958	−4.01578
H	+2.70077	−2.14182	−3.52031
C	+4.07462	−3.80433	−3.86491
H	+4.22381	−4.63162	−4.60847
C	+5.38840	−3.04193	−3.64633
H	+5.25439	−2.28374	−2.83317
C	+6.60111	−3.93364	−3.36381
H	+6.64818	−4.75850	−4.12512
H	+7.52730	−3.32762	−3.46319
O	+6.56662	−4.47045	−2.04211
H	+5.83517	−5.13390	−1.99218

345

* E = 7.021 kcal/mol

C	+0.39700	−0.79024	−0.63001
C	+0.71340	−0.80266	+0.67006
C	−0.25651	−0.68941	+1.75796
C	−1.50920	−0.21053	+1.80983

C	−2.36201	+0.45195	+0.82510
C	−2.04148	+1.49565	+0.04727
C	−0.71578	+2.16341	+0.01032
H	−0.83958	+3.24693	+0.01926
C	−3.13023	+2.03449	−0.83205
C	−3.85355	+0.90900	−1.55933
C	−4.34097	−0.18620	−0.61383
C	−3.80580	+0.02618	+0.79983
C	+1.37544	−0.96224	−1.74704
C	+2.76807	−1.39127	−1.29580
C	+3.14149	−0.66858	−0.01760
C	+2.13930	−1.08164	+1.05709
C	+4.44639	−1.09823	+0.65636
C	+4.25159	−0.63282	+2.11206
C	+2.73194	−0.49788	+2.33330
O	−5.74664	−0.15841	−0.63155
H	−6.11637	−0.86277	−0.09316
C	+3.13853	+0.83979	−0.24949
C	+5.72731	−0.60533	+0.02235
H	−3.98814	−1.15709	−0.97569
H	−3.94456	−0.88103	+1.38511
H	−4.42743	+0.80295	+1.25447
H	−3.17472	+0.48260	−2.29637
H	−4.71794	+1.28846	−2.09782
H	−3.84690	+2.59383	−0.22300
H	−2.72309	+2.74418	−1.55155
H	−0.18038	+1.91195	−0.90908
H	−0.08596	+1.87096	+0.84496
H	−2.01408	−0.37394	+2.75769
H	+0.10295	−1.10572	+2.69219
H	−0.63427	−0.63208	−0.91418
H	+1.43987	−0.01752	−2.29792
H	+0.97448	−1.67727	−2.46795
H	+2.77720	−2.46639	−1.10350
H	+3.49078	−1.20244	−2.09144
H	+4.45574	−2.19155	+0.64217
H	+4.75076	+0.32349	+2.26946
H	+4.70311	−1.33276	+2.81053
H	+2.43862	+0.54597	+2.44911
H	+2.39792	−1.02088	+3.22759

H	+3.54476	+1.38125	+0.60297
H	+2.13283	+1.21502	−0.42688
H	+3.75151	+1.08728	−1.11603
H	+6.59325	−1.00144	+0.54687
H	+5.79398	+0.48136	+0.05627
H	+5.80363	−0.91509	−1.01930
H	+2.23330	−2.17099	+1.14885
O	−6.95629	−2.93302	−3.45543
H	−6.23141	−3.17308	−4.08326
C	−7.02595	−3.92409	−2.43416
H	−7.14957	−4.94843	−2.88124
H	−7.92685	−3.71569	−1.81684
C	−5.78858	−3.90914	−1.52947
H	−5.56157	−2.86953	−1.17878
O	−6.20217	−4.72600	−0.35723
C	−5.16832	−4.87936	+0.66472
O	−4.84557	−3.56125	+1.20433
H	−5.61625	−5.52874	+1.46440
C	−3.93532	−5.52405	+0.04227
O	−2.92522	−5.64621	+1.06505
H	−2.05042	−5.82187	+0.62449
H	−4.21668	−6.55345	−0.32480
C	−3.41639	−4.70303	−1.13560
O	−2.31244	−5.43169	−1.72288
H	−2.13868	−5.05038	−2.63451
H	−3.06301	−3.69536	−0.77938
C	−4.53381	−4.51655	−2.16929
H	−4.77606	−5.49888	−2.65524
O	−4.03073	−3.57422	−3.19211
C	−3.96451	−4.09977	−4.55108
O	−4.88207	−3.32886	−5.38630
C	−4.58593	−1.87237	−5.37143
C	−5.68459	−1.25689	−6.24357
O	−5.76813	+0.15706	−6.07401
H	−4.94948	+0.57039	−6.44334
H	−5.50026	−1.51716	−7.32180
H	−6.66423	−1.69348	−5.95308
H	−4.66515	−1.50702	−4.31646
H	−4.31983	−5.16466	−4.58214
C	−2.55378	−3.94851	−5.10763

O	−1.65982	−4.72941	−4.28647
H	−0.72314	−4.48174	−4.51347
H	−2.53464	−4.34977	−6.16124
C	−2.13273	−2.48024	−5.12130
O	−0.83863	−2.40329	−5.76617
H	−0.67076	−1.44445	−6.01077
H	−2.05504	−2.09512	−4.06642
C	−3.16077	−1.64161	−5.89334
H	−3.10222	−1.87694	−6.98924
O	−2.80999	−0.22040	−5.67840
C	−2.50294	+0.53886	−6.88447
O	−3.48798	+1.61002	−7.01480
C	−3.52206	+2.50605	−5.82983
C	−4.65627	+3.48917	−6.13402
O	−5.04444	+4.22485	−4.97480
H	−4.30965	+4.83961	−4.73067
H	−4.34128	+4.18528	−6.95837
H	−5.54194	+2.92075	−6.49054
H	−3.76259	+1.88911	−4.92708
H	−2.60846	−0.10646	−7.79747
C	−1.11518	+1.16016	−6.77792
O	−0.15190	+0.09147	−6.65825
H	+0.71067	+0.47486	−6.34474
H	−0.90539	+1.74365	−7.71997
C	−1.03330	+2.10158	−5.57756
O	+0.26638	+2.73764	−5.61177
H	+0.23722	+3.53870	−5.00791
H	−1.14565	+1.51622	−4.62257
C	−2.14303	+3.15861	−5.65998
H	−1.93822	+3.86336	−6.50917
O	−2.12608	+3.91135	−4.38559
C	−1.87766	+5.34330	−4.51049
O	−3.05696	+6.05882	−4.02846
C	−3.39436	+5.73738	−2.61725
C	−4.68382	+6.52027	−2.35272
O	−5.32404	+6.09530	−1.15070
H	−4.76893	+6.37674	−0.38243
H	−4.45566	+7.62000	−2.31296
H	−5.38907	+6.35074	−3.19450
H	−3.57485	+4.63471	−2.53887

H	−1.75053	+5.62952	−5.58905
C	−0.67599	+5.75265	−3.66630
O	+0.48116	+5.03776	−4.14923
H	+1.20475	+5.11219	−3.47061
H	−0.50616	+6.86041	−3.79455
C	−0.91833	+5.45349	−2.18808
O	+0.21507	+5.96707	−1.44894
H	−0.04725	+6.03353	−0.48251
H	−1.00433	+4.34132	−2.02995
C	−2.21243	+6.13282	−1.72114
H	−2.08141	+7.24749	−1.71815
O	−2.47977	+5.66866	−0.34090
C	−2.53220	+6.72098	+0.66904
O	−3.88018	+6.75634	+1.23023
C	−4.28649	+5.47280	+1.85958
C	−5.72999	+5.72272	+2.30691
O	−6.38829	+4.51448	+2.68286
H	−5.97889	+4.17910	+3.51756
H	−5.73723	+6.45861	+3.15644
H	−6.29703	+6.17315	+1.46413
H	−4.24229	+4.66669	+1.08289
H	−2.35838	+7.72611	+0.19880
C	−1.53786	+6.44017	+1.79009
O	−0.21433	+6.39541	+1.21611
H	+0.40405	+5.98149	+1.87640
H	−1.58916	+7.28546	+2.53537
C	−1.86281	+5.12861	+2.50238
O	−0.95126	+5.00493	+3.62043
H	−1.30767	+4.29599	+4.23456
H	−1.72036	+4.26275	+1.79752
C	−3.31393	+5.14572	+3.00120
H	−3.42587	+5.88903	+3.83473
O	−3.61754	+3.79007	+3.51088
C	−3.97041	+3.71564	+4.92343
O	−5.34722	+3.23583	+5.02721
C	−5.55029	+1.91949	+4.36849
C	−7.04822	+1.64772	+4.53776
O	−7.50024	+0.60275	+3.67746
H	−7.09515	−0.24919	+3.97454
H	−7.26771	+1.39907	+5.61195

H	−7.61417	+2.56873	+4.28118
H	−5.28431	+2.02024	+3.28524
H	−3.95143	+4.73634	+5.39149
C	−3.05833	+2.73448	+5.65182
O	−1.70388	+3.22432	+5.55935
H	−1.08273	+2.49142	+5.81778
H	−3.36451	+2.69332	+6.73653
C	−3.17195	+1.33858	+5.04354
O	−2.38197	+0.43487	+5.85456
H	−2.64689	−0.50281	+5.61598
H	−2.77509	+1.34805	+3.99063
C	−4.63549	+0.87775	+5.02811
H	−4.98151	+0.65411	+6.07205
O	−4.67537	−0.36362	+4.22288
C	−5.19042	−1.54503	+4.90120
O	−6.41105	−1.96687	+4.21040
H	−5.48354	−1.30237	+5.95748
C	−4.17167	−2.67938	+4.83357
O	−2.96557	−2.23123	+5.49055
H	−2.22291	−2.84390	+5.24139
H	−4.58608	−3.57017	+5.38714
C	−3.87646	−3.08410	+3.38814
O	−3.05066	−4.27179	+3.42998
H	−3.04617	−4.67615	+2.51169
H	−3.32182	−2.25448	+2.86559
C	−5.18285	−3.37366	+2.63464
H	−5.65527	−4.31166	+3.02927
C	−6.15535	−2.19505	+2.76706
H	−5.68819	−1.26812	+2.34840
C	−7.52390	−2.41454	+2.12443
H	−7.95352	−3.39967	+2.43988
H	−8.21878	−1.61269	+2.45269
O	−7.41263	−2.35242	+0.69653
H	−7.06267	−3.22320	+0.38270
O	+5.91014	−5.55861	−1.50526
H	+5.09897	−6.07708	−1.27943
C	+5.91294	−5.30509	−2.90952
H	+5.81523	−6.26514	−3.48581
H	+6.89231	−4.85077	−3.17245
C	+4.79061	−4.34544	−3.31586

H	+4.78948	−3.43577	−2.66277
O	+5.14662	−3.93001	−4.69714
C	+4.21891	−2.94369	−5.24553
O	+4.19634	−1.78998	−4.35230
H	+4.61858	−2.65441	−6.25436
C	+2.83278	−3.57237	−5.33843
O	+1.92860	−2.58337	−5.87794
H	+0.99487	−2.88098	−5.70973
H	+2.88115	−4.45286	−6.04110
C	+2.35427	−4.04748	−3.96807
O	+1.10599	−4.75592	−4.16072
H	+0.89715	−5.24572	−3.30992
H	+2.18567	−3.16059	−3.29732
C	+3.39188	−4.97645	−3.32246
H	+3.40972	−5.96401	−3.85516
O	+2.97143	−5.18393	−1.91807
C	+2.66731	−6.56243	−1.55330
O	+3.62545	−6.98913	−0.53529
C	+3.59613	−6.13534	+0.68097
C	+4.71357	−6.70136	+1.56204
O	+5.03588	−5.82174	+2.63839
H	+4.27861	−5.81412	+3.27412
H	+4.41142	−7.71082	+1.95243
H	+5.62774	−6.83645	+0.94494
H	+3.82341	−5.08124	+0.37899
H	+2.81221	−7.24528	−2.43315
C	+1.25953	−6.66394	−0.97759
O	+0.32414	−6.23590	−1.99016
H	−0.55128	−6.05864	−1.55237
H	+1.05441	−7.74226	−0.71702
C	+1.11850	−5.81220	+0.28277
O	−0.19985	−6.05786	+0.82735
H	−0.20972	−5.73025	+1.77537
H	+1.22794	−4.72179	+0.02571
C	+2.19469	−6.20652	+1.30294
H	+1.99411	−7.24036	+1.69111
O	+2.11481	−5.24108	+2.42229
C	+1.82235	−5.82516	+3.72649
O	+2.96719	−5.58841	+4.60344
C	+3.27689	−4.14543	+4.77466

C	+4.53751	−4.13408	+5.64453
O	+5.16111	−2.85058	+5.65615
H	+4.58917	−2.22840	+6.16945
H	+4.27882	−4.45797	+6.68878
H	+5.26674	−4.86508	+5.23380
H	+3.48603	−3.70524	+3.76646
H	+1.71179	−6.93978	+3.64331
C	+0.58721	−5.17796	+4.34373
O	−0.53187	−5.41268	+3.46413
H	−1.28652	−4.82907	+3.74792
H	+0.38506	−5.67008	+5.33860
C	+0.79767	−3.68098	+4.56534
O	−0.36514	−3.17709	+5.26617
H	−0.13001	−2.28294	+5.65641
H	+0.90252	−3.15377	+3.57557
C	+2.06230	−3.44812	+5.40233
H	+1.90164	−3.81348	+6.45124
O	+2.30845	−1.98935	+5.42548
C	+2.31196	−1.37687	+6.74997
O	+3.64405	−0.83258	+7.00048
C	+4.06379	+0.15452	+5.97222
C	+5.48794	+0.54127	+6.38230
O	+6.17230	+1.22853	+5.33551
H	+5.74877	+2.11318	+5.20940
H	+5.45548	+1.16955	+7.31371
H	+6.06114	−0.38148	+6.61652
H	+4.06240	−0.35257	+4.97384
H	+2.12782	−2.15004	+7.54335
C	+1.29779	−0.24052	+6.81240
O	−0.01298	−0.79566	+6.57077
H	−0.64214	−0.04993	+6.37595
H	+1.32407	+0.21145	+7.84516
C	+1.62461	+0.83964	+5.78328
O	+0.70506	+1.93771	+5.99521
H	+1.04977	+2.73032	+5.48523
H	+1.49264	+0.42950	+4.74348
C	+3.07068	+1.32439	+5.95836
H	+3.16199	+1.92653	+6.90113
O	+3.38313	+2.18449	+4.79551
C	+3.72233	+3.56596	+5.11197

O	+5.10284	+3.80507	+4.69504
C	+5.32056	+3.54609	+3.24807
C	+6.82162	+3.77509	+3.04827
O	+7.27144	+3.27001	+1.79169
H	+6.89989	+3.83948	+1.07371
H	+7.05201	+4.87108	+3.13990
H	+7.37849	+3.24367	+3.84970
H	+5.04943	+2.48007	+3.03350
H	+3.68943	+3.73849	+6.22122
C	+2.80736	+4.52273	+4.35629
O	+1.44986	+4.27635	+4.78007
H	+0.83157	+4.71533	+4.13587
H	+3.09187	+5.58133	+4.62236
C	+2.94782	+4.33280	+2.84686
O	+2.14107	+5.34769	+2.20369
H	+2.43322	+5.41823	+1.24628
H	+2.58093	+3.30990	+2.55179
C	+4.41823	+4.48455	+2.43508
H	+4.74518	+5.55005	+2.56775
O	+4.51291	+4.11930	+1.00416
C	+5.01183	+5.17108	+0.12541
O	+6.27168	+4.72006	−0.46164
C	+6.13237	+3.45513	−1.22831
C	+7.55993	+3.13298	−1.68014
O	+7.67336	+1.79455	−2.16178
H	+7.18453	+1.72735	−3.01861
H	+7.88087	+3.86618	−2.46888
H	+8.24754	+3.24433	−0.81434
H	+5.74600	+2.66148	−0.53792
H	+5.24887	+6.09795	+0.71370
C	+4.01753	+5.44775	−0.99673
O	+2.77811	+5.88798	−0.40205
H	+2.06031	+5.83085	−1.08810
H	+4.42918	+6.27224	−1.64724
C	+3.79346	+4.20224	−1.85215
O	+2.94061	+4.58270	−2.95858
H	+2.99551	+3.86182	−3.65420
H	+3.28942	+3.40292	−1.24105
C	+5.13602	+3.67335	−2.37507
H	+5.56118	+4.38664	−3.13016

O	+4.87213	+2.37099	−3.02797
C	+5.21523	+2.30456	−4.44432
O	+6.27919	+1.31767	−4.61543
H	+5.63318	+3.28606	−4.79540
C	+4.00640	+1.87435	−5.26773
O	+2.96623	+2.85690	−5.08237
H	+2.10850	+2.48149	−5.41934
H	+4.30243	+1.84914	−6.35600
C	+3.52350	+0.48599	−4.85121
O	+2.45854	+0.10466	−5.75435
H	+2.32423	−0.88682	−5.67761
H	+3.13435	+0.51466	−3.79432
C	+4.67833	−0.52042	−4.94039
H	+4.95759	−0.69156	−6.01381
C	+5.89604	−0.03702	−4.14100
H	+5.63120	+0.02862	−3.05404
C	+7.16228	−0.87539	−4.34066
H	+7.33323	−1.04683	−5.43780
H	+8.03485	−0.31195	−3.94567
O	+7.08932	−2.11790	−3.64274
H	+6.42418	−2.69349	−4.09452

345

* E = 7.576 kcal/mol

C	−0.62030	−0.83064	+0.08107
C	−0.94011	+0.06278	−0.86551
C	−0.01094	+0.89349	−1.61907
C	+1.29915	+1.14924	−1.47115
C	+2.29895	+0.75523	−0.47493
C	+2.19928	+0.94198	+0.85020
C	+0.98652	+1.50198	+1.50637
H	+1.26539	+2.22818	+2.26942
C	+3.33681	+0.61184	+1.77282
C	+4.67839	+0.53800	+1.07523
C	+4.58365	−0.32949	−0.15099
C	+3.57262	+0.26097	−1.11512
C	−1.69301	−1.68354	+0.67283
C	−2.96976	−0.91180	+1.04792
C	−3.24656	+0.26079	+0.10603
C	−2.40901	+0.11487	−1.17527
C	−4.65300	+0.30959	−0.50026

C	−4.48297	+1.22028	−1.72523
C	−2.99185	+1.16522	−2.10634
O	+5.86392	−0.39177	−0.72218
H	+5.92086	−1.06659	−1.40066
C	−2.91664	+1.56865	+0.81294
C	−5.77050	+0.72751	+0.42828
H	+4.25077	−1.32827	+0.15309
H	+3.32473	−0.46959	−1.88831
H	+4.05350	+1.09872	−1.63000
H	+5.44499	+0.14866	+1.73953
H	+4.99276	+1.53531	+0.76367
H	+3.37426	+1.36210	+2.56394
H	+3.12169	−0.33399	+2.28060
H	+0.41302	+0.71942	+2.00988
H	+0.32381	+1.97777	+0.79111
H	+1.73911	+1.71742	−2.28673
H	−0.45701	+1.35126	−2.49327
H	+0.40622	−0.98797	+0.37583
H	−1.32158	−2.23597	+1.53363
H	−1.96342	−2.44114	−0.07122
H	−3.80885	−1.60888	+1.04897
H	−2.87659	−0.54212	+2.06891
H	−4.86488	−0.70337	−0.85351
H	−4.77762	+2.23959	−1.47704
H	−5.12564	+0.90524	−2.54301
H	−2.51509	+2.13242	−1.94522
H	−2.83776	+0.91333	−3.15373
H	−3.10502	+2.43928	+0.18604
H	−1.87096	+1.58508	+1.11210
H	−3.51866	+1.67654	+1.71403
H	−6.73232	+0.66384	−0.07456
H	−5.64484	+1.75617	+0.76289
H	−5.81698	+0.08838	+1.31023
H	−2.68317	−0.86542	−1.58691
O	−6.70590	−3.86430	−2.59509
H	−6.00139	−4.11320	−3.24275
C	−6.70020	−4.81558	−1.53140
H	−6.78764	−5.86095	−1.93439
H	−7.59020	−4.62226	−0.89492
C	−5.43675	−4.69981	−0.67371

H	−5.26599	−3.63895	−0.35822
O	−5.74125	−5.50567	+0.53679
C	−4.67446	−5.45417	+1.53408
O	−4.45810	−4.06001	+1.90458
H	−5.03552	−6.04583	+2.41778
C	−3.40655	−6.04857	+0.93073
O	−2.36078	−5.96882	+1.92397
H	−1.48524	−6.11626	+1.47600
H	−3.59864	−7.13145	+0.68113
C	−2.99910	−5.30684	−0.34129
O	−1.88184	−6.02012	−0.92307
H	−1.75377	−5.68092	−1.85882
H	−2.68578	−4.25620	−0.08531
C	−4.16786	−5.25412	−1.33509
H	−4.36166	−6.27599	−1.75645
O	−3.76972	−4.34002	−2.43050
C	−3.70425	−4.94657	−3.75567
O	−4.71256	−4.31452	−4.60385
C	−4.52009	−2.84803	−4.75227
C	−5.71109	−2.40407	−5.60679
O	−5.85029	−0.98427	−5.62762
H	−5.09960	−0.60298	−6.14554
H	−5.59572	−2.80569	−6.64980
H	−6.64279	−2.83332	−5.17960
H	−4.56186	−2.38030	−3.73585
H	−3.97314	−6.03590	−3.70304
C	−2.32942	−4.73169	−4.37854
O	−1.34453	−5.34660	−3.52149
H	−0.44567	−5.00911	−3.78182
H	−2.30989	−5.23830	−5.38646
C	−2.03068	−3.24517	−4.56563
O	−0.76814	−3.14186	−5.26585
H	−0.68655	−2.20841	−5.62463
H	−1.95405	−2.73731	−3.56392
C	−3.14718	−2.58720	−5.38707
H	−3.12607	−2.97144	−6.44139
O	−2.88899	−1.12961	−5.39334
C	−2.66348	−0.54055	−6.70909
O	−3.73971	+0.41154	−6.97747
C	−3.83257	+1.47878	−5.94817

C	−5.04435	+2.31333	−6.37380
O	−5.50226	+3.15671	−5.31741
H	−4.81916	+3.85166	−5.14810
H	−4.78530	+2.92154	−7.28296
H	−5.87637	+1.63027	−6.64964
H	−4.01618	+0.99504	−4.95493
H	−2.72582	−1.32606	−7.50936
C	−1.33343	+0.20471	−6.74479
O	−0.27614	−0.74437	−6.49049
H	+0.55886	−0.24105	−6.29182
H	−1.19620	+0.64466	−7.77418
C	−1.30690	+1.32965	−5.71218
O	−0.07537	+2.06881	−5.89847
H	−0.16171	+2.93951	−5.40658
H	−1.33410	+0.89548	−4.67329
C	−2.51209	+2.26021	−5.90511
H	−2.38400	+2.86513	−6.84177
O	−2.54649	+3.16759	−4.73671
C	−2.44367	+4.58933	−5.04177
O	−3.67516	+5.24089	−4.60064
C	−3.94231	+5.04340	−3.15223
C	−5.29752	+5.71874	−2.92384
O	−5.85974	+5.36006	−1.66190
H	−5.31814	+5.77780	−0.94768
H	−5.18292	+6.83362	−3.00363
H	−6.00320	+5.39282	−3.71792
H	−4.00838	+3.94355	−2.95069
H	−2.37467	+4.75281	−6.15074
C	−1.26614	+5.20754	−4.29674
O	−0.05849	+4.55292	−4.74070
H	+0.67209	+4.77457	−4.10215
H	−1.20956	+6.30386	−4.55579
C	−1.43702	+5.05951	−2.78574
O	−0.35064	+5.77520	−2.15213
H	−0.58837	+5.90818	−1.18673
H	−1.39583	+3.97069	−2.49890
C	−2.78424	+5.64917	−2.34738
H	−2.77082	+6.76533	−2.46450
O	−2.96523	+5.31034	−0.91769
C	−3.09877	+6.45176	−0.01956

O	−4.42822	+6.40603	+0.58565
C	−4.67517	+5.15115	+1.34149
C	−6.12925	+5.27344	+1.80595
O	−6.63425	+4.03054	+2.29303
H	−6.18676	+3.82643	+3.15067
H	−6.20820	+6.06903	+2.59523
H	−6.75950	+5.58557	+0.94565
H	−4.55364	+4.28512	+0.64112
H	−3.04436	+7.41489	−0.59478
C	−2.05203	+6.39162	+1.08768
O	−0.74591	+6.42821	+0.47468
H	−0.06662	+6.17563	+1.15607
H	−2.17602	+7.29781	+1.74789
C	−2.21557	+5.13184	+1.93654
O	−1.27040	+5.22120	+3.02943
H	−1.52904	+4.54006	+3.71913
H	−1.99483	+4.21891	+1.31620
C	−3.64935	+5.04344	+2.47808
H	−3.82387	+5.84868	+3.24013
O	−3.79678	+3.71953	+3.12340
C	−4.11172	+3.74737	+4.54667
O	−5.43069	+3.14732	+4.73836
C	−5.50899	+1.74876	+4.24203
C	−6.97140	+1.35357	+4.46773
O	−7.31326	+0.16420	+3.75713
H	−6.85469	−0.59970	+4.18606
H	−7.16379	+1.22682	+5.56747
H	−7.62787	+2.17177	+4.10101
H	−5.26159	+1.74590	+3.14918
H	−4.18720	+4.80596	+4.91413
C	−3.08523	+2.94186	+5.33550
O	−1.79076	+3.54611	+5.13155
H	−1.08906	+2.90882	+5.43318
H	−3.35006	+2.99461	+6.43080
C	−3.07807	+1.47870	+4.89522
O	−2.17194	+0.76417	+5.76935
H	−2.35700	−0.21734	+5.67376
H	−2.72193	+1.39824	+3.82990
C	−4.49067	+0.88912	+5.00323
H	−4.78665	+0.79456	+6.08170

O	−4.44900	−0.45683	+4.38791
C	−4.81577	−1.55901	+5.27013
O	−6.02700	−2.18526	+4.74524
H	−5.07250	−1.17661	+6.29440
C	−3.70258	−2.60039	+5.31391
O	−2.51516	−1.95795	+5.82632
H	−1.72946	−2.53635	+5.63309
H	−4.00910	−3.42780	+6.01620
C	−3.44402	−3.18918	+3.92795
O	−2.48848	−4.26730	+4.07973
H	−2.49584	−4.80624	+3.23353
H	−3.01574	−2.39771	+3.25326
C	−4.74656	−3.72657	+3.31835
H	−5.06883	−4.65521	+3.85989
C	−5.86309	−2.67420	+3.35219
H	−5.58337	−1.80507	+2.70222
C	−7.24870	−3.20845	+2.97631
H	−7.44798	−4.16545	+3.53078
H	−8.01829	−2.47002	+3.28817
O	−7.37587	−3.40640	+1.56899
H	−6.80065	−4.16622	+1.30454
O	+6.34169	−5.22513	−0.91871
H	+5.60495	−5.81837	−0.63074
C	+6.27880	−5.07548	−2.33541
H	+6.24983	−6.07911	−2.83973
H	+7.20513	−4.55770	−2.66626
C	+5.06371	−4.24871	−2.76882
H	+5.00016	−3.29655	−2.18117
O	+5.34953	−3.90878	−4.18673
C	+4.31756	−3.08049	−4.80986
O	+4.22356	−1.82307	−4.07445
H	+4.66001	−2.89393	−5.86302
C	+2.99026	−3.82923	−4.77255
O	+1.98494	−2.99332	−5.38141
H	+1.08787	−3.35562	−5.14865
H	+3.09775	−4.77766	−5.37440
C	+2.59854	−4.19220	−3.34175
O	+1.39377	−4.99116	−3.40904
H	+1.27082	−5.44283	−2.52154
H	+2.40379	−3.25736	−2.74599

C	+3.72630	−4.99430	−2.67979
H	+3.80928	−6.00695	−3.15629
O	+3.37434	−5.15146	−1.25183
C	+3.21229	−6.52695	−0.79430
O	+4.23713	−6.80000	+0.21082
C	+4.18022	−5.85891	+1.35957
C	+5.35676	−6.27615	+2.24740
O	+5.67481	−5.27384	+3.21155
H	+4.93603	−5.21761	+3.86642
H	+5.12132	−7.25239	+2.75260
H	+6.25349	−6.43292	+1.61005
H	+4.32622	−4.81927	+0.97099
H	+3.38941	−7.24828	−1.63655
C	+1.84022	−6.71365	−0.15750
O	+0.83874	−6.45869	−1.16512
H	−0.04542	−6.36681	−0.71781
H	+1.74683	−7.78026	+0.19639
C	+1.66348	−5.77229	+1.03237
O	+0.39462	−6.08704	+1.65434
H	+0.38395	−5.66723	+2.56573
H	+1.65832	−4.70420	+0.67735
C	+2.80727	−5.96807	+2.03696
H	+2.70139	−6.96050	+2.55033
O	+2.69496	−4.88432	+3.03770
C	+2.51003	−5.32044	+4.41645
O	+3.65685	−4.86435	+5.19858
C	+3.84290	−3.39154	+5.13572
C	+5.12340	−3.13727	+5.93649
O	+5.63600	−1.82493	+5.71020
H	+5.01922	−1.17065	+6.12160
H	+4.92443	−3.30211	+7.03022
H	+5.89939	−3.86672	+5.61954
H	+3.98120	−3.09897	+4.06400
H	+2.50357	−6.44158	+4.48068
C	+1.24323	−4.70482	+4.99897
O	+0.12006	−5.17006	+4.22025
H	−0.67653	−4.61887	+4.44801
H	+1.12574	−5.05568	+6.06421
C	+1.32198	−3.17889	+4.98042
O	+0.14499	−2.67589	+5.65584

H	+0.30663	−1.71272	+5.88681
H	+1.33824	−2.80878	+3.91733
C	+2.59158	−2.70694	+5.70285
H	+2.50264	−2.90827	+6.80339
O	+2.70767	−1.24774	+5.48104
C	+2.70650	−0.43254	+6.69118
O	+3.99054	+0.25776	+6.78452
C	+4.26900	+1.13277	+5.61616
C	+5.67263	+1.68233	+5.88765
O	+6.22857	+2.30787	+4.73243
H	+5.73558	+3.14784	+4.56229
H	+5.63495	+2.40269	+6.74916
H	+6.33996	+0.84227	+6.17716
H	+4.26080	+0.50162	+4.69106
H	+2.62863	−1.08243	+7.60386
C	+1.59092	+0.60491	+6.63549
O	+0.33288	−0.09465	+6.53342
H	−0.37217	+0.55287	+6.26238
H	+1.60571	+1.20117	+7.59300
C	+1.78124	+1.55667	+5.45546
O	+0.75236	+2.57014	+5.54673
H	+1.01704	+3.33789	+4.95716
H	+1.67733	+0.99487	+4.48491
C	+3.17182	+2.20234	+5.52352
H	+3.22955	+2.89620	+6.40368
O	+3.36049	+2.97906	+4.27683
C	+3.57859	+4.41180	+4.46328
O	+4.91830	+4.74313	+3.98731
C	+5.12886	+4.41882	+2.55216
C	+6.59217	+4.79398	+2.29792
O	+7.06738	+4.27487	+1.05767
H	+6.59685	+4.73392	+0.31968
H	+6.70339	+5.91239	+2.32893
H	+7.22156	+4.36935	+3.10939
H	+4.96787	+3.31956	+2.40939
H	+3.55741	+4.67137	+5.55593
C	+2.55781	+5.22008	+3.67004
O	+1.24025	+4.86579	+4.14305
H	+0.56748	+5.19644	+3.48952
H	+2.73832	+6.31606	+3.86596

C	+2.68120	+4.95404	+2.17125
O	+1.76915	+5.85308	+1.49511
H	+1.99989	+5.84972	+0.51815
H	+2.39931	+3.88858	+1.94755
C	+4.12048	+5.20146	+1.70001
H	+4.35311	+6.29918	+1.72411
O	+4.21230	+4.71020	+0.30729
C	+4.54152	+5.71631	−0.69460
O	+5.82472	+5.36042	−1.29637
C	+5.82681	+4.00566	−1.90989
C	+7.25869	+3.84203	−2.43034
O	+7.57247	+2.48273	−2.72385
H	+6.99596	+2.17467	−3.46551
H	+7.40314	+4.48883	−3.33997
H	+7.96843	+4.19403	−1.65115
H	+5.60232	+3.25336	−1.11156
H	+4.68121	+6.72254	−0.21553
C	+3.48106	+5.74404	−1.78956
O	+2.22120	+6.11816	−1.19261
H	+1.49153	+5.88970	−1.82900
H	+3.77225	+6.51866	−2.55604
C	+3.37580	+4.38035	−2.46759
O	+2.44058	+4.50133	−3.56757
H	+2.57545	+3.71410	−4.17427
H	+3.00022	+3.61511	−1.73298
C	+4.74650	+3.94401	−2.99953
H	+5.03814	+4.58014	−3.87718
O	+4.59374	+2.54027	−3.44445
C	+4.93323	+2.27737	−4.83738
O	+6.06144	+1.34740	−4.86012
H	+5.27852	+3.21718	−5.34565
C	+3.75311	+1.63588	−5.56193
O	+2.63725	+2.55087	−5.49656
H	+1.81237	+2.05987	−5.75645
H	+4.03786	+1.47612	−6.64134
C	+3.37714	+0.28920	−4.94228
O	+2.37669	−0.31577	−5.79612
H	+2.29175	−1.28050	−5.53515
H	+2.95026	+0.44857	−3.91233
C	+4.61210	−0.61833	−4.84555

H	+4.94295	−0.92867	−5.87181
C	+5.75402	+0.10068	−4.11530
H	+5.43069	+0.38115	−3.08112
C	+7.07091	−0.67286	−4.05675
H	+7.36424	−1.02114	−5.08089
H	+7.87203	−0.00499	−3.67577
O	+6.95892	−1.78812	−3.16398
H	+6.46492	−2.50474	−3.63407

345

* E = 8.991 kcal/mol

C	−0.18638	+1.16485	+0.19137
C	−0.57398	−0.06383	+0.56330
C	+0.28933	−1.16850	+0.95986
C	+1.62425	−1.27983	+1.04343
C	+2.71479	−0.37027	+0.70369
C	+2.98417	+0.14117	−0.50475
C	+2.14912	−0.05570	−1.71752
H	+2.76391	−0.38760	−2.55551
C	+4.25794	+0.92149	−0.62720
C	+5.44322	+0.13996	−0.05778
C	+5.12721	−0.51752	+1.28908
C	+3.74268	−0.12505	+1.77491
C	−1.21661	+2.21393	−0.06649
C	−2.40191	+1.73150	−0.92036
C	−2.72444	+0.25447	−0.69569
C	−2.06634	−0.23497	+0.60494
C	−4.19211	−0.05666	−0.38312
C	−4.13424	−1.44003	+0.27803
C	−2.71148	−1.59080	+0.84622
O	+5.10388	−1.92976	+1.19484
H	+5.99257	−2.27026	+1.07555
C	−2.23041	−0.56374	−1.88212
C	−5.16175	+0.03560	−1.53831
H	+5.85846	−0.22115	+2.04040
H	+3.75197	+0.93450	+2.04254
H	+3.51178	−0.68217	+2.68067
H	+6.30347	+0.79981	+0.02842
H	+5.71075	−0.64991	−0.75695
H	+4.45051	+1.18630	−1.66566
H	+4.16247	+1.86663	−0.08434

H	+1.67791	+0.88204	−2.02459
H	+1.35715	−0.77969	−1.54912
H	+1.98541	−2.20157	+1.49046
H	−0.25234	−2.03835	+1.30655
H	+0.85713	+1.42968	+0.13819
H	−0.76379	+3.09607	−0.51452
H	−1.61086	+2.54387	+0.90151
H	−3.27252	+2.34652	−0.68885
H	−2.17822	+1.90029	−1.97354
H	−4.49761	+0.66999	+0.37474
H	−4.33613	−2.21646	−0.46028
H	−4.89627	−1.54280	+1.04665
H	−2.17188	−2.37869	+0.32065
H	−2.71036	−1.85875	+1.90086
H	−2.46004	−1.62332	−1.77640
H	−1.15188	−0.46024	−1.99062
H	−2.69218	−0.21475	−2.80498
H	−6.17937	−0.15017	−1.20394
H	−4.93155	−0.69890	−2.30920
H	−5.14029	+1.02432	−1.99628
H	−2.44085	+0.43873	+1.38692
O	−6.29966	−4.53697	−2.62344
H	−5.66128	−4.49214	−3.37742
C	−5.94792	−5.64702	−1.79829
H	−5.86662	−6.58570	−2.41074
H	−6.76351	−5.79494	−1.05822
C	−4.63387	−5.40381	−1.05029
H	−4.64380	−4.40320	−0.54635
O	−4.60939	−6.45798	−0.00313
C	−3.45153	−6.35047	+0.88308
O	−3.47169	−5.03282	+1.50453
H	−3.56659	−7.15600	+1.65720
C	−2.18127	−6.52557	+0.05903
O	−1.05243	−6.41291	+0.95100
H	−0.22653	−6.29423	+0.40763
H	−2.18766	−7.55479	−0.40285
C	−2.10389	−5.48058	−1.05347
O	−0.94276	−5.78769	−1.86060
H	−1.03522	−5.30624	−2.73580
H	−1.99829	−4.45089	−0.60839

C	−3.37263	−5.53595	−1.91469
H	−3.39865	−6.49554	−2.49636
O	−3.31207	−4.39583	−2.85749
C	−3.34648	−4.75511	−4.27099
O	−4.55640	−4.18704	−4.86107
C	−4.62714	−2.70934	−4.72764
C	−5.98867	−2.34554	−5.32711
O	−6.35766	−1.00054	−5.02536
H	−5.76109	−0.39232	−5.52683
H	−5.96723	−2.51019	−6.43841
H	−6.76396	−3.01724	−4.89997
H	−4.58975	−2.44817	−3.63859
H	−3.42674	−5.86879	−4.39265
C	−2.13172	−4.19110	−5.00012
O	−0.94668	−4.75084	−4.39492
H	−0.15709	−4.22520	−4.69443
H	−2.18142	−4.51036	−6.08086
C	−2.10303	−2.66510	−4.93207
O	−1.00256	−2.21490	−5.75879
H	−1.11363	−1.22937	−5.90934
H	−1.94046	−2.33298	−3.86972
C	−3.42632	−2.08443	−5.45062
H	−3.50973	−2.24793	−6.55782
O	−3.41163	−0.62964	−5.17163
C	−3.48304	+0.23123	−6.34764
O	−4.72957	+0.99151	−6.29065
C	−4.84406	+1.83339	−5.07136
C	−6.23179	+2.47112	−5.18869
O	−6.65416	+3.05176	−3.95569
H	−6.09984	+3.85117	−3.77820
H	−6.22601	+3.24074	−6.00754
H	−6.96779	+1.68635	−5.46676
H	−4.78828	+1.16576	−4.17385
H	−3.53074	−0.38595	−7.28480
C	−2.31202	+1.20728	−6.37042
O	−1.09204	+0.43927	−6.42275
H	−0.32620	+1.04065	−6.21736
H	−2.39080	+1.84106	−7.30046
C	−2.32960	+2.11941	−5.14481
O	−1.26558	+3.08845	−5.30421

H	−1.43285	+3.84181	−4.66291
H	−2.15631	+1.51407	−4.21052
C	−3.68225	+2.83601	−5.04107
H	−3.78715	+3.58267	−5.87244
O	−3.69757	+3.54565	−3.74186
C	−3.87736	+4.99132	−3.81815
O	−5.13418	+5.33271	−3.15578
C	−5.18158	+4.86774	−1.74565
C	−6.58928	+5.24257	−1.27253
O	−6.93176	+4.58244	−0.05484
H	−6.36814	+4.94740	+0.67128
H	−6.66286	+6.35781	−1.15322
H	−7.32569	+4.93569	−2.04598
H	−5.04143	+3.75636	−1.73506
H	−3.97851	+5.32147	−4.88682
C	−2.73686	+5.70940	−3.10395
O	−1.50304	+5.35351	−3.76637
H	−0.74292	+5.58941	−3.17073
H	−2.89768	+6.82193	−3.19181
C	−2.68515	+5.32078	−1.62773
O	−1.68212	+6.14722	−0.98690
H	−1.79621	+6.05091	+0.00524
H	−2.39619	+4.23884	−1.53612
C	−4.04874	+5.53755	−0.95627
H	−4.24961	+6.63525	−0.83760
O	−3.97636	+4.90510	+0.38142
C	−4.17968	+5.80473	+1.51108
O	−5.39588	+5.39757	+2.21206
C	−5.33960	+3.99533	+2.70125
C	−6.72404	+3.75279	+3.30934
O	−6.95537	+2.36738	+3.56111
H	−6.36340	+2.07949	+4.29896
H	−6.83032	+4.35047	+4.25505
H	−7.50026	+4.10530	+2.59670
H	−5.16711	+3.32153	+1.82381
H	−4.35612	+6.85432	+1.15208
C	−3.00281	+5.72524	+2.47725
O	−1.80813	+6.10539	+1.76040
H	−1.01794	+5.82941	+2.29793
H	−3.17811	+6.45631	+3.31819

C	−2.85585	+4.32095	+3.06141
O	−1.81411	+4.37454	+4.06478
H	−1.87680	+3.54091	+4.62013
H	−2.56359	+3.59704	+2.24896
C	−4.17688	+3.86332	+3.69407
H	−4.38661	+4.46062	+4.62077
O	−4.01834	+2.43643	+4.05737
C	−4.16438	+2.13604	+5.47782
O	−5.32768	+1.26867	+5.64873
C	−5.21647	−0.01543	+4.90823
C	−6.55489	−0.71079	+5.17482
O	−6.75263	−1.83121	+4.31412
H	−6.12118	−2.54473	+4.57816
H	−6.60727	−1.02525	+6.25229
H	−7.37979	+0.01112	+4.99224
H	−5.09551	+0.21286	+3.81883
H	−4.38129	+3.07361	+6.05722
C	−2.92945	+1.41597	+6.00830
O	−1.78825	+2.27828	+5.81967
H	−0.96114	+1.73821	+5.94142
H	−3.07214	+1.22784	+7.11164
C	−2.71997	+0.07826	+5.30098
O	−1.60824	−0.58662	+5.94698
H	−1.62777	−1.55398	+5.68207
H	−2.48274	+0.24907	+4.21388
C	−3.98660	−0.77916	+5.41843
H	−4.13922	−1.09459	+6.48470
O	−3.77949	−1.98034	+4.57886
C	−3.82953	−3.25437	+5.28823
O	−4.96846	−4.01634	+4.78110
H	−4.01713	−3.08963	+6.38327
C	−2.55656	−4.05806	+5.04364
O	−1.44031	−3.29456	+5.55002
H	−0.59819	−3.69304	+5.20120
H	−2.62977	−5.02913	+5.61217
C	−2.37097	−4.35837	+3.55758
O	−1.23320	−5.24473	+3.42744
H	−1.23171	−5.60401	+2.49056
H	−2.16823	−3.40097	+3.00214
C	−3.62707	−5.02411	+2.97647

H	−3.70948	−6.07985	+3.34815
C	−4.89973	−4.23568	+3.31362
H	−4.86759	−3.23679	+2.80773
C	−6.20198	−4.97052	+2.98143
H	−6.15226	−6.02292	+3.37346
H	−7.04713	−4.45963	+3.49086
O	−6.46966	−4.96793	+1.57979
H	−5.79102	−5.52717	+1.12747
O	+6.88857	−3.51081	−2.57228
H	+6.30588	−4.26281	−2.30011
C	+6.62148	−3.20844	−3.94182
H	+6.71366	−4.13152	−4.57569
H	+7.38845	−2.48236	−4.28746
C	+5.23121	−2.59310	−4.12537
H	+5.07057	−1.75322	−3.40243
O	+5.26036	−2.02973	−5.50013
C	+4.03321	−1.31527	−5.84362
O	+3.84180	−0.24070	−4.87493
H	+4.18742	−0.89422	−6.87339
C	+2.86322	−2.29208	−5.79872
O	+1.66077	−1.56316	−6.12664
H	+0.87380	−2.12118	−5.88317
H	+3.03373	−3.09279	−6.57453
C	+2.74036	−2.94718	−4.42398
O	+1.70381	−3.95403	−4.51145
H	+1.78152	−4.54747	−3.70537
H	+2.45574	−2.17490	−3.65614
C	+4.07263	−3.59399	−4.02032
H	+4.27387	−4.49391	−4.66022
O	+3.94087	−4.01463	−2.60896
C	+4.10809	−5.44078	−2.36105
O	+5.28708	−5.62233	−1.51583
C	+5.18253	−4.89266	−0.22412
C	+6.53775	−5.12794	+0.44784
O	+6.75452	−4.21871	+1.53133
H	+6.13818	−4.46745	+2.26645
H	+6.60823	−6.18871	+0.80799
H	+7.34672	−4.96867	−0.29738
H	+5.03016	−3.80539	−0.44105
H	+4.31603	−5.98756	−3.32007

C	+2.88847	−6.00179	−1.63769
O	+1.73702	−5.79795	−2.48446
H	+0.91489	−5.91599	−1.93654
H	+3.04035	−7.10852	−1.47977
C	+2.69410	−5.32654	−0.28105
O	+1.60772	−6.00386	+0.39666
H	+1.65307	−5.76204	+1.36926
H	+2.43537	−4.24125	−0.42378
C	+3.97717	−5.43830	+0.55275
H	+4.15338	−6.50730	+0.84821
O	+3.77360	−4.62092	+1.76531
C	+3.89186	−5.32984	+3.03022
O	+5.04065	−4.78990	+3.75565
C	+4.92650	−3.32611	+4.00899
C	+6.23988	−2.97197	+4.71259
O	+6.45798	−1.56025	+4.74713
H	+5.79958	−1.16149	+5.36791
H	+6.24229	−3.39539	+5.75351
H	+7.08613	−3.43421	+4.15924
H	+4.84432	−2.79580	+3.01872
H	+4.10792	−6.41899	+2.85676
C	+2.63385	−5.12613	+3.86865
O	+1.51255	−5.65173	+3.12373
H	+0.67328	−5.32466	+3.54445
H	+2.74136	−5.70295	+4.83176
C	+2.42007	−3.64799	+4.18936
O	+1.28771	−3.55313	+5.08817
H	+1.28281	−2.62894	+5.47830
H	+2.20063	−3.08103	+3.24222
C	+3.67264	−3.05845	+4.85088
H	+3.79435	−3.47404	+5.88684
O	+3.47399	−1.59231	+4.93384
C	+3.46504	−1.03747	+6.28035
O	+4.61172	−0.13836	+6.41714
C	+4.60302	+0.96331	+5.42104
C	+5.90411	+1.72521	+5.68909
O	+6.22376	+2.61513	+4.61925
H	+5.57173	+3.35882	+4.62556
H	+5.82227	+2.28619	+6.65892
H	+6.73735	+0.99666	+5.78832

H	+4.61264	+0.51317	+4.39528
H	+3.60469	−1.84824	+7.04477
C	+2.18636	−0.24199	+6.51789
O	+1.06589	−1.14117	+6.38103
H	+0.23778	−0.59985	+6.27753
H	+2.20594	+0.16887	+7.56845
C	+2.07287	+0.91841	+5.53070
O	+0.90520	+1.69145	+5.89751
H	+0.96560	+2.58092	+5.43766
H	+1.95535	+0.52298	+4.48369
C	+3.32833	+1.79724	+5.60582
H	+3.35739	+2.34248	+6.58636
O	+3.24087	+2.78063	+4.50251
C	+3.22822	+4.17932	+4.91393
O	+4.42331	+4.82614	+4.37425
C	+4.50617	+4.73938	+2.89352
C	+5.84883	+5.39307	+2.55378
O	+6.24327	+5.12281	+1.20899
H	+5.64021	+5.61666	+0.60042
H	+5.78604	+6.50040	+2.73281
H	+6.63162	+4.98464	+3.22855
H	+4.50586	+3.65796	+2.60207
H	+3.29829	+4.26355	+6.03162
C	+1.99563	+4.88942	+4.36496
O	+0.82371	+4.24202	+4.90300
H	+0.03152	+4.53813	+4.37881
H	+2.01650	+5.96339	+4.70963
C	+1.97732	+4.85695	+2.83782
O	+0.85144	+5.65385	+2.39844
H	+0.97649	+5.85598	+1.42407
H	+1.85797	+3.79765	+2.47705
C	+3.28421	+5.43980	+2.28404
H	+3.32771	+6.54325	+2.48418
O	+3.28190	+5.20787	+0.82245
C	+3.34663	+6.41053	+0.00042
O	+4.59168	+6.37579	−0.76440
C	+4.70534	+5.16826	−1.62225
C	+6.09256	+5.28967	−2.26008
O	+6.50955	+4.06237	−2.85734
H	+5.95357	+3.89840	−3.65862

H	+6.08867	+6.11811	−3.01908
H	+6.83085	+5.55496	−1.47300
H	+4.64801	+4.26173	−0.96672
H	+3.39427	+7.32863	+0.64543
C	+2.17413	+6.45760	−0.97295
O	+0.95286	+6.49485	−0.20498
H	+0.18620	+6.33189	−0.81834
H	+2.25381	+7.40016	−1.58716
C	+2.18872	+5.24973	−1.90857
O	+1.12452	+5.43377	−2.87248
H	+1.28323	+4.80090	−3.63499
H	+2.01067	+4.30652	−1.32056
C	+3.54308	+5.15500	−2.62447
H	+3.64975	+5.99842	−3.35715
O	+3.56439	+3.86774	−3.35369
C	+3.73307	+3.97149	−4.79841
O	+4.98884	+3.31855	−5.16184
H	+3.83076	+5.04613	−5.10949
C	+2.58689	+3.26988	−5.51802
O	+1.35686	+3.92808	−5.14718
H	+0.59262	+3.34295	−5.39879
H	+2.74109	+3.37191	−6.63061
C	+2.54075	+1.78731	−5.15312
O	+1.51609	+1.16872	−5.96664
H	+1.64236	+0.17421	−5.92147
H	+2.28489	+1.66869	−4.06297
C	+3.89927	+1.12990	−5.43159
H	+4.07795	+1.07333	−6.53814
C	+5.04032	+1.89322	−4.74579
H	+4.91198	+1.84356	−3.63343
C	+6.44472	+1.43636	−5.15144
H	+6.51092	+1.36848	−6.27109
H	+7.18372	+2.19458	−4.81391
O	+6.78972	+0.18879	−4.55074
H	+6.23391	−0.52007	−4.95927

345

* E = 10.375 kcal/mol

C	−0.38091	−1.03461	−0.30936
C	−0.61631	+0.15572	−0.88035
C	+0.31687	+1.26926	−0.98345

C	+1.55310	+1.45546	−0.49220
C	+2.39579	+0.65523	+0.39353
C	+2.08130	+0.22442	+1.62373
C	+0.77419	+0.44199	+2.29360
H	+0.92754	+0.79014	+3.31595
C	+3.15081	−0.50951	+2.37627
C	+3.80444	−1.55179	+1.48057
C	+4.35433	−0.94290	+0.18895
C	+3.82240	+0.46523	−0.04532
C	−1.41465	−2.10581	−0.40177
C	−2.83750	−1.62175	−0.07992
C	−3.08138	−0.18323	−0.53838
C	−1.97525	+0.25556	−1.51326
C	−4.31274	+0.02718	−1.42586
C	−4.02972	+1.36693	−2.11939
C	−2.50211	+1.55951	−2.08995
O	+5.75583	−0.79847	+0.20881
H	+6.19145	−1.64905	+0.11106
C	−3.11556	+0.74006	+0.67254
C	−5.65832	−0.04061	−0.74102
H	+4.06400	−1.57527	−0.65424
H	+3.95931	+0.73241	−1.09081
H	+4.47359	+1.12563	+0.53367
H	+3.05972	−2.31081	+1.23793
H	+4.61563	−2.05435	+2.00426
H	+3.90993	+0.19751	+2.72177
H	+2.73752	−0.97952	+3.26805
H	+0.20438	−0.48895	+2.35887
H	+0.16194	+1.16352	+1.76081
H	+2.05609	+2.34686	−0.85733
H	−0.03137	+2.06564	−1.62846
H	+0.56501	−1.25208	+0.15996
H	−1.15150	−2.95322	+0.22797
H	−1.41375	−2.48742	−1.42891
H	−3.55193	−2.29843	−0.55072
H	−3.00650	−1.69660	+0.99420
H	−4.27678	−0.76246	−2.18135
H	−4.52865	+2.17592	−1.58582
H	−4.42423	+1.37853	−3.13216
H	−2.23252	+2.40385	−1.45507

H	−2.08961	+1.76508	−3.07579
H	−3.31238	+1.77530	+0.39686
H	−2.16487	+0.70459	+1.20232
H	−3.89441	+0.43037	+1.36811
H	−6.46318	+0.07023	−1.46347
H	−5.77048	+0.75336	−0.00362
H	−5.79970	−0.99548	−0.23508
H	−2.00036	−0.48251	−2.32560
O	−6.41892	−4.72899	−2.15989
H	−5.62706	−5.18522	−2.53787
C	−6.62093	−5.19746	−0.82745
H	−6.67747	−6.31973	−0.80612
H	−7.59646	−4.80259	−0.47080
C	−5.51344	−4.72026	+0.11702
H	−5.34534	−3.61787	+0.00739
O	−6.05721	−4.97934	+1.47506
C	−5.16725	−4.52151	+2.53964
O	−4.92941	−3.09344	+2.35845
H	−5.70346	−4.71267	+3.50774
C	−3.85665	−5.29666	+2.45150
O	−2.98838	−4.82162	+3.50363
H	−2.05904	−5.11558	+3.30787
H	−4.07194	−6.39158	+2.61408
C	−3.20012	−5.10942	+1.08508
O	−2.04409	−5.98086	+1.02631
H	−1.74917	−6.03428	+0.06883
H	−2.87306	−4.04024	+0.96934
C	−4.18017	−5.46351	−0.04216
H	−4.35070	−6.57237	−0.07003
O	−3.55175	−5.03264	−1.31273
C	−3.31424	−6.09327	−2.28571
O	−4.13000	−5.82617	−3.46844
C	−3.83781	−4.50638	−4.08569
C	−4.84727	−4.39543	−5.23201
O	−4.91341	−3.06825	−5.75184
H	−4.06035	−2.86681	−6.20948
H	−4.57799	−5.12563	−6.04281
H	−5.85681	−4.66553	−4.85456
H	−4.01738	−3.71044	−3.31895
H	−3.65613	−7.08340	−1.88033

C	−1.84619	−6.12432	−2.69722
O	−1.05458	−6.36430	−1.51285
H	−0.10862	−6.13529	−1.71580
H	−1.69376	−6.97418	−3.42280
C	−1.42822	−4.81569	−3.36554
O	−0.07528	−4.98573	−3.85207
H	+0.12416	−4.22498	−4.47550
H	−1.45721	−3.97832	−2.61341
C	−2.36910	−4.47609	−4.52957
H	−2.20318	−5.18872	−5.38043
O	−2.03785	−3.10123	−4.96996
C	−1.56999	−2.98227	−6.34660
O	−2.53983	−2.18573	−7.09541
C	−2.72714	−0.82005	−6.53902
C	−3.82477	−0.20899	−7.41519
O	−4.35366	+0.98742	−6.84518
H	−3.66387	+1.69353	−6.90242
H	−3.41952	−0.01164	−8.44444
H	−4.65724	−0.93858	−7.51300
H	−3.07146	−0.91425	−5.47787
H	−1.54017	−3.99093	−6.84009
C	−0.21431	−2.28616	−6.39828
O	+0.72761	−3.07537	−5.64181
H	+1.54328	−2.52691	−5.48536
H	+0.12252	−2.24014	−7.47413
C	−0.29656	−0.86456	−5.84405
O	+0.99539	−0.24340	−6.04710
H	+0.88127	+0.74903	−5.95410
H	−0.53767	−0.89216	−4.74487
C	−1.38474	−0.07718	−6.58541
H	−1.07391	+0.09855	−7.64947
O	−1.52923	+1.22557	−5.89774
C	−1.27470	+2.39961	−6.72577
O	−2.51010	+3.17468	−6.81597
C	−3.02716	+3.58832	−5.48635
C	−4.34093	+4.30984	−5.80144
O	−5.13752	+4.49444	−4.63190
H	−4.68315	+5.14306	−4.03983
H	−4.12123	+5.29738	−6.29112
H	−4.92717	+3.69853	−6.52078

H	−3.21669	+2.66696	−4.87809
H	−1.01287	+2.09393	−7.77428
C	−0.19263	+3.27423	−6.10139
O	+1.02053	+2.49603	−6.01777
H	+1.65277	+2.95953	−5.40548
H	−0.01708	+4.16328	−6.77271
C	−0.61198	+3.76674	−4.71773
O	+0.39672	+4.69932	−4.26031
H	+0.02673	+5.18580	−3.46431
H	−0.67159	+2.89598	−4.00645
C	−1.97900	+4.46283	−4.78496
H	−1.88100	+5.45131	−5.30735
O	−2.42174	+4.68739	−3.39051
C	−2.61374	+6.08127	−3.00939
O	−4.02522	+6.27833	−2.68420
C	−4.49625	+5.38610	−1.59304
C	−5.99059	+5.69816	−1.47088
O	−6.67643	+4.70722	−0.70630
H	−6.38764	+4.78264	+0.23657
H	−6.12881	+6.71600	−1.01503
H	−6.44053	+5.71804	−2.48674
H	−4.33825	+4.32380	−1.91123
H	−2.38704	+6.76284	−3.87275
C	−1.77544	+6.41529	−1.78085
O	−0.38604	+6.21860	−2.11820
H	+0.14503	+6.20090	−1.27667
H	−1.94182	+7.49842	−1.51320
C	−2.17468	+5.54078	−0.59334
O	−1.42305	+5.99578	+0.55646
H	−1.86223	+5.62825	+1.38009
H	−1.92742	+4.46284	−0.80905
C	−3.67968	+5.67274	−0.32541
H	−3.91134	+6.69961	+0.06395
O	−4.02847	+4.66925	+0.70593
C	−4.60662	+5.20980	+1.93109
O	−5.96749	+4.69413	+2.06270
C	−6.01975	+3.21014	+2.09889
C	−7.51617	+2.88806	+2.14321
O	−7.76666	+1.50794	+1.87953
H	−7.44135	+0.97890	+2.64877

H	−7.93680	+3.18263	+3.14249
H	−8.03896	+3.48549	+1.36577
H	−5.55685	+2.81487	+1.15807
H	−4.69495	+6.32757	+1.86578
C	−3.79563	+4.77465	+3.14705
O	−2.45179	+5.27887	+2.99208
H	−1.86123	+4.82024	+3.64806
H	−4.25807	+5.23111	+4.06906
C	−3.78788	+3.25379	+3.29447
O	−3.11990	+2.94273	+4.54097
H	−3.31313	+1.98379	+4.76333
H	−3.22677	+2.78938	+2.43700
C	−5.22490	+2.71319	+3.31409
H	−5.73697	+3.01491	+4.26608
O	−5.14220	+1.23596	+3.25099
C	−5.70703	+0.52645	+4.39324
O	−6.84030	−0.27010	+3.92730
H	−6.11665	+1.25300	+5.14537
C	−4.66985	−0.40606	+5.00951
O	−3.55726	+0.39776	+5.45409
H	−2.78599	−0.20064	+5.64673
H	−5.13340	−0.92142	+5.89964
C	−4.21130	−1.46428	+4.00662
O	−3.33089	−2.37634	+4.70597
H	−3.24359	−3.20924	+4.15343
H	−3.65624	−0.97613	+3.15659
C	−5.42331	−2.22323	+3.45014
H	−5.88104	−2.85998	+4.25301
C	−6.46472	−1.25235	+2.87726
H	−6.02261	−0.69398	+2.01268
C	−7.79300	−1.90321	+2.48032
H	−8.15121	−2.56995	+3.31065
H	−8.55505	−1.10832	+2.33111
O	−7.67792	−2.63602	+1.26113
H	−7.13291	−3.44327	+1.43151
O	+6.10263	−5.07853	+2.15982
H	+5.29554	−5.50869	+2.53660
C	+6.26910	−5.52486	+0.81487
H	+6.24100	−6.64645	+0.76276
H	+7.27176	−5.19492	+0.46633

C	+5.19993	−4.93764	−0.11187
H	+5.10764	−3.83101	+0.04176
O	+5.72838	−5.17911	−1.47897
C	+4.86619	−4.63839	−2.53088
O	+4.73597	−3.20069	−2.32359
H	+5.38400	−4.85165	−3.50413
C	+3.50538	−5.32118	−2.44872
O	+2.66640	−4.78550	−3.49230
H	+1.72672	−5.06128	−3.31518
H	+3.64861	−6.42712	−2.61937
C	+2.86523	−5.10805	−1.07836
O	+1.63780	−5.87394	−1.04509
H	+1.34785	−5.95326	−0.08785
H	+2.63807	−4.01587	−0.92513
C	+3.81763	−5.58854	+0.02402
H	+3.90629	−6.70682	−0.00413
O	+3.23378	−5.17481	+1.31837
C	+2.92569	−6.26217	+2.24071
O	+3.75377	−6.09924	+3.43317
C	+3.54998	−4.78682	+4.09980
C	+4.54420	−4.79796	+5.26512
O	+4.73815	−3.49271	+5.80744
H	+3.90816	−3.21594	+6.26850
H	+4.18790	−5.50872	+6.05937
H	+5.52710	−5.16520	+4.89882
H	+3.80419	−3.97642	+3.36941
H	+3.20338	−7.25314	+1.79135
C	+1.45817	−6.20858	+2.64839
O	+0.65693	−6.38075	+1.46013
H	−0.28714	−6.15086	+1.67595
H	+1.24889	−7.05604	+3.36224
C	+1.13328	−4.88291	+3.33481
O	−0.23725	−4.95528	+3.79415
H	−0.38033	−4.22034	+4.46264
H	+1.24591	−4.03474	+2.60307
C	+2.08001	−4.65875	+4.52200
H	+1.84443	−5.38687	+5.34299
O	+1.85163	−3.28070	+5.00919
C	+1.41686	−3.17304	+6.39719
O	+2.43812	−2.44144	+7.14266

C	+2.70024	−1.08796	+6.58790
C	+3.83627	−0.54120	+7.45760
O	+4.43321	+0.61907	+6.88113
H	+3.78508	+1.36420	+6.93178
H	+3.44769	−0.31693	+8.48791
H	+4.62391	−1.31911	+7.55464
H	+3.03153	−1.19985	+5.52348
H	+1.34390	−4.18952	+6.86906
C	+0.10146	−2.40754	+6.48103
O	−0.89275	−3.14502	+5.73833
H	−1.67532	−2.55263	+5.57569
H	−0.21407	−2.34586	+7.56207
C	+0.25808	−0.99350	+5.92449
O	−0.99032	−0.29673	+6.14826
H	−0.82317	+0.68636	+6.03522
H	+0.47776	−1.03728	+4.82072
C	+1.40416	−0.26773	+6.64223
H	+1.11868	−0.06046	+7.70766
O	+1.62039	+1.01504	+5.93566
C	+1.43984	+2.21250	+6.75089
O	+2.72067	+2.90863	+6.83978
C	+3.26318	+3.29626	+5.51161
C	+4.61897	+3.93304	+5.83188
O	+5.42522	+4.07931	+4.66416
H	+5.01387	+4.76311	+4.08033
H	+4.45953	+4.92751	+6.33099
H	+5.16641	+3.28040	+6.54553
H	+3.39428	+2.36836	+4.89907
H	+1.15588	+1.93392	+7.80130
C	+0.41466	+3.14384	+6.11386
O	−0.83940	+2.43368	+6.02413
H	−1.44618	+2.93415	+5.41503
H	+0.28301	+4.04381	+6.78074
C	+0.87196	+3.60894	+4.73242
O	−0.08949	+4.58440	+4.26335
H	+0.31750	+5.07006	+3.48475
H	+0.90162	+2.73425	+4.02472
C	+2.26952	+4.23787	+4.81788
H	+2.21732	+5.21932	+5.35988
O	+2.73255	+4.46756	+3.43137

C	+3.00597	+5.85750	+3.08589
O	+4.42664	+5.98231	+2.76757
C	+4.85175	+5.10776	+1.64315
C	+6.35940	+5.35433	+1.53131
O	+6.99858	+4.38096	+0.70787
H	+6.70418	+4.51455	−0.22618
H	+6.54229	+6.39051	+1.13553
H	+6.81160	+5.29485	+2.54469
H	+4.64087	+4.04440	+1.92212
H	+2.81772	+6.52788	+3.96730
C	+2.18995	+6.27508	+1.86798
O	+0.79058	+6.14629	+2.19713
H	+0.26382	+6.16909	+1.35348
H	+2.41703	+7.35548	+1.63568
C	+2.54234	+5.42047	+0.65187
O	+1.81613	+5.95676	−0.48025
H	+2.23147	+5.59205	−1.31756
H	+2.23670	+4.35179	+0.82817
C	+4.05256	+5.48329	+0.38771
H	+4.33766	+6.51094	+0.03756
O	+4.35264	+4.50309	−0.68008
C	+4.93179	+5.06300	−1.89650
O	+6.27252	+4.50911	−2.06644
C	+6.28838	+3.02429	−2.14339
C	+7.77509	+2.68112	−2.28327
O	+8.03640	+1.30419	−2.02173
H	+7.57069	+0.75706	−2.70059
H	+8.12917	+2.96422	−3.31342
H	+8.35577	+3.28254	−1.55117
H	+5.87067	+2.61448	−1.18912
H	+5.05656	+6.17497	−1.79913
C	+4.09136	+4.68711	−3.11153
O	+2.77349	+5.25182	−2.94385
H	+2.16352	+4.83288	−3.60874
H	+4.56924	+5.13002	−4.03204
C	+4.01286	+3.17043	−3.26661
O	+3.30187	+2.88986	−4.49662
H	+3.46361	+1.92813	−4.73322
H	+3.45476	+2.72536	−2.39515
C	+5.42229	+2.56733	−3.32620

H	+5.91390	+2.83574	−4.29898
O	+5.25799	+1.09863	−3.25013
C	+5.79155	+0.34549	−4.37743
O	+6.85584	−0.52512	−3.87907
H	+6.26395	+1.03447	−5.12792
C	+4.70150	−0.52037	−5.00172
O	+3.64755	+0.34853	−5.46968
H	+2.83647	−0.20233	−5.63627
H	+5.14111	−1.07487	−5.88023
C	+4.15604	−1.53500	−3.99697
O	+3.24198	−2.40207	−4.70949
H	+3.07845	−3.21335	−4.14359
H	+3.60931	−1.00185	−3.16953
C	+5.30720	−2.35393	−3.39753
H	+5.76141	−3.01400	−4.18337
C	+6.37301	−1.42527	−2.79997
H	+5.92750	−0.79593	−1.98621
C	+7.62706	−2.13386	−2.28674
H	+8.03637	−2.81927	−3.07374
H	+8.40448	−1.37701	−2.05056
O	+7.34639	−2.86895	−1.08720
H	+6.85811	−3.69173	−1.34084

345

* E = 10.744 kcal/mol

C	−0.45037	−1.00694	−0.37991
C	−0.68706	+0.19132	−0.93278
C	+0.24497	+1.30999	−1.00740
C	+1.45075	+1.51058	−0.45033
C	+2.22138	+0.72028	+0.50739
C	+1.80352	+0.29946	+1.70984
C	+0.45612	+0.55228	+2.27813
H	+0.53481	+0.86653	+3.31937
C	+2.79546	−0.44392	+2.55450
C	+3.55600	−1.51152	+1.77073
C	+3.88995	−1.08455	+0.33987
C	+3.65022	+0.40573	+0.14820
C	−1.46910	−2.08707	−0.51369
C	−2.90238	−1.62672	−0.20658
C	−3.15532	−0.18358	−0.64609
C	−2.03461	+0.28648	−1.59002

C	−4.37050	+0.02804	−1.55518
C	−4.08951	+1.38429	−2.21714
C	−2.56493	+1.59405	−2.15378
O	+5.18508	−1.47435	−0.04639
H	+5.82598	−1.11296	+0.56482
C	−3.22872	+0.71763	+0.57935
C	−5.72858	−0.06853	−0.89861
H	+3.23843	−1.60825	−0.35836
H	+3.87618	+0.67430	−0.88102
H	+4.34127	+0.96183	+0.79076
H	+2.98109	−2.43622	+1.74920
H	+4.48537	−1.74775	+2.28744
H	+3.50737	+0.27864	+2.96312
H	+2.30270	−0.89478	+3.41474
H	−0.14907	−0.35825	+2.26575
H	−0.08286	+1.30956	+1.71684
H	+1.97993	+2.39107	−0.80328
H	−0.07128	+2.09032	−1.68823
H	+0.48853	−1.22501	+0.10392
H	−1.20470	−2.94506	+0.10057
H	−1.44507	−2.44429	−1.54915
H	−3.60049	−2.30491	−0.69899
H	−3.08701	−1.72173	+0.86344
H	−4.30965	−0.74671	−2.32427
H	−4.60853	+2.17674	−1.67804
H	−4.46381	+1.41128	−3.23718
H	−2.31544	+2.43032	−1.50000
H	−2.13608	+1.81969	−3.12807
H	−3.43418	+1.75497	+0.31814
H	−2.28986	+0.68541	+1.12909
H	−4.01752	+0.38414	+1.25223
H	−6.52001	+0.04269	−1.63573
H	−5.86705	+0.71200	−0.15144
H	−5.86715	−1.03282	−0.41025
H	−2.03455	−0.43712	−2.41562
O	−6.26829	−4.87497	−2.33495
H	−5.45331	−5.29400	−2.70716
C	−6.46669	−5.36273	−1.00899
H	−6.48379	−6.48653	−0.99818
H	−7.45920	−5.00597	−0.65890

C	−5.38624	−4.85556	−0.04894
H	−5.25301	−3.74772	−0.15053
O	−5.93773	−5.13978	+1.30129
C	−5.07273	−4.66950	+2.38090
O	−4.87124	−3.23321	+2.21746
H	−5.61492	−4.88504	+3.34053
C	−3.74090	−5.40796	+2.29945
O	−2.89657	−4.92616	+3.36817
H	−1.95758	−5.18788	+3.17325
H	−3.92859	−6.51047	+2.44335
C	−3.07416	−5.18100	+0.94455
O	−1.88795	−6.00994	+0.89008
H	−1.57793	−6.04028	−0.06388
H	−2.78489	−4.09890	+0.84736
C	−4.02728	−5.55269	−0.19959
H	−4.15990	−6.66611	−0.24512
O	−3.39718	−5.08214	−1.45487
C	−3.11161	−6.11875	−2.44082
O	−3.92116	−5.85821	−3.62947
C	−3.66604	−4.51597	−4.21350
C	−4.66921	−4.40560	−5.36534
O	−4.78564	−3.06347	−5.83664
H	−3.93846	−2.81139	−6.28029
H	−4.36343	−5.09348	−6.19980
H	−5.67003	−4.73030	−5.00821
H	−3.87601	−3.74468	−3.42943
H	−3.42609	−7.12588	−2.05558
C	−1.63794	−6.09532	−2.83277
O	−0.85564	−6.32455	−1.64011
H	+0.08873	−6.07407	−1.82803
H	−1.44809	−6.93052	−3.56647
C	−1.25241	−4.76557	−3.47946
O	+0.10663	−4.89313	−3.96021
H	+0.28956	−4.12052	−4.57382
H	−1.30760	−3.94122	−2.71443
C	−2.19548	−4.43100	−4.64347
H	−2.00090	−5.11959	−5.50785
O	−1.90486	−3.03705	−5.05225
C	−1.45126	−2.87238	−6.42887
O	−2.43980	−2.07013	−7.14662

C	−2.64112	−0.72185	−6.55458
C	−3.75646	−0.10723	−7.40529
O	−4.27754	+1.08396	−6.81714
H	−3.59049	+1.79165	−6.88336
H	−3.37031	+0.09892	−8.43996
H	−4.58872	−0.83823	−7.49348
H	−2.97267	−0.84690	−5.49243
H	−1.41136	−3.86613	−6.95095
C	−0.10613	−2.15569	−6.47205
O	+0.85009	−2.95063	−5.74056
H	+1.65737	−2.39489	−5.56523
H	+0.22336	−2.07851	−7.54849
C	−0.20472	−0.74885	−5.88352
O	+1.07609	−0.10544	−6.08334
H	+0.95337	+0.88109	−5.95059
H	−0.43594	−0.80549	−4.78328
C	−1.30989	+0.04102	−6.59710
H	−1.01197	+0.24360	−7.66000
O	−1.46542	+1.32796	−5.88080
C	−1.20657	+2.52129	−6.67987
O	−2.44674	+3.28835	−6.77810
C	−2.99071	+3.67789	−5.45199
C	−4.30000	+4.40170	−5.78056
O	−5.12395	+4.55666	−4.62572
H	−4.68475	+5.19058	−4.00692
H	−4.07356	+5.40098	−6.24275
H	−4.86718	+3.80465	−6.52666
H	−3.19069	+2.74562	−4.86428
H	−0.92307	+2.23924	−7.72934
C	−0.14399	+3.39410	−6.02002
O	+1.07643	+2.62856	−5.93287
H	+1.69363	+3.08507	−5.30059
H	+0.03158	+4.29861	−6.67055
C	−0.59067	+3.85431	−4.63394
O	+0.40349	+4.78340	−4.13802
H	+0.01432	+5.25232	−3.34071
H	−0.65669	+2.96841	−3.94213
C	−1.96092	+4.54273	−4.71202
H	−1.85959	+5.54357	−5.20968
O	−2.43156	+4.73263	−3.32138

C	−2.64391	+6.11552	−2.91248
O	−4.06260	+6.29141	−2.60670
C	−4.54225	+5.36610	−1.54742
C	−6.04233	+5.65628	−1.44315
O	−6.72890	+4.63267	−0.72342
H	−6.45914	+4.68160	+0.22683
H	−6.20202	+6.65712	−0.95768
H	−6.47403	+5.70308	−2.46599
H	−4.36568	+4.31441	−1.88997
H	−2.40931	+6.81884	−3.75602
C	−1.83024	+6.42885	−1.66203
O	−0.43323	+6.25450	−1.97989
H	+0.08268	+6.22316	−1.12938
H	−2.01271	+7.50340	−1.37139
C	−2.24035	+5.52143	−0.50319
O	−1.51498	+5.95575	+0.67166
H	−1.96975	+5.56872	+1.47767
H	−1.97687	+4.45197	−0.74063
C	−3.75111	+5.62964	−0.25881
H	−4.00157	+6.64349	+0.15245
O	−4.10482	+4.59624	+0.74083
C	−4.71928	+5.09725	+1.96513
O	−6.07092	+4.54988	+2.05721
C	−6.09026	+3.06471	+2.05708
C	−7.57905	+2.70662	+2.05968
O	−7.78925	+1.32868	+1.75239
H	−7.47084	+0.78665	+2.51552
H	−8.02831	+2.96261	+3.05702
H	−8.09939	+3.31292	+1.28748
H	−5.59790	+2.70305	+1.11770
H	−4.83020	+6.21408	+1.92253
C	−3.92393	+4.65171	+3.18768
O	−2.58856	+5.18829	+3.07278
H	−2.00460	+4.73330	+3.73713
H	−4.41503	+5.07626	+4.11003
C	−3.88547	+3.12826	+3.30001
O	−3.23512	+2.80203	+4.55211
H	−3.41513	+1.83512	+4.74998
H	−3.29768	+2.69619	+2.44333
C	−5.31055	+2.55707	+3.27776

H	−5.84853	+2.82664	+4.22510
O	−5.19451	+1.08389	+3.18429
C	−5.77101	+0.33663	+4.29619
O	−6.87446	−0.47353	+3.78477
H	−6.21517	+1.03711	+5.05347
C	−4.72787	−0.58581	+4.91751
O	−3.64327	+0.23195	+5.40438
H	−2.86429	−0.35435	+5.60212
H	−5.20002	−1.12950	+5.78594
C	−4.22315	−1.61284	+3.90445
O	−3.33497	−2.51601	+4.60503
H	−3.21420	−3.33599	+4.03916
H	−3.66347	−1.09500	+3.07511
C	−5.40596	−2.38854	+3.30956
H	−5.86928	−3.04518	+4.09295
C	−6.45273	−1.42901	+2.72740
H	−6.00117	−0.84678	+1.88341
C	−7.75746	−2.09928	+2.28712
H	−8.12732	−2.77884	+3.10164
H	−8.52871	−1.31639	+2.12236
O	−7.59452	−2.82001	+1.06629
H	−7.04418	−3.62123	+1.24733
O	+6.19898	−4.78937	+2.09532
H	+5.40949	−5.26508	+2.45203
C	+6.42829	−5.24627	+0.76091
H	+6.46751	−6.36870	+0.73165
H	+7.41924	−4.86421	+0.43429
C	+5.35354	−4.73957	−0.20448
H	+5.22999	−3.62612	−0.11994
O	+5.90281	−5.05344	−1.55187
C	+5.05177	−4.55221	−2.63040
O	+4.88165	−3.12161	−2.44881
H	+5.58716	−4.77438	−3.59292
C	+3.69658	−5.24709	−2.55185
O	+2.86628	−4.72985	−3.61498
H	+1.92631	−5.00223	−3.43873
H	+3.84291	−6.35524	−2.70199
C	+3.04174	−5.00808	−1.19152
O	+1.82490	−5.79276	−1.15155
H	+1.53774	−5.87280	−0.19448

H	+2.79427	−3.91581	−1.07156
C	+3.99403	−5.43945	−0.06803
H	+4.12720	−6.55410	−0.08671
O	+3.37031	−5.04824	+1.21855
C	+3.08814	−6.15112	+2.12950
O	+3.89902	−5.97900	+3.33467
C	+3.63900	−4.69407	+4.03195
C	+4.62481	−4.69629	+5.20410
O	+4.74728	−3.40211	+5.79402
H	+3.90308	−3.18980	+6.26297
H	+4.30042	−5.45120	+5.97034
H	+5.62757	−4.99914	+4.83325
H	+3.86702	−3.85556	+3.32519
H	+3.40064	−7.12945	+1.67542
C	+1.61687	−6.15380	+2.52885
O	+0.82496	−6.33410	+1.33681
H	−0.12525	−6.13429	+1.55320
H	+1.43514	−7.01993	+3.22846
C	+1.24011	−4.85413	+3.23767
O	−0.13563	−4.97777	+3.66991
H	−0.31264	−4.26402	+4.35238
H	+1.34052	−3.98555	+2.52730
C	+2.16184	−4.63126	+4.44413
H	+1.94439	−5.39204	+5.23996
O	+1.87815	−3.27721	+4.97176
C	+1.40787	−3.23175	+6.35164
O	+2.39031	−2.50038	+7.14898
C	+2.62668	−1.12051	+6.65219
C	+3.72399	−0.57450	+7.57059
O	+4.30400	+0.62052	+7.04922
H	+3.63587	+1.34699	+7.11075
H	+3.30314	−0.39547	+8.59683
H	+4.52985	−1.33409	+7.66250
H	+2.98886	−1.18308	+5.59391
H	+1.35214	−4.26611	+6.78512
C	+0.07038	−2.50492	+6.43305
O	−0.88821	−3.24146	+5.64511
H	−1.68505	−2.66652	+5.48978
H	−0.26816	−2.48903	+7.50885
C	+0.20002	−1.06869	+5.92911

O	−1.07241	−0.41393	+6.14348
H	−0.93048	+0.57649	+6.06656
H	+0.44907	−1.06789	+4.83016
C	+1.30754	−0.33713	+6.69936
H	+0.99007	−0.16940	+7.76259
O	+1.50748	+0.97125	+6.03622
C	+1.27581	+2.14048	+6.87818
O	+2.53612	+2.86724	+7.01282
C	+3.10087	+3.28361	+5.70328
C	+4.43708	+3.94208	+6.05917
O	+5.26921	+4.10225	+4.91079
H	+4.86350	+4.78403	+4.32023
H	+4.25019	+4.93333	+6.55452
H	+4.97745	+3.29732	+6.78517
H	+3.26418	+2.36633	+5.08218
H	+0.97555	+1.82746	+7.91409
C	+0.24261	+3.06185	+6.23904
O	−0.99226	+2.32475	+6.10695
H	−1.59564	+2.82136	+5.49143
H	+0.07579	+3.94464	+6.92054
C	+0.71944	+3.56303	+4.87701
O	−0.24770	+4.53141	+4.40516
H	+0.16692	+5.03502	+3.64218
H	+0.77817	+2.70232	+4.15419
C	+2.10437	+4.21341	+4.99787
H	+2.02565	+5.19057	+5.54414
O	+2.58928	+4.45814	+3.62141
C	+2.85887	+5.85182	+3.29027
O	+4.28397	+5.98385	+2.99277
C	+4.72241	+5.10555	+1.87706
C	+6.23519	+5.32846	+1.79446
O	+6.87098	+4.33779	+0.98721
H	+6.61700	+4.49296	+0.04398
H	+6.44479	+6.35774	+1.39615
H	+6.66680	+5.26599	+2.81656
H	+4.49209	+4.04471	+2.15115
H	+2.65468	+6.51508	+4.17335
C	+2.05795	+6.27222	+2.06296
O	+0.65496	+6.12879	+2.37081
H	+0.14072	+6.15340	+1.51934

H	+2.27930	+7.35610	+1.84214
C	+2.43456	+5.42950	+0.84540
O	+1.73156	+5.97548	−0.29627
H	+2.16690	+5.62107	−1.12771
H	+2.12588	+4.35912	+1.00613
C	+3.94929	+5.49378	+0.60949
H	+4.24193	+6.52481	+0.27635
O	+4.26610	+4.52546	−0.46429
C	+4.88430	+5.09764	−1.65571
O	+6.22622	+4.53541	−1.79180
C	+6.22239	+3.05364	−1.90167
C	+7.70535	+2.67413	−1.94823
O	+7.89792	+1.27496	−1.74405
H	+7.56643	+0.79333	−2.54202
H	+8.14705	+2.99581	−2.92975
H	+8.24378	+3.21572	−1.14073
H	+5.73351	+2.63250	−0.98602
H	+5.01484	+6.20694	−1.53779
C	+4.07526	+4.74998	−2.90067
O	+2.75009	+5.30164	−2.74591
H	+2.15917	+4.90331	−3.44008
H	+4.57115	+5.22525	−3.79524
C	+4.00791	+3.23865	−3.11259
O	+3.33610	+3.00576	−4.37313
H	+3.50190	+2.05231	−4.64143
H	+3.42460	+2.75968	−2.27585
C	+5.42322	+2.64668	−3.14665
H	+5.95605	+2.97774	−4.07728
O	+5.28431	+1.17459	−3.15771
C	+5.83379	+0.50692	−4.33478
O	+6.93729	−0.34651	−3.90737
H	+6.27051	+1.25969	−5.04526
C	+4.76814	−0.35463	−5.00239
O	+3.69097	+0.50807	−5.42735
H	+2.89425	−0.05543	−5.62032
H	+5.22111	−0.85351	−5.90698
C	+4.25995	−1.42374	−4.03969
O	+3.33324	−2.27137	−4.76231
H	+3.21207	−3.11407	−4.23110
H	+3.73296	−0.93840	−3.17191

C	+5.43131	−2.26413	−3.51698
H	+5.84085	−2.90851	−4.34087
C	+6.53577	−1.38350	−2.91718
H	+6.14592	−0.87674	−1.99684
C	+7.84085	−2.12825	−2.61864
H	+8.10222	−2.79708	−3.48532
H	+8.66354	−1.38879	−2.50670
O	+7.76229	−2.88026	−1.40888
H	+7.07107	−3.57911	−1.51992

345

* E = 11.419 kcal/mol

C	−0.75582	−1.03138	−0.25490
C	−0.99558	+0.15496	−0.82961
C	+0.00843	+1.06252	−1.37026
C	+1.34943	+1.06864	−1.27780
C	+2.27510	+0.23689	−0.51434
C	+2.22048	−0.00434	+0.80409
C	+1.18077	+0.51611	+1.72275
H	+1.63199	+0.85342	+2.65542
C	+3.28922	−0.89080	+1.36610
C	+3.43266	−2.14054	+0.50950
C	+3.63265	−1.81504	−0.96898
C	+3.47671	−0.31934	−1.23467
C	−1.89635	−1.90905	+0.13041
C	−3.04144	−1.17387	+0.84530
C	−3.20603	+0.26838	+0.36205
C	−2.45836	+0.46656	−0.96803
C	−4.62180	+0.66497	−0.06953
C	−4.39612	+1.90446	−0.94765
C	−2.94551	+1.81961	−1.45979
O	+4.91764	−2.27896	−1.30843
H	+5.12790	−2.10064	−2.22727
C	−2.67725	+1.22272	+1.42561
C	−5.62771	+0.87057	+1.03973
H	+2.88324	−2.34697	−1.56282
H	+3.41176	−0.13481	−2.30681
H	+4.38864	+0.16434	−0.87596
H	+2.54133	−2.75457	+0.63718
H	+4.28340	−2.73697	+0.83035
H	+4.24242	−0.35624	+1.39162

H	+3.06118	−1.16337	+2.39574
H	+0.46279	−0.26723	+1.98205
H	+0.62249	+1.33472	+1.27890
H	+1.84722	+1.79669	−1.91125
H	−0.40355	+1.83219	−2.01162
H	+0.25138	−1.39441	−0.12336
H	−1.54205	−2.73975	+0.73662
H	−2.30026	−2.35997	−0.78286
H	−3.96441	−1.73560	+0.69551
H	−2.85280	−1.18051	+1.91874
H	−4.98114	−0.14582	−0.70896
H	−4.54217	+2.81117	−0.36009
H	−5.11587	+1.94673	−1.76113
H	−2.34072	+2.62389	−1.04040
H	−2.88066	+1.90587	−2.54325
H	−2.79762	+2.26826	+1.14500
H	−1.62048	+1.03933	+1.60659
H	−3.20493	+1.06853	+2.36584
H	−6.61306	+1.08254	+0.63216
H	−5.35006	+1.70944	+1.67640
H	−5.71381	−0.01525	+1.66856
H	−2.87919	−0.28793	−1.64578
O	−7.29969	−3.95094	−0.34856
H	−6.71239	−4.39626	−1.00799
C	−7.11328	−4.57204	+0.92246
H	−7.28809	−5.68023	+0.85176
H	−7.87002	−4.15414	+1.62062
C	−5.71326	−4.30747	+1.48416
H	−5.46438	−3.21623	+1.43320
O	−5.81369	−4.69867	+2.91396
C	−4.57701	−4.46151	+3.65572
O	−4.22991	−3.04934	+3.53156
H	−4.79117	−4.72341	+4.72654
C	−3.47617	−5.33356	+3.06088
O	−2.26017	−5.08512	+3.80140
H	−1.48974	−5.41790	+3.26896
H	−3.76690	−6.41668	+3.17618
C	−3.27645	−5.01317	+1.58175
O	−2.31416	−5.95408	+1.04684
H	−2.33284	−5.87388	+0.04705

H	−2.87900	−3.96706	+1.48259
C	−4.59861	−5.11828	+0.80942
H	−4.90160	−6.19339	+0.70234
O	−4.35571	−4.54020	−0.53344
C	−4.54508	−5.45237	−1.65520
O	−5.66096	−4.96249	−2.46250
C	−5.44237	−3.58143	−2.96574
C	−6.74802	−3.22848	−3.68397
O	−6.83999	−1.83001	−3.95386
H	−6.16729	−1.59841	−4.64078
H	−6.82852	−3.82164	−4.63506
H	−7.60602	−3.50874	−3.03604
H	−5.27871	−2.90739	−2.08676
H	−4.84040	−6.47213	−1.28853
C	−3.29267	−5.50132	−2.52411
O	−2.19635	−5.95998	−1.70320
H	−1.34497	−5.76907	−2.18023
H	−3.46387	−6.23980	−3.35929
C	−2.97609	−4.13281	−3.12629
O	−1.87165	−4.30682	−4.04559
H	−1.81598	−3.48528	−4.61885
H	−2.67960	−3.41640	−2.30926
C	−4.19885	−3.57231	−3.86529
H	−4.38919	−4.16419	−4.79954
O	−3.88528	−2.17083	−4.22937
C	−3.90835	−1.87922	−5.65841
O	−4.96969	−0.90739	−5.91418
C	−4.77989	+0.37434	−5.18625
C	−6.03229	+1.18548	−5.53100
O	−6.15510	+2.34545	−4.70888
H	−5.45688	+2.99253	−4.97520
H	−6.00849	+1.47138	−6.61700
H	−6.93022	+0.55190	−5.36652
H	−4.73529	+0.15674	−4.08868
H	−4.17689	−2.80030	−6.24262
C	−2.57680	−1.29094	−6.11327
O	−1.53854	−2.25035	−5.82467
H	−0.65831	−1.79035	−5.88598
H	−2.62405	−1.12159	−7.22797
C	−2.28865	+0.04206	−5.42411

O	−1.08113	+0.58285	−6.01206
H	−1.03045	+1.55690	−5.77848
H	−2.13722	−0.11713	−4.32013
C	−3.45949	+1.00837	−5.64441
H	−3.52165	+1.29751	−6.72699
O	−3.19085	+2.21681	−4.83226
C	−3.07148	+3.46277	−5.58381
O	−4.16027	+4.34980	−5.18127
C	−4.16322	+4.63374	−3.72276
C	−5.39513	+5.51977	−3.51354
O	−5.75865	+5.60421	−2.13609
H	−5.05365	+6.10071	−1.65190
H	−5.20128	+6.54337	−3.93593
H	−6.25443	+5.08308	−4.06659
H	−4.27672	+3.66424	−3.17469
H	−3.20366	+3.27365	−6.68292
C	−1.74479	+4.15091	−5.28032
O	−0.67548	+3.27267	−5.69186
H	+0.17644	+3.61329	−5.30781
H	−1.69087	+5.10522	−5.87859
C	−1.62386	+4.48308	−3.79462
O	−0.41073	+5.25399	−3.61405
H	−0.43702	+5.65382	−2.69376
H	−1.55804	+3.53219	−3.19562
C	−2.83769	+5.29764	−3.32569
H	−2.78200	+6.34015	−3.73778
O	−2.77686	+5.35042	−1.84823
C	−2.66166	+6.68357	−1.27143
O	−3.85323	+6.93582	−0.46322
C	−4.04656	+5.92315	+0.60700
C	−5.36861	+6.32287	+1.26851
O	−5.87548	+5.28482	+2.10651
H	−5.28495	+5.20151	+2.89533
H	−5.22661	+7.27114	+1.85452
H	−6.12313	+6.52307	+0.47769
H	−4.13195	+4.91331	+0.12852
H	−2.65009	+7.46556	−2.07738
C	−1.43255	+6.76532	−0.37383
O	−0.26467	+6.51985	−1.18608
H	+0.50490	+6.33260	−0.58356

H	−1.36937	+7.80399	+0.06129
C	−1.52432	+5.75071	+0.76463
O	−0.38819	+5.96708	+1.63451
H	−0.57341	+5.50919	+2.50760
H	−1.49421	+4.70401	+0.35025
C	−2.83093	+5.95205	+1.54365
H	−2.79595	+6.92293	+2.10578
O	−2.94157	+4.83382	+2.50693
C	−3.00378	+5.22521	+3.91060
O	−4.29774	+4.80821	+4.44623
C	−4.52721	+3.34541	+4.32567
C	−5.94870	+3.14338	+4.85877
O	−6.46004	+1.85390	+4.52354
H	−5.96693	+1.17609	+5.04812
H	−5.96079	+3.29707	+5.97177
H	−6.61761	+3.90547	+4.40422
H	−4.46703	+3.06447	+3.24251
H	−2.96309	+6.34312	+4.01097
C	−1.89896	+4.54195	+4.70873
O	−0.62782	+4.95956	+4.16708
H	+0.08399	+4.37142	+4.53710
H	−1.97134	+4.87847	+5.78286
C	−2.03664	+3.02124	+4.65272
O	−1.03010	+2.46415	+5.53187
H	−1.26784	+1.50521	+5.70590
H	−1.86550	+2.65891	+3.60117
C	−3.43976	+2.60267	+5.11421
H	−3.55510	+2.79682	+6.21362
O	−3.57757	+1.15047	+4.85794
C	−3.83395	+0.33348	+6.03961
O	−5.14918	−0.28657	+5.89831
H	−3.88518	+0.97706	+6.95866
C	−2.78076	−0.76079	+6.17374
O	−1.49230	−0.12279	+6.29265
H	−0.77982	−0.80301	+6.15280
H	−2.99092	−1.34871	+7.11351
C	−2.81224	−1.71154	+4.97765
O	−1.87373	−2.77995	+5.24614
H	−2.08045	−3.54218	+4.62711
H	−2.50880	−1.16385	+4.04091

C	−4.22586	−2.28067	+4.79656
H	−4.47654	−2.96742	+5.64808
C	−5.26197	−1.15267	+4.69639
H	−5.05543	−0.52935	+3.78840
C	−6.71990	−1.62223	+4.71224
H	−6.87366	−2.34879	+5.55516
H	−7.37936	−0.74663	+4.89493
O	−7.10115	−2.20761	+3.46803
H	−6.64403	−3.07980	+3.37883
O	+5.86831	−6.00095	−0.56529
H	+5.12434	−6.33938	−0.00992
C	+5.51550	−6.13436	−1.94020
H	+5.29383	−7.20917	−2.19072
H	+6.39269	−5.82266	−2.54798
C	+4.31234	−5.26219	−2.31669
H	+4.45295	−4.21027	−1.94712
O	+4.34253	−5.25806	−3.80837
C	+3.24603	−4.53387	−4.44093
O	+3.33324	−3.12198	−4.06394
H	+3.39663	−4.64708	−5.54873
C	+1.91835	−5.13294	−3.99211
O	+0.84957	−4.39295	−4.61948
H	−0.00302	−4.62191	−4.16082
H	+1.87611	−6.20618	−4.33755
C	+1.78213	−5.09412	−2.47290
O	+0.54247	−5.75887	−2.12915
H	+0.56443	−5.96804	−1.14817
H	+1.75324	−4.02596	−2.11593
C	+2.96874	−5.81763	−1.82444
H	+2.89988	−6.92030	−2.02557
O	+2.87544	−5.58073	−0.36669
C	+2.72432	−6.77326	+0.45763
O	+3.89396	−6.88119	+1.32698
C	+4.09784	−5.66788	+2.15953
C	+5.39115	−5.94892	+2.93015
O	+5.93156	−4.76108	+3.50801
H	+5.32026	−4.44739	+4.21899
H	+5.19947	−6.72357	+3.72225
H	+6.14795	−6.36160	+2.22916
H	+4.22835	−4.79005	+1.47658

H	+2.70929	−7.69830	−0.17891
C	+1.48253	−6.65546	+1.33329
O	+0.32630	−6.58249	+0.47207
H	−0.46053	−6.32007	+1.02143
H	+1.40493	−7.57630	+1.97930
C	+1.56989	−5.41947	+2.22682
O	+0.41647	−5.43222	+3.10198
H	+0.58817	−4.78399	+3.84834
H	+1.55592	−4.48818	+1.59288
C	+2.86554	−5.45089	+3.05006
H	+2.80255	−6.25221	+3.83348
O	+2.99423	−4.13145	+3.70958
C	+3.04140	−4.16200	+5.16635
O	+4.33066	−3.62532	+5.59575
C	+4.56911	−2.24014	+5.11408
C	+5.98811	−1.91691	+5.59091
O	+6.50554	−0.74750	+4.95798
H	+6.01298	+0.03846	+5.30042
H	+5.99279	−1.79764	+6.70826
H	+6.65674	−2.76751	+5.33785
H	+4.51564	−2.24022	+3.99541
H	+2.99497	−5.21941	+5.54187
C	+1.92970	−3.29894	+5.75237
O	+0.66598	−3.84089	+5.31368
H	−0.04878	−3.17055	+5.48376
H	+1.98469	−3.35338	+6.87776
C	+2.07978	−1.84159	+5.31741
O	+1.06458	−1.07599	+6.00816
H	+1.31853	−0.10635	+5.96287
H	+1.93179	−1.75409	+4.20389
C	+3.47719	−1.32440	+5.68535
H	+3.57668	−1.24919	+6.80072
O	+3.61682	+0.02519	+5.09123
C	+3.87888	+1.10049	+6.04321
O	+5.19375	+1.66654	+5.75196
C	+5.30329	+2.20117	+4.36998
C	+6.76120	+2.65702	+4.25919
O	+7.13018	+2.92829	+2.90787
H	+6.63906	+3.73011	+2.60242
H	+6.92180	+3.56386	+4.90346

H	+7.42383	+1.85007	+4.63910
H	+5.09047	+1.36941	+3.65126
H	+3.93288	+0.69461	+7.08911
C	+2.83034	+2.20078	+5.91626
O	+1.53556	+1.61812	+6.18185
H	+0.83266	+2.24335	+5.86037
H	+3.04527	+2.99271	+6.69000
C	+2.85792	+2.83774	+4.52825
O	+1.93303	+3.95125	+4.54169
H	+2.10593	+4.50517	+3.72287
H	+2.53166	+2.08361	+3.75942
C	+4.27241	+3.32266	+4.18241
H	+4.54163	+4.21216	+4.81143
O	+4.26211	+3.71637	+2.75469
C	+4.56859	+5.11714	+2.48946
O	+5.81025	+5.18026	+1.72082
C	+5.73398	+4.45269	+0.42687
C	+7.14330	+4.58786	−0.15743
O	+7.34959	+3.70323	−1.25782
H	+6.79993	+4.01397	−2.01841
H	+7.32187	+5.65322	−0.46723
H	+7.88647	+4.33458	+0.62882
H	+5.49094	+3.38038	+0.63727
H	+4.76202	+5.66676	+3.44979
C	+3.45348	+5.76763	+1.67792
O	+2.23345	+5.68698	+2.44441
H	+1.46686	+5.87948	+1.84006
H	+3.71590	+6.85260	+1.51359
C	+3.28699	+5.08639	+0.32052
O	+2.30302	+5.84003	−0.42848
H	+2.38164	+5.58010	−1.39394
H	+2.92978	+4.02876	+0.46129
C	+4.62468	+5.07746	−0.43069
H	+4.90461	+6.12229	−0.73018
O	+4.43473	+4.25554	−1.64747
C	+4.66473	+4.94413	−2.91219
O	+5.79511	+4.30457	−3.58357
C	+5.58265	+2.85084	−3.80243
C	+6.89356	+2.36946	−4.43074
O	+6.99050	+0.94521	−4.42693

H	+6.31285	+0.58088	−5.04799
H	+6.97948	+2.76899	−5.47766
H	+7.74693	+2.77092	−3.84353
H	+5.41747	+2.36471	−2.80751
H	+4.96133	+6.01244	−2.73369
C	+3.44018	+4.83333	−3.81473
O	+2.32838	+5.46989	−3.14828
H	+1.48916	+5.20197	−3.60958
H	+3.65306	+5.37481	−4.78058
C	+3.11729	+3.37171	−4.11606
O	+2.03545	+3.34594	−5.07785
H	+1.96122	+2.40889	−5.42914
H	+2.79443	+2.85509	−3.16910
C	+4.34434	+2.64756	−4.68633
H	+4.54687	+2.99410	−5.73418
O	+4.01622	+1.20332	−4.70214
C	+4.01869	+0.56994	−6.01440
O	+5.07489	−0.44334	−6.02617
H	+4.27787	+1.31398	−6.81429
C	+2.68132	−0.11396	−6.27909
O	+1.64978	+0.89469	−6.25023
H	+0.76646	+0.44008	−6.18826
H	+2.71412	−0.58079	−7.30556
C	+2.39761	−1.20658	−5.24686
O	+1.19272	−1.89137	−5.66110
H	+1.14918	−2.76690	−5.17278
H	+2.24630	−0.74614	−4.23008
C	+3.57381	−2.19035	−5.18993
H	+3.63332	−2.77343	−6.14657
C	+4.88934	−1.44105	−4.94430
H	+4.83222	−0.89073	−3.96861
C	+6.13992	−2.32037	−4.98278
H	+6.12585	−2.97396	−5.89331
H	+7.04118	−1.67281	−5.03833
O	+6.23362	−3.11226	−3.79612
H	+5.60757	−3.87529	−3.87630

S3.6. Cartesian Coordinates of encapsulated TS Conformers

345

* E = 0.000 kcal/mol

C	−0.23192	+0.73539	+0.49256
C	−0.96201	−0.45595	+0.30810
C	−0.37072	−1.67088	−0.00353
C	+0.96781	−2.01398	−0.21009
C	+2.14039	−1.30768	−0.50414
C	+2.22790	−0.08574	−1.17882
C	+1.10810	+0.51031	−1.76384
H	+0.32678	+0.95135	−0.70552
C	+3.54138	+0.63740	−1.31239
C	+4.66625	+0.05052	−0.48725
C	+4.68521	−1.45322	−0.66557
C	+3.40869	−2.03445	−0.10840
C	−0.99642	+1.91749	+1.02360
C	−2.29341	+2.20830	+0.26041
C	−2.96786	+0.93821	−0.26690
C	−2.43829	−0.29519	+0.48061
C	−4.46378	+0.81374	+0.04370
C	−4.74376	−0.68461	−0.13095
C	−3.40097	−1.40475	+0.09211
O	+4.74766	−1.79872	−2.03435
H	+5.63744	−1.68027	−2.36918
C	−2.70956	+0.80564	−1.76174
C	−5.39094	+1.71259	−0.74188
H	+5.52797	−1.89412	−0.13413
H	+3.49118	−2.04239	+0.97931
H	+3.33393	−3.07721	−0.42038
H	+4.52630	+0.27270	+0.57145
H	+5.61668	+0.49027	−0.78470
H	+3.82712	+0.62090	−2.36539
H	+3.39169	+1.68736	−1.05488
H	+0.31297	−0.15332	−2.08924
H	+1.27825	+1.36877	−2.40365
H	+1.14539	−3.07665	−0.06971
H	−1.03923	−2.52332	+0.01838
H	+0.76945	+0.60247	+0.90172
H	−0.36237	+2.80261	+1.03796

H	−1.24170	+1.71233	+2.07074
H	−2.97400	+2.74971	+0.91904
H	−2.07980	+2.87772	−0.57357
H	−4.57058	+1.05888	+1.10425
H	−5.11960	−0.87867	−1.13548
H	−5.51353	−1.02608	+0.55611
H	−3.06711	−1.89889	−0.82094
H	−3.46209	−2.17423	+0.85940
H	−3.16455	−0.08980	−2.18263
H	−1.64143	+0.76410	−1.96346
H	−3.10951	+1.66620	−2.29621
H	−6.41761	+1.59453	−0.40435
H	−5.36803	+1.47687	−1.80486
H	−5.12398	+2.76262	−0.62179
H	−2.60752	−0.07706	+1.54393
O	−7.12254	−4.14275	−0.65287
H	−6.53040	−4.47213	−1.37327
C	−6.86800	−4.90119	+0.52884
H	−6.97692	−6.00126	+0.32495
H	−7.63183	−4.62039	+1.28558
C	−5.47387	−4.61823	+1.09651
H	−5.29239	−3.51542	+1.16948
O	−5.51880	−5.17750	+2.47210
C	−4.28044	−4.94991	+3.21339
O	−4.02905	−3.51327	+3.25461
H	−4.44922	−5.35151	+4.24856
C	−3.14326	−5.67222	+2.49953
O	−1.92861	−5.44755	+3.24706
H	−1.15427	−5.71137	+2.68082
H	−3.37060	−6.77667	+2.48569
C	−2.99240	−5.17326	+1.06395
O	−1.99609	−6.00009	+0.41648
H	−2.04530	−5.82438	−0.57067
H	−2.65006	−4.10054	+1.06697
C	−4.32927	−5.27844	+0.31587
H	−4.56793	−6.35509	+0.10723
O	−4.16783	−4.55505	−0.96489
C	−4.34096	−5.36155	−2.16652
O	−5.49480	−4.84563	−2.90098
C	−5.34931	−3.41320	−3.26859

C	−6.67976	−3.06073	−3.94010
O	−6.85056	−1.64923	−4.06564
H	−6.20925	−1.31538	−4.74047
H	−6.73958	−3.55767	−4.94610
H	−7.51369	−3.45219	−3.31869
H	−5.20977	−2.81831	−2.32946
H	−4.58301	−6.42502	−1.89842
C	−3.10599	−5.26202	−3.05436
O	−1.97867	−5.77269	−2.31214
H	−1.14093	−5.50135	−2.77717
H	−3.26691	−5.90120	−3.96995
C	−2.86057	−3.81851	−3.49175
O	−1.75369	−3.82964	−4.42284
H	−1.75818	−2.96071	−4.92395
H	−2.60278	−3.18338	−2.59748
C	−4.11694	−3.25568	−4.16977
H	−4.28550	−3.77102	−5.15249
O	−3.87799	−1.81425	−4.40966
C	−3.95357	−1.39566	−5.80488
O	−5.05963	−0.45011	−5.94131
C	−4.90737	+0.74457	−5.07220
C	−6.19530	+1.54080	−5.30073
O	−6.35702	+2.57354	−4.32887
H	−5.67748	+3.27120	−4.49873
H	−6.19430	+1.97109	−6.33852
H	−7.06535	+0.85452	−5.21901
H	−4.83544	+0.40322	−4.00746
H	−4.19975	−2.26951	−6.46603
C	−2.66090	−0.70809	−6.23172
O	−1.58061	−1.65179	−6.07357
H	−0.71656	−1.15907	−6.10159
H	−2.74812	−0.42319	−7.31983
C	−2.40426	+0.55198	−5.40673
O	−1.24557	+1.21224	−5.97065
H	−1.20902	+2.14283	−5.59849
H	−2.19907	+0.27361	−4.33618
C	−3.62211	+1.48486	−5.46585
H	−3.72269	+1.92196	−6.49460
O	−3.38790	+2.57635	−4.49261
C	−3.32551	+3.91867	−5.05898

O	−4.44189	+4.69628	−4.52449
C	−4.42858	+4.78392	−3.04110
C	−5.70574	+5.55765	−2.70073
O	−6.02825	+5.46127	−1.31394
H	−5.36428	+5.98411	−0.80061
H	−5.58974	+6.63258	−3.00641
H	−6.55264	+5.13019	−3.27938
H	−4.47099	+3.74490	−2.62433
H	−3.46583	+3.88429	−6.17269
C	−2.01957	+4.60309	−4.66999
O	−0.92880	+3.82329	−5.20230
H	−0.08365	+4.12218	−4.77062
H	−2.00045	+5.63155	−5.13329
C	−1.90083	+4.73883	−3.15276
O	−0.70128	+5.49970	−2.87249
H	−0.75485	+5.82637	−1.92535
H	−1.82448	+3.71946	−2.67895
C	−3.12949	+5.46662	−2.59201
H	−3.12044	+6.54182	−2.91367
O	−3.03842	+5.39703	−1.11617
C	−2.99154	+6.68353	−0.43037
O	−4.18374	+6.80133	+0.40625
C	−4.30860	+5.69083	+1.38498
C	−5.64112	+5.95931	+2.09096
O	−6.08524	+4.82437	+2.83292
H	−5.47540	+4.69043	+3.59979
H	−5.53919	+6.85587	+2.76104
H	−6.41490	+6.19290	+1.32845
H	−4.34595	+4.72373	+0.82023
H	−3.03476	+7.52784	−1.16959
C	−1.75713	+6.76744	+0.46115
O	−0.58909	+6.64534	−0.37984
H	+0.19578	+6.44359	+0.19615
H	−1.74497	+7.77411	+0.96894
C	−1.77274	+5.66827	+1.52153
O	−0.65281	+5.89833	+2.41117
H	−0.78614	+5.32719	+3.22535
H	−1.66135	+4.66482	+1.02629
C	−3.08720	+5.69886	+2.31398
H	−3.11118	+6.59881	+2.98401

O	−3.12308	+4.47114	+3.14111
C	−3.18008	+4.68992	+4.58188
O	−4.44658	+4.15634	+5.07886
C	−4.61738	+2.70855	+4.78988
C	−6.02071	+2.38428	+5.31055
O	−6.47759	+1.11553	+4.84414
H	−5.94426	+0.40871	+5.28434
H	−6.02348	+2.41804	+6.43375
H	−6.72997	+3.15837	+4.94653
H	−4.56067	+2.56009	+3.68145
H	−3.18458	+5.78872	+4.81437
C	−2.03290	+3.96540	+5.27683
O	−0.79093	+4.49476	+4.76543
H	−0.05479	+3.87969	+5.02915
H	−2.09484	+4.17159	+6.38408
C	−2.11236	+2.45708	+5.04638
O	−1.06952	+1.84107	+5.83838
H	−1.27300	+0.86092	+5.91208
H	−1.94833	+2.22574	+3.95550
C	−3.48751	+1.92929	+5.47657
H	−3.59158	+1.99545	+6.59210
O	−3.56373	+0.51046	+5.06190
C	−3.76252	−0.44468	+6.14714
O	−5.04885	−1.10784	+5.94748
H	−3.83056	+0.08878	+7.13324
C	−2.65922	−1.49682	+6.14594
O	−1.39588	−0.82571	+6.33545
H	−0.66380	−1.45866	+6.10362
H	−2.83585	−2.20247	+7.00825
C	−2.65818	−2.29495	+4.84343
O	−1.66702	−3.34132	+4.97601
H	−1.83557	−4.02281	+4.25891
H	−2.39109	−1.62379	+3.98003
C	−4.04491	−2.90634	+4.60392
H	−4.24986	−3.70372	+5.36673
C	−5.13805	−1.82999	+4.65153
H	−4.97939	−1.09294	+3.82382
C	−6.56917	−2.37528	+4.62842
H	−6.67249	−3.19450	+5.39046
H	−7.27100	−1.56078	+4.90950

O	−6.93732	−2.84488	+3.33239
H	−6.42621	−3.66915	+3.14009
O	+6.06766	−5.41636	−1.25900
H	+5.40154	−5.92088	−0.73047
C	+5.72155	−5.53113	−2.64024
H	+5.60202	−6.61142	−2.92689
H	+6.56218	−5.11582	−3.23778
C	+4.44144	−4.75925	−2.97379
H	+4.48159	−3.71085	−2.56531
O	+4.44990	−4.69077	−4.46312
C	+3.31335	−3.95303	−5.01179
O	+3.34701	−2.59723	−4.48677
H	+3.44638	−3.95002	−6.12802
C	+2.02000	−4.64509	−4.59509
O	+0.91550	−3.89153	−5.14141
H	+0.08419	−4.16151	−4.66728
H	+2.00818	−5.68603	−5.03052
C	+1.91390	−4.73936	−3.07413
O	+0.72202	−5.50470	−2.76617
H	+0.78464	−5.80152	−1.81068
H	+1.83204	−3.71128	−2.62630
C	+3.15172	−5.44724	−2.50935
H	+3.14799	−6.52903	−2.81092
O	+3.07249	−5.35510	−1.03179
C	+3.03214	−6.62904	−0.32650
O	+4.23514	−6.73883	+0.49919
C	+4.37203	−5.61862	+1.46391
C	+5.71196	−5.87896	+2.15821
O	+6.16251	−4.73438	+2.88309
H	+5.56293	−4.59693	+3.65743
H	+5.61882	−6.76729	+2.83995
H	+6.47809	−6.12009	+1.39039
H	+4.40447	−4.65831	+0.88807
H	+3.06485	−7.48572	−1.05185
C	+1.80987	−6.69672	+0.58273
O	+0.63092	−6.59059	−0.24333
H	−0.15044	−6.40089	+0.34192
H	+1.80544	−7.69420	+1.10934
C	+1.84027	−5.58009	+1.62481
O	+0.72524	−5.79028	+2.52440

H	+0.86563	−5.20786	+3.32911
H	+1.73480	−4.58104	+1.11607
C	+3.16091	−5.61191	+2.40631
H	+3.18658	−6.50609	+3.08402
O	+3.21346	−4.37735	+3.22239
C	+3.27898	−4.58239	+4.66367
O	+4.54998	−4.04282	+5.14564
C	+4.71873	−2.60098	+4.82985
C	+6.12560	−2.26705	+5.33413
O	+6.57992	−1.00821	+4.83765
H	+6.05269	−0.29231	+5.27043
H	+6.13678	−2.27664	+6.45755
H	+6.83200	−3.04894	+4.98144
H	+4.65449	−2.47256	+3.71925
H	+3.28497	−5.67842	+4.90831
C	+2.13866	−3.84775	+5.35967
O	+0.89165	−4.38857	+4.87465
H	+0.15832	−3.76326	+5.12109
H	+2.21553	−4.03263	+6.46993
C	+2.21465	−2.34420	+5.09910
O	+1.18006	−1.71309	+5.89075
H	+1.38365	−0.73216	+5.94322
H	+2.04083	−2.13359	+4.00735
C	+3.59319	−1.80869	+5.50867
H	+3.70532	−1.85158	+6.62455
O	+3.66647	−0.39820	+5.06462
C	+3.87488	+0.57896	+6.12690
O	+5.15847	+1.24015	+5.89808
C	+5.23246	+1.92106	+4.57964
C	+6.66241	+2.46617	+4.51966
O	+7.01294	+2.88871	+3.20229
H	+6.49499	+3.70263	+2.98580
H	+6.77451	+3.31277	+5.24974
H	+7.36899	+1.66315	+4.82076
H	+5.06403	+1.15817	+3.77755
H	+3.95417	+0.06797	+7.12382
C	+2.77142	+1.63116	+6.11263
O	+1.50998	+0.96703	+6.33572
H	+0.77641	+1.59172	+6.08822
H	+2.95945	+2.36043	+6.95255

C	+2.75453	+2.39171	+4.78804
O	+1.76571	+3.44274	+4.90157
H	+1.92706	+4.10229	+4.16320
H	+2.47553	+1.69559	+3.94856
C	+4.13788	+2.99470	+4.51100
H	+4.35209	+3.81733	+5.24376
O	+4.10439	+3.55613	+3.14202
C	+4.35404	+4.99006	+3.04941
O	+5.58198	+5.19323	+2.28313
C	+5.51544	+4.60051	+0.92243
C	+6.90642	+4.85348	+0.33343
O	+7.13414	+4.07230	−0.83925
H	+6.56006	+4.42069	−1.56528
H	+7.02941	+5.94772	+0.11018
H	+7.67462	+4.57267	+1.08564
H	+5.32195	+3.50246	+1.02461
H	+4.53779	+5.42627	+4.06780
C	+3.20613	+5.68781	+2.32824
O	+2.00094	+5.48551	+3.09590
H	+1.22163	+5.75510	+2.53886
H	+3.43115	+6.79173	+2.27685
C	+3.04068	+5.14535	+0.90974
O	+2.01832	+5.93540	+0.25703
H	+2.08311	+5.77224	−0.73117
H	+2.72189	+4.06621	+0.94665
C	+4.36645	+5.25720	+0.14506
H	+4.60061	+6.33494	−0.06248
O	+4.19233	+4.53851	−1.13653
C	+4.37411	+5.34748	−2.33647
O	+5.51661	+4.81670	−3.07729
C	+5.34951	+3.38506	−3.43708
C	+6.66816	+3.00863	−4.11832
O	+6.80880	+1.59346	−4.24607
H	+6.15741	+1.27388	−4.91904
H	+6.73040	+3.50464	−5.12421
H	+7.51439	+3.38256	−3.50272
H	+5.21020	+2.79794	−2.49346
H	+4.63264	+6.40606	−2.06519
C	+3.13515	+5.26910	−3.22131
O	+2.01750	+5.79925	−2.47700

H	+1.17590	+5.53249	−2.93566
H	+3.30424	+5.90385	−4.13800
C	+2.86397	+3.82911	−3.65253
O	+1.75736	+3.85204	−4.58453
H	+1.73092	+2.96580	−5.05591
H	+2.59266	+3.20406	−2.75503
C	+4.10784	+3.23379	−4.32628
H	+4.27772	+3.72322	−5.32181
O	+3.84403	+1.79244	−4.52698
C	+3.86633	+1.33808	−5.91393
O	+4.96722	+0.38957	−6.06432
H	+4.08562	+2.19557	−6.60557
C	+2.55775	+0.64007	−6.26749
O	+1.48524	+1.59259	−6.10107
H	+0.61997	+1.10101	−6.11655
H	+2.60250	+0.32032	−7.34834
C	+2.33173	−0.59167	−5.39173
O	+1.14532	−1.26272	−5.88130
H	+1.14168	−2.19388	−5.50500
H	+2.17940	−0.28156	−4.32064
C	+3.54387	−1.52884	−5.48391
H	+3.60642	−1.98009	−6.51048
C	+4.83833	−0.77595	−5.15016
H	+4.78823	−0.39505	−4.09799
C	+6.12114	−1.58084	−5.37126
H	+6.07878	−2.10794	−6.36148
H	+6.98913	−0.88690	−5.39355
O	+6.33592	−2.51963	−4.31372
H	+5.65237	−3.23205	−4.39548

345

* E = 0.048 kcal/mol

C	−0.58633	−0.60826	+0.16643
C	−0.83320	+0.39509	−0.79942
C	+0.19291	+0.98416	−1.52327
C	+1.57867	+0.79803	−1.48712
C	+2.48896	+0.30378	−0.54884
C	+2.31620	+0.28314	+0.84236
C	+1.20279	+0.85662	+1.44998
H	+0.12164	+0.03090	+1.05302
C	+3.34240	−0.35201	+1.74358

C	+4.39528	−1.16497	+1.02204
C	+4.92115	−0.37298	−0.15688
C	+3.80264	−0.15381	−1.14586
C	−1.71564	−1.35598	+0.85584
C	−3.12448	−0.91015	+0.46529
C	−3.16054	+0.57268	+0.16396
C	−2.25141	+0.81689	−1.03729
C	−4.48609	+1.10467	−0.38742
C	−4.07147	+2.39352	−1.11852
C	−2.57454	+2.24604	−1.45036
O	+5.38251	+0.89923	+0.25061
H	+6.24554	+0.82601	+0.65934
C	−2.72780	+1.38053	+1.38176
C	−5.60081	+1.30086	+0.61507
H	+5.72392	−0.91435	−0.65654
H	+3.64150	−1.09155	−1.67989
H	+4.13053	+0.57258	−1.89039
H	+3.97501	−2.10073	+0.65111
H	+5.20095	−1.42417	+1.70677
H	+3.83502	+0.44759	+2.29932
H	+2.82982	−0.96704	+2.48442
H	+0.71284	+1.68093	+0.94025
H	+1.19521	+0.91299	+2.53239
H	+2.05671	+1.09202	−2.41774
H	−0.13580	+1.63033	−2.32943
H	+0.28554	−1.22158	−0.06498
H	−1.59308	−1.24787	+1.93542
H	−1.60918	−2.42333	+0.66173
H	−3.44686	−1.44382	−0.43165
H	−3.82625	−1.17572	+1.25769
H	−4.81940	+0.37828	−1.13396
H	−4.23903	+3.25736	−0.47506
H	−4.67481	+2.55155	−2.00876
H	−1.96941	+2.94553	−0.87272
H	−2.36019	+2.43750	−2.49998
H	−2.83920	+2.45122	+1.21740
H	−1.68816	+1.19713	+1.64570
H	−3.34075	+1.11707	+2.24292
H	−6.51300	+1.62020	+0.11733
H	−5.34436	+2.06475	+1.34777

H	−5.82425	+0.37983	+1.15257
H	−2.64368	+0.16304	−1.82710
O	−7.38900	−2.55648	−2.42441
H	−6.76641	−2.84070	−3.13820
C	−7.45333	−3.58842	−1.44108
H	−7.70181	−4.57523	−1.91784
H	−8.27376	−3.33726	−0.73497
C	−6.14178	−3.71061	−0.65960
H	−5.80784	−2.70979	−0.28427
O	−6.49135	−4.55769	+0.51020
C	−5.37445	−4.73254	+1.43581
O	−4.94298	−3.41420	+1.88706
H	−5.76589	−5.33645	+2.29802
C	−4.23979	−5.44557	+0.70863
O	−3.14194	−5.60333	+1.63313
H	−2.32666	−5.84203	+1.11572
H	−4.60193	−6.46413	+0.38708
C	−3.79658	−4.66086	−0.52497
O	−2.81839	−5.46622	−1.22531
H	−2.71140	−5.08894	−2.14929
H	−3.33010	−3.68524	−0.21111
C	−4.99940	−4.37966	−1.43578
H	−5.35504	−5.33412	−1.90702
O	−4.53970	−3.45296	−2.49441
C	−4.65418	−3.95952	−3.85778
O	−5.59654	−3.11256	−4.58552
C	−5.18172	−1.68612	−4.62946
C	−6.33120	−0.98565	−5.36006
O	−6.25308	+0.43339	−5.23331
H	−5.47629	+0.75272	−5.75517
H	−6.32917	−1.28735	−6.44247
H	−7.29575	−1.31618	−4.91814
H	−5.09147	−1.31119	−3.57815
H	−5.09122	−4.99416	−3.85842
C	−3.30295	−3.91223	−4.56184
O	−2.38192	−4.74681	−3.82823
H	−1.45750	−4.53726	−4.13100
H	−3.42647	−4.32563	−5.60417
C	−2.77910	−2.48021	−4.65403
O	−1.56181	−2.51336	−5.43600

H	−1.35364	−1.57584	−5.72726
H	−2.56001	−2.08252	−3.62387
C	−3.82244	−1.58440	−5.33486
H	−3.92328	−1.86688	−6.41640
O	−3.33599	−0.19039	−5.23711
C	−3.11140	+0.48159	−6.51228
O	−4.03184	+1.61286	−6.60313
C	−3.86478	+2.58465	−5.49182
C	−4.95602	+3.62988	−5.74195
O	−5.15742	+4.46801	−4.60475
H	−4.36221	+5.04540	−4.49419
H	−4.69064	+4.24418	−6.64472
H	−5.91393	+3.10903	−5.95658
H	−4.04157	+2.05025	−4.52344
H	−3.36163	−0.20161	−7.36791
C	−1.68151	+1.00324	−6.60004
O	−0.78453	−0.12366	−6.51110
H	+0.13545	+0.21557	−6.34087
H	−1.54401	+1.50753	−7.59963
C	−1.39480	+2.01220	−5.48971
O	−0.07107	+2.54982	−5.72069
H	+0.03282	+3.37282	−5.15539
H	−1.42845	+1.50197	−4.48616
C	−2.43456	+3.14067	−5.51983
H	−2.28103	+3.78206	−6.42784
O	−2.22418	+3.95532	−4.30333
C	−1.88321	+5.35471	−4.53339
O	−2.95310	+6.18134	−3.97930
C	−3.18131	+5.92356	−2.53365
C	−4.37512	+6.81321	−2.17479
O	−4.95017	+6.44467	−0.92139
H	−4.30892	+6.67100	−0.20313
H	−4.05494	+7.89046	−2.16212
H	−5.15815	+6.70206	−2.95542
H	−3.44102	+4.84213	−2.40424
H	−1.84332	+5.57643	−5.63362
C	−0.57695	+5.70536	−3.82997
O	+0.46861	+4.88279	−4.39112
H	+1.26121	+4.92093	−3.79011
H	−0.34080	+6.79042	−4.02691

C	−0.68887	+5.48244	−2.32269
O	+0.53191	+5.97223	−1.71898
H	+0.37869	+6.04566	−0.73030
H	−0.80560	+4.38408	−2.10634
C	−1.89677	+6.24320	−1.75736
H	−1.69567	+7.34727	−1.77343
O	−2.07491	+5.79902	−0.35602
C	−1.97267	+6.85052	+0.64814
O	−3.26319	+6.96572	+1.32541
C	−3.68702	+5.70346	+1.98451
C	−5.07881	+6.02154	+2.53854
O	−5.76354	+4.84208	+2.95907
H	−5.32399	+4.50113	+3.77636
H	−4.98878	+6.75066	+3.38840
H	−5.68430	+6.50335	+1.74107
H	−3.74222	+4.89730	+1.20765
H	−1.78051	+7.84428	+0.16138
C	−0.90172	+6.50540	+1.67705
O	+0.35753	+6.37802	+0.98487
H	+1.01010	+5.92560	+1.58541
H	−0.83192	+7.35092	+2.42102
C	−1.24634	+5.21705	+2.42328
O	−0.25160	+5.03654	+3.45864
H	−0.60686	+4.37266	+4.12126
H	−1.22377	+4.34189	+1.71504
C	−2.64382	+5.33098	+3.04681
H	−2.63642	+6.09541	+3.86853
O	−2.98240	+4.00685	+3.61591
C	−3.23348	+3.99254	+5.05282
O	−4.62442	+3.60224	+5.27406
C	−4.95168	+2.27163	+4.70034
C	−6.44696	+2.10066	+4.98528
O	−7.01148	+1.02854	+4.23163
H	−6.64925	+0.17591	+4.57723
H	−6.60510	+1.93731	+6.08588
H	−6.97858	+3.03440	+4.70186
H	−4.75890	+2.30342	+3.59693
H	−3.12064	+5.02363	+5.48348
C	−2.32507	+2.98409	+5.74826
O	−0.95631	+3.38163	+5.52213

H	−0.36165	+2.61811	+5.75123
H	−2.54038	+3.01007	+6.85522
C	−2.57051	+1.57041	+5.22487
O	−1.76966	+0.66247	+6.02022
H	−2.10186	−0.26916	+5.85227
H	−2.25972	+1.50301	+4.14553
C	−4.05653	+1.20764	+5.35012
H	−4.32939	+1.07352	+6.43048
O	−4.25281	−0.07279	+4.63343
C	−4.74020	−1.17834	+5.44943
O	−6.05847	−1.56880	+4.95326
H	−4.88374	−0.85167	+6.51425
C	−3.80140	−2.37474	+5.34005
O	−2.50478	−1.97534	+5.83366
H	−1.83555	−2.65275	+5.54649
H	−4.20224	−3.20930	+5.98409
C	−3.70276	−2.85954	+3.89494
O	−2.90015	−4.06462	+3.88895
H	−3.03939	−4.52429	+3.00783
H	−3.20802	−2.07066	+3.26164
C	−5.10097	−3.15552	+3.33585
H	−5.52787	−4.06757	+3.83126
C	−6.04200	−1.95710	+3.51940
H	−5.66616	−1.08768	+2.92002
C	−7.50972	−2.24710	+3.19149
H	−7.82729	−3.20176	+3.69211
H	−8.14016	−1.42640	+3.59612
O	−7.73279	−2.31865	+1.78391
H	−7.29495	−3.13451	+1.43665
O	+5.38762	−5.95042	−1.80048
H	+4.58604	−6.42909	−1.47478
C	+5.25417	−5.75090	−3.20755
H	+5.03192	−6.72271	−3.72562
H	+6.22619	−5.37551	−3.59356
C	+4.15970	−4.73083	−3.53424
H	+4.29415	−3.79736	−2.93040
O	+4.38425	−4.40012	−4.96508
C	+3.47510	−3.36569	−5.45707
O	+3.65659	−2.17204	−4.64052
H	+3.76826	−3.16497	−6.52243

C	+2.04323	−3.87756	−5.34598
O	+1.15665	−2.84764	−5.83139
H	+0.23149	−3.06210	−5.53529
H	+1.93736	−4.79375	−5.99578
C	+1.70262	−4.24890	−3.90366
O	+0.38016	−4.83746	−3.90701
H	+0.25379	−5.32284	−3.03829
H	+1.70791	−3.32741	−3.25753
C	+2.72750	−5.25565	−3.36451
H	+2.60952	−6.24115	−3.88836
O	+2.45104	−5.43398	−1.92103
C	+2.11522	−6.79381	−1.51470
O	+3.14793	−7.26680	−0.59449
C	+3.28563	−6.40918	+0.61082
C	+4.45352	−7.03105	+1.38213
O	+4.93141	−6.16295	+2.40922
H	+4.24625	−6.11087	+3.12022
H	+4.13742	−8.01889	+1.81411
H	+5.29179	−7.22176	+0.67805
H	+3.53764	−5.37004	+0.27853
H	+2.13550	−7.48664	−2.39832
C	+0.76935	−6.82340	−0.79866
O	−0.24113	−6.35356	−1.71525
H	−1.06673	−6.15050	−1.19850
H	+0.53970	−7.88921	−0.50914
C	+0.79871	−5.96063	+0.46159
O	−0.46790	−6.13663	+1.13995
H	−0.36590	−5.80323	+2.08082
H	+0.93412	−4.87831	+0.18310
C	+1.95294	−6.39994	+1.37202
H	+1.74029	−7.41582	+1.79895
O	+2.04060	−5.41532	+2.47329
C	+1.85137	−5.95885	+3.81334
O	+3.08930	−5.76503	+4.56569
C	+3.49606	−4.33837	+4.64730
C	+4.83099	−4.37079	+5.39729
O	+5.52717	−3.12933	+5.29189
H	+5.03555	−2.44795	+5.81348
H	+4.65281	−4.63452	+6.47484
H	+5.47472	−5.16221	+4.95673

H	+3.63521	−3.95063	+3.60620
H	+1.67454	−7.06698	+3.76981
C	+0.72253	−5.22746	+4.53109
O	−0.49505	−5.43096	+3.78343
H	−1.17603	−4.78125	+4.10608
H	+0.60291	−5.67073	+5.56124
C	+1.03413	−3.73782	+4.66095
O	−0.01989	−3.13771	+5.45088
H	+0.29758	−2.23699	+5.75919
H	+1.06335	−3.26192	+3.64103
C	+2.38997	−3.54107	+5.35193
H	+2.31852	−3.84141	+6.43089
O	+2.71657	−2.10061	+5.26529
C	+2.87965	−1.41870	+6.54427
O	+4.25940	−0.94602	+6.63710
C	+4.63009	−0.04346	+5.51670
C	+6.11258	+0.26137	+5.74922
O	+6.71576	+0.84896	+4.59614
H	+6.35624	+1.76463	+4.48758
H	+6.22611	+0.93440	+6.64139
H	+6.64784	−0.68723	+5.96964
H	+4.48680	−0.59824	+4.55429
H	+2.72841	−2.13406	+7.39661
C	+1.94269	−0.21915	+6.63039
O	+0.58472	−0.70096	+6.54114
H	−0.01556	+0.07442	+6.37203
H	+2.09174	+0.28470	+7.62822
C	+2.23490	+0.78294	+5.51513
O	+1.39615	+1.94099	+5.73712
H	+1.75305	+2.69430	+5.17683
H	+1.99366	+0.32469	+4.51419
C	+3.71490	+1.18821	+5.54256
H	+3.92577	+1.81274	+6.45088
O	+3.96865	+1.98978	+4.32617
C	+4.43192	+3.35366	+4.56182
O	+5.77846	+3.47862	+4.00962
C	+5.82541	+3.17336	+2.55509
C	+7.31191	+3.24726	+2.19831
O	+7.57759	+2.62860	+0.93666
H	+7.18374	+3.19936	+0.22950

H	+7.65216	+4.31709	+2.19038
H	+7.90094	+2.71023	+2.97297
H	+5.43187	+2.13644	+2.39872
H	+4.51795	+3.55547	+5.66348
C	+3.51489	+4.35522	+3.86868
O	+2.18981	+4.20251	+4.42175
H	+1.54187	+4.66356	+3.82352
H	+3.88890	+5.39654	+4.08897
C	+3.50202	+4.13973	+2.35612
O	+2.72030	+5.20834	+1.76922
H	+2.92772	+5.23734	+0.78729
H	+3.03410	+3.14634	+2.11093
C	+4.93586	+4.17711	+1.81007
H	+5.35649	+5.21468	+1.89921
O	+4.87002	+3.79269	+0.38792
C	+5.37337	+4.77681	−0.55667
O	+6.53973	+4.21273	−1.23434
C	+6.23469	+2.95060	−1.96594
C	+7.58906	+2.53016	−2.54437
O	+7.57525	+1.17674	−3.00163
H	+7.00585	+1.12877	−3.80851
H	+7.87977	+3.22398	−3.37948
H	+8.36445	+2.61417	−1.75203
H	+5.85531	+2.19011	−1.22724
H	+5.73229	+5.69659	−0.01931
C	+4.30493	+5.10476	−1.59437
O	+3.16244	+5.65230	−0.90067
H	+2.38293	+5.63936	−1.51802
H	+4.71362	+5.88141	−2.30345
C	+3.91141	+3.86072	−2.38887
O	+2.98575	+4.27779	−3.42379
H	+2.92466	+3.53801	−4.09776
H	+3.41395	+3.11144	−1.71391
C	+5.15796	+3.22961	−3.02223
H	+5.55904	+3.89602	−3.83222
O	+4.73948	+1.93863	−3.61862
C	+4.94311	+1.81435	−5.05550
O	+5.91481	+0.74663	−5.29420
H	+5.39262	+2.75377	−5.47558
C	+3.63477	+1.44672	−5.74684

O	+2.68759	+2.50882	−5.50564
H	+1.77699	+2.17896	−5.73131
H	+3.82246	+1.36145	−6.85586
C	+3.09659	+0.11504	−5.22621
O	+1.93267	−0.22545	−6.01728
H	+1.71513	−1.18968	−5.84579
H	+2.80383	+0.21821	−4.14380
C	+4.16248	−0.98102	−5.36082
H	+4.31505	−1.23896	−6.44234
C	+5.49003	−0.55508	−4.71971
H	+5.34930	−0.42544	−3.61604
C	+6.66269	−1.49680	−5.00823
H	+6.70215	−1.72700	−6.10729
H	+7.61223	−0.98894	−4.73414
O	+6.57291	−2.70052	−4.24561
H	+5.80971	−3.23052	−4.58426

345

* E = 0.939 kcal/mol

C	−0.07202	−0.11736	−0.98062
C	−0.60595	+0.83922	−0.08583
C	+0.18258	+1.49113	+0.85047
C	+1.54182	+1.37931	+1.17146
C	+2.52679	+0.40470	+0.99553
C	+2.30917	−0.97010	+0.81731
C	+1.04233	−1.52972	+0.94722
H	+0.33504	−1.04948	−0.16944
C	+3.43983	−1.89710	+0.46127
C	+4.73939	−1.19288	+0.13841
C	+5.01031	−0.15750	+1.20430
C	+3.94656	+0.91911	+1.11758
C	−0.90493	−0.74500	−2.08512
C	−2.36930	−0.30893	−2.12535
C	−2.88940	−0.02710	−0.73160
C	−2.07376	+1.13441	−0.16673
C	−4.29693	+0.57164	−0.66249
C	−4.31630	+1.27281	+0.70659
C	−2.84746	+1.55206	+1.07612
O	+4.98193	−0.83432	+2.44271
H	+5.44127	−0.32960	+3.11716
C	−2.79597	−1.27335	+0.14203

C	−5.44649	−0.38724	−0.87509
H	+5.98867	+0.29590	+1.04803
H	+4.17908	+1.55056	+0.25754
H	+4.00943	+1.57194	+1.99099
H	+4.67800	−0.69144	−0.82798
H	+5.55323	−1.91126	+0.09178
H	+3.60759	−2.56903	+1.30329
H	+3.12896	−2.52474	−0.37604
H	+0.32700	−1.02690	+1.59105
H	+0.96676	−2.61085	+0.92723
H	+1.92057	+2.27391	+1.65966
H	−0.31635	+2.29900	+1.37360
H	+0.96534	+0.07884	−1.25376
H	−0.85979	−1.83053	−1.97584
H	−0.43779	−0.53617	−3.04746
H	−2.46794	+0.60761	−2.71135
H	−2.96521	−1.07189	−2.62948
H	−4.34085	+1.33850	−1.44121
H	−4.78369	+0.62681	+1.44999
H	−4.91068	+2.18277	+0.67332
H	−2.53181	+0.95334	+1.93107
H	−2.67877	+2.59435	+1.34095
H	−3.25275	−1.11610	+1.11787
H	−1.76478	−1.57677	+0.30970
H	−3.31403	−2.10624	−0.33236
H	−6.39563	+0.14271	−0.86760
H	−5.48579	−1.13853	−0.08738
H	−5.36602	−0.90515	−1.83024
H	−2.19188	+1.94413	−0.89875
O	−7.00970	−4.02270	−0.99138
H	−6.38766	−4.47581	−1.61206
C	−6.90945	−4.65040	+0.28627
H	−7.05087	−5.76120	+0.19363
H	−7.72822	−4.25680	+0.92616
C	−5.56531	−4.35755	+0.95940
H	−5.35010	−3.25785	+0.95528
O	−5.76164	−4.79480	+2.36540
C	−4.59984	−4.52757	+3.21046
O	−4.32660	−3.09572	+3.17297
H	−4.88099	−4.84951	+4.24902

C	−3.40894	−5.31342	+2.67323
O	−2.26977	−5.03310	+3.51390
H	−1.44720	−5.34123	+3.04660
H	−3.64861	−6.41427	+2.72913
C	−3.11433	−4.94050	+1.22135
O	−2.06163	−5.81721	+0.75311
H	−2.03397	−5.76077	−0.24803
H	−2.77236	−3.86988	+1.16168
C	−4.37460	−5.12108	+0.36399
H	−4.62048	−6.21204	+0.26926
O	−4.07846	−4.56919	−0.97775
C	−4.16652	−5.52595	−2.07536
O	−5.24510	−5.10726	−2.96786
C	−5.04310	−3.74676	−3.52955
C	−6.30410	−3.49951	−4.36328
O	−6.43473	−2.12800	−4.73399
H	−5.73641	−1.91472	−5.40062
H	−6.28809	−4.15626	−5.27480
H	−7.19558	−3.77756	−3.76096
H	−4.97780	−3.01728	−2.68136
H	−4.44996	−6.54236	−1.69086
C	−2.86109	−5.56012	−2.86201
O	−1.80817	−5.97201	−1.96592
H	−0.93088	−5.77490	−2.39146
H	−2.95998	−6.31906	−3.69082
C	−2.55219	−4.19449	−3.47281
O	−1.36729	−4.34056	−4.29125
H	−1.30774	−3.54456	−4.89941
H	−2.36071	−3.44142	−2.65702
C	−3.73432	−3.72501	−4.33106
H	−3.82514	−4.36925	−5.24551
O	−3.45011	−2.33168	−4.74102
C	−3.36691	−2.10749	−6.18069
O	−4.44650	−1.20236	−6.56738
C	−4.40256	+0.08736	−5.83124
C	−5.64063	+0.84444	−6.32124
O	−5.94809	+1.95632	−5.48153
H	−5.23545	+2.63474	−5.58103
H	−5.48232	+1.18048	−7.38218
H	−6.51388	+0.15735	−6.31080

H	−4.47786	−0.12940	−4.73527
H	−3.53672	−3.06613	−6.74041
C	−2.03374	−1.46227	−6.54298
O	−0.97758	−2.36891	−6.15694
H	−0.11848	−1.86855	−6.14596
H	−1.99880	−1.30632	−7.65914
C	−1.86995	−0.11613	−5.84065
O	−0.65503	+0.49478	−6.33915
H	−0.65752	+1.45771	−6.05598
H	−1.78392	−0.27655	−4.73086
C	−3.07268	+0.79401	−6.12609
H	−3.04397	+1.14807	−7.19060
O	−2.95747	+1.95769	−5.21844
C	−2.83849	+3.25407	−5.87478
O	−4.00607	+4.05728	−5.51747
C	−4.14805	+4.24642	−4.05020
C	−5.45811	+5.02436	−3.89568
O	−5.91789	+5.02151	−2.54473
H	−5.30529	+5.57871	−2.00419
H	−5.31861	+6.07658	−4.26449
H	−6.23960	+4.54575	−4.52417
H	−4.22640	+3.23800	−3.56951
H	−2.86221	+3.13588	−6.99139
C	−1.58083	+3.97431	−5.40270
O	−0.43887	+3.16566	−5.75731
H	+0.35176	+3.49958	−5.25360
H	−1.51152	+4.96614	−5.93560
C	−1.62274	+4.22109	−3.89531
O	−0.45972	+5.01098	−3.55260
H	−0.61320	+5.41420	−2.64661
H	−1.59422	+3.24012	−3.34305
C	−2.90523	+4.97762	−3.52444
H	−2.86622	+6.02133	−3.93538
O	−2.97008	+5.03711	−2.04687
C	−3.00077	+6.38150	−1.48085
O	−4.27028	+6.55660	−0.77922
C	−4.47208	+5.57154	+0.31459
C	−5.87294	+5.88860	+0.84639
O	−6.35884	+4.85854	+1.70602
H	−5.84106	+4.88295	+2.54803

H	−5.85849	+6.87642	+1.38131
H	−6.57378	+5.97787	−0.01137
H	−4.43683	+4.54123	−0.12343
H	−2.98112	+7.15277	−2.29713
C	−1.85767	+6.57560	−0.49055
O	−0.61399	+6.38649	−1.19703
H	+0.11696	+6.27686	−0.53072
H	−1.90164	+7.63136	−0.09517
C	−1.96876	+5.60245	+0.68177
O	−0.92625	+5.94042	+1.62735
H	−1.14243	+5.49700	+2.50124
H	−1.82802	+4.54490	+0.32144
C	−3.34622	+5.73506	+1.34470
H	−3.43073	+6.72830	+1.86029
O	−3.45473	+4.65290	+2.34800
C	−3.64310	+5.09423	+3.72618
O	−4.94833	+4.62446	+4.18409
C	−5.09267	+3.14828	+4.09331
C	−6.52973	+2.88459	+4.55295
O	−6.95457	+1.56039	+4.23352
H	−6.43840	+0.92172	+4.78440
H	−6.61226	+3.07130	+5.65833
H	−7.21156	+3.59525	+4.03822
H	−4.95501	+2.84453	+3.02426
H	−3.66974	+6.21565	+3.78126
C	−2.56539	+4.50394	+4.62866
O	−1.28265	+4.98008	+4.16752
H	−0.57098	+4.42012	+4.57942
H	−2.73862	+4.86751	+5.68202
C	−2.61076	+2.97758	+4.61090
O	−1.65849	+2.50004	+5.59153
H	−1.83056	+1.52180	+5.73486
H	−2.32552	+2.59908	+3.59041
C	−4.01941	+2.47826	+4.96165
H	−4.22974	+2.65415	+6.04998
O	−4.04345	+1.02443	+4.68630
C	−4.30445	+0.17229	+5.83916
O	−5.56649	−0.52989	+5.61097
H	−4.44585	+0.79073	+6.76589
C	−3.19142	−0.85674	+6.00182

O	−1.95430	−0.14906	+6.22815
H	−1.19787	−0.78520	+6.10846
H	−3.42126	−1.49802	+6.90099
C	−3.08805	−1.74956	+4.76608
O	−2.10098	−2.77033	+5.04658
H	−2.22694	−3.51535	+4.38663
H	−2.76393	−1.13965	+3.87673
C	−4.44803	−2.39592	+4.47117
H	−4.69946	−3.14147	+5.27159
C	−5.55427	−1.33752	+4.36368
H	−5.34220	−0.65426	+3.50148
C	−6.97308	−1.90624	+4.26831
H	−7.11716	−2.69796	+5.05268
H	−7.70312	−1.09306	+4.46969
O	−7.24654	−2.42971	+2.96903
H	−6.70950	−3.25040	+2.84197
O	+6.10983	−5.57664	−0.19391
H	+5.38919	−6.02218	+0.31552
C	+5.90424	−5.82134	−1.58403
H	+5.80064	−6.92279	−1.78179
H	+6.80268	−5.46253	−2.13100
C	+4.67025	−5.08632	−2.11639
H	+4.69173	−4.00600	−1.82167
O	+4.80728	−5.17403	−3.59357
C	+3.74157	−4.47818	−4.31101
O	+3.75765	−3.07506	−3.91252
H	+3.97523	−4.58082	−5.40474
C	+2.40584	−5.11802	−3.95082
O	+1.36546	−4.42192	−4.66900
H	+0.48671	−4.66339	−4.27031
H	+2.42250	−6.19738	−4.27796
C	+2.16001	−5.05771	−2.44447
O	+0.93234	−5.77502	−2.17546
H	+0.89581	−5.97549	−1.19237
H	+2.05647	−3.98610	−2.11319
C	+3.33138	−5.70834	−1.69665
H	+3.33866	−6.81524	−1.88200
O	+3.12525	−5.45460	−0.25395
C	+2.98076	−6.64561	+0.57725
O	+4.09324	−6.68372	+1.52268

C	+4.17310	−5.46810	+2.37523
C	+5.41236	−5.70217	+3.24506
O	+5.88398	−4.49335	+3.83676
H	+5.19342	−4.15343	+4.45866
H	+5.17907	−6.46779	+4.03597
H	+6.22821	−6.11163	+2.61100
H	+4.31679	−4.57842	+1.71037
H	+3.05944	−7.57569	−0.04734
C	+1.67429	−6.58898	+1.36063
O	+0.58159	−6.57338	+0.41661
H	−0.24414	−6.29093	+0.89414
H	+1.59579	−7.51273	+2.00256
C	+1.63689	−5.35002	+2.25145
O	+0.43012	−5.41228	+3.04914
H	+0.53013	−4.76232	+3.80783
H	+1.62214	−4.42253	+1.61253
C	+2.86701	−5.31309	+3.16717
H	+2.78605	−6.11296	+3.95077
O	+2.86669	−3.98600	+3.81810
C	+2.85218	−3.99941	+5.27447
O	+4.08532	−3.37271	+5.74529
C	+4.23301	−1.98167	+5.23962
C	+5.63382	−1.56152	+5.68718
O	+6.00965	−0.30092	+5.11657
H	+5.51110	+0.40753	+5.59506
H	+5.68039	−1.50779	+6.80633
H	+6.36916	−2.32250	+5.34998
H	+4.16346	−1.99881	+4.12267
H	+2.86050	−5.05437	+5.66075
C	+1.65827	−3.21004	+5.80317
O	+0.45544	−3.81927	+5.28432
H	−0.29804	−3.18197	+5.40742
H	+1.65130	−3.28086	+6.92916
C	+1.73435	−1.73702	+5.40066
O	+0.65241	−1.04935	+6.07480
H	+0.83837	−0.06475	+6.03431
H	+1.61521	−1.63396	+4.28698
C	+3.08636	−1.14639	+5.82442
H	+3.15976	−1.11171	+6.94423
O	+3.15836	+0.23172	+5.28957

C	+3.26667	+1.28339	+6.28981
O	+4.56696	+1.94002	+6.13042
C	+4.75729	+2.52043	+4.77697
C	+6.17652	+3.09548	+4.80818
O	+6.66166	+3.37055	+3.49390
H	+6.16457	+4.14565	+3.13201
H	+6.19686	+4.02397	+5.43981
H	+6.85910	+2.35324	+5.27601
H	+4.68272	+1.69335	+4.02383
H	+3.25684	+0.84993	+7.32589
C	+2.16928	+2.32428	+6.09664
O	+0.89459	+1.67217	+6.27279
H	+0.18135	+2.27284	+5.92604
H	+2.28870	+3.12372	+6.88375
C	+2.26158	+2.96627	+4.71389
O	+1.26758	+4.01779	+4.65052
H	+1.47505	+4.58825	+3.85165
H	+2.05484	+2.19861	+3.91792
C	+3.66142	+3.55691	+4.50008
H	+3.80196	+4.46152	+5.14920
O	+3.75547	+3.95458	+3.07743
C	+4.00097	+5.37175	+2.83898
O	+5.29570	+5.50609	+2.17331
C	+5.36369	+4.75170	+0.89528
C	+6.80148	+4.95856	+0.40993
O	+7.14675	+4.03765	−0.62451
H	+6.64102	+4.27754	−1.43997
H	+6.93388	+6.01726	+0.05795
H	+7.49832	+4.79472	+1.26001
H	+5.17074	+3.66975	+1.11294
H	+4.08246	+5.92873	+3.81071
C	+2.91896	+5.95160	+1.93548
O	+1.65079	+5.81150	+2.61054
H	+0.92170	+5.95488	+1.94891
H	+3.13281	+7.04700	+1.77191
C	+2.89547	+5.23921	+0.58410
O	+1.93801	+5.92853	−0.25467
H	+2.09603	+5.64375	−1.20392
H	+2.58098	+4.16683	+0.72075
C	+4.28773	+5.28453	−0.06019

H	+4.53096	+6.33380	−0.37577
O	+4.24609	+4.41173	−1.25444
C	+4.53517	+5.07273	−2.52128
O	+5.74839	+4.47914	−3.08089
C	+5.62759	+3.01328	−3.29123
C	+7.01040	+2.59010	−3.79541
O	+7.17912	+1.17369	−3.74900
H	+6.59802	+0.76590	−4.43743
H	+7.16543	+2.97332	−4.84015
H	+7.78817	+3.04863	−3.14752
H	+5.40010	+2.53249	−2.30581
H	+4.75913	+6.16106	−2.35802
C	+3.38820	+4.86661	−3.50429
O	+2.19856	+5.45672	−2.93923
H	+1.40834	+5.10929	−3.43385
H	+3.64103	+5.39419	−4.46887
C	+3.17241	+3.38163	−3.79025
O	+2.15838	+3.27891	−4.81821
H	+2.19758	+2.35276	−5.20131
H	+2.82402	+2.85696	−2.85769
C	+4.48124	+2.74445	−4.27547
H	+4.74334	+3.13511	−5.29447
O	+4.25419	+1.28425	−4.35557
C	+4.43106	+0.69784	−5.67893
O	+5.54120	−0.25069	−5.61588
H	+4.72648	+1.48465	−6.42389
C	+3.17206	−0.04807	−6.10643
O	+2.08450	+0.89868	−6.16660
H	+1.22962	+0.39508	−6.24069
H	+3.34195	−0.48177	−7.13369
C	+2.84924	−1.18156	−5.13425
O	+1.71591	−1.90394	−5.67192
H	+1.65834	−2.78774	−5.20006
H	+2.58662	−0.75870	−4.12451
C	+4.06042	−2.11413	−4.99638
H	+4.22340	−2.67522	−5.95465
C	+5.32334	−1.32880	−4.61696
H	+5.18601	−0.85663	−3.61084
C	+6.61695	−2.14760	−4.66060
H	+6.67261	−2.71424	−5.62927

H	+7.48519	−1.45503	−4.62295
O	+6.71706	−3.03843	−3.55031
H	+6.04185	−3.75252	−3.65911

345

* E = 1.247 kcal/mol

C	−0.41117	+0.38200	+1.13290
C	+0.21507	+0.77042	−0.07026
C	−0.48137	+1.33177	−1.13288
C	−1.84159	+1.59590	−1.32525
C	−3.01218	+1.08582	−0.76991
C	−3.18930	−0.15677	−0.15093
C	−2.19171	−1.12686	−0.09833
H	−1.22722	−0.58837	+0.72503
C	−4.53271	−0.38468	+0.46960
C	−5.66201	−0.17933	−0.54299
C	−5.39631	+0.99687	−1.48592
C	−4.28117	+1.87427	−0.95125
C	+0.37596	−0.11360	+2.33341
C	+1.88548	−0.23603	+2.11988
C	+2.20813	−0.58709	+0.68316
C	+1.69483	+0.55660	−0.18866
C	+3.69492	−0.56130	+0.31637
C	+3.67923	−0.35827	−1.20813
C	+2.32589	+0.29234	−1.54848
O	−4.96370	+0.55510	−2.75713
H	−5.71019	+0.20524	−3.25268
C	+1.58761	−1.92541	+0.30117
C	+4.52067	−1.74486	+0.76832
H	−6.29701	+1.59930	−1.59848
H	−4.59905	+2.29614	+0.00519
H	−4.11817	+2.70968	−1.62839
H	−6.59584	−0.03189	−0.00518
H	−5.77693	−1.07728	−1.14852
H	−4.58940	−1.38250	+0.90080
H	−4.67410	+0.32150	+1.29218
H	−1.46577	−1.13121	−0.90784
H	−2.46083	−2.10522	+0.28167
H	−2.02239	+2.35889	−2.07597
H	+0.14764	+1.71899	−1.92607
H	−1.29096	+0.97585	+1.38091

H	−0.02278	−1.08626	+2.62857
H	+0.18438	+0.54191	+3.18307
H	+2.37011	+0.71651	+2.34495
H	+2.29628	−0.97144	+2.81387
H	+4.10748	+0.34037	+0.77768
H	+3.78261	−1.31878	−1.71360
H	+4.52009	+0.25055	−1.53294
H	+1.69455	−0.37988	−2.12974
H	+2.44024	+1.20242	−2.13421
H	+1.87429	−2.23065	−0.70421
H	+0.50130	−1.89538	+0.33676
H	+1.92017	−2.70295	+0.98792
H	+5.56869	−1.60196	+0.51729
H	+4.19179	−2.66446	+0.28535
H	+4.45696	−1.89066	+1.84625
H	+2.17806	+1.45666	+0.21339
O	−7.08258	−3.25466	−2.13779
H	−6.47215	−3.54834	−2.85896
C	−7.06795	−4.24641	−1.10923
H	−7.26614	−5.26433	−1.54005
H	−7.88841	−4.01324	−0.39710
C	−5.73547	−4.25134	−0.35582
H	−5.46119	−3.21365	−0.03511
O	−5.99737	−5.06314	+0.86030
C	−4.85094	−5.09369	+1.76612
O	−4.51924	−3.71946	+2.12698
H	−5.17421	−5.67293	+2.67233
C	−3.67730	−5.75780	+1.05342
O	−2.55072	−5.76420	+1.95752
H	−1.72781	−5.96735	+1.43715
H	−3.96043	−6.82127	+0.80737
C	−3.32602	−5.02102	−0.23803
O	−2.30776	−5.79216	−0.91889
H	−2.23881	−5.44876	−1.85988
H	−2.92641	−3.99703	+0.00627
C	−4.56739	−4.87654	−1.12974
H	−4.86288	−5.87571	−1.54685
O	−4.20383	−3.96874	−2.23880
C	−4.29201	−4.54279	−3.57497
O	−5.31902	−3.81381	−4.31918

C	−5.03237	−2.35821	−4.41808
C	−6.24568	−1.78818	−5.15767
O	−6.32162	−0.36384	−5.03244
H	−5.58396	+0.03750	−5.56190
H	−6.20839	−2.08242	−6.23973
H	−7.17377	−2.21700	−4.72158
H	−4.96527	−1.93881	−3.38187
H	−4.63363	−5.61172	−3.52529
C	−2.95995	−4.40063	−4.30245
O	−1.96089	−5.12546	−3.55333
H	−1.06175	−4.83946	−3.86953
H	−3.05808	−4.85976	−5.32826
C	−2.56924	−2.93092	−4.44777
O	−1.36366	−2.87544	−5.24761
H	−1.25564	−1.93322	−5.57687
H	−2.37614	−2.48117	−3.43513
C	−3.69596	−2.15641	−5.14393
H	−3.78068	−2.48156	−6.21557
O	−3.34138	−0.72474	−5.09066
C	−3.22035	−0.05993	−6.37978
O	−4.24696	+0.97701	−6.46261
C	−4.12578	+2.02343	−5.40572
C	−5.29686	+2.96553	−5.70248
O	−5.55489	+3.85742	−4.61799
H	−4.79592	+4.48689	−4.54592
H	−5.09147	+3.54168	−6.64603
H	−6.21470	+2.36094	−5.87197
H	−4.24511	+1.53761	−4.39929
H	−3.43151	−0.78014	−7.21683
C	−1.84532	+0.58326	−6.51963
O	−0.85020	−0.45894	−6.42497
H	+0.03825	−0.03382	−6.28647
H	−1.77530	+1.06851	−7.53588
C	−1.62330	+1.64473	−5.44438
O	−0.34334	+2.27030	−5.71029
H	−0.30781	+3.13267	−5.19918
H	−1.61032	+1.16899	−4.42561
C	−2.74626	+2.68736	−5.50904
H	−2.66443	+3.28104	−6.45905
O	−2.57006	+3.59489	−4.35218

C	−2.35053	+4.99605	−4.68326
O	−3.47243	+5.77493	−4.15856
C	−3.64999	+5.60230	−2.69465
C	−4.90124	+6.42117	−2.36477
O	−5.42183	+6.09456	−1.07587
H	−4.78442	+6.41241	−0.38960
H	−4.66492	+7.51799	−2.42947
H	−5.68883	+6.19980	−3.11675
H	−3.82268	+4.51484	−2.48615
H	−2.35022	+5.14687	−5.79620
C	−1.06484	+5.50066	−4.03722
O	+0.03586	+4.73541	−4.57252
H	+0.83140	+4.87605	−3.99275
H	−0.92659	+6.58721	−4.30675
C	−1.12863	+5.36516	−2.51708
O	+0.06325	+5.98142	−1.97139
H	−0.08060	+6.10931	−0.98663
H	−1.15853	+4.27734	−2.23087
C	−2.37924	+6.06779	−1.97101
H	−2.26418	+7.18100	−2.05711
O	−2.49561	+5.69657	−0.54288
C	−2.45324	+6.80628	+0.39910
O	−3.73311	+6.85407	+1.10590
C	−4.04192	+5.58703	+1.81757
C	−5.43986	+5.81333	+2.39994
O	−6.02502	+4.59410	+2.85759
H	−5.53514	+4.29605	+3.66317
H	−5.38529	+6.56324	+3.23467
H	−6.09831	+6.23325	+1.60947
H	−4.05406	+4.75413	+1.06900
H	−2.35183	+7.78447	−0.14336
C	−1.33802	+6.59855	+1.41782
O	−0.08183	+6.55102	+0.70847
H	+0.60615	+6.16257	+1.31279
H	−1.32955	+7.47573	+2.12730
C	−1.55735	+5.31448	+2.21617
O	−0.53430	+5.25849	+3.23919
H	−0.81723	+4.58244	+3.92355
H	−1.46860	+4.42021	+1.53934
C	−2.94865	+5.32924	+2.86333

H	−2.98894	+6.11051	+3.66815
O	−3.16688	+3.99521	+3.46764
C	−3.38999	+3.99419	+4.90834
O	−4.73744	+3.48773	+5.16470
C	−4.95132	+2.11682	+4.63424
C	−6.42381	+1.82431	+4.93715
O	−6.89016	+0.67250	+4.23484
H	−6.46346	−0.12799	+4.62814
H	−6.56319	+1.69559	+6.04439
H	−7.03872	+2.69267	+4.61677
H	−4.76847	+2.13166	+3.52943
H	−3.36233	+5.04130	+5.31343
C	−2.38417	+3.08703	+5.60850
O	−1.05917	+3.59475	+5.34776
H	−0.39593	+2.90500	+5.62037
H	−2.58172	+3.12176	+6.71862
C	−2.51464	+1.64324	+5.12596
O	−1.61009	+0.83817	+5.91901
H	−1.86957	−0.12456	+5.80629
H	−2.23164	+1.57341	+4.03829
C	−3.95955	+1.15946	+5.30921
H	−4.19550	+1.05989	+6.40188
O	−4.06711	−0.16780	+4.66318
C	−4.43793	−1.26506	+5.54990
O	−5.72757	−1.79529	+5.11192
H	−4.58601	−0.89320	+6.59910
C	−3.40039	−2.37979	+5.47969
O	−2.13605	−1.84451	+5.92346
H	−1.41243	−2.47495	+5.66099
H	−3.71522	−3.21085	+6.17424
C	−3.28893	−2.93088	+4.05943
O	−2.37816	−4.05511	+4.09551
H	−2.49930	−4.58098	+3.24940
H	−2.88671	−2.13700	+3.36991
C	−4.66685	−3.38630	+3.56142
H	−4.99509	−4.30093	+4.12288
C	−5.70916	−2.26852	+3.70368
H	−5.42890	−1.40685	+3.04543
C	−7.15184	−2.70771	+3.43631
H	−7.36823	−3.65796	+3.99547

H	−7.84448	−1.92604	+3.81599
O	−7.40287	−2.87668	+2.04141
H	−6.90261	−3.66970	+1.72612
O	+5.92572	−5.52817	−1.63168
H	+5.17469	−6.05377	−1.26111
C	+5.74072	−5.40571	−3.04131
H	+5.58932	−6.41575	−3.50999
H	+6.66730	−4.96821	−3.47114
C	+4.55573	−4.49756	−3.38264
H	+4.62287	−3.52872	−2.82459
O	+4.71922	−4.21698	−4.83265
C	+3.71448	−3.28786	−5.34692
O	+3.80857	−2.04763	−4.58717
H	+3.96683	−3.11114	−6.42689
C	+2.33432	−3.91301	−5.17752
O	+1.35434	−2.98523	−5.68795
H	+0.45516	−3.26330	−5.36419
H	+2.29313	−4.86511	−5.78142
C	+2.05907	−4.24200	−3.71116
O	+0.79191	−4.93875	−3.65033
H	+0.73141	−5.39782	−2.76059
H	+2.00175	−3.29329	−3.10691
C	+3.17730	−5.13286	−3.15444
H	+3.13264	−6.14774	−3.63164
O	+2.94364	−5.26712	−1.69912
C	+2.74562	−6.63113	−1.22276
O	+3.83403	−6.95829	−0.30414
C	+3.91531	−6.02318	+0.84739
C	+5.15155	−6.48980	+1.62187
O	+5.57107	−5.52231	+2.58314
H	+4.89570	−5.48293	+3.30419
H	+4.93593	−7.47436	+2.11899
H	+5.98816	−6.64710	+0.90769
H	+4.06223	−4.98525	+0.45184
H	+2.81570	−7.36128	−2.07321
C	+1.42176	−6.75160	−0.47576
O	+0.35547	−6.42569	−1.39255
H	−0.47954	−6.28527	−0.87065
H	+1.29934	−7.81813	−0.12983
C	+1.39083	−5.82554	+0.73867

O	+0.16275	−6.09183	+1.45859
H	+0.24521	−5.68280	+2.37091
H	+1.40798	−4.75195	+0.40140
C	+2.60343	−6.09307	+1.64092
H	+2.49990	−7.09483	+2.13626
O	+2.62129	−5.03507	+2.67641
C	+2.51343	−5.50811	+4.05147
O	+3.74616	−5.16334	+4.75624
C	+4.02482	−3.70382	+4.76234
C	+5.37508	−3.58729	+5.47614
O	+5.95186	−2.29275	+5.31285
H	+5.41798	−1.64306	+5.83285
H	+5.24709	−3.82930	+6.56593
H	+6.07752	−4.33198	+5.04393
H	+4.10352	−3.35224	+3.70138
H	+2.43462	−6.62810	+4.07895
C	+1.33893	−4.83783	+4.75519
O	+0.13317	−5.18455	+4.04236
H	−0.59826	−4.57954	+4.34021
H	+1.27403	−5.23665	+5.80842
C	+1.52303	−3.32209	+4.80795
O	+0.43382	−2.77719	+5.59041
H	+0.68180	−1.84365	+5.86292
H	+1.49510	−2.89330	+3.76655
C	+2.86743	−2.97957	+5.46357
H	+2.84232	−3.24867	+6.55287
O	+3.06159	−1.51874	+5.32575
C	+3.19097	−0.78544	+6.58116
O	+4.52683	−0.19684	+6.63511
C	+4.80674	+0.69970	+5.48387
C	+6.25754	+1.14277	+5.69597
O	+6.80481	+1.74378	+4.52341
H	+6.34602	+2.60555	+4.36610
H	+6.31199	+1.85113	+6.56706
H	+6.87585	+0.25342	+5.94402
H	+4.70713	+0.10448	+4.54092
H	+3.11453	−1.48490	+7.45647
C	+2.15875	+0.33438	+6.65409
O	+0.84459	−0.26245	+6.59905
H	+0.18299	+0.44879	+6.38706

H	+2.27974	+0.87365	+7.63697
C	+2.34631	+1.32730	+5.50909
O	+1.42555	+2.42431	+5.72442
H	+1.69501	+3.17553	+5.11555
H	+2.11330	+0.82500	+4.53010
C	+3.78968	+1.84886	+5.47836
H	+3.96946	+2.53556	+6.34759
O	+3.95014	+2.60379	+4.21531
C	+4.28778	+4.01415	+4.36769
O	+5.60778	+4.23576	+3.78086
C	+5.67202	+3.85626	+2.34528
C	+7.13658	+4.08579	+1.96066
O	+7.45703	+3.47568	+0.71115
H	+6.99243	+3.97036	−0.00813
H	+7.35062	+5.18855	+1.92967
H	+7.78995	+3.63563	+2.73867
H	+5.40272	+2.77335	+2.25372
H	+4.37230	+4.28526	+5.45432
C	+3.27165	+4.88560	+3.63872
O	+1.97405	+4.65268	+4.22647
H	+1.28121	+5.02528	+3.61672
H	+3.55476	+5.96782	+3.78414
C	+3.25255	+4.57268	+2.14330
O	+2.35880	+5.52006	+1.51275
H	+2.54335	+5.51318	+0.52645
H	+2.87823	+3.52407	+1.97382
C	+4.66575	+4.70977	+1.56131
H	+4.98028	+5.78714	+1.56990
O	+4.61381	+4.22743	+0.16308
C	+4.99568	+5.20821	−0.84730
O	+6.18993	+4.72043	−1.53310
C	+5.98713	+3.40108	−2.18571
C	+7.36285	+3.05794	−2.76494
O	+7.44046	+1.69377	−3.17581
H	+6.86684	+1.57241	−3.97198
H	+7.59101	+3.74405	−3.62507
H	+8.13599	+3.22477	−1.98437
H	+5.68895	+2.65924	−1.40150
H	+5.28225	+6.18071	−0.36391
C	+3.87961	+5.38969	−1.87000

O	+2.70642	+5.86355	−1.17619
H	+1.92104	+5.77532	−1.78121
H	+4.20405	+6.16625	−2.62113
C	+3.58400	+4.08245	−2.60310
O	+2.61159	+4.37422	−3.63430
H	+2.59361	+3.60074	−4.27365
H	+3.16178	+3.32452	−1.88503
C	+4.86981	+3.52695	−3.23034
H	+5.19991	+4.18587	−4.07673
O	+4.55463	+2.17906	−3.75409
C	+4.73100	+2.01154	−5.19273
O	+5.78661	+1.02552	−5.41414
H	+5.08539	+2.96877	−5.66123
C	+3.44402	+1.50488	−5.83418
O	+2.41208	+2.48888	−5.60801
H	+1.52753	+2.07027	−5.78747
H	+3.61389	+1.39452	−6.94374
C	+3.03631	+0.15253	−5.25306
O	+1.89119	−0.31681	−6.00450
H	+1.76050	−1.28881	−5.79289
H	+2.75669	+0.27126	−4.16995
C	+4.19215	−0.85071	−5.36961
H	+4.34333	−1.14152	−6.44296
C	+5.49104	−0.28502	−4.77967
H	+5.36507	−0.12396	−3.67841
C	+6.73572	−1.13027	−5.06615
H	+6.76845	−1.39616	−6.15778
H	+7.64240	−0.53013	−4.83686
O	+6.77408	−2.30750	−4.26042
H	+6.04508	−2.91044	−4.54814

345

* E = 1.331 kcal/mol

C	−0.91021	−0.28570	+1.03510
C	−0.27498	+0.78504	+0.37352
C	−0.95641	+1.75919	−0.33856
C	−2.31904	+1.92510	−0.61232
C	−3.44034	+1.08988	−0.61211
C	−3.44417	−0.30340	−0.75982
C	−2.28393	−1.00423	−1.09173
H	−1.48092	−0.97108	+0.02920

C	−4.70240	−1.10979	−0.58568
C	−5.91943	−0.30865	−0.17375
C	−5.95351	+0.97548	−0.96696
C	−4.76475	+1.81889	−0.55712
C	−0.04257	−1.13501	+1.92553
C	+1.23160	−1.63600	+1.23802
C	+1.79593	−0.62848	+0.23084
C	+1.21597	+0.77065	+0.49311
C	+3.29211	−0.32453	+0.36951
C	+3.45839	+1.00943	−0.37260
C	+2.07492	+1.68543	−0.36269
O	−5.90019	+0.60953	−2.32612
H	−5.99607	+1.38262	−2.88761
C	+1.46975	−1.08936	−1.18334
C	+4.24252	−1.40775	−0.08815
H	−6.87200	+1.52499	−0.75720
H	−4.94540	+2.18914	+0.45415
H	−4.70144	+2.70748	−1.18895
H	−5.88269	−0.05970	+0.88633
H	−6.82078	−0.88972	−0.34742
H	−4.91290	−1.60586	−1.53282
H	−4.51020	−1.90368	+0.13922
H	−1.53500	−0.46199	−1.66059
H	−2.39516	−2.04862	−1.36153
H	−2.56243	+2.95363	−0.86522
H	−0.34675	+2.59353	−0.66509
H	−1.90607	−0.06695	+1.41884
H	−0.61058	−1.97629	+2.31896
H	+0.23770	−0.53318	+2.79520
H	+1.97635	−1.86846	+2.00114
H	+1.01793	−2.57535	+0.72741
H	+3.46684	−0.14712	+1.43422
H	+3.79020	+0.82920	−1.39519
H	+4.22198	+1.62798	+0.09303
H	+1.66545	+1.74958	−1.37071
H	+2.10722	+2.70120	+0.02612
H	+1.84935	−0.40898	−1.94445
H	+0.39585	−1.18038	−1.31980
H	+1.90548	−2.07001	−1.36982
H	+5.27331	−1.12914	+0.11661

H	+4.15819	−1.58236	−1.16005
H	+4.04783	−2.35140	+0.42217
H	+1.45546	+0.98574	+1.54369
O	−6.69300	−4.62936	−1.45921
H	−6.02152	−4.91146	−2.12784
C	−6.49141	−5.37792	−0.26171
H	−6.49709	−6.48115	−0.47736
H	−7.34065	−5.16291	+0.42220
C	−5.18134	−4.99653	+0.43448
H	−5.08874	−3.88352	+0.52390
O	−5.31384	−5.56607	+1.80047
C	−4.16710	−5.26750	+2.65544
O	−4.01890	−3.81820	+2.73742
H	−4.40143	−5.69871	+3.66556
C	−2.92187	−5.89534	+2.04019
O	−1.79942	−5.61063	+2.90152
H	−0.96063	−5.80851	+2.40440
H	−3.07080	−7.01184	+1.98339
C	−2.67707	−5.35078	+0.63449
O	−1.54941	−6.06839	+0.07866
H	−1.54044	−5.91354	−0.91315
H	−2.44100	−4.25113	+0.68488
C	−3.92266	−5.56358	−0.23619
H	−4.05421	−6.65591	−0.45803
O	−3.70044	−4.82963	−1.50126
C	−3.70911	−5.64626	−2.71022
O	−4.81490	−5.20320	−3.55467
C	−4.73734	−3.76046	−3.90572
C	−5.99083	−3.51582	−4.75236
O	−6.30976	−2.12996	−4.84839
H	−5.57214	−1.66388	−5.31359
H	−5.84694	−3.96366	−5.77507
H	−6.85401	−4.03145	−4.27869
H	−4.77353	−3.16262	−2.95878
H	−3.90835	−6.72277	−2.45978
C	−2.40495	−5.46610	−3.47784
O	−1.32185	−5.93180	−2.64411
H	−0.46376	−5.60983	−3.03125
H	−2.44549	−6.09133	−4.41537
C	−2.20536	−4.00059	−3.85683

O	−1.01341	−3.91551	−4.67417
H	−1.02681	−3.03052	−5.14743
H	−2.07312	−3.38081	−2.92593
C	−3.41840	−3.48493	−4.64207
H	−3.43719	−3.95100	−5.66329
O	−3.25039	−2.02108	−4.77547
C	−3.23543	−1.51649	−6.14176
O	−4.37182	−0.61270	−6.30740
C	−4.33693	+0.53883	−5.36782
C	−5.65644	+1.26813	−5.62967
O	−5.88443	+2.31154	−4.67425
H	−5.24602	+3.04410	−4.86269
H	−5.65905	+1.68874	−6.66954
H	−6.49479	+0.54434	−5.55063
H	−4.30175	+0.14673	−4.32028
H	−3.38781	−2.35480	−6.87394
C	−1.94472	−0.75159	−6.41747
O	−0.83397	−1.64927	−6.20258
H	−0.00234	−1.10960	−6.12722
H	−1.94754	−0.42038	−7.49607
C	−1.82274	+0.48169	−5.52326
O	−0.64520	+1.20668	−5.95123
H	−0.69438	+2.13482	−5.57402
H	−1.70924	+0.17097	−4.44759
C	−3.07427	+1.35774	−5.67167
H	−3.12774	+1.77945	−6.71069
O	−2.95978	+2.46933	−4.70177
C	−2.90038	+3.80246	−5.28771
O	−4.09692	+4.53820	−4.87500
C	−4.22313	+4.64536	−3.39935
C	−5.54785	+5.38250	−3.18278
O	−6.00717	+5.25101	−1.83711
H	−5.41179	+5.77914	−1.24939
H	−5.43085	+6.46535	−3.45683
H	−6.32066	+4.94579	−3.85192
H	−4.27439	+3.60992	−2.97394
H	−2.93464	+3.74483	−6.40876
C	−1.67061	+4.55666	−4.79323
O	−0.49571	+3.83699	−5.22078
H	+0.28573	+4.17681	−4.70753

H	−1.66733	+5.58294	−5.26175
C	−1.69086	+4.70059	−3.27321
O	−0.56076	+5.52290	−2.89313
H	−0.69900	+5.81353	−1.94268
H	−1.60388	+3.68822	−2.79028
C	−2.99789	+5.36740	−2.82495
H	−3.00186	+6.44969	−3.12184
O	−3.05596	+5.26213	−1.35066
C	−3.10211	+6.52833	−0.63006
O	−4.37136	+6.59412	+0.09247
C	−4.55510	+5.44982	+1.02236
C	−5.95338	+5.65723	+1.61146
O	−6.44081	+4.47070	+2.23856
H	−5.91443	+4.31181	+3.06106
H	−5.93657	+6.51196	+2.34048
H	−6.65623	+5.92428	+0.79299
H	−4.51486	+4.50181	+0.42687
H	−3.09540	+7.39320	−1.34639
C	−1.95920	+6.60520	+0.37548
O	−0.71513	+6.55248	−0.35518
H	+0.02262	+6.38056	+0.29016
H	−2.02491	+7.58840	+0.92417
C	−2.04432	+5.46073	+1.38416
O	−1.00901	+5.67372	+2.37413
H	−1.21673	+5.09450	+3.16680
H	−1.87814	+4.47668	+0.86427
C	−3.42314	+5.45462	+2.05830
H	−3.52123	+6.34032	+2.74089
O	−3.51390	+4.21088	+2.85386
C	−3.73919	+4.39628	+4.28100
O	−5.02235	+3.78462	+4.62404
C	−5.07804	+2.33911	+4.28476
C	−6.51053	+1.92526	+4.63347
O	−6.83756	+0.64681	+4.08913
H	−6.32760	−0.04163	+4.58300
H	−6.64378	+1.92602	+5.74886
H	−7.21689	+2.66985	+4.20729
H	−4.88829	+2.22582	+3.18680
H	−3.82969	+5.48768	+4.53016
C	−2.63756	+3.71549	+5.08546

O	−1.37911	+4.32700	+4.73060
H	−0.64105	+3.73926	+5.04549
H	−2.83460	+3.88457	+6.18331
C	−2.60696	+2.21268	+4.81092
O	−1.62374	+1.63049	+5.69960
H	−1.78109	+0.64025	+5.73220
H	−2.31438	+2.02187	+3.74130
C	−3.98996	+1.60385	+5.07889
H	−4.21877	+1.64169	+6.17708
O	−3.94567	+0.19134	+4.63836
C	−4.20890	−0.79262	+5.68234
O	−5.43215	−1.51503	+5.33872
H	−4.40766	−0.28298	+6.66329
C	−3.05944	−1.78906	+5.78318
O	−1.85832	−1.06167	+6.11552
H	−1.07494	−1.65187	+5.95057
H	−3.28967	−2.52036	+6.61079
C	−2.88101	−2.55887	+4.47600
O	−1.84872	−3.54960	+4.69460
H	−1.90207	−4.22495	+3.95412
H	−2.56551	−1.85618	+3.65440
C	−4.20056	−3.23844	+4.08659
H	−4.43938	−4.05917	+4.81392
C	−5.34846	−2.22103	+4.03384
H	−5.14592	−1.46452	+3.23334
C	−6.73778	−2.84147	+3.85786
H	−6.87569	−3.67273	+4.60107
H	−7.50841	−2.06866	+4.06745
O	−6.94236	−3.31688	+2.52820
H	−6.36791	−4.10874	+2.38589
O	+6.57271	−5.10419	−0.84016
H	+5.88625	−5.64289	−0.37515
C	+6.36655	−5.22138	−2.24731
H	+6.33717	−6.30198	−2.55476
H	+7.23085	−4.74697	−2.75981
C	+5.07763	−4.52450	−2.69321
H	+5.02518	−3.48402	−2.28173
O	+5.20204	−4.43885	−4.17106
C	+4.08629	−3.72844	−4.79188
O	+4.01541	−2.39146	−4.21299

H	+4.31488	−3.67255	−5.88997
C	+2.79960	−4.49810	−4.51498
O	+1.70820	−3.78370	−5.13330
H	+0.85188	−4.12648	−4.76065
H	+2.88356	−5.52192	−4.98066
C	+2.56530	−4.64911	−3.01320
O	+1.40510	−5.49622	−2.83453
H	+1.38850	−5.80096	−1.87870
H	+2.37283	−3.63930	−2.55305
C	+3.79093	−5.28381	−2.34212
H	+3.87859	−6.36197	−2.64113
O	+3.57783	−5.19692	−0.87993
C	+3.54171	−6.47291	−0.17576
O	+4.66290	−6.51146	+0.76093
C	+4.63882	−5.38984	+1.73507
C	+5.92083	−5.57618	+2.55195
O	+6.22224	−4.42277	+3.33598
H	+5.54598	−4.34435	+4.05290
H	+5.82221	−6.48485	+3.20579
H	+6.77075	−5.74925	+1.85743
H	+4.66583	−4.42516	+1.16572
H	+3.69398	−7.32490	−0.89139
C	+2.24200	−6.61037	+0.60913
O	+1.14407	−6.57092	−0.32741
H	+0.30215	−6.41304	+0.17801
H	+2.24231	−7.60679	+1.13755
C	+2.10927	−5.49530	+1.64474
O	+0.91933	−5.76394	+2.42397
H	+0.95581	−5.19736	+3.25130
H	+2.00734	−4.50083	+1.12582
C	+3.34423	−5.46880	+2.55567
H	+3.35463	−6.37711	+3.21478
O	+3.24191	−4.25513	+3.39734
C	+3.17881	−4.49667	+4.83460
O	+4.36377	−3.90681	+5.45370
C	+4.48429	−2.44721	+5.20177
C	+5.80871	−2.06107	+5.86711
O	+6.24499	−0.76332	+5.46573
H	+5.62953	−0.09155	+5.84973
H	+5.69881	−2.11433	+6.98432

H	+6.59049	−2.79213	+5.56854
H	+4.53222	−2.28224	+4.09546
H	+3.22079	−5.59767	+5.05218
C	+1.93708	−3.84445	+5.43173
O	+0.77459	−4.43421	+4.81182
H	−0.01390	−3.86113	+5.00927
H	+1.91466	−4.05886	+6.53891
C	+1.95483	−2.33224	+5.21694
O	+0.80818	−1.78206	+5.90777
H	+0.94555	−0.79205	+5.99899
H	+1.88383	−2.10012	+4.11688
C	+3.25250	−1.73545	+5.77806
H	+3.25236	−1.80057	+6.89848
O	+3.29369	−0.31296	+5.37071
C	+3.32738	+0.64888	+6.46710
O	+4.58979	+1.38128	+6.39862
C	+4.77573	+2.09717	+5.10962
C	+6.17024	+2.72014	+5.22556
O	+6.65597	+3.17119	+3.96210
H	+6.12314	+3.95555	+3.68169
H	+6.14575	+3.56557	+5.96557
H	+6.87750	+1.95406	+5.60997
H	+4.74409	+1.34580	+4.28028
H	+3.32048	+0.11883	+7.45725
C	+2.17568	+1.64017	+6.34400
O	+0.93776	+0.90161	+6.41083
H	+0.19823	+1.49184	+6.10248
H	+2.22506	+2.35863	+7.21225
C	+2.26669	+2.42981	+5.03926
O	+1.21442	+3.42338	+5.05734
H	+1.42839	+4.11744	+4.36468
H	+2.12385	+1.73985	+4.16078
C	+3.63721	+3.11161	+4.93389
H	+3.71981	+3.92722	+5.70048
O	+3.72750	+3.70221	+3.58029
C	+3.93507	+5.14542	+3.54036
O	+5.22207	+5.40546	+2.89916
C	+5.30854	+4.83162	+1.53147
C	+6.73976	+5.14189	+1.08313
O	+7.10595	+4.39237	−0.07460

H	+6.58186	+4.72518	−0.84435
H	+6.84451	+6.24423	+0.89103
H	+7.44218	+4.87243	+1.90090
H	+5.14476	+3.72602	+1.60057
H	+4.00572	+5.56396	+4.58011
C	+2.83610	+5.81998	+2.72631
O	+1.57178	+5.55164	+3.37007
H	+0.83918	+5.75756	+2.72935
H	+3.02090	+6.93251	+2.72130
C	+2.82881	+5.30579	+1.28829
O	+1.85700	+6.08267	+0.54806
H	+2.01440	+5.92286	−0.43000
H	+2.53493	+4.21886	+1.27540
C	+4.21794	+5.46168	+0.65473
H	+4.43907	+6.54730	+0.47564
O	+4.18473	+4.74826	−0.64177
C	+4.44465	+5.57259	−1.81624
O	+5.67374	+5.09721	−2.44796
C	+5.60452	+3.66598	−2.84204
C	+6.99823	+3.36537	−3.40111
O	+7.21235	+1.96447	−3.56691
H	+6.62855	+1.64167	−4.29655
H	+7.13651	+3.90531	−4.37702
H	+7.76367	+3.74681	−2.69168
H	+5.40251	+3.05566	−1.92509
H	+4.63179	+6.63890	−1.51713
C	+3.30084	+5.45289	−2.81687
O	+2.09229	+5.91998	−2.17958
H	+1.31557	+5.62782	−2.72813
H	+3.52859	+6.11008	−3.70474
C	+3.13505	+4.01070	−3.29195
O	+2.12225	+4.00806	−4.32646
H	+2.16760	+3.12462	−4.79999
H	+2.80452	+3.36047	−2.43483
C	+4.46230	+3.47891	−3.84983
H	+4.70685	+3.99390	−4.81655
O	+4.28427	+2.03013	−4.09883
C	+4.43333	+1.61448	−5.48897
O	+5.59036	+0.72634	−5.58182
H	+4.65892	+2.49920	−6.14309

C	+3.19504	+0.86037	−5.96068
O	+2.06132	+1.74683	−5.86113
H	+1.22872	+1.20910	−5.95084
H	+3.34294	+0.56918	−7.04065
C	+2.96922	−0.40389	−5.13297
O	+1.85113	−1.11246	−5.71875
H	+1.87245	−2.05891	−5.38616
H	+2.72994	−0.13013	−4.06657
C	+4.22967	−1.27823	−5.16392
H	+4.38188	−1.69558	−6.19463
C	+5.46509	−0.48058	−4.72386
H	+5.34158	−0.14854	−3.66158
C	+6.79684	−1.21326	−4.91305
H	+6.84082	−1.65629	−5.94486
H	+7.62792	−0.48107	−4.82319
O	+6.99212	−2.22253	−3.92353
H	+6.35313	−2.95760	−4.09400

345

* E = 1.465 kcal/mol

C	−0.55395	−0.14305	−1.41661
C	−0.45160	−0.74549	−0.15007
C	−1.54362	−1.09871	+0.63089
C	−2.91557	−0.93765	+0.42029
C	−3.67572	−0.09211	−0.38763
C	−3.31534	+1.15627	−0.90272
C	−2.12094	+1.80129	−0.57270
H	−1.13075	+1.07537	−1.14748
C	−4.31139	+1.78768	−1.82453
C	−5.68223	+1.91874	−1.15558
C	−6.02724	+0.69636	−0.30266
C	−5.11247	−0.46591	−0.63587
C	+0.71555	−0.03016	−2.21604
C	+1.87336	+0.60111	−1.43595
C	+1.84992	+0.24260	+0.05368
C	+0.95772	−0.98518	+0.29236
C	+3.17279	−0.27642	+0.62899
C	+2.74641	−0.99835	+1.91528
C	+1.26924	−1.38904	+1.72337
O	−5.82934	+0.95123	+1.07465
H	−6.57088	+1.45230	+1.42552

C	+1.34708	+1.43566	+0.85541
C	+4.26622	+0.74866	+0.82923
H	−7.06086	+0.39680	−0.47179
H	−5.25596	−0.73873	−1.68357
H	−5.39169	−1.33011	−0.03836
H	−6.43675	+2.07021	−1.92457
H	−5.69474	+2.79710	−0.51133
H	−3.95955	+2.76231	−2.15682
H	−4.42107	+1.17081	−2.71990
H	−1.72361	+1.58078	+0.41459
H	−2.00921	+2.83251	−0.88745
H	−3.51826	−1.62073	+1.01053
H	−1.30468	−1.69086	+1.50624
H	−1.45033	−0.39319	−1.98219
H	+0.53800	+0.52589	−3.13504
H	+1.00783	−1.03699	−2.53004
H	+2.81590	+0.28773	−1.88838
H	+1.82877	+1.68488	−1.54560
H	+3.53622	−1.02634	−0.07898
H	+2.85947	−0.33355	+2.77176
H	+3.37835	−1.86114	+2.11162
H	+0.62893	−0.84482	+2.41734
H	+1.08976	−2.44798	+1.89857
H	+1.31112	+1.23599	+1.92544
H	+0.34971	+1.72203	+0.53385
H	+1.99811	+2.29519	+0.70113
H	+5.18333	+0.27384	+1.16928
H	+3.98469	+1.48830	+1.57767
H	+4.49196	+1.27726	−0.09732
H	+1.36816	−1.76627	−0.36249
O	−6.85468	−4.13332	−1.71945
H	−6.13166	−4.57790	−2.22732
C	−6.91218	−4.70872	−0.41373
H	−7.01607	−5.82586	−0.47809
H	−7.81837	−4.31333	+0.09366
C	−5.67700	−4.35063	+0.41750
H	−5.46761	−3.25152	+0.36045
O	−6.06387	−4.67911	+1.81292
C	−5.02187	−4.33917	+2.78154
O	−4.73192	−2.91537	+2.66282

H	−5.44117	−4.57502	+3.79638
C	−3.78200	−5.16990	+2.46891
O	−2.76396	−4.84957	+3.43993
H	−1.89537	−5.21021	+3.11605
H	−4.04939	−6.26206	+2.56033
C	−3.28225	−4.89468	+1.05214
O	−2.18283	−5.80153	+0.79264
H	−2.01439	−5.80496	−0.19641
H	−2.92514	−3.83071	+0.97001
C	−4.41125	−5.13234	+0.04035
H	−4.63541	−6.22955	−0.03625
O	−3.93246	−4.63770	−1.26955
C	−3.87563	−5.63126	−2.33260
O	−4.79625	−5.21863	−3.39172
C	−4.48229	−3.86989	−3.92980
C	−5.59872	−3.59937	−4.94224
O	−5.63460	−2.22724	−5.33517
H	−4.83425	−2.03884	−5.88445
H	−5.46610	−4.26375	−5.83844
H	−6.57722	−3.84796	−4.47796
H	−4.52423	−3.13397	−3.08689
H	−4.23867	−6.62842	−1.96480
C	−2.46758	−5.71263	−2.91180
O	−1.56916	−6.12210	−1.85886
H	−0.63628	−5.93764	−2.15002
H	−2.45796	−6.48991	−3.72940
C	−2.03838	−4.36826	−3.49726
O	−0.75128	−4.56243	−4.13128
H	−0.58295	−3.78171	−4.73824
H	−1.94781	−3.60261	−2.67793
C	−3.06956	−3.89216	−4.52930
H	−3.04145	−4.55587	−5.43399
O	−2.69540	−2.51484	−4.92310
C	−2.39467	−2.33202	−6.33789
O	−3.37817	−1.40839	−6.90164
C	−3.38465	−0.08460	−6.22735
C	−4.51874	+0.68001	−6.91625
O	−4.87142	+1.86302	−6.19973
H	−4.13174	+2.51352	−6.28507
H	−4.21982	+0.93015	−7.96993

H	−5.41767	+0.02854	−6.96461
H	−3.61269	−0.24074	−5.14211
H	−2.51233	−3.29975	−6.89551
C	−1.00282	−1.73623	−6.51841
O	−0.04030	−2.65387	−5.95851
H	+0.82443	−2.17498	−5.84821
H	−0.80277	−1.61825	−7.62242
C	−0.90007	−0.36895	−5.84542
O	+0.40633	+0.17177	−6.15546
H	+0.39145	+1.15543	−5.95779
H	−1.01103	−0.47919	−4.73023
C	−1.99966	+0.55981	−6.37672
H	−1.80152	+0.81558	−7.45137
O	−1.96011	+1.79612	−5.56453
C	−1.68443	+3.02219	−6.30472
O	−2.85055	+3.89647	−6.19363
C	−3.19776	+4.21133	−4.78387
C	−4.47098	+5.05613	−4.88726
O	−5.14554	+5.14809	−3.63248
H	−4.60048	+5.70591	−3.02356
H	−4.21763	+6.07924	−5.27650
H	−5.16394	+4.57936	−5.61350
H	−3.40465	+3.24963	−4.24850
H	−1.55181	+2.80356	−7.39818
C	−0.47674	+3.74135	−5.71412
O	+0.67000	+2.87491	−5.84135
H	+1.40336	+3.23357	−5.27198
H	−0.29496	+4.68543	−6.30381
C	−0.72169	+4.10473	−4.25074
O	+0.41041	+4.88402	−3.79608
H	+0.14502	+5.35018	−2.94819
H	−0.81145	+3.16793	−3.63289
C	−2.01164	+4.92509	−4.12082
H	−1.86632	+5.94278	−4.57115
O	−2.28971	+5.06534	−2.67416
C	−2.36762	+6.43640	−2.18314
O	−3.72213	+6.66527	−1.68446
C	−4.09849	+5.70784	−0.61301
C	−5.56655	+6.02203	−0.31528
O	−6.16608	+5.00352	+0.48860

H	−5.78438	+5.06823	+1.39875
H	−5.64740	+7.02095	+0.19072
H	−6.12880	+6.07897	−1.27195
H	−3.99208	+4.66753	−1.01507
H	−2.20800	+7.16616	−3.02177
C	−1.37698	+6.65265	−1.04384
O	−0.04996	+6.39587	−1.55347
H	+0.57456	+6.30460	−0.78494
H	−1.44282	+7.72725	−0.70821
C	−1.68477	+5.73961	+0.14246
O	−0.79520	+6.11578	+1.22018
H	−1.13136	+5.68652	+2.06326
H	−1.50089	+4.66650	−0.14465
C	−3.14912	+5.90189	+0.57644
H	−3.30480	+6.91207	+1.04012
O	−3.43477	+4.85253	+1.57827
C	−3.79310	+5.33700	+2.90552
O	−5.17070	+4.93609	+3.18812
C	−5.37842	+3.46323	+3.16044
C	−6.86709	+3.30342	+3.48670
O	−7.36416	+2.00950	+3.12981
H	−6.92992	+1.32903	+3.70823
H	−7.04065	+3.49977	+4.57859
H	−7.44823	+4.05751	+2.91263
H	−5.15089	+3.08898	+2.12994
H	−3.77939	+6.45992	+2.93325
C	−2.87426	+4.72799	+3.95761
O	−1.52311	+5.14867	+3.67269
H	−0.89702	+4.56669	+4.18180
H	−3.17685	+5.11824	+4.97198
C	−2.98231	+3.20511	+3.95347
O	−2.15966	+2.70321	+5.03410
H	−2.43694	+1.75586	+5.21871
H	−2.61551	+2.79504	+2.97251
C	−4.44183	+2.78586	+4.16956
H	−4.75748	+3.02511	+5.22063
O	−4.50931	+1.32720	+3.95517
C	−5.00650	+0.54770	+5.07840
O	−6.24180	−0.11590	+4.66559
H	−5.27909	+1.21938	+5.93760

C	−3.98270	−0.50685	+5.48234
O	−2.76995	+0.17075	+5.87772
H	−2.03169	−0.49531	+5.89639
H	−4.38595	−1.08536	+6.36305
C	−3.71160	−1.47422	+4.33201
O	−2.81431	−2.49676	+4.83051
H	−2.85102	−3.27721	+4.20162
H	−3.23016	−0.93087	+3.47288
C	−5.03017	−2.10410	+3.86585
H	−5.43807	−2.77514	+4.66898
C	−6.05962	−1.02637	+3.49770
H	−5.70201	−0.42023	+2.62109
C	−7.46789	−1.57141	+3.24169
H	−7.76250	−2.27047	+4.07148
H	−8.19020	−0.72599	+3.24246
O	−7.56384	−2.22803	+1.97694
H	−7.02585	−3.05653	+2.01536
O	+6.04481	−5.72808	+1.00986
H	+5.24686	−6.12687	+1.43636
C	+6.02413	−6.04605	−0.38080
H	+5.91355	−7.15412	−0.53238
H	+7.00006	−5.74068	−0.81581
C	+4.89900	−5.31207	−1.11674
H	+4.91301	−4.21888	−0.87566
O	+5.23755	−5.48018	−2.55403
C	+4.30766	−4.79056	−3.44512
O	+4.32152	−3.36929	−3.11433
H	+4.68691	−4.95566	−4.48935
C	+2.91292	−5.37380	−3.24809
O	+2.00590	−4.68283	−4.13246
H	+1.07304	−4.88718	−3.85245
H	+2.93854	−6.46698	−3.52523
C	+2.46014	−5.23946	−1.79540
O	+1.18789	−5.91832	−1.67149
H	+1.01100	−6.07376	−0.69582
H	+2.33923	−4.15215	−1.52879
C	+3.49602	−5.88044	−0.86244
H	+3.49599	−6.99477	−0.99651
O	+3.09499	−5.55444	+0.52360
C	+2.81922	−6.70106	+1.38174

O	+3.78601	−6.69500	+2.47757
C	+3.75541	−5.43332	+3.26167
C	+4.87491	−5.60366	+4.29280
O	+5.20678	−4.36740	+4.92347
H	+4.44488	−4.08593	+5.48756
H	+4.57049	−6.36944	+5.05724
H	+5.78590	−5.98080	+3.78030
H	+3.97906	−4.58181	+2.56979
H	+2.97600	−7.66185	+0.82178
C	+1.41531	−6.60196	+1.96803
O	+0.46884	−6.61529	+0.87749
H	−0.41128	−6.30162	+1.21889
H	+1.23424	−7.49909	+2.62687
C	+1.26174	−5.32936	+2.79790
O	−0.04253	−5.36890	+3.42386
H	−0.06305	−4.66528	+4.13936
H	+1.33383	−4.42756	+2.12703
C	+2.35803	−5.25060	+3.86926
H	+2.17364	−6.01973	+4.66550
O	+2.28256	−3.89864	+4.46665
C	+1.99492	−3.86038	+5.89527
O	+3.14404	−3.26763	+6.57726
C	+3.46113	−1.90147	+6.08596
C	+4.72668	−1.50906	+6.85390
O	+5.35706	−0.36250	+6.28396
H	+4.78189	+0.42515	+6.44683
H	+4.47232	−1.32573	+7.93306
H	+5.45000	−2.35170	+6.81480
H	+3.66583	−1.95965	+4.98660
H	+1.88265	−4.90033	+6.30438
C	+0.76457	−3.00281	+6.16892
O	−0.36051	−3.59688	+5.48722
H	−1.09849	−2.93003	+5.45472
H	+0.56605	−3.00230	+7.27943
C	+0.98152	−1.56446	+5.70137
O	−0.17599	−0.79768	+6.11002
H	+0.06203	+0.17570	+6.06069
H	+1.08139	−1.53501	+4.58022
C	+2.25287	−0.99001	+6.34067
H	+2.09757	−0.84888	+7.44322

O	+2.50699	+0.32574	+5.71113
C	+2.52125	+1.46011	+6.62802
O	+3.85561	+2.05514	+6.60396
C	+4.26181	+2.52244	+5.25326
C	+5.69276	+3.03066	+5.45300
O	+6.35420	+3.25885	+4.20963
H	+5.93844	+4.04190	+3.77241
H	+5.67568	+3.96940	+6.07031
H	+6.27265	+2.26673	+6.01418
H	+4.24302	+1.64754	+4.55454
H	+2.34637	+1.11677	+7.68297
C	+1.50503	+2.51150	+6.19687
O	+0.19502	+1.90809	+6.22033
H	−0.43343	+2.49216	+5.71611
H	+1.53359	+3.36312	+6.93648
C	+1.82895	+3.05257	+4.80550
O	+0.89163	+4.11913	+4.52624
H	+1.24572	+4.65084	+3.75303
H	+1.71750	+2.23635	+4.03713
C	+3.26642	+3.58878	+4.77553
H	+3.34589	+4.50892	+5.41326
O	+3.57599	+3.94008	+3.37128
C	+3.89995	+5.34294	+3.13656
O	+5.28248	+5.42379	+2.67015
C	+5.52063	+4.63196	+1.43578
C	+7.02058	+4.79356	+1.17048
O	+7.49849	+3.83714	+0.22562
H	+7.11616	+4.05064	−0.66095
H	+7.23093	+5.83951	+0.81665
H	+7.57487	+4.64019	+2.12128
H	+5.26741	+3.56182	+1.64779
H	+3.85227	+5.92552	+4.09550
C	+2.98798	+5.93229	+2.06593
O	+1.62741	+5.85279	+2.54100
H	+1.01190	+6.02631	+1.77921
H	+3.26434	+7.01514	+1.91373
C	+3.14383	+5.18529	+0.74282
O	+2.34544	+5.87692	−0.24775
H	+2.63122	+5.55557	−1.15440
H	+2.77488	+4.12724	+0.85604

C	+4.61720	+5.16214	+0.31391
H	+4.94327	+6.18893	−0.00070
O	+4.71658	+4.24125	−0.84033
C	+5.21763	+4.83360	−2.07426
O	+6.47461	+4.17367	−2.42163
C	+6.32776	+2.70248	−2.56896
C	+7.75100	+2.20699	−2.84146
O	+7.85419	+0.79075	−2.70097
H	+7.35260	+0.36389	−3.43844
H	+8.07165	+2.52762	−3.86979
H	+8.44392	+2.67452	−2.10940
H	+5.93968	+2.28704	−1.60353
H	+5.45734	+5.92041	−1.92413
C	+4.22060	+4.61730	−3.20712
O	+2.98455	+5.27240	−2.84810
H	+2.26174	+4.92980	−3.43898
H	+4.63032	+5.08811	−4.14640
C	+3.99051	+3.12709	−3.45157
O	+3.13946	+3.00114	−4.61607
H	+3.18662	+2.04988	−4.93220
H	+3.48193	+2.66871	−2.55779
C	+5.32683	+2.41282	−3.69559
H	+5.75325	+2.72361	−4.68611
O	+5.04648	+0.95905	−3.71830
C	+5.37955	+0.27946	−4.96541
O	+6.43137	−0.69729	−4.69174
H	+5.80590	+1.00564	−5.70858
C	+4.16120	−0.45140	−5.51804
O	+3.12821	+0.52265	−5.77767
H	+2.26714	+0.04113	−5.90454
H	+4.45043	−0.95070	−6.48736
C	+3.66658	−1.51461	−4.53901
O	+2.58710	−2.23041	−5.18472
H	+2.43751	−3.08708	−4.68355
H	+3.28894	−1.02439	−3.59742
C	+4.80993	−2.47604	−4.18867
H	+5.08252	−3.09139	−5.08685
C	+6.03667	−1.70860	−3.67663
H	+5.77947	−1.17462	−2.72680
C	+7.29269	−2.56622	−3.49596

H	+7.46472	−3.18162	−4.42038
H	+8.17118	−1.89842	−3.36526
O	+7.20167	−3.40318	−2.34412
H	+6.51868	−4.09765	−2.51357

345

* E = 1.644 kcal/mol

C	−0.45443	+0.57792	+0.67392
C	−1.11590	−0.58936	+0.24308
C	−0.46007	−1.66152	−0.34257
C	+0.89004	−1.85440	−0.64473
C	+2.00177	−1.01380	−0.78439
C	+1.98346	+0.33439	−1.16941
C	+0.80868	+0.95588	−1.60007
H	+0.03580	+1.09280	−0.45680
C	+3.22864	+1.18738	−1.12021
C	+4.46394	+0.47961	−0.60014
C	+4.50219	−0.92975	−1.15141
C	+3.33579	−1.70538	−0.58473
C	−1.26951	+1.55969	+1.46902
C	−2.60631	+1.92602	+0.81559
C	−3.21832	+0.76223	+0.02996
C	−2.59208	−0.56890	+0.47400
C	−4.69380	+0.47334	+0.32880
C	−4.88695	−0.96537	−0.16895
C	−3.49612	−1.62584	−0.13655
O	+4.47077	−0.92224	−2.56202
H	+3.57727	−0.72598	−2.84806
C	−2.99846	+0.98062	−1.46073
C	−5.69694	+1.45985	−0.22425
H	+5.43354	−1.42069	−0.88750
H	+3.50847	−1.87050	+0.47972
H	+3.29877	−2.69259	−1.04898
H	+4.45035	+0.42745	+0.48863
H	+5.35606	+1.02666	−0.89308
H	+3.42793	+1.56318	−2.12588
H	+3.01821	+2.07207	−0.51662
H	+0.05207	+0.32221	−2.05186
H	+0.89653	+1.94621	−2.03164
H	+1.14348	−2.90516	−0.75121
H	−1.07160	−2.54207	−0.49938

H	+0.56763	+0.42715	+1.02064
H	−0.68760	+2.45778	+1.66907
H	−1.46553	+1.11011	+2.44764
H	−3.29501	+2.26066	+1.59245
H	−2.46220	+2.77752	+0.14970
H	−4.78365	+0.47176	+1.41874
H	−5.28317	−0.95829	−1.18416
H	−5.61085	−1.50052	+0.43962
H	−3.16187	−1.88057	−1.14291
H	−3.48353	−2.54917	+0.43935
H	−3.40858	+0.17121	−2.06291
H	−1.93698	+1.05905	−1.68507
H	−3.46840	+1.90821	−1.78400
H	−6.70346	+1.20355	+0.09700
H	−5.69385	+1.46389	−1.31324
H	−5.49078	+2.47367	+0.11885
H	−2.73904	−0.60401	+1.56198
O	−6.84852	−4.46852	−1.31996
H	−6.26073	−4.57318	−2.10846
C	−6.47641	−5.43783	−0.34064
H	−6.49858	−6.47282	−0.77869
H	−7.22706	−5.40095	+0.47797
C	−5.08824	−5.15270	+0.24034
H	−4.99883	−4.07666	+0.53717
O	−5.02664	−5.98296	+1.47080
C	−3.77758	−5.80839	+2.20932
O	−3.63625	−4.39524	+2.54490
H	−3.86823	−6.42878	+3.14102
C	−2.62177	−6.26685	+1.32732
O	−1.39475	−6.10900	+2.07110
H	−0.63000	−6.18008	+1.43880
H	−2.76747	−7.35762	+1.08080
C	−2.57308	−5.46198	+0.03050
O	−1.53833	−6.03744	−0.80216
H	−1.65162	−5.67322	−1.73061
H	−2.32380	−4.38697	+0.25638
C	−3.92686	−5.52891	−0.69022
H	−4.08357	−6.55496	−1.11720
O	−3.87933	−4.54181	−1.79188
C	−4.04952	−5.08863	−3.13230

O	−5.26542	−4.51623	−3.70730
C	−5.23042	−3.03183	−3.76248
C	−6.60995	−2.64095	−4.30014
O	−6.87348	−1.25037	−4.11405
H	−6.28951	−0.73679	−4.72498
H	−6.68522	−2.91606	−5.38681
H	−7.38808	−3.21318	−3.75075
H	−5.08612	−2.64172	−2.72216
H	−4.20856	−6.19956	−3.08980
C	−2.86342	−4.71327	−4.01339
O	−1.67269	−5.28293	−3.42987
H	−0.87671	−4.86020	−3.85258
H	−3.02119	−5.15577	−5.03899
C	−2.73387	−3.19588	−4.14036
O	−1.66965	−2.92870	−5.08322
H	−1.75759	−1.97864	−5.39319
H	−2.48035	−2.74578	−3.13903
C	−4.05324	−2.59792	−4.64670
H	−4.23124	−2.90750	−5.71069
O	−3.92091	−1.12458	−4.58309
C	−4.08550	−0.43011	−5.85555
O	−5.25319	+0.44288	−5.75487
C	−5.13676	+1.43805	−4.65857
C	−6.47921	+2.17439	−4.67476
O	−6.66054	+2.96431	−3.49965
H	−6.03403	+3.72813	−3.53754
H	−6.54909	+2.81381	−5.59569
H	−7.30173	+1.42847	−4.71631
H	−4.99895	+0.88750	−3.69256
H	−4.30591	−1.16175	−6.67855
C	−2.85929	+0.42168	−6.16597
O	−1.71472	−0.45349	−6.25007
H	−0.88839	+0.09704	−6.19396
H	−3.01470	+0.92512	−7.16343
C	−2.64378	+1.49125	−5.09672
O	−1.54823	+2.32980	−5.53543
H	−1.55203	+3.16148	−4.97476
H	−2.38108	+1.00536	−4.11619
C	−3.91685	+2.33132	−4.92252
H	−4.08673	+2.96731	−5.83140

O	−3.70880	+3.20627	−3.74619
C	−3.74929	+4.63915	−4.01341
O	−4.88777	+5.20870	−3.29485
C	−4.81842	+4.98199	−1.82787
C	−6.12623	+5.57398	−1.29438
O	−6.38137	+5.16373	+0.04856
H	−5.73575	+5.61933	+0.64289
H	−6.09199	+6.69452	−1.36618
H	−6.96838	+5.21910	−1.92658
H	−4.77820	+3.87785	−1.64127
H	−3.93217	+4.83236	−5.10445
C	−2.47131	+5.31023	−3.52155
O	−1.35829	+4.73304	−4.23546
H	−0.51578	+4.98773	−3.77180
H	−2.53027	+6.41214	−3.75587
C	−2.30066	+5.13112	−2.01361
O	−1.13596	+5.89090	−1.61165
H	−1.17092	+6.01191	−0.61621
H	−2.14781	+4.04178	−1.76941
C	−3.54672	+5.64341	−1.27955
H	−3.61549	+6.75993	−1.36941
O	−3.39390	+5.27605	+0.14630
C	−3.39612	+6.39347	+1.08447
O	−4.55869	+6.25701	+1.95923
C	−4.57896	+4.96399	+2.69032
C	−5.89380	+4.99451	+3.47547
O	−6.24570	+3.70211	+3.96739
H	−5.59925	+3.44481	+4.67027
H	−5.81275	+5.73169	+4.31992
H	−6.70958	+5.33779	+2.80359
H	−4.58532	+4.13288	+1.93879
H	−3.51902	+7.36740	+0.53914
C	−2.13377	+6.37234	+1.93979
O	−0.99542	+6.50408	+1.06039
H	−0.17762	+6.23607	+1.55824
H	−2.16008	+7.25025	+2.64682
C	−2.04307	+5.07797	+2.74527
O	−0.90095	+5.18713	+3.62925
H	−0.97096	+4.45610	+4.31301
H	−1.89809	+4.20940	+2.04570

C	−3.32224	+4.85821	+3.56466
H	−3.36947	+5.59509	+4.40970
O	−3.25177	+3.48511	+4.11505
C	−3.27191	+3.39278	+5.57057
O	−4.48205	+2.67715	+5.96928
C	−4.56718	+1.31515	+5.38110
C	−5.92862	+0.78853	+5.84427
O	−6.31304	−0.38371	+5.12698
H	−5.72010	−1.12671	+5.39940
H	−5.89862	+0.58508	+6.94889
H	−6.69942	+1.56831	+5.66396
H	−4.53634	+1.40938	+4.26566
H	−3.34073	+4.41538	+6.02981
C	−2.05539	+2.62344	+6.07319
O	−0.87021	+3.33438	+5.65510
H	−0.08606	+2.73258	+5.76736
H	−2.08965	+2.59170	+7.20010
C	−2.04392	+1.19348	+5.53490
O	−0.93866	+0.50183	+6.16250
H	−1.07060	−0.48356	+6.02476
H	−1.89926	+1.20576	+4.41719
C	−3.36716	+0.49027	+5.86574
H	−3.44003	+0.30955	+6.97093
O	−3.36325	−0.80950	+5.15685
C	−3.45778	−1.98606	+6.01511
O	−4.70477	−2.68243	+5.70730
H	−3.52344	−1.68118	+7.09414
C	−2.28870	−2.93163	+5.76316
O	−1.06518	−2.22614	+6.05945
H	−0.30333	−2.74236	+5.68109
H	−2.38478	−3.81336	+6.46031
C	−2.28520	−3.43741	+4.32164
O	−1.22102	−4.41153	+4.20789
H	−1.36678	−4.93592	+3.36479
H	−2.09740	−2.58274	+3.61334
C	−3.63682	−4.08481	+3.99189
H	−3.75993	−5.03583	+4.57495
C	−4.79457	−3.12250	+4.29064
H	−4.71440	−2.21948	+3.63347
C	−6.18708	−3.75147	+4.18488

H	−6.21172	−4.71141	+4.76807
H	−6.93119	−3.06105	+4.63713
O	−6.56546	−3.97802	+2.82782
H	−6.00913	−4.71008	+2.46466
O	+6.38093	−4.55087	−2.59452
H	+5.76309	−5.18641	−2.15758
C	+5.98475	−4.40077	−3.95821
H	+5.92535	−5.40359	−4.46376
H	+6.76697	−3.80632	−4.47702
C	+4.64119	−3.67699	−4.08666
H	+4.64001	−2.72046	−3.49416
O	+4.55692	−3.33973	−5.53579
C	+3.35924	−2.58410	−5.87938
O	+3.34011	−1.35689	−5.08881
H	+3.43236	−2.34544	−6.97464
C	+2.12900	−3.42447	−5.55750
O	+0.95789	−2.64696	−5.89012
H	+0.16610	−3.06898	−5.46101
H	+2.14732	−4.35604	−6.19286
C	+2.11201	−3.82552	−4.08332
O	+0.98849	−4.71885	−3.89013
H	+1.11887	−5.19899	−3.01935
H	+1.98595	−2.91212	−3.43680
C	+3.42247	−4.53339	−3.71385
H	+3.47409	−5.53244	−4.22373
O	+3.40611	−4.73946	−2.24556
C	+3.48300	−6.12945	−1.81047
O	+4.72261	−6.31010	−1.05694
C	+4.82013	−5.40377	+0.11500
C	+6.20657	−5.69003	+0.69879
O	+6.59256	−4.70117	+1.65324
H	+6.02161	−4.79799	+2.45452
H	+6.21679	−6.71315	+1.16312
H	+6.95445	−5.68260	−0.12274
H	+4.75389	−4.34657	−0.24919
H	+3.54518	−6.81783	−2.69546
C	+2.30789	−6.47445	−0.90196
O	+1.08823	−6.29470	−1.65250
H	+0.32377	−6.28623	−1.01653
H	+2.39692	−7.55585	−0.59387

C	+2.30415	−5.59783	+0.34894
O	+1.24141	−6.07223	+1.21001
H	+1.37608	−5.66755	+2.11815
H	+2.11146	−4.52527	+0.06312
C	+3.65500	−5.69423	+1.07115
H	+3.77277	−6.70994	+1.53358
O	+3.65415	−4.66793	+2.13869
C	+3.78600	−5.18073	+3.49733
O	+5.03587	−4.67260	+4.06028
C	+5.10058	−3.18841	+4.07530
C	+6.49861	−2.87911	+4.61876
O	+6.85521	−1.51235	+4.41516
H	+6.29546	−0.95017	+5.00517
H	+6.54612	−3.14237	+5.71010
H	+7.24198	−3.50957	+4.08530
H	+4.99284	−2.81924	+3.02362
H	+3.87164	−6.30045	+3.49101
C	+2.62776	−4.70190	+4.36550
O	+1.39986	−5.21084	+3.80360
H	+0.63844	−4.71144	+4.20373
H	+2.76045	−5.12037	+5.40469
C	+2.59526	−3.17668	+4.44348
O	+1.54901	−2.81084	+5.37467
H	+1.68797	−1.85268	+5.63763
H	+2.37013	−2.74453	+3.42887
C	+3.94933	−2.64689	+4.93389
H	+4.10247	−2.92316	+6.01081
O	+3.91434	−1.17241	+4.80800
C	+4.08961	−0.43536	+6.05416
O	+5.31736	+0.35137	+5.95357
C	+5.30566	+1.29408	+4.80527
C	+6.69055	+1.94719	+4.84063
O	+6.97752	+2.64483	+3.62932
H	+6.39409	+3.44145	+3.57872
H	+6.75895	+2.64006	+5.72266
H	+7.46043	+1.15651	+4.97032
H	+5.16963	+0.70289	+3.86425
H	+4.23339	−1.14197	+6.91505
C	+2.91824	+0.51329	+6.28341
O	+1.71227	−0.27266	+6.38621

H	+0.93073	+0.33631	+6.29743
H	+3.08505	+1.06391	+7.25360
C	+2.80650	+1.52754	+5.14681
O	+1.75314	+2.45817	+5.49366
H	+1.84720	+3.26625	+4.90663
H	+2.54721	+1.00039	+4.18650
C	+4.13613	+2.27359	+4.97436
H	+4.31516	+2.94748	+5.85376
O	+4.02064	+3.09183	+3.74674
C	+4.17606	+4.52933	+3.93717
O	+5.35644	+4.96527	+3.19414
C	+5.27282	+4.64674	+1.74536
C	+6.62017	+5.10499	+1.17919
O	+6.84842	+4.58252	−0.12892
H	+6.22798	+5.02636	−0.75807
H	+6.66486	+6.22768	+1.17102
H	+7.43412	+4.73816	+1.84081
H	+5.15233	+3.53920	+1.63392
H	+4.37240	+4.76685	+5.01705
C	+2.95792	+5.27242	+3.40048
O	+1.80010	+4.83944	+4.14559
H	+0.98363	+5.15789	+3.67445
H	+3.10945	+6.37818	+3.56220
C	+2.77048	+5.00710	+1.90760
O	+1.67129	+5.83246	+1.45376
H	+1.71065	+5.87992	+0.45202
H	+2.52563	+3.92100	+1.73865
C	+4.05451	+5.36250	+1.14597
H	+4.21098	+6.47376	+1.15607
O	+3.87732	+4.90621	−0.25065
C	+3.96402	+5.95371	−1.26293
O	+5.10688	+5.66734	−2.12607
C	+5.01436	+4.34232	−2.79225
C	+6.32102	+4.22730	−3.58358
O	+6.55255	+2.89406	−4.03299
H	+5.88814	+2.67472	−4.73131
H	+6.29945	+4.94131	−4.45132
H	+7.16848	+4.51911	−2.92641
H	+4.95749	+3.55146	−2.00119
H	+4.16770	+6.94911	−0.78454

C	+2.69835	+5.97699	−2.11182
O	+1.57988	+6.26259	−1.24502
H	+0.73910	+6.04480	−1.72978
H	+2.79193	+6.79935	−2.87791
C	+2.49856	+4.64488	−2.83217
O	+1.34819	+4.78936	−3.69822
H	+1.35857	+4.03431	−4.35987
H	+2.30895	+3.82583	−2.08179
C	+3.74685	+4.30164	−3.65650
H	+3.84157	+5.01239	−4.51985
O	+3.57312	+2.92538	−4.17289
C	+3.55229	+2.80778	−5.63001
O	+4.69778	+2.00801	−6.04826
H	+3.67944	+3.81715	−6.10608
C	+2.27285	+2.11890	−6.08876
O	+1.14932	+2.93079	−5.68624
H	+0.31982	+2.38859	−5.77537
H	+2.28779	+2.04050	−7.21369
C	+2.17359	+0.71891	−5.48947
O	+0.98843	+0.08904	−6.03210
H	+1.06656	−0.90083	−5.88137
H	+2.08839	+0.78713	−4.36776
C	+3.41909	−0.09697	−5.85816
H	+3.40985	−0.33135	−6.95610
C	+4.71505	+0.63368	−5.47604
H	+4.79262	+0.71047	−4.36180
C	+5.98185	+0.00822	−6.07095
H	+5.80260	−0.24017	−7.15494
H	+6.80563	+0.75369	−6.03092
O	+6.40304	−1.14617	−5.35089
H	+5.68949	−1.82878	−5.39522

345

* E = 1.645 kcal/mol

C	+0.58033	+0.47198	+0.65693
C	+1.20460	+0.37999	−0.60906
C	+0.47016	+0.29967	−1.78273
C	−0.90920	+0.26287	−2.01242
C	−2.02985	−0.06854	−1.24513
C	−2.05245	−0.93615	−0.13920
C	−0.92529	−1.65634	+0.24546

H	−0.04219	−0.66317	+0.73529
C	−3.29729	−1.11838	+0.69788
C	−4.49101	−0.29137	+0.26150
C	−4.54948	−0.24286	−1.25028
C	−3.33647	+0.50142	−1.75808
C	+1.37386	+0.69820	+1.93195
C	+2.88879	+0.77598	+1.74953
C	+3.34477	−0.13296	+0.62812
C	+2.70170	+0.37210	−0.66074
C	+4.82469	−0.02886	+0.24989
C	+4.85787	−0.55971	−1.19407
C	+3.43310	−0.39205	−1.75536
O	−4.61491	−1.54013	−1.80221
H	−3.74341	−1.93637	−1.75582
C	+2.97124	−1.58108	+0.92384
C	+5.80385	−0.71302	+1.17684
H	−5.45403	+0.25689	−1.58234
H	−3.42072	+1.54926	−1.46559
H	−3.33092	+0.48390	−2.84903
H	−4.41295	+0.72894	+0.63709
H	−5.40508	−0.72020	+0.66319
H	−3.56897	−2.17521	+0.67572
H	−3.05060	−0.90419	+1.73890
H	−0.18550	−1.89310	−0.51309
H	−1.04353	−2.39838	+1.02636
H	−1.16954	+0.59051	−3.01485
H	+1.05804	+0.37562	−2.69043
H	−0.38074	+0.98675	+0.62608
H	+1.13867	−0.10720	+2.63052
H	+1.01903	+1.60809	+2.41595
H	+3.18008	+1.79799	+1.49716
H	+3.38327	+0.53154	+2.69100
H	+5.05987	+1.03889	+0.22891
H	+5.15106	−1.60955	−1.19902
H	+5.59740	−0.03128	−1.78980
H	+2.95458	−1.35837	−1.91850
H	+3.42000	+0.13082	−2.70971
H	+3.38421	−2.26458	+0.18365
H	+1.89352	−1.73105	+0.94086
H	+3.36192	−1.87754	+1.89656

H	+6.82658	−0.53068	+0.85645
H	+5.65261	−1.79142	+1.18675
H	+5.70983	−0.34968	+2.19950
H	+3.01549	+1.42076	−0.74247
O	−6.78853	−3.76011	−3.30942
H	−6.08684	−3.51190	−3.96010
C	−6.41234	−4.97530	−2.66843
H	−6.31642	−5.80973	−3.41893
H	−7.22452	−5.25302	−1.96196
C	−5.09969	−4.83791	−1.88894
H	−5.09420	−3.90011	−1.27799
O	−5.11259	−6.00688	−0.96706
C	−3.93352	−6.09575	−0.11305
O	−3.84666	−4.87615	+0.68723
H	−4.08744	−6.98987	+0.54912
C	−2.69638	−6.24178	−0.99085
O	−1.54031	−6.35064	−0.13319
H	−0.72506	−6.19186	−0.68067
H	−2.79417	−7.18507	−1.60117
C	−2.56530	−5.04660	−1.93036
O	−1.43087	−5.28570	−2.79737
H	−1.50830	−4.66896	−3.58591
H	−2.39556	−4.10661	−1.33283
C	−3.84124	−4.89483	−2.76772
H	−3.91569	−5.73859	−3.50468
O	−3.71150	−3.61689	−3.49912
C	−3.80459	−3.70822	−4.95229
O	−4.97988	−2.96420	−5.38807
C	−4.94996	−1.53541	−4.96760
C	−6.29470	−0.98475	−5.45088
O	−6.56581	+0.30937	−4.91141
H	−5.93439	+0.95356	−5.31471
H	−6.30904	−0.95335	−6.57470
H	−7.10558	−1.66877	−5.12099
H	−4.87426	−1.49290	−3.84564
H	−3.96146	−4.77391	−5.27116
C	−2.56267	−3.09755	−5.59186
O	−1.41178	−3.83553	−5.12524
H	−0.59321	−3.31382	−5.34220
H	−2.64077	−3.20350	−6.71188

C	−2.43422	−1.61644	−5.23854
O	−1.31446	−1.08763	−5.99003
H	−1.37515	−0.08669	−5.97431
H	−2.23948	−1.49900	−4.13559
C	−3.72340	−0.86988	−5.60840
H	−3.83689	−0.82976	−6.72463
O	−3.59260	+0.51116	−5.08529
C	−3.64302	+1.56863	−6.08845
O	−4.82594	+2.38983	−5.83601
C	−4.83546	+2.98479	−4.47540
C	−6.17572	+3.72141	−4.39583
O	−6.49155	+4.10080	−3.05645
H	−5.86484	+4.81175	−2.77495
H	−6.15198	+4.62223	−5.06702
H	−6.98137	+3.04833	−4.75948
H	−4.79521	+2.15382	−3.72546
H	−3.77111	+1.13286	−7.11540
C	−2.40621	+2.45574	−5.99559
O	−1.24392	+1.63782	−6.24632
H	−0.43561	+2.13819	−5.95438
H	−2.47572	+3.25367	−6.78986
C	−2.30919	+3.12363	−4.62513
O	−1.18845	+4.03931	−4.66565
H	−1.25830	+4.64830	−3.87135
H	−2.13462	+2.34429	−3.83087
C	−3.60398	+3.88638	−4.31323
H	−3.68901	+4.78698	−4.97737
O	−3.52373	+4.32606	−2.90136
C	−3.56141	+5.76918	−2.69448
O	−4.76586	+6.09234	−1.93158
C	−4.82283	+5.39600	−0.62045
C	−6.17709	+5.80736	−0.03539
O	−6.54132	+4.99382	+1.07909
H	−5.93833	+5.20538	+1.83347
H	−6.14760	+6.89148	+0.25857
H	−6.95947	+5.68881	−0.81544
H	−4.78881	+4.29203	−0.80483
H	−3.65245	+6.30714	−3.67602
C	−2.34238	+6.22921	−1.90274
O	−1.15975	+5.90839	−2.66478

H	−0.37020	+5.97212	−2.06309
H	−2.40308	+7.34747	−1.76684
C	−2.29748	+5.56248	−0.52916
O	−1.18680	+6.13861	+0.19900
H	−1.30289	+5.91537	+1.17009
H	−2.14241	+4.45366	−0.64513
C	−3.61098	+5.81721	+0.22203
H	−3.68878	+6.90098	+0.50293
O	−3.57747	+4.99476	+1.45220
C	−3.66009	+5.73913	+2.70356
O	−4.88750	+5.34428	+3.39196
C	−4.95196	+3.88455	+3.66086
C	−6.32680	+3.67983	+4.30404
O	−6.69717	+2.30199	+4.33289
H	−6.11400	+1.83222	+4.97854
H	−6.32695	+4.11204	+5.34132
H	−7.08975	+4.22637	+3.70912
H	−4.88682	+3.34231	+2.68348
H	−3.74624	+6.84088	+2.50405
C	−2.47067	+5.41163	+3.59942
O	−1.26456	+5.81210	+2.91558
H	−0.48677	+5.40519	+3.38372
H	−2.56680	+6.00115	+4.55614
C	−2.43298	+3.92136	+3.93257
O	−1.35212	+3.71119	+4.87258
H	−1.48301	+2.81305	+5.30049
H	−2.24458	+3.32421	+2.99695
C	−3.76765	+3.48677	+4.55254
H	−3.88028	+3.93568	+5.57496
O	−3.73599	+2.01177	+4.66666
C	−3.87330	+1.48703	+6.02040
O	−5.09919	+0.69428	+6.08299
C	−5.11454	−0.42065	+5.10098
C	−6.49480	−1.06038	+5.27806
O	−6.80923	−1.94406	+4.20280
H	−6.22903	−2.74181	+4.27091
H	−6.53678	−1.60203	+6.26159
H	−7.26475	−0.25940	+5.29906
H	−5.00668	+0.01191	+4.07388
H	−3.99596	+2.32393	+6.75906

C	−2.69053	+0.58896	+6.36429
O	−1.48723	+1.38378	+6.30431
H	−0.70357	+0.77095	+6.30282
H	−2.82510	+0.20024	+7.41441
C	−2.60613	−0.59387	+5.40118
O	−1.53440	−1.45188	+5.85930
H	−1.64014	−2.34615	+5.41624
H	−2.38151	−0.22682	+4.36035
C	−3.93604	−1.35947	+5.39346
H	−4.08554	−1.87781	+6.37758
O	−3.85527	−2.37031	+4.31561
C	−3.99522	−3.75616	+4.75148
O	−5.19835	−4.31305	+4.13897
H	−4.15144	−3.80617	+5.86250
C	−2.79051	−4.57774	+4.30793
O	−1.61076	−4.01757	+4.92266
H	−0.80863	−4.39484	+4.47109
H	−2.92567	−5.63848	+4.66648
C	−2.65881	−4.57946	+2.78610
O	−1.56528	−5.46475	+2.44771
H	−1.63437	−5.68817	+1.47108
H	−2.43347	−3.53849	+2.41709
C	−3.96448	−5.07366	+2.14881
H	−4.10628	−6.16563	+2.36578
C	−5.16986	−4.26906	+2.65381
H	−5.07073	−3.20056	+2.33280
C	−6.52885	−4.83947	+2.23567
H	−6.55376	−5.94357	+2.44514
H	−7.32475	−4.36147	+2.84667
O	−6.81369	−4.57984	+0.86300
H	−6.19912	−5.11584	+0.30424
O	+6.56086	−3.45759	−3.44963
H	+5.92189	−4.18415	−3.24411
C	+6.25372	−2.93511	−4.74214
H	+6.22465	−3.75907	−5.50584
H	+7.06783	−2.23553	−5.02970
C	+4.92139	−2.17983	−4.74439
H	+4.87273	−1.46397	−3.88505
O	+4.94378	−1.39591	−6.00680
C	+3.76784	−0.54180	−6.15826

O	+3.69629	+0.34428	−5.00162
H	+3.91993	+0.04957	−7.10070
C	+2.52602	−1.42342	−6.22770
O	+1.37497	−0.56621	−6.38439
H	+0.55454	−1.09682	−6.19496
H	+2.61131	−2.09753	−7.12777
C	+2.39403	−2.28082	−4.97015
O	+1.27862	−3.18097	−5.17173
H	+1.33534	−3.90351	−4.47794
H	+2.19585	−1.62361	−4.07773
C	+3.68117	−3.08355	−4.73534
H	+3.77810	−3.88734	−5.51265
O	+3.56638	−3.70813	−3.39792
C	+3.63469	−5.16424	−3.38262
O	+4.81349	−5.56005	−2.61433
C	+4.79074	−5.05547	−1.21729
C	+6.13501	−5.50363	−0.63654
O	+6.41179	−4.86590	+0.60996
H	+5.79655	−5.23258	+1.29140
H	+6.14154	−6.62075	−0.51912
H	+6.94483	−5.23254	−1.34749
H	+4.72205	−3.93687	−1.24397
H	+3.78387	−5.56394	−4.42150
C	+2.39288	−5.74984	−2.71999
O	+1.24015	−5.33749	−3.48368
H	+0.42358	−5.49837	−2.93845
H	+2.46901	−6.87525	−2.74292
C	+2.27453	−5.29025	−1.26751
O	+1.14461	−5.98198	−0.68558
H	+1.21447	−5.91055	+0.31259
H	+2.10313	−4.17798	−1.22734
C	+3.56081	−5.63414	−0.50427
H	+3.65974	−6.74721	−0.40018
O	+3.45397	−5.02588	+0.84104
C	+3.49836	−5.96703	+1.95497
O	+4.69123	−5.68455	+2.75085
C	+4.72328	−4.29591	+3.27785
C	+6.06343	−4.20636	+4.01418
O	+6.41811	−2.85515	+4.30563
H	+5.79720	−2.50779	+4.99233

H	+6.01825	−4.81618	+4.95700
H	+6.85986	−4.63725	+3.37006
H	+4.70021	−3.58689	+2.41057
H	+3.60969	−7.01922	+1.57853
C	+2.26834	−5.80698	+2.84168
O	+1.09906	−6.09356	+2.04601
H	+0.29602	−5.76052	+2.52890
H	+2.33367	−6.55238	+3.68574
C	+2.19344	−4.39748	+3.42504
O	+1.07308	−4.36262	+4.34171
H	+1.16493	−3.54252	+4.91231
H	+2.03210	−3.64928	+2.59978
C	+3.49428	−4.06346	+4.16761
H	+3.56893	−4.67553	+5.10538
O	+3.43598	−2.62767	+4.52425
C	+3.49806	−2.33516	+5.95169
O	+4.71571	−1.56945	+6.21090
C	+4.78050	−0.30795	+5.42922
C	+6.14777	+0.28489	+5.78253
O	+6.52128	+1.32476	+4.87935
H	+5.93463	+2.10499	+5.03765
H	+6.13376	+0.66453	+6.83995
H	+6.91649	−0.51501	+5.71966
H	+4.72923	−0.56262	+4.33878
H	+3.58505	−3.28367	+6.54654
C	+2.29428	−1.50197	+6.37803
O	+1.10029	−2.26905	+6.11285
H	+0.31644	−1.65809	+6.15008
H	+2.36749	−1.30117	+7.48540
C	+2.25854	−0.17142	+5.62868
O	+1.16774	+0.60994	+6.17209
H	+1.29385	+1.56230	+5.88209
H	+2.08323	−0.35739	+4.53178
C	+3.58571	+0.57758	+5.80874
H	+3.68493	+0.93667	+6.86729
O	+3.55240	+1.74351	+4.89735
C	+3.67464	+3.04685	+5.54152
O	+4.90612	+3.67700	+5.07120
C	+4.94414	+3.83328	+3.59404
C	+6.32947	+4.42239	+3.31215

O	+6.66388	+4.34657	+1.92695
H	+6.08222	+4.97163	+1.42805
H	+6.36740	+5.48647	+3.67134
H	+7.09112	+3.84585	+3.87979
H	+4.84113	+2.81980	+3.12862
H	+3.78049	+2.92914	+6.65335
C	+2.49374	+3.93795	+5.17190
O	+1.28482	+3.29633	+5.63284
H	+0.50803	+3.73433	+5.19249
H	+2.61096	+4.92834	+5.69834
C	+2.43398	+4.17690	+3.66423
O	+1.37743	+5.13492	+3.41584
H	+1.48723	+5.48004	+2.47976
H	+2.20234	+3.21125	+3.13371
C	+3.77430	+4.72430	+3.15524
H	+3.92466	+5.77457	+3.52086
O	+3.71113	+4.72476	+1.67618
C	+3.85533	+6.03293	+1.04669
O	+5.07152	+6.02124	+0.23673
C	+5.05820	+4.97228	−0.81593
C	+6.43462	+5.08931	−1.47723
O	+6.71677	+3.96874	−2.31442
H	+6.12530	+4.01177	−3.10560
H	+6.49080	+6.04594	−2.06385
H	+7.21400	+5.13058	−0.68622
H	+4.93855	+3.97510	−0.32047
H	+3.99901	+6.83070	+1.82407
C	+2.66349	+6.32705	+0.14282
O	+1.46970	+6.32669	+0.95403
H	+0.67968	+6.27984	+0.35095
H	+2.80135	+7.35038	−0.31138
C	+2.55419	+5.29601	−0.97912
O	+1.47925	+5.71725	−1.85155
H	+1.56378	+5.21296	−2.71497
H	+2.32151	+4.28182	−0.54937
C	+3.87428	+5.22840	−1.75846
H	+4.03142	+6.18123	−2.33029
O	+3.76653	+4.09975	−2.70967
C	+3.88551	+4.45978	−4.11843
O	+5.07825	+3.81096	−4.65893

H	+4.04678	+5.56556	−4.23070
C	+2.66810	+3.98031	−4.90113
O	+1.49830	+4.62729	−4.35780
H	+0.68961	+4.15902	−4.69962
H	+2.79229	+4.28583	−5.97986
C	+2.52859	+2.46104	−4.82748
O	+1.43269	+2.08261	−5.69379
H	+1.48467	+1.09188	−5.84591
H	+2.30449	+2.14434	−3.77010
C	+3.82709	+1.78865	−5.29338
H	+3.96223	+1.93378	−6.39789
C	+5.04155	+2.33161	−4.52806
H	+4.94728	+2.07374	−3.44216
C	+6.39498	+1.87291	−5.07904
H	+6.41933	+2.02108	−6.19255
H	+7.19785	+2.50066	−4.63611
O	+6.66789	+0.51214	−4.74668
H	+6.05220	−0.06687	−5.25993

345

* E = 1.721 kcal/mol

C	+0.36730	+1.05564	+0.12758
C	+0.69700	+0.00579	−0.75355
C	−0.26872	−0.70117	−1.45979
C	−1.66448	−0.61341	−1.46494
C	−2.60449	−0.10975	−0.56488
C	−2.45749	+0.04793	+0.82151
C	−1.33337	−0.39604	+1.51575
H	−0.32436	+0.42505	+1.07158
C	−3.56077	+0.78745	+1.51872
C	−4.94121	+0.18432	+1.23383
C	−5.02009	−0.45641	−0.14708
C	−3.99471	+0.17064	−1.07874
C	+1.42086	+1.91705	+0.79649
C	+2.86674	+1.54936	+0.46666
C	+3.00982	+0.05513	+0.26661
C	+2.14729	−0.32944	−0.93365
C	+4.38087	−0.41837	−0.22440
C	+4.07252	−1.77650	−0.87807
C	+2.57973	−1.75301	−1.25570
O	−4.81067	−1.85103	−0.07849

H	−3.88440	−2.00239	+0.11687
C	+2.60622	−0.69875	+1.52909
C	+5.48302	−0.47251	+0.80918
H	−6.01861	−0.34715	−0.55602
H	−4.16908	+1.24811	−1.13339
H	−4.11185	−0.22481	−2.08581
H	−5.69441	+0.96294	+1.32878
H	−5.18313	−0.58629	+1.96258
H	−3.37466	+0.82548	+2.59013
H	−3.55674	+1.82178	+1.16481
H	−0.79659	−1.24655	+1.10282
H	−1.35690	−0.34852	+2.59762
H	−2.11032	−1.01215	−2.37107
H	+0.12550	−1.37502	−2.21126
H	−0.54649	+1.58362	−0.14462
H	+1.27636	+1.86271	+1.87752
H	+1.23973	+2.96011	+0.53839
H	+3.17451	+2.04491	−0.45669
H	+3.52600	+1.91418	+1.25605
H	+4.68144	+0.28009	−1.01044
H	+4.27879	−2.58439	−0.17558
H	+4.71064	−1.95028	−1.74103
H	+2.00719	−2.46378	−0.65911
H	+2.41401	−2.01101	−2.30017
H	+2.79903	−1.76654	+1.43909
H	+1.54933	−0.57608	+1.75682
H	+3.17417	−0.33326	+2.38406
H	+6.42580	−0.76020	+0.35088
H	+5.26172	−1.20328	+1.58611
H	+5.63162	+0.49421	+1.28892
H	+2.50948	+0.30197	−1.75522
O	−7.31832	−4.16393	+0.12607
H	−6.68848	−4.43884	−0.58499
C	−6.93556	−4.79474	+1.34495
H	−6.99177	−5.91570	+1.24889
H	−7.66303	−4.49029	+2.12874
C	−5.52543	−4.39034	+1.78886
H	−5.37832	−3.28380	+1.69291
O	−5.49229	−4.75854	+3.23183
C	−4.21841	−4.47896	+3.88513

O	−3.94496	−3.04846	+3.77540
H	−4.34260	−4.77351	+4.96214
C	−3.11962	−5.28106	+3.19762
O	−1.86952	−5.00155	+3.86460
H	−1.12568	−5.30475	+3.27788
H	−3.35480	−6.37945	+3.29770
C	−3.03899	−4.91553	+1.71862
O	−2.04819	−5.77080	+1.09887
H	−2.19004	−5.73549	+0.10559
H	−2.73145	−3.83751	+1.60849
C	−4.40087	−5.12634	+1.04595
H	−4.62690	−6.22422	+0.97626
O	−4.28027	−4.56301	−0.31384
C	−4.57073	−5.47685	−1.40952
O	−5.74536	−4.98609	−2.12232
C	−5.56850	−3.59813	−2.63413
C	−6.93045	−3.25868	−3.24575
O	−7.03736	−1.87200	−3.57158
H	−6.43607	−1.68648	−4.33343
H	−7.10391	−3.89100	−4.15884
H	−7.72818	−3.49941	−2.51090
H	−5.33312	−2.92368	−1.76573
H	−4.83390	−6.49635	−1.01722
C	−3.38844	−5.52120	−2.37134
O	−2.23377	−5.98210	−1.63484
H	−1.42177	−5.81097	−2.18296
H	−3.61664	−6.25392	−3.19758
C	−3.12475	−4.14522	−2.98277
O	−2.08234	−4.30627	−3.97588
H	−2.08194	−3.49126	−4.56044
H	−2.78034	−3.42910	−2.18490
C	−4.40384	−3.60171	−3.63450
H	−4.66507	−4.21707	−4.53663
O	−4.12394	−2.20970	−4.06237
C	−4.24092	−1.96585	−5.49506
O	−5.33606	−1.02236	−5.71961
C	−5.14063	+0.27057	−5.01640
C	−6.41604	+1.06142	−5.32136
O	−6.54450	+2.20459	−4.47590
H	−5.84622	+2.85904	−4.72421

H	−6.42202	+1.36673	−6.40274
H	−7.29784	+0.40792	−5.14863
H	−5.05436	+0.06598	−3.91823
H	−4.52095	−2.90973	−6.03533
C	−2.95503	−1.35753	−6.04428
O	−1.88093	−2.29502	−5.82153
H	−1.01586	−1.81901	−5.93877
H	−3.07986	−1.19455	−7.15367
C	−2.65014	−0.01861	−5.37559
O	−1.48880	+0.54331	−6.03304
H	−1.42996	+1.51273	−5.78122
H	−2.42951	−0.17696	−4.28273
C	−3.84860	+0.92876	−5.51922
H	−3.96188	+1.24756	−6.58931
O	−3.56830	+2.11735	−4.68187
C	−3.50245	+3.38527	−5.39768
O	−4.58006	+4.24407	−4.90804
C	−4.50607	+4.48083	−3.44337
C	−5.75069	+5.31804	−3.13472
O	−6.01147	+5.38148	−1.73286
H	−5.30687	+5.92850	−1.30654
H	−5.62699	+6.35012	−3.56129
H	−6.63173	+4.85169	−3.62554
H	−4.55424	+3.49080	−2.92217
H	−3.69047	+3.23176	−6.49415
C	−2.16941	+4.07848	−5.13799
O	−1.11244	+3.22686	−5.62782
H	−0.25176	+3.54020	−5.23993
H	−2.15363	+5.05269	−5.70664
C	−1.98396	+4.36699	−3.64955
O	−0.76214	+5.12967	−3.50079
H	−0.76464	+5.54295	−2.58717
H	−1.90113	+3.40080	−3.07851
C	−3.17577	+5.17304	−3.11652
H	−3.16024	+6.21050	−3.54424
O	−3.02721	+5.24880	−1.64567
C	−2.90928	+6.59472	−1.09784
O	−4.05748	+6.83631	−0.22581
C	−4.16417	+5.83843	+0.86932
C	−5.45601	+6.21559	+1.60045

O	−5.89313	+5.17296	+2.47166
H	−5.26252	+5.11245	+3.23093
H	−5.30271	+7.17087	+2.17173
H	−6.25721	+6.39337	+0.85134
H	−4.25134	+4.82023	+0.41167
H	−2.96209	+7.36209	−1.91597
C	−1.63452	+6.72559	−0.27178
O	−0.50574	+6.49764	−1.14158
H	+0.30860	+6.38795	−0.58065
H	−1.57805	+7.77369	+0.14068
C	−1.63212	+5.73117	+0.88810
O	−0.45281	+5.99468	+1.68538
H	−0.57261	+5.54532	+2.57450
H	−1.59432	+4.67862	+0.49007
C	−2.89917	+5.90825	+1.73590
H	−2.85768	+6.88398	+2.28876
O	−2.93361	+4.79490	+2.71057
C	−2.92957	+5.19422	+4.11338
O	−4.17941	+4.74559	+4.72285
C	−4.37724	+3.27666	+4.62086
C	−5.75914	+3.03944	+5.23711
O	−6.24978	+1.73176	+4.94496
H	−5.71644	+1.07485	+5.45674
H	−5.71153	+3.20598	+6.34706
H	−6.47514	+3.77646	+4.81397
H	−4.37190	+2.99330	+3.53725
H	−2.91340	+6.31322	+4.20608
C	−1.76240	+4.54344	+4.84676
O	−0.53797	+4.99558	+4.23077
H	+0.20766	+4.41243	+4.53654
H	−1.78025	+4.87771	+5.92379
C	−1.86576	+3.01987	+4.79618
O	−0.79354	+2.48484	+5.60719
H	−1.00255	+1.52383	+5.80758
H	−1.75556	+2.66166	+3.73414
C	−3.22525	+2.56771	+5.34610
H	−3.27800	+2.76686	+6.44923
O	−3.33449	+1.11100	+5.10658
C	−3.49467	+0.29385	+6.30672
O	−4.79748	−0.36313	+6.24750

H	−3.50616	+0.94146	+7.22421
C	−2.40628	−0.77148	+6.37624
O	−1.12704	−0.10352	+6.42562
H	−0.41310	−0.76969	+6.23594
H	−2.54772	−1.36958	+7.32182
C	−2.47836	−1.71178	+5.17458
O	−1.49620	−2.75502	+5.37858
H	−1.70691	−3.50817	+4.74852
H	−2.23994	−1.14384	+4.23135
C	−3.88388	−2.31663	+5.05930
H	−4.06507	−3.03196	+5.90496
C	−4.95858	−1.22162	+5.04536
H	−4.83066	−0.58137	+4.13494
C	−6.39474	−1.74542	+5.14106
H	−6.46737	−2.50115	+5.96950
H	−7.07232	−0.90109	+5.39284
O	−6.83310	−2.30738	+3.90554
H	−6.34262	−3.15207	+3.75057
O	+5.88694	−5.73350	−1.06492
H	+5.23225	−6.12649	−0.43649
C	+5.45706	−6.00337	−2.39879
H	+5.28744	−7.10479	−2.54519
H	+6.27047	−5.69178	−3.08882
C	+4.18033	−5.23393	−2.75090
H	+4.27858	−4.15393	−2.47130
O	+4.09255	−5.32859	−4.23096
C	+2.95706	−4.58832	−4.77611
O	+3.07857	−3.19365	−4.36300
H	+3.02542	−4.67605	−5.89361
C	+1.66813	−5.19675	−4.23440
O	+0.55792	−4.45541	−4.78382
H	−0.26272	−4.67734	−4.26630
H	+1.60419	−6.26905	−4.57770
C	+1.64065	−5.15699	−2.70776
O	+0.46393	−5.87939	−2.27268
H	+0.55526	−6.05296	−1.28861
H	+1.58212	−4.08991	−2.35512
C	+2.90238	−5.81284	−2.12949
H	+2.86167	−6.92419	−2.28019
O	+2.91743	−5.51487	−0.67991

C	+2.88618	−6.67636	+0.19939
O	+4.12612	−6.69612	+0.97358
C	+4.32880	−5.45413	+1.76333
C	+5.70097	−5.64420	+2.41628
O	+6.20230	−4.42303	+2.95877
H	+5.65549	−4.18259	+3.74680
H	+5.63426	−6.43343	+3.21324
H	+6.42132	−5.99837	+1.64793
H	+4.34187	−4.58349	+1.05783
H	+2.86722	−7.62680	−0.39839
C	+1.70750	−6.57997	+1.16121
O	+0.49280	−6.56401	+0.38235
H	−0.25401	−6.26048	+0.96602
H	+1.70859	−7.48981	+1.82802
C	+1.81360	−5.32599	+2.02778
O	+0.73227	−5.36662	+2.98873
H	+0.94364	−4.71675	+3.72295
H	+1.71860	−4.40573	+1.38554
C	+3.16567	−5.30191	+2.75324
H	+3.20162	−6.11697	+3.52395
O	+3.27770	−3.98833	+3.42648
C	+3.43177	−4.04341	+4.87568
O	+4.73338	−3.47618	+5.22130
C	+4.89545	−2.07504	+4.75537
C	+6.33272	−1.71646	+5.14466
O	+6.77019	−0.51698	+4.50738
H	+6.27736	+0.24416	+4.90144
H	+6.40870	−1.62159	+6.26165
H	+7.00941	−2.53882	+4.82758
H	+4.76844	−2.05328	+3.64212
H	+3.44435	−5.10848	+5.23158
C	+2.34196	−3.22708	+5.56170
O	+1.06408	−3.79585	+5.20605
H	+0.34860	−3.14895	+5.44872
H	+2.48351	−3.30356	+6.67813
C	+2.41350	−1.75774	+5.14952
O	+1.43332	−1.03795	+5.93542
H	+1.63939	−0.05874	+5.86602
H	+2.17264	−1.65473	+4.05503
C	+3.81953	−1.20255	+5.41619

H	+4.00030	−1.13198	+6.52158
O	+3.87870	+0.15386	+4.82436
C	+4.15053	+1.23196	+5.76911
O	+5.43280	+1.84020	+5.42148
C	+5.46290	+2.39221	+4.04249
C	+6.89818	+2.90118	+3.87711
O	+7.20686	+3.18149	+2.51264
H	+6.70515	+3.98736	+2.23602
H	+7.05033	+3.81458	+4.51331
H	+7.60216	+2.11873	+4.23345
H	+5.25051	+1.56091	+3.32148
H	+4.26496	+0.82462	+6.80940
C	+3.06194	+2.29672	+5.69336
O	+1.80441	+1.67375	+6.02873
H	+1.06411	+2.28165	+5.76062
H	+3.29080	+3.10071	+6.45084
C	+3.00353	+2.92581	+4.30235
O	+2.03297	+3.99887	+4.34763
H	+2.18040	+4.58741	+3.54830
H	+2.68338	+2.15500	+3.54558
C	+4.38275	+3.47512	+3.91468
H	+4.63842	+4.36048	+4.55512
O	+4.30007	+3.90279	+2.50000
C	+4.58347	+5.31350	+2.25365
O	+5.78084	+5.40347	+1.42158
C	+5.64428	+4.66528	+0.13955
C	+7.01134	+4.81856	−0.53387
O	+7.17312	+3.90274	−1.61612
H	+6.55049	+4.15608	−2.34149
H	+7.14122	+5.87755	−0.88757
H	+7.80892	+4.61044	+0.21128
H	+5.43491	+3.59017	+0.37255
H	+4.82153	+5.84191	+3.21552
C	+3.42223	+5.96887	+1.51367
O	+2.24572	+5.86522	+2.34606
H	+1.44470	+6.04520	+1.78556
H	+3.66565	+7.05740	+1.34970
C	+3.18417	+5.29566	+0.16382
O	+2.16996	+6.05697	−0.53579
H	+2.16262	+5.75092	−1.49173

H	+2.82128	+4.24410	+0.32666
C	+4.47651	+5.25419	−0.66306
H	+4.73235	+6.28190	−1.03380
O	+4.21728	+4.36505	−1.81816
C	+4.33671	+4.99025	−3.13000
O	+5.43272	+4.34195	−3.84732
C	+5.23253	+2.87755	−4.00412
C	+6.51325	+2.39348	−4.69009
O	+6.63203	+0.97211	−4.64378
H	+5.94294	+0.58075	−5.23532
H	+6.53367	+2.75831	−5.75262
H	+7.39138	+2.82770	−4.16559
H	+5.13207	+2.42344	−2.98541
H	+4.61822	+6.07269	−3.02788
C	+3.04966	+4.80673	−3.92587
O	+1.97547	+5.44465	−3.20236
H	+1.11179	+5.13186	−3.58507
H	+3.17078	+5.31047	−4.92782
C	+2.74653	+3.32553	−4.14668
O	+1.58671	+3.24375	−5.00792
H	+1.53994	+2.31253	−5.37880
H	+2.52641	+2.82646	−3.16146
C	+3.94778	+2.63776	−4.80882
H	+4.07229	+3.01258	−5.85934
O	+3.66216	+1.18559	−4.83930
C	+3.62791	+0.58824	−6.17023
O	+4.70457	−0.39529	−6.26079
H	+3.84069	+1.36341	−6.95471
C	+2.29636	−0.11337	−6.41395
O	+1.24308	+0.86686	−6.30827
H	+0.37191	+0.39029	−6.24184
H	+2.30234	−0.53830	−7.45902
C	+2.07790	−1.25114	−5.41826
O	+0.86017	−1.93266	−5.80058
H	+0.83853	−2.82034	−5.33297
H	+1.97276	−0.83829	−4.37559
C	+3.26538	−2.22172	−5.46327
H	+3.27777	−2.76890	−6.44297
C	+4.59168	−1.47918	−5.25069
H	+4.60680	−1.01728	−4.23045

C +5.84310 −2.33175 −5.48053
H +5.75624 −2.87155 −6.46191
H +6.72978 −1.66437 −5.53807
O +6.05711 −3.25675 −4.41536
H +5.35825 −3.95468 −4.46143
345
* E = 1.850 kcal/mol
C −0.65934 +0.78928 +0.20912
C −1.32144 −0.36158 +0.68642
C −0.63767 −1.52106 +1.02200
C +0.71908 −1.85214 +0.96843
C +1.83069 −1.36512 +0.27804
C +1.82226 −0.66311 −0.93547
C +0.65004 −0.50425 −1.67369
H −0.12301 +0.35634 −0.92013
C +3.06674 +0.04122 −1.41343
C +4.36184 −0.56841 −0.90936
C +4.26976 −0.80145 +0.57978
C +3.16305 −1.78412 +0.85329
C −1.39182 +2.09075 −0.05763
C −2.89430 +2.06449 +0.22302
C −3.47911 +0.70093 −0.08115
C −2.80885 −0.30325 +0.85200
C −4.94339 +0.50242 +0.32113
C −5.07058 −1.02563 +0.44233
C −3.65198 −1.56257 +0.70936
O +5.43382 −1.36323 +1.13038
H +6.17756 −0.78009 +0.99076
C −3.26673 +0.33837 −1.54625
C −5.98487 +1.13588 −0.57321
H +4.03772 +0.14806 +1.07782
H +3.08609 −1.93715 +1.92682
H +3.47495 −2.73847 +0.42009
H +5.19713 +0.08864 −1.14904
H +4.55169 −1.52664 −1.39497
H +3.06925 +0.07980 −2.50182
H +3.01452 +1.08187 −1.07765
H −0.11276 −1.27057 −1.56849
H +0.73117 −0.07031 −2.66302
H +0.96923 −2.66357 +1.64570

H	−1.24416	−2.27535	+1.51055
H	+0.34966	+0.90655	+0.60595
H	−1.22977	+2.37083	−1.10079
H	−0.92504	+2.88430	+0.52560
H	−3.07840	+2.28567	+1.27669
H	−3.38647	+2.84793	−0.35606
H	−5.04370	+0.93367	+1.32122
H	−5.46961	−1.44057	−0.48306
H	−5.76868	−1.30053	+1.22815
H	−3.29055	−2.16845	−0.12239
H	−3.60472	−2.18926	+1.59784
H	−3.76095	−0.59711	−1.80385
H	−2.21087	+0.22457	−1.78593
H	−3.67199	+1.11476	−2.19465
H	−6.98320	+0.95613	−0.18164
H	−5.95099	+0.72211	−1.58022
H	−5.85181	+2.21497	−0.64476
H	−2.99154	+0.08411	+1.86243
O	−6.68428	−4.88394	+0.22658
H	−6.11292	−5.17829	−0.52519
C	−6.23890	−5.52934	+1.41945
H	−6.22852	−6.64552	+1.28608
H	−6.96502	−5.29210	+2.22658
C	−4.84780	−5.04576	+1.83979
H	−4.79631	−3.92775	+1.81514
O	−4.71471	−5.48767	+3.25190
C	−3.44935	−5.07586	+3.85646
O	−3.34709	−3.62310	+3.76232
H	−3.48733	−5.40142	+4.93048
C	−2.30759	−5.73901	+3.09529
O	−1.06461	−5.35138	+3.71788
H	−0.31437	−5.59519	+3.11085
H	−2.42555	−6.85816	+3.16859
C	−2.32850	−5.32620	+1.62525
O	−1.29726	−6.08043	+0.94537
H	−1.45095	−5.99171	−0.04227
H	−2.11663	−4.22355	+1.53481
C	−3.69968	−5.63212	+1.00660
H	−3.82794	−6.74085	+0.88797
O	−3.72534	−4.99078	−0.32716

C	−3.91840	−5.89503	−1.45325
O	−5.17007	−5.53505	−2.11729
C	−5.18713	−4.12579	−2.58730
C	−6.59488	−3.93857	−3.16000
O	−6.89398	−2.56153	−3.38754
H	−6.34492	−2.24386	−4.14593
H	−6.69757	−4.52794	−4.11091
H	−7.33672	−4.33467	−2.43372
H	−5.02535	−3.45482	−1.70457
H	−4.03911	−6.95304	−1.09656
C	−2.77381	−5.75376	−2.45016
O	−1.54684	−6.11123	−1.77988
H	−0.77926	−5.78830	−2.32425
H	−2.94971	−6.46761	−3.30589
C	−2.69942	−4.32929	−2.99799
O	−1.67063	−4.31055	−4.01516
H	−1.79306	−3.48331	−4.56914
H	−2.43347	−3.61186	−2.17187
C	−4.05253	−3.93077	−3.60273
H	−4.25144	−4.53423	−4.52791
O	−3.97045	−2.49716	−3.96433
C	−4.17140	−2.20050	−5.37855
O	−5.37486	−1.38158	−5.51151
C	−5.29618	−0.10867	−4.74979
C	−6.66708	+0.54080	−4.95915
O	−6.88774	+1.61178	−4.04162
H	−6.28965	+2.36045	−4.28535
H	−6.75743	+0.90267	−6.01893
H	−7.45848	−0.22115	−4.79229
H	−5.13558	−0.35209	−3.66817
H	−4.36450	−3.14419	−5.95597
C	−2.98500	−1.42402	−5.93975
O	−1.80406	−2.24111	−5.79835
H	−1.00242	−1.66636	−5.92531
H	−3.17035	−1.22792	−7.03517
C	−2.80856	−0.08917	−5.21834
O	−1.74619	+0.63102	−5.88872
H	−1.79586	+1.59166	−5.60417
H	−2.52691	−0.26784	−4.14337
C	−4.11335	+0.71685	−5.27495

H	−4.31010	+1.05825	−6.32582
O	−3.93549	+1.89994	−4.40262
C	−4.06331	+3.19333	−5.06406
O	−5.21244	+3.89083	−4.49043
C	−5.08988	+4.08758	−3.02307
C	−6.41815	+4.72995	−2.61365
O	−6.60333	+4.69406	−1.19863
H	−5.98188	+5.34312	−0.78622
H	−6.46186	+5.78659	−2.99166
H	−7.25292	+4.16634	−3.08292
H	−4.97108	+3.08351	−2.53997
H	−4.28487	+3.05795	−6.15662
C	−2.81218	+4.03523	−4.83621
O	−1.68856	+3.32558	−5.39833
H	−0.84877	+3.74111	−5.06371
H	−2.93662	+5.01831	−5.37523
C	−2.59305	+4.30850	−3.34849
O	−1.46850	+5.21256	−3.23046
H	−1.48526	+5.61239	−2.31023
H	−2.36487	+3.34564	−2.80939
C	−3.84924	+4.94485	−2.73897
H	−3.98950	+5.98343	−3.14013
O	−3.63878	+5.01043	−1.27528
C	−3.68376	+6.34820	−0.69303
O	−4.81225	+6.40917	+0.23279
C	−4.73519	+5.38244	+1.30409
C	−6.02298	+5.57926	+2.10966
O	−6.28207	+4.47172	+2.97116
H	−5.59008	+4.45098	+3.67751
H	−5.95719	+6.53384	+2.69950
H	−6.87964	+5.67359	+1.40812
H	−4.71936	+4.37109	+0.82311
H	−3.87975	+7.11757	−1.48724
C	−2.39996	+6.63619	+0.07807
O	−1.29468	+6.56896	−0.85022
H	−0.45013	+6.49727	−0.33083
H	−2.46082	+7.67694	+0.50720
C	−2.20808	+5.63216	+1.21244
O	−1.05181	+6.04801	+1.97921
H	−1.04682	+5.52205	+2.83413

H	−2.02776	+4.60884	+0.78296
C	−3.44855	+5.58584	+2.11512
H	−3.51466	+6.52305	+2.72876
O	−3.28299	+4.42080	+3.01362
C	−3.23027	+4.73058	+4.43763
O	−4.38479	+4.10988	+5.08396
C	−4.42792	+2.63569	+4.89807
C	−5.73823	+2.21085	+5.56716
O	−6.09439	+0.87126	+5.22810
H	−5.45335	+0.25721	+5.66397
H	−5.64793	+2.32685	+6.68110
H	−6.55510	+2.88037	+5.22177
H	−4.45385	+2.41842	+3.79962
H	−3.32806	+5.83692	+4.60485
C	−1.95602	+4.16970	+5.05819
O	−0.82518	+4.78257	+4.40230
H	−0.01072	+4.25849	+4.63038
H	−1.93959	+4.44077	+6.15301
C	−1.89931	+2.64902	+4.92113
O	−0.72819	+2.19328	+5.63799
H	−0.82048	+1.20536	+5.78875
H	−1.81781	+2.36316	+3.83513
C	−3.16569	+2.02223	+5.51940
H	−3.17361	+2.16346	+6.63279
O	−3.13073	+0.57503	+5.21100
C	−3.12574	−0.30659	+6.37401
O	−4.35029	−1.10365	+6.35198
H	−3.15677	+0.29439	+7.32247
C	−1.92425	−1.24467	+6.33835
O	−0.72507	−0.44269	+6.34709
H	+0.04788	−1.02419	+6.11131
H	−1.94602	−1.88660	+7.26598
C	−1.95945	−2.15144	+5.10907
O	−0.85858	−3.08330	+5.23069
H	−1.01651	−3.83870	+4.58964
H	−1.83899	−1.53898	+4.17239
C	−3.29424	−2.90697	+5.05622
H	−3.34814	−3.65147	+5.89426
C	−4.48062	−1.93621	+5.12827
H	−4.46939	−1.25948	+4.23610

C	−5.84634	−2.61265	+5.28094
H	−5.80072	−3.37182	+6.10783
H	−6.59962	−1.84675	+5.56548
O	−6.27637	−3.22081	+4.06359
H	−5.70724	−4.00997	+3.88842
O	+6.50957	−4.96078	−1.39615
H	+5.94246	−5.49544	−0.78789
C	+6.06928	−5.17483	−2.73523
H	+6.02791	−6.27351	−2.96865
H	+6.81424	−4.71244	−3.41847
C	+4.69739	−4.54428	−2.99568
H	+4.67579	−3.47794	−2.65148
O	+4.57161	−4.56491	−4.47677
C	+3.33402	−3.96033	−4.96242
O	+3.30006	−2.56693	−4.52955
H	+3.37132	−4.02205	−6.08351
C	+2.14750	−4.72937	−4.39288
O	+0.93748	−4.10993	−4.87714
H	+0.17178	−4.44036	−4.33450
H	+2.19487	−5.79161	−4.77042
C	+2.17789	−4.74446	−2.86562
O	+1.08812	−5.58696	−2.41941
H	+1.25525	−5.83193	−1.46047
H	+2.04265	−3.70193	−2.46506
C	+3.51878	−5.31069	−2.38004
H	+3.58859	−6.40179	−2.63448
O	+3.56085	−5.14785	−0.91059
C	+3.68985	−6.38605	−0.14807
O	+4.95698	−6.35076	+0.57573
C	+5.08334	−5.17361	+1.47629
C	+6.47014	−5.34021	+2.10678
O	+6.95251	−4.12506	+2.67510
H	+6.33517	−3.84244	+3.39439
H	+6.43486	−6.15604	+2.88222
H	+7.18987	−5.65677	+1.32096
H	+5.03702	−4.24381	+0.85486
H	+3.74002	−7.27123	−0.83774
C	+2.55287	−6.51030	+0.86014
O	+1.30842	−6.56923	+0.12888
H	+0.56158	−6.38800	+0.75970

H	+2.68656	−7.46657	+1.44241
C	+2.55862	−5.32498	+1.82094
O	+1.54650	−5.56112	+2.83140
H	+1.70224	−4.90520	+3.57467
H	+2.31399	−4.38277	+1.25757
C	+3.93122	−5.17434	+2.49131
H	+4.07696	−5.98838	+3.25083
O	+3.91805	−3.86313	+3.17454
C	+4.15909	−3.89272	+4.60901
O	+5.40016	−3.17121	+4.87792
C	+5.36546	−1.76685	+4.38623
C	+6.77655	−1.24298	+4.66579
O	+7.01024	+0.01328	+4.02522
H	+6.46726	+0.69789	+4.48753
H	+6.93734	−1.15397	+5.77416
H	+7.51835	−1.97057	+4.27261
H	+5.15817	−1.77251	+3.28241
H	+4.31740	−4.94704	+4.96357
C	+3.01323	−3.20851	+5.34752
O	+1.79310	−3.91551	+5.03022
H	+1.02218	−3.34255	+5.28610
H	+3.20328	−3.27853	+6.45710
C	+2.89983	−1.73586	+4.95400
O	+1.89756	−1.13573	+5.81005
H	+1.99535	−0.13954	+5.75071
H	+2.57933	−1.65000	+3.87801
C	+4.24990	−1.02957	+5.14064
H	+4.49930	−0.96632	+6.23357
O	+4.11047	+0.34191	+4.59609
C	+4.31870	+1.41387	+5.56094
O	+5.50464	+2.17296	+5.16270
C	+5.38591	+2.77307	+3.80972
C	+6.74636	+3.43683	+3.57732
O	+6.91961	+3.82756	+2.21533
H	+6.32177	+4.59321	+2.03208
H	+6.85499	+4.32275	+4.25906
H	+7.55046	+2.71308	+3.83101
H	+5.21255	+1.95003	+3.06994
H	+4.54186	+0.99243	+6.57778
C	+3.12054	+2.35718	+5.59096

O	+1.95515	+1.59795	+5.97267
H	+1.14551	+2.13055	+5.74919
H	+3.31297	+3.15482	+6.36574
C	+2.90751	+3.02673	+4.23468
O	+1.83259	+3.98497	+4.38502
H	+1.85756	+4.60525	+3.59769
H	+2.62523	+2.25602	+3.46437
C	+4.19346	+3.73877	+3.79577
H	+4.39467	+4.61715	+4.46458
O	+3.98107	+4.21779	+2.41123
C	+4.06307	+5.66272	+2.23181
O	+5.19831	+5.95385	+1.35779
C	+5.09673	+5.27733	+0.03933
C	+6.39433	+5.65760	−0.68040
O	+6.63312	+4.82669	−1.81585
H	+5.95969	+5.03685	−2.50878
H	+6.35664	+6.73950	−0.98199
H	+7.24745	+5.53109	+0.02035
H	+5.04940	+4.17216	+0.21262
H	+4.27445	+6.17226	+3.20988
C	+2.79390	+6.19225	+1.57360
O	+1.68108	+5.91807	+2.45031
H	+0.83674	+6.07725	+1.94874
H	+2.89470	+7.30756	+1.44062
C	+2.57826	+5.54221	+0.20835
O	+1.42556	+6.17250	−0.40027
H	+1.42675	+5.94244	−1.37709
H	+2.38538	+4.44037	+0.33610
C	+3.81872	+5.74050	−0.67326
H	+3.90657	+6.81717	−0.97734
O	+3.63153	+4.90412	−1.87911
C	+3.62105	+5.62375	−3.14686
O	+4.75193	+5.15743	−3.94647
C	+4.72670	+3.68848	−4.16726
C	+6.01619	+3.39803	−4.94079
O	+6.32794	+2.00532	−4.94140
H	+5.66240	+1.53387	−5.50102
H	+5.92260	+3.78384	−5.99197
H	+6.85852	+3.93666	−4.45582
H	+4.74193	+3.18188	−3.16891

H	+3.77637	+6.72341	−2.98127
C	+2.33136	+5.33207	−3.90504
O	+1.22563	+5.80903	−3.10860
H	+0.38367	+5.41917	−3.46746
H	+2.35049	+5.89013	−4.88467
C	+2.19486	+3.83588	−4.18213
O	+1.01843	+3.64897	−5.00387
H	+1.06478	+2.73203	−5.40960
H	+2.07442	+3.27671	−3.21220
C	+3.43923	+3.31759	−4.91586
H	+3.46472	+3.72523	−5.96117
O	+3.33066	+1.84260	−4.96834
C	+3.31450	+1.26658	−6.30890
O	+4.49483	+0.41910	−6.45847
H	+3.40092	+2.07308	−7.08571
C	+2.06554	+0.41541	−6.50733
O	+0.91057	+1.26621	−6.34347
H	+0.10575	+0.69060	−6.23982
H	+2.07399	+0.00220	−7.55677
C	+2.02666	−0.74577	−5.51515
O	+0.87877	−1.56067	−5.84820
H	+0.98684	−2.45050	−5.39579
H	+1.92226	−0.35059	−4.46453
C	+3.31766	−1.56888	−5.62133
H	+3.35392	−2.09986	−6.60951
C	+4.55268	−0.67358	−5.45296
H	+4.54907	−0.21437	−4.43111
C	+5.88734	−1.37130	−5.73073
H	+5.82683	−1.92789	−6.70474
H	+6.68434	−0.60299	−5.82841
O	+6.25287	−2.25226	−4.66945
H	+5.63876	−3.02744	−4.68088

345

* E = 1.985 kcal/mol

C	+1.04333	+0.20057	−1.07923
C	+1.14696	−0.94338	−0.26816
C	+0.04694	−1.61880	+0.24156
C	−1.32051	−1.36797	+0.10840
C	−2.08999	−0.25446	−0.24894
C	−1.72959	+1.09022	−0.09995

C	−0.56025	+1.46748	+0.56944
H	+0.46648	+1.11854	−0.25578
C	−2.54835	+2.17591	−0.74873
C	−4.00685	+1.81349	−0.94371
C	−4.10812	+0.45861	−1.60106
C	−3.49692	−0.58667	−0.70130
C	+2.31290	+0.67952	−1.72369
C	+3.47290	+0.85552	−0.74309
C	+3.46689	−0.20665	+0.35909
C	+2.55514	−1.38209	−0.02691
C	+4.78840	−0.95726	+0.55415
C	+4.37054	−2.21271	+1.32993
C	+2.88403	−2.44895	+1.00185
O	−5.43486	+0.05705	−1.82826
H	−5.84116	+0.60423	−2.50327
C	+2.99302	+0.41788	+1.66320
C	+5.92033	−0.18428	+1.19042
H	−3.55247	+0.48052	−2.54771
H	−3.51490	−1.54435	−1.21755
H	−4.15333	−0.68597	+0.16639
H	−4.50093	+2.57284	−1.54989
H	−4.52605	+1.77130	+0.01349
H	−2.46159	+3.09322	−0.16859
H	−2.10747	+2.39823	−1.72738
H	−0.20186	+0.79040	+1.33934
H	−0.43394	+2.51960	+0.79625
H	−1.92255	−2.25605	+0.28125
H	+0.27340	−2.56255	+0.72345
H	+0.14736	+0.25247	−1.69752
H	+2.13923	+1.60307	−2.27337
H	+2.60087	−0.06773	−2.46919
H	+4.40992	+0.82296	−1.30001
H	+3.41942	+1.84765	−0.29330
H	+5.10721	−1.26314	−0.44588
H	+4.50649	−2.04375	+2.39851
H	+4.99077	−3.06639	+1.07030
H	+2.26211	−2.32649	+1.88917
H	+2.69137	−3.45084	+0.62482
H	+2.95258	−0.30274	+2.47834
H	+1.99931	+0.84590	+1.54652

H	+3.66235	+1.22113	+1.96622
H	+6.82740	−0.78343	+1.21114
H	+5.69140	+0.09059	+2.21997
H	+6.14212	+0.72774	+0.63692
H	+2.93966	−1.73546	−0.99306
O	−7.54739	−3.00168	−2.21529
H	−6.86036	−3.12509	−2.91545
C	−7.43538	−4.06388	−1.27103
H	−7.58328	−5.06009	−1.77382
H	−8.24825	−3.94135	−0.52315
C	−6.08535	−4.05417	−0.54655
H	−5.83792	−3.02842	−0.17400
O	−6.30635	−4.94211	+0.62602
C	−5.13372	−5.05888	+1.48873
O	−4.76266	−3.72328	+1.94250
H	−5.43958	−5.70138	+2.35779
C	−3.99824	−5.68400	+0.68661
O	−2.84996	−5.81696	+1.55134
H	−2.05017	−5.99444	+0.98732
H	−4.32101	−6.70773	+0.34046
C	−3.66262	−4.82499	−0.52966
O	−2.66120	−5.52852	−1.30388
H	−2.62690	−5.11027	−2.21552
H	−3.25398	−3.82969	−0.19533
C	−4.91776	−4.59874	−1.38213
H	−5.21289	−5.55543	−1.89012
O	−4.55709	−3.59310	−2.40504
C	−4.71574	−4.01795	−3.78946
O	−5.72491	−3.16425	−4.41371
C	−5.36354	−1.72521	−4.32031
C	−6.58500	−0.97658	−4.85440
O	−6.48035	+0.42864	−4.58583
H	−5.82996	+0.80844	−5.22719
H	−6.70141	−1.15288	−5.95568
H	−7.49736	−1.35542	−4.34711
H	−5.19844	−1.46551	−3.24020
H	−5.10644	−5.06970	−3.84036
C	−3.40071	−3.85921	−4.54678
O	−2.40794	−4.68784	−3.90418
H	−1.50863	−4.39878	−4.21503

H	−3.54992	−4.21540	−5.60673
C	−2.94873	−2.39927	−4.57257
O	−1.78485	−2.31927	−5.43025
H	−1.64094	−1.35643	−5.66722
H	−2.67986	−2.06140	−3.53277
C	−4.07234	−1.50965	−5.12049
H	−4.24408	−1.73102	−6.20745
O	−3.63104	−0.10175	−4.97656
C	−3.51699	+0.64136	−6.22489
O	−4.50541	+1.72105	−6.21183
C	−4.32186	+2.66598	−5.08149
C	−5.47969	+3.65817	−5.22974
O	−5.67177	+4.42508	−4.04175
H	−4.92264	+5.06538	−3.95718
H	−5.29240	+4.33062	−6.10942
H	−6.41667	+3.09406	−5.42837
H	−4.41124	+2.09354	−4.12153
H	−3.77716	−0.01280	−7.09973
C	−2.12576	+1.25012	−6.36651
O	−1.16231	+0.17682	−6.37845
H	−0.25117	+0.56683	−6.28913
H	−2.07504	+1.80423	−7.34783
C	−1.83321	+2.22684	−5.22881
O	−0.54869	+2.83864	−5.49554
H	−0.46853	+3.65605	−4.91915
H	−1.79681	+1.67572	−4.24707
C	−2.92773	+3.29976	−5.16728
H	−2.85703	+3.97458	−6.06101
O	−2.69844	+4.08857	−3.93726
C	−2.46224	+5.51304	−4.14351
O	−3.54598	+6.25499	−3.50376
C	−3.67292	+5.95028	−2.05513
C	−4.88484	+6.77047	−1.60225
O	−5.36291	+6.34756	−0.32641
H	−4.69442	+6.59445	+0.35933
H	−4.61678	+7.86173	−1.58384
H	−5.70787	+6.63672	−2.33697
H	−3.87099	+4.85349	−1.94074
H	−2.50146	+5.76740	−5.23649
C	−1.14018	+5.92263	−3.50551

O	−0.08362	+5.18592	−4.15789
H	+0.74449	+5.26547	−3.61208
H	−0.98108	+7.02625	−3.67259
C	−1.15303	+5.63733	−2.00494
O	+0.07557	+6.16301	−1.44915
H	−0.02707	+6.20040	−0.45129
H	−1.20621	+4.52635	−1.82809
C	−2.36235	+6.31441	−1.34491
H	−2.21963	+7.42753	−1.33451
O	−2.43445	+5.81645	+0.04652
C	−2.31535	+6.83774	+1.08164
O	−3.56544	+6.87573	+1.83607
C	−3.90552	+5.56955	+2.45875
C	−5.26156	+5.81471	+3.12739
O	−5.89603	+4.59310	+3.50162
H	−5.38600	+4.18852	+4.24597
H	−5.12595	+6.48160	+4.02180
H	−5.92836	+6.34103	+2.41086
H	−3.99514	+4.80390	+1.64676
H	−2.18939	+7.85426	+0.62114
C	−1.17463	+6.49492	+2.03221
O	+0.05106	+6.46455	+1.27027
H	+0.75404	+6.02212	+1.81831
H	−1.10238	+7.30174	+2.81698
C	−1.42139	+5.15215	+2.71773
O	−0.35877	+4.95393	+3.67967
H	−0.64677	+4.23540	+4.31888
H	−1.40565	+4.32209	+1.95572
C	−2.78276	+5.17011	+3.42596
H	−2.75075	+5.87615	+4.29800
O	−3.04252	+3.79906	+3.91601
C	−3.21114	+3.67492	+5.35981
O	−4.56829	+3.20446	+5.62586
C	−4.86601	+1.89746	+4.98344
C	−6.33487	+1.63627	+5.33047
O	−6.88930	+0.58847	+4.53692
H	−6.47086	−0.26596	+4.80623
H	−6.42678	+1.39624	+6.42468
H	−6.92218	+2.55956	+5.13633
H	−4.73222	+2.00852	+3.87762

H	−3.11956	+4.67877	+5.85539
C	−2.21938	+2.66653	+5.92833
O	−0.88469	+3.14495	+5.65739
H	−0.24349	+2.40059	+5.81448
H	−2.36968	+2.60406	+7.04453
C	−2.43304	+1.28221	+5.31868
O	−1.53449	+0.36893	+5.99274
H	−1.84708	−0.56719	+5.80960
H	−2.19795	+1.30534	+4.21846
C	−3.88932	+0.84229	+5.52049
H	−4.08395	+0.64960	+6.60908
O	−4.08072	−0.41200	+4.76005
C	−4.47916	−1.56829	+5.55485
O	−5.80097	−2.00103	+5.10785
H	−4.58592	−1.28600	+6.63680
C	−3.49450	−2.71530	+5.35942
O	−2.19526	−2.27707	+5.81037
H	−1.51160	−2.91558	+5.47173
H	−3.82918	−3.58887	+5.98975
C	−3.44234	−3.14746	+3.89547
O	−2.57979	−4.30776	+3.81566
H	−2.73653	−4.75064	+2.92883
H	−3.02244	−2.31679	+3.26329
C	−4.85170	−3.50221	+3.40274
H	−5.20522	−4.44462	+3.89967
C	−5.84029	−2.35786	+3.66543
H	−5.54348	−1.45773	+3.06987
C	−7.30700	−2.72041	+3.41282
H	−7.54607	−3.69425	+3.92134
H	−7.95559	−1.93722	+3.86116
O	−7.60599	−2.79292	+2.02028
H	−7.13017	−3.56842	+1.63392
O	+5.50738	−5.45004	−2.20567
H	+4.75448	−6.00587	−1.88694
C	+5.31265	−5.17939	−3.59324
H	+5.13853	−6.13269	−4.16166
H	+6.24319	−4.71584	−3.98572
C	+4.14093	−4.22118	−3.82659
H	+4.24072	−3.30710	−3.18741
O	+4.28145	−3.81725	−5.25023

C	+3.29236	−2.82590	−5.66859
O	+3.45772	−1.63244	−4.84588
H	+3.51476	−2.59350	−6.74495
C	+1.89524	−3.41065	−5.49458
O	+0.93725	−2.40132	−5.87759
H	+0.03568	−2.69031	−5.56938
H	+1.79168	−4.29860	−6.18310
C	+1.65108	−3.87042	−4.05716
O	+0.36149	−4.52666	−4.02841
H	+0.30606	−5.06796	−3.18540
H	+1.64389	−2.98392	−3.36353
C	+2.75393	−4.84676	−3.62622
H	+2.67083	−5.80172	−4.20996
O	+2.55716	−5.13637	−2.18727
C	+2.31662	−6.53896	−1.86153
O	+3.41379	−7.01415	−1.02061
C	+3.55683	−6.24388	+0.24136
C	+4.77989	−6.86290	+0.92421
O	+5.25696	−6.04751	+1.99426
H	+4.58836	−6.05892	+2.72250
H	+4.52510	−7.89289	+1.29447
H	+5.59907	−6.96358	+0.18013
H	+3.75074	−5.17222	−0.02013
H	+2.33612	−7.16753	−2.79212
C	+1.00804	−6.70023	−1.09556
O	−0.06677	−6.23006	−1.93538
H	−0.87844	−6.10683	−1.37303
H	+0.85448	−7.79620	−0.87658
C	+1.03980	−5.92917	+0.22249
O	−0.18017	−6.23814	+0.93769
H	−0.07044	−5.92615	+1.88518
H	+1.09078	−4.82266	+0.01891
C	+2.25926	−6.34913	+1.05513
H	+2.12149	−7.39369	+1.44164
O	+2.34951	−5.41207	+2.19765
C	+2.22566	−6.01197	+3.52131
O	+3.48042	−5.79740	+4.24015
C	+3.83851	−4.35872	+4.34235
C	+5.20180	−4.35237	+5.03941
O	+5.85619	−3.09074	+4.90247

H	+5.35632	−2.41819	+5.42793
H	+5.07563	−4.61735	+6.12428
H	+5.85085	−5.12562	+4.57510
H	+3.92373	−3.94553	+3.30598
H	+2.08729	−7.12353	+3.44108
C	+1.09820	−5.34767	+4.30399
O	−0.13780	−5.57657	+3.59462
H	−0.83017	−4.95990	+3.95600
H	+1.03498	−5.82781	+5.32262
C	+1.35615	−3.85098	+4.47125
O	+0.31871	−3.32111	+5.33047
H	+0.61094	−2.41364	+5.64268
H	+1.31561	−3.34127	+3.46778
C	+2.73385	−3.61370	+5.10456
H	+2.72073	−3.92804	+6.18187
O	+3.00335	−2.15876	+5.02322
C	+3.21674	−1.48969	+6.30138
O	+4.57794	−0.95715	+6.31854
C	+4.83553	+0.00119	+5.21343
C	+6.32043	+0.34738	+5.35221
O	+6.81173	+1.02881	+4.19773
H	+6.41879	+1.93605	+4.17948
H	+6.48010	+0.96774	+6.27485
H	+6.90280	−0.59160	+5.46935
H	+4.63936	−0.51867	+4.23969
H	+3.15332	−2.22629	+7.14691
C	+2.23426	−0.33657	+6.47975
O	+0.89660	−0.87734	+6.43328
H	+0.25453	−0.12858	+6.30453
H	+2.40981	+0.12962	+7.49199
C	+2.42231	+0.73134	+5.40261
O	+1.55366	+1.84045	+5.73559
H	+1.84306	+2.63353	+5.19384
H	+2.13741	+0.31420	+4.39677
C	+3.88480	+1.19696	+5.36338
H	+4.13025	+1.77746	+6.29195
O	+4.03704	+2.07841	+4.18262
C	+4.45145	+3.44769	+4.46642
O	+5.75946	+3.67154	+3.85248
C	+5.75441	+3.46635	+2.38057

C	+7.21542	+3.66405	+1.96686
O	+7.45383	+3.19480	+0.63993
H	+7.00576	+3.80776	+0.00669
H	+7.49233	+4.74889	+2.05867
H	+7.86872	+3.08748	+2.65644
H	+5.42369	+2.41689	+2.17111
H	+4.58985	+3.59591	+5.57113
C	+3.45657	+4.44402	+3.87988
O	+2.16917	+4.19855	+4.48360
H	+1.47199	+4.66433	+3.94821
H	+3.79642	+5.48693	+4.14388
C	+3.37357	+4.32015	+2.35877
O	+2.51427	+5.38323	+1.88140
H	+2.65412	+5.47354	+0.89222
H	+2.93511	+3.32224	+2.07708
C	+4.77083	+4.45397	+1.73866
H	+5.14301	+5.50665	+1.85159
O	+4.64425	+4.13268	+0.29742
C	+5.02374	+5.20497	−0.61748
O	+6.18042	+4.76404	−1.39343
C	+5.92290	+3.52494	−2.17205
C	+7.26719	+3.21916	−2.83996
O	+7.31381	+1.88823	−3.35160
H	+6.70071	+1.82595	−4.12526
H	+7.46126	+3.96530	−3.65758
H	+8.07736	+3.32414	−2.08660
H	+5.64083	+2.71024	−1.45695
H	+5.35345	+6.11273	−0.04424
C	+3.88145	+5.52374	−1.57663
O	+2.74697	+5.94927	−0.79210
H	+1.93743	+5.94095	−1.37021
H	+4.20309	+6.36798	−2.25177
C	+3.52418	+4.30799	−2.42971
O	+2.53175	+4.72232	−3.40012
H	+2.47949	+4.01384	−4.10864
H	+3.09780	+3.49835	−1.77451
C	+4.76805	+3.77377	−3.15240
H	+5.08375	+4.49104	−3.95575
O	+4.38310	+2.48446	−3.77133
C	+4.53144	+2.41243	−5.22116

O	+5.54114	+1.40285	−5.52970
H	+4.91732	+3.38612	−5.62677
C	+3.21454	+2.00611	−5.87554
O	+2.22526	+3.00330	−5.53366
H	+1.32319	+2.63117	−5.72232
H	+3.35643	+1.99280	−6.99384
C	+2.76218	+0.62340	−5.40825
O	+1.61313	+0.24184	−6.20424
H	+1.43124	−0.72872	−6.02569
H	+2.46601	+0.67372	−4.32573
C	+3.88246	−0.41419	−5.57489
H	+4.02071	−0.66014	−6.66091
C	+5.20382	+0.07127	−4.96441
H	+5.09024	+0.17789	−3.85535
C	+6.41399	−0.80343	−5.30490
H	+6.43358	−1.00646	−6.41016
H	+7.34442	−0.25458	−5.04489
O	+6.40542	−2.02578	−4.56868
H	+5.65092	−2.58008	−4.88763

345

* E = 2.402 kcal/mol

C	+0.00431	−1.03322	+0.66559
C	+0.67470	+0.15506	+0.99594
C	+0.02941	+1.37195	+1.17496
C	−1.31972	+1.71132	+1.05304
C	−2.40385	+1.13461	+0.38501
C	−2.37472	+0.30118	−0.74022
C	−1.19911	−0.00310	−1.43487
H	−0.48162	−0.78677	−0.59863
C	−3.69145	−0.28900	−1.14610
C	−4.75735	+0.78796	−1.36880
C	−4.59600	+1.96790	−0.41315
C	−3.79005	+1.55402	+0.80871
C	+0.79184	−2.30904	+0.75868
C	+2.14859	−2.25983	+0.04736
C	+2.77563	−0.86343	+0.06163
C	+2.15069	−0.00800	+1.17409
C	+4.24847	−0.80879	+0.48407
C	+4.45393	+0.65905	+0.88264
C	+3.06339	+1.20340	+1.25820

O	−3.96460	+3.06428	−1.04139
H	−3.04480	+2.83259	−1.18080
C	+2.58091	−0.20693	−1.29897
C	+5.25372	−1.32304	−0.52043
H	−5.56701	+2.34593	−0.11260
H	−4.29744	+0.72933	+1.31544
H	−3.73479	+2.38218	+1.51258
H	−5.74231	+0.34194	−1.25255
H	−4.70085	+1.17804	−2.38301
H	−3.57572	−0.90140	−2.03779
H	−4.03520	−0.95662	−0.35167
H	−0.41905	+0.75368	−1.41112
H	−1.30011	−0.52027	−2.38169
H	−1.58712	+2.60039	+1.61478
H	+0.64714	+2.16256	+1.58357
H	−1.03005	−1.09450	+1.00022
H	+0.19863	−3.14081	+0.38311
H	+0.95630	−2.51516	+1.82150
H	+2.81833	−2.98108	+0.51761
H	+2.02556	−2.58954	−0.98455
H	+4.32467	−1.42149	+1.38657
H	+4.87175	+1.21870	+0.04534
H	+5.16542	+0.75043	+1.69937
H	+2.73912	+1.96426	+0.54793
H	+3.04703	+1.66661	+2.24287
H	+3.00437	+0.79507	−1.34549
H	+1.52465	−0.13662	−1.54683
H	+3.05775	−0.80478	−2.07391
H	+6.25594	−1.31948	−0.09966
H	+5.27355	−0.70167	−1.41484
H	+5.02686	−2.34474	−0.82537
H	+2.29116	−0.59075	+2.09495
O	−7.15953	−3.77250	−1.63246
H	−6.52404	−4.09516	−2.31806
C	−7.06209	−4.61893	−0.48746
H	−7.18559	−5.69685	−0.77979
H	−7.89319	−4.35749	+0.20225
C	−5.72935	−4.43155	+0.24369
H	−5.51790	−3.34420	+0.40800
O	−5.94227	−5.07209	+1.56693

C	−4.78790	−4.92990	+2.45234
O	−4.50779	−3.50773	+2.61141
H	−5.07951	−5.38901	+3.43476
C	−3.59764	−5.64135	+1.81850
O	−2.46553	−5.50951	+2.70391
H	−1.64777	−5.79148	+2.21238
H	−3.85100	−6.73408	+1.70222
C	−3.27938	−5.05248	+0.44531
O	−2.21999	−5.84926	−0.13697
H	−2.18891	−5.65234	−1.12045
H	−2.93521	−3.98620	+0.55684
C	−4.52774	−5.09179	−0.44642
H	−4.76932	−6.15243	−0.72236
O	−4.21219	−4.32651	−1.67268
C	−4.31127	−5.07661	−2.91928
O	−5.35371	−4.46509	−3.74043
C	−5.09234	−3.03108	−4.02755
C	−6.32375	−2.57187	−4.81376
O	−6.39159	−1.14932	−4.90333
H	−5.66958	−0.83775	−5.50300
H	−6.31204	−3.03341	−5.83798
H	−7.23895	−2.92634	−4.29254
H	−5.01657	−2.48319	−3.05339
H	−4.63855	−6.13244	−2.72150
C	−2.99006	−5.01891	−3.67707
O	−1.97252	−5.62685	−2.85320
H	−1.08016	−5.39482	−3.22692
H	−3.09409	−5.60909	−4.63244
C	−2.62059	−3.57652	−4.01943
O	−1.42446	−3.61706	−4.83231
H	−1.32181	−2.72528	−5.28128
H	−2.42068	−2.99528	−3.07530
C	−3.76711	−2.91007	−4.79192
H	−3.86153	−3.37105	−5.81076
O	−3.42896	−1.47624	−4.93296
C	−3.30525	−1.00040	−6.30759
O	−4.34321	−0.00122	−6.54576
C	−4.25176	+1.16918	−5.63408
C	−5.44916	+2.04123	−6.02496
O	−5.71599	+3.04409	−5.04731

H	−4.98658	+3.71121	−5.07146
H	−5.26380	+2.50619	−7.03125
H	−6.35157	+1.39794	−6.11102
H	−4.35225	+0.80615	−4.57864
H	−3.49830	−1.83686	−7.03190
C	−1.93998	−0.36026	−6.53004
O	−0.92819	−1.36030	−6.28633
H	−0.04527	−0.90770	−6.21197
H	−1.87246	−0.02091	−7.60362
C	−1.74223	+0.84607	−5.61391
O	−0.47468	+1.45083	−5.96287
H	−0.45758	+2.38195	−5.58707
H	−1.72281	+0.51361	−4.53765
C	−2.88728	+1.84647	−5.82020
H	−2.81586	+2.30319	−6.84308
O	−2.74104	+2.90645	−4.79959
C	−2.55097	+4.25585	−5.32651
O	−3.68651	+5.07622	−4.92154
C	−3.86874	+5.16733	−3.44577
C	−5.08793	+6.08284	−3.28862
O	−5.69034	+5.96220	−2.00256
H	−5.02911	+6.22666	−1.31606
H	−4.78609	+7.14953	−3.48870
H	−5.85026	+5.80380	−4.04828
H	−4.08282	+4.14455	−3.04234
H	−2.55270	+4.24282	−6.44982
C	−1.27167	+4.86602	−4.76524
O	−0.15774	+4.05224	−5.19205
H	+0.63128	+4.27562	−4.62915
H	−1.15135	+5.90676	−5.18238
C	−1.34222	+4.93647	−3.24325
O	−0.15180	+5.60940	−2.76565
H	−0.32491	+5.90609	−1.82245
H	−1.38577	+3.89516	−2.81587
C	−2.58649	+5.71701	−2.80368
H	−2.45932	+6.80664	−3.04462
O	−2.67358	+5.54506	−1.34004
C	−2.71568	+6.76832	−0.55497
O	−4.00383	+6.82836	+0.13022
C	−4.25130	+5.62386	+0.96945

C	−5.68057	+5.81738	+1.48221
O	−6.17244	+4.64622	+2.13641
H	−5.69256	+4.54883	+2.99462
H	−5.71721	+6.69934	+2.17730
H	−6.34902	+6.03525	+0.62212
H	−4.17372	+4.71230	+0.31662
H	−2.65948	+7.67226	−1.21979
C	−1.60086	+6.74972	+0.48579
O	−0.34085	+6.64915	−0.21630
H	+0.36401	+6.40113	+0.43945
H	−1.62503	+7.71524	+1.06749
C	−1.77097	+5.57667	+1.45124
O	−0.76703	+5.71712	+2.48621
H	−1.01649	+5.10847	+3.24308
H	−1.61414	+4.60709	+0.90098
C	−3.17791	+5.58656	+2.06630
H	−3.29081	+6.46871	+2.75133
O	−3.32815	+4.33908	+2.85530
C	−3.54438	+4.53405	+4.28534
O	−4.87907	+4.04496	+4.63036
C	−5.07562	+2.60414	+4.32804
C	−6.53152	+2.33363	+4.71908
O	−6.99664	+1.08939	+4.19598
H	−6.52835	+0.35572	+4.66523
H	−6.63348	+2.35537	+5.83793
H	−7.17393	+3.13992	+4.30446
H	−4.92815	+2.45006	+3.22892
H	−3.53579	+5.62818	+4.53845
C	−2.51436	+3.75745	+5.09769
O	−1.20247	+4.24855	+4.75364
H	−0.52520	+3.60517	+5.09555
H	−2.70509	+3.94810	+6.19336
C	−2.61652	+2.25737	+4.82969
O	−1.68643	+1.59231	+5.71837
H	−1.92130	+0.61714	+5.73817
H	−2.34525	+2.03721	+3.75944
C	−4.04590	+1.77324	+5.10539
H	−4.25998	+1.81409	+6.20639
O	−4.12944	+0.36896	+4.64318
C	−4.46133	−0.60916	+5.67180

O	−5.73973	−1.22614	+5.32089
H	−4.61211	−0.10534	+6.66403
C	−3.39610	−1.69810	+5.73966
O	−2.13954	−1.08232	+6.09174
H	−1.40704	−1.72251	+5.88491
H	−3.68548	−2.43510	+6.54323
C	−3.28174	−2.43380	+4.40609
O	−2.34474	−3.52292	+4.58573
H	−2.45731	−4.15857	+3.81814
H	−2.90189	−1.73236	+3.61202
C	−4.65211	−2.98236	+3.98757
H	−4.95752	−3.81709	+4.67271
C	−5.71689	−1.87684	+3.98569
H	−5.45592	−1.10274	+3.21960
C	−7.15081	−2.37635	+3.78667
H	−7.35152	−3.23494	+4.48304
H	−7.85803	−1.55881	+4.04378
O	−7.39488	−2.75889	+2.43350
H	−6.88156	−3.58180	+2.24178
O	+5.95305	−5.75288	−0.96842
H	+5.21879	−6.24500	−0.52507
C	+5.74330	−5.79671	−2.37907
H	+5.59956	−6.85595	−2.72560
H	+6.65628	−5.39976	−2.87269
C	+4.53970	−4.94861	−2.80174
H	+4.59363	−3.92737	−2.34356
O	+4.69121	−4.81280	−4.27349
C	+3.66362	−3.96781	−4.87827
O	+3.72983	−2.64755	−4.26148
H	+3.90792	−3.90468	−5.97262
C	+2.29868	−4.60327	−4.63650
O	+1.29834	−3.75167	−5.23382
H	+0.40476	−4.01569	−4.88555
H	+2.27612	−5.61194	−5.14096
C	+2.03438	−4.78630	−3.14298
O	+0.78544	−5.50484	−3.00101
H	+0.73812	−5.86403	−2.06529
H	+1.95222	−3.77972	−2.64348
C	+3.17546	−5.58148	−2.49481
H	+3.15001	−6.64699	−2.84627

O	+2.95170	−5.54348	−1.03206
C	+2.79765	−6.84211	−0.38563
O	+3.89946	−7.01792	+0.55847
C	+3.96922	−5.92733	+1.56490
C	+5.22166	−6.24689	+2.38614
O	+5.62584	−5.13601	+3.18562
H	+4.94655	−4.98953	+3.88931
H	+5.03197	−7.14950	+3.02840
H	+6.05663	−6.49217	+1.69533
H	+4.08725	−4.95403	+1.02290
H	+2.88428	−7.67163	−1.13759
C	+1.48177	−6.89933	+0.38309
O	+0.40236	−6.71356	−0.55992
H	−0.42130	−6.48348	−0.05344
H	+1.38472	−7.91484	+0.86294
C	+1.43744	−5.82101	+1.46339
O	+0.22805	−6.01811	+2.23556
H	+0.30272	−5.46276	+3.06792
H	+1.41585	−4.80784	+0.97727
C	+2.66741	−5.90815	+2.37705
H	+2.59971	−6.81574	+3.03333
O	+2.66343	−4.69042	+3.22084
C	+2.57303	−4.92537	+4.65743
O	+3.80306	−4.44168	+5.28108
C	+4.04463	−2.99367	+5.04738
C	+5.40374	−2.73102	+5.70247
O	+5.93364	−1.45979	+5.32928
H	+5.38775	−0.75561	+5.75797
H	+5.30483	−2.80646	+6.81904
H	+6.12174	−3.51208	+5.37204
H	+4.09467	−2.81672	+3.94269
H	+2.51928	−6.02623	+4.87369
C	+1.38785	−4.17125	+5.24986
O	+0.18484	−4.64501	+4.60842
H	−0.55422	−4.01430	+4.82396
H	+1.33272	−4.39945	+6.35327
C	+1.54037	−2.66226	+5.06285
O	+0.44113	−2.02639	+5.75588
H	+0.67221	−1.05845	+5.88614
H	+1.50106	−2.40383	+3.96601

C	+2.87926	−2.19692	+5.64985
H	+2.86795	−2.31132	+6.76629
O	+3.03923	−0.76550	+5.30803
C	+3.15761	+0.13553	+6.45056
O	+4.47884	+0.75777	+6.41178
C	+4.72475	+1.53063	+5.16687
C	+6.16666	+2.02508	+5.31677
O	+6.68241	+2.53718	+4.08904
H	+6.21454	+3.38258	+3.87916
H	+6.21485	+2.80623	+6.12301
H	+6.81011	+1.17494	+5.63002
H	+4.63072	+0.83626	+4.29302
H	+3.10785	−0.44171	+7.41278
C	+2.09613	+1.22881	+6.39087
O	+0.79781	+0.59964	+6.41857
H	+0.11671	+1.26733	+6.13519
H	+2.20833	+1.88622	+7.30069
C	+2.25318	+2.08482	+5.13605
O	+1.29739	+3.16804	+5.22294
H	+1.54677	+3.85731	+4.53774
H	+2.04360	+1.46467	+4.21988
C	+3.67833	+2.64751	+5.05250
H	+3.83714	+3.41767	+5.85305
O	+3.81274	+3.28629	+3.72472
C	+4.10108	+4.71629	+3.73752
O	+5.41779	+4.92056	+3.13763
C	+5.50538	+4.37014	+1.76010
C	+6.96196	+4.60492	+1.34878
O	+7.32023	+3.82673	+0.20713
H	+6.82833	+4.17312	−0.57798
H	+7.12852	+5.69877	+1.15092
H	+7.62831	+4.30743	+2.18680
H	+5.27834	+3.27445	+1.80278
H	+4.16571	+5.09696	+4.79209
C	+3.06558	+5.47719	+2.91634
O	+1.77120	+5.26774	+3.52133
H	+1.06994	+5.56872	+2.88242
H	+3.31667	+6.57598	+2.94912
C	+3.06178	+5.00600	+1.46299
O	+2.17016	+5.87477	+0.72332

H	+2.33480	+5.72192	−0.25448
H	+2.68775	+3.94589	+1.40839
C	+4.47544	+5.07594	+0.86809
H	+4.76934	+6.14628	+0.70231
O	+4.43194	+4.37258	−0.43392
C	+4.79006	+5.18027	−1.59297
O	+5.98953	+4.60553	−2.19915
C	+5.80543	+3.19086	−2.61402
C	+7.18533	+2.76896	−3.12685
O	+7.27688	+1.35487	−3.29671
H	+6.71364	+1.09602	−4.06698
H	+7.40715	+3.29850	−4.09249
H	+7.95563	+3.07574	−2.38711
H	+5.51563	+2.59133	−1.71243
H	+5.06073	+6.22424	−1.27970
C	+3.66246	+5.16641	−2.61858
O	+2.48661	+5.72552	−1.99666
H	+1.69599	+5.51635	−2.56418
H	+3.96177	+5.81266	−3.49350
C	+3.39129	+3.74796	−3.11900
O	+2.40116	+3.84107	−4.16998
H	+2.41448	+2.98250	−4.68869
H	+2.99678	+3.11048	−2.27822
C	+4.68580	+3.12773	−3.66212
H	+5.00127	+3.65822	−4.59948
O	+4.39832	+1.70987	−3.97759
C	+4.59518	+1.33157	−5.37299
O	+5.66778	+0.34134	−5.43618
H	+4.94223	+2.21375	−5.97517
C	+3.32400	+0.71673	−5.94857
O	+2.27426	+1.70393	−5.87169
H	+1.39873	+1.25062	−6.00184
H	+3.50896	+0.45627	−7.03047
C	+2.93162	−0.55062	−5.19147
O	+1.79665	−1.13329	−5.87589
H	+1.68454	−2.07327	−5.54392
H	+2.64526	−0.29185	−4.13423
C	+4.10419	−1.54099	−5.17208
H	+4.27768	−1.95145	−6.20207
C	+5.38164	−0.87859	−4.63831

H	+5.23078	−0.57661	−3.56963
C	+6.64803	−1.72662	−4.79054
H	+6.71632	−2.11604	−5.84238
H	+7.53706	−1.08535	−4.60844
O	+6.68259	−2.80184	−3.85326
H	+5.98477	−3.45668	−4.10150

345

* E = 2.462 kcal/mol

C	−0.13247	+0.67873	−0.76148
C	−0.86854	+0.64886	+0.44268
C	−0.26421	+0.40147	+1.66733
C	+1.06358	+0.12649	+2.01167
C	+2.18603	−0.35170	+1.33137
C	+2.18050	−1.14758	+0.17651
C	+0.99444	−1.66334	−0.34224
H	+0.32345	−0.55737	−0.83862
C	+3.45565	−1.37962	−0.59015
C	+4.70337	−1.33172	+0.26715
C	+4.70754	−0.07989	+1.10461
C	+3.50575	−0.09007	+2.02786
C	−0.76393	+1.06095	−2.08714
C	−2.24812	+1.41676	−2.02075
C	−2.95640	+0.57604	−0.97906
C	−2.34209	+0.91595	+0.37661
C	−4.41819	+0.94480	−0.71202
C	−4.67026	+0.38919	+0.70035
C	−3.29277	+0.27876	+1.38063
O	+5.91087	−0.05254	+1.82110
H	+6.02892	+0.78362	+2.27937
C	−2.82960	−0.90722	−1.30852
C	−5.42734	+0.48433	−1.73912
H	+4.63859	+0.78884	+0.43653
H	+3.44780	+0.85998	+2.55744
H	+3.68672	−0.86308	+2.77957
H	+5.59658	−1.36148	−0.35107
H	+4.74082	−2.19176	+0.93614
H	+3.39606	−2.33226	−1.11451
H	+3.53114	−0.61361	−1.36902
H	+0.17592	−1.83528	+0.35094
H	+1.06722	−2.37755	−1.15367

H	+1.27416	+0.36591	+3.05093
H	−0.90999	+0.54727	+2.52562
H	+0.89575	+1.01878	−0.63233
H	−0.62772	+0.23389	−2.78714
H	−0.20746	+1.89259	−2.51892
H	−2.36649	+2.46723	−1.74588
H	−2.69737	+1.29612	−3.00804
H	−4.45293	+2.03670	−0.66214
H	−5.14667	−0.58951	+0.63650
H	−5.35285	+1.02670	+1.25650
H	−3.01622	−0.76207	+1.55203
H	−3.26837	+0.77568	+2.34892
H	−3.42110	−1.52255	−0.63246
H	−1.79882	−1.25076	−1.24433
H	−3.18226	−1.09942	−2.32146
H	−6.42252	+0.84201	−1.48728
H	−5.47423	−0.60288	−1.78815
H	−5.18607	+0.85631	−2.73423
H	−2.46382	+2.00230	+0.47641
O	−7.35590	−3.46684	−0.93316
H	−6.75677	−3.98036	−1.52903
C	−7.32031	−4.06028	+0.36418
H	−7.53330	−5.16174	+0.30157
H	−8.12255	−3.59424	+0.97575
C	−5.97226	−3.83524	+1.05526
H	−5.68180	−2.75336	+1.01711
O	−6.22508	−4.20514	+2.47206
C	−5.06276	−3.98868	+3.33192
O	−4.68913	−2.58242	+3.24881
H	−5.38466	−4.25224	+4.37508
C	−3.91929	−4.87192	+2.84601
O	−2.78068	−4.64644	+3.70339
H	−1.97106	−5.01856	+3.26040
H	−4.23679	−5.95133	+2.92821
C	−3.57218	−4.56456	+1.39025
O	−2.56544	−5.51647	+0.97284
H	−2.53045	−5.51469	−0.02976
H	−3.16697	−3.51740	+1.30349
C	−4.82489	−4.69953	+0.51448
H	−5.14048	−5.77523	+0.45993

O	−4.46513	−4.22710	−0.84129
C	−4.61695	−5.21382	−1.90475
O	−5.63619	−4.73314	−2.83514
C	−5.30366	−3.40922	−3.42097
C	−6.52037	−3.06603	−4.28573
O	−6.52492	−1.69203	−4.67120
H	−5.78885	−1.54446	−5.31473
H	−6.54035	−3.73134	−5.19108
H	−7.44577	−3.26037	−3.70209
H	−5.18918	−2.67003	−2.58648
H	−4.99730	−6.18588	−1.49041
C	−3.30569	−5.39230	−2.66220
O	−2.30916	−5.86347	−1.73039
H	−1.41085	−5.75336	−2.14289
H	−3.45738	−6.16868	−3.46626
C	−2.86212	−4.08214	−3.31048
O	−1.68963	−4.36735	−4.11098
H	−1.54239	−3.59242	−4.73074
H	−2.60422	−3.32998	−2.51436
C	−3.98392	−3.52237	−4.19612
H	−4.11789	−4.17200	−5.10145
O	−3.57018	−2.16656	−4.62472
C	−3.44689	−1.98009	−6.06623
O	−4.43283	−0.98797	−6.48945
C	−4.26667	+0.32222	−5.80931
C	−5.43191	+1.16684	−6.33355
O	−5.62304	+2.34600	−5.55290
H	−4.86885	+2.96204	−5.72461
H	−5.25343	+1.42990	−7.41111
H	−6.36571	+0.56679	−6.28118
H	−4.35816	+0.16308	−4.70367
H	−3.69877	−2.92991	−6.60991
C	−2.05346	−1.47375	−6.42192
O	−1.09542	−2.46189	−5.98833
H	−0.18839	−2.05312	−5.99679
H	−1.98641	−1.35901	−7.54212
C	−1.77558	−0.12239	−5.76542
O	−0.49263	+0.34295	−6.24794
H	−0.42287	+1.32447	−6.05142
H	−1.74107	−0.23958	−4.64547

C	−2.87535	+0.88108	−6.13702
H	−2.80544	+1.14292	−7.22603
O	−2.64709	+2.09829	−5.32607
C	−2.42083	+3.32413	−6.08526
O	−3.50831	+4.25420	−5.79107
C	−3.62723	+4.56025	−4.34208
C	−4.85303	+5.47374	−4.24934
O	−5.30336	+5.62023	−2.90332
H	−4.62452	+6.13040	−2.39674
H	−4.61101	+6.47559	−4.69761
H	−5.68251	+5.02982	−4.84060
H	−3.80341	+3.60093	−3.79184
H	−2.46551	+3.11876	−7.18843
C	−1.10023	+3.96850	−5.67749
O	−0.03759	+3.03366	−5.96674
H	+0.78061	+3.32416	−5.48237
H	−0.94926	+4.90250	−6.29085
C	−1.10356	+4.33519	−4.19496
O	+0.11788	+5.06295	−3.92001
H	+0.02622	+5.48443	−3.01374
H	−1.13315	+3.39828	−3.57346
C	−2.32255	+5.20259	−3.85221
H	−2.20188	+6.22892	−4.28959
O	−2.38050	+5.30157	−2.37600
C	−2.24849	+6.64876	−1.83342
O	−3.49561	+6.99325	−1.15360
C	−3.83763	+6.04831	−0.05844
C	−5.19459	+6.54169	+0.45217
O	−5.82603	+5.57697	+1.29283
H	−5.31646	+5.51604	+2.13809
H	−5.06153	+7.51543	+0.99701
H	−5.86219	+6.72790	−0.41642
H	−3.92690	+5.02034	−0.49343
H	−2.11956	+7.39698	−2.66104
C	−1.10600	+6.70928	−0.82603
O	+0.11856	+6.36366	−1.50767
H	+0.81176	+6.16488	−0.82241
H	−1.02647	+7.76472	−0.43581
C	−1.35621	+5.76265	+0.34663
O	−0.29167	+5.96774	+1.30556

H	−0.58095	+5.56982	+2.18001
H	−1.34670	+4.69522	−0.01114
C	−2.71504	+6.07305	+0.98806
H	−2.67937	+7.07308	+1.49637
O	−2.97532	+5.02159	+1.99662
C	−3.14208	+5.50024	+3.36456
O	−4.50086	+5.18396	+3.79919
C	−4.80009	+3.72950	+3.74968
C	−6.27368	+3.63633	+4.15681
O	−6.82309	+2.35205	+3.86468
H	−6.42787	+1.69278	+4.48678
H	−6.37934	+3.87275	+5.25004
H	−6.85448	+4.39431	+3.58845
H	−4.65557	+3.37334	+2.69788
H	−3.04819	+6.61875	+3.40486
C	−2.15299	+4.81407	+4.30043
O	−0.81771	+5.12920	+3.85400
H	−0.17674	+4.52856	+4.32228
H	−2.30134	+5.22414	+5.34087
C	−2.37259	+3.30228	+4.32744
O	−1.48549	+2.75237	+5.33087
H	−1.80083	+1.82537	+5.55070
H	−2.13118	+2.85797	+3.32138
C	−3.83216	+2.99103	+4.68411
H	−4.03410	+3.26815	+5.75271
O	−4.02257	+1.53376	+4.51292
C	−4.43581	+0.81228	+5.71218
O	−5.75724	+0.23886	+5.46978
H	−4.54729	+1.51771	+6.57884
C	−3.45859	−0.31741	+6.01674
O	−2.16050	+0.26662	+6.25863
H	−1.47497	−0.45332	+6.21789
H	−3.80351	−0.85246	+6.94755
C	−3.39561	−1.31305	+4.86019
O	−2.56214	−2.41905	+5.28174
H	−2.69065	−3.16840	+4.62667
H	−2.94260	−0.81995	+3.95560
C	−4.80251	−1.81952	+4.51204
H	−5.17747	−2.49826	+5.32345
C	−5.78366	−0.66115	+4.28790

H	−5.47178	−0.07211	+3.38805
C	−7.24970	−1.08959	+4.17434
H	−7.50449	−1.79130	+5.01450
H	−7.89857	−0.19296	+4.27386
O	−7.52809	−1.69434	+2.91199
H	−7.05763	−2.56306	+2.86824
O	+5.60328	−5.93410	+0.17644
H	+4.83827	−6.27824	+0.69983
C	+5.40140	−6.26679	−1.19586
H	+5.21434	−7.36882	−1.31364
H	+6.33366	−6.02077	−1.74835
C	+4.23726	−5.48075	−1.80638
H	+4.33950	−4.38588	−1.59215
O	+4.38565	−5.68857	−3.27050
C	+3.39759	−4.95089	−4.05319
O	+3.53593	−3.53078	−3.74696
H	+3.63596	−5.14646	−5.13316
C	+2.00430	−5.44331	−3.67876
O	+1.04001	−4.69971	−4.45469
H	+0.13827	−4.84183	−4.05940
H	+1.92561	−6.53723	−3.94184
C	+1.74417	−5.26954	−2.18357
O	+0.45611	−5.86286	−1.89547
H	+0.39044	−5.99694	−0.90263
H	+1.72562	−4.17331	−1.92276
C	+2.84660	−5.96420	−1.37280
H	+2.76628	−7.07755	−1.49056
O	+2.64156	−5.60592	+0.04783
C	+2.39692	−6.73007	+0.94471
O	+3.49048	−6.79645	+1.91091
C	+3.63017	−5.56079	+2.72584
C	+4.85096	−5.83866	+3.60834
O	+5.31921	−4.65880	+4.25824
H	+4.64539	−4.37657	+4.92382
H	+4.59540	−6.63207	+4.36274
H	+5.67498	−6.22912	+2.97319
H	+3.82312	−4.70055	+2.03581
H	+2.41667	−7.69888	+0.37646
C	+1.08511	−6.53888	+1.69697
O	+0.01538	−6.47217	+0.73027

H	−0.79306	−6.10518	+1.17896
H	+0.92178	−7.43165	+2.36715
C	+1.12138	−5.27457	+2.55318
O	−0.11081	−5.23077	+3.31216
H	+0.00709	−4.57017	+4.05813
H	+1.19648	−4.36465	+1.89577
C	+2.32708	−5.32203	+3.50124
H	+2.17856	−6.12615	+4.27032
O	+2.40841	−4.00842	+4.17798
C	+2.27702	−4.04453	+5.62971
O	+3.52764	−3.56906	+6.21715
C	+3.89207	−2.19637	+5.78000
C	+5.23606	−1.93781	+6.46837
O	+5.91130	−0.80762	+5.91927
H	+5.38901	+0.00595	+6.12551
H	+5.07228	−1.80341	+7.57293
H	+5.88982	−2.82531	+6.32940
H	+4.00715	−2.19636	+4.66575
H	+2.13791	−5.09980	+5.98833
C	+1.14560	−3.13218	+6.09003
O	−0.08715	−3.61890	+5.51959
H	−0.78474	−2.91733	+5.62483
H	+1.07796	−3.18204	+7.21490
C	+1.40582	−1.68796	+5.66799
O	+0.35975	−0.86626	+6.24181
H	+0.65345	+0.09122	+6.18304
H	+1.38153	−1.60413	+4.54609
C	+2.77483	−1.22265	+6.18198
H	+2.74587	−1.09489	+7.29669
O	+3.04027	+0.08722	+5.54639
C	+3.24880	+1.20540	+6.45557
O	+4.61124	+1.70380	+6.25356
C	+4.84695	+2.05047	+4.83045
C	+6.33990	+2.35390	+4.71468
O	+6.73106	+2.39691	+3.33579
H	+6.43231	+3.26355	+2.96207
H	+6.58495	+3.32362	+5.21967
H	+6.91993	+1.54813	+5.21205
H	+4.59571	+1.15336	+4.21060
H	+3.18123	+0.86952	+7.52470

C	+2.26820	+2.33137	+6.14114
O	+0.92974	+1.82225	+6.32953
H	+0.28976	+2.44360	+5.88990
H	+2.44738	+3.17908	+6.86302
C	+2.45199	+2.84442	+4.71224
O	+1.60961	+4.01114	+4.55692
H	+1.89638	+4.48907	+3.72290
H	+2.13959	+2.04939	+3.97814
C	+3.92022	+3.21554	+4.45972
H	+4.19347	+4.13132	+5.04762
O	+4.06338	+3.50335	+3.01319
C	+4.50150	+4.85839	+2.69506
O	+5.81102	+4.78156	+2.04877
C	+5.80649	+4.03162	+0.76523
C	+7.26971	+4.07714	+0.31205
O	+7.52901	+3.15947	−0.74738
H	+7.07105	+3.48043	−1.56253
H	+7.53358	+5.12498	+0.00342
H	+7.92212	+3.80346	+1.16951
H	+5.48313	+2.97761	+0.96800
H	+4.65308	+5.45161	+3.63677
C	+3.51597	+5.54099	+1.75284
O	+2.22718	+5.58362	+2.39994
H	+1.53661	+5.78423	+1.71251
H	+3.87194	+6.59577	+1.56762
C	+3.42488	+4.80837	+0.41609
O	+2.55747	+5.58228	−0.44635
H	+2.69524	+5.26718	−1.38863
H	+2.99361	+3.78000	+0.56729
C	+4.82109	+4.69114	−0.20795
H	+5.19222	+5.70520	−0.51374
O	+4.69719	+3.83299	−1.40654
C	+5.05846	+4.46699	−2.66972
O	+6.22133	+3.77196	−3.21711
C	+5.98158	+2.31926	−3.41933
C	+7.32283	+1.78477	−3.93124
O	+7.38934	+0.36161	−3.85907
H	+6.75595	−0.01962	−4.51556
H	+7.49182	+2.13707	−4.98534
H	+8.14056	+2.19676	−3.30169

H	+5.72429	+1.86316	−2.42992
H	+5.37181	+5.53237	−2.50268
C	+3.91052	+4.35692	−3.66639
O	+2.77331	+5.06128	−3.12480
H	+1.97098	+4.83021	−3.66606
H	+4.22296	+4.84587	−4.63329
C	+3.56296	+2.89373	−3.93338
O	+2.54924	+2.86685	−4.96677
H	+2.50007	+1.93188	−5.32843
H	+3.16147	+2.41889	−2.99490
C	+4.81153	+2.12956	−4.39454
H	+5.10396	+2.45923	−5.42674
O	+4.45614	+0.69368	−4.42109
C	+4.57789	+0.04156	−5.71958
O	+5.58892	−1.00749	−5.61227
H	+4.94762	+0.76551	−6.49431
C	+3.25079	−0.59521	−6.11502
O	+2.26531	+0.45380	−6.22719
H	+1.36104	+0.03972	−6.24791
H	+3.37281	−1.09731	−7.11737
C	+2.82132	−1.63585	−5.08228
O	+1.62244	−2.27560	−5.58021
H	+1.48701	−3.12706	−5.06627
H	+2.60395	−1.13263	−4.09864
C	+3.93390	−2.67466	−4.88662
H	+4.03340	−3.30709	−5.80845
C	+5.27312	−2.00215	−4.55440
H	+5.19305	−1.46366	−3.57598
C	+6.47666	−2.94940	−4.55563
H	+6.45892	−3.58155	−5.48473
H	+7.41056	−2.34750	−4.57317
O	+6.50495	−3.76968	−3.38871
H	+5.75530	−4.41276	−3.43659

345

* E = 2.555 kcal/mol

C	+0.61818	−0.70915	−0.40823
C	+1.18066	−0.38048	+0.84326
C	+0.42949	+0.18046	+1.86583
C	−0.91496	+0.55598	+1.93654
C	−1.90850	+0.85810	+1.00309

C	−1.71756	+1.31517	−0.30854
C	−0.45198	+1.66934	−0.77475
H	+0.23277	+0.47375	−0.86077
C	−2.86052	+1.31147	−1.29275
C	−4.24183	+1.37937	−0.66600
C	−4.33376	+0.40710	+0.48530
C	−3.32122	+0.79812	+1.53579
C	+1.41038	−1.42117	−1.48840
C	+2.85588	−1.75778	−1.12311
C	+3.44820	−0.69365	−0.22269
C	+2.63145	−0.67847	+1.06607
C	+4.83649	−1.00213	+0.34533
C	+4.91154	−0.10885	+1.59532
C	+3.45798	+0.17289	+2.01945
O	−5.62235	+0.29998	+1.03032
H	−5.93465	+1.16249	+1.30258
C	+3.43666	+0.66441	−0.91473
C	+6.00670	−0.83746	−0.59758
H	−4.10102	−0.59599	+0.12324
H	−3.38826	+0.10081	+2.36690
H	−3.60787	+1.78239	+1.92259
H	−5.00592	+1.15844	−1.40719
H	−4.43088	+2.38802	−0.28925
H	−2.73079	+2.12463	−2.00574
H	−2.79137	+0.38916	−1.87821
H	+0.26790	+2.02440	−0.04283
H	−0.38552	+2.13830	−1.74915
H	−1.27576	+0.59169	+2.96025
H	+0.94432	+0.24728	+2.81765
H	−0.43303	−0.99357	−0.35129
H	+1.40607	−0.79992	−2.38665
H	+0.88283	−2.33211	−1.77048
H	+2.89148	−2.71115	−0.59135
H	+3.44245	−1.88160	−2.03518
H	+4.80508	−2.04527	+0.67245
H	+5.42865	+0.82078	+1.35808
H	+5.48493	−0.58596	+2.38528
H	+3.20468	+1.22732	+1.90284
H	+3.27188	−0.08145	+3.06115
H	+3.94523	+1.42225	−0.32078

H	+2.42281	+1.01841	−1.09394
H	+3.94258	+0.60600	−1.87811
H	+6.93608	−1.10909	−0.10291
H	+6.10604	+0.19391	−0.93344
H	+5.90710	−1.47264	−1.47714
H	+2.67941	−1.70798	+1.44316
O	−7.15721	−3.62842	−2.08234
H	−6.49667	−4.01417	−2.70793
C	−7.15283	−4.40190	−0.88320
H	−7.31694	−5.48979	−1.11389
H	−7.99956	−4.05790	−0.25165
C	−5.84735	−4.23641	−0.09973
H	−5.61147	−3.15406	+0.06012
O	−6.14033	−4.85498	+1.21933
C	−5.03113	−4.73753	+2.16408
O	−4.72607	−3.32409	+2.34411
H	−5.38514	−5.19236	+3.12817
C	−3.82589	−5.47776	+1.59456
O	−2.73430	−5.35596	+2.53037
H	−1.89299	−5.61024	+2.06498
H	−4.09462	−6.56744	+1.47926
C	−3.43177	−4.91584	+0.23006
O	−2.36291	−5.74322	−0.29019
H	−2.27958	−5.56072	−1.27257
H	−3.07160	−3.85519	+0.34068
C	−4.63645	−4.94812	−0.71962
H	−4.89652	−6.01001	−0.97374
O	−4.23786	−4.23525	−1.95475
C	−4.27040	−5.03316	−3.17447
O	−5.26868	−4.45751	−4.07403
C	−4.99900	−3.03197	−4.39294
C	−6.18401	−2.61158	−5.26750
O	−6.26841	−1.19314	−5.40016
H	−5.50237	−0.88020	−5.94106
H	−6.10063	−3.09950	−6.27663
H	−7.12537	−2.96677	−4.79618
H	−4.98736	−2.45196	−3.43555
H	−4.60585	−6.08191	−2.95388
C	−2.91512	−5.00106	−3.87227
O	−1.93517	−5.58684	−2.98981

H	−1.02902	−5.38871	−3.34911
H	−2.98015	−5.61774	−4.81432
C	−2.52544	−3.56993	−4.23722
O	−1.28996	−3.63155	−4.99025
H	−1.16546	−2.75051	−5.45391
H	−2.36911	−2.96542	−3.30036
C	−3.63106	−2.92098	−5.08084
H	−3.66490	−3.39304	−6.09846
O	−3.28858	−1.48765	−5.21774
C	−3.10050	−1.01684	−6.58455
O	−4.11531	−0.00299	−6.86392
C	−4.06825	+1.13317	−5.90791
C	−5.24596	+2.02158	−6.31910
O	−5.55091	+2.99134	−5.31748
H	−4.81641	+3.65267	−5.28611
H	−5.01784	+2.52225	−7.29889
H	−6.14546	+1.38571	−6.46452
H	−4.21747	+0.73081	−4.87382
H	−3.26931	−1.84981	−7.31842
C	−1.72218	−0.38477	−6.74088
O	−0.72950	−1.39746	−6.47136
H	+0.14987	−0.95235	−6.33709
H	−1.60594	−0.02571	−7.80360
C	−1.55877	+0.80081	−5.79139
O	−0.28137	+1.41743	−6.07898
H	−0.27734	+2.33216	−5.66629
H	−1.57656	+0.44225	−4.72409
C	−2.69432	+1.81033	−6.00713
H	−2.57754	+2.31058	−7.00493
O	−2.58480	+2.82580	−4.93559
C	−2.38256	+4.19586	−5.39475
O	−3.53527	+4.99338	−4.98303
C	−3.74472	+4.99025	−3.51202
C	−5.03104	+5.79759	−3.31507
O	−5.54997	+5.65090	−1.99377
H	−4.94261	+6.11489	−1.36628
H	−4.83630	+6.87972	−3.54594
H	−5.80094	+5.43195	−4.02798
H	−3.88455	+3.93054	−3.17664
H	−2.35227	+4.23559	−6.51668

C	−1.12478	+4.78844	−4.76948
O	+0.00259	+3.98449	−5.17752
H	+0.78138	+4.21618	−4.60344
H	−0.98981	+5.83864	−5.15813
C	−1.23511	+4.83120	−3.24636
O	−0.05918	+5.50877	−2.74500
H	−0.23613	+5.77576	−1.79365
H	−1.27579	+3.78266	−2.83454
C	−2.50483	+5.58725	−2.83226
H	−2.40538	+6.67541	−3.08844
O	−2.64695	+5.44036	−1.36680
C	−2.63174	+6.68794	−0.61143
O	−3.93141	+6.84784	+0.03764
C	−4.26061	+5.72751	+0.95594
C	−5.67484	+6.05043	+1.44675
O	−6.27107	+4.93537	+2.10976
H	−5.82960	+4.82103	+2.98729
H	−5.64485	+6.94384	+2.12628
H	−6.31064	+6.30956	+0.57297
H	−4.25287	+4.77334	+0.36832
H	−2.51196	+7.56550	−1.30217
C	−1.54567	+6.65531	+0.45829
O	−0.27364	+6.48544	−0.20165
H	+0.40508	+6.22271	+0.47674
H	−1.55012	+7.64280	+1.00393
C	−1.79067	+5.53139	+1.46407
O	−0.79328	+5.65581	+2.50603
H	−1.09214	+5.10547	+3.29021
H	−1.69024	+4.53171	+0.95781
C	−3.19838	+5.66118	+2.06169
H	−3.25762	+6.57109	+2.71596
O	−3.43963	+4.45313	+2.88094
C	−3.69752	+4.69397	+4.29633
O	−5.05498	+4.23948	+4.59690
C	−5.25929	+2.81469	+4.23376
C	−6.73908	+2.54023	+4.51488
O	−7.18519	+1.37091	+3.82755
H	−6.77598	+0.57860	+4.25761
H	−6.90840	+2.44003	+5.62064
H	−7.34429	+3.40047	+4.15551

H	−5.04484	+2.69943	+3.13950
H	−3.66856	+5.79318	+4.52380
C	−2.71702	+3.90287	+5.15581
O	−1.38418	+4.37259	+4.85790
H	−0.72847	+3.71093	+5.20643
H	−2.94606	+4.10035	+6.24211
C	−2.83620	+2.40549	+4.87804
O	−1.98937	+1.70953	+5.82477
H	−2.23640	+0.73709	+5.79660
H	−2.48992	+2.18737	+3.83012
C	−4.29019	+1.93952	+5.03879
H	−4.57511	+1.93521	+6.12432
O	−4.36507	+0.56185	+4.50543
C	−4.80935	−0.45514	+5.45213
O	−6.07239	−1.00830	+4.97675
H	−5.02484	+0.00962	+6.45240
C	−3.77331	−1.56863	+5.55761
O	−2.52635	−0.98117	+5.99228
H	−1.79452	−1.63048	+5.81584
H	−4.12027	−2.31263	+6.33122
C	−3.59397	−2.28646	+4.22177
O	−2.69347	−3.39857	+4.44504
H	−2.76991	−4.02116	+3.66194
H	−3.14705	−1.58347	+3.46448
C	−4.94755	−2.79635	+3.70971
H	−5.31594	−3.62636	+4.37051
C	−5.98659	−1.66813	+3.64248
H	−5.68477	−0.90610	+2.87390
C	−7.42013	−2.15385	+3.39975
H	−7.65248	−2.99860	+4.10684
H	−8.12549	−1.32484	+3.62544
O	−7.63606	−2.55543	+2.04952
H	−7.06284	−3.33680	+1.85700
O	+5.86971	−5.74882	−0.77548
H	+5.09026	−6.19950	−0.36614
C	+5.74290	−5.81644	−2.19517
H	+5.61044	−6.88076	−2.53135
H	+6.68723	−5.43606	−2.64067
C	+4.57275	−4.96752	−2.70125
H	+4.60947	−3.93871	−2.26006

O	+4.80484	−4.85788	−4.16478
C	+3.81257	−4.02043	−4.83448
O	+3.83743	−2.69739	−4.21821
H	+4.12137	−3.95891	−5.91245
C	+2.43781	−4.65949	−4.66920
O	+1.46858	−3.81609	−5.33026
H	+0.56064	−4.07344	−5.01743
H	+2.44758	−5.67191	−5.16521
C	+2.08480	−4.82446	−3.19266
O	+0.83948	−5.55960	−3.11728
H	+0.71431	−5.85369	−2.16574
H	+1.95492	−3.81208	−2.72066
C	+3.19112	−5.58578	−2.44969
H	+3.18405	−6.66714	−2.74943
O	+2.89312	−5.47470	−1.00373
C	+2.65518	−6.73777	−0.31472
O	+3.70377	−6.91640	+0.68764
C	+3.74999	−5.80994	+1.67904
C	+4.95191	−6.14956	+2.56507
O	+5.32855	−5.04758	+3.38988
H	+4.62382	−4.91042	+4.06988
H	+4.71516	−7.05107	+3.19250
H	+5.81849	−6.40499	+1.91807
H	+3.92040	−4.84925	+1.12909
H	+2.74648	−7.60014	−1.02845
C	+1.30130	−6.71669	+0.38534
O	+0.27730	−6.52873	−0.61483
H	−0.56853	−6.27913	−0.15368
H	+1.14363	−7.71137	+0.89343
C	+1.24058	−5.60714	+1.43391
O	−0.02117	−5.73780	+2.12929
H	+0.03288	−5.20112	+2.97543
H	+1.29836	−4.60049	+0.93290
C	+2.40719	−5.75307	+2.42002
H	+2.26871	−6.67598	+3.04346
O	+2.39322	−4.56279	+3.29995
C	+2.21318	−4.84370	+4.72050
O	+3.41702	−4.41546	+5.42980
C	+3.70505	−2.96736	+5.26428
C	+5.02551	−2.76186	+6.01276

O	+5.61436	−1.49695	+5.71269
H	+5.06463	−0.78807	+6.12814
H	+4.85265	−2.86548	+7.11800
H	+5.74200	−3.55408	+5.70621
H	+3.82954	−2.75316	+4.17219
H	+2.12033	−5.94955	+4.89389
C	+1.01547	−4.08122	+5.27630
O	−0.16278	−4.50715	+4.56141
H	−0.90178	−3.87032	+4.75885
H	+0.90079	−4.34433	+6.36752
C	+1.20845	−2.57085	+5.14975
O	+0.08656	−1.93720	+5.80949
H	+0.32221	−0.97581	+5.97190
H	+1.23041	−2.27279	+4.06452
C	+2.52594	−2.15653	+5.81903
H	+2.45054	−2.28854	+6.93096
O	+2.74918	−0.72631	+5.51099
C	+2.81803	+0.15827	+6.66952
O	+4.14992	+0.75869	+6.71163
C	+4.48578	+1.50088	+5.46927
C	+5.91624	+1.99804	+5.69814
O	+6.53305	+2.41109	+4.47884
H	+6.07250	+3.22217	+4.14980
H	+5.91121	+2.83864	+6.44450
H	+6.52405	+1.17108	+6.12436
H	+4.45181	+0.78316	+4.61088
H	+2.70057	−0.42577	+7.62136
C	+1.78511	+1.27241	+6.54851
O	+0.47291	+0.67161	+6.53669
H	−0.18763	+1.35449	+6.24050
H	+1.87496	+1.94869	+7.44650
C	+2.01866	+2.08856	+5.27911
O	+1.08271	+3.19277	+5.29115
H	+1.37966	+3.85595	+4.59910
H	+1.83739	+1.44557	+4.37219
C	+3.45854	+2.61949	+5.24643
H	+3.58636	+3.42750	+6.01496
O	+3.67973	+3.18757	+3.89764
C	+4.02216	+4.60352	+3.86318
O	+5.35886	+4.73586	+3.28661

C	+5.45718	+4.15624	+1.92219
C	+6.93376	+4.31964	+1.55081
O	+7.27661	+3.55169	+0.39755
H	+6.84508	+3.96284	−0.39132
H	+7.16369	+5.40670	+1.38486
H	+7.56106	+3.96561	+2.39691
H	+5.17913	+3.07204	+1.97584
H	+4.08023	+5.02312	+4.90327
C	+3.03225	+5.36823	+2.99187
O	+1.71886	+5.22656	+3.57307
H	+1.04060	+5.50597	+2.90100
H	+3.32019	+6.45896	+2.98950
C	+3.05089	+4.84543	+1.55620
O	+2.17718	+5.69429	+0.77519
H	+2.38590	+5.54946	−0.19533
H	+2.67756	+3.78347	+1.52784
C	+4.48007	+4.89796	+0.99941
H	+4.80204	+5.96538	+0.87040
O	+4.46449	+4.23041	−0.32211
C	+4.86727	+5.06837	−1.44656
O	+6.08555	+4.50710	−2.02693
C	+5.91331	+3.10762	−2.49455
C	+7.30612	+2.70065	−2.98405
O	+7.40712	+1.28922	−3.17091
H	+6.85386	+1.03631	−3.95039
H	+7.54656	+3.24436	−3.93741
H	+8.05932	+2.99983	−2.22384
H	+5.60164	+2.47802	−1.62191
H	+5.13058	+6.10162	−1.09394
C	+3.78037	+5.09277	−2.51579
O	+2.58325	+5.64531	−1.92894
H	+1.81394	+5.44230	−2.52559
H	+4.12110	+5.76018	−3.35901
C	+3.51616	+3.69210	−3.06486
O	+2.56757	+3.82180	−4.15113
H	+2.58146	+2.96795	−4.67768
H	+3.08215	+3.03906	−2.25749
C	+4.82324	+3.06989	−3.57465
H	+5.16933	+3.60431	−4.49887
O	+4.53449	+1.65627	−3.90723

C	+4.76048	+1.27944	−5.29797
O	+5.82610	+0.27997	−5.33534
H	+5.12765	+2.15975	−5.89075
C	+3.49636	+0.67450	−5.89956
O	+2.45376	+1.67061	−5.84323
H	+1.57968	+1.22691	−6.01349
H	+3.70049	+0.41264	−6.97760
C	+3.07887	−0.59083	−5.15102
O	+1.95536	−1.16740	−5.85862
H	+1.84910	−2.11755	−5.55358
H	+2.77257	−0.33162	−4.09799
C	+4.24486	−1.58732	−5.10847
H	+4.44561	−1.99085	−6.13614
C	+5.50685	−0.92973	−4.53418
H	+5.31722	−0.61367	−3.47595
C	+6.77151	−1.78846	−4.62301
H	+6.87202	−2.21076	−5.65885
H	+7.65865	−1.14785	−4.42975
O	+6.76408	−2.83464	−3.65188
H	+6.09392	−3.50867	−3.92433

345

* E = 2.594 kcal/mol

C	−0.36190	+0.88123	−0.06865
C	−1.13321	−0.02346	+0.68303
C	−0.61173	−1.17193	+1.25919
C	+0.68144	−1.69426	+1.24530
C	+1.81527	−1.52222	+0.44359
C	+1.83587	−1.13870	−0.90224
C	+0.65325	−0.97849	−1.63319
H	+0.04325	+0.14294	−1.13350
C	+3.14449	−0.82980	−1.58258
C	+4.31912	−1.60217	−1.01240
C	+4.36774	−1.41146	+0.48432
C	+3.10387	−1.96078	+1.10824
C	−1.02027	+2.18051	−0.44302
C	−2.41766	+2.02328	−1.05590
C	−3.17472	+0.80823	−0.51302
C	−2.56924	+0.36345	+0.82604
C	−4.61850	+1.07682	−0.07199
C	−4.93902	−0.10699	+0.85022

C	−3.58850	−0.61280	+1.39070
O	+5.50860	−1.95779	+1.08642
H	+5.54581	−2.90749	+0.94463
C	−3.11651	−0.32426	−1.52989
C	−5.63068	+1.29605	−1.17287
H	+4.43095	−0.34171	+0.69124
H	+3.09333	−1.69281	+2.16256
H	+3.16157	−3.05357	+1.06779
H	+5.25211	−1.26822	−1.45936
H	+4.20671	−2.66713	−1.23636
H	+3.05038	−1.01942	−2.65110
H	+3.34958	+0.24061	−1.48313
H	−0.19322	−1.58488	−1.32346
H	+0.74311	−0.83913	−2.70380
H	+0.85933	−2.36677	+2.07970
H	−1.28484	−1.69373	+1.92909
H	+0.68463	+0.95338	+0.22697
H	−0.37718	+2.75007	−1.11192
H	−1.10176	+2.78055	+0.46934
H	−2.98662	+2.93407	−0.86426
H	−2.33093	+1.94232	−2.13979
H	−4.57996	+1.98321	+0.53927
H	−5.44330	−0.89191	+0.28715
H	−5.61829	+0.18555	+1.64628
H	−3.38311	−1.62388	+1.03799
H	−3.55756	−0.64788	+2.47848
H	−3.63611	−1.21780	−1.18717
H	−2.08481	−0.59810	−1.74039
H	−3.56917	−0.01367	−2.47052
H	−6.60624	+1.53169	−0.75476
H	−5.74696	+0.40730	−1.79117
H	−5.34279	+2.12441	−1.82029
H	−2.59072	+1.26030	+1.46015
O	−7.18954	−3.40616	−1.56506
H	−6.63536	−3.57314	−2.36697
C	−6.98281	−4.47590	−0.64290
H	−7.14339	−5.46803	−1.14511
H	−7.73731	−4.37986	+0.16719
C	−5.58142	−4.42940	−0.02720
H	−5.35033	−3.40171	+0.35433

O	−5.65945	−5.35496	+1.13236
C	−4.42448	−5.38172	+1.91381
O	−4.14010	−4.02458	+2.36093
H	−4.61499	−6.05727	+2.79044
C	−3.29248	−5.89369	+1.03071
O	−2.08434	−5.91040	+1.82041
H	−1.30452	−6.02375	+1.21245
H	−3.53522	−6.94528	+0.70275
C	−3.12407	−5.01179	−0.20616
O	−2.12543	−5.63346	−1.04925
H	−2.20660	−5.24253	−1.96973
H	−2.78125	−3.98333	+0.09779
C	−4.45729	−4.90304	−0.95872
H	−4.72190	−5.89462	−1.41316
O	−4.27374	−3.90591	−2.03752
C	−4.51391	−4.40144	−3.38839
O	−5.64312	−3.66696	−3.95486
C	−5.41349	−2.20022	−4.00509
C	−6.73120	−1.63258	−4.54009
O	−6.79717	−0.21553	−4.38234
H	−6.15510	+0.19820	−5.00999
H	−6.85431	−1.91642	−5.61997
H	−7.57562	−2.08013	−3.97322
H	−5.21820	−1.83546	−2.96408
H	−4.81892	−5.48205	−3.36353
C	−3.28850	−4.17786	−4.26792
O	−2.18158	−4.89736	−3.68459
H	−1.33605	−4.56484	−4.08961
H	−3.50155	−4.59498	−5.29418
C	−2.96151	−2.69086	−4.39376
O	−1.87714	−2.56370	−5.34456
H	−1.81942	−1.60126	−5.62167
H	−2.64171	−2.28178	−3.39531
C	−4.19175	−1.91895	−4.89035
H	−4.41187	−2.19111	−5.95664
O	−3.86755	−0.47606	−4.81201
C	−3.89129	+0.24003	−6.08305
O	−4.95555	+1.23996	−6.02869
C	−4.77924	+2.21403	−4.92048
C	−6.02536	+3.10075	−4.99992

O	−6.18458	+3.89363	−3.82414
H	−5.48043	+4.58769	−3.81659
H	−5.96784	+3.75329	−5.91259
H	−6.92361	+2.45442	−5.10119
H	−4.75505	+1.64929	−3.95319
H	−4.15281	−0.45753	−6.92345
C	−2.56272	+0.94866	−6.32465
O	−1.52263	−0.05038	−6.36974
H	−0.64148	+0.40457	−6.29048
H	−2.61288	+1.47619	−7.32050
C	−2.27956	+1.97664	−5.23061
O	−1.07080	+2.68480	−5.59520
H	−1.02013	+3.51937	−5.04028
H	−2.13230	+1.45579	−4.24237
C	−3.45184	+2.95951	−5.11397
H	−3.49839	+3.61458	−6.02399
O	−3.19777	+3.79688	−3.92034
C	−3.08473	+5.22956	−4.17084
O	−4.18156	+5.90369	−3.47931
C	−4.19741	+5.62158	−2.02081
C	−5.44457	+6.34819	−1.50822
O	−5.81210	+5.90968	−0.20104
H	−5.12006	+6.20877	+0.43921
H	−5.26657	+7.45799	−1.51888
H	−6.29501	+6.13544	−2.19107
H	−4.29598	+4.51482	−1.87601
H	−3.21107	+5.45133	−5.26445
C	−1.76298	+5.76204	−3.62757
O	−0.68921	+5.08692	−4.31937
H	+0.15202	+5.22689	−3.80828
H	−1.70331	+6.86781	−3.83931
C	−1.65945	+5.52918	−2.12184
O	−0.45371	+6.18234	−1.65525
H	−0.49625	+6.22333	−0.65349
H	−1.59462	+4.42562	−1.91615
C	−2.88251	+6.10702	−1.39623
H	−2.83745	+7.22831	−1.39918
O	−2.82518	+5.61746	+0.00009
C	−2.73577	+6.65301	+1.02280
O	−3.93935	+6.58632	+1.84935

C	−4.12150	+5.26102	+2.49679
C	−5.46502	+5.37659	+3.22263
O	−5.94879	+4.10148	+3.64317
H	−5.37133	+3.77395	+4.37616
H	−5.36184	+6.07026	+4.10042
H	−6.21458	+5.81567	+2.52973
H	−4.16911	+4.47948	+1.69597
H	−2.72866	+7.67287	+0.55243
C	−1.51502	+6.42456	+1.90692
O	−0.33850	+6.47518	+1.07177
H	+0.42725	+6.10069	+1.58524
H	−1.46297	+7.25362	+2.67004
C	−1.60254	+5.08380	+2.63485
O	−0.47813	+5.00862	+3.54192
H	−0.66757	+4.28863	+4.21510
H	−1.54938	+4.23826	+1.89088
C	−2.92090	+4.99347	+3.41451
H	−2.91300	+5.72486	+4.26576
O	−3.02156	+3.61853	+3.95324
C	−3.11123	+3.52746	+5.40703
O	−4.40239	+2.93737	+5.75265
C	−4.59660	+1.57895	+5.18253
C	−6.01735	+1.19837	+5.60953
O	−6.48235	+0.03336	+4.92933
H	−5.97782	−0.74786	+5.26429
H	−6.04703	+1.04499	+6.72204
H	−6.70557	+2.03574	+5.36447
H	−4.51994	+1.64738	+4.06743
H	−3.09893	+4.55229	+5.86651
C	−1.99912	+2.64441	+5.96205
O	−0.73211	+3.23248	+5.60065
H	−0.01729	+2.55417	+5.74038
H	−2.08856	+2.62087	+7.08648
C	−2.10479	+1.21780	+5.42664
O	−1.09352	+0.42845	+6.09615
H	−1.30552	−0.54056	+5.94707
H	−1.92372	+1.20760	+4.31572
C	−3.49975	+0.65066	+5.72117
H	−3.62701	+0.49719	+6.82559
O	−3.59884	−0.65523	+5.03192

C	−3.80080	−1.80735	+5.90455
O	−5.10195	−2.39564	+5.59377
H	−3.84352	−1.48660	+6.97988
C	−2.72284	−2.85765	+5.66260
O	−1.44328	−2.28178	+5.99990
H	−0.72696	−2.87711	+5.65058
H	−2.92125	−3.73928	+6.33729
C	−2.73778	−3.32435	+4.20885
O	−1.77149	−4.39531	+4.08316
H	−1.93799	−4.85824	+3.20868
H	−2.44992	−2.47221	+3.53055
C	−4.13782	−3.82481	+3.82697
H	−4.34807	−4.80284	+4.33593
C	−5.22543	−2.79665	+4.16848
H	−5.09119	−1.88467	+3.53295
C	−6.65589	−3.33154	+4.04986
H	−6.72857	−4.33405	+4.55344
H	−7.34681	−2.63822	+4.57611
O	−7.07734	−3.41992	+2.68925
H	−6.54803	−4.12346	+2.23863
O	+5.90749	−5.09062	−2.64383
H	+5.22097	−5.65284	−2.20629
C	+5.53888	−4.90972	−4.01101
H	+5.35605	−5.90176	−4.50613
H	+6.39027	−4.42225	−4.53261
C	+4.29614	−4.02568	−4.14987
H	+4.40154	−3.09553	−3.53518
O	+4.28293	−3.63983	−5.58473
C	+3.19643	−2.71972	−5.91120
O	+3.33195	−1.53439	−5.07068
H	+3.31889	−2.45169	−6.99483
C	+1.86756	−3.41574	−5.64077
O	+0.80423	−2.49961	−5.97691
H	−0.04820	−2.85090	−5.60200
H	+1.79554	−4.32539	−6.30366
C	+1.76716	−3.85131	−4.18013
O	+0.54279	−4.60962	−4.03220
H	+0.59562	−5.11358	−3.16605
H	+1.73819	−2.94677	−3.51033
C	+2.97283	−4.72370	−3.80562

H	+2.90655	−5.71614	−4.32568
O	+2.91151	−4.93329	−2.34220
C	+2.84415	−6.32202	−1.90762
O	+4.04788	−6.60893	−1.12599
C	+4.17662	−5.68745	+0.02910
C	+5.56006	−5.96064	+0.61869
O	+5.92546	−4.92271	+1.53508
H	+5.46287	−5.09954	+2.39151
H	+5.57735	−6.95722	+1.12971
H	+6.31118	−5.97991	−0.19963
H	+4.12476	−4.63473	−0.35475
H	+2.86185	−7.01549	−2.79068
C	+1.61697	−6.55017	−1.02923
O	+0.45035	−6.21845	−1.81180
H	−0.32997	−6.12296	−1.20186
H	+1.57596	−7.64201	−0.74796
C	+1.67036	−5.71269	+0.25027
O	+0.56398	−6.12663	+1.08671
H	+0.73164	−5.78321	+2.01427
H	+1.57125	−4.61970	+0.00100
C	+3.00115	−5.94721	+0.97950
H	+3.04652	−6.99414	+1.38014
O	+3.06978	−4.98964	+2.10483
C	+3.17991	−5.58573	+3.43361
O	+4.47308	−5.21779	+4.00312
C	+4.67169	−3.75353	+4.18509
C	+6.05574	−3.65904	+4.83791
O	+6.59236	−2.34097	+4.77898
H	+6.00804	−1.73214	+5.29389
H	+5.99009	−4.01570	+5.90358
H	+6.75619	−4.33589	+4.30154
H	+4.66983	−3.26206	+3.17820
H	+3.17722	−6.70666	+3.35892
C	+2.06374	−5.07472	+4.33824
O	+0.80040	−5.43792	+3.73789
H	+0.08319	−4.89136	+4.15590
H	+2.15426	−5.57716	+5.34386
C	+2.15566	−3.56229	+4.52008
O	+1.13797	−3.16810	+5.47232
H	+1.32530	−2.21972	+5.74265

H	+1.96996	−3.05037	+3.53545
C	+3.54287	−3.16448	+5.04076
H	+3.65530	−3.47485	+6.11367
O	+3.63089	−1.69057	+4.94297
C	+3.81774	−0.98461	+6.20411
O	+5.11684	−0.31720	+6.16895
C	+5.25158	+0.62379	+5.02518
C	+6.68792	+1.14354	+5.13784
O	+7.10539	+1.81793	+3.95221
H	+6.57873	+2.64911	+3.85882
H	+6.77348	+1.82175	+6.03093
H	+7.37213	+0.28327	+5.29978
H	+5.10878	+0.04935	+4.07568
H	+3.85228	−1.70844	+7.06235
C	+2.72942	+0.06687	+6.38381
O	+1.45337	−0.60805	+6.41695
H	+0.73676	+0.07059	+6.29264
H	+2.89437	+0.59535	+7.36652
C	+2.77032	+1.09178	+5.25146
O	+1.79616	+2.11835	+5.55861
H	+2.00026	+2.91980	+4.99062
H	+2.50647	+0.59663	+4.27601
C	+4.17184	+1.70841	+5.14777
H	+4.37146	+2.35651	+6.04236
O	+4.19058	+2.54519	+3.92619
C	+4.48599	+3.95669	+4.14137
O	+5.72925	+4.27848	+3.44369
C	+5.66415	+4.00088	+1.98531
C	+7.07413	+4.32217	+1.48120
O	+7.28626	+3.84127	+0.15467
H	+6.73012	+4.37319	−0.46555
H	+7.24865	+5.43130	+1.53292
H	+7.81652	+3.83082	+2.14587
H	+5.42706	+2.91645	+1.84136
H	+4.67042	+4.16052	+5.23036
C	+3.37052	+4.83154	+3.57954
O	+2.14603	+4.50638	+4.27030
H	+1.38432	+4.88095	+3.75190
H	+3.62915	+5.91226	+3.77442
C	+3.21334	+4.62191	+2.07468

O	+2.22303	+5.56779	+1.60407
H	+2.31058	+5.63954	+0.60755
H	+2.86496	+3.57186	+1.86696
C	+4.55380	+4.86321	+1.36862
H	+4.82768	+5.95009	+1.42699
O	+4.37574	+4.48093	−0.05055
C	+4.60240	+5.54958	−1.01683
O	+5.74001	+5.17760	−1.85546
C	+5.53588	+3.88932	−2.56678
C	+6.85545	+3.65853	−3.30924
O	+6.96027	+2.32353	−3.80216
H	+6.30414	+2.20460	−4.53193
H	+6.95001	+4.39936	−4.14905
H	+7.69998	+3.83127	−2.60821
H	+5.36348	+3.08789	−1.80436
H	+4.88948	+6.50121	−0.49414
C	+3.38022	+5.73577	−1.90927
O	+2.26212	+6.11000	−1.07722
H	+1.42936	+6.03379	−1.61619
H	+3.59222	+6.56844	−2.63988
C	+3.06996	+4.45767	−2.68611
O	+1.97505	+4.75056	−3.58709
H	+1.93489	+4.02043	−4.27419
H	+2.76753	+3.63987	−1.97381
C	+4.30622	+4.01046	−3.47769
H	+4.50868	+4.73189	−4.31321
O	+4.00367	+2.67748	−4.04540
C	+4.04075	+2.59624	−5.50097
O	+5.10359	+1.66884	−5.88247
H	+4.31014	+3.59121	−5.94652
C	+2.71544	+2.06890	−6.03863
O	+1.67483	+2.99584	−5.66356
H	+0.79359	+2.55461	−5.79903
H	+2.77695	+2.01691	−7.16359
C	+2.42134	+0.67411	−5.48996
O	+1.21526	+0.19850	−6.13324
H	+1.15028	−0.79123	−5.97956
H	+2.26403	+0.72351	−4.37577
C	+3.59185	−0.27027	−5.79527
H	+3.64066	−0.47760	−6.89710

C	+4.92292	+0.31338	−5.30170
H	+4.90300	+0.40602	−4.18597
C	+6.16325	−0.45852	−5.76142
H	+6.10421	−0.64139	−6.86863
H	+7.06636	+0.15904	−5.56755
O	+6.31327	−1.68953	−5.05567
H	+5.58943	−2.30263	−5.33406

345

* E = 2.872 kcal/mol

C	−0.65668	−0.59998	−0.39071
C	−0.95383	+0.77277	−0.31234
C	+0.01505	+1.76517	−0.30139
C	+1.40780	+1.68858	−0.35114
C	+2.35793	+0.69036	−0.10951
C	+2.21150	−0.40871	+0.74421
C	+1.09282	−0.54526	+1.57387
H	+0.04405	−0.83733	+0.74946
C	+3.23188	−1.51585	+0.73512
C	+4.62435	−1.05820	+0.35245
C	+4.57485	−0.27118	−0.93030
C	+3.71324	+0.95967	−0.73391
C	−1.81931	−1.53246	−0.58898
C	−2.98356	−1.29107	+0.37888
C	−3.14325	+0.18323	+0.76208
C	−2.41569	+1.07742	−0.25247
C	−4.56411	+0.74514	+0.63538
C	−4.34698	+2.26335	+0.58217
C	−2.91255	+2.47779	+0.06471
O	+5.89089	+0.07109	−1.27039
H	+5.93614	+0.47780	−2.13859
C	−2.59062	+0.40931	+2.16298
C	−5.56169	+0.29914	+1.67948
H	+4.12911	−0.90186	−1.70928
H	+3.57636	+1.46053	−1.69202
H	+4.27709	+1.64538	−0.09489
H	+5.29107	−1.90825	+0.23501
H	+5.04354	−0.42043	+1.13205
H	+3.25147	−1.99728	+1.71167
H	+2.90352	−2.28388	+0.02755
H	+0.60945	+0.37434	+1.89101

H	+1.12263	−1.32515	+2.32545
H	+1.85510	+2.61626	−0.69674
H	−0.36684	+2.77610	−0.38059
H	+0.24724	−0.84836	−0.94634
H	−1.48863	−2.56807	−0.52882
H	−2.17958	−1.39686	−1.61407
H	−3.90135	−1.66129	−0.08004
H	−2.83315	−1.88555	+1.28059
H	−4.92807	+0.41883	−0.34304
H	−4.46283	+2.69146	+1.57772
H	−5.08875	+2.74637	−0.04808
H	−2.28779	+2.94106	+0.82917
H	−2.87376	+3.12877	−0.80681
H	−2.67728	+1.44773	+2.47955
H	−1.53945	+0.13232	+2.20974
H	−3.12299	−0.20576	+2.88714
H	−6.54697	+0.70673	+1.46723
H	−5.27648	+0.63793	+2.67451
H	−5.65433	−0.78674	+1.70412
H	−2.83390	+0.79820	−1.22913
O	−7.69689	−2.68364	−1.27504
H	−7.21238	−2.85656	−2.11974
C	−7.54282	−3.81717	−0.42197
H	−7.85493	−4.75765	−0.95215
H	−8.21636	−3.68135	+0.45132
C	−6.10141	−3.96046	+0.07587
H	−5.72176	−2.98537	+0.47699
O	−6.19612	−4.92532	+1.20123
C	−4.91370	−5.13837	+1.86849
O	−4.41992	−3.84718	+2.33383
H	−5.11425	−5.82172	+2.73676
C	−3.93767	−5.75303	+0.87084
O	−2.67554	−5.94944	+1.54540
H	−1.97122	−6.10700	+0.86131
H	−4.34033	−6.75240	+0.53850
C	−3.76061	−4.85022	−0.34859
O	−2.94404	−5.56292	−1.30958
H	−3.01480	−5.08297	−2.18779
H	−3.24124	−3.90089	−0.04241
C	−5.12080	−4.50533	−0.97126

H	−5.55189	−5.40957	−1.47711
O	−4.87926	−3.44836	−1.98030
C	−5.25163	−3.79364	−3.34763
O	−6.32513	−2.89795	−3.77288
C	−5.94056	−1.46373	−3.71423
C	−7.21751	−0.71658	−4.10981
O	−7.12009	+0.68000	−3.83373
H	−6.46430	+1.07777	−4.45767
H	−7.43378	−0.89088	−5.19855
H	−8.07097	−1.12248	−3.52543
H	−5.64603	−1.21901	−2.66187
H	−5.67047	−4.83483	−3.39117
C	−4.06236	−3.61949	−4.28557
O	−3.00587	−4.49621	−3.83850
H	−2.15984	−4.22435	−4.28560
H	−4.37738	−3.92029	−5.32600
C	−3.58454	−2.16848	−4.31053
O	−2.54988	−2.06945	−5.31726
H	−2.40470	−1.09690	−5.51837
H	−3.16402	−1.88546	−3.30388
C	−4.74997	−1.23108	−4.65419
H	−5.06192	−1.38187	−5.72163
O	−4.26535	+0.15681	−4.47785
C	−4.28777	+0.98079	−5.68216
O	−5.22827	+2.07911	−5.47241
C	−4.87151	+2.94416	−4.31758
C	−6.01145	+3.96530	−4.25516
O	−5.99530	+4.70630	−3.03609
H	−5.21049	+5.30738	−3.04381
H	−5.94645	+4.65595	−5.13909
H	−6.98214	+3.42772	−4.31612
H	−4.85168	+2.31177	−3.39384
H	−4.67912	+0.39127	−6.55447
C	−2.90662	+1.56150	−5.96424
O	−1.98642	+0.46766	−6.16129
H	−1.05899	+0.82276	−6.10174
H	−2.96221	+2.17255	−6.91102
C	−2.43988	+2.45992	−4.82064
O	−1.18206	+3.05408	−5.22084
H	−0.99630	+3.82865	−4.61101

H	−2.29318	+1.85008	−3.88597
C	−3.48333	+3.55350	−4.55744
H	−3.51554	+4.26991	−5.42079
O	−3.06246	+4.27704	−3.33726
C	−2.78577	+5.69807	−3.51742
O	−3.74774	+6.45692	−2.72050
C	−3.70912	+6.09245	−1.28082
C	−4.81996	+6.93326	−0.64422
O	−5.17908	+6.44294	+0.64722
H	−4.42201	+6.59466	+1.26550
H	−4.49577	+8.00798	−0.58442
H	−5.72382	+6.88817	−1.28917
H	−3.93932	+5.00047	−1.18804
H	−2.94723	+6.00028	−4.58692
C	−1.37963	+6.03527	−3.03413
O	−0.43612	+5.29725	−3.83970
H	+0.46133	+5.35974	−3.41411
H	−1.20474	+7.13990	−3.17845
C	−1.21324	+5.69018	−1.55548
O	+0.09261	+6.15934	−1.14097
H	+0.11412	+6.16228	−0.13744
H	−1.27794	+4.57467	−1.41310
C	−2.30721	+6.37247	−0.72219
H	−2.11853	+7.47780	−0.67334
O	−2.23112	+5.80168	+0.64072
C	−1.98036	+6.76103	+1.70880
O	−3.12576	+6.74377	+2.61620
C	−3.38603	+5.39731	+3.18792
C	−4.65700	+5.58254	+4.02163
O	−5.22693	+4.32986	+4.40023
H	−4.63173	+3.90452	+5.06561
H	−4.42673	+6.20295	+4.92949
H	−5.40977	+6.13259	+3.41702
H	−3.56569	+4.68218	+2.34437
H	−1.91400	+7.80404	+1.29773
C	−0.72874	+6.37030	+2.48586
O	+0.38851	+6.38912	+1.57205
H	+1.15457	+5.91453	+1.99472
H	−0.55444	+7.13175	+3.29959
C	−0.88939	+4.98970	+3.12093

O	+0.28120	+4.74918	+3.93598
H	+0.07792	+3.98859	+4.55799
H	−0.96604	+4.20279	+2.31853
C	−2.15413	+4.95531	+3.98967
H	−2.01931	+5.61371	+4.88856
O	−2.34493	+3.55585	+4.43452
C	−2.33253	+3.35676	+5.87988
O	−3.64826	+2.86941	+6.28866
C	−4.02577	+1.59407	+5.62728
C	−5.44601	+1.31923	+6.12968
O	−6.08083	+0.28482	+5.37869
H	−5.64118	−0.57349	+5.59602
H	−5.41575	+1.05399	+7.22090
H	−6.05395	+2.24309	+6.02194
H	−4.02103	+1.75340	+4.51820
H	−2.17787	+4.33440	+6.41064
C	−1.28043	+2.32555	+6.27412
O	+0.00942	+2.81520	+5.85037
H	+0.65648	+2.06005	+5.86021
H	−1.28552	+2.22008	+7.39732
C	−1.57414	+0.96454	+5.64578
O	−0.60838	+0.02499	+6.17520
H	−0.92851	−0.90132	+5.96131
H	−1.46869	+1.02797	+4.52733
C	−2.99918	+0.51509	+5.99787
H	−3.06708	+0.26902	+7.09070
O	−3.28663	−0.70165	+5.20445
C	−3.55613	−1.90758	+5.97922
O	−4.93449	−2.32103	+5.72230
H	−3.48724	−1.69363	+7.07950
C	−2.61886	−3.03391	+5.55709
O	−1.26413	−2.61038	+5.81540
H	−0.64265	−3.22311	+5.33755
H	−2.84758	−3.94535	+6.18130
C	−2.80713	−3.38148	+4.08112
O	−1.97609	−4.53077	+3.79080
H	−2.29465	−4.93545	+2.92972
H	−2.49265	−2.51180	+3.43734
C	−4.27896	−3.71115	+3.80108
H	−4.55702	−4.68132	+4.29192

C	−5.19961	−2.58213	+4.28357
H	−4.97946	−1.64654	+3.70824
C	−6.69424	−2.91183	+4.22950
H	−6.87579	−3.92504	+4.67984
H	−7.25146	−2.16626	+4.83653
O	−7.19824	−2.85672	+2.89536
H	−6.83253	−3.62395	+2.38988
O	+5.03697	−5.70643	−3.51027
H	+4.31094	−6.22411	−3.08329
C	+4.61392	−5.31462	−4.81497
H	+4.27957	−6.20857	−5.40887
H	+5.48762	−4.86906	−5.33747
C	+3.48406	−4.28148	−4.76225
H	+3.74144	−3.44618	−4.06282
O	+3.42710	−3.73576	−6.14356
C	+2.42916	−2.67975	−6.29935
O	+2.74143	−1.60814	−5.35985
H	+2.51437	−2.31452	−7.35792
C	+1.05092	−3.26003	−6.00421
O	+0.07516	−2.21011	−6.17091
H	−0.78223	−2.50823	−5.76356
H	+0.83945	−4.08381	−6.74498
C	+0.99333	−3.83199	−4.58930
O	−0.29901	−4.46344	−4.42410
H	−0.25378	−5.05506	−3.61489
H	+1.11068	−3.00346	−3.83640
C	+2.11301	−4.86208	−4.38994
H	+1.89990	−5.78322	−4.99469
O	+2.12775	−5.21217	−2.95269
C	+1.88904	−6.61747	−2.64596
O	+3.08370	−7.15312	−1.99710
C	+3.45574	−6.39623	−0.77377
C	+4.75416	−7.05552	−0.29897
O	+5.44079	−6.24472	+0.65348
H	+4.91356	−6.22334	+1.48986
H	+4.52864	−8.06895	+0.13174
H	+5.42660	−7.20048	−1.17164
H	+3.63587	−5.32930	−1.06084
H	+1.74180	−7.21035	−3.58822
C	+0.71056	−6.75618	−1.68915

O	−0.46672	−6.24006	−2.34633
H	−1.18665	−6.13310	−1.66774
H	+0.55676	−7.84919	−1.45880
C	+0.97877	−6.00160	−0.38814
O	−0.12264	−6.27490	+0.51081
H	+0.16310	−6.01899	+1.43841
H	+1.03696	−4.89595	−0.59273
C	+2.30038	−6.47275	+0.23392
H	+2.18917	−7.51998	+0.62196
O	+2.59910	−5.56219	+1.36130
C	+2.71830	−6.20267	+2.66617
O	+4.07802	−5.99376	+3.15746
C	+4.43797	−4.55624	+3.26727
C	+5.90275	−4.56758	+3.71417
O	+6.50492	−3.28100	+3.58358
H	+6.10346	−2.67615	+4.25482
H	+5.97008	−4.93135	+4.77534
H	+6.47063	−5.27807	+3.07576
H	+4.33449	−4.08698	+2.25565
H	+2.57854	−7.31333	+2.57596
C	+1.73678	−5.58122	+3.65291
O	+0.39932	−5.79348	+3.15257
H	−0.22199	−5.19388	+3.64659
H	+1.84611	−6.10122	+4.64777
C	+2.01595	−4.09062	+3.83694
O	+1.12450	−3.60783	+4.86934
H	+1.46596	−2.71814	+5.18465
H	+1.81836	−3.53824	+2.87481
C	+3.47758	−3.87779	+4.25365
H	+3.64122	−4.27498	+5.29052
O	+3.73276	−2.41994	+4.24254
C	+4.11912	−1.84954	+5.53010
O	+5.47733	−1.32645	+5.41618
C	+5.61775	−0.26056	+4.38927
C	+7.10890	+0.08894	+4.42657
O	+7.49908	+0.88551	+3.31043
H	+7.07990	+1.77633	+3.39574
H	+7.34632	+0.61452	+5.39170
H	+7.70096	−0.85118	+4.39949
H	+5.33156	−0.68563	+3.39312

H	+4.15386	−2.64937	+6.31815
C	+3.18170	−0.71269	+5.91926
O	+1.84403	−1.24406	+6.02342
H	+1.20380	−0.48350	+6.06421
H	+3.49986	−0.31622	+6.92648
C	+3.23567	+0.42358	+4.89993
O	+2.40013	+1.49289	+5.40472
H	+2.63048	+2.33150	+4.90346
H	+2.84472	+0.07135	+3.90495
C	+4.68398	+0.90579	+4.74159
H	+5.02608	+1.40723	+5.68594
O	+4.70878	+1.88452	+3.63246
C	+5.14253	+3.22890	+3.99558
O	+6.37392	+3.52804	+3.26913
C	+6.22266	+3.43324	+1.79321
C	+7.62544	+3.75009	+1.26388
O	+7.78932	+3.35807	−0.09720
H	+7.18757	+3.90342	−0.66125
H	+7.83399	+4.84879	+1.38843
H	+8.37325	+3.19464	+1.86963
H	+5.92319	+2.38626	+1.53253
H	+5.39704	+3.28085	+5.08833
C	+4.08472	+4.25541	+3.60640
O	+2.88160	+3.96421	+4.34756
H	+2.12260	+4.45241	+3.92868
H	+4.45570	+5.28212	+3.89065
C	+3.82295	+4.21491	+2.10302
O	+2.90272	+5.28690	+1.78201
H	+2.93626	+5.43387	+0.79053
H	+3.36811	+3.22604	+1.81839
C	+5.13347	+4.41212	+1.33040
H	+5.48669	+5.47248	+1.43446
O	+4.81960	+4.13886	−0.08936
C	+5.11805	+5.21163	−1.02763
O	+6.13491	+4.72277	−1.96031
C	+5.69616	+3.46205	−2.60768
C	+6.91210	+2.93599	−3.36842
O	+6.68331	+1.58532	−3.79209
H	+6.08849	+1.61498	−4.58218
H	+7.13670	+3.58863	−4.25111

H	+7.79764	+2.94179	−2.69870
H	+5.41333	+2.73855	−1.80212
H	+5.56115	+6.09575	−0.49609
C	+3.86721	+5.58283	−1.81992
O	+2.86361	+6.02524	−0.87848
H	+1.98013	+6.02650	−1.33406
H	+4.11933	+6.43040	−2.51940
C	+3.34939	+4.39651	−2.63469
O	+2.29056	+4.88393	−3.49363
H	+2.08770	+4.16648	−4.16433
H	+2.93958	+3.61227	−1.93902
C	+4.47287	+3.77593	−3.47827
H	+4.75703	+4.46697	−4.31514
O	+3.95215	+2.50914	−4.04556
C	+3.89201	+2.46587	−5.50268
O	+4.82933	+1.44797	−5.97662
H	+4.24426	+3.43978	−5.93748
C	+2.48578	+2.11531	−5.97641
O	+1.57825	+3.11592	−5.46955
H	+0.64890	+2.77180	−5.55602
H	+2.47251	+2.14116	−7.10462
C	+2.06915	+0.72171	−5.51002
O	+0.77823	+0.43455	−6.09814
H	+0.61572	−0.55272	−6.02388
H	+1.99144	+0.69637	−4.38766
C	+3.10266	−0.31194	−5.97524
H	+3.07252	−0.41007	−7.09279
C	+4.51225	+0.07316	−5.50961
H	+4.55252	+0.05831	−4.38975
C	+5.63367	−0.78311	−6.10741
H	+5.50076	−0.85113	−7.22097
H	+6.60948	−0.28718	−5.91342
O	+5.67828	−2.08376	−5.52515
H	+4.88561	−2.59117	−5.82794

345

* E = 2.959 kcal/mol

C	+0.67593	−0.72324	−0.77332
C	+0.94892	−0.89214	+0.59894
C	−0.04625	−0.82100	+1.56489
C	−1.41281	−0.54663	+1.46636

C	−2.20882	+0.12251	+0.53685
C	−1.81272	+1.08753	−0.39745
C	−0.52093	+1.60890	−0.45293
H	+0.26163	+0.54279	−0.83449
C	−2.86529	+1.50789	−1.38019
C	−3.63041	+0.30140	−1.90436
C	−4.24834	−0.52740	−0.77071
C	−3.69395	−0.10860	+0.58266
C	+1.73135	−0.93976	−1.84081
C	+3.12117	−1.30530	−1.32094
C	+3.39312	−0.65060	+0.01581
C	+2.36177	−1.18267	+1.00701
C	+4.68642	−1.08138	+0.71326
C	+4.41621	−0.74669	+2.18966
C	+2.88584	−0.72168	+2.36001
O	−5.64282	−0.36066	−0.66909
H	−6.10080	−0.87196	−1.34057
C	+3.33012	+0.86660	−0.10314
C	+5.97236	−0.49302	+0.17738
H	−4.01330	−1.58203	−0.93302
H	−3.96242	−0.85469	+1.32657
H	−4.21598	+0.81287	+0.84960
H	−2.94443	−0.31981	−2.48178
H	−4.42450	+0.61565	−2.57907
H	−3.56477	+2.18946	−0.88965
H	−2.41400	+2.06887	−2.19726
H	+0.03336	+1.65355	+0.48148
H	−0.34023	+2.43355	−1.13230
H	−1.97958	−0.94821	+2.30039
H	+0.26285	−1.13397	+2.55605
H	−0.33134	−1.02707	−1.05929
H	+1.80064	−0.03011	−2.44104
H	+1.38896	−1.71267	−2.52931
H	+3.19462	−2.38736	−1.19024
H	+3.87318	−1.02652	−2.06058
H	+4.73851	−2.16876	+0.61156
H	+4.84662	+0.22528	+2.43226
H	+4.89095	−1.46702	+2.85060
H	+2.52455	+0.28375	+2.57960
H	+2.54894	−1.35828	+3.17549

H	+3.58972	+1.35422	+0.83546
H	+2.33909	+1.21382	−0.38806
H	+4.03380	+1.21750	−0.85675
H	+6.83007	−0.91612	+0.69448
H	+6.00883	+0.58687	+0.31965
H	+6.09584	−0.69881	−0.88501
H	+2.47571	−2.27394	+0.97882
O	−7.25966	−2.51922	−1.84103
H	−6.78603	−3.09788	−2.48917
C	−7.48992	−3.28835	−0.65226
H	−7.87531	−4.30644	−0.91865
H	−8.26835	−2.77035	−0.05340
C	−6.21038	−3.40518	+0.17611
H	−5.79686	−2.38624	+0.39003
O	−6.63600	−4.01708	+1.46119
C	−5.54728	−4.03695	+2.43870
O	−5.08733	−2.66854	+2.62995
H	−5.97789	−4.44797	+3.39099
C	−4.41729	−4.90984	+1.90001
O	−3.33547	−4.88616	+2.85626
H	−2.51531	−5.22977	+2.41134
H	−4.79789	−5.96700	+1.79843
C	−3.93583	−4.42436	+0.53223
O	−2.98510	−5.39767	+0.03845
H	−2.85977	−5.23927	−0.94365
H	−3.43591	−3.42051	+0.63374
C	−5.12370	−4.30240	−0.43221
H	−5.54146	−5.31815	−0.66231
O	−4.62003	−3.69040	−1.68458
C	−4.74935	−4.51851	−2.87843
O	−5.68014	−3.86247	−3.79794
C	−5.23413	−2.51022	−4.22240
C	−6.36442	−2.00977	−5.12758
O	−6.27807	−0.60356	−5.35127
H	−5.49812	−0.42363	−5.93224
H	−6.34411	−2.56865	−6.10175
H	−7.34019	−2.21912	−4.63790
H	−5.14109	−1.86465	−3.31069
H	−5.20789	−5.51077	−2.62142
C	−3.40070	−4.67820	−3.57197

O	−2.49351	−5.32183	−2.65432
H	−1.56646	−5.21129	−2.99861
H	−3.53983	−5.33551	−4.47849
C	−2.84739	−3.32661	−4.01895
O	−1.62705	−3.57352	−4.75665
H	−1.40576	−2.74474	−5.27719
H	−2.62701	−2.68369	−3.12058
C	−3.86909	−2.61465	−4.91384
H	−3.96502	−3.15298	−5.89374
O	−3.36112	−1.24655	−5.15275
C	−3.12038	−0.90319	−6.55000
O	−4.01700	+0.19170	−6.91249
C	−3.84480	+1.38572	−6.04472
C	−4.90251	+2.37303	−6.54770
O	−5.11764	+3.43623	−5.62117
H	−4.30951	+4.00569	−5.59897
H	−4.59562	+2.77665	−7.55082
H	−5.86604	+1.83529	−6.67997
H	−4.05382	+1.08569	−4.98589
H	−3.38003	−1.76576	−7.22048
C	−1.68038	−0.44132	−6.73897
O	−0.80643	−1.53713	−6.39358
H	+0.12211	−1.18724	−6.31702
H	−1.52518	−0.17109	−7.82259
C	−1.38592	+0.77914	−5.86906
O	−0.04599	+1.22694	−6.18445
H	+0.06454	+2.15490	−5.81838
H	−1.44668	+0.49668	−4.78085
C	−2.40087	+1.89283	−6.16131
H	−2.21758	+2.32078	−7.18255
O	−2.19574	+2.94669	−5.14407
C	−1.82961	+4.25789	−5.66668
O	−2.88517	+5.20330	−5.31191
C	−3.11947	+5.28076	−3.84639
C	−4.29209	+6.25570	−3.70389
O	−4.86475	+6.20775	−2.39822
H	−4.21649	+6.58755	−1.75532
H	−3.94834	+7.29746	−3.94845
H	−5.08272	+5.97990	−4.43450
H	−3.40324	+4.26144	−3.47978

H	−1.78086	+4.23826	−6.78846
C	−0.52034	+4.72616	−5.04316
O	+0.51400	+3.78912	−5.41187
H	+1.31121	+3.95349	−4.83979
H	−0.26356	+5.74266	−5.45857
C	−0.64819	+4.82482	−3.52399
O	+0.59099	+5.37598	−3.02026
H	+0.43188	+5.69664	−2.08223
H	−0.81717	+3.80119	−3.08341
C	−1.82657	+5.73703	−3.15623
H	−1.59006	+6.79846	−3.43412
O	−2.01445	+5.64478	−1.69178
C	−1.87038	+6.90197	−0.96526
O	−3.15048	+7.22115	−0.33862
C	−3.61634	+6.16899	+0.60247
C	−4.98254	+6.67226	+1.07827
O	−5.72263	+5.64975	+1.74261
H	−5.28032	+5.45044	+2.60414
H	−4.84295	+7.56177	+1.75103
H	−5.57261	+7.00404	+0.19690
H	−3.72122	+5.20909	+0.03591
H	−1.64500	+7.74336	−1.67444
C	−0.80778	+6.77168	+0.11965
O	+0.44842	+6.45408	−0.51651
H	+1.08590	+6.13746	+0.17856
H	−0.71232	+7.76237	+0.65069
C	−1.18875	+5.69713	+1.13668
O	−0.18763	+5.71865	+2.18181
H	−0.55974	+5.23752	+2.98035
H	−1.20272	+4.68576	+0.64298
C	−2.57586	+6.00183	+1.71853
H	−2.53059	+6.92891	+2.34984
O	−2.96521	+4.85281	+2.56529
C	−3.21074	+5.16907	+3.96834
O	−4.61043	+4.87971	+4.26945
C	−4.98087	+3.46085	+4.02639
C	−6.47688	+3.40933	+4.35218
O	−7.08393	+2.20628	+3.88532
H	−6.73312	+1.44851	+4.41414
H	−6.62376	+3.52343	+5.46099

H	−6.98468	+4.26348	+3.85459
H	−4.79898	+3.22710	+2.94690
H	−3.06670	+6.26726	+4.15556
C	−2.32603	+4.31814	+4.87214
O	−0.94859	+4.61678	+4.56209
H	−0.37188	+3.91264	+4.96406
H	−2.53254	+4.59901	+5.94510
C	−2.61594	+2.83044	+4.68265
O	−1.82444	+2.10644	+5.65551
H	−2.19147	+1.17504	+5.72373
H	−2.32779	+2.51050	+3.64339
C	−4.10888	+2.55504	+4.90697
H	−4.36688	+2.69800	+5.99005
O	−4.35561	+1.14629	+4.52773
C	−4.86575	+0.28992	+5.59190
O	−6.19421	−0.18019	+5.20586
H	−4.99648	+0.87346	+6.54260
C	−3.95921	−0.92114	+5.78187
O	−2.65392	−0.45126	+6.18297
H	−2.00126	−1.19190	+6.06416
H	−4.38684	−1.57003	+6.59902
C	−3.86745	−1.72894	+4.49019
O	−3.09576	−2.92311	+4.76657
H	−3.22680	−3.55587	+3.99916
H	−3.35028	−1.11862	+3.69803
C	−5.26612	−2.11569	+3.99193
H	−5.70778	−2.90439	+4.65737
C	−6.20030	−0.90012	+3.90508
H	−5.83571	−0.20088	+3.11092
C	−7.67481	−1.25424	+3.68766
H	−7.97221	−2.07481	+4.39810
H	−8.29923	−0.36506	+3.92035
O	−7.94222	−1.63449	+2.33938
H	−7.43537	−2.45832	+2.13465
O	+5.23182	−6.08017	−0.35003
H	+4.41821	−6.45582	+0.06799
C	+5.12582	−6.23675	−1.76451
H	+4.90259	−7.30565	−2.02902
H	+6.10801	−5.97436	−2.21299
C	+4.04698	−5.32498	−2.35564

H	+4.18778	−4.26755	−2.01295
O	+4.28511	−5.37934	−3.82169
C	+3.40013	−4.49246	−4.57433
O	+3.60598	−3.12997	−4.09822
H	+3.70004	−4.58327	−5.65294
C	+1.95636	−4.92613	−4.34551
O	+1.09355	−4.02805	−5.07668
H	+0.16429	−4.15030	−4.74402
H	+1.82693	−5.97196	−4.74774
C	+1.60394	−4.91751	−2.85870
O	+0.26952	−5.46197	−2.72570
H	+0.12973	−5.70722	−1.76261
H	+1.62580	−3.86230	−2.46528
C	+2.60652	−5.77365	−2.07383
H	+2.47806	−6.85623	−2.34050
O	+2.31426	−5.58726	−0.63429
C	+1.94276	−6.80170	+0.08520
O	+2.96228	−7.06765	+1.09754
C	+3.10387	−5.97017	+2.08969
C	+4.25679	−6.42933	+2.98748
O	+4.73120	−5.37516	+3.82376
H	+4.03965	−5.17769	+4.50214
H	+3.92539	−7.30672	+3.60618
H	+5.10125	−6.76552	+2.34807
H	+3.37392	−5.03072	+1.54321
H	+1.94875	−7.68539	−0.60829
C	+0.59297	−6.63506	+0.77488
O	−0.40051	−6.35589	−0.23345
H	−1.22548	−6.03230	+0.21954
H	+0.33437	−7.60578	+1.28871
C	+0.63129	−5.51917	+1.81749
O	−0.64658	−5.51894	+2.49796
H	−0.54773	−4.98925	+3.34427
H	+0.79603	−4.52563	+1.31500
C	+1.76576	−5.77981	+2.81699
H	+1.52933	−6.68275	+3.44027
O	+1.86203	−4.59215	+3.69413
C	+1.64002	−4.84480	+5.11354
O	+2.87221	−4.52941	+5.83354
C	+3.31695	−3.12771	+5.62444

C	+4.63491	−3.03110	+6.39869
O	+5.37517	−1.86464	+6.04057
H	+4.88852	−1.06834	+6.36751
H	+4.42652	−3.04453	+7.50304
H	+5.26061	−3.91834	+6.16165
H	+3.48904	−2.97079	+4.52870
H	+1.43479	−5.93408	+5.29413
C	+0.52174	−3.95679	+5.64773
O	−0.69233	−4.28560	+4.94001
H	−1.36526	−3.57450	+5.11799
H	+0.38149	−4.17544	+6.74525
C	+0.86582	−2.47929	+5.47137
O	−0.17693	−1.70727	+6.11310
H	+0.14853	−0.76178	+6.20008
H	+0.90663	−2.22414	+4.37446
C	+2.22450	−2.16744	+6.11460
H	+2.13950	−2.20621	+7.23298
O	+2.59538	−0.79735	+5.69785
C	+2.76247	+0.16789	+6.77725
O	+4.15221	+0.62102	+6.77281
C	+4.55529	+1.20008	+5.46501
C	+6.04206	+1.52533	+5.63352
O	+6.67543	+1.75658	+4.37505
H	+6.32888	+2.60402	+4.00052
H	+6.16250	+2.41674	+6.30720
H	+6.54950	+0.66357	+6.11791
H	+4.40926	+0.42029	+4.67523
H	+2.58510	−0.31652	+7.77472
C	+1.85517	+1.37192	+6.55210
O	+0.48715	+0.91122	+6.57182
H	−0.09479	+1.62260	+6.19037
H	+2.01115	+2.10631	+7.39372
C	+2.17881	+2.05508	+5.22424
O	+1.37466	+3.25565	+5.14405
H	+1.75409	+3.83387	+4.41734
H	+1.92518	+1.36900	+4.36807
C	+3.66940	+2.41581	+5.16034
H	+3.89267	+3.25098	+5.87610
O	+3.94812	+2.86945	+3.77804
C	+4.46777	+4.22663	+3.66089

O	+5.81085	+4.15613	+3.08827
C	+5.83499	+3.51516	+1.74846
C	+7.32212	+3.44329	+1.39213
O	+7.54993	+2.60927	+0.25599
H	+7.21460	+3.08070	−0.54560
H	+7.71785	+4.47793	+1.20803
H	+7.88065	+3.01358	+2.25120
H	+5.39905	+2.48602	+1.83819
H	+4.58032	+4.69442	+4.67581
C	+3.58173	+5.06567	+2.74600
O	+2.25459	+5.09356	+3.31249
H	+1.61810	+5.41877	+2.62069
H	+3.99247	+6.11612	+2.71448
C	+3.55287	+4.50399	+1.32467
O	+2.80156	+5.43394	+0.50935
H	+2.99526	+5.23123	−0.45373
H	+3.04814	+3.49744	+1.31830
C	+4.98399	+4.35812	+0.78870
H	+5.44671	+5.37057	+0.64603
O	+4.90429	+3.66994	−0.52029
C	+5.41526	+4.42958	−1.65645
O	+6.57797	+3.72953	−2.19930
C	+6.27195	+2.35128	−2.66239
C	+7.62623	+1.80591	−3.12620
O	+7.59492	+0.39049	−3.30459
H	+7.03627	+0.18457	−4.09388
H	+7.93388	+2.31818	−4.07778
H	+8.39226	+2.03619	−2.35475
H	+5.88443	+1.76196	−1.79166
H	+5.78151	+5.43744	−1.32234
C	+4.35571	+4.54637	−2.74729
O	+3.21374	+5.22832	−2.18812
H	+2.43506	+5.09979	−2.79335
H	+4.77789	+5.16282	−3.59261
C	+3.95790	+3.17280	−3.28509
O	+3.04954	+3.38268	−4.39323
H	+2.98347	+2.52372	−4.90762
H	+3.44241	+2.57936	−2.47933
C	+5.20140	+2.40942	−3.76048
H	+5.61516	+2.88549	−4.68874

O	+4.77074	+1.02708	−4.06944
C	+4.97547	+0.59553	−5.44755
O	+5.92624	−0.51511	−5.44478
H	+5.44230	+1.41629	−6.05530
C	+3.66161	+0.10918	−6.04980
O	+2.73380	+1.21678	−6.05645
H	+1.81637	+0.85913	−6.19319
H	+3.84839	−0.22525	−7.11010
C	+3.09852	−1.05672	−5.24035
O	+1.92729	−1.55476	−5.93223
H	+1.69379	−2.44606	−5.53472
H	+2.80746	−0.69349	−4.21684
C	+4.13825	−2.17689	−5.09923
H	+4.27238	−2.70468	−6.08049
C	+5.48334	−1.64412	−4.58677
H	+5.36155	−1.26341	−3.54004
C	+6.63085	−2.65550	−4.66281
H	+6.63505	−3.14859	−5.67303
H	+7.59666	−2.11899	−4.54416
O	+6.54228	−3.63211	−3.62583
H	+5.74582	−4.19590	−3.78776

345

* E = 3.365 kcal/mol

C	−0.71032	+0.95182	+0.75809
C	−1.30543	−0.32402	+0.80480
C	−0.55244	−1.48861	+0.87935
C	+0.83014	−1.69954	+0.86914
C	+1.91854	−0.96469	+0.40315
C	+1.91933	+0.03571	−0.57652
C	+0.79248	+0.36576	−1.32505
H	−0.06727	+0.92170	−0.41006
C	+3.21680	+0.76008	−0.76251
C	+4.36113	−0.20584	−1.07406
C	+4.24628	−1.50995	−0.29620
C	+3.29204	−1.35664	+0.88048
C	−1.52313	+2.22885	+0.83060
C	−3.03986	+2.04073	+0.85925
C	−3.45282	+0.80157	+0.09587
C	−2.80235	−0.39976	+0.77887
C	−4.92917	+0.41369	+0.21797

C	−4.92781	−1.08419	−0.12894
C	−3.50330	−1.59691	+0.15242
O	+3.75585	−2.47734	−1.20183
H	+3.60188	−3.30229	−0.73838
C	−3.04810	+0.90960	−1.36907
C	−5.91180	+1.23092	−0.59035
H	+5.22684	−1.81752	+0.06703
H	+3.69499	−0.59717	+1.55631
H	+3.24549	−2.28033	+1.45976
H	+5.30936	+0.27968	−0.85881
H	+4.36019	−0.46364	−2.13090
H	+3.12910	+1.50742	−1.54867
H	+3.45901	+1.30068	+0.15655
H	+0.06647	−0.42515	−1.49663
H	+0.92123	+1.05719	−2.14874
H	+1.11279	−2.64353	+1.32532
H	−1.12712	−2.38780	+1.07244
H	+0.25909	+1.00273	+1.25372
H	−1.25260	+2.85350	−0.02322
H	−1.21391	+2.79319	+1.71080
H	−3.37862	+1.93064	+1.89193
H	−3.52685	+2.93254	+0.46226
H	−5.18436	+0.52026	+1.27576
H	−5.18566	−1.22042	−1.17931
H	−5.67804	−1.62272	+0.44400
H	−2.99540	−1.89416	−0.76601
H	−3.49749	−2.46621	+0.80639
H	−3.39414	+0.05271	−1.94555
H	−1.96913	+0.97089	−1.49182
H	−3.48263	+1.80140	−1.81917
H	−6.93153	+0.91563	−0.38305
H	−5.74712	+1.11337	−1.66104
H	−5.84184	+2.29139	−0.35259
H	−3.13676	−0.34857	+1.82322
O	−6.92093	−4.66666	−0.01959
H	−6.34894	−5.00249	−0.75307
C	−6.53163	−5.30589	+1.19565
H	−6.56891	−6.42402	+1.08519
H	−7.26381	−5.01928	+1.98099
C	−5.12933	−4.87772	+1.63864

H	−5.02737	−3.76297	+1.60070
O	−5.04549	−5.30656	+3.05838
C	−3.77604	−4.94387	+3.68568
O	−3.60984	−3.49758	+3.58444
H	−3.84996	−5.25906	+4.76104
C	−2.64971	−5.66619	+2.95465
O	−1.40130	−5.32513	+3.59405
H	−0.65684	−5.59512	+2.99261
H	−2.81876	−6.77778	+3.04197
C	−2.62140	−5.27621	+1.47885
O	−1.61301	−6.08662	+0.82818
H	−1.73977	−5.99581	−0.16367
H	−2.35386	−4.18639	+1.37845
C	−3.99264	−5.52405	+0.83455
H	−4.16957	−6.62634	+0.71874
O	−3.96204	−4.88867	−0.50070
C	−4.15579	−5.78575	−1.63258
O	−5.37651	−5.38226	−2.32866
C	−5.33688	−3.96215	−2.76433
C	−6.71561	−3.71196	−3.38157
O	−6.98138	−2.31648	−3.52532
H	−6.38278	−1.95297	−4.22395
H	−6.78852	−4.23338	−4.37441
H	−7.49470	−4.14104	−2.71547
H	−5.18850	−3.32043	−1.85887
H	−4.31854	−6.84007	−1.28180
C	−2.98274	−5.67304	−2.59905
O	−1.78654	−6.08985	−1.90640
H	−0.99653	−5.79903	−2.43753
H	−3.16421	−6.36288	−3.47264
C	−2.84060	−4.24078	−3.11186
O	−1.79269	−4.23745	−4.10935
H	−1.86882	−3.38744	−4.63675
H	−2.56220	−3.55485	−2.26279
C	−4.16201	−3.76789	−3.73277
H	−4.35266	−4.31684	−4.69298
O	−4.01637	−2.32195	−4.01732
C	−4.19464	−1.94122	−5.41347
O	−5.34802	−1.04748	−5.50058
C	−5.18982	+0.18010	−4.67945

C	−6.52920	+0.90701	−4.82493
O	−6.65539	+1.96826	−3.87818
H	−6.03938	+2.69438	−4.14392
H	−6.63680	+1.29737	−5.87238
H	−7.35506	+0.18587	−4.64447
H	−5.01851	−0.12407	−3.61423
H	−4.44240	−2.84060	−6.03893
C	−2.96145	−1.21055	−5.93402
O	−1.83051	−2.09898	−5.81485
H	−0.99463	−1.56992	−5.92074
H	−3.12437	−0.96352	−7.02284
C	−2.71493	+0.08697	−5.16471
O	−1.61157	+0.76482	−5.80986
H	−1.60672	+1.71853	−5.49947
H	−2.44878	−0.14306	−4.09507
C	−3.97537	+0.96262	−5.19791
H	−4.16432	+1.32533	−6.24303
O	−3.73693	+2.12693	−4.31397
C	−3.78939	+3.42850	−4.97123
O	−4.91054	+4.18466	−4.41640
C	−4.79833	+4.42319	−2.95405
C	−6.08961	+5.16815	−2.60163
O	−6.30661	+5.21721	−1.19219
H	−5.64176	+5.83005	−0.79264
H	−6.05670	+6.20485	−3.03322
H	−6.95022	+4.63670	−3.06200
H	−4.75698	+3.43141	−2.43366
H	−4.00376	+3.30644	−6.06700
C	−2.49982	+4.20662	−4.73140
O	−1.40632	+3.43655	−5.27148
H	−0.55081	+3.80070	−4.91813
H	−2.56734	+5.18967	−5.28123
C	−2.28709	+4.48536	−3.24430
O	−1.10950	+5.31839	−3.12082
H	−1.11223	+5.73131	−2.20633
H	−2.12954	+3.51843	−2.68891
C	−3.51112	+5.20845	−2.66725
H	−3.58140	+6.24601	−3.08921
O	−3.32048	+5.28828	−1.20168
C	−3.27652	+6.63583	−0.64373

O	−4.41230	+6.79475	+0.26291
C	−4.43934	+5.76093	+1.32937
C	−5.71545	+6.06022	+2.12151
O	−6.09810	+4.95646	+2.94151
H	−5.41587	+4.83165	+3.64685
H	−5.56733	+6.98469	+2.74402
H	−6.54612	+6.26056	+1.41113
H	−4.50556	+4.75496	+0.84099
H	−3.40058	+7.40471	−1.45261
C	−1.99072	+6.83687	+0.15126
O	−0.87426	+6.69661	−0.75555
H	−0.04884	+6.55635	−0.21989
H	−1.98863	+7.87713	+0.58534
C	−1.89503	+5.81155	+1.27801
O	−0.72617	+6.12760	+2.07348
H	−0.78190	+5.59320	+2.92096
H	−1.78365	+4.78515	+0.83414
C	−3.15111	+5.84241	+2.15971
H	−3.15152	+6.76428	+2.79965
O	−3.09048	+4.64277	+3.02614
C	−3.06376	+4.90107	+4.46016
O	−4.27044	+4.32469	+5.05071
C	−4.39007	+2.86436	+4.80202
C	−5.74834	+2.48679	+5.39938
O	−6.16297	+1.18619	+4.98231
H	−5.57739	+0.51778	+5.41640
H	−5.69840	+2.54608	+6.52018
H	−6.51174	+3.21685	+5.05458
H	−4.38203	+2.69229	+3.69474
H	−3.10409	+6.00483	+4.66415
C	−1.84202	+4.24647	+5.09521
O	−0.66077	+4.81160	+4.48618
H	+0.11686	+4.23775	+4.72232
H	−1.83849	+4.48312	+6.19805
C	−1.86897	+2.72936	+4.91145
O	−0.75128	+2.18359	+5.65120
H	−0.91054	+1.20029	+5.77296
H	−1.76528	+2.47312	+3.81955
C	−3.18907	+2.15781	+5.44553
H	−3.22908	+2.26542	+6.56198

O	−3.22504	+0.71991	+5.09259
C	−3.32509	−0.19277	+6.22733
O	−4.59017	−0.91691	+6.12801
H	−3.36816	+0.38395	+7.19040
C	−2.17660	−1.19607	+6.22407
O	−0.93589	−0.46336	+6.29294
H	−0.19227	−1.07876	+6.05179
H	−2.27181	−1.84729	+7.14086
C	−2.21035	−2.08628	+4.98293
O	−1.16393	−3.07536	+5.13370
H	−1.33832	−3.81599	+4.47993
H	−2.02424	−1.47161	+4.05869
C	−3.57846	−2.77211	+4.87396
H	−3.70974	−3.50471	+5.71403
C	−4.70857	−1.73374	+4.89216
H	−4.61271	−1.05477	+4.00640
C	−6.11843	−2.32621	+4.97762
H	−6.16394	−3.06564	+5.82207
H	−6.84002	−1.51129	+5.20157
O	−6.51347	−2.93948	+3.75147
H	−5.98476	−3.76576	+3.63038
O	+6.29149	−5.32713	−1.46165
H	+5.68523	−5.82526	−0.86097
C	+5.86197	−5.52618	−2.80956
H	+5.76833	−6.62337	−3.03481
H	+6.64268	−5.10837	−3.48072
C	+4.53127	−4.82372	−3.09317
H	+4.56246	−3.75424	−2.76062
O	+4.41540	−4.85513	−4.57636
C	+3.22171	−4.16714	−5.06638
O	+3.25929	−2.78998	−4.59565
H	+3.26921	−4.20544	−6.18828
C	+1.99000	−4.88107	−4.52081
O	+0.81680	−4.20360	−5.01924
H	+0.03383	−4.48800	−4.47592
H	+1.99101	−5.94430	−4.89838
C	+2.00107	−4.89278	−2.99339
O	+0.86670	−5.68202	−2.55619
H	+1.00747	−5.92116	−1.59306
H	+1.91145	−3.84432	−2.59870

C	+3.30527	−5.52004	−2.48468
H	+3.32107	−6.61901	−2.71258
O	+3.32875	−5.33356	−1.01107
C	+3.38991	−6.55772	−0.21858
O	+4.64118	−6.54948	+0.53715
C	+4.77962	−5.35158	+1.40312
C	+6.16879	−5.49225	+2.03210
O	+6.59610	−4.27315	+2.63931
H	+6.03570	−4.10383	+3.43650
H	+6.16134	−6.32793	+2.78297
H	+6.90124	−5.75446	+1.23895
H	+4.73300	−4.43870	+0.75430
H	+3.42377	−7.45918	−0.88644
C	+2.22778	−6.61340	+0.76710
O	+0.99558	−6.62956	+0.01610
H	+0.24517	−6.42805	+0.63691
H	+2.30933	−7.56460	+1.36734
C	+2.26160	−5.41696	+1.71616
O	+1.21312	−5.60583	+2.69505
H	+1.36543	−4.95086	+3.44007
H	+2.07615	−4.46696	+1.13896
C	+3.62535	−5.32285	+2.41442
H	+3.73581	−6.15667	+3.15688
O	+3.66032	−4.02464	+3.12639
C	+3.81418	−4.11309	+4.57547
O	+5.08237	−3.48573	+4.93969
C	+5.16114	−2.05886	+4.53222
C	+6.57935	−1.63395	+4.92394
O	+6.93537	−0.38129	+4.34026
H	+6.40944	+0.33031	+4.78147
H	+6.66386	−1.58754	+6.04314
H	+7.29958	−2.39826	+4.56075
H	+5.02073	−1.99952	+3.42241
H	+3.88672	−5.18645	+4.89775
C	+2.67853	−3.38284	+5.28350
O	+1.43339	−4.00377	+4.90094
H	+0.68658	−3.39765	+5.15610
H	+2.82095	−3.49577	+6.39701
C	+2.67036	−1.89552	+4.93378
O	+1.64985	−1.26770	+5.74520

H	+1.81153	−0.27746	+5.73926
H	+2.43071	−1.75642	+3.84266
C	+4.04270	−1.28049	+5.23836
H	+4.21943	−1.26653	+6.34654
O	+4.02739	+0.10934	+4.72913
C	+4.24711	+1.14228	+5.73626
O	+5.48471	+1.84736	+5.40887
C	+5.45658	+2.47563	+4.06277
C	+6.85255	+3.08597	+3.90527
O	+7.11696	+3.46274	+2.55468
H	+6.54762	+4.23807	+2.32620
H	+6.95712	+3.97000	+4.59093
H	+7.61269	+2.33292	+4.20523
H	+5.28557	+1.67273	+3.30124
H	+4.40350	+0.67974	+6.74761
C	+3.09727	+2.14383	+5.74085
O	+1.88149	+1.43747	+6.06400
H	+1.10820	+2.01643	+5.82580
H	+3.29890	+2.91726	+6.53704
C	+2.97222	+2.84271	+4.38872
O	+1.94375	+3.85408	+4.51244
H	+2.03543	+4.48682	+3.73940
H	+2.68019	+2.09788	+3.59680
C	+4.30860	+3.49273	+4.00807
H	+4.52199	+4.35983	+4.68785
O	+4.17379	+3.98187	+2.61849
C	+4.34942	+5.41876	+2.44052
O	+5.51603	+5.63396	+1.58727
C	+5.39682	+4.94961	+0.27400
C	+6.72845	+5.23818	−0.42562
O	+6.93227	+4.38172	−1.54865
H	+6.28108	+4.62293	−2.25276
H	+6.76570	+6.31724	−0.73780
H	+7.55943	+5.06444	+0.29144
H	+5.27643	+3.85186	+0.45840
H	+4.57363	+5.91578	+3.42219
C	+3.12841	+6.02267	+1.75640
O	+1.98412	+5.82672	+2.61432
H	+1.16208	+6.03565	+2.09417
H	+3.30154	+7.12815	+1.61739

C	+2.89679	+5.37501	+0.39262
O	+1.79757	+6.07245	−0.24083
H	+1.79815	+5.82980	−1.21470
H	+2.63155	+4.28893	+0.52564
C	+4.16388	+5.48561	−0.46674
H	+4.32591	+6.55221	−0.77609
O	+3.94809	+4.65337	−1.67055
C	+4.00294	+5.36632	−2.94139
O	+5.11854	+4.83207	−3.71868
C	+5.00347	+3.37053	−3.96240
C	+6.29007	+3.01130	−4.71144
O	+6.50142	+1.60092	−4.75246
H	+5.82054	+1.19852	−5.34629
H	+6.25333	+3.43722	−5.75067
H	+7.15477	+3.47211	−4.18700
H	+4.96152	+2.84931	−2.97208
H	+4.22119	+6.45541	−2.77562
C	+2.71142	+5.15277	−3.72168
O	+1.62147	+5.69030	−2.94260
H	+0.76433	+5.35740	−3.32193
H	+2.78030	+5.71431	−4.69739
C	+2.49137	+3.66948	−4.01447
O	+1.31042	+3.56305	−4.84314
H	+1.30676	+2.65287	−5.26719
H	+2.33445	+3.10443	−3.05185
C	+3.71265	+3.09596	−4.74615
H	+3.78229	+3.53473	−5.77692
O	+3.52097	+1.63287	−4.84886
C	+3.47603	+1.10635	−6.20949
O	+4.61186	+0.20759	−6.39488
H	+3.60361	+1.93608	−6.95605
C	+2.18494	+0.32969	−6.43985
O	+1.07607	+1.23353	−6.24524
H	+0.24254	+0.69857	−6.15382
H	+2.17476	−0.04205	−7.50499
C	+2.08369	−0.86607	−5.49488
O	+0.88962	−1.60152	−5.85563
H	+0.95348	−2.51686	−5.44798
H	+2.00559	−0.51188	−4.42993
C	+3.32536	−1.75538	−5.64762

H	+3.32378	−2.24844	−6.65644
C	+4.61218	−0.94180	−5.45169
H	+4.64983	−0.54345	−4.40519
C	+5.89817	−1.70059	−5.79348
H	+5.78173	−2.20795	−6.79023
H	+6.73510	−0.97443	−5.88069
O	+6.24273	−2.64733	−4.78392
H	+5.55798	−3.36047	−4.77462

345

* E = 3.543 kcal/mol

C	−0.62985	−0.12439	+0.63649
C	−1.13894	−0.33806	−0.66013
C	−0.34268	−0.78229	−1.70862
C	+1.02863	−1.04384	−1.78950
C	+2.14206	−0.60574	−1.07319
C	+2.25678	+0.54630	−0.28392
C	+1.25988	+1.51665	−0.19905
H	+0.22075	+0.88792	+0.44068
C	+3.51301	+0.65058	+0.52919
C	+3.84628	−0.69112	+1.16172
C	+4.04002	−1.77840	+0.11139
C	+3.43708	−1.35737	−1.22985
C	−1.52715	+0.20593	+1.81489
C	−3.01415	+0.33010	+1.48395
C	−3.21091	+0.89930	+0.09470
C	−2.59910	−0.09072	−0.89371
C	−4.65478	+0.92780	−0.41497
C	−4.48784	+0.97223	−1.94409
C	−3.09255	+0.39563	−2.24930
O	+5.42562	−1.98647	−0.01293
H	+5.60188	−2.66670	−0.66061
C	−2.58077	+2.28380	−0.01244
C	−5.54128	+2.02133	+0.13656
H	+3.55808	−2.69960	+0.45038
H	+3.31017	−2.22425	−1.87685
H	+4.18149	−0.70834	−1.69832
H	+3.03407	−0.96878	+1.83228
H	+4.75617	−0.63671	+1.75348
H	+4.34236	+0.96398	−0.11148
H	+3.40408	+1.42424	+1.28779

H	+0.62238	+1.64753	−1.07012
H	+1.49245	+2.42763	+0.33966
H	+1.27686	−1.73617	−2.58843
H	−0.89109	−1.07160	−2.59817
H	+0.22817	−0.75140	+0.87873
H	−1.18170	+1.14020	+2.26303
H	−1.38955	−0.54867	+2.58955
H	−3.48441	−0.65529	+1.51531
H	−3.50797	+0.93988	+2.24249
H	−5.09256	−0.03752	−0.14523
H	−4.56429	+2.00030	−2.29776
H	−5.27846	+0.41663	−2.44157
H	−2.42161	+1.16199	−2.63886
H	−3.12504	−0.40084	−2.99041
H	−2.79780	+2.75317	−0.97056
H	−1.49867	+2.24915	+0.09639
H	−2.97117	+2.93604	+0.76823
H	−6.55488	+1.92151	−0.24370
H	−5.18021	+3.00745	−0.15161
H	−5.59344	+1.98560	+1.22416
H	−3.11090	−1.04120	−0.69494
O	−7.33006	−3.17910	−1.53634
H	−6.71559	−3.62179	−2.17222
C	−7.34681	−3.93271	−0.32464
H	−7.57194	−5.01357	−0.53349
H	−8.16327	−3.53397	+0.31507
C	−6.02003	−3.82002	+0.43199
H	−5.71288	−2.74817	+0.53708
O	−6.32482	−4.35587	+1.78422
C	−5.18959	−4.26508	+2.70008
O	−4.79815	−2.86424	+2.80611
H	−5.54872	−4.65109	+3.69173
C	−4.04040	−5.09896	+2.14543
O	−2.92633	−4.99449	+3.05717
H	−2.10997	−5.32866	+2.59689
H	−4.37178	−6.17575	+2.08837
C	−3.64241	−4.62625	+0.74809
O	−2.63647	−5.54036	+0.25119
H	−2.56920	−5.41896	−0.74271
H	−3.21897	−3.58436	+0.79973

C	−4.86826	−4.63291	−0.17490
H	−5.19701	−5.68860	−0.36756
O	−4.46081	−4.00746	−1.45280
C	−4.59559	−4.85700	−2.63100
O	−5.57455	−4.24697	−3.52810
C	−5.19381	−2.87626	−3.95622
C	−6.37648	−2.40945	−4.81003
O	−6.32487	−1.00603	−5.06355
H	−5.57465	−0.82612	−5.68191
H	−6.39619	−2.98537	−5.77445
H	−7.32200	−2.62470	−4.26734
H	−5.08554	−2.23617	−3.04354
H	−5.00853	−5.86216	−2.34714
C	−3.26330	−4.97655	−3.36270
O	−2.30665	−5.57300	−2.46209
H	−1.39377	−5.43334	−2.83236
H	−3.40338	−5.65306	−4.25459
C	−2.77410	−3.61273	−3.84642
O	−1.57990	−3.83377	−4.63419
H	−1.39486	−2.99872	−5.15915
H	−2.53269	−2.95319	−2.96629
C	−3.85506	−2.94095	−4.70373
H	−3.97513	−3.49207	−5.67391
O	−3.39835	−1.56093	−4.97870
C	−3.20738	−1.23123	−6.38716
O	−4.15618	−0.17896	−6.74364
C	−4.00726	+1.03798	−5.90352
C	−5.11822	+1.97174	−6.39325
O	−5.34963	+3.04330	−5.47940
H	−4.56236	+3.64183	−5.48942
H	−4.85745	+2.36897	−7.41184
H	−6.06260	+1.39356	−6.48728
H	−4.17411	+0.75189	−4.83405
H	−3.45043	−2.11488	−7.03621
C	−1.79238	−0.71676	−6.62580
O	−0.86553	−1.76891	−6.28220
H	+0.04582	−1.37607	−6.21059
H	−1.67927	−0.46641	−7.71958
C	−1.51958	+0.53487	−5.79410
O	−0.21350	+1.03284	−6.17116

H	−0.12563	+1.96721	−5.81581
H	−1.52590	+0.27587	−4.69851
C	−2.58922	+1.60010	−6.07225
H	−2.45447	+2.01762	−7.10536
O	−2.39543	+2.67978	−5.07892
C	−2.09854	+3.99389	−5.63481
O	−3.18494	+4.90104	−5.26898
C	−3.37869	+5.00378	−3.79947
C	−4.59756	+5.91794	−3.64469
O	−5.11810	+5.88497	−2.31621
H	−4.47429	+6.33596	−1.71636
H	−4.32198	+6.96754	−3.93582
H	−5.39795	+5.57393	−4.33448
H	−3.59608	+3.98061	−3.39761
H	−2.08016	+3.95473	−6.75703
C	−0.79299	+4.53136	−5.05994
O	+0.26757	+3.62407	−5.42709
H	+1.06370	+3.82140	−4.86378
H	−0.58811	+5.54360	−5.51380
C	−0.88245	+4.67538	−3.54156
O	+0.34305	+5.29991	−3.09202
H	+0.19306	+5.65046	−2.16401
H	−0.99164	+3.66150	−3.06421
C	−2.08930	+5.54650	−3.16812
H	−1.91291	+6.60587	−3.49414
O	−2.22214	+5.50495	−1.69471
C	−2.11854	+6.79608	−1.02418
O	−3.39346	+7.07608	−0.36684
C	−3.77791	+6.03288	+0.61861
C	−5.15320	+6.48182	+1.12141
O	−5.81979	+5.44262	+1.83720
H	−5.34538	+5.29666	+2.69236
H	−5.04046	+7.39624	+1.76462
H	−5.78538	+6.75657	+0.24982
H	−3.85223	+5.05037	+0.08525
H	−1.95931	+7.61830	−1.77242
C	−1.01833	+6.76521	+0.03090
O	+0.23395	+6.48820	−0.63077
H	+0.90406	+6.22623	+0.05602
H	−0.96143	+7.77947	+0.52128

C	−1.31073	+5.71149	+1.09730
O	−0.28987	+5.82931	+2.11713
H	−0.60807	+5.33965	+2.93291
H	−1.27543	+4.68314	+0.64092
C	−2.69864	+5.95069	+1.70681
H	−2.69131	+6.89042	+2.32040
O	−2.99676	+4.79646	+2.58462
C	−3.21651	+5.12303	+3.98917
O	−4.59218	+4.77007	+4.33339
C	−4.89567	+3.33445	+4.10205
C	−6.38277	+3.20542	+4.44500
O	−6.92648	+1.97028	+3.98099
H	−6.55357	+1.23715	+4.52980
H	−6.52533	+3.30886	+5.55458
H	−6.94051	+4.03106	+3.95303
H	−4.71685	+3.10298	+3.02015
H	−3.12028	+6.22920	+4.15731
C	−2.26726	+4.32711	+4.87806
O	−0.91464	+4.68421	+4.52390
H	−0.29483	+4.01371	+4.91889
H	−2.45618	+4.61187	+5.95302
C	−2.49187	+2.82514	+4.71328
O	−1.64436	+2.14847	+5.67185
H	−1.96790	+1.20271	+5.76053
H	−2.21357	+2.50771	+3.66871
C	−3.96435	+2.48020	+4.97284
H	−4.20680	+2.62033	+6.05956
O	−4.14862	+1.05735	+4.61002
C	−4.60094	+0.18845	+5.69145
O	−5.91683	−0.33771	+5.33657
H	−4.73594	+0.77517	+6.63941
C	−3.63816	−0.97964	+5.87423
O	−2.34319	−0.44394	+6.22315
H	−1.66195	−1.15808	+6.10124
H	−4.01131	−1.62655	+6.71910
C	−3.54791	−1.81901	+4.60138
O	−2.72815	−2.97528	+4.89704
H	−2.85046	−3.63778	+4.15307
H	−3.06934	−1.21570	+3.78073
C	−4.94604	−2.26497	+4.15157

H	−5.35005	−3.03624	+4.85961
C	−5.91102	−1.07633	+4.04738
H	−5.56385	−0.37938	+3.24245
C	−7.37572	−1.46894	+3.83186
H	−7.66354	−2.26999	+4.56579
H	−8.02048	−0.58490	+4.02563
O	−7.61624	−1.90230	+2.49382
H	−7.15339	−2.76485	+2.35348
O	+5.54090	−6.04980	−0.38146
H	+4.76683	−6.43525	+0.09899
C	+5.35378	−6.25674	−1.78126
H	+5.12546	−7.33526	−1.99568
H	+6.30579	−6.00274	−2.29536
C	+4.23455	−5.36981	−2.33420
H	+4.36538	−4.31061	−1.99021
O	+4.42097	−5.40921	−3.80692
C	+3.47902	−4.54230	−4.51507
O	+3.64806	−3.18333	−4.01575
H	+3.74698	−4.60099	−5.60387
C	+2.06204	−5.03570	−4.24486
O	+1.14383	−4.17726	−4.95362
H	+0.22694	−4.33455	−4.60049
H	+1.96516	−6.08805	−4.63925
C	+1.75795	−5.03329	−2.74740
O	+0.45170	−5.62939	−2.56714
H	+0.35349	−5.87376	−1.59875
H	+1.75253	−3.97599	−2.35881
C	+2.81728	−5.84889	−1.99426
H	+2.70613	−6.93884	−2.23734
O	+2.58454	−5.64400	−0.54723
C	+2.30000	−6.85593	+0.21372
O	+3.36598	−7.04313	+1.19492
C	+3.49725	−5.90456	+2.14135
C	+4.69295	−6.28889	+3.01850
O	+5.15924	−5.18778	+3.79545
H	+4.47802	−4.97280	+4.47868
H	+4.40981	−7.15092	+3.68191
H	+5.52723	−6.62456	+2.36557
H	+3.71495	−4.97667	+1.55255
H	+2.32454	−7.75672	−0.45668

C	+0.96874	−6.72807	+0.94546
O	−0.06973	−6.53375	−0.03776
H	−0.88518	−6.20184	+0.42548
H	+0.77284	−7.68739	+1.50585
C	+0.99646	−5.56858	+1.93933
O	−0.25367	−5.59205	+2.66843
H	−0.14641	−5.02475	+3.48891
H	+1.09838	−4.59199	+1.38897
C	+2.17688	−5.73545	+2.90532
H	+2.00040	−6.61648	+3.57789
O	+2.25323	−4.50428	+3.72277
C	+2.09259	−4.69875	+5.15971
O	+3.33451	−4.30283	+5.81953
C	+3.71366	−2.89284	+5.54235
C	+5.05263	−2.71696	+6.26525
O	+5.72707	−1.52952	+5.85296
H	+5.21891	−0.74703	+6.17967
H	+4.88275	−2.71120	+7.37645
H	+5.70826	−3.58207	+6.02763
H	+3.83977	−2.77155	+4.43667
H	+1.93793	−5.78557	+5.39669
C	+0.95804	−3.83107	+5.69240
O	−0.26538	−4.23658	+5.04319
H	−0.95983	−3.54583	+5.21747
H	+0.86255	−4.00427	+6.80276
C	+1.23929	−2.35121	+5.43987
O	+0.17818	−1.59250	+6.06797
H	+0.47997	−0.63812	+6.14107
H	+1.24919	−2.14632	+4.33288
C	+2.59745	−1.96425	+6.04049
H	+2.54158	−1.98599	+7.16125
O	+2.89635	−0.58628	+5.59208
C	+3.05762	+0.39702	+6.65694
O	+4.42524	+0.90836	+6.60666
C	+4.76683	+1.51366	+5.29309
C	+6.23874	+1.91267	+5.43574
O	+6.83063	+2.21670	+4.17395
H	+6.42997	+3.05237	+3.82966
H	+6.32586	+2.78766	+6.13603
H	+6.80167	+1.06479	+5.88176

H	+4.64176	+0.73263	+4.50085
H	+2.93305	−0.08493	+7.66362
C	+2.09298	+1.55980	+6.45499
O	+0.74585	+1.04501	+6.51820
H	+0.12627	+1.73462	+6.15770
H	+2.24390	+2.30450	+7.28837
C	+2.34720	+2.24901	+5.11580
O	+1.47579	+3.40291	+5.04693
H	+1.81259	+4.00409	+4.31719
H	+2.11174	+1.54355	+4.27066
C	+3.81497	+2.68641	+5.01924
H	+4.01265	+3.52547	+5.73801
O	+4.03761	+3.16442	+3.63652
C	+4.46407	+4.55448	+3.51790
O	+5.79750	+4.57680	+2.92190
C	+5.84684	+3.91876	+1.59022
C	+7.32395	+3.99089	+1.19145
O	+7.61842	+3.13474	+0.08924
H	+7.17312	+3.49358	−0.71726
H	+7.59769	+5.05457	+0.95196
H	+7.94735	+3.66662	+2.05225
H	+5.51784	+2.85500	+1.70881
H	+4.55986	+5.02658	+4.53242
C	+3.51024	+5.32838	+2.61519
O	+2.19771	+5.30218	+3.21585
H	+1.53309	+5.59125	+2.53452
H	+3.86442	+6.39703	+2.54738
C	+3.47481	+4.72375	+1.21302
O	+2.64072	+5.57585	+0.39276
H	+2.81981	+5.35275	−0.56956
H	+3.03563	+3.68645	+1.25334
C	+4.89455	+4.65146	+0.63496
H	+5.27494	+5.68586	+0.42254
O	+4.81467	+3.89003	−0.63144
C	+5.24218	+4.61633	−1.82225
O	+6.40982	+3.93850	−2.37966
C	+6.14290	+2.52285	−2.74542
C	+7.50065	+2.00568	−3.23048
O	+7.51737	+0.58462	−3.35405
H	+6.92628	+0.32678	−4.10321

H	+7.75880	+2.49144	−4.21076
H	+8.28118	+2.29390	−2.49406
H	+5.81122	+1.97502	−1.82627
H	+5.57628	+5.65415	−1.55214
C	+4.13389	+4.63017	−2.86875
O	+2.98723	+5.29891	−2.30244
H	+2.19435	+5.10318	−2.87065
H	+4.49459	+5.21152	−3.76585
C	+3.77315	+3.21224	−3.30786
O	+2.80649	+3.32345	−4.38004
H	+2.76477	+2.44005	−4.85403
H	+3.32404	+2.64316	−2.44720
C	+5.03016	+2.48389	−3.80230
H	+5.38788	+2.94340	−4.76189
O	+4.65148	+1.07460	−4.04756
C	+4.83225	+0.60664	−5.41632
O	+5.82474	−0.46774	−5.40440
H	+5.25233	+1.42320	−6.06275
C	+3.52395	+0.05334	−5.97108
O	+2.55193	+1.12030	−5.98441
H	+1.64819	+0.72307	−6.10595
H	+3.70162	−0.29886	−7.02785
C	+3.02475	−1.12018	−5.13013
O	+1.86177	−1.66994	−5.79558
H	+1.66693	−2.56477	−5.38616
H	+2.74022	−0.76197	−4.10218
C	+4.11807	−2.19108	−5.00945
H	+4.27152	−2.70079	−5.99734
C	+5.43589	−1.58543	−4.50752
H	+5.29192	−1.17450	−3.47546
C	+6.63140	−2.54063	−4.54308
H	+6.69711	−3.03177	−5.55056
H	+7.56529	−1.96010	−4.38520
O	+6.54483	−3.52482	−3.51033
H	+5.82778	−4.16335	−3.74742

345

* E = 3.613 kcal/mol

C	−0.56807	+0.75613	+0.24586
C	−1.22817	−0.45141	+0.55264
C	−0.54122	−1.60763	+0.90261

C	+0.82622	−1.87787	+1.00159
C	+1.96859	−1.30188	+0.44514
C	+2.06498	−0.52501	−0.71494
C	+0.99550	−0.31865	−1.58494
H	+0.11125	+0.44684	−0.85344
C	+3.40624	+0.09652	−0.97366
C	+4.04187	+0.70519	+0.27570
C	+3.83231	−0.15459	+1.52868
C	+3.28204	−1.52046	+1.14460
C	−1.31974	+2.05250	+0.00744
C	−2.83999	+1.95442	+0.12345
C	−3.32968	+0.60949	−0.37183
C	−2.72651	−0.45832	+0.53661
C	−4.82026	+0.32625	−0.16303
C	−4.89527	−1.20997	−0.19153
C	−3.49130	−1.72424	+0.17765
O	+4.97951	−0.23990	+2.33464
H	+5.71544	−0.57350	+1.82340
C	−2.94265	+0.40204	−1.83201
C	−5.77514	+1.00369	−1.11920
H	+3.09245	+0.31670	+2.17479
H	+3.16327	−2.12461	+2.04019
H	+4.00319	−2.02816	+0.49630
H	+3.64796	+1.70553	+0.44560
H	+5.11199	+0.82643	+0.11338
H	+4.06709	−0.68215	−1.36351
H	+3.32942	+0.84083	−1.76363
H	+0.24446	−1.10260	−1.63993
H	+1.19634	+0.19029	−2.52026
H	+1.04509	−2.70271	+1.67247
H	−1.16971	−2.41094	+1.27016
H	+0.38794	+0.87206	+0.75698
H	−1.06208	+2.42489	−0.98655
H	−0.94846	+2.80847	+0.69897
H	−3.14008	+2.06230	+1.16798
H	−3.30182	+2.77742	−0.42478
H	−5.05438	+0.66211	+0.85096
H	−5.17900	−1.55085	−1.18740
H	−5.65917	−1.57863	+0.48774
H	−3.01603	−2.22647	−0.66574

H	−3.51315	−2.43802	+0.99911
H	−3.37743	−0.50958	−2.23897
H	−1.86379	+0.33390	−1.96019
H	−3.29699	+1.23516	−2.43855
H	−6.80556	+0.77748	−0.85652
H	−5.61789	+0.66592	−2.14266
H	−5.66153	+2.08688	−1.09994
H	−3.04361	−0.17835	+1.54936
O	−6.69974	−4.65601	−1.42994
H	−6.05760	−4.83728	−2.15993
C	−6.39525	−5.51412	−0.33075
H	−6.37984	−6.58895	−0.65946
H	−7.20420	−5.40263	+0.42301
C	−5.05518	−5.15258	+0.31704
H	−4.99341	−4.05079	+0.50483
O	−5.08245	−5.85059	+1.62850
C	−3.89060	−5.59355	+2.43396
O	−3.77406	−4.15282	+2.63659
H	−4.04853	−6.11860	+3.41395
C	−2.67233	−6.12689	+1.68900
O	−1.50509	−5.88568	+2.50284
H	−0.69393	−6.00168	+1.93858
H	−2.79773	−7.23795	+1.54108
C	−2.53009	−5.45260	+0.32624
O	−1.43347	−6.09544	−0.36672
H	−1.48144	−5.82943	−1.33304
H	−2.30369	−4.35809	+0.46242
C	−3.82629	−5.60940	−0.48108
H	−3.94649	−6.67630	−0.80841
O	−3.70186	−4.74262	−1.67438
C	−3.77300	−5.42992	−2.95739
O	−4.94701	−4.93694	−3.67571
C	−4.91693	−3.46657	−3.88630
C	−6.25402	−3.15598	−4.56526
O	−6.53792	−1.75719	−4.55769
H	−5.91202	−1.30701	−5.17683
H	−6.24574	−3.55247	−5.61647
H	−7.06845	−3.67338	−4.01415
H	−4.85495	−2.96575	−2.88556
H	−3.92922	−6.53208	−2.80904

C	−2.52860	−5.13451	−3.78687
O	−1.37999	−5.62707	−3.06533
H	−0.55939	−5.23232	−3.46638
H	−2.61095	−5.68158	−4.77001
C	−2.39795	−3.63677	−4.05953
O	−1.26653	−3.45262	−4.94305
H	−1.33787	−2.54134	−5.35663
H	−2.22282	−3.08356	−3.09446
C	−3.67893	−3.11216	−4.72167
H	−3.77516	−3.53349	−5.75750
O	−3.55981	−1.63901	−4.80344
C	−3.62374	−1.08338	−6.15080
O	−4.80152	−0.22278	−6.23811
C	−4.78666	+0.87974	−5.24267
C	−6.12311	+1.59663	−5.45596
O	−6.42356	+2.48426	−4.37953
H	−5.79302	+3.24507	−4.41521
H	−6.10315	+2.15141	−6.43285
H	−6.93514	+0.84018	−5.51233
H	−4.73755	+0.43108	−4.21710
H	−3.76936	−1.89969	−6.90819
C	−2.38271	−0.24800	−6.44544
O	−1.22798	−1.10927	−6.35489
H	−0.41299	−0.54193	−6.29990
H	−2.46164	+0.15606	−7.49557
C	−2.26288	+0.92030	−5.46879
O	−1.13839	+1.72843	−5.89215
H	−1.20271	+2.61543	−5.42784
H	−2.08142	+0.53262	−4.42801
C	−3.54997	+1.75616	−5.48421
H	−3.64236	+2.29988	−6.46168
O	−3.44591	+2.74265	−4.38467
C	−3.49090	+4.14166	−4.79642
O	−4.68476	+4.75392	−4.21786
C	−4.71344	+4.68121	−2.73428
C	−6.06328	+5.29761	−2.35522
O	−6.40192	+5.03109	−0.99485
H	−5.80163	+5.55902	−0.41290
H	−6.04120	+6.40405	−2.54834
H	−6.85543	+4.85687	−2.99794

H	−4.67024	+3.60327	−2.43032
H	−3.60166	+4.22095	−5.91118
C	−2.26062	+4.88856	−4.29302
O	−1.09185	+4.26622	−4.86664
H	−0.28775	+4.58800	−4.37710
H	−2.32054	+5.95858	−4.64498
C	−2.19074	+4.87008	−2.76694
O	−1.07003	+5.69605	−2.37088
H	−1.17028	+5.91101	−1.39568
H	−2.03405	+3.81567	−2.40185
C	−3.49294	+5.42409	−2.17392
H	−3.57406	+6.52368	−2.38289
O	−3.43347	+5.20727	−0.71111
C	−3.50600	+6.41540	+0.10498
O	−4.72439	+6.35388	+0.90872
C	−4.78389	+5.14559	+1.77146
C	−6.14846	+5.24050	+2.46051
O	−6.51728	+4.00714	+3.07600
H	−5.90951	+3.83946	+3.83794
H	−6.13218	+6.07185	+3.21677
H	−6.92192	+5.48822	+1.70206
H	−4.73278	+4.24003	+1.11444
H	−3.59799	+7.32595	−0.54589
C	−2.30476	+6.50052	+1.04032
O	−1.10853	+6.55565	+0.23221
H	−0.32607	+6.35622	+0.81236
H	−2.38580	+7.44744	+1.64699
C	−2.25910	+5.30052	+1.98367
O	−1.17893	+5.52124	+2.92187
H	−1.27893	+4.85717	+3.66820
H	−2.06242	+4.36293	+1.39373
C	−3.58916	+5.14990	+2.73445
H	−3.69935	+5.96870	+3.49382
O	−3.54810	+3.84132	+3.42503
C	−3.64371	+3.89710	+4.87914
O	−4.87201	+3.21505	+5.28230
C	−4.92041	+1.80111	+4.82707
C	−6.30194	+1.30926	+5.26824
O	−6.64973	+0.07704	+4.63760
H	−6.07064	−0.63643	+5.00327

H	−6.32613	+1.20253	+6.38653
H	−7.06394	+2.06631	+4.98350
H	−4.83231	+1.78630	+3.71078
H	−3.73972	+4.96020	+5.22835
C	−2.45413	+3.19039	+5.51881
O	−1.24941	+3.87049	+5.10671
H	−0.47125	+3.28540	+5.31413
H	−2.55390	+3.26155	+6.64019
C	−2.40755	+1.71730	+5.11573
O	−1.33187	+1.09858	+5.85937
H	−1.45962	+0.10416	+5.82252
H	−2.20958	+1.62680	+4.01108
C	−3.74289	+1.03959	+5.45079
H	−3.86975	+0.97239	+6.56388
O	−3.69899	−0.32588	+4.88106
C	−3.85417	−1.40704	+5.84814
O	−5.07434	−2.14245	+5.52282
H	−4.00010	−0.99280	+6.88186
C	−2.66871	−2.36365	+5.78307
O	−1.47301	−1.61949	+6.09521
H	−0.68100	−2.16785	+5.84444
H	−2.81617	−3.16929	+6.55905
C	−2.55673	−3.01740	+4.40656
O	−1.49303	−3.99613	+4.47981
H	−1.56993	−4.60075	+3.68286
H	−2.30874	−2.24258	+3.62831
C	−3.88221	−3.69983	+4.04171
H	−4.05235	−4.58889	+4.70501
C	−5.05691	−2.71861	+4.15372
H	−4.92318	−1.88399	+3.41906
C	−6.43901	−3.36075	+4.00203
H	−6.51268	−4.25945	+4.67193
H	−7.21376	−2.63261	+4.32518
O	−6.71073	−3.72015	+2.64779
H	−6.13493	−4.48634	+2.40556
O	+6.56649	−4.62074	−1.66409
H	+5.94416	−5.24076	−1.20953
C	+6.28652	−4.64566	−3.06350
H	+6.28254	−5.70131	−3.44755
H	+7.10069	−4.09814	−3.58535

C	+4.94796	−3.97474	−3.38497
H	+4.88230	−2.96782	−2.89899
O	+4.97986	−3.78279	−4.85800
C	+3.80490	−3.07508	−5.36184
O	+3.73307	−1.77822	−4.69806
H	+3.96037	−2.94540	−6.46654
C	+2.56120	−3.90097	−5.05357
O	+1.41178	−3.18576	−5.55450
H	+0.59258	−3.57918	−5.14943
H	+2.64364	−4.89005	−5.58967
C	+2.42923	−4.15706	−3.55319
O	+1.30240	−5.04509	−3.36189
H	+1.36433	−5.43091	−2.43745
H	+2.24711	−3.18787	−3.01118
C	+3.71220	−4.80774	−3.01855
H	+3.81139	−5.84998	−3.42305
O	+3.59542	−4.86407	−1.54499
C	+3.64974	−6.20132	−0.96560
O	+4.83262	−6.28180	−0.11057
C	+4.82873	−5.26521	+0.97273
C	+6.17073	−5.46456	+1.68303
O	+6.47703	−4.37031	+2.54676
H	+5.86999	−4.40784	+3.32693
H	+6.15573	−6.42982	+2.25710
H	+6.97677	−5.53582	+0.92112
H	+4.77570	−4.24885	+0.50375
H	+3.78424	−6.97484	−1.76876
C	+2.41057	−6.46868	−0.11838
O	+1.25257	−6.38254	−0.97589
H	+0.44013	−6.31640	−0.40563
H	+2.48158	−7.51248	+0.30345
C	+2.30644	−5.47080	+1.03356
O	+1.18255	−5.86950	+1.85492
H	+1.25303	−5.38628	+2.73183
H	+2.13409	−4.43451	+0.63017
C	+3.59817	−5.48648	+1.86219
H	+3.68745	−6.45750	+2.41781
O	+3.51138	−4.37332	+2.83302
C	+3.55607	−4.76432	+4.23877
O	+4.76374	−4.20142	+4.83506

C	+4.83094	−2.71784	+4.74352
C	+6.16721	−2.36750	+5.40668
O	+6.60761	−1.05629	+5.06140
H	+5.95051	−0.40245	+5.40610
H	+6.07373	−2.47513	+6.52316
H	+6.94194	−3.08798	+5.06480
H	+4.83405	−2.42325	+3.66280
H	+3.64444	−5.88011	+4.33387
C	+2.33755	−4.22414	+4.97870
O	+1.15655	−4.79608	+4.37512
H	+0.36491	−4.27076	+4.66917
H	+2.39619	−4.54872	+6.05717
C	+2.29347	−2.69960	+4.91398
O	+1.18846	−2.25418	+5.73776
H	+1.30638	−1.27084	+5.90274
H	+2.13042	−2.36765	+3.85050
C	+3.60747	−2.10539	+5.43833
H	+3.67462	−2.24861	+6.55010
O	+3.57448	−0.66159	+5.12962
C	+3.68799	+0.22983	+6.27431
O	+4.91764	+1.00537	+6.13535
C	+4.96928	+1.79736	+4.87476
C	+6.34754	+2.46347	+4.92198
O	+6.70291	+3.04579	+3.66758
H	+6.10599	+3.81588	+3.50263
H	+6.36523	+3.23962	+5.73542
H	+7.11209	+1.69643	+5.17079
H	+4.89348	+1.09368	+4.00439
H	+3.78617	−0.35706	+7.22746
C	+2.49977	+1.18430	+6.30976
O	+1.29382	+0.39923	+6.43605
H	+0.51968	+0.97613	+6.19816
H	+2.59870	+1.85747	+7.20955
C	+2.45697	+2.04164	+5.04641
O	+1.37280	+2.98991	+5.19935
H	+1.51225	+3.73129	+4.53927
H	+2.27012	+1.39140	+4.14584
C	+3.78990	+2.78063	+4.87275
H	+3.91474	+3.54007	+5.69049
O	+3.73600	+3.47952	+3.56739

C	+3.87429	+4.92839	+3.63260
O	+5.10043	+5.30749	+2.93115
C	+5.11429	+4.86190	+1.51487
C	+6.49763	+5.27710	+1.00558
O	+6.80659	+4.66662	−0.24723
H	+6.22810	+5.06659	−0.94194
H	+6.54925	+6.39643	+0.92315
H	+7.26552	+4.95538	+1.74127
H	+5.00004	+3.74797	+1.49227
H	+3.99842	+5.26810	+4.69576
C	+2.69458	+5.61290	+2.95043
O	+1.48809	+5.24244	+3.64930
H	+0.70717	+5.51066	+3.09502
H	+2.83585	+6.72973	+3.02259
C	+2.60824	+5.21635	+1.47772
O	+1.53785	+5.98739	+0.88076
H	+1.63968	+5.94046	−0.11615
H	+2.38013	+4.11712	+1.38884
C	+3.93912	+5.51611	+0.77504
H	+4.09309	+6.62517	+0.69953
O	+3.85611	+4.94042	−0.58604
C	+3.99035	+5.89769	−1.67753
O	+5.19352	+5.56316	−2.43715
C	+5.17109	+4.17623	−2.96876
C	+6.53122	+4.01445	−3.65403
O	+6.81779	+2.64707	−3.94527
H	+6.20768	+2.34314	−4.66160
H	+6.55778	+4.63307	−4.59170
H	+7.32561	+4.39312	−2.97561
H	+5.07474	+3.46870	−2.10631
H	+4.14313	+6.93600	−1.27824
C	+2.78580	+5.81319	−2.60794
O	+1.60623	+6.15640	−1.85047
H	+0.80415	+5.87616	−2.36754
H	+2.91993	+6.55926	−3.44302
C	+2.65824	+4.41268	−3.20405
O	+1.56966	+4.44120	−4.15749
H	+1.64037	+3.62346	−4.73491
H	+2.43166	+3.66862	−2.38990
C	+3.96499	+4.01654	−3.90440

H	+4.10536	+4.63244	−4.83197
O	+3.84348	+2.58971	−4.27818
C	+3.93938	+2.30791	−5.70609
O	+5.12099	+1.47821	−5.93129
H	+4.09887	+3.25569	−6.28685
C	+2.70706	+1.54640	−6.18070
O	+1.54923	+2.37863	−5.95608
H	+0.73316	+1.81626	−6.04420
H	+2.80969	+1.34558	−7.28571
C	+2.56888	+0.21642	−5.44184
O	+1.45289	−0.49384	−6.02836
H	+1.51531	−1.45633	−5.74952
H	+2.36854	+0.40362	−4.34860
C	+3.85764	−0.60381	−5.58879
H	+3.97172	−0.95239	−6.64947
C	+5.08633	+0.20941	−5.15916
H	+5.00999	+0.46085	−4.07052
C	+6.42956	−0.45830	−5.46809
H	+6.43277	−0.82905	−6.52871
H	+7.23985	+0.29580	−5.37015
O	+6.70975	−1.52435	−4.56147
H	+6.08609	−2.26869	−4.74918

345

* E = 3.762 kcal/mol

C	−0.51708	+0.98693	+0.69976
C	−1.15199	−0.25204	+0.87301
C	−0.46396	−1.45337	+0.98007
C	+0.90432	−1.72740	+0.91662
C	+2.00571	−1.04743	+0.39299
C	+2.02088	−0.09258	−0.62703
C	+0.89161	+0.23002	−1.38474
H	+0.07045	+0.88275	−0.54142
C	+3.32952	+0.60070	−0.85146
C	+4.46607	−0.38892	−1.11588
C	+4.30665	−1.67523	−0.32028
C	+3.37452	−1.46433	+0.86496
C	−1.36532	+2.21731	+0.83248
C	−2.66116	+2.17409	+0.01645
C	−3.22949	+0.75854	−0.11992
C	−2.64169	−0.15989	+0.96216

C	−4.72237	+0.61760	+0.19896
C	−4.89289	−0.88301	+0.47339
C	−3.50760	−1.40599	+0.89727
O	+3.76149	−2.62950	−1.20831
H	+3.55038	−3.43284	−0.73008
C	−2.91952	+0.22109	−1.51110
C	−5.68236	+1.17093	−0.82868
H	+5.27781	−2.02084	+0.03333
H	+3.80766	−0.69936	+1.51536
H	+3.30828	−2.37094	+1.46885
H	+5.41570	+0.08413	−0.88013
H	+4.49228	−0.66514	−2.16770
H	+3.24951	+1.31228	−1.67092
H	+3.57815	+1.18145	+0.04108
H	+0.16298	−0.56615	−1.51350
H	+1.04074	+0.85310	−2.25838
H	+1.16723	−2.66167	+1.40288
H	−1.06980	−2.30534	+1.26610
H	+0.48762	+1.06050	+1.11286
H	−0.77949	+3.09435	+0.56349
H	−1.61562	+2.33906	+1.89151
H	−3.39286	+2.83402	+0.48447
H	−2.47418	+2.58568	−0.97578
H	−4.88033	+1.15604	+1.13736
H	−5.23415	−1.39118	−0.42888
H	−5.65023	−1.06192	+1.23203
H	−3.11158	−2.10216	+0.15740
H	−3.53080	−1.93588	+1.84781
H	−3.30743	−0.78420	−1.66777
H	−1.84600	+0.19640	−1.68414
H	−3.35734	+0.86931	−2.26865
H	−6.70831	+1.09661	−0.47685
H	−5.61898	+0.62180	−1.76698
H	−5.48030	+2.22112	−1.03855
H	−2.86572	+0.34290	+1.91297
O	−7.09570	−4.26854	−0.16859
H	−6.53049	−4.59646	−0.91113
C	−6.75440	−4.98584	+1.01722
H	−6.83827	−6.09407	+0.84834
H	−7.48614	−4.70783	+1.80609

C	−5.34239	−4.64205	+1.50117
H	−5.19007	−3.53321	+1.51462
O	−5.30304	−5.13737	+2.90201
C	−4.03240	−4.85450	+3.56566
O	−3.80687	−3.41332	+3.52672
H	−4.13832	−5.20901	+4.62599
C	−2.92106	−5.58852	+2.82348
O	−1.67217	−5.31979	+3.49622
H	−0.92862	−5.59830	+2.89730
H	−3.13189	−6.69562	+2.86292
C	−2.85173	−5.14169	+1.36473
O	−1.86812	−5.97014	+0.69829
H	−1.98146	−5.84401	−0.29111
H	−2.53569	−4.06223	+1.31076
C	−4.22098	−5.30632	+0.69066
H	−4.44250	−6.39562	+0.53491
O	−4.14020	−4.63000	−0.62293
C	−4.37316	−5.48251	−1.78177
O	−5.56412	−4.99500	−2.47499
C	−5.44113	−3.57681	−2.90065
C	−6.80427	−3.24915	−3.51671
O	−6.97772	−1.84417	−3.69910
H	−6.37018	−1.54446	−4.41943
H	−6.91799	−3.79209	−4.49382
H	−7.60535	−3.60715	−2.83487
H	−5.25573	−2.94829	−1.99128
H	−4.59880	−6.53489	−1.46092
C	−3.18616	−5.41633	−2.73549
O	−2.01948	−5.89902	−2.03581
H	−1.21013	−5.64408	−2.55601
H	−3.39306	−6.08837	−3.61752
C	−2.96618	−3.98971	−3.23666
O	−1.90745	−4.03639	−4.22134
H	−1.93185	−3.18242	−4.74736
H	−2.66360	−3.32303	−2.38109
C	−4.25678	−3.45097	−3.86891
H	−4.47470	−4.00043	−4.82301
O	−4.03553	−2.01862	−4.17018
C	−4.16126	−1.65209	−5.57656
O	−5.27706	−0.71782	−5.71051

C	−5.10444	+0.51161	−4.89486
C	−6.39944	+1.29675	−5.12306
O	−6.54928	+2.35565	−4.17806
H	−5.86962	+3.04694	−4.37252
H	−6.41856	+1.69732	−6.17272
H	−7.26495	+0.60955	−5.00692
H	−5.00665	+0.21285	−3.81921
H	−4.42366	−2.55100	−6.19662
C	−2.88919	−0.97223	−6.07068
O	−1.79734	−1.90507	−5.92909
H	−0.93901	−1.40912	−6.01209
H	−3.01894	−0.71713	−7.16191
C	−2.61020	+0.31080	−5.28967
O	−1.46576	+0.94999	−5.90340
H	−1.42384	+1.89554	−5.57157
H	−2.37795	+0.06420	−4.21655
C	−3.83112	+1.23962	−5.34712
H	−3.95982	+1.63908	−6.38802
O	−3.57480	+2.36681	−4.42019
C	−3.54875	+3.68744	−5.04042
O	−4.65184	+4.47669	−4.49688
C	−4.57884	+4.64730	−3.02286
C	−5.85239	+5.42440	−2.67549
O	−6.11985	+5.40413	−1.27404
H	−5.44604	+5.96560	−0.81752
H	−5.76308	+6.48118	−3.04626
H	−6.71502	+4.95467	−3.19518
H	−4.59044	+3.63298	−2.54653
H	−3.73278	+3.60633	−6.14540
C	−2.23468	+4.39942	−4.73727
O	−1.16108	+3.59515	−5.26782
H	−0.29973	+3.91276	−4.88451
H	−2.24033	+5.40015	−5.25839
C	−2.05824	+4.62350	−3.23606
O	−0.85691	+5.40907	−3.04866
H	−0.87805	+5.79690	−2.12363
H	−1.95140	+3.63319	−2.70947
C	−3.27225	+5.37074	−2.66819
H	−3.29114	+6.42445	−3.05398
O	−3.12039	+5.39218	−1.19576

C	−3.07204	+6.71755	−0.58720
O	−4.22871	+6.86091	+0.29483
C	−4.29349	+5.79934	+1.33198
C	−5.59623	+6.08314	+2.08542
O	−5.99147	+4.97469	+2.89272
H	−5.33490	+4.86525	+3.62425
H	−5.47593	+7.00795	+2.71300
H	−6.40700	+6.27603	+1.35053
H	−4.34174	+4.80545	+0.81695
H	−3.16557	+7.51653	−1.37075
C	−1.80290	+6.87482	+0.24422
O	−0.66827	+6.72397	−0.63867
H	+0.13994	+6.54798	−0.08802
H	−1.78831	+7.90798	+0.69492
C	−1.75417	+5.83120	+1.35743
O	−0.60153	+6.11676	+2.18727
H	−0.68221	+5.56329	+3.02008
H	−1.64688	+4.81103	+0.90004
C	−3.03320	+5.86233	+2.20524
H	−3.04747	+6.77779	+2.85392
O	−3.00656	+4.65321	+3.06178
C	−2.99898	+4.90089	+4.49858
O	−4.23358	+4.36051	+5.06472
C	−4.39426	+2.90365	+4.81755
C	−5.77256	+2.57304	+5.39713
O	−6.22021	+1.28070	+4.98956
H	−5.65757	+0.60020	+5.43462
H	−5.73756	+2.64478	+6.51789
H	−6.50846	+3.32127	+5.03218
H	−4.37581	+2.72851	+3.71170
H	−3.00911	+6.00430	+4.70837
C	−1.80982	+4.20802	+5.15497
O	−0.60111	+4.73358	+4.56504
H	+0.15376	+4.13556	+4.81417
H	−1.81796	+4.44798	+6.25713
C	−1.88001	+2.69188	+4.97604
O	−0.79543	+2.11406	+5.74010
H	−0.97956	+1.13325	+5.84614
H	−1.76116	+2.42847	+3.88666
C	−3.22751	+2.15975	+5.48164

H	−3.28652	+2.26273	+6.59764
O	−3.29971	+0.72543	+5.11923
C	−3.43269	−0.19327	+6.24550
O	−4.71978	−0.87647	+6.13465
H	−3.46004	+0.37530	+7.21389
C	−2.31766	−1.23380	+6.23410
O	−1.05383	−0.54347	+6.32155
H	−0.33017	−1.17848	+6.07075
H	−2.44013	−1.89609	+7.13948
C	−2.37323	−2.10047	+4.97738
O	−1.36559	−3.13103	+5.11557
H	−1.56018	−3.84811	+4.44141
H	−2.15711	−1.47589	+4.06632
C	−3.76325	−2.73536	+4.84098
H	−3.92445	−3.49213	+5.65380
C	−4.86048	−1.66298	+4.88180
H	−4.74160	−0.96877	+4.01103
C	−6.28768	−2.21477	+4.94754
H	−6.35570	−2.98755	+5.76009
H	−6.98362	−1.38814	+5.20677
O	−6.70225	−2.76278	+3.69686
H	−6.19809	−3.59805	+3.53740
O	+6.09041	−5.59286	−1.48858
H	+5.44814	−6.05607	−0.89742
C	+5.65951	−5.74883	−2.84187
H	+5.50457	−6.83587	−3.08280
H	+6.46804	−5.36802	−3.50208
C	+4.37387	−4.96642	−3.12399
H	+4.46139	−3.90806	−2.76626
O	+4.27445	−4.95717	−4.60904
C	+3.12350	−4.19857	−5.09682
O	+3.22494	−2.83468	−4.59645
H	+3.18233	−4.21558	−6.21873
C	+1.85101	−4.86042	−4.58004
O	+0.71874	−4.11759	−5.07991
H	−0.08439	−4.37262	−4.55188
H	+1.80500	−5.91537	−4.97755
C	+1.84210	−4.90047	−3.05318
O	+0.66135	−5.63490	−2.64400
H	+0.77979	−5.90528	−1.68605

H	+1.80426	−3.85604	−2.63978
C	+3.10454	−5.60598	−2.54207
H	+3.06343	−6.70031	−2.78873
O	+3.12004	−5.44556	−1.06523
C	+3.11529	−6.68326	−0.29243
O	+4.35353	−6.74020	+0.48323
C	+4.52919	−5.56051	+1.36702
C	+5.90223	−5.76439	+2.01365
O	+6.37211	−4.56734	+2.63367
H	+5.80953	−4.37971	+3.42535
H	+5.84938	−6.60288	+2.75945
H	+6.63370	−6.05295	+1.22880
H	+4.52962	−4.63821	+0.72994
H	+3.12163	−7.57503	−0.97401
C	+1.93608	−6.70397	+0.67448
O	+0.71606	−6.65441	−0.09510
H	−0.03272	−6.42273	+0.51706
H	+1.96678	−7.66703	+1.26040
C	+2.00652	−5.52449	+1.64252
O	+0.93698	−5.68220	+2.60437
H	+1.11098	−5.05046	+3.36441
H	+1.86982	−4.55994	+1.07668
C	+3.36208	−5.49744	+2.36185
H	+3.42650	−6.34569	+3.09331
O	+3.43910	−4.21206	+3.09406
C	+3.57840	−4.32863	+4.54271
O	+4.86373	−3.74973	+4.92659
C	+4.99200	−2.31830	+4.54866
C	+6.42098	−1.94898	+4.95700
O	+6.81838	−0.69237	+4.40951
H	+6.31413	+0.02266	+4.87001
H	+6.50170	−1.93588	+6.07728
H	+7.11870	−2.72522	+4.57560
H	+4.86038	−2.23189	+3.43947
H	+3.61345	−5.40895	+4.84763
C	+2.46185	−3.57394	+5.25564
O	+1.19941	−4.14422	+4.85161
H	+0.47213	−3.52034	+5.12018
H	+2.59107	−3.71349	+6.36777
C	+2.50601	−2.08047	+4.93614

O	+1.50013	−1.43545	+5.75256
H	+1.69327	−0.45103	+5.76547
H	+2.28027	−1.91128	+3.84631
C	+3.89533	−1.51849	+5.26491
H	+4.06386	−1.53701	+6.37432
O	+3.93039	−0.11707	+4.78960
C	+4.16894	+0.88369	+5.82458
O	+5.43421	+1.55430	+5.53243
C	+5.44818	+2.21731	+4.20284
C	+6.86486	+2.78780	+4.08588
O	+7.16359	+3.19768	+2.75230
H	+6.61994	+3.99509	+2.53793
H	+6.98524	+3.64649	+4.80060
H	+7.59623	+2.00234	+4.37409
H	+5.26621	+1.43983	+3.41782
H	+4.29455	+0.39214	+6.82652
C	+3.05400	+1.92388	+5.83777
O	+1.80998	+1.25376	+6.12877
H	+1.06133	+1.86811	+5.90104
H	+3.27152	+2.67143	+6.65420
C	+2.97132	+2.65699	+4.50080
O	+1.97475	+3.69873	+4.63396
H	+2.09042	+4.33790	+3.86964
H	+2.66651	+1.94052	+3.68796
C	+4.33357	+3.27119	+4.15354
H	+4.56397	+4.11735	+4.85385
O	+4.23589	+3.79159	+2.77226
C	+4.44725	+5.22711	+2.62674
O	+5.63802	+5.43191	+1.80458
C	+5.53387	+4.77131	+0.47794
C	+6.88747	+5.03870	−0.18721
O	+7.10429	+4.18140	−1.30728
H	+6.47642	+4.43686	−2.02754
H	+6.95191	+6.11772	−0.49517
H	+7.69700	+4.84825	+0.54988
H	+5.38431	+3.67391	+0.64191
H	+4.65917	+5.70036	+3.62279
C	+3.25649	+5.87111	+1.92602
O	+2.08886	+5.68767	+2.75480
H	+1.28429	+5.93690	+2.22521

H	+3.45829	+6.97441	+1.81048
C	+3.04140	+5.25232	+0.54602
O	+1.97359	+5.98641	−0.09983
H	+1.99683	+5.76786	−1.07911
H	+2.74767	+4.17084	+0.65412
C	+4.33065	+5.34681	−0.28186
H	+4.52371	+6.41399	−0.57073
O	+4.12440	+4.53795	−1.50314
C	+4.24444	+5.26456	−2.76155
O	+5.35941	+4.69570	−3.51483
C	+5.19223	+3.24347	−3.78175
C	+6.48634	+2.83857	−4.49352
O	+6.63616	+1.42069	−4.54987
H	+5.96157	+1.05870	−5.17598
H	+6.50344	+3.28040	−5.52645
H	+7.35233	+3.25268	−3.93350
H	+5.09572	+2.71235	−2.80044
H	+4.50166	+6.34158	−2.57424
C	+2.96730	+5.11446	−3.57945
O	+1.87944	+5.68028	−2.81739
H	+1.01913	+5.38680	−3.22136
H	+3.08301	+5.69035	−4.54228
C	+2.69797	+3.64626	−3.90633
O	+1.54104	+3.60258	−4.77353
H	+1.51341	+2.70054	−5.21358
H	+2.48770	+3.07164	−2.95989
C	+3.91734	+3.03490	−4.61035
H	+4.03773	+3.48812	−5.63009
O	+3.67009	+1.58255	−4.74646
C	+3.64533	+1.08314	−6.11823
O	+4.74921	+0.14286	−6.28947
H	+3.82851	+1.92102	−6.84382
C	+2.33181	+0.36441	−6.40255
O	+1.25453	+1.30837	−6.22215
H	+0.39707	+0.80647	−6.17379
H	+2.33914	+0.01431	−7.47500
C	+2.15381	−0.84456	−5.48624
O	+0.93998	−1.51969	−5.89495
H	+0.94752	−2.44105	−5.49583
H	+2.06116	−0.50871	−4.41628

C	+3.36064	−1.78305	−5.62490
H	+3.36547	−2.25564	−6.64346
C	+4.67484	−1.02932	−5.37781
H	+4.70286	−0.66002	−4.32026
C	+5.93534	−1.83448	−5.70824
H	+5.82520	−2.30448	−6.72388
H	+6.80595	−1.14468	−5.74887
O	+6.20815	−2.82705	−4.72112
H	+5.48920	−3.50503	−4.74920

345

* E = 3.878 kcal/mol

C	−0.55380	+0.02102	+0.72773
C	−1.16513	−0.47911	−0.43421
C	−0.46494	−0.91301	−1.55238
C	+0.90341	−0.93688	−1.82800
C	+2.01016	−0.25833	−1.31229
C	+2.03639	+0.97299	−0.65106
C	+0.91875	+1.80662	−0.53526
H	+0.07074	+1.17207	+0.29972
C	+3.34599	+1.33803	−0.01601
C	+3.95001	+0.12129	+0.66662
C	+4.21777	−1.00362	−0.33299
C	+3.37550	−0.82698	−1.58884
C	−1.44162	+0.24173	+1.92043
C	−2.71174	+1.04122	+1.60767
C	−3.24452	+0.77731	+0.19635
C	−2.65785	−0.52912	−0.35804
C	−4.74063	+0.45554	+0.10738
C	−4.87987	−0.23094	−1.25840
C	−3.49502	−0.81554	−1.59264
O	+5.54969	−1.00919	−0.78831
H	+6.13498	−1.27197	−0.08133
C	−2.88699	+1.94621	−0.71076
C	−5.68988	+1.61322	+0.31760
H	+3.98324	−1.96519	+0.13102
H	+3.31696	−1.76797	−2.12906
H	+3.94207	−0.13541	−2.21752
H	+3.25429	−0.22737	+1.43012
H	+4.87986	+0.37724	+1.17149
H	+4.04029	+1.70439	−0.77841

H	+3.20438	+2.15625	+0.68857
H	+0.19933	+1.75123	−1.34801
H	+1.08181	+2.80354	−0.14254
H	+1.16402	−1.65740	−2.59704
H	−1.06709	−1.42030	−2.29676
H	+0.43909	−0.37205	+0.94109
H	−0.88446	+0.72071	+2.72414
H	−1.73037	−0.74145	+2.30659
H	−3.47154	+0.79411	+2.35039
H	−2.50651	+2.10598	+1.72370
H	−4.93378	−0.28910	+0.88486
H	−5.17992	+0.49637	−2.01230
H	−5.65454	−0.99271	−1.24055
H	−3.06500	−0.32602	−2.46718
H	−3.53153	−1.87946	−1.81866
H	−3.23054	+1.79703	−1.73333
H	−1.81011	+2.09564	−0.73823
H	−3.33392	+2.86752	−0.34049
H	−6.72155	+1.27042	+0.30567
H	−5.58563	+2.36044	−0.46752
H	−5.51734	+2.10302	+1.27622
H	−2.90823	−1.29428	+0.38926
O	−6.76305	−4.46882	−1.23253
H	−6.14020	−4.80497	−1.92274
C	−6.55108	−5.20806	−0.03060
H	−6.60990	−6.31237	−0.22946
H	−7.36557	−4.94792	+0.67924
C	−5.20232	−4.87379	+0.61366
H	−5.07550	−3.76646	+0.72022
O	−5.29387	−5.46920	+1.97232
C	−4.11396	−5.20950	+2.79400
O	−3.95426	−3.76510	+2.92780
H	−4.31589	−5.67718	+3.79505
C	−2.89496	−5.82451	+2.11586
O	−1.73744	−5.55170	+2.93355
H	−0.92073	−5.73487	+2.39560
H	−3.04630	−6.94053	+2.05059
C	−2.70461	−5.26623	+0.70670
O	−1.60469	−5.98616	+0.10140
H	−1.63352	−5.82486	−0.88886

H	−2.46056	−4.16840	+0.75655
C	−3.98681	−5.46629	−0.11236
H	−4.14721	−6.55846	−0.31499
O	−3.80644	−4.75209	−1.39556
C	−3.86407	−5.58771	−2.59026
O	−5.00729	−5.16055	−3.39475
C	−4.94110	−3.72429	−3.76902
C	−6.23851	−3.47176	−4.54272
O	−6.51750	−2.07722	−4.66447
H	−5.83435	−1.66745	−5.25077
H	−6.17566	−3.95364	−5.55639
H	−7.08431	−3.94219	−3.99655
H	−4.91918	−3.11693	−2.82850
H	−4.05034	−6.66027	−2.31439
C	−2.59571	−5.42128	−3.41931
O	−1.47463	−5.86583	−2.62623
H	−0.63620	−5.54887	−3.05835
H	−2.67852	−6.06895	−4.33888
C	−2.41199	−3.96645	−3.84571
O	−1.26405	−3.91085	−4.72574
H	−1.27867	−3.02446	−5.19648
H	−2.22827	−3.32159	−2.94056
C	−3.66350	−3.46550	−4.57974
H	−3.73567	−3.95004	−5.58956
O	−3.50940	−2.00329	−4.74795
C	−3.50890	−1.52721	−6.12571
O	−4.65659	−0.63948	−6.30147
C	−4.63909	+0.51521	−5.36650
C	−5.95071	+1.25136	−5.65371
O	−6.24971	+2.21164	−4.64075
H	−5.60198	+2.95518	−4.71426
H	−5.89518	+1.74408	−6.66183
H	−6.78131	+0.51374	−5.68211
H	−4.62652	+0.12214	−4.31747
H	−3.65388	−2.38169	−6.83999
C	−2.23269	−0.74666	−6.41873
O	−1.10988	−1.63507	−6.23659
H	−0.27779	−1.09081	−6.19168
H	−2.26014	−0.40058	−7.49208
C	−2.11311	+0.47281	−5.50572

O	−0.94817	+1.21772	−5.93082
H	−1.00440	+2.13793	−5.53526
H	−1.98759	+0.14296	−4.43585
C	−3.37217	+1.34222	−5.62559
H	−3.41722	+1.81373	−6.64310
O	−3.27388	+2.40526	−4.59947
C	−3.27491	+3.76966	−5.11590
O	−4.46496	+4.45178	−4.61065
C	−4.52414	+4.48823	−3.12689
C	−5.86678	+5.15765	−2.82027
O	−6.23111	+5.00462	−1.44856
H	−5.62092	+5.55591	−0.89977
H	−5.81956	+6.24522	−3.09793
H	−6.65828	+4.68357	−3.43952
H	−4.51049	+3.43496	−2.74560
H	−3.36207	+3.76732	−6.23560
C	−2.03718	+4.52569	−4.64454
O	−0.87177	+3.83539	−5.14235
H	−0.07369	+4.15346	−4.64125
H	−2.06529	+5.56614	−5.07987
C	−1.99649	+4.62541	−3.12078
O	−0.86859	+5.46255	−2.77048
H	−0.97153	+5.73524	−1.81071
H	−1.86363	+3.60165	−2.67249
C	−3.29906	+5.24507	−2.59575
H	−3.35364	+6.32897	−2.88155
O	−3.27611	+5.12960	−1.11997
C	−3.31769	+6.38972	−0.38841
O	−4.55451	+6.42772	+0.39037
C	−4.67310	+5.29214	+1.34155
C	−6.05320	+5.47747	+1.97895
O	−6.47464	+4.30408	+2.67378
H	−5.92124	+4.20605	+3.48736
H	−6.03559	+6.36235	+2.67093
H	−6.79590	+5.68690	+1.17937
H	−4.63171	+4.33591	+0.75891
H	−3.36117	+7.25839	−1.09882
C	−2.13302	+6.49086	+0.56652
O	−0.92106	+6.44997	−0.21539
H	−0.15430	+6.28108	+0.39557

H	−2.19072	+7.47829	+1.10895
C	−2.15600	+5.35781	+1.59152
O	−1.07560	+5.59082	+2.52664
H	−1.24536	+5.03341	+3.34338
H	−2.00601	+4.36769	+1.07543
C	−3.50050	+5.34424	+2.33035
H	−3.58572	+6.24680	+2.99191
O	−3.52476	+4.12208	+3.16527
C	−3.68466	+4.34616	+4.59780
O	−4.94527	+3.73962	+5.01970
C	−5.01597	+2.28881	+4.70880
C	−6.42238	+1.87609	+5.15360
O	−6.78862	+0.59945	+4.63193
H	−6.23247	−0.09140	+5.06950
H	−6.47906	+1.87678	+6.27603
H	−7.15514	+2.62183	+4.77718
H	−4.89572	+2.15766	+3.60230
H	−3.76813	+5.44387	+4.81956
C	−2.54333	+3.69289	+5.36982
O	−1.30662	+4.30121	+4.93811
H	−0.55025	+3.72926	+5.23742
H	−2.69014	+3.89153	+6.47002
C	−2.51342	+2.18458	+5.13049
O	−1.50276	+1.62095	+6.00179
H	−1.63411	+0.62641	+6.02081
H	−2.24561	+1.97694	+4.05798
C	−3.88118	+1.56100	+5.44208
H	−4.06322	+1.56926	+6.54944
O	−3.83848	+0.16025	+4.96345
C	−4.04099	−0.85653	+5.98953
O	−5.27013	−1.58390	+5.68077
H	−4.19813	−0.37798	+6.99337
C	−2.87607	−1.83984	+6.00402
O	−1.66929	−1.10245	+6.29487
H	−0.88787	−1.67784	+6.07650
H	−3.05347	−2.59623	+6.82171
C	−2.75293	−2.57082	+4.66803
O	−1.71337	−3.56719	+4.81256
H	−1.79516	−4.21426	+4.04960
H	−2.47060	−1.84199	+3.85616

C	−4.08525	−3.24088	+4.30598
H	−4.29035	−4.09176	+5.00843
C	−5.23951	−2.23004	+4.34289
H	−5.07261	−1.43645	+3.57053
C	−6.63185	−2.85017	+4.19336
H	−6.73442	−3.72039	+4.89677
H	−7.39697	−2.09504	+4.47461
O	−6.88825	−3.25638	+2.84975
H	−6.31169	−4.03214	+2.64112
O	+6.47113	−5.00105	−1.16895
H	+5.79972	−5.53956	−0.68241
C	+6.22286	−5.12756	−2.56947
H	+6.19587	−6.21043	−2.86930
H	+7.06565	−4.64673	−3.11041
C	+4.91369	−4.44717	−2.97891
H	+4.87267	−3.39833	−2.58986
O	+4.97892	−4.39721	−4.46275
C	+3.84130	−3.69338	−5.05104
O	+3.79413	−2.35240	−4.48351
H	+4.02729	−3.65035	−6.15778
C	+2.56654	−4.45873	−4.71258
O	+1.44883	−3.75589	−5.29733
H	+0.61090	−4.09917	−4.88633
H	+2.63279	−5.48994	−5.16447
C	+2.39131	−4.58476	−3.20029
O	+1.24091	−5.43155	−2.96378
H	+1.26016	−5.71688	−2.00235
H	+2.21283	−3.56723	−2.75059
C	+3.64411	−5.20615	−2.56739
H	+3.72489	−6.28735	−2.85780
O	+3.48285	−5.10657	−1.09851
C	+3.47266	−6.37680	−0.38486
O	+4.63105	−6.40957	+0.50769
C	+4.63831	−5.28423	+1.47658
C	+5.96358	−5.44088	+2.22711
O	+6.27105	−4.28026	+3.00025
H	+5.64975	−4.24551	+3.76866
H	+5.92239	−6.35193	+2.88227
H	+6.78205	−5.58874	+1.49039
H	+4.61867	−4.32209	+0.90252

H	+3.59727	−7.23409	−1.09947
C	+2.20568	−6.51320	+0.45286
O	+1.06954	−6.47121	−0.43559
H	+0.25037	−6.31247	+0.10569
H	+2.22836	−7.51168	+0.97766
C	+2.11367	−5.40453	+1.50035
O	+0.95751	−5.68274	+2.32550
H	+1.03272	−5.13426	+3.16191
H	+1.98968	−4.40646	+0.99485
C	+3.38615	−5.38653	+2.35795
H	+3.43330	−6.30923	+2.99489
O	+3.31395	−4.19354	+3.23189
C	+3.31969	−4.47165	+4.66383
O	+4.52919	−3.88818	+5.24171
C	+4.62545	−2.42157	+5.02642
C	+5.97640	−2.04044	+5.63931
O	+6.38408	−0.73018	+5.24741
H	+5.79386	−0.07177	+5.69004
H	+5.91920	−2.11917	+6.75851
H	+6.74866	−2.75830	+5.28821
H	+4.62028	−2.22497	+3.92397
H	+3.38012	−5.57723	+4.85117
C	+2.10275	−3.84615	+5.33658
O	+0.91689	−4.43020	+4.75830
H	+0.13326	−3.87217	+5.01156
H	+2.13608	−4.08901	+6.43766
C	+2.09841	−2.32884	+5.16121
O	+0.98363	−1.80463	+5.92115
H	+1.11957	−0.81664	+6.03102
H	+1.97325	−2.06848	+4.07306
C	+3.41635	−1.73651	+5.67736
H	+3.46842	−1.82961	+6.79449
O	+3.42779	−0.30481	+5.30421
C	+3.50688	+0.63159	+6.41972
O	+4.75872	+1.37672	+6.30477
C	+4.87873	+2.10055	+5.01284
C	+6.26786	+2.74362	+5.06685
O	+6.69451	+3.18826	+3.77979
H	+6.13876	+3.96144	+3.51247
H	+6.26220	+3.59596	+5.79914

H	+7.00131	+1.99125	+5.42875
H	+4.82128	+1.35163	+4.18246
H	+3.55119	+0.08051	+7.39715
C	+2.34420	+1.61622	+6.36939
O	+1.11571	+0.86888	+6.49537
H	+0.35589	+1.46485	+6.25505
H	+2.43521	+2.32832	+7.23901
C	+2.36200	+2.41231	+5.06571
O	+1.30761	+3.40112	+5.14288
H	+1.47282	+4.08744	+4.42922
H	+2.17502	+1.72410	+4.19459
C	+3.72172	+3.10087	+4.88593
H	+3.83537	+3.92777	+5.63617
O	+3.74352	+3.67107	+3.52113
C	+3.92662	+5.11627	+3.44984
O	+5.17877	+5.38521	+2.74697
C	+5.21054	+4.79636	+1.38302
C	+6.61278	+5.12524	+0.86209
O	+6.93549	+4.36656	−0.30213
H	+6.37219	+4.68284	−1.05090
H	+6.68838	+6.22661	+0.65168
H	+7.35831	+4.87894	+1.64858
H	+5.06960	+3.68899	+1.47297
H	+4.03706	+5.55271	+4.47867
C	+2.78012	+5.75637	+2.67583
O	+1.55187	+5.48435	+3.38444
H	+0.78732	+5.67696	+2.77784
H	+2.94629	+6.87117	+2.63785
C	+2.71437	+5.21164	+1.25023
O	+1.68451	+5.94838	+0.55010
H	+1.80281	+5.78878	−0.43408
H	+2.45173	+4.11562	+1.26917
C	+4.06854	+5.39635	+0.55171
H	+4.25583	+6.48652	+0.36175
O	+3.99911	+4.67889	−0.74053
C	+4.18240	+5.51246	−1.92475
O	+5.39359	+5.07562	−2.61333
C	+5.34391	+3.65466	−3.04769
C	+6.71476	+3.41713	−3.68922
O	+6.97775	+2.03048	−3.89010

H	+6.36175	+1.68860	−4.58340
H	+6.77542	+3.97806	−4.66184
H	+7.50277	+3.82078	−3.01732
H	+5.20937	+3.01044	−2.14063
H	+4.35442	+6.58243	−1.62904
C	+2.99544	+5.36826	−2.86951
O	+1.80822	+5.80158	−2.17227
H	+1.01181	+5.50933	−2.69228
H	+3.16384	+6.03801	−3.76159
C	+2.84536	+3.92671	−3.35251
O	+1.76982	+3.90893	−4.32140
H	+1.83495	+3.05418	−4.84404
H	+2.59129	+3.25183	−2.48795
C	+4.15626	+3.46051	−4.00050
H	+4.32999	+4.02095	−4.95755
O	+4.01913	+2.01779	−4.29729
C	+4.13515	+1.65841	−5.70699
O	+5.29943	+0.79188	−5.86838
H	+4.32967	+2.57139	−6.33184
C	+2.89436	+0.90439	−6.17058
O	+1.75362	+1.77628	−6.02414
H	+0.92626	+1.22641	−6.07362
H	+3.01983	+0.63985	−7.26009
C	+2.70529	−0.37757	−5.36343
O	+1.58056	−1.09093	−5.93365
H	+1.60945	−2.03482	−5.59503
H	+2.49091	−0.12831	−4.28748
C	+3.97036	−1.24215	−5.44598
H	+4.07969	−1.66347	−6.48084
C	+5.22664	−0.45055	−5.05455
H	+5.17182	−0.16424	−3.97355
C	+6.54417	−1.16879	−5.36426
H	+6.51020	−1.57899	−6.41164
H	+7.37615	−0.43346	−5.31796
O	+6.82524	−2.20551	−4.42656
H	+6.13186	−2.90522	−4.50774

345

* E = 3.975 kcal/mol

C	+0.50648	+0.48689	+0.52279
C	+1.06932	−0.08993	−0.62810

C	+0.32497	−0.54355	−1.70977
C	−1.05454	−0.58628	−1.92293
C	−2.16802	−0.54981	−1.07994
C	−2.23675	−0.89402	+0.27324
C	−1.17909	−1.49138	+0.96706
H	−0.22920	−0.53927	+1.07409
C	−3.51673	−0.53404	+0.96753
C	−3.97229	+0.85681	+0.55494
C	−4.17825	+0.96410	−0.95210
C	−3.50493	−0.20096	−1.67803
C	+1.45728	+1.12070	+1.49949
C	+2.63554	+0.22038	+1.88721
C	+3.08927	−0.68682	+0.73968
C	+2.56354	−0.15315	−0.60133
C	+4.59543	−0.68228	+0.45445
C	+4.69192	−1.23356	−0.97466
C	+3.33399	−0.94724	−1.64202
O	−5.56611	+0.95594	−1.17950
H	−5.74922	+1.04358	−2.11321
C	+2.58843	−2.10410	+0.98070
C	+5.47019	−1.40145	+1.45589
H	−3.75156	+1.90491	−1.31052
H	−3.41249	+0.01750	−2.74091
H	−4.18742	−1.04875	−1.57883
H	−3.21982	+1.57631	+0.87501
H	−4.90844	+1.12627	+1.03663
H	−4.29365	−1.26048	+0.71115
H	−3.38638	−0.59651	+2.04693
H	−0.51809	−2.12092	+0.37724
H	−1.38574	−1.86080	+1.96477
H	−1.31491	−0.63670	−2.97579
H	+0.90869	−0.80991	−2.58295
H	−0.43735	+1.00866	+0.37210
H	+0.92361	+1.44595	+2.39115
H	+1.84786	+2.03445	+1.03934
H	+3.46194	+0.84954	+2.22065
H	+2.35437	−0.39304	+2.74389
H	+4.89382	+0.36977	+0.43521
H	+4.88688	−2.30542	−0.94833
H	+5.51951	−0.78403	−1.51682

H	+2.81143	−1.87480	−1.87870
H	+3.43405	−0.39909	−2.57685
H	+2.87755	−2.78659	+0.18284
H	+1.50370	−2.11987	+1.05805
H	+2.98720	−2.49632	+1.91503
H	+6.52086	−1.28217	+1.20336
H	+5.25850	−2.46942	+1.47419
H	+5.32782	−1.01072	+2.46363
H	+2.92109	+0.88412	−0.65483
O	−7.29898	−3.56440	−1.23068
H	−6.70770	−3.91976	−1.93861
C	−7.14481	−4.36935	−0.06323
H	−7.31626	−5.45388	−0.30422
H	−7.91946	−4.05924	+0.67055
C	−5.76105	−4.19897	+0.57116
H	−5.51948	−3.11453	+0.71791
O	−5.89835	−4.83604	+1.90697
C	−4.68926	−4.73103	+2.71969
O	−4.37881	−3.31706	+2.90109
H	−4.92718	−5.21178	+3.70659
C	−3.54902	−5.44251	+2.00085
O	−2.36167	−5.32878	+2.81343
H	−1.57583	−5.59320	+2.26328
H	−3.81843	−6.53262	+1.89226
C	−3.31500	−4.84824	+0.61297
O	−2.30151	−5.65409	−0.03498
H	−2.32967	−5.45740	−1.01874
H	−2.95709	−3.78455	+0.70182
C	−4.62018	−4.88451	−0.19379
H	−4.89430	−5.94668	−0.43189
O	−4.38137	−4.14787	−1.45486
C	−4.55572	−4.92661	−2.67529
O	−5.65268	−4.33832	−3.44234
C	−5.41599	−2.91092	−3.77744
C	−6.70056	−2.47038	−4.48457
O	−6.76984	−1.04962	−4.61484
H	−6.09998	−0.76336	−5.28353
H	−6.76688	−2.96251	−5.49205
H	−7.57576	−2.80185	−3.88589
H	−5.27628	−2.34278	−2.82205

H	−4.86496	−5.97867	−2.43271
C	−3.28973	−4.88554	−3.52398
O	−2.21581	−5.47298	−2.76005
H	−1.35136	−5.22817	−3.18714
H	−3.46113	−5.50023	−4.45440
C	−2.94621	−3.45351	−3.93006
O	−1.81662	−3.51734	−4.83417
H	−1.74043	−2.63104	−5.29749
H	−2.67248	−2.84873	−3.02152
C	−4.14586	−2.80312	−4.63269
H	−4.30855	−3.27708	−5.63689
O	−3.81753	−1.37108	−4.81650
C	−3.78537	−0.90508	−6.19710
O	−4.83095	+0.10518	−6.36139
C	−4.67721	+1.23344	−5.40932
C	−5.90207	+2.11881	−5.65317
O	−6.08667	+3.05320	−4.58888
H	−5.37756	+3.74038	−4.64937
H	−5.79809	+2.65290	−6.63522
H	−6.80867	+1.47864	−5.70830
H	−4.69465	+0.81960	−4.36735
H	−4.03303	−1.74095	−6.90468
C	−2.43762	−0.26542	−6.51273
O	−1.41480	−1.27149	−6.35486
H	−0.52822	−0.82088	−6.32745
H	−2.44783	+0.08804	−7.58370
C	−2.16850	+0.92603	−5.59463
O	−0.94479	+1.55512	−6.04319
H	−0.88668	+2.45930	−5.61178
H	−2.04701	+0.56993	−4.53268
C	−3.33156	+1.92517	−5.66341
H	−3.33901	+2.43938	−6.66077
O	−3.11258	+2.92767	−4.59662
C	−2.96681	+4.30413	−5.05688
O	−4.07145	+5.08988	−4.51139
C	−4.11553	+5.08534	−3.02569
C	−5.36380	+5.90728	−2.68936
O	−5.74637	+5.75870	−1.32368
H	−5.06612	+6.19737	−0.75611
H	−5.17886	+6.98850	−2.93440

H	−6.20836	+5.55719	−3.32128
H	−4.22863	+4.02670	−2.67696
H	−3.06129	+4.35798	−6.17474
C	−1.64895	+4.89430	−4.56815
O	−0.57127	+4.10138	−5.10964
H	+0.26117	+4.31412	−4.60837
H	−1.56198	+5.95025	−4.95557
C	−1.58960	+4.91821	−3.04197
O	−0.36631	+5.59432	−2.66642
H	−0.43921	+5.85692	−1.70069
H	−1.58302	+3.86684	−2.64011
C	−2.80460	+5.67070	−2.48337
H	−2.73018	+6.76129	−2.73788
O	−2.78463	+5.50970	−1.01235
C	−2.68510	+6.75044	−0.25117
O	−3.90061	+6.90169	+0.54509
C	−4.12994	+5.76824	+1.47883
C	−5.46614	+6.10269	+2.14896
O	−6.00287	+4.98388	+2.85234
H	−5.42921	+4.79788	+3.63558
H	−5.33119	+6.97846	+2.84060
H	−6.19859	+6.39838	+1.36741
H	−4.20985	+4.82638	+0.87915
H	−2.64515	+7.63510	−0.94192
C	−1.48533	+6.70621	+0.68825
O	−0.29144	+6.55934	−0.10905
H	+0.45716	+6.29297	+0.48943
H	−1.43481	+7.68073	+1.25401
C	−1.61413	+5.55676	+1.68566
O	−0.50093	+5.65204	+2.60620
H	−0.71096	+5.08630	+3.40780
H	−1.57565	+4.57122	+1.14288
C	−2.94226	+5.66759	+2.44605
H	−2.91945	+6.55695	+3.13032
O	−3.08865	+4.43475	+3.25132
C	−3.17702	+4.63678	+4.69320
O	−4.48647	+4.17046	+5.14303
C	−4.73579	+2.74384	+4.81002
C	−6.16504	+2.48853	+5.29819
O	−6.69628	+1.27370	+4.77182

H	−6.20991	+0.51385	+5.17620
H	−6.18306	+2.47539	+6.42207
H	−6.81730	+3.32171	+4.95894
H	−4.66818	+2.62340	+3.69906
H	−3.12366	+5.73024	+4.94369
C	−2.09918	+3.83239	+5.41091
O	−0.81028	+4.30834	+4.96862
H	−0.11517	+3.66088	+5.26463
H	−2.19701	+4.00725	+6.52070
C	−2.25230	+2.33983	+5.12416
O	−1.27194	+1.63868	+5.92639
H	−1.53127	+0.66953	+5.95700
H	−2.06468	+2.13821	+4.03260
C	−3.66840	+1.87557	+5.49059
H	−3.80172	+1.89411	+6.60486
O	−3.81063	+0.48555	+5.00389
C	−4.10422	−0.50915	+6.02931
O	−5.41058	−1.09523	+5.73957
H	−4.18780	−0.02450	+7.03894
C	−3.05575	−1.61545	+6.01009
O	−1.77134	−1.02519	+6.30388
H	−1.06060	−1.67483	+6.05457
H	−3.30885	−2.36821	+6.81084
C	−3.03074	−2.31712	+4.65373
O	−2.10105	−3.42197	+4.75143
H	−2.26242	−4.03310	+3.97160
H	−2.68541	−1.59934	+3.85628
C	−4.43203	−2.83272	+4.29863
H	−4.71031	−3.68340	+4.97584
C	−5.47854	−1.71356	+4.38998
H	−5.25427	−0.92448	+3.62775
C	−6.92857	−2.19159	+4.26865
H	−7.09452	−3.06788	+4.95297
H	−7.60759	−1.37452	+4.59428
O	−7.26416	−2.53160	+2.92459
H	−6.75895	−3.34173	+2.66778
O	+5.79529	−5.51840	−1.36833
H	+5.08799	−6.00439	−0.87695
C	+5.51035	−5.59714	−2.76469
H	+5.35388	−6.66497	−3.07707

H	+6.39295	−5.20830	−3.31660
C	+4.28091	−4.76474	−3.13967
H	+4.36221	−3.72770	−2.72369
O	+4.33480	−4.68254	−4.62243
C	+3.27337	−3.84827	−5.18236
O	+3.39108	−2.51408	−4.60715
H	+3.44354	−3.81744	−6.29208
C	+1.92438	−4.46330	−4.82754
O	+0.88836	−3.62563	−5.38297
H	+0.01961	−3.87856	−4.96856
H	+1.86122	−5.48825	−5.29442
C	+1.76205	−4.59480	−3.31385
O	+0.51957	−5.29515	−3.07046
H	+0.52836	−5.62561	−2.12314
H	+1.72486	−3.57338	−2.83996
C	+2.93913	−5.38523	−2.72673
H	+2.88666	−6.45559	−3.06030
O	+2.81591	−5.32708	−1.25241
C	+2.68422	−6.61920	−0.58800
O	+3.84504	−6.81048	+0.27920
C	+3.98446	−5.74187	+1.30154
C	+5.28810	−6.08469	+2.02814
O	+5.73837	−5.00231	+2.84244
H	+5.12007	−4.90902	+3.60826
H	+5.14392	−7.01152	+2.64648
H	+6.07738	−6.29835	+1.27575
H	+4.07105	−4.75657	+0.77545
H	+2.70872	−7.45328	−1.33971
C	+1.42040	−6.66052	+0.26454
O	+0.28570	−6.45716	−0.60402
H	−0.50507	−6.22709	−0.04629
H	+1.34312	−7.67961	+0.74213
C	+1.45739	−5.59831	+1.36174
O	+0.29245	−5.79657	+2.19814
H	+0.42782	−5.27665	+3.04528
H	+1.42485	−4.57152	+0.90232
C	+2.73765	−5.74144	+2.19643
H	+2.69546	−6.68040	+2.80965
O	+2.80516	−4.57053	+3.09978
C	+2.79195	−4.87426	+4.52565

O	+4.06440	−4.43944	+5.09914
C	+4.32258	−2.98970	+4.89897
C	+5.71865	−2.77163	+5.48962
O	+6.26633	−1.51319	+5.09808
H	+5.76803	−0.79653	+5.56273
H	+5.67318	−2.85240	+6.60927
H	+6.39699	−3.57010	+5.11931
H	+4.32045	−2.77788	+3.79867
H	+2.72886	−5.98294	+4.69314
C	+1.65891	−4.12621	+5.21989
O	+0.41132	−4.56312	+4.64144
H	−0.30475	−3.92892	+4.91528
H	+1.67149	−4.39076	+6.31643
C	+1.82599	−2.61473	+5.07063
O	+0.78631	−1.97981	+5.85258
H	+1.04251	−1.01945	+5.98969
H	+1.72210	−2.32018	+3.98811
C	+3.20763	−2.18526	+5.58092
H	+3.26190	−2.30759	+6.69519
O	+3.37177	−0.75486	+5.23744
C	+3.59293	+0.13855	+6.36915
O	+4.91858	+0.73699	+6.22904
C	+5.08419	+1.46970	+4.94757
C	+6.54144	+1.94099	+4.96886
O	+6.97497	+2.37818	+3.68161
H	+6.49992	+3.21587	+3.45670
H	+6.66191	+2.76141	+5.72743
H	+7.19164	+1.09496	+5.27908
H	+4.91156	+0.75194	+4.10435
H	+3.60295	−0.43845	+7.33252
C	+2.55060	+1.25153	+6.38101
O	+1.24805	+0.64229	+6.51527
H	+0.55647	+1.31552	+6.27569
H	+2.74221	+1.91727	+7.27070
C	+2.62532	+2.08552	+5.10350
O	+1.71154	+3.19928	+5.25220
H	+1.91933	+3.86555	+4.53132
H	+2.31562	+1.45771	+4.22303
C	+4.05287	+2.60404	+4.87984
H	+4.29543	+3.39819	+5.63476

O	+4.09363	+3.19131	+3.52108
C	+4.42852	+4.60977	+3.46399
O	+5.69229	+4.75580	+2.74487
C	+5.64098	+4.20224	+1.36677
C	+7.06684	+4.38080	+0.83707
O	+7.28358	+3.63517	−0.36009
H	+6.75687	+4.04923	−1.08729
H	+7.27172	+5.47238	+0.66647
H	+7.78581	+4.01656	+1.60201
H	+5.37392	+3.11661	+1.43135
H	+4.60121	+5.01633	+4.49657
C	+3.34578	+5.38393	+2.72086
O	+2.10319	+5.21825	+3.43684
H	+1.35739	+5.51139	+2.84732
H	+3.62540	+6.47665	+2.71285
C	+3.20808	+4.89496	+1.27993
O	+2.25743	+5.76122	+0.61686
H	+2.34855	+5.62285	−0.37301
H	+2.82794	+3.83401	+1.26882
C	+4.56684	+4.95402	+0.56898
H	+4.87306	+6.02264	+0.41472
O	+4.40374	+4.29721	−0.74765
C	+4.66793	+5.14726	−1.90389
O	+5.81735	+4.60314	−2.62335
C	+5.60498	+3.20921	−3.09287
C	+6.93855	+2.82841	−3.74296
O	+7.02874	+1.42530	−3.98633
H	+6.39955	+1.19101	−4.71205
H	+7.07043	+3.40515	−4.69817
H	+7.76782	+3.11108	−3.05936
H	+5.39782	+2.56333	−2.20208
H	+4.96035	+6.18038	−1.57425
C	+3.46512	+5.16925	−2.84037
O	+2.33769	+5.70163	−2.11393
H	+1.50828	+5.50228	−2.62603
H	+3.69958	+5.84965	−3.70918
C	+3.15435	+3.77196	−3.37406
O	+2.08167	+3.90452	−4.33680
H	+2.04895	+3.06454	−4.88488
H	+2.82911	+3.10060	−2.53096

C	+4.40119	+3.18001	−4.04468
H	+4.63489	+3.74340	−4.98682
O	+4.09391	+1.77204	−4.38057
C	+4.16411	+1.43810	−5.79901
O	+5.22350	+0.44894	−5.98642
H	+4.45680	+2.33819	−6.40346
C	+2.84544	+0.83621	−6.27125
O	+1.80981	+1.82661	−6.09655
H	+0.92569	+1.37691	−6.17236
H	+2.93566	+0.58404	−7.36675
C	+2.51513	−0.43376	−5.48998
O	+1.32735	−1.01445	−6.08009
H	+1.23911	−1.95255	−5.73419
H	+2.31625	−0.17873	−4.41116
C	+3.68148	−1.42867	−5.57037
H	+3.74810	−1.85832	−6.60512
C	+5.01391	−0.77742	−5.17456
H	+4.98312	−0.48359	−4.09434
C	+6.24631	−1.63936	−5.46443
H	+6.18151	−2.05441	−6.50705
H	+7.15543	−1.00263	−5.40976
O	+6.39081	−2.69202	−4.51155
H	+5.64958	−3.33495	−4.63568

345

* E = 4.145 kcal/mol

C	−0.79702	−0.78997	−0.45836
C	−0.94468	+0.44113	−1.11578
C	+0.12250	+1.27695	−1.41823
C	+1.48576	+1.13659	−1.15542
C	+2.22161	+0.38901	−0.23166
C	+1.80332	−0.11321	+1.00326
C	+0.56135	+0.18604	+1.57241
H	−0.34678	−0.44528	+0.78955
C	+2.76854	−1.03117	+1.69374
C	+3.38321	−2.05365	+0.74264
C	+3.82509	−1.43362	−0.59090
C	+3.65705	+0.07903	−0.56245
C	−1.99464	−1.69457	−0.42183
C	−3.28834	−1.00689	+0.02726
C	−3.34376	+0.47094	−0.36846

C	−2.35245	+0.75694	−1.50557
C	−4.64767	+0.92350	−1.03614
C	−4.25471	+2.22304	−1.75246
C	−2.73489	+2.14522	−1.98794
O	+5.12368	−1.81820	−0.96777
H	+5.74700	−1.57602	−0.28422
C	−3.02941	+1.33222	+0.84768
C	−5.85218	+1.05553	−0.13315
H	+3.19637	−1.81373	−1.39504
H	+3.94568	+0.48831	−1.52767
H	+4.33533	+0.49481	+0.18882
H	+2.66967	−2.85556	+0.55762
H	+4.24732	−2.52249	+1.21128
H	+3.56566	−0.42584	+2.13336
H	+2.28076	−1.53187	+2.52826
H	+0.13744	+1.15332	+1.31572
H	+0.39372	−0.12412	+2.59697
H	+2.10724	+1.70646	−1.83791
H	−0.11712	+2.10999	−2.06878
H	+0.16073	−1.28657	−0.60945
H	−1.78561	−2.55379	+0.21315
H	−2.13638	−2.09627	−1.43056
H	−4.13665	−1.54597	−0.39646
H	−3.38497	−1.09480	+1.10974
H	−4.87206	+0.16976	−1.79562
H	−4.50134	+3.08235	−1.12841
H	−4.80901	+2.34650	−2.67888
H	−2.21198	+2.90378	−1.40475
H	−2.46093	+2.30449	−3.02916
H	−3.06021	+2.39789	+0.62601
H	−2.04173	+1.10011	+1.24044
H	−3.74988	+1.13596	+1.64000
H	−6.74113	+1.29797	−0.71005
H	−5.71317	+1.84897	+0.59965
H	−6.05063	+0.12933	+0.40589
H	−2.62021	+0.04909	−2.30186
O	−6.89358	−3.45343	−2.89232
H	−6.14950	−3.76755	−3.46319
C	−7.02943	−4.34550	−1.78672
H	−7.15952	−5.40322	−2.14419

H	−7.94790	−4.06116	−1.22974
C	−5.82571	−4.26561	−0.84334
H	−5.59195	−3.19988	−0.58797
O	−6.27906	−4.95408	+0.39259
C	−5.27775	−4.91891	+1.45666
O	−4.94804	−3.52379	+1.72969
H	−5.74978	−5.39698	+2.35656
C	−4.04023	−5.67723	+0.98777
O	−3.05490	−5.62658	+2.04435
H	−2.16984	−5.86455	+1.66032
H	−4.32371	−6.75132	+0.79601
C	−3.47882	−5.06290	−0.29223
O	−2.39758	−5.90904	−0.75304
H	−2.16429	−5.62446	−1.68667
H	−3.08475	−4.03500	−0.06717
C	−4.56145	−4.95399	−1.37468
H	−4.80730	−5.97035	−1.78157
O	−4.00097	−4.11292	−2.45786
C	−3.88106	−4.76262	−3.75798
O	−4.75975	−4.07060	−4.69921
C	−4.43256	−2.62777	−4.84317
C	−5.51379	−2.08771	−5.78352
O	−5.53910	−0.66071	−5.79381
H	−4.72196	−0.33691	−6.24697
H	−5.34764	−2.48826	−6.81996
H	−6.50711	−2.44536	−5.43689
H	−4.50710	−2.14616	−3.83493
H	−4.24610	−5.82342	−3.70346
C	−2.44642	−4.67945	−4.26705
O	−1.59057	−5.34762	−3.31592
H	−0.64645	−5.10901	−3.52277
H	−2.38595	−5.21438	−5.25845
C	−2.00847	−3.22781	−4.45901
O	−0.69154	−3.24860	−5.05653
H	−0.51047	−2.34105	−5.44442
H	−1.96778	−2.70360	−3.46159
C	−2.99911	−2.49896	−5.37642
H	−2.93390	−2.91372	−6.41723
O	−2.61605	−1.06882	−5.39646
C	−2.27860	−0.53593	−6.71289

O	−3.24374	+0.50856	−7.04879
C	−3.25080	+1.63252	−6.07759
C	−4.36740	+2.55201	−6.58095
O	−4.71208	+3.54515	−5.61571
H	−3.96564	+4.18965	−5.54773
H	−4.05354	+3.03168	−7.54707
H	−5.27425	+1.94269	−6.78398
H	−3.49763	+1.22469	−5.06401
H	−2.38849	−1.33149	−7.49813
C	−0.87796	+0.06707	−6.71181
O	+0.05922	−0.97248	−6.36303
H	+0.93020	−0.55095	−6.12975
H	−0.64860	+0.43808	−7.75242
C	−0.77545	+1.23769	−5.73568
O	+0.53893	+1.82184	−5.89654
H	+0.53678	+2.72029	−5.45028
H	−0.90368	+0.87231	−4.67873
C	−1.85896	+2.27871	−6.04805
H	−1.63997	+2.78422	−7.02565
O	−1.82743	+3.28222	−4.96099
C	−1.51016	+4.64705	−5.36775
O	−2.66927	+5.49096	−5.08429
C	−3.07278	+5.45149	−3.65438
C	−4.31527	+6.34451	−3.58527
O	−5.05340	+6.12918	−2.38252
H	−4.51483	+6.45221	−1.61832
H	−4.01360	+7.42349	−3.67754
H	−4.98213	+6.10549	−4.44152
H	−3.33343	+4.39558	−3.39065
H	−1.33852	+4.69933	−6.47626
C	−0.31870	+5.17408	−4.57583
O	+0.82624	+4.34877	−4.87885
H	+1.53516	+4.53187	−4.20540
H	−0.10873	+6.23310	−4.90161
C	−0.61244	+5.15794	−3.07709
O	+0.50595	+5.78546	−2.40462
H	+0.22316	+5.98829	−1.46356
H	−0.72011	+4.09487	−2.72107
C	−1.90625	+5.92815	−2.77800
H	−1.73681	+7.02913	−2.91576

O	−2.25323	+5.65952	−1.36391
C	−2.32320	+6.83353	−0.50320
O	−3.69219	+6.95561	−0.00537
C	−4.14108	+5.75644	+0.74797
C	−5.60389	+6.05274	+1.09054
O	−6.28494	+4.88604	+1.55152
H	−5.93605	+4.65365	+2.44697
H	−5.65407	+6.86993	+1.85976
H	−6.12349	+6.41421	+0.17725
H	−4.06458	+4.86415	+0.07373
H	−2.11338	+7.76780	−1.09007
C	−1.37803	+6.68015	+0.68294
O	−0.03485	+6.55047	+0.17120
H	+0.55410	+6.21936	+0.90175
H	−1.44354	+7.60939	+1.31932
C	−1.75534	+5.46801	+1.53385
O	−0.89112	+5.46889	+2.69455
H	−1.29813	+4.86924	+3.38835
H	−1.60311	+4.52032	+0.94499
C	−3.22485	+5.56805	+1.96502
H	−3.35627	+6.41838	+2.68567
O	−3.57550	+4.29797	+2.64034
C	−4.01351	+4.42970	+4.02605
O	−5.39753	+3.96987	+4.11896
C	−5.57236	+2.56113	+3.68131
C	−7.08018	+2.31740	+3.79453
O	−7.47559	+1.12719	+3.11351
H	−7.11492	+0.34916	+3.60582
H	−7.37538	+2.27059	+4.87777
H	−7.62095	+3.17127	+3.33268
H	−5.23729	+2.47785	+2.61535
H	−4.01592	+5.50864	+4.33779
C	−3.15017	+3.57404	+4.94660
O	−1.78752	+4.03860	+4.84546
H	−1.18641	+3.34979	+5.23706
H	−3.51016	+3.71116	+6.00680
C	−3.24625	+2.09558	+4.57490
O	−2.50262	+1.34805	+5.56729
H	−2.75863	+0.38183	+5.48179
H	−2.79691	+1.92632	+3.55718

C	−4.71389	+1.64630	+4.56572
H	−5.11547	+1.62789	+5.61359
O	−4.75083	+0.27573	+4.00441
C	−5.28094	−0.74917	+4.89766
O	−6.50508	−1.28723	+4.30885
H	−5.57518	−0.29800	+5.88320
C	−4.27688	−1.88373	+5.07158
O	−3.07175	−1.32996	+5.64017
H	−2.33854	−1.99556	+5.54563
H	−4.71033	−2.64018	+5.78749
C	−3.98264	−2.57186	+3.73952
O	−3.12596	−3.70665	+4.01144
H	−3.14735	−4.31228	+3.21156
H	−3.45711	−1.85598	+3.04614
C	−5.28952	−3.04157	+3.08685
H	−5.73420	−3.88488	+3.67894
C	−6.29261	−1.88588	+2.96572
H	−5.87947	−1.09624	+2.28618
C	−7.69649	−2.30557	+2.51927
H	−8.03914	−3.18534	+3.12823
H	−8.40129	−1.46745	+2.70819
O	−7.73998	−2.61061	+1.12610
H	−7.24827	−3.45488	+0.97366
O	+5.90418	−5.71292	−0.25255
H	+5.08844	−6.19924	+0.02087
C	+5.95372	−5.68736	−1.67994
H	+5.86248	−6.72787	−2.09525
H	+6.94665	−5.28917	−1.98002
C	+4.85826	−4.79635	−2.27211
H	+4.86986	−3.77931	−1.79747
O	+5.24656	−4.65674	−3.70260
C	+4.35498	−3.77012	−4.44688
O	+4.34451	−2.47256	−3.78986
H	+4.77643	−3.68884	−5.48516
C	+2.95104	−4.36540	−4.44208
O	+2.07999	−3.47456	−5.17314
H	+1.13954	−3.70613	−4.94937
H	+2.97627	−5.36678	−4.96052
C	+2.44960	−4.55414	−3.01168
O	+1.16746	−5.22429	−3.08208

H	+0.96378	−5.60120	−2.17597
H	+2.32776	−3.55110	−2.51295
C	+3.44803	−5.40085	−2.21209
H	+3.45852	−6.45230	−2.60584
O	+2.98026	−5.41519	−0.80595
C	+2.66277	−6.73316	−0.27105
O	+3.58863	−7.02183	+0.82388
C	+3.53112	−6.00777	+1.90687
C	+4.61863	−6.44647	+2.89194
O	+4.92554	−5.41805	+3.83327
H	+4.15675	−5.31402	+4.44619
H	+4.29442	−7.38301	+3.42113
H	+5.54612	−6.68014	+2.32652
H	+3.77465	−5.00628	+1.46774
H	+2.83029	−7.52745	−1.04693
C	+1.24036	−6.76326	+0.27762
O	+0.32703	−6.49208	−0.80572
H	−0.57474	−6.32379	−0.42132
H	+1.03428	−7.79456	+0.68539
C	+1.06550	−5.74019	+1.39844
O	−0.27111	−5.90125	+1.93093
H	−0.30804	−5.44122	+2.82170
H	+1.18968	−4.69763	+0.98928
C	+2.11289	−5.98136	+2.49369
H	+1.89361	−6.94170	+3.03136
O	+2.01852	−4.85409	+3.44770
C	+1.69004	−5.22363	+4.81920
O	+2.81111	−4.84585	+5.67753
C	+3.13322	−3.39788	+5.59887
C	+4.36177	−3.23599	+6.49887
O	+5.00071	−1.97556	+6.29959
H	+4.41320	−1.26512	+6.65720
H	+4.06158	−3.36225	+7.57420
H	+5.09606	−4.03389	+6.25601
H	+3.38405	−3.14877	+4.53622
H	+1.57601	−6.33666	+4.91403
C	+0.44437	−4.47795	+5.28336
O	−0.65566	−4.86781	+4.43446
H	−1.40530	−4.22809	+4.56980
H	+0.21655	−4.77839	+6.34629

C	+0.66472	−2.96736	+5.22749
O	−0.51181	−2.33527	+5.78390
H	−0.28263	−1.38173	+5.99739
H	+0.80584	−2.63794	+4.15920
C	+1.90801	−2.58723	+6.04296
H	+1.71201	−2.74279	+7.13695
O	+2.17415	−1.15331	+5.79242
C	+2.13211	−0.29581	+6.97200
O	+3.46156	+0.27623	+7.17160
C	+3.93551	+1.05709	+5.99966
C	+5.35126	+1.49438	+6.38722
O	+6.08178	+1.97894	+5.26121
H	+5.69065	+2.84300	+4.98110
H	+5.29811	+2.27703	+7.19167
H	+5.89959	+0.62058	+6.80043
H	+3.95990	+0.37810	+5.10957
H	+1.90305	−0.89899	+7.89115
C	+1.13406	+0.83826	+6.76925
O	−0.17371	+0.25807	+6.57828
H	−0.78346	+0.95944	+6.22285
H	+1.12235	+1.47900	+7.69734
C	+1.52386	+1.70228	+5.57114
O	+0.59848	+2.81361	+5.51836
H	+0.99118	+3.51769	+4.92010
H	+1.45026	+1.09991	+4.62245
C	+2.96091	+2.21414	+5.74036
H	+3.00652	+2.95803	+6.57953
O	+3.34283	+2.87933	+4.47542
C	+3.69302	+4.29046	+4.59319
O	+5.09336	+4.44946	+4.20700
C	+5.36641	+3.97806	+2.82456
C	+6.87842	+4.15582	+2.65742
O	+7.37076	+3.44789	+1.52013
H	+7.04534	+3.90086	+0.70320
H	+7.12499	+5.24925	+2.58057
H	+7.39168	+3.75531	+3.55806
H	+5.08503	+2.89635	+2.75868
H	+3.61670	+4.62864	+5.66145
C	+2.82647	+5.13370	+3.66456
O	+1.44892	+4.96369	+4.06048

H	+0.86367	+5.29683	+3.32811
H	+3.11553	+6.21717	+3.78697
C	+3.02715	+4.72585	+2.20601
O	+2.27184	+5.64990	+1.38673
H	+2.60446	+5.57231	+0.44307
H	+2.65067	+3.67789	+2.04418
C	+4.51620	+4.79458	+1.84178
H	+4.85847	+5.86335	+1.82435
O	+4.66110	+4.21232	+0.48981
C	+5.22731	+5.10171	−0.51753
O	+6.49114	+4.52702	−0.97530
C	+6.32974	+3.15625	−1.52545
C	+7.75600	+2.70807	−1.85696
O	+7.82298	+1.30273	−2.09669
H	+7.37181	+1.10921	−2.95555
H	+8.13578	+3.28032	−2.74594
H	+8.41891	+2.94076	−0.99579
H	+5.88551	+2.50625	−0.72874
H	+5.47450	+6.10140	−0.06933
C	+4.28406	+5.22586	−1.70922
O	+3.03879	+5.78928	−1.24219
H	+2.34593	+5.64347	−1.94042
H	+4.74598	+5.92405	−2.46491
C	+4.04711	+3.86503	−2.36077
O	+3.24813	+4.08142	−3.54866
H	+3.28863	+3.24626	−4.10396
H	+3.49316	+3.19438	−1.64673
C	+5.38469	+3.21113	−2.73282
H	+5.86469	+3.77099	−3.57876
O	+5.09267	+1.82590	−3.16533
C	+5.46690	+1.51329	−4.54244
O	+6.51442	+0.49820	−4.51947
H	+5.91281	+2.41614	−5.04072
C	+4.26607	+0.97657	−5.31249
O	+3.24294	+1.99542	−5.32034
H	+2.38459	+1.57739	−5.59941
H	+4.58416	+0.76414	−6.37369
C	+3.74460	−0.31007	−4.67877
O	+2.67907	−0.81619	−5.51962
H	+2.52317	−1.77696	−5.27410

H	+3.34616	−0.09603	−3.64776
C	+4.87211	−1.34549	−4.58530
H	+5.14430	−1.70896	−5.61258
C	+6.10561	−0.77782	−3.86988
H	+5.85515	−0.57788	−2.79580
C	+7.35520	−1.65761	−3.98429
H	+7.46934	−2.01268	−5.04673
H	+8.25170	−1.04778	−3.73800
O	+7.31896	−2.76303	−3.08488
H	+6.54294	−3.33047	−3.31560

S3.7. Initial MD Geometry for the Free System

C	−0.367918	−0.774568	−1.051312
C	−0.724112	0.469905	−0.713968
C	0.198446	1.601820	−0.623419
C	1.520551	1.667927	−0.419185
C	2.525507	0.627879	−0.172301
C	2.465225	−0.285322	0.809418
C	1.340340	−0.388965	1.779138
H	1.723878	−0.527438	2.790024
C	3.546158	−1.306388	1.017141
C	4.578608	−1.358717	−0.090622
C	4.948241	0.047688	−0.512686
C	3.725023	0.731851	−1.082119
C	−1.316204	−1.919615	−1.215650
C	−2.787565	−1.517932	−1.190074
C	−3.012343	−0.441760	−0.148043
C	−2.186263	0.776712	−0.550030
C	−4.405667	0.189751	−0.104063
C	−4.151512	1.536223	0.600485
C	−2.653396	1.851838	0.422346
O	5.477862	0.780059	0.568950
H	4.754701	1.002078	1.156099
C	−2.627202	−0.959985	1.233880
C	−5.499830	−0.637204	0.533336
H	5.739195	0.024712	−1.258500
H	3.482892	0.310996	−2.060015
H	3.955923	1.783617	−1.254605
H	4.177114	−1.888365	−0.955019
H	5.463717	−1.896247	0.242081
H	4.036633	−1.104788	1.973808
H	3.079197	−2.284751	1.143519
H	0.729005	−1.263750	1.549668
H	0.691218	0.480162	1.760358
H	1.949518	2.661220	−0.502553
H	−0.285204	2.559916	−0.771595
H	0.678655	−0.991995	−1.215460
H	−1.116307	−2.647974	−0.423690
H	−1.087060	−2.444212	−2.143963
H	−3.078255	−1.124570	−2.166006

H	−3.409277	−2.393778	−1.000468
H	−4.690048	0.389762	−1.140821
H	−4.408946	1.459651	1.656609
H	−4.784020	2.318932	0.187830
H	−2.116824	1.771825	1.367173
H	−2.485892	2.858917	0.048998
H	−2.913936	−0.265456	2.020926
H	−1.555426	−1.119954	1.313498
H	−3.127863	−1.905901	1.435296
H	−6.457555	−0.123612	0.474797
H	−5.293062	−0.822464	1.586085
H	−5.613033	−1.600634	0.038123
H	−2.558353	1.076791	−1.537428

S3.8. Initial MD Geometry for the Encapsulated System

C	0.203770	−1.232150	−0.644250
C	0.495800	−0.716360	0.554830
C	−0.500970	−0.341070	1.556070
C	−1.798490	−0.011660	1.467620
C	−2.719610	0.160830	0.340480
C	−2.481320	0.894740	−0.755640
C	−1.218710	1.643860	−1.000040
H	−1.445180	2.639080	−1.385300
C	−3.509200	1.064530	−1.832580
C	−4.676850	0.106540	−1.735700
C	−5.168660	0.048480	−0.305980
C	−4.066290	−0.471350	0.586620
C	1.217850	−1.675410	−1.649690
C	2.646520	−1.716680	−1.115480
C	2.895870	−0.524160	−0.214970
C	1.938520	−0.630710	0.968870
C	4.233460	−0.491320	0.527880
C	3.947800	0.458960	1.707230
C	2.418960	0.470120	1.905730
O	−5.506330	1.339720	0.161270
H	−6.336440	1.623890	−0.221830
C	2.695990	0.772080	−0.995520
C	5.447700	−0.108390	−0.287620
H	−6.030970	−0.612500	−0.221220
H	−3.990640	−1.554500	0.470950
H	−4.356080	−0.295890	1.624320
H	−4.371660	−0.895700	−2.038890
H	−5.476010	0.415010	−2.407910
H	−3.871560	2.095380	−1.789550
H	−3.020430	0.963220	−2.804430
H	−0.614600	1.140560	−1.758250
H	−0.609640	1.739970	−0.106960
H	−2.292010	0.115630	2.426510
H	−0.111720	−0.357210	2.568040
H	−0.835050	−1.330710	−0.929590
H	1.165260	−1.004140	−2.513900
H	0.932430	−2.654220	−2.039900
H	2.794580	−2.628900	−0.533120

H	3.353050	−1.748620	−1.946740
H	4.391270	−1.497750	0.925650
H	4.315020	1.459500	1.479550
H	4.470300	0.137050	2.604610
H	1.987790	1.429760	1.618970
H	2.136200	0.294040	2.941910
H	3.017800	1.641400	−0.425190
H	1.651620	0.922510	−1.258850
H	3.279380	0.751300	−1.915970
H	6.348930	−0.157630	0.318430
H	5.365110	0.908610	−0.668470
H	5.585820	−0.776110	−1.137310
H	2.180920	−1.579530	1.464280
O	−6.569070	−5.388840	−1.964610
H	−5.827760	−5.740360	−2.516470
C	−6.496910	−5.991610	−0.672860
H	−6.453900	−7.110850	−0.760350
H	−7.425370	−5.730270	−0.121050
C	−5.286700	−5.490470	0.120650
H	−5.244120	−4.371430	0.115630
O	−5.549910	−5.943610	1.510650
C	−4.529960	−5.495330	2.457420
O	−4.480120	−4.038360	2.420960
H	−4.856990	−5.854330	3.470040
C	−3.186110	−6.088910	2.049420
O	−2.188680	−5.636400	2.988670
H	−1.288910	−5.800270	2.596850
H	−3.259880	−7.213560	2.100640
C	−2.813170	−5.679560	0.625770
O	−1.596660	−6.381530	0.275580
H	−1.485670	−6.332420	−0.719980
H	−2.639360	−4.568670	0.577710
C	−3.939920	−6.061740	−0.343120
H	−4.002390	−7.178230	−0.438380
O	−3.602110	−5.477180	−1.660220
C	−3.439230	−6.437180	−2.745720
O	−4.469290	−6.173580	−3.748910
C	−4.411930	−4.787820	−4.281130
C	−5.602680	−4.703790	−5.240680
O	−5.899690	−3.354400	−5.598500

H	−5.169380	−3.012960	−6.171120
H	−5.393980	−5.319620	−6.157090
H	−6.499790	−5.130620	−4.742850
H	−4.540420	−4.075820	−3.426640
H	−3.612700	−7.483970	−2.377930
C	−2.070320	−6.280550	−3.398420
O	−1.061250	−6.551940	−2.403210
H	−0.179310	−6.256770	−2.757060
H	−1.980430	−7.035090	−4.231910
C	−1.897210	−4.876540	−3.975090
O	−0.630320	−4.846550	−4.675000
H	−0.620730	−4.034440	−5.264280
H	−1.890180	−4.118380	−3.143130
C	−3.045800	−4.559270	−4.942400
H	−2.950860	−5.184510	−5.869550
O	−2.931630	−3.128610	−5.303220
C	−2.741120	−2.855530	−6.722730
O	−3.891870	−2.092240	−7.201570
C	−4.087870	−0.819690	−6.460180
C	−5.368430	−0.230470	−7.058780
O	−5.883630	0.834840	−6.260780
H	−5.270560	1.607180	−6.335460
H	−5.168590	0.121160	−8.106980
H	−6.143330	−1.025280	−7.109850
H	−4.230910	−1.060770	−5.376180
H	−2.725030	−3.811650	−7.311510
C	−1.479010	−2.028640	−6.939790
O	−0.352220	−2.796030	−6.465790
H	0.431210	−2.189600	−6.376190
H	−1.357860	−1.836700	−8.044590
C	−1.570750	−0.690930	−6.207720
O	−0.396430	0.078520	−6.558510
H	−0.563260	1.034910	−6.304080
H	−1.595360	−0.862830	−5.095450
C	−2.840650	0.057630	−6.634790
H	−2.747040	0.394920	−7.701080
O	−2.966030	1.242390	−5.756420
C	−2.950650	2.530880	−6.440380
O	−4.243300	3.178720	−6.227150
C	−4.557250	3.372560	−4.787980

C	−5.967500	3.969390	−4.784800
O	−6.559390	3.912270	−3.486700
H	−6.094130	4.561890	−2.902740
H	−5.932510	5.028450	−5.157290
H	−6.609410	3.386320	−5.479700
H	−4.551090	2.370630	−4.287360
H	−2.844510	2.388330	−7.549250
C	−1.852860	3.423810	−5.872400
O	−0.585180	2.767720	−6.086940
H	0.102180	3.211120	−5.520420
H	−1.861390	4.404230	−6.429980
C	−2.078360	3.692990	−4.385600
O	−1.074280	4.642140	−3.954930
H	−1.366520	5.024570	−3.073810
H	−1.971740	2.735300	−3.802100
C	−3.482330	4.269980	−4.159620
H	−3.544470	5.308240	−4.581030
O	−3.700410	4.321230	−2.697200
C	−3.972330	5.647050	−2.149840
O	−5.322120	5.642480	−1.591530
C	−5.495170	4.615040	−0.531000
C	−6.979030	4.689160	−0.163360
O	−7.392720	3.530770	0.565530
H	−6.974470	3.570180	1.462120
H	−7.181810	5.620380	0.430050
H	−7.584360	4.741820	−1.094040
H	−5.246500	3.612640	−0.964920
H	−3.966860	6.418070	−2.966650
C	−2.976180	5.981540	−1.044520
O	−1.651410	5.951890	−1.619200
H	−0.984940	5.924820	−0.880850
H	−3.192720	7.022630	−0.668280
C	−3.085060	4.996930	0.118850
O	−2.214820	5.468870	1.176060
H	−2.469290	4.988970	2.020420
H	−2.758470	3.972950	−0.213820
C	−4.533080	4.927070	0.622360
H	−4.817540	5.892130	1.121090
O	−4.596930	3.827070	1.603030
C	−5.021600	4.198210	2.943760

O	−6.287800	3.525920	3.229420
C	−6.196630	2.040740	3.139150
C	−7.622940	1.571700	3.441700
O	−7.822690	0.205120	3.077300
H	−7.289250	−0.360060	3.688590
H	−7.852160	1.725690	4.531540
H	−8.340080	2.188200	2.857140
H	−5.890810	1.759500	2.093340
H	−5.226230	5.301710	3.005070
C	−3.980630	3.752450	3.965420
O	−2.737250	4.424010	3.662720
H	−2.003310	3.953500	4.140160
H	−4.325120	4.058410	4.995020
C	−3.797470	2.237230	3.924160
O	−2.877560	1.874380	4.984130
H	−2.945730	0.883570	5.122950
H	−3.370310	1.932380	2.929780
C	−5.145000	1.535660	4.134590
H	−5.494610	1.679150	5.192030
O	−4.925820	0.090460	3.886280
C	−5.206370	−0.788740	5.012830
O	−6.327310	−1.658230	4.654060
H	−5.537980	−0.195200	5.906660
C	−3.992090	−1.657180	5.321830
O	−2.895350	−0.786090	5.669830
H	−2.052620	−1.313810	5.641400
H	−4.234650	−2.318370	6.203070
C	−3.625970	−2.534670	4.125580
O	−2.551990	−3.411760	4.541540
H	−2.483080	−4.156450	3.872650
H	−3.282100	−1.890750	3.267910
C	−4.841990	−3.361770	3.687360
H	−5.080340	−4.135130	4.464980
C	−6.061920	−2.465370	3.435960
H	−5.847330	−1.765630	2.587970
C	−7.369280	−3.224920	3.192940
H	−7.498980	−4.017200	3.978830
H	−8.221530	−2.517380	3.280850
O	−7.409580	−3.801060	1.887180
H	−6.757250	−4.543580	1.852480

O	6.534620	−5.032170	0.048940
H	5.815050	−5.530900	0.508960
C	6.491740	−5.347110	−1.342080
H	6.547120	−6.458890	−1.498960
H	7.385060	−4.892980	−1.822240
C	5.229140	−4.795400	−2.011040
H	5.076290	−3.718860	−1.743450
O	5.518400	−4.879450	−3.465960
C	4.441370	−4.328630	−4.284940
O	4.217610	−2.945650	−3.876740
H	4.792910	−4.374410	−5.350530
C	3.182420	−5.159350	−4.061060
O	2.128640	−4.604410	−4.878880
H	1.257900	−4.961330	−4.558020
H	3.385330	−6.219410	−4.387060
C	2.779010	−5.152440	−2.588170
O	1.670550	−6.071570	−2.430630
H	1.543760	−6.231540	−1.447860
H	2.455870	−4.117560	−2.289950
C	3.952990	−5.589000	−1.700600
H	4.136830	−6.689750	−1.818990
O	3.563030	−5.304020	−0.301390
C	3.504200	−6.463100	0.580660
O	4.509110	−6.292170	1.628200
C	4.312380	−5.047940	2.417000
C	5.503240	−5.019830	3.379430
O	5.651080	−3.743040	3.999550
H	4.899690	−3.612040	4.629010
H	5.382180	−5.824760	4.154050
H	6.433550	−5.233930	2.810690
H	4.347900	−4.175130	1.716100
H	3.780060	−7.398580	0.023660
C	2.128500	−6.568400	1.228540
O	1.147890	−6.728220	0.181160
H	0.246600	−6.557370	0.566650
H	2.111770	−7.479970	1.892880
C	1.822240	−5.331970	2.072540
O	0.563540	−5.568230	2.746570
H	0.487690	−4.912840	3.502660
H	1.736940	−4.424870	1.411210

C	2.939680	−5.110660	3.100560
H	2.924540	−5.932440	3.864910
O	2.673580	−3.817380	3.769550
C	2.489750	−3.893670	5.214830
O	3.560220	−3.133070	5.855670
C	3.580980	−1.702750	5.453890
C	4.806640	−1.132860	6.173980
O	5.150830	0.164130	5.688760
H	4.456500	0.803210	5.983600
H	4.613300	−1.102930	7.280260
H	5.674030	−1.805250	5.999590
H	3.710360	−1.646890	4.342730
H	2.598690	−4.953690	5.569930
C	1.147090	−3.295870	5.620900
O	0.102310	−4.047820	4.969350
H	−0.745080	−3.529630	5.029630
H	1.033790	−3.397660	6.738840
C	1.062360	−1.817110	5.247700
O	−0.182700	−1.306250	5.780020
H	−0.138030	−0.303670	5.770880
H	1.074770	−1.698270	4.127560
C	2.248290	−1.055160	5.853460
H	2.151960	−1.019100	6.970990
O	2.204840	0.322050	5.314590
C	2.040250	1.377670	6.308840
O	3.239550	2.211760	6.293790
C	3.511300	2.803900	4.958190
C	4.821300	3.574210	5.150130
O	5.417260	3.927300	3.902560
H	4.845850	4.601690	3.459160
H	4.632810	4.490950	5.772730
H	5.541290	2.932730	5.702510
H	3.645180	1.970470	4.222320
H	1.968140	0.940130	7.340650
C	0.834360	2.247090	5.971270
O	−0.344580	1.414900	6.000630
H	−1.090580	1.908060	5.563640
H	0.739660	3.051290	6.756300
C	0.997830	2.901240	4.600960
O	−0.118350	3.803540	4.412250

H	0.095580	4.399590	3.634160
H	0.989370	2.113040	3.797330
C	2.321190	3.676470	4.536700
H	2.260720	4.594590	5.179450
O	2.519670	4.077470	3.126570
C	2.597460	5.512410	2.887660
O	3.925390	5.814700	2.355790
C	4.229220	5.055870	1.115110
C	5.672120	5.443730	0.778960
O	6.244700	4.563060	−0.187280
H	5.799770	4.719060	−1.056600
H	5.703410	6.506170	0.414130
H	6.287380	5.384400	1.702370
H	4.155810	3.961350	1.342300
H	2.500790	6.082050	3.850600
C	1.550250	5.933960	1.863300
O	0.247150	5.635900	2.407070
H	−0.425470	5.688580	1.675150
H	1.635780	7.045740	1.693120
C	1.766250	5.207110	0.536730
O	0.819500	5.747290	−0.415580
H	1.124660	5.492420	−1.336650
H	1.586220	4.103710	0.669320
C	3.200730	5.432920	0.040160
H	3.334830	6.503940	−0.267670
O	3.398590	4.558240	−1.137280
C	3.748120	5.248600	−2.372990
O	5.078590	4.806970	−2.785650
C	5.161460	3.339000	−2.998980
C	6.634760	3.094560	−3.338350
O	6.966850	1.707740	−3.286420
H	6.525040	1.252240	−4.044530
H	6.863410	3.518500	−4.353450
H	7.269870	3.626720	−2.597940
H	4.882890	2.823880	−2.043720
H	3.820210	6.356040	−2.200460
C	2.751970	4.907990	−3.475680
O	1.443580	5.343710	−3.049860
H	0.761320	4.919440	−3.636240
H	3.042850	5.469210	−4.410040

C	2.753450	3.409770	−3.774400
O	1.877010	3.192670	−4.906200
H	2.067810	2.279780	−5.275870
H	2.372060	2.838710	−2.882640
C	4.177040	2.943280	−4.108010
H	4.502500	3.378640	−5.089990
O	4.145480	1.466870	−4.215570
C	4.528810	0.931460	−5.517570
O	5.748310	0.143790	−5.352900
H	4.783100	1.764450	−6.226680
C	3.430780	0.030440	−6.071630
O	2.234560	0.822670	−6.222830
H	1.461820	0.210990	−6.358780
H	3.753750	−0.349120	−7.083730
C	3.180230	−1.162000	−5.150330
O	2.214270	−2.021960	−5.800980
H	2.243270	−2.919070	−5.352060
H	2.769540	−0.806940	−4.163200
C	4.490470	−1.923900	−4.911490
H	4.818380	−2.431850	−5.857020
C	5.590390	−0.984190	−4.398880
H	5.295090	−0.568060	−3.401170
C	6.985570	−1.613120	−4.333910
H	7.209560	−2.135300	−5.303220
H	7.739210	−0.807350	−4.201090
O	7.109520	−2.515850	−3.236050
H	6.563880	−3.318280	−3.425750

References

(1) Ferro-Costas, D.; Fernández-Ramos, A. New computational tools for chemical kinetics: the Cathedral Package. *Theor. Chem. Acc.* **2023**, *142*, 76.

(2) Ferro-Costas, D.; Fernández-Ramos, A. A Combined Systematic-Stochastic Algorithm for the Conformational Search in Flexible Acyclic Molecules. *Front. Chem.* **2020**, *8*, 16.

(3) Ferro-Costas, D.; Mosquera-Lois, I.; Fernández-Ramos, A. TorsiFlex, An Automatic Generator of Structural Data Sets: Conformational Search of the Twenty Proteinogenic Amino Acids. *J. Cheminformatics* **2021**, *13*, 100.

(4) Ríos, M. A.; Rodríguez, J. Semiempirical study of compounds with O–H$\cdots$O intramolecular hydrogen bond. *J. Comput. Chem.* **1992**, *13*, 860–866.

(5) Zhao, Y.; Truhlar, D. G. Hybrid meta density functional theory methods for thermochemistry, thermochemical kinetics, and noncovalent interactions: the MPW1B95 and MPWB1K models and comparative assessments for hydrogen bonding and van der Waals interactions. *J. Phys. Chem. A* **2004**, *108*, 6908–6918.

(6) Hehre, W. J.; Ditchfield, R.; Pople, J. A. Self-consistent molecular orbital methods. XII. Further extensions of Gaussian-type basis sets for use in molecular orbital studies of organic molecules. *J. Chem. Phys.* **1972**, *56*, 2257–2261.

(7) Meana-Pañeda, R.; Fernández-Ramos, A. Tunneling and conformational flexibility play critical roles in the isomerization mechanism of vitamin D. *J. Am. Chem. Soc.* **2012**, *134*, 346–354.

(8) Frisch, M. J.; Trucks, G. W.; Schlegel, H. B.; Scuseria, G. E.; Robb, M. A.; Cheeseman, J. R.; Scalmani, G.; Barone, V.; Mennucci, B.; Petersson, G. A.;

Nakatsuji, H.; Caricato, M.; Li, X.; Hratchian, H. P.; Izmaylov, A. F.; Bloino, J.; Zheng, G.; Sonnenberg, J. L.; Hada, M.; Ehara, M.; Toyota, K.; Fukuda, R.; Hasegawa, J.; Ishida, M.; Nakajima, T.; Honda, Y.; Kitao, O.; Nakai, H.; Vreven, T.; Montgomery, J. A., Jr.; Peralta, J. E.; Ogliaro, F.; Bearpark, M.; Heyd, J. J.; Brothers, E.; Kudin, K. N.; Staroverov, V. N.; Kobayashi, R.; Normand, J.; Raghavachari, K.; Rendell, A.; Burant, J. C.; Iyengar, S. S.; Tomasi, J.; Cossi, M.; Rega, N.; Millam, J. M.; Klene, M.; Knox, J. E.; Cross, J. B.; Bakken, V.; Adamo, C.; Jaramillo, J.; Gomperts, R.; Stratmann, R. E.; Yazyev, O.; Austin, A. J.; Cammi, R.; Pomelli, C.; Ochterski, J. W.; Martin, R. L.; Morokuma, K.; Zakrzewski, V. G.; Voth, G. A.; Salvador, P.; Dannenberg, J. J.; Dapprich, S.; Daniels, A. D.; Farkas, O.; Foresman, J. B.; Ortiz, J. V.; Cioslowski, J.; Fox, D. J. Gaussian 09 Revision E.01. 2009; Gaussian Inc. Wallingford CT.

(9) Dapprich, S.; Komáromi, I.; Byun, K. S.; Morokuma, K.; Frisch, M. J. A new ONIOM implementation in Gaussian98. Part I. The calculation of energies, gradients, vibrational frequencies and electric field derivatives. *J Mol Struct Theochem* **1999**, *461*, 1–21.

(10) Zheng, G.; Witek, H. A.; Bobadova-Parvanova, P.; Irle, S.; Musaev, D. G.; Prabhakar, R.; Morokuma, K.; Lundberg, M.; Elstner, M.; Köhler, C.; Frauenheim, T. Parameter calibration of transition-metal elements for the spin-polarized self-consistent-charge density-functional tight-binding (DFTB) method: Sc, Ti, Fe, Co, and Ni. *J. Chem. Theory Comput.* **2007**, *3*, 1349–1367.

(11) Ferro-Costas, D.; Truhlar, D. G.; Fernández-Ramos, A. Pilgrim: A thermal rate constant calculator and a chemical kinetics simulator. *Comput. Phys. Commun.* **2020**, *256*, 107457.

(12) Alecu, I. M.; Zheng, J.; Zhao, Y.; Truhlar, D. G. Computational thermochemistry:

scale factor databases and scale factors for vibrational frequencies obtained from electronic model chemistries. *J. Chem. Theory Comput.* **2010**, *6*, 2872–2887.
